# Research on chemical resistance characteristics of water-immersed coal with different metamorphic degrees

**DOI:** 10.1038/s41598-022-17865-x

**Published:** 2022-08-12

**Authors:** Xun Zhang, Mengfan Zhao, Jing Yang, Bing Lu, Gang Wang, Fengwei Dai

**Affiliations:** 1 College of Mining, Liaoning Technical University, Fuxin, 123000 Liaoning China; 2 China Coal Technology & Engineering Group, Shenyang Research Institute Co. Ltd, Fushun, 113122 Liaoning China; 3 College of Safety Science and Engineering, Liaoning Technical University, Huludao, 125105 Liaoning China

**Keywords:** Energy science and technology, Fossil fuels, Coal

## Abstract

In order to reduce the risk of spontaneous combustion of coal left after long-term flooding in the goaf of the mine, in this paper, the inhibitory properties of different inhibitors on two kinds of water-immersed coals with different metamorphic degrees were studied in depth. The experiment selected Pingzhuang brown coal and Shaqu coking coal as research objects. The raw coal and water-immersed coal samples were compared and analyzed by thermogravimetric experiment method and Fourier transform infrared spectroscopy experiment method. The study showed that the activation temperature of brown coal and coking coal decreased by 7.91 and 2.25 °C respectively, and the activation energy decreased by 43.18 kJ/mol and 20.58 kJ/mol respectively. The natural tendency of coal was enhanced after water immersion, and water immersion had a greater impact on low-metamorphic brown coal. After adding four kinds of inhibitors, MgCl_2_, TEMPO, TPPI and PA to the two water-immersed coals, it was found that TPPI could significantly reduce the heat release rate of water-immersed brown coal, and the reduction value was 10.49 W/mg. The dry cracking temperature of water-immersed brown coal increased by 11.75 °C, and PA greatly increased the combustion activation energy of water-immersed coking coal by 25.77 kJ/mol. Meanwhile, it was found from the microscopic active groups that TTPI increased the content of water-immersed brown coal ether bonds by 4.84%. The absorption peak intensity of oxygen-containing functional groups such as C=O was significantly weakened. Similarly, PA also produced a large number of stable ethers in water-immersed coking coal, whose content increased by 5.21%, and the hydroxyl content decreased most significantly. The decomposition of TPPI into phosphoric acid after heating can inhibit the growth of active groups such as a large number of oxygen-containing functional groups in the water-immersed brown coal, thereby reducing the risk of spontaneous combustion. As a metal chelator, PA can reduce the catalytic effect of metal ions in water-immersed coking coal with fewer active groups, and inhibit coal spontaneous combustion by generating stable metal complexes to increase activation energy. This indicated that TTPI had the best inhibitory effect on water-immersed brown coal, while PA was more suitable for water-immersed coking coal.

## Introduction

Coal spontaneous combustion is an important disaster-causing factor for the frequent occurrence of mine disasters, among which water is an important factor affecting coal spontaneous combustion^1–4^. Xu et al.^5^ used TA-DSC to test the exothermic properties of four different grades of coal with different total moisture content during the oxidation process, and found that different grades of coal had different critical moisture content, and for gas coal and fat coal, the lower the moisture, the greater the chance of spontaneous combustion of coal. BHAT et al.^6^ proved through experiments that water in coal can promote the generation of free radicals and peroxide complexes. Qin et al.^7^ found that long-flame coal samples that had been immersed in water for a long time had more developed pores, increased concentration of free radicals in coal, decreased activation energy and crosspoint temperature, and higher gas production and production rate than raw coal, showed higher spontaneous combustion tendency. Wen, Deng and others^8,9^ used temperature-programmed and infrared spectroscopy experiments to compare and analyze raw coal and water-immersed coal, and showed that the oxygen consumption rate and CO and CO_2_ generation rate of immersed coal samples were higher than those of raw coal samples, the content of active groups mostly increased, and the characteristic temperatures were all reduced, so the immersed coal was more prone to spontaneous combustion. Nandy et al.^10^ studied the influence of moisture on the change of the critical oxidation temperature of coal on the self-heating characteristics, and indicated that the self-heating characteristics of coal were affected by the moisture in the coal and the humidity of the air, and the moisture in the coal played a role in the spontaneous combustion process. Song et al.^11^ studied the influence of water immersion on physical and chemical structure and spontaneous combustion characteristics of coal through pore structure test, infrared spectrum test, coal spontaneous combustion characteristics and TG-DSC experiment, and showed that water immersion promotes coal spontaneous combustion. Qiao et al.^12^ studied immersed coal samples of different metamorphic degrees and found that the active groups of immersed coal increased and the oxidation activity was higher than that of raw coal, which indicated that immersed coal was more prone to spontaneous combustion from the perspective of heat change.

In order to effectively inhibit the spontaneous combustion of coal and prolong its spontaneous combustion period, scholars have developed different chemical inhibitors from physical and chemical perspectives to inhibit the spontaneous combustion of coal. Li et al.^13^ used temperature-programmed oxidation and Fourier transform infrared spectroscopy and found that the use of sodium persulfate inhibitor with a mass fraction of 5% in the low temperature stage had the best effect. The sodium persulfate inhibitor could promote the active groups in coal to form relatively stable ethers, alkyls and carboxylic acids, which could effectively prevent coal spontaneous combustion. Sun et al.^14^ selected sodium bicarbonate as the physical resistive component and tea polyphenol, a highly effective antioxidant, as the chemical resistive component to carry out the experimental study on the optimization of composite resistive agent. Thermogravimetric differential scanning calorimetry experiment and in-situ Fourier infrared spectroscopy experiment were used to verify the high efficiency of compound inhibitors on the inhibition of spontaneous combustion of coal. Sun et al.^15^ compared and analyzed the characteristic temperature points of coal spontaneous combustion of raw coal samples and four kinds of CEPPA inhibited coal samples by TG-DTG, and found that the inhibition effect was the best when the CEPPA inhibitor concentration was 20%. Guo et al.^16^ used quantum chemistry, electron paramagnetism, thermogravimetry, infrared and other methods to verify that the natural antioxidant catechin has a good effect on inhibiting spontaneous combustion of coal. Gao et al.^17^ used thermal analysis, infrared spectroscopy experiments, low temperature oxidation experiments and intersection temperatures to prove that the metal chelating agent EDTA has an inhibitory effect on coal spontaneous combustion, and can play a good inhibitory effect on different coal samples. Li et al.^18^ used thermal analysis, infrared spectroscopy experiments, low-temperature oxidation experiments and cross-point temperature to prove that the metal chelating agent EDTA has an inhibitory effect on coal spontaneous combustion, and can exert a good inhibitory effect on different coal samples.

The above studies show that water-immersed coal is easier to spontaneous combustion than raw coal, and the application of coal spontaneous combustion inhibitors is mostly aimed at the spontaneous combustion of raw coal, and the research on the resistance characteristics of immersed coal is relatively few. Therefore, this paper mainly carries out the research on the resistance of immersed coal with different metamorphic degrees, analyzes the characteristic temperature, exothermic characteristics and reaction activation energy of immersed coal before and after the resistance, and obtains the influence of different inhibitors on the organic structure of coal through infrared spectrum test.

## Experimental Materials and Methods

### Coal sample processing

Pingzhuang brown coal and Shaqu coking coal were used as raw materials, ground to 150–250 mesh, and used after vacuum degassing. Take 20 g of each coal sample and put it into a sand bottle, stir evenly according to the coal-water ratio of 1:1, and soak it for 15 days after sealing. The raw coal of the same quality was sealed in a frosted bottle and placed in the same environment for 15 days as a control sample. After 15 days, the raw coal and the immersed coal samples were dried to constant weight, and when the water content was guaranteed to be 2%, they were used as experimental coal samples. According to GB/T 212-2008 and GB/T 31391-2015, industrial analysis and elemental analysis of coal samples were carried out in this experiment. The results are shown in Table 1. According to GB5751-86 classification standard in China, brown coal belongs to low metamorphic coal, and coking coal belongs to high metamorphic coal. With the deepening of coal metamorphism, carbon element increases, oxygen element, volatile matter and moisture decrease, and the spontaneous combustion tendency of coal decreases.

**Table 1:** Industrial analysis and elemental analysis of raw coal.

Industrial analysis (%)					Elemental analysis (%)				
Coal	Moisture	Volatile matter	Ash	Fixed carbon	C	H	O	N	S
Brown coal	8.36	30.84	20.26	40.54	69.22	3.87	24.67	1.06	1.18
Coking coal	1.48	20.75	9.67	68.10	86.51	5.52	5.79	1.72	0.46

The proportions of the four inhibitor solutions selected in the experiment are shown in Table 2. In order to ensure the accuracy of the experimental data, immersed coal and distilled water were used as the experimental control group. The proportion of other inhibitors and coal samples was mixed at 1:3, and the samples were evenly stirred. The prepared samples were sealed and stored in room temperature for 24 h for use.

**Table 2:** Proportion of inhibitor solution.

Drug name	Dosage	Solvent (mL)	Concentration (%)
MgCl_2_	3 g	Water 27	10
Tetramethylpiperidine nitroxid (TEMPO)	2.63 g	Ethanol 30	10
Triphenyl phosphite (TPPI)	2.22 ml	Ethanol 30	10
Phytic acid (PA)	1.1 mL	Ethanol 30	10

### Experimental procedure

#### Thermal analysis experimental method

The experiment adopts non-isothermal thermogravimetric method. The reaction gas was oxygen with a flow rate of 10 mL/min, and the protective gas was nitrogen with a flow rate of 40 mL/min. The heating rate was 5 °C/min, and the reaction temperature was 30–800 °C. The sample was loaded in an alumina dry pot, with a mass of 10 mg. The experimental instrument was STA449C integrated thermogravimetric analyzer.

#### Fourier infrared spectroscopy experimental method

**Figure 1:**
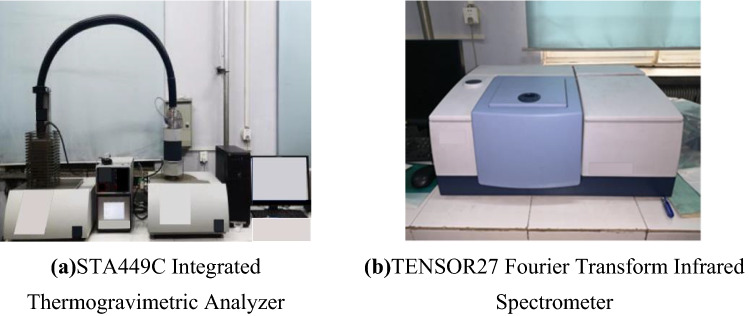
Experimental instrument.

Dry potassium bromide in a drying oven at 110 °C for 4 h. The coal sample and potassium bromide were ground in a mortar in a ratio of 1:180. After grinding, and the tablets were pressed by HY-15 tablet press at a pressure of 10 MPa to produce flake samples with good light transmitance and uniformity. The experiment was performed with a German TENSOR27 Fourier transform infrared spectrometer. The experimental setup is shown in Fig. 1.

## Experimental results and discussion

### Analysis on oxidation spontaneous combustion characteristics of water-immersed coal

**Figure 2:**
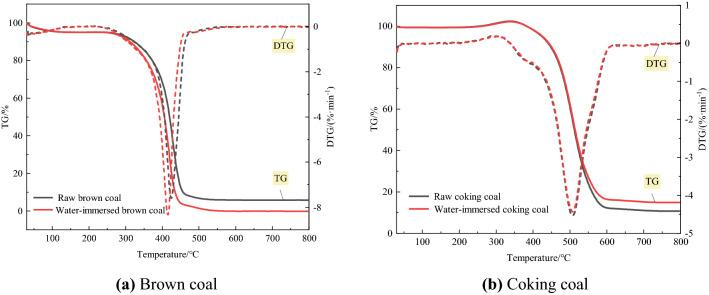
Thermal analysis curves of raw coal samples and water-immersed coal samples immersion.

The TG curve reflects the change of coal weight in the process of coal oxidative heating. According to the change process of coal sample weight, four characteristic temperature points in the oxidation process were determined and analyzed, including drying temperature (T1), activation temperature (T2), ignition temperature (T3) and maximum weight loss rate temperature (T4). The TG-DTG curves of raw coal and water-immersed coal are shown in Fig. 2. The characteristic temperature is shown in Table 3. The Coats–Redfern integral method^19^ was used to fit and calculate each stage of the TG curve, and the most probable mechanism function with better linear correlation was selected, and the reaction kinetic parameters were calculated to obtain the oxidation kinetic parameters in Table 4.

**Table 3:** Auto-ignition temperature characteristics of brown coal and coking coal before and after water-immersed.

Sample name	T_1_ (°C)	T_2_ (°C)	T_3_ (°C)	T_4_ (°C)
Raw brown coal	178.61	227.61	389.61	424.61
Water-immersed brown coal	168.70	219.70	379.70	415.70
Raw coking coal	177.80	338.80	455.80	509.80
Water-immersed coking coal	160.55	336.55	456.55	505.55

**Table 4:** Fitting parameters of brown coal and coking coal oxidation kinetics before and after water-immersed.

Coal sample	Reaction stage	lnA (min)	E (kJ/mol)	R^2^
Raw brown coal	Combustion stage	55.21	328.07	0.9603
Water-immersed brown coal	Combustion stage	48.56	284.89	0.9972
Raw coking coal	Combustion stage	35.33	242.10	0.9365
Water-immersed coking coal	Combustion stage	32.16	221.52	0.9809

By analyzing the characteristic temperature on TG curve and calculating the activation energy, it can be seen that the characteristic temperature of water-immersed brown coal is 9.91 °C, 7.91 °C, 9.91 °C and 8.91 °C lower than that of raw brown coal, and the characteristic temperature of water-immersed coking coal is 17.25 °C, 2.25 °C, − 0.75 °C and 3.45 °C lower than that of raw coking coal, respectively. At the same time, in the combustion stage, the activation energy of raw brown coal was 328.07 kJ/mol, and the activation energy after immersion was 284.89 kJ/mol, which decreased by 43.18 kJ/mol. The activation energy of coking coal decreased by 20.58 kJ/mol after immersion in water. The activation temperature was ranked from low to high as water-immersed brown coal < raw brown coal < water-immersed coking coal < raw coking coal, and the activation energy in the combustion stage showed the same trend. This showed that the water-immersed coal was the most likely to spontaneously ignite among the coal samples with the same degree of metamorphism. Among the coal samples with different metamorphic degrees, the water-immersed brown coal with a lower degree of metamorphism was the most likely to spontaneously ignite.

**Figure 3:**
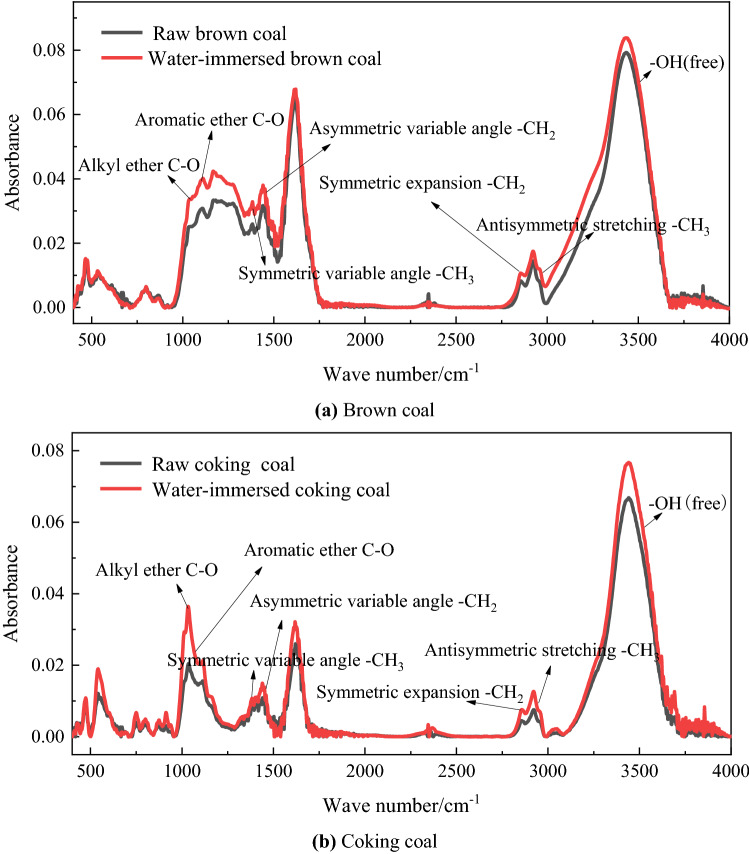
Infrared spectral curves of raw coal samples and water-immersed coal samples.

The infrared spectrum showed the change of functional group content and the change of absorption peak intensity after coal was soaked in water, and the group changes of coal before and after immersion can be analyzed from the microscopic perspective. The infrared spectra of raw coal and water-immersed coal are shown in Fig. 3 below.

After water immersion treatment, the vibration shrinkage absorption peaks of aryl ethers (1100.36 and 1105.64 cm^−1^) and alkyl ethers (1031.7 and 11,031.43 cm^−1^) in brown coal and coking coal were enhanced, and their contents increased to different degrees. Yang et al.^20^ found that alkyl ethers and aryl ethers are relatively active groups in coal and are easily oxidized. Therefore, the content of aryl ether and alkyl ether increases, which improves the oxidation activity of coal. After immersion in brown coal and coking coal, the –OH (3605, 3611 cm^−1^) stretching vibration absorption peaks were enhanced, and the contents of –CH_3_ and –CH_2_ (at 1300–1500 and 2800–3000 cm^−1^) were increased in varying degrees, and the vibration absorption peak was enhanced. Studies by Shi^21^, Chu et al.^22^ showed that hydroxyl group, methyl group and methylene group were active groups in coal, which first reacted with oxygen at low temperature. It can be considered that the active groups of the water-immersed coal were increased, and the oxidation activity was higher than that of the raw coal. After the coal sample was immersed in water, the coal sample had more cracks and holes due to swelling, which made the adsorption capacity of oxygen enhanced and more prone to oxidation reaction, and was more prone to spontaneous combustion than raw coal.

### Analysis of thermal characteristics of water-immersed coal after adding inhibitors

#### Characteristic temperature and exothermic parameters

##### Characteristic temperature

**Figure 4:**
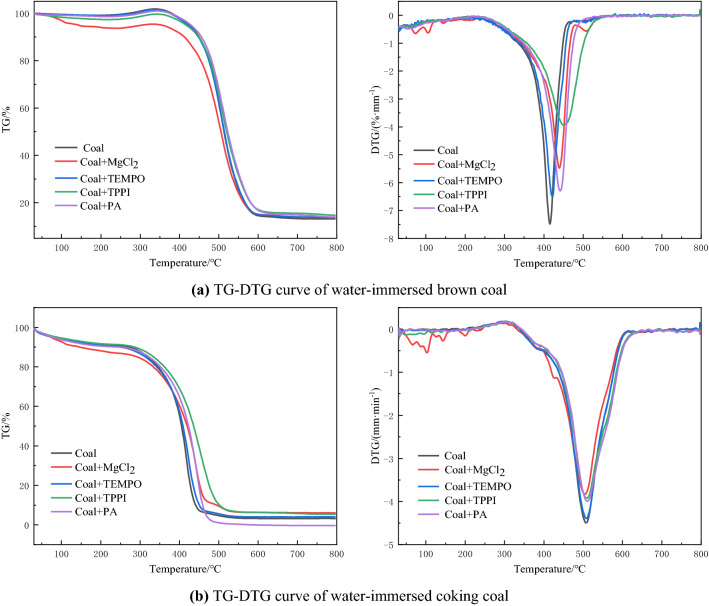
Thermal analysis curves of water-immersed coal samples with different degrees of metamorphism.

Figure 4 and Table 5 show the simultaneous thermal analysis curves and characteristic temperatures of water-immersed brown coal and water-immersed coking coal after adding inhibitors.

**Table 5:** Characteristic temperature of water-immersed brown coal and coking coal after adding inhibitors.

Sample name	Inhibitor	T_1_ (°C)	T_2_ (°C)	T_3_ (°C)	T_4_ (°C)
Water-immersed brown coal	Coal	177.18	280.18	366.18	404.18
	MgCl_2_	202.92	315.92	390.92	439.92
	TEMPO	175.47	311.47	381.47	421.47
	TPPI	188.93	326.93	396.93	450.93
	PA	184.69	325.69	390.69	442.69
Water-immersed coking coal	Coal	160.50	336.50	452.50	505.50
	MgCl_2_	245.99	333.99	443.99	504.99
	TEMPO	204.26	340.26	454.26	507.26
	TPPI	218.57	343.57	454.57	509.57
	PA	218.99	345.33	456.33	510.33

Compared with other inhibitors, the dry-cracking temperature, activation temperature and ignition temperature of water-immersed brown coal increased by 25.74 °C, 35.74 °C and 24.74 °C respectively after adding MgCl_2_, and in the range of 375–500 °C, the weight loss rate decreased, and the temperature at the point of maximum weight loss rate increased by 35.74 °C. Because MgCl_2_ had good moisture absorption and water retention, it had the best inhibitory effect on brown coal immersed in water at low temperature, however, the inhibitory effect of MgCl_2_ gradually weakened with the increase of temperature. After adding TEMPO, the dry cracking temperature of water-immersed brown coal decreased, the activation temperature increased by 31.29 °C, the ignition temperature increased by 15.29 °C, and the temperature at the point of maximum weight loss rate increased by 17.29 °C. The main reason was that TEMPO was a chemical free radical scavenger, and the free radicals were exposed more at high temperature, as a result, TEMPO had poor inhibitory effect on water-immersed brown coal at dry cracking temperature, and its inhibitory effect increased with the increased of temperature. After adding TPPI inhibitor, the weight loss rate of water-immersed brown coal did not change significantly from 30 to 250 °C, but the dry cracking temperature increased by 11.75 °C. From 250 to 500 °C, the weight loss rate of water-immersed brown coal slows down significantly, the activation temperature increased by 46.75 °C, the ignition temperature increased by 30.75 °C, and the temperature at the point of maximum weight loss rate increased by 46.75 °C. The results showed that TPPI had the best inhibitory effect on the whole oxidative spontaneous combustion process of water-immersed brown coal.

MgCl_2_ had the greatest influence on the dry-cracking temperature point of water-immersed coking coal, and the dry cracking temperature increased by 85.49 °C, but had little effect on other characteristic temperatures. The inhibitory effect of MgCl_2_ on coal spontaneous combustion decreased with the increase of temperature. The activity temperature of coal was ranked from high to low as follows: water-immersed coking coal + PA > water-immersed coking coal + TPPI > water-immersed coking coal + TEMPO > water-immersed coking coal > water-immersed coking coal + MgCl_2_. The inhibitory effect of TEMPO and TPPI on water-immersed coking coal was average.

Comprehensive analysis shows that PA is most suitable for the spontaneous combustion of water-immersed coking coal, and TPPI is more suitable for water-immersed brown coal. Comparing the changes of the characteristic temperature and weight loss rate of the two coal samples after water immersion, it can be seen that the inhibitory effect of the inhibitors on the water-immersed brown coal are greater than that of the water-immersed coking coal. The inhibition effect of chemical inhibitors are better than that of physical inhibitors for water-immersed coals with different metamorphic degrees.

##### Thermal parameters

**Figure 5:**
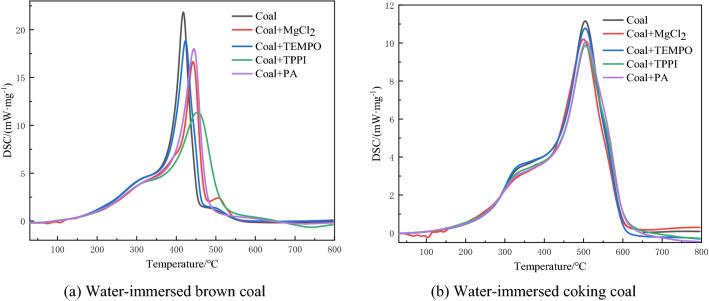
Thermal characteristic curves of water-immersed coal samples with different metamorphic degrees.

DSC curves show endothermic/exothermic versus temperature. Figure 5 and Table 6 show the DSC curves and auto-ignition exotherm parameters of water-immersed brown coal and water-immersed coking coal after adding inhibitors.

**Table 6:** Spontaneous combustion and exothermic parameters of water-immersed brown coal and water-immersed coking coal after adding inhibitors.

Sample name	Inhibitor	Endothermic peak area (J/g)	Maximum exothermic temperature (°C)	ΔT _max_ (°C)	Maximum heat release rate (mW/mg)
Water-immersed brown coal	Coal	− 1322	406.8	0	21.83
	MgCl_2_	− 1577	442.3	35.5	16.64
	TEMPO	− 1442	423.2	16.4	18.84
	TPPI	− 1507	452.8	46	11.34
	PA	− 1479	444.4	37.6	18.01
Water-immersed coking coal	Coal	− 1631	503.2	0	11.16
	MgCl_2_	− 1738	499.9	− 3.3	10.19
	TEMPO	− 1721	504.2	1	10.78
	TPPI	− 1713	504.8	1.6	9.978
	PA	− 1738	506.5	3.3	10.02

After soaking, the water-immersed brown coal after adding MgCl_2_ and TPPI inhibitors in the low temperature stage increased by 255 J/g and 185 J/g in the water evaporation and desorption stages, verified that MgCl_2_ and TPPI reduced the coal temperature by endothermic in the initial stage of oxidation. At the same time, the occurrence of oxidative spontaneous combustion of water-immersed brown coal was delayed, and the endothermic increased was not large after adding other inhibitors. In the high temperature stage, the maximum exothermic peak temperature of DSC of coal samples with different inhibitors added in descending order was: water-immersed brown coal + TPPI > water-immersed brown coal + PA > water-immersed brown coal + MgCl_2_ > water-immersed brown coal + TEMPO > water-immersed brown coal. Among them, the maximum heat release rate of the coal sample suppressed by TPPI decreased the most, which was 10.49 W/mg. Therefore, TPPI had the best inhibitory effect on the whole oxidation spontaneous combustion stage of water-immersed brown coal.

In the low temperature stage, the heat absorption of water-immersed coking coal increased after adding MgCl_2_, PA, TPPI and TEMPO, and the increased of heat absorption of MgCl_2_ and PA was 107 J/g at most, which indicated that the temperature of coal body decreased and effectively delayed the occurrence of water loss and weight loss. After the addition of MgCl_2_, the maximum exothermic peak temperature and the maximum heat release rate of immersed coking coal decreased, which indicated that MgCl_2_ played a role in promoting the combustion of immersed coking coal to a certain extent in the high temperature stage. After adding PA inhibitor, the maximum exothermic peak temperature of water-immersed coking coal was delayed by 3.3 °C, and the maximum exothermic rate was reduced by 1.14 mW/mg, which had a good inhibitory effect at high temperature. The parameters of the effects of TPPI and TEMPO on water-immersed coking coal were second only to PA inhibitor, and the inhibitory effect was not as good as that of PA.

#### Reaction kinetic parameters

The kinetic theory of chemical reaction points out that activation energy represents the minimum energy required for ordinary molecules to be activated into high-energy activated molecules, and can be used as an important indicator for judging the difficulty of the reaction. The oxidation kinetic parameters of water-immersed brown coal and coking coal after adding inhibitors are shown in Table 7.

**Table 7:** Fitting parameters of oxidation kinetics of water-immersed brown coal and water-immersed coking coal after adding inhibitors.

Sample name	Inhibitor	E (kJ/mol)	lnA (min)	R^2^
Water-immersed brown coal	Coal	205.79	32.42	0.9437
	MgCl_2_	296.98	50.31	0.9559
	TEMPO	285.21	48.13	0.955
	TPPI	326.22	55.52	0.9586
	PA	303.78	51.94	0.9609
Water-immersed coking coal	Coal	216.59	32.32	0.9414
	MgCl_2_	210.96	30.17	0.9358
	TEMPO	235.55	34.35	0.9361
	TPPI	238.75	34.64	0.9397
	PA	242.36	34.90	0.9368

For soaked coal, in the combustion stage, the combustion activation energy of water-immersed brown coal after adding TPPI inhibitor was the highest, which was increased by 120.43 kJ/mol, indicated that the acid decomposed by phosphorus-containing inhibitor is a catalyst that can dehydrate the coal sample. Coal becomes incombustible coke agglomerates, and phosphorus-based compounds increase the energy barrier of coal oxidation reaction to a certain extent, and have the best inhibition effect on coal spontaneous combustion. After adding PA, MgCl_2_ and TEMPO, the activation energy of water-immersed brown coal increased by 97.99 kJ/mol, 91.19 kJ/mol and 79.42 kJ/mol, and indicated that PA, MgCl_2_ and TEMPO also had good inhibitory effect on water-immersed brown coal. The inhibition effect of inhibitors on water-immersed coking coal are from large to small: TPPI > PA > MgCl_2_ > TEMPO.

After adding PA, the combustion activation energy of water-immersed coking coal was the highest, which was increased by 25.77 kJ/mol, indicated that the addition of PA inhibitor had a good inhibitory effect on the high-temperature stage of water-immersed coking coal. The activation energies of TPPI and TEMPO on water-immersed coking coal increased by 22.16 and 18.96 kJ/mol, and also delayed the spontaneous combustion of water-immersed coking coal to varying degrees. The activation energy of the water-immersed coking coal with the addition of MgCl_2_ decreased in the combustion stage, and the inhibition effect was the worst. The inhibition effect of inhibitors on water-immersed coking coal are from large to small: PA > TPPI > TEMPO > MgCl_2_. At the same time, it was found that the activation energy of water-immersed brown coal was higher than that of water-immersed coking coal after adding the inhibitor, which indicated that the inhibitor had a better inhibitory effect on the spontaneous combustion of low-metamorphic water-immersed brown coal.

### Infrared spectroscopy analysis of the effect of inhibitors on water-immersed coal

#### Analysis of inhibitory effect on water-immersed brown coal

**Figure 6:**
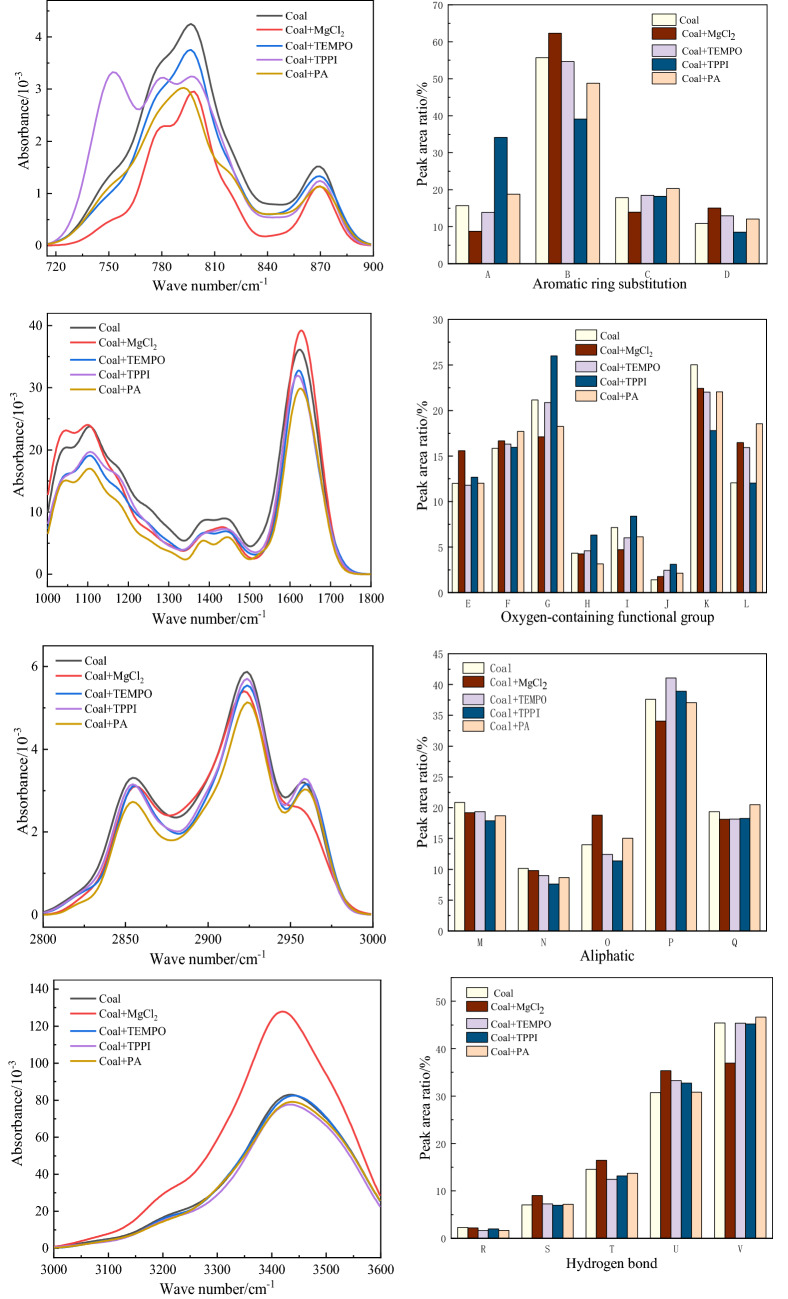
Fitting results of infrared spectral peaks and functional group peak areas of water-immersed brown coal after adding inhibitors.

Wang and Xin, etc.^23^, found in various basic reactions of coal spontaneous combustion and their interrelationships, and found that the absorption peaks in coal can be roughly divided into three categories: oxygen-containing functional groups, aliphatic hydrocarbons and aromatic hydrocarbons. Oxygen-containing functional groups include C–O stretching vibrations of phenols, alcohols, ethers, and esters, stretching vibrations of conjugated C=O, and stretching vibrations of self-associating –OH. Aliphatic hydrocarbon functional groups include methyl (–CH_3_) symmetrical variable angle vibration and methylene (–CH_2_) asymmetric variable angle vibration. methylene (–CH_2_) symmetrical stretching vibration and antisymmetric stretching vibration; methyl (–CH_3_) Symmetric stretching vibration and antisymmetric stretching vibration. Aromatic functional groups include out-of-plane vibrations of aromatic C–H bonds, which can determine the substitution state of hydrogen atoms. Aromatic C=C stretching vibrations. These three types of functional groups have a large number and high activity in coal, and have an important impact on the oxidation process of coal. Therefore, in this paper, the above three types of functional groups were used to analyzed the microstructure changes before and after adding inhibitors, and then analyzed the inhibition effect. Peak fit software was used to divide the peaks in each interval of the water-immersed coal sample to obtain the absorption peak position and peak area of each functional group. Figure 6 shows the functional group peak area and infrared spectrum fitting results of brown coal after adding inhibitors.

Combined with the analysis of experimental data, it can be seen that:

1. Oxygen-containing functional groups: E, F and G belong to ether bonds in oxygen-containing functional groups, the absorption peaks at K and L belong to the stretching vibration of C=O bond, and the absorption peak at U belongs to self-associating hydroxyl groups. After adding MgCl_2_, TEMPO and PA inhibitors, the content of ether bonds did not change much. After adding inhibitor TPPI, the content of ether bonds in water-immersed brown coal increased the most by 4.84%. The ether bond is a relatively stable structure in the coal molecule^24^, and the more ether bonds that are formed, the stronger the inhibition effect on coal spontaneous combustion is. After adding polymerization inhibitor TPPI, the C=O bond content of water-immersed brown coal decreased the most, and the decrease value was 7.27%. The remaining inhibitors reduced the C=O content in the coal by about 3%. C=O is an oxidizable active group in coal, which can be directly oxidized to form CO at low temperature, and reducing its content is beneficial to inhibit the spontaneous combustion of coal. After adding MgCl_2_ inhibitor, the OH–OH content of water-immersed brown coal increased the most, increased by 4.6%, and this is because MgCl_2_ contains a lot of crystal water, and the water is difficult to evaporate cleanly, and the coal body has larger voids after soaking in water, making it easier to absorb air medium moisture.
2. Aliphatic hydrocarbons: H, I, M, N, P, Q belong to the functional groups of aliphatic hydrocarbons. The addition of inhibitors PA, MgCl_2_, TEMPO and TPPI reduced the –CH_2_ and –CH_3_ contents in water-immersed brown coal. After adding PA, MgCl_2_ and TEMPO, the content of –CH_2_ and –CH_3_ decreased by about 1.2% and 1.49%. The content of –CH_2_ and –CH_3_ decreased most obviously after TPPI inhibition, which decreased by 2.97% and 2.6%. After the coal structure is destroyed by interfering factors, the methylene structure is released from molecular steric hindrance, which increases the content of aliphatic structures in coal^25^ and increases the side chains and bridge bonds. Therefore, as a bridge bond in coal, –CH_2_– has the most content in aliphatic hydrocarbon functional groups and has the highest activity. –CH_2_– reacts more violently after contact with oxygen molecules and accelerates the oxidation reaction of coal. –CH_3_ itself is more active. Phosphate ions in TPPI inhibitors combine with H+ in –CH_2_ and –CH_3_ in coal, reducing the content of methyl and methylene groups, thereby reducing the oxidation reaction rate of coal, which indicates that TTPI has the most significant inhibitory effect on water-immersed brown coal.
3. Aromatic hydrocarbon: B belongs to C–H vibration, J is the aromatic C=C skeleton vibration. After adding TPPI and PA inhibitors, the C–H content of aromatics decreased relatively, and the C–H content decreased the most after adding TPPI inhibitor, increased by 16.6%. This shows that the anion in TPPI replaces the H in C–H^26^, reducing the C–H bond and inhibiting the oxidation reaction of the C–H functional group, thereby increasing the stability of the structure and making it less susceptible to oxidation during low-temperature oxidation. The C–H bond content of water-immersed brown coal increased after the addition of MgCl_2_. After adding TEMPO and PA inhibitors, the C–H content of water-immersed brown coal decreased by 1.05% and 6.9%. TEMPO is a free radical scavenger and thus inhibits the regeneration of branched chains and reduces oxidation reactions. After adding TPPI inhibitor, the C=C content increased the most, increased by 1.7%. After adding MgCl_2_, TEMPO, PA inhibitor, the C=C content increased less, which were 0.35%, 1.02% and 0.72%, respectively. The TPPI, MgCl_2_, TEMPO and PA inhibitors can break the bridge bonds in the brown coal samples soaked in water, exposing more benzene ring structures in the coal, thus making the coal molecular structure more stable. Among them, the inhibitory effect of TPPI is the most obvious.

#### Analysis of inhibitory effect on water-immersed coking coal

**Figure 7:**
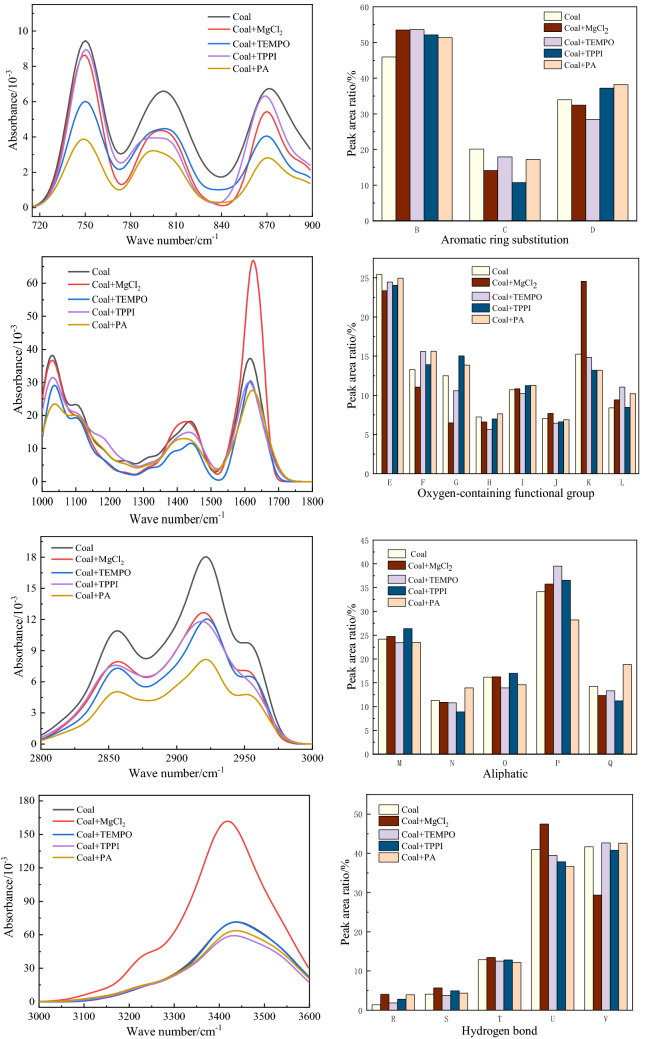
Fitting results of infrared spectral peaks and functional group peak areas of water-immersed coking coal after adding inhibitors.

Figure 7 shows the peak area of functional groups and the fitting results of infrared spectrum peaks of water-immersed coking coal after adding inhibitors.

Combined with the analysis of experimental data, it can be seen that:

1. Oxygen-containing functional groups: D, E, F belong to the ether bonds in the oxygen-containing functional groups, T belongs to the hydroxyl absorption vibration peak, and the absorption peaks at J and K belong to the C=O stretching vibration. After PA, TPPI and TEMPO treatments, the content of ether bonds in water-immersed coking coal increased by 5.21%, 4.56% and 0.74%. Under the action of the inhibitor, stable ethers were formed in the coal through a series of reactions, which slows down the further oxidation of the water-immersed coking coal. Many other functional groups were produced in MgCl_2_ treated water-immersed coking coal, and the ether bond content was reduced by the inhibitor. After adding PA and TPPI inhibitors, the C=O bond content of active groups in water-immersed coking coal decreased by 1.91% and 1.87%, which was beneficial to inhibit coal spontaneous combustion. the C=O content of water-immersed coking coal after adding TEMPO inhibitor did not change much. After adding MgCl_2_ inhibitor, the C=O content in the water-immersed coking coal increased the most. After adding MgCl_2_ inhibitor, the –OH content of water-immersed coking coal increased by 0.56%. After addition of TEMPO and TPPI inhibitors, the -OH content decreased by 0.37% and 0.05%. After adding PA inhibitor, the –OH content of water-immersed coking coal decreased the most, which was 0.72%. Hydroxyl is an important active group in oxygen-containing functional groups, and the reduction of its content will reduce the spontaneous combustion activity of coal. This indicates that metal chelating agent PA can reduce the content of oxygen-containing functional groups in coal and inhibit the oxygen-containing functional groups in coal to a greater extent. That is to say, PA inhibits the further oxidation of active oxygen-loving functional groups, delays the oxidation of coal, and reduces the risk of coking coal spontaneous combustion. Because of its strong water absorption, MgCl_2_ increases oxygen-containing functional groups, does not inhibit the formation of hydroxyl groups, and has a poor inhibitory effect on water-immersed coking coal.
2. Aliphatic hydrocarbons: G, H, L, M, O, P belong to the absorption peaks of –CH_2_ and –CH_3_. After adding inhibitor PA, the content of –CH_2_ and –CH_3_ in water-immersed coking coal decreased by 5.9% and 1.58%. After adding several other inhibitors, the content of –CH_2_ and –CH_3_ did not change significantly. The amount of –CH_2_ and –CH_3_ with higher reactivity was obviously reduced, which reduced the reaction rate of coal, increased the barrier for the reaction between coal and oxygen, and achieved the purpose of preventing spontaneous combustion of coal. This indicated that the addition of PA inhibitor inhibited the heat storage of water-immersed coking coal to some extent.
3. Aromatic hydrocarbons: Aromatic hydrocarbons include C–H absorption peaks and C=C (located at I) absorption peaks. The C–H absorption peaks of water-immersed coking coal were significantly weakened after adding the four inhibitors. After adding PA inhibitor, the C=C content in the water-immersed coking coal increased the most, from 10.71 to 11.28%, and this indicated that the bridge bonds in the water-immersed cooking coal were broken, the benzene ring was more exposed. The aromatic hydrocarbons are the core of the coal structure, the reactive functional groups in coal are converted into aromatic rings, a small amount of alicyclic and heterocyclic rings during heating. These aromatic rings are connected by oxygen-containing functional groups or chemical bonds of aliphatic hydrocarbons, which makes the skeleton structure of aromatic hydrocarbons relatively stable, and the molecular structure of coal is more stable. Therefore, the increase of C=C content is beneficial to delay the oxidative spontaneous combustion of coal.

#### Inhibitory mechanism analysis

By analyzing the differences in microstructure before and after low-temperature oxidation between the coals that are easy to spontaneously combust and those that are not easy to spontaneously combust, it is found that the content of active functional groups (–OH, –CH_3_, –CH_2_, etc.) in the coal that is easy to spontaneously combust is much more than that of the coal that is not easy to spontaneously combust. Brown coal is more prone to spontaneous combustion than coking coal. The study in this paper found that brown coal and coking coal are more prone to spontaneous combustion after soaking in water, and that water-immersed brown coal is more prone to spontaneous combustion than water-immersed coking coal, the content of oxygen-containing functional groups such as –CH_3_, –CH_2_, –OH in water-immersed brown coal is much higher than that in water-immersed coking coal. TPPI generates phosphoric acid after heating, which has a high-water absorption rate and these two substances form a thin film on the surface of the coal body, which prevents the coal body from combining with oxygen. At the same time, TPPI can capture H· and HO· for multiple times, and hydroxyl radicals (·OH) can be converted into various aliphatic hydrocarbons and oxygen-containing functional groups in coal through multiple consecutive reactions. TTPI is a hydrogen peroxide complex decomposer and chain terminator. The inhibitor inhibits the oxidation of –CH_3_ and –CH_2_ groups and reduces heat accumulation. Therefore, it is considered that the inhibitory effect of TPPI on water-immersed brown coal is better than that of water-immersed coking coal.

Compared with water-immersed coking coal, water-immersed brown coal is easier to spontaneously combust, and the content of active groups in water-immersed coking coal is relatively less, therefore, in the spontaneous combustion process of water-immersed coking coal, the presence and effect of metal ions are more obvious, the content of these metal ions is more important in suppressing coking coal. After adding PA inhibitor, the metal chelator PA can capture the metal ions connected with oxygen-containing functional groups in coal, generate more stable chelates, improve the activation energy of coal combustion stage, and inhibit the formation of oxygen-containing functional groups in coal molecules. At the same time, as a metal chelator, PA will definitely reduce the catalytic effect of metal ions, thereby delaying the process of coal oxidation reaction. Therefore, it is considered that the inhibitory effect of PA on water-immersed coking coal is better than that of water-immersed brown coal.

## Conclusion

The activation temperature from low to high was water-immersed brown coal, raw brown coal, water-immersed coking coal, and raw coking coal. The activation energy in the combustion stage presented the same trend. The absorption peak intensity of active groups increased after the two coal samples were soaked in water. The degree of change of brown coal after soaking in water was greater than that of coking coal, and the overall surface adsorption capacity of the coal sample was enhanced. This indicated that the water-immersed coal in the same metamorphic coal sample was more prone to spontaneous combustion than the raw coal. Among the coal samples with different degrees of metamorphism, the water-immersed brown coal with a lower degree of metamorphism was the most prone to spontaneous combustion.

In the low temperature stage, the inhibitor MgCl_2_ mainly played the role of physical inhibition, which can effectively improve the characteristic temperature of water-immersed brown coal and water-immersed coking coal in the stage of water loss and weight loss. In the high temperature stage, the weight loss rate of the water-immersed brown coal treated by TPPI decreased most obviously, the maximum exothermic temperature increased significantly, and the activation energy was the highest. The weight loss rate of water-immersed coking coal treated with PA decreased the most obviously, and the maximum heat release rate decreased the most. It showed that TTPI was more suitable for water-immersed brown coal, and PA was more suitable for water-immersed coking coal. In addition, from the viewpoint of activation energy, in terms of coal types, the inhibitory effect on low-metamorphic water-immersed brown coal was better than that of water-immersed coking coal.

The anion in TPPI decomposes H in C–H, effectively preventing the oxidation of oxygen-containing functional groups and methyl and methylene groups in aliphatic hydrocarbons, TPPI increased the content of ether bonds in water-immersed brown coal most obviously, and greatly reduced a large number of active groups. PA made the water-immersed coking coal form relatively stable ether bond structure, and the C=C content increased the most. At the same time, the bridge bond was broken, the benzene ring was more exposed, the molecular structure of coal was more stable, and it was not easy to spontaneously ignite. This showed that from the perspective of inhibitor, TPPI had the best inhibitory effect on water-immersed brown coal, while PA was more suitable for inhibiting the spontaneous combustion of water-immersed coking coal.

## Supplementary Information

Supplementary Information.

## Data Availability

All data generated or analysed during this study are included in this published article [and its Supplementary Information files]. The datasets used and/or analysed during the current study are also available from the corresponding author on reasonable request.
