# Supplementary material for: Research on chemical resistance characteristics of water-immersed coal with different metamorphic degrees
Source: Sci Rep. 2022 Aug 12;12:13781. doi: 10.1038/s41598-022-17865-x (PMC9374776; doi:10.1038/s41598-022-17865-x)
Supplement: Supplementary file 1 — Supplementary Information. [file 41598_2022_17865_MOESM1_ESM.pdf]

Research on chemical resistance characteristics of water-immersed coal with different metamorphic degrees

Xun Zhang[1], Mengfan Zhao[1,*], Jing Yang[1], Bing Lu[1], Gang Wang[2], and Fengwei Dai[3]

[1]College of Mining, Liaoning Technical University, Fuxin 123000, Liaoning, China

[2]China Coal Technology & Engineering Group Shenyang Research Institute Co. Ltd, Fushun 113122, Liaoning, China

[3]College of Safety Science and Engineering, Liaoning Technical University, Huludao 125105, Liaoning, China

[*]Mengfan Zhao@ zmf3418769918@163.com

Industrial Analysis (%)

Coal	***Moisture***	***Volatile matter***	***Ash***	***Fixed carbon***
Brown coal	8.36	30.84	20.26	40.54
Coking coal	1.48	20.75	9.67	68.1

Elemental analysis (%)

C	***H***	***O***	***N***	***S***
69.22	3.87	24.67	1.06	1.18
86.51	5.52	5.79	1.72	0.46

TG-DTG curves of Brown coal before

temperature	TG	temperature	TG	temperature	DTG	temperature	DTG
℃	%	℃	%	℃	%/min	℃	%/min
	Raw coal		Soaked coal		Raw coal		Soaked coal
33.612	100	27.704	100	33.612	-0.2166	27.704	-0.03296
34.612	99.609	28.704	99.96749	34.612	-0.4448	28.704	-0.12255
35.612	99.2371	29.704	99.85892	35.612	-0.4205	29.704	-0.23196
36.612	98.9381	30.704	99.70522	36.612	-0.3968	30.704	-0.3063
37.612	98.7077	31.704	99.49999	37.612	-0.3778	31.704	-0.35399
38.612	98.5202	32.704	99.29562	38.612	-0.3493	32.704	-0.36486
39.612	98.3733	33.704	99.13887	39.612	-0.3348	33.704	-0.35557
40.612	98.2442	34.704	98.99522	40.612	-0.3097	34.704	-0.3557
41.612	98.1346	35.704	98.87267	41.612	-0.2996	35.704	-0.34866
42.612	98.0478	36.704	98.75511	42.612	-0.2911	36.704	-0.34517
43.612	97.9609	37.704	98.6457	43.612	-0.2814	37.704	-0.34072
44.612	97.8785	38.704	98.54373	44.612	-0.2796	38.704	-0.33996
45.612	97.8074	39.704	98.45687	45.612	-0.284	39.704	-0.32566
46.612	97.7366	40.704	98.37354	46.612	-0.281	40.704	-0.31708
47.612	97.6683	41.704	98.29547	47.612	-0.2769	41.704	-0.31242
48.612	97.5992	42.704	98.2255	48.612	-0.2777	42.704	-0.30887
49.612	97.5376	43.704	98.16186	49.612	-0.2798	43.704	-0.30644
50.612	97.4808	44.704	98.09369	50.612	-0.2808	44.704	-0.30751
51.612	97.4207	45.704	98.0263	51.612	-0.2805	45.704	-0.30711
52.612	97.3627	46.704	97.96343	52.612	-0.2818	46.704	-0.31612
53.612	97.3066	47.704	97.90324	53.612	-0.2854	47.704	-0.31853
54.612	97.2471	48.704	97.83648	54.612	-0.2874	48.704	-0.31286
55.612	97.1921	49.704	97.77481	55.612	-0.2841	49.704	-0.31083
56.612	97.1355	50.704	97.71054	56.612	-0.2797	50.704	-0.31253
57.612	97.0784	51.704	97.6508	57.612	-0.2748	51.704	-0.31875
58.612	97.0265	52.704	97.60124	58.612	-0.2718	52.704	-0.31415
59.612	96.9788	53.704	97.54887	59.612	-0.2708	53.704	-0.31016
60.612	96.9345	54.704	97.49322	60.612	-0.2685	54.704	-0.30686
61.612	96.8909	55.704	97.43226	61.612	-0.2603	55.704	-0.30214
62.612	96.8462	56.704	97.3772	62.612	-0.2549	56.704	-0.30419
63.612	96.7973	57.704	97.32775	63.612	-0.2536	57.704	-0.31039
64.612	96.7483	58.704	97.27655	64.612	-0.2552	58.704	-0.30661
65.612	96.705	59.704	97.22596	65.612	-0.2565	59.704	-0.30011
66.612	96.6641	60.704	97.17483	66.612	-0.2594	60.704	-0.29019
67.612	96.6227	61.704	97.12204	67.612	-0.258	61.704	-0.287
68.612	96.5766	62.704	97.06801	68.612	-0.2524	62.704	-0.28587
69.612	96.5332	63.704	97.02569	69.612	-0.2484	63.704	-0.28247
70.612	96.4886	64.704	96.98357	70.612	-0.2486	64.704	-0.28269
71.612	96.4422	65.704	96.93992	71.612	-0.2474	65.704	-0.28275
72.612	96.4018	66.704	96.8953	72.612	-0.2458	66.704	-0.27638
73.612	96.3631	67.704	96.85023	73.612	-0.242	67.704	-0.26903
74.612	96.3228	68.704	96.80525	74.612	-0.2385	68.704	-0.27957
75.612	96.2824	69.704	96.75566	75.612	-0.2321	69.704	-0.28061
76.612	96.2443	70.704	96.70656	76.612	-0.2236	70.704	-0.27419
77.612	96.2038	71.704	96.66579	77.612	-0.2178	71.704	-0.26953
78.612	96.1663	72.704	96.62379	78.612	-0.213	72.704	-0.26865
79.612	96.1284	73.704	96.5658	79.612	-0.206	73.704	-0.27127
80.612	96.0958	74.704	96.52298	80.612	-0.2016	74.704	-0.26349
81.612	96.0654	75.704	96.49125	81.612	-0.1964	75.704	-0.24625
82.612	96.0347	76.704	96.45289	82.612	-0.1921	76.704	-0.24682
83.612	96.0043	77.704	96.40928	83.612	-0.1907	77.704	-0.24508

84.612	95.9744	78.704	96.35991	84.612	-0.1877	78.704	-0.22852
85.612	95.9424	79.704	96.32399	85.612	-0.1867	79.704	-0.2287
86.612	95.9111	80.704	96.30296	86.612	-0.1875	80.704	-0.23528
87.612	95.8785	81.704	96.25928	87.612	-0.1858	81.704	-0.23471
88.612	95.8427	82.704	96.21977	88.612	-0.185	82.704	-0.22586
89.612	95.8121	83.704	96.18894	89.612	-0.1836	83.704	-0.2114
90.612	95.7805	84.704	96.14648	90.612	-0.1821	84.704	-0.21288
91.612	95.7496	85.704	96.10301	91.612	-0.1796	85.704	-0.22683
92.612	95.7221	86.704	96.06578	92.612	-0.1737	86.704	-0.21963
93.612	95.6929	87.704	96.03557	93.612	-0.1673	87.704	-0.21311
94.612	95.6644	88.704	96.01019	94.612	-0.1637	88.704	-0.21175
95.612	95.6343	89.704	95.97201	95.612	-0.1594	89.704	-0.20358
96.612	95.6071	90.704	95.92765	96.612	-0.1547	90.704	-0.19715
97.612	95.5832	91.704	95.89509	97.612	-0.1524	91.704	-0.19369
98.612	95.5588	92.704	95.8679	98.612	-0.1469	92.704	-0.19428
99.612	95.5328	93.704	95.83772	99.612	-0.1408	93.704	-0.19576
100.612	95.5089	94.704	95.80567	100.612	-0.1325	94.704	-0.18548
101.612	95.4849	95.704	95.77282	101.612	-0.1254	95.704	-0.17341
102.612	95.4612	96.704	95.74188	102.612	-0.119	96.704	-0.16929
103.612	95.4409	97.704	95.71107	103.612	-0.1115	97.704	-0.16791
104.612	95.4226	98.704	95.68282	104.612	-0.1047	98.704	-0.16105
105.612	95.4067	99.704	95.65818	105.612	-0.1008	99.704	-0.15292
106.612	95.3921	100.704	95.63466	106.612	-0.095	100.704	-0.14488
107.612	95.3784	101.704	95.60961	107.612	-0.0887	101.704	-0.13848
108.612	95.3647	102.704	95.58373	108.612	-0.0839	102.704	-0.13466
109.612	95.3478	103.704	95.56503	109.612	-0.0842	103.704	-0.13097
110.612	95.33	104.704	95.54506	110.612	-0.0861	104.704	-0.12969
111.612	95.3161	105.704	95.52557	111.612	-0.088	105.704	-0.12867
112.612	95.3047	106.704	95.50155	112.612	-0.0896	106.704	-0.12134
113.612	95.2896	107.704	95.47799	113.612	-0.0889	107.704	-0.11782
114.612	95.2706	108.704	95.45561	114.612	-0.0849	108.704	-0.1217
115.612	95.2523	109.704	95.43212	115.612	-0.0827	109.704	-0.12286
116.612	95.2356	110.704	95.41266	116.612	-0.0849	110.704	-0.12218
117.612	95.2201	111.704	95.39841	117.612	-0.0863	111.704	-0.11515
118.612	95.2077	112.704	95.37488	118.612	-0.0806	112.704	-0.10967
119.612	95.1953	113.704	95.35113	119.612	-0.0721	113.704	-0.10161
120.612	95.1811	114.704	95.3289	120.612	-0.0678	114.704	-0.09386
121.612	95.164	115.704	95.31213	121.612	-0.0645	115.704	-0.09492
122.612	95.153	116.704	95.29848	122.612	-0.0612	116.704	-0.09488
123.612	95.1468	117.704	95.28588	123.612	-0.0582	117.704	-0.08662
124.612	95.1385	118.704	95.27705	124.612	-0.0524	118.704	-0.08074
125.612	95.1271	119.704	95.26178	125.612	-0.0439	119.704	-0.07585
126.612	95.1174	120.704	95.24324	126.612	-0.0365	120.704	-0.07625
127.612	95.1091	121.704	95.22879	127.612	-0.0332	121.704	-0.07693
128.612	95.1043	122.704	95.21597	128.612	-0.0301	122.704	-0.08055
129.612	95.101	123.704	95.20276	129.612	-0.0221	123.704	-0.08144
130.612	95.0987	124.704	95.1887	130.612	-0.0157	124.704	-0.07749
131.612	95.0965	125.704	95.17367	131.612	-0.0102	125.704	-0.07342
132.612	95.0937	126.704	95.1579	132.612	-0.0063	126.704	-0.07211
133.612	95.0952	127.704	95.14335	133.612	-0.0046	127.704	-0.06999
134.612	95.0959	128.704	95.13121	134.612	-0.005	128.704	-0.06823
135.612	95.0956	129.704	95.1198	135.612	-0.0073	129.704	-0.06341
136.612	95.0957	130.704	95.10962	136.612	-0.0091	130.704	-0.05647
137.612	95.0952	131.704	95.09903	137.612	-0.0114	131.704	-0.04951
138.612	95.0931	132.704	95.08822	138.612	-0.0145	132.704	-0.04449
139.612	95.0878	133.704	95.08003	139.612	-0.0174	133.704	-0.0373
140.612	95.0816	134.704	95.07441	140.612	-0.0207	134.704	-0.03164
141.612	95.0771	135.704	95.07091	141.612	-0.0247	135.704	-0.02394

142.612	95.0746	136.704	95.06429	142.612	-0.028	136.704	-0.01495
143.612	95.069	137.704	95.06411	143.612	-0.0292	137.704	-0.00886
144.612	95.0641	138.704	95.06291	144.612	-0.0284	138.704	-0.00801
145.612	95.0578	139.704	95.06279	145.612	-0.0279	139.704	-0.01242
146.612	95.051	140.704	95.06695	146.612	-0.0308	140.704	-0.01168
147.612	95.0441	141.704	95.06873	147.612	-0.0297	141.704	-0.01334
148.612	95.039	142.704	95.06637	148.612	-0.0279	142.704	-0.01588
149.612	95.0347	143.704	95.05525	149.612	-0.0269	143.704	-0.02207
150.612	95.0281	144.704	95.04829	150.612	-0.0247	144.704	-0.02752
151.612	95.023	145.704	95.0449	151.612	-0.0213	145.704	-0.03146
152.612	95.0214	146.704	95.03921	152.612	-0.0168	146.704	-0.03594
153.612	95.0173	147.704	95.0305	153.612	-0.0144	147.704	-0.03197
154.612	95.0138	148.704	95.02233	154.612	-0.0118	148.704	-0.02732
155.612	95.0111	149.704	95.01931	155.612	-0.0058	149.704	-0.02955
156.612	95.0128	150.704	95.01246	156.612	-0.0032	150.704	-0.03067
157.612	95.0134	151.704	95.00726	157.612	-0.0023	151.704	-0.02658
158.612	95.0111	152.704	95.00682	158.612	-0.0031	152.704	-0.01802
159.612	95.0131	153.704	95.00112	159.612	-0.0048	153.704	-0.02036
160.612	95.0167	154.704	94.99273	160.612	-0.0096	154.704	-0.01881
161.612	95.0156	155.704	94.98978	161.612	-0.0117	155.704	-0.01269
162.612	95.011	156.704	94.99536	162.612	-0.0094	156.704	-0.01139
163.612	95.0052	157.704	94.98935	163.612	-0.0093	157.704	-0.01371
164.612	94.9976	158.704	94.98381	164.612	-0.0136	158.704	-0.01236
165.612	94.9949	159.704	94.98605	165.612	-0.0142	159.704	-0.01274
166.612	94.9961	160.704	94.98984	166.612	-0.0111	160.704	-0.02035
167.612	94.9975	161.704	94.98304	167.612	-0.0051	161.704	-0.0212
168.612	94.9948	162.704	94.97588	168.612	0.0034	162.704	-0.01685
169.612	94.9932	163.704	94.97091	169.612	0.00562	163.704	-0.01677
170.612	94.9938	164.704	94.96293	170.612	0.00431	164.704	-0.02118
171.612	94.9971	165.704	94.95785	171.612	0.00165	165.704	-0.01656
172.612	95.0036	166.704	94.95668	172.612	-0.0003	166.704	-0.006
173.612	95.0047	167.704	94.95838	173.612	-0.0005	167.704	0.0005541
174.612	95.0029	168.704	94.95607	174.612	-0.0003	168.704	0.00794
175.612	95.0008	169.704	94.95651	175.612	5.4E-05	169.704	0.01098
176.612	94.9958	170.704	94.96561	176.612	-0.0025	170.704	0.01064
177.612	94.9927	171.704	94.97215	177.612	-0.0034	171.704	0.00704
178.612	94.9926	172.704	94.97684	178.612	-0.0031	172.704	0.00613
179.612	94.9961	173.704	94.97668	179.612	-0.0023	173.704	0.00695
180.612	94.9982	174.704	94.9745	180.612	0.00249	174.704	0.0028
181.612	94.9996	175.704	94.97016	181.612	0.00727	175.704	0.0007867
182.612	94.9983	176.704	94.9673	182.612	0.00931	176.704	-0.000161
183.612	94.9977	177.704	94.96649	183.612	0.00561	177.704	0.00162
184.612	94.9998	178.704	94.96801	184.612	0.00146	178.704	0.00459
185.612	95.0041	179.704	94.97179	185.612	-0.0005	179.704	0.00951
186.612	95.0073	180.704	94.97551	186.612	0.00254	180.704	0.01448
187.612	95.0054	181.704	94.97894	187.612	0.00574	181.704	0.01464
188.612	95.0006	182.704	94.98201	188.612	0.00673	182.704	0.01394
189.612	94.9986	183.704	94.98539	189.612	0.00599	183.704	0.01084
190.612	95.002	184.704	94.99059	190.612	0.00451	184.704	0.00374
191.612	95.0064	185.704	94.99042	191.612	0.00758	185.704	3.196E-05
192.612	95.0098	186.704	94.98999	192.612	0.01453	186.704	0.0002109
193.612	95.0129	187.704	94.98942	193.612	0.0189	187.704	-0.00181
194.612	95.014	188.704	94.98224	194.612	0.01965	188.704	-0.00698
195.612	95.0174	189.704	94.97795	195.612	0.01769	189.704	-0.00722
196.612	95.0239	190.704	94.9817	196.612	0.01657	190.704	-0.00624
197.612	95.0289	191.704	94.98206	197.612	0.01627	191.704	-0.00784
198.612	95.0327	192.704	94.9792	198.612	0.01585	192.704	-0.00312
199.612	95.0344	193.704	94.97859	199.612	0.01378	193.704	0.00127

200.612	95.0358	194.704	94.9801	200.612	0.00905	194.704	-0.00171
201.612	95.0385	195.704	94.97734	201.612	0.005	195.704	-0.0022
202.612	95.0395	196.704	94.97809	202.612	0.00234	196.704	-0.00384
203.612	95.0391	197.704	94.98028	203.612	0.00144	197.704	-0.00234
204.612	95.0379	198.704	94.97842	204.612	0.00122	198.704	3.677E-05
205.612	95.0364	199.704	94.97892	205.612	-0.001	199.704	0.00718
206.612	95.0362	200.704	94.97411	206.612	-0.0009	200.704	0.00985
207.612	95.0365	201.704	94.97409	207.612	0.00146	201.704	0.00952
208.612	95.0376	202.704	94.97895	208.612	0.00413	202.704	0.01442
209.612	95.0367	203.704	94.9886	209.612	0.00867	203.704	0.01652
210.612	95.0376	204.704	94.99358	210.612	0.0125	204.704	0.02346
211.612	95.0413	205.704	94.99482	211.612	0.0154	205.704	0.02628
212.612	95.0443	206.704	95.00159	212.612	0.0163	206.704	0.02174
213.612	95.0499	207.704	95.00496	213.612	0.01832	207.704	0.01289
214.612	95.0558	208.704	95.01203	214.612	0.01854	208.704	0.01078
215.612	95.061	209.704	95.01635	215.612	0.01894	209.704	0.0134
216.612	95.0637	210.704	95.01363	216.612	0.02224	210.704	0.00912
217.612	95.0663	211.704	95.00859	217.612	0.02246	211.704	0.0068
218.612	95.067	212.704	95.01025	218.612	0.0214	212.704	0.00206
219.612	95.0709	213.704	95.01575	219.612	0.02256	213.704	0.0006434
220.612	95.0793	214.704	95.01602	220.612	0.02525	214.704	0.00397
221.612	95.0853	215.704	95.01546	221.612	0.02554	215.704	0.00869
222.612	95.0893	216.704	95.01442	222.612	0.02757	216.704	0.00571
223.612	95.0964	217.704	95.01703	223.612	0.02579	217.704	0.00212
224.612	95.1038	218.704	95.02015	224.612	0.01848	218.704	0.000936
225.612	95.107	219.704	95.02303	225.612	0.01274	219.704	0.00256
226.612	95.1112	220.704	95.01936	226.612	0.00933	220.704	0.00416
227.612	95.1123	221.704	95.01856	227.612	0.00221	221.704	0.00309
228.612	95.1087	222.704	95.01759	228.612	-0.0056	222.704	0.00187
229.612	95.1056	223.704	95.01957	229.612	-0.0099	223.704	-0.0014
230.612	95.1044	224.704	95.02096	230.612	-0.0149	224.704	-0.00393
231.612	95.0997	225.704	95.02179	231.612	-0.0184	225.704	-0.00106
232.612	95.0946	226.704	95.02284	232.612	-0.0184	226.704	-0.00195
233.612	95.0911	227.704	95.02129	233.612	-0.0194	227.704	-0.00656
234.612	95.0872	228.704	95.01331	234.612	-0.0225	228.704	-0.00981
235.612	95.0829	229.704	95.0168	235.612	-0.023	229.704	-0.01358
236.612	95.0795	230.704	95.01485	236.612	-0.0247	230.704	-0.01909
237.612	95.0749	231.704	95.00909	237.612	-0.0289	231.704	-0.02399
238.612	95.0685	232.704	95.0053	238.612	-0.0335	232.704	-0.02029
239.612	95.0634	233.704	95.00046	239.612	-0.0391	233.704	-0.03083
240.612	95.0557	234.704	94.9926	240.612	-0.0451	234.704	-0.03817
241.612	95.0453	235.704	94.98338	241.612	-0.0503	235.704	-0.04167
242.612	95.0342	236.704	94.98143	242.612	-0.0576	236.704	-0.0455
243.612	95.0212	237.704	94.96801	243.612	-0.0672	237.704	-0.05167
244.612	95.0077	238.704	94.95446	244.612	-0.073	238.704	-0.05553
245.612	94.9954	239.704	94.94307	245.612	-0.0737	239.704	-0.0575
246.612	94.9775	240.704	94.93292	246.612	-0.0742	240.704	-0.06141
247.612	94.9563	241.704	94.91849	247.612	-0.072	241.704	-0.0608
248.612	94.939	242.704	94.90408	248.612	-0.0698	242.704	-0.05956
249.612	94.9276	243.704	94.89162	249.612	-0.0702	243.704	-0.05992
250.612	94.9161	244.704	94.88288	250.612	-0.0657	244.704	-0.06511
251.612	94.9064	245.704	94.8716	251.612	-0.0601	245.704	-0.06866
252.612	94.8965	246.704	94.85956	252.612	-0.0597	246.704	-0.07022
253.612	94.8833	247.704	94.84788	253.612	-0.0663	247.704	-0.07472
254.612	94.873	248.704	94.8296	254.612	-0.074	248.704	-0.08471
255.612	94.8613	249.704	94.80939	255.612	-0.0832	249.704	-0.09243
256.612	94.8445	250.704	94.79245	256.612	-0.0918	250.704	-0.10267
257.612	94.8222	251.704	94.7728	257.612	-0.0996	251.704	-0.11668

258.612	94.7982	252.704	94.74805	258.612	-0.1094	252.704	-0.12544
259.612	94.7736	253.704	94.72425	259.612	-0.1168	253.704	-0.12968
260.612	94.7496	254.704	94.69593	260.612	-0.1218	254.704	-0.13748
261.612	94.7243	255.704	94.66216	261.612	-0.1266	255.704	-0.14867
262.612	94.6978	256.704	94.62967	262.612	-0.1304	256.704	-0.15821
263.612	94.6741	257.704	94.6025	263.612	-0.1334	257.704	-0.16548
264.612	94.6502	258.704	94.57376	264.612	-0.1384	258.704	-0.16583
265.612	94.6203	259.704	94.53669	265.612	-0.1448	259.704	-0.16231
266.612	94.5898	260.704	94.49625	266.612	-0.1506	260.704	-0.16652
267.612	94.5604	261.704	94.4602	267.612	-0.1604	261.704	-0.17674
268.612	94.5289	262.704	94.4315	268.612	-0.1688	262.704	-0.18584
269.612	94.4933	263.704	94.40413	269.612	-0.1728	263.704	-0.18878
270.612	94.4574	264.704	94.36535	270.612	-0.1771	264.704	-0.18844
271.612	94.4176	265.704	94.32133	271.612	-0.1841	265.704	-0.19224
272.612	94.3797	266.704	94.27735	272.612	-0.192	266.704	-0.20732
273.612	94.3437	267.704	94.23631	273.612	-0.197	267.704	-0.22545
274.612	94.3071	268.704	94.19634	274.612	-0.2023	268.704	-0.23486
275.612	94.2663	269.704	94.15343	275.612	-0.2091	269.704	-0.24121
276.612	94.2223	270.704	94.09964	276.612	-0.2175	270.704	-0.24986
277.612	94.1784	271.704	94.04376	277.612	-0.2277	271.704	-0.25935
278.612	94.1337	272.704	93.98956	278.612	-0.2373	272.704	-0.27278
279.612	94.0838	273.704	93.936	279.612	-0.2456	273.704	-0.29117
280.612	94.0319	274.704	93.87837	280.612	-0.2523	274.704	-0.30361
281.612	93.9795	275.704	93.82102	281.612	-0.2613	275.704	-0.3124
282.612	93.9271	276.704	93.75891	282.612	-0.2716	276.704	-0.32407
283.612	93.8723	277.704	93.68833	283.612	-0.2762	277.704	-0.33801
284.612	93.8181	278.704	93.61437	284.612	-0.2856	278.704	-0.35034
285.612	93.7601	279.704	93.54382	285.612	-0.2934	279.704	-0.36336
286.612	93.6983	280.704	93.47202	286.612	-0.3039	280.704	-0.38011
287.612	93.6418	281.704	93.39437	287.612	-0.3132	281.704	-0.39115
288.612	93.5736	282.704	93.31799	288.612	-0.3213	282.704	-0.39923
289.612	93.5098	283.704	93.2384	289.612	-0.3293	283.704	-0.40955
290.612	93.4406	284.704	93.14897	290.612	-0.3379	284.704	-0.42096
291.612	93.3711	285.704	93.0611	291.612	-0.3541	285.704	-0.42816
292.612	93.3028	286.704	92.97549	292.612	-0.3632	286.704	-0.4404
293.612	93.2323	287.704	92.88737	293.612	-0.3719	287.704	-0.45642
294.612	93.1567	288.704	92.79635	294.612	-0.3768	288.704	-0.45962
295.612	93.0739	289.704	92.70752	295.612	-0.3832	289.704	-0.46715
296.612	92.9913	290.704	92.61153	296.612	-0.3946	290.704	-0.48546
297.612	92.9129	291.704	92.50642	297.612	-0.4077	291.704	-0.49717
298.612	92.8347	292.704	92.41244	298.612	-0.4204	292.704	-0.50593
299.612	92.7557	293.704	92.31282	299.612	-0.4253	293.704	-0.52438
300.612	92.6702	294.704	92.19655	300.612	-0.4299	294.704	-0.53963
301.612	92.5774	295.704	92.08853	301.612	-0.4408	295.704	-0.5481
302.612	92.4831	296.704	91.98441	302.612	-0.4566	296.704	-0.56745
303.612	92.391	297.704	91.86556	303.612	-0.4755	297.704	-0.58107
304.612	92.3009	298.704	91.74463	304.612	-0.4917	298.704	-0.58061
305.612	92.2037	299.704	91.62533	305.612	-0.5008	299.704	-0.59629
306.612	92.1006	300.704	91.5004	306.612	-0.5071	300.704	-0.62043
307.612	91.9894	301.704	91.37835	307.612	-0.5196	301.704	-0.63061
308.612	91.878	302.704	91.26318	308.612	-0.5342	302.704	-0.63868
309.612	91.7706	303.704	91.12946	309.612	-0.5442	303.704	-0.65128
310.612	91.665	304.704	90.98661	310.612	-0.5527	304.704	-0.66145
311.612	91.5545	305.704	90.84935	311.612	-0.5539	305.704	-0.67488
312.612	91.4418	306.704	90.7159	312.612	-0.5594	306.704	-0.69456
313.612	91.3282	307.704	90.57857	313.612	-0.5718	307.704	-0.7047
314.612	91.2121	308.704	90.43643	314.612	-0.5832	308.704	-0.70647
315.612	91.0992	309.704	90.29354	315.612	-0.5897	309.704	-0.71918

316.612	90.9769	310.704	90.14593	316.612	-0.5988	310.704	-0.73474
317.612	90.8495	311.704	89.99639	317.612	-0.6093	311.704	-0.74768
318.612	90.7256	312.704	89.84898	318.612	-0.6201	312.704	-0.76282
319.612	90.6046	313.704	89.69464	319.612	-0.6388	313.704	-0.77829
320.612	90.4771	314.704	89.53387	320.612	-0.6507	314.704	-0.79413
321.612	90.3465	315.704	89.37658	321.612	-0.6557	315.704	-0.80936
322.612	90.2127	316.704	89.2083	322.612	-0.6672	316.704	-0.8272
323.612	90.0701	317.704	89.0395	323.612	-0.6818	317.704	-0.84215
324.612	89.9316	318.704	88.86673	324.612	-0.6936	318.704	-0.85668
325.612	89.7956	319.704	88.69307	325.612	-0.7061	319.704	-0.87508
326.612	89.6539	320.704	88.52067	326.612	-0.7173	320.704	-0.89293
327.612	89.5094	321.704	88.33971	327.612	-0.721	321.704	-0.91093
328.612	89.3622	322.704	88.15658	328.612	-0.7267	322.704	-0.92702
329.612	89.2113	323.704	87.9647	329.612	-0.741	323.704	-0.94185
330.612	89.0604	324.704	87.76823	330.612	-0.7534	324.704	-0.9589
331.612	88.9119	325.704	87.57307	331.612	-0.7662	325.704	-0.9738
332.612	88.7621	326.704	87.37404	332.612	-0.7781	326.704	-0.99131
333.612	88.6055	327.704	87.175	333.612	-0.7885	327.704	-1.00548
334.612	88.4428	328.704	86.97351	334.612	-0.8057	328.704	-1.01687
335.612	88.2768	329.704	86.76964	335.612	-0.8273	329.704	-1.02835
336.612	88.1109	330.704	86.5565	336.612	-0.8447	330.704	-1.03816
337.612	87.9429	331.704	86.34443	337.612	-0.8574	331.704	-1.05162
338.612	87.7659	332.704	86.13011	338.612	-0.8691	332.704	-1.06763
339.612	87.5811	333.704	85.91564	339.612	-0.8826	333.704	-1.08515
340.612	87.4036	334.704	85.70105	340.612	-0.8964	334.704	-1.09844
341.612	87.2235	335.704	85.47948	341.612	-0.912	335.704	-1.11111
342.612	87.0444	336.704	85.25109	342.612	-0.9168	336.704	-1.12401
343.612	86.8552	337.704	85.017	343.612	-0.9196	337.704	-1.13599
344.612	86.6683	338.704	84.78994	344.612	-0.9313	338.704	-1.15632
345.612	86.4749	339.704	84.55474	345.612	-0.9462	339.704	-1.1775
346.612	86.2895	340.704	84.32309	346.612	-0.9611	340.704	-1.19216
347.612	86.1012	341.704	84.08508	347.612	-0.9714	341.704	-1.20581
348.612	85.9045	342.704	83.8366	348.612	-0.9841	342.704	-1.22341
349.612	85.7003	343.704	83.58604	349.612	-0.9972	343.704	-1.24491
350.612	85.4988	344.704	83.33131	350.612	-1.0143	344.704	-1.26854
351.612	85.2937	345.704	83.08	351.612	-1.0354	345.704	-1.2924
352.612	85.0864	346.704	82.81943	352.612	-1.053	346.704	-1.31398
353.612	84.8732	347.704	82.55223	353.612	-1.0649	347.704	-1.33758
354.612	84.6573	348.704	82.27958	354.612	-1.0815	348.704	-1.36206
355.612	84.4396	349.704	82.00352	355.612	-1.1074	349.704	-1.38348
356.612	84.2149	350.704	81.72164	356.612	-1.1322	350.704	-1.4031
357.612	83.9942	351.704	81.43383	357.612	-1.1542	351.704	-1.42447
358.612	83.7633	352.704	81.14312	358.612	-1.1759	352.704	-1.44709
359.612	83.5157	353.704	80.85312	359.612	-1.1998	353.704	-1.4731
360.612	83.2686	354.704	80.56228	360.612	-1.2222	354.704	-1.49618
361.612	83.0179	355.704	80.26048	361.612	-1.2541	355.704	-1.51554
362.612	82.7733	356.704	79.95572	362.612	-1.2816	356.704	-1.53686
363.612	82.5148	357.704	79.64048	363.612	-1.2979	357.704	-1.56582
364.612	82.2549	358.704	79.3246	364.612	-1.3133	358.704	-1.59834
365.612	81.9857	359.704	79.00163	365.612	-1.3374	359.704	-1.62697
366.612	81.7099	360.704	78.68012	366.612	-1.3656	360.704	-1.6565
367.612	81.4405	361.704	78.34207	367.612	-1.386	361.704	-1.68827
368.612	81.1645	362.704	78.00219	368.612	-1.4108	362.704	-1.72264
369.612	80.8772	363.704	77.65721	369.612	-1.432	363.704	-1.75873
370.612	80.5848	364.704	77.30481	370.612	-1.4503	364.704	-1.80089
371.612	80.2946	365.704	76.93408	371.612	-1.4764	365.704	-1.8461
372.612	79.9973	366.704	76.56711	372.612	-1.5076	366.704	-1.88732
373.612	79.692	367.704	76.19356	373.612	-1.5358	367.704	-1.93004

374.612	79.3887	368.704	75.80549	374.612	-1.5634	368.704	-1.97317
375.612	79.0759	369.704	75.40129	375.612	-1.5988	369.704	-2.01529
376.612	78.7559	370.704	74.99584	376.612	-1.6323	370.704	-2.05639
377.612	78.4246	371.704	74.58486	377.612	-1.6699	371.704	-2.10102
378.612	78.0878	372.704	74.16261	378.612	-1.7104	372.704	-2.14729
379.612	77.7422	373.704	73.7407	379.612	-1.7487	373.704	-2.197
380.612	77.3882	374.704	73.30199	380.612	-1.7862	374.704	-2.25
381.612	77.0263	375.704	72.85527	381.612	-1.8231	375.704	-2.31179
382.612	76.6611	376.704	72.39666	382.612	-1.8617	376.704	-2.37753
383.612	76.2906	377.704	71.91241	383.612	-1.9023	377.704	-2.4523
384.612	75.9074	378.704	71.42959	384.612	-1.9464	378.704	-2.53835
385.612	75.52	379.704	70.92842	385.612	-1.9867	379.704	-2.62663
386.612	75.1256	380.704	70.40181	386.612	-2.0354	380.704	-2.71497
387.612	74.7137	381.704	69.86586	387.612	-2.0979	381.704	-2.7968
388.612	74.3002	382.704	69.29385	388.612	-2.1608	382.704	-2.89824
389.612	73.8744	383.704	68.7052	389.612	-2.2334	383.704	-3.00138
390.612	73.4323	384.704	68.1189	390.612	-2.3009	384.704	-3.09925
391.612	72.9637	385.704	67.52006	391.612	-2.3738	385.704	-3.1995
392.612	72.4881	386.704	66.87891	392.612	-2.4529	386.704	-3.29079
393.612	71.9856	387.704	66.21982	393.612	-2.5469	387.704	-3.38954
394.612	71.4925	388.704	65.54487	394.612	-2.6353	388.704	-3.51102
395.612	70.9659	389.704	64.86436	395.612	-2.7183	389.704	-3.63697
396.612	70.433	390.704	64.16337	396.612	-2.8117	390.704	-3.75357
397.612	69.8616	391.704	63.43332	397.612	-2.9052	391.704	-3.86833
398.612	69.2893	392.704	62.66246	398.612	-3.0116	392.704	-4.00474
399.612	68.7001	393.704	61.87461	399.612	-3.1263	393.704	-4.15135
400.612	68.0788	394.704	61.07397	400.612	-3.2461	394.704	-4.31693
401.612	67.4444	395.704	60.25479	401.612	-3.3622	395.704	-4.48797
402.612	66.7803	396.704	59.3944	402.612	-3.4973	396.704	-4.65543
403.612	66.0896	397.704	58.49516	403.612	-3.6441	397.704	-4.83085
404.612	65.3765	398.704	57.5568	404.612	-3.8003	398.704	-5.02033
405.612	64.6485	399.704	56.5865	405.612	-3.9667	399.704	-5.2331
406.612	63.8712	400.704	55.57602	406.612	-4.1533	400.704	-5.45692
407.612	63.0755	401.704	54.54027	407.612	-4.3478	401.704	-5.68144
408.612	62.2448	402.704	53.46219	408.612	-4.5574	402.704	-5.91533
409.612	61.3722	403.704	52.34992	409.612	-4.7848	403.704	-6.15823
410.612	60.4605	404.704	51.19102	410.612	-5.0241	404.704	-6.4101
411.612	59.5103	405.704	49.97943	411.612	-5.2748	405.704	-6.67044
412.612	58.5142	406.704	48.73957	412.612	-5.5336	406.704	-6.93053
413.612	57.4838	407.704	47.44822	413.612	-5.7927	407.704	-7.19561
414.612	56.4156	408.704	46.11222	414.612	-6.0479	408.704	-7.44246
415.612	55.2852	409.704	44.74811	415.612	-6.3012	409.704	-7.67172
416.612	54.119	410.704	43.31814	416.612	-6.5417	410.704	-7.87471
417.612	52.9165	411.704	41.85789	417.612	-6.7718	411.704	-8.04357
418.612	51.6729	412.704	40.3527	418.612	-6.9766	412.704	-8.17783
419.612	50.3739	413.704	38.82361	419.612	-7.1586	413.704	-8.27516
420.612	49.0356	414.704	37.19782	420.612	-7.3119	414.704	-8.33046
421.612	47.6757	415.704	35.57776	421.612	-7.4322	415.704	-8.32611
422.612	46.2555	416.704	33.93643	422.612	-7.5251	416.704	-8.26137
423.612	44.8192	417.704	32.24288	423.612	-7.5809	417.704	-8.13778
424.612	43.3307	418.704	30.53252	424.612	-7.6002	418.704	-7.96542
425.612	41.837	419.704	28.79092	425.612	-7.5776	419.704	-7.74414
426.612	40.3021	420.704	27.09301	426.612	-7.5157	420.704	-7.47831
427.612	38.7591	421.704	25.41175	427.612	-7.4244	421.704	-7.16639
428.612	37.2171	422.704	23.77458	428.612	-7.2917	422.704	-6.81322
429.612	35.6762	423.704	22.18187	429.612	-7.1323	423.704	-6.43696
430.612	34.1319	424.704	20.65626	430.612	-6.9448	424.704	-6.05789
431.612	32.6181	425.704	19.20519	431.612	-6.7398	425.704	-5.67667

432.612	31.1255	426.704	17.87732	432.612	-6.5137	426.704	-5.30636
433.612	29.6725	427.704	16.63543	433.612	-6.2946	427.704	-4.94389
434.612	28.273	428.704	15.47256	434.612	-6.0709	428.704	-4.60477
435.612	26.9058	429.704	14.37839	435.612	-5.8574	429.704	-4.30318
436.612	25.5936	430.704	13.4035	436.612	-5.6489	430.704	-4.03104
437.612	24.324	431.704	12.4924	437.612	-5.4432	431.704	-3.77511
438.612	23.1128	432.704	11.65313	438.612	-5.2465	432.704	-3.5314
439.612	21.9172	433.704	10.86967	439.612	-5.0538	433.704	-3.30799
440.612	20.8054	434.704	10.13682	440.612	-4.855	434.704	-3.09509
441.612	19.7423	435.704	9.47603	441.612	-4.6573	435.704	-2.90054
442.612	18.6927	436.704	8.85931	442.612	-4.4403	436.704	-2.71203
443.612	17.7101	437.704	8.28355	443.612	-4.2178	437.704	-2.53794
444.612	16.7546	438.704	7.76242	444.612	-3.9796	438.704	-2.3592
445.612	15.8881	439.704	7.27309	445.612	-3.7425	439.704	-2.1947
446.612	15.0529	440.704	6.80695	446.612	-3.4883	440.704	-2.02996
447.612	14.2853	441.704	6.38848	447.612	-3.238	441.704	-1.86889
448.612	13.5815	442.704	6.01228	448.612	-2.9761	442.704	-1.7137
449.612	12.9295	443.704	5.66275	449.612	-2.7196	443.704	-1.55936
450.612	12.3448	444.704	5.35607	450.612	-2.4767	444.704	-1.40381
451.612	11.8119	445.704	5.08329	451.612	-2.2409	445.704	-1.26043
452.612	11.3462	446.704	4.83899	452.612	-2.0156	446.704	-1.12983
453.612	10.9271	447.704	4.61845	453.612	-1.7976	447.704	-0.99919
454.612	10.5582	448.704	4.42883	454.612	-1.5921	448.704	-0.88994
455.612	10.2315	449.704	4.25845	455.612	-1.4134	449.704	-0.78623
456.612	9.94903	450.704	4.11051	456.612	-1.2515	450.704	-0.69466
457.612	9.70991	451.704	3.98398	457.612	-1.1094	451.704	-0.61271
458.612	9.50392	452.704	3.86926	458.612	-0.9862	452.704	-0.54382
459.612	9.31686	453.704	3.77212	459.612	-0.8773	453.704	-0.4845
460.612	9.14961	454.704	3.68679	460.612	-0.7836	454.704	-0.4342
461.612	9.00013	455.704	3.61085	461.612	-0.7076	455.704	-0.39418
462.612	8.86538	456.704	3.54016	462.612	-0.6436	456.704	-0.35749
463.612	8.74504	457.704	3.47331	463.612	-0.5831	457.704	-0.33275
464.612	8.63698	458.704	3.41083	464.612	-0.529	458.704	-0.31622
465.612	8.5377	459.704	3.3541	465.612	-0.4822	459.704	-0.30253
466.612	8.44764	460.704	3.30065	466.612	-0.4378	460.704	-0.28963
467.612	8.36642	461.704	3.24603	467.612	-0.4015	461.704	-0.2778
468.612	8.29384	462.704	3.19007	468.612	-0.3732	462.704	-0.26897
469.612	8.22534	463.704	3.13546	469.612	-0.3465	463.704	-0.26462
470.612	8.16528	464.704	3.08631	470.612	-0.3236	464.704	-0.26344
471.612	8.10523	465.704	3.03935	471.612	-0.3045	465.704	-0.26322
472.612	8.04679	466.704	2.99098	472.612	-0.2914	466.704	-0.25908
473.612	7.99133	467.704	2.94013	473.612	-0.2803	467.704	-0.25352
474.612	7.9391	468.704	2.8897	474.612	-0.2743	468.704	-0.25095
475.612	7.88847	469.704	2.83597	475.612	-0.2695	469.704	-0.24989
476.612	7.83685	470.704	2.78546	476.612	-0.2645	470.704	-0.24843
477.612	7.78767	471.704	2.73942	477.612	-0.2593	471.704	-0.24644
478.612	7.73611	472.704	2.69331	478.612	-0.2566	472.704	-0.2432
479.612	7.68469	473.704	2.64759	479.612	-0.257	473.704	-0.23243
480.612	7.6346	474.704	2.60183	480.612	-0.2573	474.704	-0.22352
481.612	7.58602	475.704	2.55515	481.612	-0.2583	475.704	-0.21905
482.612	7.53783	476.704	2.50855	482.612	-0.258	476.704	-0.21815
483.612	7.48702	477.704	2.47114	483.612	-0.2567	477.704	-0.21904
484.612	7.43542	478.704	2.4347	484.612	-0.2562	478.704	-0.221
485.612	7.38312	479.704	2.39588	485.612	-0.2566	479.704	-0.22139
486.612	7.33263	480.704	2.35259	486.612	-0.2565	480.704	-0.21842
487.612	7.28279	481.704	2.30596	487.612	-0.255	481.704	-0.22654
488.612	7.23234	482.704	2.25605	488.612	-0.251	482.704	-0.23936
489.612	7.18321	483.704	2.20876	489.612	-0.2454	483.704	-0.25117

490.612	7.13466	484.704	2.16646	490.612	-0.2401	484.704	-0.25708
491.612	7.08628	485.704	2.11551	491.612	-0.2338	485.704	-0.25744
492.612	7.04045	486.704	2.0587	492.612	-0.228	486.704	-0.25521
493.612	6.99705	487.704	2.00049	493.612	-0.2229	487.704	-0.25394
494.612	6.95374	488.704	1.94724	494.612	-0.2178	488.704	-0.25781
495.612	6.91402	489.704	1.89927	495.612	-0.2077	489.704	-0.25695
496.612	6.87317	490.704	1.85337	496.612	-0.1974	490.704	-0.24918
497.612	6.83171	491.704	1.80744	497.612	-0.1903	491.704	-0.23863
498.612	6.79097	492.704	1.75833	498.612	-0.1827	492.704	-0.23297
499.612	6.75778	493.704	1.70988	499.612	-0.1773	493.704	-0.23186
500.612	6.72881	494.704	1.66533	500.612	-0.1699	494.704	-0.23136
501.612	6.69646	495.704	1.6238	501.612	-0.1603	495.704	-0.23277
502.612	6.66572	496.704	1.57923	502.612	-0.1498	496.704	-0.23184
503.612	6.63417	497.704	1.53192	503.612	-0.1467	497.704	-0.23054
504.612	6.60468	498.704	1.48815	504.612	-0.1489	498.704	-0.22999
505.612	6.57864	499.704	1.43903	505.612	-0.1464	499.704	-0.22682
506.612	6.55484	500.704	1.39238	506.612	-0.1434	500.704	-0.22298
507.612	6.52629	501.704	1.3446	507.612	-0.1373	501.704	-0.22015
508.612	6.49367	502.704	1.30061	508.612	-0.1308	502.704	-0.22132
509.612	6.46532	503.704	1.26381	509.612	-0.1261	503.704	-0.21602
510.612	6.43911	504.704	1.22625	510.612	-0.1237	504.704	-0.20921
511.612	6.41706	505.704	1.18446	511.612	-0.1178	505.704	-0.20212
512.612	6.39746	506.704	1.13775	512.612	-0.107	506.704	-0.1994
513.612	6.37901	507.704	1.09744	513.612	-0.0976	507.704	-0.20274
514.612	6.35899	508.704	1.0605	514.612	-0.0899	508.704	-0.20167
515.612	6.34021	509.704	1.02526	515.612	-0.086	509.704	-0.19737
516.612	6.32483	510.704	0.98544	516.612	-0.0857	510.704	-0.18921
517.612	6.31089	511.704	0.94195	517.612	-0.0867	511.704	-0.18231
518.612	6.29696	512.704	0.90605	518.612	-0.0857	512.704	-0.17643
519.612	6.28133	513.704	0.87134	519.612	-0.0828	513.704	-0.17215
520.612	6.26234	514.704	0.83721	520.612	-0.0834	514.704	-0.16589
521.612	6.24252	515.704	0.80765	521.612	-0.0844	515.704	-0.15978
522.612	6.2235	516.704	0.78009	522.612	-0.0855	516.704	-0.15855
523.612	6.20944	517.704	0.75158	523.612	-0.085	517.704	-0.15103
524.612	6.19278	518.704	0.7224	524.612	-0.0816	518.704	-0.14352
525.612	6.17689	519.704	0.68869	525.612	-0.0768	519.704	-0.14241
526.612	6.16139	520.704	0.65375	526.612	-0.0725	520.704	-0.14546
527.612	6.14651	521.704	0.63102	527.612	-0.0714	521.704	-0.14699
528.612	6.13327	522.704	0.60955	528.612	-0.0673	522.704	-0.14395
529.612	6.12083	523.704	0.58165	529.612	-0.0636	523.704	-0.13658
530.612	6.10862	524.704	0.549	530.612	-0.0591	524.704	-0.12655
531.612	6.09602	525.704	0.51761	531.612	-0.054	525.704	-0.1281
532.612	6.08588	526.704	0.49335	532.612	-0.0507	526.704	-0.13037
533.612	6.07623	527.704	0.47202	533.612	-0.0471	527.704	-0.12668
534.612	6.06751	528.704	0.45316	534.612	-0.0437	528.704	-0.12069
535.612	6.06101	529.704	0.42708	535.612	-0.0415	529.704	-0.114
536.612	6.05297	530.704	0.40204	536.612	-0.0412	530.704	-0.11057
537.612	6.04608	531.704	0.38058	537.612	-0.0406	531.704	-0.10703
538.612	6.03974	532.704	0.35791	538.612	-0.041	532.704	-0.10622
539.612	6.03073	533.704	0.33695	539.612	-0.0421	533.704	-0.10126
540.612	6.02067	534.704	0.31717	540.612	-0.0382	534.704	-0.09739
541.612	6.01192	535.704	0.30163	541.612	-0.035	535.704	-0.09614
542.612	6.00251	536.704	0.2836	542.612	-0.0332	536.704	-0.09149
543.612	5.99383	537.704	0.26629	543.612	-0.0292	537.704	-0.08699
544.612	5.99183	538.704	0.2474	544.612	-0.0241	538.704	-0.08356
545.612	5.99024	539.704	0.2274	545.612	-0.0204	539.704	-0.0819
546.612	5.98677	540.704	0.21224	546.612	-0.0162	540.704	-0.0804
547.612	5.98422	541.704	0.19843	547.612	-0.0122	541.704	-0.07931

548.612	5.9825	542.704	0.18412	548.612	-0.0148	542.704	-0.07284
549.612	5.97962	543.704	0.17089	549.612	-0.0177	543.704	-0.0653
550.612	5.97723	544.704	0.15607	550.612	-0.0186	544.704	-0.06253
551.612	5.97483	545.704	0.14005	551.612	-0.0194	545.704	-0.06168
552.612	5.96834	546.704	0.13101	552.612	-0.0195	546.704	-0.06216
553.612	5.9622	547.704	0.12333	553.612	-0.0175	547.704	-0.06368
554.612	5.95716	548.704	0.11256	554.612	-0.0158	548.704	-0.06091
555.612	5.95323	549.704	0.10006	555.612	-0.016	549.704	-0.05731
556.612	5.95114	550.704	0.08534	556.612	-0.0142	550.704	-0.06148
557.612	5.95161	551.704	0.06956	557.612	-0.012	551.704	-0.06578
558.612	5.9519	552.704	0.05903	558.612	-0.01	552.704	-0.06665
559.612	5.94939	553.704	0.04889	559.612	-0.0098	553.704	-0.06426
560.612	5.94604	554.704	0.03305	560.612	-0.0109	554.704	-0.05654
561.612	5.9433	555.704	0.01854	561.612	-0.0151	555.704	-0.04852
562.612	5.94158	556.704	0.00622	562.612	-0.0202	556.704	-0.04581
563.612	5.93779	557.704	-0.00258	563.612	-0.0223	557.704	-0.04336
564.612	5.93398	558.704	-0.00495	564.612	-0.023	558.704	-0.03467
565.612	5.92761	559.704	-0.00753	565.612	-0.0215	559.704	-0.02459
566.612	5.91973	560.704	-0.014	566.612	-0.0205	560.704	-0.01694
567.612	5.91376	561.704	-0.02023	567.612	-0.0182	561.704	-0.01385
568.612	5.90929	562.704	-0.02193	568.612	-0.0181	562.704	-0.01727
569.612	5.90871	563.704	-0.02064	569.612	-0.0171	563.704	-0.02137
570.612	5.90863	564.704	-0.02034	570.612	-0.0147	564.704	-0.02222
571.612	5.90869	565.704	-0.02425	571.612	-0.013	565.704	-0.02141
572.612	5.90533	566.704	-0.03222	572.612	-0.0114	566.704	-0.02259
573.612	5.90052	567.704	-0.04149	573.612	-0.0144	567.704	-0.02617
574.612	5.89662	568.704	-0.04912	574.612	-0.0187	568.704	-0.02874
575.612	5.89331	569.704	-0.05441	575.612	-0.0209	569.704	-0.02662
576.612	5.89131	570.704	-0.05817	576.612	-0.0205	570.704	-0.02193
577.612	5.88589	571.704	-0.06272	577.612	-0.0184	571.704	-0.01475
578.612	5.87884	572.704	-0.06663	578.612	-0.0168	572.704	-0.00687
579.612	5.87519	573.704	-0.06697	579.612	-0.0144	573.704	-0.00285
580.612	5.87258	574.704	-0.06717	580.612	-0.0132	574.704	0.0003355
581.612	5.87115	575.704	-0.06529	581.612	-0.0108	575.704	0.00398
582.612	5.86982	576.704	-0.05991	582.612	-0.007	576.704	0.00715
583.612	5.87024	577.704	-0.05889	583.612	-0.0068	577.704	0.00732
584.612	5.87016	578.704	-0.05753	584.612	-0.0066	578.704	0.00603
585.612	5.86883	579.704	-0.05605	585.612	-0.0068	579.704	0.00139
586.612	5.86808	580.704	-0.05493	586.612	-0.0058	580.704	-0.00893
587.612	5.86443	581.704	-0.05515	587.612	-0.0059	581.704	-0.01524
588.612	5.8621	582.704	-0.05718	588.612	-0.0046	582.704	-0.02205
589.612	5.8603	583.704	-0.06262	589.612	-0.002	583.704	-0.02898
590.612	5.86051	584.704	-0.07418	590.612	-0.0004	584.704	-0.03387
591.612	5.86066	585.704	-0.08288	591.612	0.00264	585.704	-0.03616
592.612	5.86268	586.704	-0.09266	592.612	0.00398	586.704	-0.03633
593.612	5.86561	587.704	-0.10256	593.612	0.00443	587.704	-0.03289
594.612	5.86729	588.704	-0.10939	594.612	0.0025	588.704	-0.0242
595.612	5.86886	589.704	-0.11342	595.612	0.00028	589.704	-0.01721
596.612	5.86865	590.704	-0.11562	596.612	-0.0036	590.704	-0.01114
597.612	5.8677	591.704	-0.11547	597.612	-0.0085	591.704	-0.00575
598.612	5.86471	592.704	-0.11281	598.612	-0.0121	592.704	-0.00307
599.612	5.86128	593.704	-0.1105	599.612	-0.015	593.704	-0.00402
600.612	5.85695	594.704	-0.11021	600.612	-0.0164	594.704	-0.00658
601.612	5.85197	595.704	-0.11129	601.612	-0.0175	595.704	-0.00987
602.612	5.84787	596.704	-0.11391	602.612	-0.0177	596.704	-0.01457
603.612	5.8447	597.704	-0.11939	603.612	-0.018	597.704	-0.01695
604.612	5.84241	598.704	-0.12597	604.612	-0.0191	598.704	-0.01639
605.612	5.83951	599.704	-0.13124	605.612	-0.0204	599.704	-0.0154

606.612	5.83648	600.704	-0.13625	606.612	-0.022	600.704	-0.0157
607.612	5.83258	601.704	-0.13796	607.612	-0.0229	601.704	-0.01246
608.612	5.82668	602.704	-0.13671	608.612	-0.0214	602.704	-0.00961
609.612	5.81961	603.704	-0.13598	609.612	-0.0192	603.704	-0.00392
610.612	5.81289	604.704	-0.13899	610.612	-0.018	604.704	0.00172
611.612	5.80797	605.704	-0.13913	611.612	-0.0167	605.704	0.0004093
612.612	5.80789	606.704	-0.14127	612.612	-0.0143	606.704	-0.00439
613.612	5.80878	607.704	-0.13765	613.612	-0.0109	607.704	-0.00686
614.612	5.80769	608.704	-0.13317	614.612	-0.0073	608.704	-0.00329
615.612	5.806	609.704	-0.13692	615.612	-0.0052	609.704	-0.00164
616.612	5.80397	610.704	-0.14355	616.612	-0.0082	610.704	0.00216
617.612	5.80239	611.704	-0.14708	617.612	-0.0116	611.704	0.0004572
618.612	5.80147	612.704	-0.14427	618.612	-0.0131	612.704	-0.000119
619.612	5.79991	613.704	-0.14199	619.612	-0.013	613.704	0.00808
620.612	5.79492	614.704	-0.13774	620.612	-0.0113	614.704	0.0164
621.612	5.79009	615.704	-0.13724	621.612	-0.0084	615.704	0.02037
622.612	5.78676	616.704	-0.13394	622.612	-0.0066	616.704	0.01912
623.612	5.78514	617.704	-0.12378	623.612	-0.0063	617.704	0.01741
624.612	5.78574	618.704	-0.11689	624.612	-0.0047	618.704	0.01436
625.612	5.78876	619.704	-0.11416	625.612	-0.0018	619.704	0.01517
626.612	5.79082	620.704	-0.11363	626.612	0.00224	620.704	0.01196
627.612	5.78995	621.704	-0.11385	627.612	0.00538	621.704	0.0016
628.612	5.78762	622.704	-0.11476	628.612	0.00525	622.704	-0.00466
629.612	5.78728	623.704	-0.11286	629.612	0.00036	623.704	-0.0079
630.612	5.79035	624.704	-0.11454	630.612	-0.0033	624.704	-0.00914
631.612	5.79383	625.704	-0.12158	631.612	-0.0015	625.704	-0.00756
632.612	5.79429	626.704	-0.1246	632.612	0.00411	626.704	-0.0048
633.612	5.78938	627.704	-0.12687	633.612	0.00651	627.704	-0.00708
634.612	5.78542	628.704	-0.12845	634.612	0.00401	628.704	-0.00813
635.612	5.78756	629.704	-0.12621	635.612	0.00024	629.704	-0.0043
636.612	5.79423	630.704	-0.12245	636.612	-0.0006	630.704	-0.0023
637.612	5.79776	631.704	-0.12417	637.612	0.00382	631.704	-0.000108
638.612	5.7968	632.704	-0.1274	638.612	0.00784	632.704	0.00194
639.612	5.79421	633.704	-0.12823	639.612	0.00627	633.704	0.0006247
640.612	5.79333	634.704	-0.12824	640.612	0.00041	634.704	-0.000576
641.612	5.79566	635.704	-0.12701	641.612	-0.0023	635.704	0.00201
642.612	5.79816	636.704	-0.12526	642.612	-0.0005	636.704	0.00421
643.612	5.79753	637.704	-0.12543	643.612	0.00236	637.704	0.0058
644.612	5.79461	638.704	-0.12374	644.612	0.00376	638.704	0.00725
645.612	5.79392	639.704	-0.12079	645.612	0.00287	639.704	0.00826
646.612	5.79605	640.704	-0.12058	646.612	0.00105	640.704	0.01218
647.612	5.79804	641.704	-0.11897	647.612	-0.0007	641.704	0.01549
648.612	5.79941	642.704	-0.11665	648.612	-0.0011	642.704	0.01127
649.612	5.80014	643.704	-0.11408	649.612	-0.0042	643.704	0.00589
650.612	5.7999	644.704	-0.10606	650.612	-0.0091	644.704	0.00577
651.612	5.79658	645.704	-0.10022	651.612	-0.0143	645.704	0.00565
652.612	5.79309	646.704	-0.10535	652.612	-0.0199	646.704	0.00783
653.612	5.78755	647.704	-0.11135	653.612	-0.0251	647.704	0.0085
654.612	5.78151	648.704	-0.1113	654.612	-0.0299	648.704	0.00298
655.612	5.77519	649.704	-0.1102	655.612	-0.03	649.704	-0.0004
656.612	5.7676	650.704	-0.10435	656.612	-0.0291	650.704	0.00695
657.612	5.76	651.704	-0.10045	657.612	-0.0269	651.704	0.01258
658.612	5.75198	652.704	-0.1016	658.612	-0.0234	652.704	0.01131
659.612	5.74852	653.704	-0.1013	659.612	-0.0186	653.704	0.01083
660.612	5.74647	654.704	-0.09414	660.612	-0.0126	654.704	0.00698
661.612	5.74439	655.704	-0.09079	661.612	-0.0057	655.704	0.00455
662.612	5.74404	656.704	-0.09301	662.612	0.00404	656.704	0.00427
663.612	5.74533	657.704	-0.09282	663.612	0.00761	657.704	0.0005587

664.612	5.74748	658.704	-0.09334	664.612	0.00794	658.704	-0.00732
665.612	5.75078	659.704	-0.09324	665.612	0.00789	659.704	-0.00932
666.612	5.75863	660.704	-0.09441	666.612	0.00694	660.704	-0.00487
667.612	5.76092	661.704	-0.10032	667.612	0.0055	661.704	0.0004706
668.612	5.75951	662.704	-0.10629	668.612	0.00494	662.704	0.00451
669.612	5.75724	663.704	-0.10606	669.612	0.00372	663.704	0.00619
670.612	5.75535	664.704	-0.10124	670.612	-0.005	664.704	0.00712
671.612	5.7541	665.704	-0.09239	671.612	-0.0095	665.704	0.0108
672.612	5.75524	666.704	-0.08626	672.612	-0.0103	666.704	0.01471
673.612	5.75682	667.704	-0.08334	673.612	-0.0096	667.704	0.01485
674.612	5.75046	668.704	-0.08275	674.612	-0.0082	668.704	0.01107
675.612	5.7458	669.704	-0.08265	675.612	-0.0073	669.704	0.0007111
676.612	5.74311	670.704	-0.08229	676.612	-0.0087	670.704	-0.00981
677.612	5.74193	671.704	-0.08218	677.612	-0.0107	671.704	-0.01611
678.612	5.74225	672.704	-0.08335	678.612	-0.0049	672.704	-0.0178
679.612	5.7424	673.704	-0.09127	679.612	0.00157	673.704	-0.01771
680.612	5.74125	674.704	-0.10224	680.612	0.00656	674.704	-0.01845
681.612	5.7396	675.704	-0.10935	681.612	0.01066	675.704	-0.01989
682.612	5.74275	676.704	-0.11148	682.612	0.01234	676.704	-0.02097
683.612	5.74828	677.704	-0.11124	683.612	0.01473	677.704	-0.01835
684.612	5.75358	678.704	-0.11197	684.612	0.01815	678.704	-0.01221
685.612	5.75897	679.704	-0.11418	685.612	0.02175	679.704	-0.0073
686.612	5.76196	680.704	-0.11696	686.612	0.02063	680.704	-0.00688
687.612	5.76598	681.704	-0.12043	687.612	0.01772	681.704	-0.01062
688.612	5.77032	682.704	-0.12132	688.612	0.01625	682.704	-0.0128
689.612	5.77465	683.704	-0.12097	689.612	0.014	683.704	-0.01165
690.612	5.77583	684.704	-0.1223	690.612	0.0125	684.704	-0.00902
691.612	5.77654	685.704	-0.12814	691.612	0.00844	685.704	-0.00536
692.612	5.77939	686.704	-0.13253	692.612	0.00362	686.704	-0.00425
693.612	5.7813	687.704	-0.13288	693.612	-0.0017	687.704	-0.00498
694.612	5.78208	688.704	-0.13146	694.612	-0.0049	688.704	-0.00432
695.612	5.77961	689.704	-0.12896	695.612	-0.007	689.704	0.00171
696.612	5.77613	690.704	-0.12817	696.612	-0.0104	690.704	0.00681
697.612	5.7718	691.704	-0.12893	697.612	-0.0114	691.704	0.00792
698.612	5.76804	692.704	-0.12937	698.612	-0.0112	692.704	0.00716
699.612	5.76523	693.704	-0.12543	699.612	-0.0093	693.704	0.00591
700.612	5.76252	694.704	-0.12153	700.612	-0.0059	694.704	0.00772
701.612	5.76275	695.704	-0.12014	701.612	-0.0011	695.704	0.01081
702.612	5.764	696.704	-0.12	702.612	0.00476	696.704	0.01382
703.612	5.76458	697.704	-0.11973	703.612	0.00862	697.704	0.01254
704.612	5.7666	698.704	-0.11592	704.612	0.01077	698.704	0.00914
705.612	5.76992	699.704	-0.11156	705.612	0.01038	699.704	0.00745
706.612	5.77563	700.704	-0.10728	706.612	0.01062	700.704	0.00884
707.612	5.77922	701.704	-0.10533	707.612	0.01021	701.704	0.00946
708.612	5.78009	702.704	-0.1068	708.612	0.00797	702.704	0.00516
709.612	5.77938	703.704	-0.10827	709.612	0.00477	703.704	-0.000284
710.612	5.78107	704.704	-0.10582	710.612	-0.0005	704.704	-0.00633
711.612	5.78106	705.704	-0.10435	711.612	-0.0032	705.704	-0.00984
712.612	5.77946	706.704	-0.10764	712.612	-0.0032	706.704	-0.00572
713.612	5.77757	707.704	-0.11202	713.612	-0.0031	707.704	5.351E-05
714.612	5.77457	708.704	-0.11728	714.612	-0.0059	708.704	-0.000411
715.612	5.77388	709.704	-0.12133	715.612	-0.0043	709.704	-0.000879
716.612	5.77487	710.704	-0.11632	716.612	-0.0009	710.704	0.00243
717.612	5.7746	711.704	-0.10834	717.612	0.00227	711.704	0.00678
718.612	5.7714	712.704	-0.10662	718.612	0.00645	712.704	0.0116
719.612	5.77392	713.704	-0.10591	719.612	0.00924	713.704	0.01602
720.612	5.77788	714.704	-0.1037	720.612	0.01063	714.704	0.01309
721.612	5.7812	715.704	-0.10091	721.612	0.01331	715.704	0.00809

722.612	5.78496	716.704	-0.09826	722.612	0.01767	716.704	0.00761
723.612	5.78864	717.704	-0.09544	723.612	0.017	717.704	0.00679
724.612	5.79182	718.704	-0.0957	724.612	0.0138	718.704	0.00249
725.612	5.79588	719.704	-0.09568	725.612	0.01077	719.704	-0.00243
726.612	5.79994	720.704	-0.09438	726.612	0.00739	720.704	-0.00443
727.612	5.80118	721.704	-0.09484	727.612	0.00386	721.704	-0.00563
728.612	5.80005	722.704	-0.09943	728.612	0.00029	722.704	-0.00612
729.612	5.79846	723.704	-0.10487	729.612	-0.0042	723.704	-0.00928
730.612	5.79682	724.704	-0.10556	730.612	-0.009	724.704	-0.01378
731.612	5.79489	725.704	-0.10461	731.612	-0.0126	725.704	-0.01501
732.612	5.79226	726.704	-0.10532	732.612	-0.0142	726.704	-0.00976
733.612	5.78911	727.704	-0.11027	733.612	-0.0153	727.704	-0.00211
734.612	5.78541	728.704	-0.11644	734.612	-0.0161	728.704	-0.00134
735.612	5.78106	729.704	-0.11907	735.612	-0.0155	729.704	-0.00303
736.612	5.7773	730.704	-0.11533	736.612	-0.0143	730.704	-0.00382
737.612	5.77392	731.704	-0.10809	737.612	-0.0127	731.704	-0.00169
738.612	5.77101	732.704	-0.10768	738.612	-0.0104	732.704	0.00246
739.612	5.77	733.704	-0.10947	739.612	-0.0074	733.704	0.00517
740.612	5.76943	734.704	-0.11132	740.612	-0.0045	734.704	0.00456
741.612	5.76883	735.704	-0.11279	741.612	-0.001	735.704	0.00144
742.612	5.76877	736.704	-0.11232	742.612	0.00284	736.704	0.00386
743.612	5.76923	737.704	-0.11075	743.612	0.00471	737.704	0.00886
744.612	5.77019	738.704	-0.10831	744.612	0.00665	738.704	0.01397
745.612	5.77233	739.704	-0.10623	745.612	0.00926	739.704	0.01776
746.612	5.77563	740.704	-0.10167	746.612	0.01104	740.704	0.01996
747.612	5.77755	741.704	-0.09532	747.612	0.0121	741.704	0.02021
748.612	5.78004	742.704	-0.08892	748.612	0.01272	742.704	0.01719
749.612	5.78363	743.704	-0.08424	749.612	0.0133	743.704	0.01244
750.612	5.78647	744.704	-0.08034	750.612	0.01329	744.704	0.00661
751.612	5.78866	745.704	-0.07817	751.612	0.01383	745.704	0.00163
752.612	5.79057	746.704	-0.08053	752.612	0.01248	746.704	-0.00179
753.612	5.79354	747.704	-0.08596	753.612	0.00946	747.704	-0.00306
754.612	5.7968	748.704	-0.09129	754.612	0.00675	748.704	-0.00365
755.612	5.79971	749.704	-0.09303	755.612	0.00481	749.704	-0.00376
756.612	5.80014	750.704	-0.09212	756.612	0.00336	750.704	-0.000335
757.612	5.79889	751.704	-0.0895	757.612	0.00186	751.704	0.00547
758.612	5.79729	752.704	-0.08636	758.612	0.00042	752.704	0.01102
759.612	5.79635	753.704	-0.08445	759.612	-0.0002	753.704	0.01221
760.612	5.79588	754.704	-0.08108	760.612	0.00153	754.704	0.00978
761.612	5.79636	755.704	-0.07716	761.612	0.00443	755.704	0.00634
762.612	5.79727	756.704	-0.07336	762.612	0.00732	756.704	0.00236
763.612	5.79911	757.704	-0.07319	763.612	0.0095	757.704	7.25E-05
764.612	5.80249	758.704	-0.07622	764.612	0.01179	758.704	-0.00311
765.612	5.80594	759.704	-0.07929	765.612	0.01247	759.704	-0.00646
766.612	5.80908	760.704	-0.08265	766.612	0.01151	760.704	-0.00965
767.612	5.81159	761.704	-0.08438	767.612	0.00847	761.704	-0.0106
768.612	5.81481	762.704	-0.0862	768.612	0.00487	762.704	-0.00942
769.612	5.81646	763.704	-0.08773	769.612	0.00171	763.704	-0.00791
770.612	5.81588	764.704	-0.08902	770.612	-0.0007	764.704	-0.00491
771.612	5.8128	765.704	-0.09018	771.612	-0.0022	765.704	-0.00226
772.612	5.81025	766.704	-0.09124	772.612	-0.0047	766.704	0.0008551
773.612	5.80854	767.704	-0.09202	773.612	-0.0071	767.704	0.00376
774.612	5.80777	768.704	-0.09049	774.612	-0.0084	768.704	0.00543
775.612	5.80794	769.704	-0.08804	775.612	-0.0074	769.704	0.0078
776.612	5.80715	770.704	-0.08488	776.612	-0.0078	770.704	0.01318
777.612	5.80505	771.704	-0.08166	777.612	-0.0088	771.704	0.01661
778.612	5.80246	772.704	-0.08025	778.612	-0.0096	772.704	0.0163
779.612	5.8011	773.704	-0.0779	779.612	-0.0111	773.704	0.01489

780.612	5.79786	774.704	-0.07016	780.612	-0.0118	774.704	0.01181
781.612	5.79455	775.704	-0.06525	781.612	-0.0109	775.704	0.00744
782.612	5.79246	776.704	-0.06429	782.612	-0.0098	776.704	0.00659
783.612	5.79017	777.704	-0.06414	783.612	-0.009	777.704	0.00741
784.612	5.78826	778.704	-0.06578	784.612	-0.0061	778.704	0.00179
785.612	5.78764	779.704	-0.06968	785.612	-0.0034	779.704	-0.00154
786.612	5.78686	780.704	-0.06986	786.612	-0.0026	780.704	-0.00279
787.612	5.7865	781.704	-0.0661	787.612	-0.0018	781.704	-0.00155
788.612	5.78805	782.704	-0.06762	788.612	-0.0021	782.704	0.00212
789.612	5.78931	783.704	-0.06784	789.612	-0.004	783.704	0.00856
790.612	5.78843	784.704	-0.06888	790.612	-0.006	784.704	0.01178
791.612	5.78734	785.704	-0.06676	791.612	-0.0103	785.704	0.00965
792.612	5.78517	786.704	-0.06249	792.612	-0.0174	786.704	0.01044
793.612	5.78124	787.704	-0.05606	793.612	-0.0158	787.704	0.01106
794.612	5.77754	788.704	-0.05096	794.612	-0.0197	788.704	0.01407
795.612	5.77008	789.704	-0.05067	795.612	-0.0219	789.704	0.01358
796.612	5.76102	790.704	-0.05067	796.612	-0.0126	790.704	0.01101
		791.704	-0.05013	792.616	0.01102	791.704	0.00697
		792.704	-0.04621	793.616	-0.0078	792.704	0.00379
		793.704	-0.045	794.616	-0.0093	793.704	0.00381
		794.704	-0.045	795.616	-0.0033	794.704	0.00394
		795.704	-0.045	796.616	4.7E-11	795.704	-5.37E-08
		796.704	-0.045			796.704	-2.65E-17

TG-DTG curves of Coking coal before

temperature	TG	temperature	TG	temperature	DTG	temperature	DTG
℃	%	℃	%	℃	%/min	℃	%/min
	Raw coal		Soaked coal		Raw coal		Soaked coal
29.74174	99.914	31.54956	99.98974	29.74174	-0.1577	31.54956	-0.2234
30.77478	99.802	32.58259	99.91447	30.77478	-0.1536	32.58259	-0.14335
31.80781	99.726	33.4865	99.83921	31.80781	-0.1475	33.4865	-0.12487
32.84085	99.651	34.51954	99.76394	32.84085	-0.1228	34.51954	-0.12077
33.74476	99.576	35.55257	99.68868	33.74476	-0.092	35.55257	-0.12077
34.7778	99.538	36.58561	99.61341	34.7778	-0.0612	36.58561	-0.10024
35.81083	99.538	37.48952	99.57578	35.81083	-0.0448	37.48952	-0.08177
36.84387	99.501	38.52256	99.53815	36.84387	-0.0407	38.52256	-0.06329
37.74778	99.501	39.55559	99.53815	37.74778	-0.0366	39.55559	-0.05303
38.78082	99.501	40.58863	99.50051	38.78082	-0.0346	40.58863	-0.04482
39.81385	99.501	41.49254	99.50051	39.81385	-0.0325	41.49254	-0.03866
40.84689	99.463	42.52557	99.50051	40.84689	-0.0305	42.52557	-0.03455
41.7508	99.463	43.55861	99.46288	41.7508	-0.0222	43.55861	-0.0284
42.78383	99.463	44.59165	99.46288	42.78383	-0.0161	44.59165	-0.02224
43.81687	99.463	45.49556	99.46288	43.81687	-0.0099	45.49556	-0.02224
44.84991	99.463	46.52859	99.46288	44.84991	-0.0058	46.52859	-0.01813
45.75382	99.463	47.56163	99.46288	45.75382	-0.0038	47.56163	-0.02018
46.78685	99.463	48.59467	99.46288	46.78685	-0.0038	48.59467	-0.01813
47.81989	99.463	49.49857	99.46288	47.81989	-0.0038	49.49857	-0.01608
48.85293	99.463	50.53161	99.46288	48.85293	-0.0079	50.53161	-0.02018
49.75683	99.463	51.56465	99.42525	49.75683	-0.012	51.56465	-0.02018
50.78987	99.463	52.59769	99.42525	50.78987	-0.0181	52.59769	-0.01608
51.82291	99.463	53.50159	99.42525	51.82291	-0.0222	53.50159	-0.01608
52.85594	99.463	54.53463	99.42525	52.85594	-0.0243	54.53463	-0.01197
53.75985	99.425	55.56767	99.42525	53.75985	-0.0263	55.56767	-0.00787
54.79289	99.425	56.6007	99.42525	54.79289	-0.0263	56.6007	-0.00992
55.82593	99.425	57.50461	99.42525	55.82593	-0.0263	57.50461	-0.00992
56.85896	99.425	58.53765	99.42525	56.85896	-0.0263	58.53765	-0.00787
57.76287	99.425	59.57069	99.42525	57.76287	-0.0243	59.57069	-0.00582
58.79591	99.425	60.60372	99.42525	58.79591	-0.0222	60.60372	-0.00582
59.82895	99.425	61.50763	99.42525	59.82895	-0.0202	61.50763	-0.00787
60.86198	99.425	62.54067	99.42525	60.86198	-0.0202	62.54067	-0.00992
61.76589	99.425	63.5737	99.42525	61.76589	-0.0202	63.5737	-0.01197
62.79893	99.388	64.60674	99.42525	62.79893	-0.0202	64.60674	-0.01403
63.83196	99.388	65.51065	99.42525	63.83196	-0.0181	65.51065	-0.01197
64.865	99.388	66.54369	99.42525	64.865	-0.0181	66.54369	-0.00787
65.76891	99.388	67.57672	99.42525	65.76891	-0.014	67.57672	-0.00787
66.80195	99.388	68.60976	99.42525	66.80195	-0.012	68.60976	-0.00582
67.83498	99.388	69.51367	99.38762	67.83498	-0.0099	69.51367	-0.00376
68.73889	99.388	70.5467	99.38762	68.73889	-0.0079	70.5467	-0.00376
69.77193	99.388	71.57974	99.38762	69.77193	-0.0058	71.57974	-0.00582
70.80496	99.388	72.48365	99.38762	70.80496	-0.0017	72.48365	-0.00787
71.838	99.388	73.51669	99.42525	71.838	0.00239	73.51669	-0.00787
72.74191	99.388	74.54972	99.42525	72.74191	0.00239	74.54972	-0.00787
73.77495	99.388	75.58276	99.42525	73.77495	0.00239	75.58276	-0.00582
74.80798	99.388	76.48667	99.38762	74.80798	0.00239	76.48667	-0.00376
75.84102	99.388	77.5197	99.38762	75.84102	0.00034	77.5197	-0.00582
76.74493	99.388	78.55274	99.38762	76.74493	-0.0017	78.55274	-0.00787
77.77796	99.388	79.58578	99.38762	77.77796	-0.0017	79.58578	-0.00992
78.811	99.388	80.48969	99.38762	78.811	-0.0017	80.48969	-0.00992
79.84404	99.388	81.52272	99.38762	79.84404	-0.0017	81.52272	-0.00582

80.74795	99.388	82.55576	99.38762	80.74795	-0.0017	82.55576	-0.00171
81.78098	99.388	83.5888	99.38762	81.78098	-0.0017	83.5888	0.0003421
82.81402	99.388	84.49271	99.38762	82.81402	-0.0017	84.49271	-0.00171
83.84706	99.388	85.52574	99.38762	83.84706	-0.0017	85.52574	-0.00376
84.75096	99.388	86.55878	99.38762	84.75096	0.00239	86.55878	-0.00582
85.784	99.388	87.59182	99.38762	85.784	0.0065	87.59182	-0.00582
86.81704	99.388	88.49572	99.38762	86.81704	0.00855	88.49572	-0.00582
87.85008	99.388	89.52876	99.38762	87.85008	0.00855	89.52876	-0.00582
88.75398	99.388	90.5618	99.38762	88.75398	0.00445	90.5618	-0.00582
89.78702	99.388	91.59483	99.38762	89.78702	0.00239	91.59483	-0.00787
90.82006	99.388	92.49874	99.38762	90.82006	0.00034	92.49874	-0.00787
91.85309	99.388	93.53178	99.38762	91.85309	0.00034	93.53178	-0.00787
92.757	99.388	94.56482	99.38762	92.757	0.00239	94.56482	-0.00992
93.79004	99.388	95.59785	99.38762	93.79004	0.00034	95.59785	-0.00992
94.82308	99.388	96.50176	99.38762	94.82308	-0.0017	96.50176	-0.00992
95.85611	99.388	97.5348	99.38762	95.85611	-0.0017	97.5348	-0.00787
96.76002	99.388	98.56783	99.38762	96.76002	-0.0017	98.56783	-0.00582
97.79306	99.388	99.60087	99.38762	97.79306	0.00239	99.60087	-0.00171
98.82609	99.388	100.50478	99.38762	98.82609	0.00445	100.50478	-0.00171
99.85913	99.388	101.53782	99.38762	99.85913	0.00239	101.53782	-0.00376
100.76304	99.388	102.57085	99.38762	100.76304	0.00034	102.57085	-0.00171
101.79608	99.388	103.60389	99.38762	101.79608	-0.0058	103.60389	0.0003421
102.82911	99.388	104.5078	99.38762	102.82911	-0.0079	104.5078	0.00239
103.86215	99.388	105.54084	99.38762	103.86215	-0.0099	105.54084	0.00445
104.76606	99.388	106.57387	99.38762	104.76606	-0.012	106.57387	0.0003421
105.79909	99.388	107.60691	99.38762	105.79909	-0.014	107.60691	-0.00171
106.83213	99.388	108.51082	99.38762	106.83213	-0.0181	108.51082	-0.00787
107.86517	99.388	109.54385	99.38762	107.86517	-0.0181	109.54385	-0.01403
108.76908	99.388	110.57689	99.38762	108.76908	-0.0181	110.57689	-0.01403
109.80211	99.388	111.60993	99.38762	109.80211	-0.012	111.60993	-0.01608
110.83515	99.388	112.51384	99.34998	110.83515	-0.0079	112.51384	-0.01813
111.73906	99.35	113.54687	99.34998	111.73906	-0.0038	113.54687	-0.02224
112.77209	99.35	114.57991	99.34998	112.77209	-0.0017	114.57991	-0.02224
113.80513	99.388	115.48382	99.34998	113.80513	0.00034	115.48382	-0.02018
114.83817	99.388	116.51685	99.34998	114.83817	0.00034	116.51685	-0.01608
115.74208	99.388	117.54989	99.34998	115.74208	0.00034	117.54989	-0.01197
116.77511	99.388	118.58293	99.34998	116.77511	-0.0017	118.58293	-0.00787
117.80815	99.388	119.48684	99.34998	117.80815	-0.0017	119.48684	-0.00787
118.84119	99.388	120.51987	99.34998	118.84119	-0.0038	120.51987	-0.00582
119.74509	99.388	121.55291	99.34998	119.74509	-0.0058	121.55291	-0.00582
120.77813	99.35	122.58595	99.34998	120.77813	-0.0079	122.58595	-0.00376
121.81117	99.35	123.48985	99.34998	121.81117	-0.0079	123.48985	-0.00376
122.84421	99.35	124.52289	99.34998	122.84421	-0.0058	124.52289	-0.00171
123.74811	99.35	125.55593	99.34998	123.74811	-0.0038	125.55593	-0.00171
124.78115	99.35	126.58897	99.34998	124.78115	0.00034	126.58897	-0.00171
125.81419	99.35	127.49287	99.34998	125.81419	0.00034	127.49287	-0.00171
126.84722	99.35	128.52591	99.34998	126.84722	0.00034	128.52591	0.0003421
127.75113	99.35	129.55895	99.34998	127.75113	0.00239	129.55895	-0.00171
128.78417	99.35	130.59198	99.34998	128.78417	0.00239	130.59198	0.0003421
129.81721	99.35	131.49589	99.34998	129.81721	0.00239	131.49589	0.00239
130.85024	99.35	132.52893	99.34998	130.85024	0.00239	132.52893	0.0003421
131.75415	99.35	133.56197	99.34998	131.75415	-0.0017	133.56197	-0.00171
132.78719	99.388	134.595	99.34998	132.78719	-0.0038	134.595	-0.00582
133.82022	99.388	135.49891	99.34998	133.82022	-0.0058	135.49891	-0.00787
134.85326	99.35	136.53195	99.34998	134.85326	-0.0058	136.53195	-0.00582
135.75717	99.35	137.56498	99.34998	135.75717	-0.0079	137.56498	-0.00582
136.79021	99.35	138.59802	99.34998	136.79021	-0.0079	138.59802	-0.00376
137.82324	99.35	139.50193	99.31235	137.82324	-0.0079	139.50193	-0.00376

138.85628	99.35	140.53497	99.31235	138.85628	-0.0038	140.53497	-0.00376
139.76019	99.35	141.568	99.34998	139.76019	0.00034	141.568	-0.00171
140.79322	99.35	142.60104	99.34998	140.79322	0.00445	142.60104	0.0003421
141.82626	99.35	143.50495	99.34998	141.82626	0.00855	143.50495	0.00445
142.8593	99.35	144.53798	99.34998	142.8593	0.01266	144.53798	0.00445
143.76321	99.35	145.57102	99.34998	143.76321	0.01471	145.57102	0.00239
144.79624	99.388	146.60406	99.34998	144.79624	0.01676	146.60406	0.0003421
145.82928	99.388	147.50797	99.34998	145.82928	0.01676	147.50797	0.0003421
146.86232	99.388	148.541	99.34998	146.86232	0.01471	148.541	0.0003421
147.76623	99.388	149.57404	99.34998	147.76623	0.01061	149.57404	-0.00171
148.79926	99.388	150.60708	99.34998	148.79926	0.00855	150.60708	-0.00171
149.8323	99.388	151.51098	99.34998	149.8323	0.0065	151.51098	-0.00376
150.86534	99.388	152.54402	99.34998	150.86534	0.0065	152.54402	-0.00582
151.76924	99.388	153.57706	99.34998	151.76924	0.0065	153.57706	-0.00787
152.80228	99.388	154.6101	99.31235	152.80228	0.00445	154.6101	-0.00992
153.83532	99.388	155.514	99.31235	153.83532	0.0065	155.514	-0.00992
154.73923	99.388	156.54704	99.31235	154.73923	0.01061	156.54704	-0.00992
155.77226	99.388	157.58008	99.31235	155.77226	0.01471	157.58008	-0.00787
156.8053	99.388	158.48398	99.31235	156.8053	0.01676	158.48398	-0.00376
157.83834	99.388	159.51702	99.31235	157.83834	0.01676	159.51702	0.0003421
158.74224	99.388	160.55006	99.31235	158.74224	0.01266	160.55006	0.00445
159.77528	99.388	161.5831	99.31235	159.77528	0.01061	161.5831	0.01266
160.80832	99.388	162.487	99.31235	160.80832	0.01061	162.487	0.01882
161.84136	99.425	163.52004	99.31235	161.84136	0.00855	163.52004	0.02497
162.74526	99.388	164.55308	99.34998	162.74526	0.00445	164.55308	0.02497
163.7783	99.388	165.58611	99.34998	163.7783	0.00034	165.58611	0.02292
164.81134	99.388	166.49002	99.34998	164.81134	-0.0038	166.49002	0.01882
165.84437	99.425	167.52306	99.34998	165.84437	-0.0079	167.52306	0.01471
166.74828	99.388	168.5561	99.34998	166.74828	-0.012	168.5561	0.01061
167.78132	99.388	169.58913	99.34998	167.78132	-0.0161	169.58913	0.00445
168.81436	99.388	170.49304	99.34998	168.81436	-0.0181	170.49304	0.0003421
169.84739	99.388	171.52608	99.34998	169.84739	-0.0202	171.52608	-0.00376
170.7513	99.388	172.55911	99.34998	170.7513	-0.0222	172.55911	-0.00171
171.78434	99.388	173.59215	99.34998	171.78434	-0.0202	173.59215	0.0003421
172.81737	99.388	174.49606	99.34998	172.81737	-0.0202	174.49606	0.00445
173.85041	99.388	175.5291	99.34998	173.85041	-0.0161	175.5291	0.00855
174.75432	99.388	176.56213	99.34998	174.75432	-0.012	176.56213	0.01266
175.78736	99.388	177.59517	99.34998	175.78736	-0.0058	177.59517	0.01471
176.82039	99.35	178.49908	99.34998	176.82039	-0.0017	178.49908	0.01882
177.85343	99.35	179.53211	99.34998	177.85343	0.00239	179.53211	0.02087
178.75734	99.388	180.56515	99.38762	178.75734	0.0065	180.56515	0.02087
179.79037	99.388	181.59819	99.38762	179.79037	0.01061	181.59819	0.01882
180.82341	99.388	182.5021	99.38762	180.82341	0.01471	182.5021	0.01471
181.85645	99.388	183.53513	99.38762	181.85645	0.01882	183.53513	0.01266
182.76036	99.388	184.56817	99.38762	182.76036	0.02292	184.56817	0.00855
183.79339	99.388	185.60121	99.38762	183.79339	0.02292	185.60121	0.0065
184.82643	99.388	186.50511	99.38762	184.82643	0.02497	186.50511	0.00445
185.85947	99.388	187.53815	99.38762	185.85947	0.02292	187.53815	0.00239
186.76337	99.388	188.57119	99.38762	186.76337	0.02087	188.57119	0.0003421
187.79641	99.425	189.60423	99.38762	187.79641	0.01882	189.60423	0.0003421
188.82945	99.425	190.50813	99.38762	188.82945	0.01676	190.50813	0.00239
189.86249	99.425	191.54117	99.38762	189.86249	0.01471	191.54117	0.00445
190.76639	99.425	192.57421	99.38762	190.76639	0.01471	192.57421	0.00855
191.79943	99.425	193.60724	99.38762	191.79943	0.01471	193.60724	0.01061
192.83247	99.425	194.51115	99.38762	192.83247	0.01471	194.51115	0.01471
193.8655	99.425	195.54419	99.38762	193.8655	0.01676	195.54419	0.01676
194.76941	99.425	196.57723	99.38762	194.76941	0.02087	196.57723	0.01676
195.80245	99.425	197.61026	99.38762	195.80245	0.02497	197.61026	0.01266

196.83549	99.425	198.51417	99.38762	196.83549	0.02497	198.51417	0.00855
197.73939	99.425	199.54721	99.42525	197.73939	0.02292	199.54721	0.00239
198.77243	99.463	200.58024	99.42525	198.77243	0.01882	200.58024	0.0003421
199.80547	99.463	201.48415	99.38762	199.80547	0.01676	201.48415	-0.00171
200.8385	99.463	202.51719	99.38762	200.8385	0.01676	202.51719	-0.00376
201.74241	99.463	203.55023	99.38762	201.74241	0.01471	203.55023	-0.00582
202.77545	99.463	204.58326	99.38762	202.77545	0.01266	204.58326	-0.00787
203.80849	99.463	205.48717	99.38762	203.80849	0.01061	205.48717	-0.00171
204.84152	99.463	206.52021	99.38762	204.84152	0.01061	206.52021	0.00445
205.74543	99.463	207.55325	99.38762	205.74543	0.01471	207.55325	0.00855
206.77847	99.463	208.58628	99.38762	206.77847	0.02292	208.58628	0.01266
207.8115	99.463	209.49019	99.38762	207.8115	0.02703	209.49019	0.01471
208.84454	99.463	210.52323	99.42525	208.84454	0.02908	210.52323	0.01676
209.74845	99.463	211.55626	99.42525	209.74845	0.03113	211.55626	0.02087
210.78149	99.501	212.5893	99.42525	210.78149	0.03524	212.5893	0.02292
211.81452	99.501	213.49321	99.42525	211.81452	0.03729	213.49321	0.02497
212.84756	99.501	214.52625	99.42525	212.84756	0.03934	214.52625	0.02908
213.75147	99.501	215.55928	99.42525	213.75147	0.03934	215.55928	0.03113
214.7845	99.538	216.59232	99.42525	214.7845	0.03729	216.59232	0.03319
215.81754	99.538	217.49623	99.42525	215.81754	0.03729	217.49623	0.03729
216.85058	99.538	218.52926	99.46288	216.85058	0.03524	218.52926	0.03934
217.75449	99.538	219.5623	99.46288	217.75449	0.03524	219.5623	0.04345
218.78752	99.538	220.59534	99.46288	218.78752	0.03319	220.59534	0.04345
219.82056	99.576	221.49925	99.46288	219.82056	0.03319	221.49925	0.0455
220.8536	99.576	222.53228	99.50051	220.8536	0.03319	222.53228	0.0455
221.7575	99.576	223.56532	99.50051	221.7575	0.03319	223.56532	0.04345
222.79054	99.576	224.59836	99.50051	222.79054	0.03524	224.59836	0.0414
223.82358	99.576	225.50226	99.50051	223.82358	0.03729	225.50226	0.03729
224.85662	99.576	226.5353	99.53815	224.85662	0.04345	226.5353	0.03729
225.76052	99.613	227.56834	99.53815	225.76052	0.04755	227.56834	0.0414
226.79356	99.613	228.60138	99.53815	226.79356	0.05371	228.60138	0.0455
227.8266	99.613	229.50528	99.53815	227.8266	0.05576	229.50528	0.04961
228.85963	99.613	230.53832	99.53815	228.85963	0.05782	230.53832	0.05166
229.76354	99.651	231.57136	99.57578	229.76354	0.05987	231.57136	0.05371
230.79658	99.651	232.60439	99.57578	230.79658	0.06398	232.60439	0.05782
231.82962	99.689	233.5083	99.57578	231.82962	0.06398	233.5083	0.06192
232.86265	99.689	234.54134	99.61341	232.86265	0.06192	234.54134	0.06192
233.76656	99.689	235.57438	99.61341	233.76656	0.05782	235.57438	0.05987
234.7996	99.689	236.60741	99.61341	234.7996	0.05576	236.60741	0.05782
235.83263	99.726	237.51132	99.65104	235.83263	0.05987	237.51132	0.05371
236.73654	99.726	238.54436	99.65104	236.73654	0.06398	238.54436	0.05371
237.76958	99.726	239.57739	99.65104	237.76958	0.06808	239.57739	0.05371
238.80262	99.764	240.61043	99.65104	238.80262	0.06808	240.61043	0.05576
239.83565	99.764	241.51434	99.68868	239.83565	0.06808	241.51434	0.05782
240.73956	99.764	242.54738	99.68868	240.73956	0.07013	242.54738	0.06192
241.7726	99.802	243.58041	99.68868	241.7726	0.07424	243.58041	0.06603
242.80563	99.802	244.48432	99.72631	242.80563	0.07424	244.48432	0.07013
243.83867	99.839	245.51736	99.72631	243.83867	0.07424	245.51736	0.07424
244.74258	99.839	246.55039	99.76394	244.74258	0.07219	246.55039	0.07629
245.77562	99.839	247.58343	99.76394	245.77562	0.07013	247.58343	0.07834
246.80865	99.877	248.48734	99.76394	246.80865	0.07013	248.48734	0.07834
247.84169	99.877	249.52038	99.80157	247.84169	0.07219	249.52038	0.07834
248.7456	99.877	250.55341	99.80157	248.7456	0.07424	250.55341	0.07834
249.77863	99.914	251.58645	99.83921	249.77863	0.07629	251.58645	0.0804
250.81167	99.914	252.49036	99.83921	250.81167	0.0804	252.49036	0.0804
251.84471	99.952	253.52339	99.83921	251.84471	0.08655	253.52339	0.08245
252.74862	99.952	254.55643	99.87684	252.74862	0.09066	254.55643	0.0845
253.78165	99.99	255.58947	99.87684	253.78165	0.09477	255.58947	0.08655

254.81469	99.99	256.49338	99.91447	254.81469	0.09682	256.49338	0.09271
255.84773	100.03	257.52641	99.91447	255.84773	0.10092	257.52641	0.09682
256.75164	100.03	258.55945	99.9521	256.75164	0.10092	258.55945	0.10092
257.78467	100.07	259.59249	99.9521	257.78467	0.10298	259.59249	0.10503
258.81771	100.07	260.49639	99.98974	258.81771	0.10503	260.49639	0.10913
259.85075	100.1	261.52943	100.02737	259.85075	0.10503	261.52943	0.11529
260.75465	100.14	262.56247	100.02737	260.75465	0.10708	262.56247	0.11735
261.78769	100.14	263.59551	100.065	261.78769	0.10913	263.59551	0.12145
262.82073	100.18	264.49941	100.065	262.82073	0.11119	264.49941	0.1235
263.85376	100.18	265.53245	100.10263	263.85376	0.11529	265.53245	0.1235
264.75767	100.22	266.56549	100.14027	264.75767	0.12145	266.56549	0.12761
265.79071	100.25	267.59852	100.1779	265.79071	0.12556	267.59852	0.13171
266.82375	100.25	268.50243	100.1779	266.82375	0.13171	268.50243	0.13171
267.85678	100.29	269.53547	100.21553	267.85678	0.13787	269.53547	0.13171
268.76069	100.33	270.56851	100.25316	268.76069	0.14198	270.56851	0.13377
269.79373	100.33	271.60154	100.25316	269.79373	0.14608	271.60154	0.13377
270.82677	100.37	272.50545	100.2908	270.82677	0.15019	272.50545	0.13582
271.8598	100.4	273.53849	100.32843	271.8598	0.15429	273.53849	0.13787
272.76371	100.44	274.57152	100.36606	272.76371	0.15429	274.57152	0.13787
273.79675	100.48	275.60456	100.36606	273.79675	0.15224	275.60456	0.13582
274.82978	100.52	276.50847	100.40369	274.82978	0.15224	276.50847	0.13582
275.86282	100.52	277.54151	100.44133	275.86282	0.15429	277.54151	0.13992
276.76673	100.55	278.57454	100.44133	276.76673	0.15429	278.57454	0.13992
277.79977	100.59	279.60758	100.47896	277.79977	0.15635	279.60758	0.14403
278.8328	100.63	280.51149	100.51659	278.8328	0.16045	280.51149	0.14814
279.73671	100.67	281.54452	100.55423	279.73671	0.16456	281.54452	0.15224
280.76975	100.67	282.57756	100.55423	280.76975	0.17277	282.57756	0.1584
281.80278	100.7	283.48147	100.59186	281.80278	0.17893	283.48147	0.16661
282.83582	100.74	284.51451	100.62949	282.83582	0.18714	284.51451	0.17277
283.73973	100.78	285.54754	100.66712	283.73973	0.19124	285.54754	0.17687
284.77277	100.82	286.58058	100.70476	284.77277	0.19535	286.58058	0.18098
285.8058	100.89	287.48449	100.74239	285.8058	0.1974	287.48449	0.18098
286.83884	100.93	288.51752	100.78002	286.83884	0.1974	288.51752	0.18098
287.74275	100.97	289.55056	100.81765	287.74275	0.19535	289.55056	0.18098
288.77578	101.01	290.5836	100.85529	288.77578	0.19124	290.5836	0.17893
289.80882	101.04	291.48751	100.89292	289.80882	0.18714	291.48751	0.17482
290.84186	101.08	292.52054	100.93055	290.84186	0.18098	292.52054	0.17277
291.74577	101.12	293.55358	100.96818	291.74577	0.17687	293.55358	0.17277
292.7788	101.16	294.58662	101.00582	292.7788	0.17687	294.58662	0.17072
293.81184	101.16	295.49052	101.04345	293.81184	0.17687	295.49052	0.17072
294.84488	101.19	296.52356	101.08108	294.84488	0.17687	296.52356	0.17277
295.74878	101.23	297.5566	101.08108	295.74878	0.18098	297.5566	0.17277
296.78182	101.27	298.58964	101.11871	296.78182	0.18303	298.58964	0.17482
297.81486	101.31	299.49354	101.15635	297.81486	0.18508	299.49354	0.17893
298.8479	101.34	300.52658	101.19398	298.8479	0.18714	300.52658	0.18303
299.7518	101.38	301.55962	101.23161	299.7518	0.18919	301.55962	0.18508
300.78484	101.42	302.59265	101.26924	300.78484	0.18714	302.59265	0.18919
301.81788	101.46	303.49656	101.30688	301.81788	0.18508	303.49656	0.18919
302.85091	101.5	304.5296	101.34451	302.85091	0.18508	304.5296	0.18919
303.75482	101.53	305.56264	101.38214	303.75482	0.18508	305.56264	0.18919
304.78786	101.57	306.59567	101.41977	304.78786	0.18508	306.59567	0.18919
305.8209	101.61	307.49958	101.45741	305.8209	0.18714	307.49958	0.18714
306.85393	101.65	308.53262	101.49504	306.85393	0.18714	308.53262	0.18303
307.75784	101.68	309.56565	101.53267	307.75784	0.18714	309.56565	0.17893
308.79088	101.72	310.59869	101.5703	308.79088	0.18919	310.59869	0.17482
309.82391	101.76	311.5026	101.60794	309.82391	0.18919	311.5026	0.17072
310.85695	101.8	312.53564	101.64557	310.85695	0.18508	312.53564	0.16661
311.76086	101.83	313.56867	101.6832	311.76086	0.18303	313.56867	0.1625

312.7939	101.87	314.60171	101.72083	312.7939	0.17687	314.60171	0.1584
313.82693	101.91	315.50562	101.75847	313.82693	0.17277	315.50562	0.15635
314.85997	101.95	316.53866	101.75847	314.85997	0.17072	316.53866	0.15429
315.76388	101.98	317.57169	101.7961	315.76388	0.16661	317.57169	0.15429
316.79691	102.02	318.60473	101.83373	316.79691	0.16045	318.60473	0.15429
317.82995	102.06	319.50864	101.87137	317.82995	0.15635	319.50864	0.15429
318.86299	102.06	320.54167	101.909	318.86299	0.15224	320.54167	0.15019
319.7669	102.1	321.57471	101.94663	319.7669	0.14608	321.57471	0.14814
320.79993	102.13	322.60775	101.94663	320.79993	0.13992	322.60775	0.14403
321.83297	102.17	323.51166	101.98426	321.83297	0.13171	323.51166	0.13582
322.73688	102.21	324.54469	102.0219	322.73688	0.12145	324.54469	0.12556
323.76991	102.21	325.57773	102.05953	323.76991	0.11119	325.57773	0.11529
324.80295	102.25	326.48164	102.05953	324.80295	0.10092	326.48164	0.10503
325.83599	102.25	327.51467	102.09716	325.83599	0.09066	327.51467	0.09477
326.7399	102.29	328.54771	102.09716	326.7399	0.0804	328.54771	0.0845
327.77293	102.29	329.58075	102.09716	327.77293	0.07424	329.58075	0.07219
328.80597	102.29	330.48466	102.13479	328.80597	0.06603	330.48466	0.06192
329.83901	102.32	331.51769	102.13479	329.83901	0.05782	331.51769	0.05371
330.74291	102.32	332.55073	102.13479	330.74291	0.05166	332.55073	0.04755
331.77595	102.32	333.58377	102.17243	331.77595	0.0455	333.58377	0.03729
332.80899	102.36	334.48767	102.17243	332.80899	0.04345	334.48767	0.02497
333.84203	102.36	335.52071	102.17243	333.84203	0.03934	335.52071	0.01471
334.74593	102.36	336.55375	102.17243	334.74593	0.03319	336.55375	0.0065
335.77897	102.36	337.58679	102.17243	335.77897	0.02497	337.58679	-0.00376
336.81201	102.36	338.49069	102.17243	336.81201	0.01882	338.49069	-0.01197
337.84504	102.36	339.52373	102.17243	337.84504	0.01266	339.52373	-0.02224
338.74895	102.36	340.55677	102.17243	338.74895	0.00445	340.55677	-0.0325
339.78199	102.36	341.5898	102.17243	339.78199	-0.0079	341.5898	-0.03866
340.81503	102.36	342.49371	102.13479	340.81503	-0.0222	342.49371	-0.04687
341.84806	102.36	343.52675	102.13479	341.84806	-0.0387	343.52675	-0.05508
342.75197	102.36	344.55979	102.13479	342.75197	-0.053	344.55979	-0.06534
343.78501	102.36	345.59282	102.09716	343.78501	-0.0653	345.59282	-0.07766
344.81804	102.32	346.49673	102.09716	344.81804	-0.0777	346.49673	-0.08792
345.85108	102.32	347.52977	102.05953	345.85108	-0.092	347.52977	-0.10024
346.75499	102.29	348.5628	102.05953	346.75499	-0.1064	348.5628	-0.11461
347.78803	102.29	349.59584	102.0219	347.78803	-0.1167	349.59584	-0.12898
348.82106	102.25	350.49975	102.0219	348.82106	-0.1249	350.49975	-0.14335
349.8541	102.21	351.53279	101.98426	349.8541	-0.1351	351.53279	-0.15566
350.75801	102.17	352.56582	101.94663	350.75801	-0.1475	352.56582	-0.16798
351.79104	102.17	353.59886	101.909	351.79104	-0.1639	353.59886	-0.18029
352.82408	102.13	354.50277	101.87137	352.82408	-0.1782	354.50277	-0.1885
353.85712	102.1	355.5358	101.83373	353.85712	-0.1947	355.5358	-0.19877
354.76103	102.06	356.56884	101.7961	354.76103	-0.209	356.56884	-0.20698
355.79406	102.02	357.60188	101.75847	355.79406	-0.2275	357.60188	-0.21519
356.8271	101.95	358.50579	101.6832	356.8271	-0.246	358.50579	-0.2234
357.86014	101.91	359.53882	101.64557	357.86014	-0.2624	359.53882	-0.23161
358.76404	101.83	360.57186	101.60794	358.76404	-0.2768	360.57186	-0.24187
359.79708	101.8	361.6049	101.5703	359.79708	-0.2891	361.6049	-0.25419
360.83012	101.72	362.5088	101.49504	360.83012	-0.2994	362.5088	-0.26445
361.86316	101.68	363.54184	101.45741	361.86316	-0.3076	363.54184	-0.27472
362.76706	101.61	364.57488	101.38214	362.76706	-0.3117	364.57488	-0.28703
363.8001	101.53	365.60792	101.34451	363.8001	-0.3178	365.60792	-0.29935
364.83314	101.5	366.51182	101.26924	364.83314	-0.324	366.51182	-0.31372
365.73705	101.42	367.54486	101.19398	365.73705	-0.3322	367.54486	-0.32603
366.77008	101.34	368.5779	101.15635	366.77008	-0.3404	368.5779	-0.3363
367.80312	101.27	369.4818	101.08108	367.80312	-0.3486	369.4818	-0.34861
368.83616	101.19	370.51484	101.00582	368.83616	-0.3589	370.51484	-0.36093
369.74006	101.12	371.54788	100.93055	369.74006	-0.3712	371.54788	-0.37325

370.7731	101.04	372.58092	100.85529	370.7731	-0.3876	372.58092	-0.38146
371.80614	100.97	373.48482	100.78002	371.80614	-0.3999	373.48482	-0.38967
372.83917	100.89	374.51786	100.70476	372.83917	-0.4102	374.51786	-0.39583
373.74308	100.82	375.5509	100.59186	373.74308	-0.4184	375.5509	-0.40404
374.77612	100.74	376.58393	100.51659	374.77612	-0.4266	376.58393	-0.41635
375.80916	100.63	377.48784	100.44133	375.80916	-0.4328	377.48784	-0.42456
376.84219	100.55	378.52088	100.36606	376.84219	-0.4369	378.52088	-0.42867
377.7461	100.48	379.55392	100.2908	377.7461	-0.441	379.55392	-0.43483
378.77914	100.37	380.58695	100.1779	378.77914	-0.443	380.58695	-0.44509
379.81218	100.29	381.49086	100.10263	379.81218	-0.4451	381.49086	-0.45125
380.84521	100.18	382.5239	99.98974	380.84521	-0.4492	382.5239	-0.45741
381.74912	100.1	383.55693	99.91447	381.74912	-0.4533	383.55693	-0.46151
382.78216	100.03	384.58997	99.80157	382.78216	-0.4574	384.58997	-0.46151
383.81519	99.914	385.49388	99.72631	383.81519	-0.4677	385.49388	-0.46151
384.84823	99.839	386.52692	99.61341	384.84823	-0.4759	386.52692	-0.46562
385.75214	99.726	387.55995	99.53815	385.75214	-0.4841	387.55995	-0.47178
386.78518	99.651	388.59299	99.42525	386.78518	-0.4923	388.59299	-0.47383
387.81821	99.538	389.4969	99.34998	387.81821	-0.5005	389.4969	-0.47793
388.85125	99.425	390.52993	99.23709	388.85125	-0.5108	390.52993	-0.48204
389.75516	99.312	391.56297	99.16182	389.75516	-0.519	391.56297	-0.4882
390.78819	99.237	392.59601	99.04892	390.78819	-0.5272	392.59601	-0.49436
391.82123	99.124	393.49992	98.93602	391.82123	-0.5313	393.49992	-0.49846
392.85427	99.011	394.53295	98.86076	392.85427	-0.5334	394.53295	-0.49846
393.75818	98.898	395.56599	98.74786	393.75818	-0.5375	395.56599	-0.50051
394.79121	98.785	396.59903	98.63496	394.79121	-0.5395	396.59903	-0.50257
395.82425	98.673	397.50293	98.5597	395.82425	-0.5416	397.50293	-0.50257
396.85729	98.56	398.53597	98.4468	396.85729	-0.5436	398.53597	-0.50667
397.76119	98.447	399.56901	98.3339	397.76119	-0.5477	399.56901	-0.51078
398.79423	98.372	400.60205	98.25864	398.79423	-0.5518	400.60205	-0.51693
399.82727	98.259	401.50595	98.14574	399.82727	-0.5559	401.50595	-0.52515
400.86031	98.146	402.53899	98.03284	400.86031	-0.5621	402.53899	-0.53541
401.76421	98.033	403.57203	97.91995	401.76421	-0.5703	403.57203	-0.54567
402.79725	97.882	404.60506	97.80705	402.79725	-0.5806	404.60506	-0.55799
403.83029	97.769	405.50897	97.69415	403.83029	-0.5888	405.50897	-0.5703
404.86332	97.657	406.54201	97.58125	404.86332	-0.599	406.54201	-0.58057
405.76723	97.544	407.57505	97.46835	405.76723	-0.6073	407.57505	-0.58673
406.80027	97.431	408.60808	97.35546	406.80027	-0.6155	408.60808	-0.59494
407.83331	97.28	409.51199	97.20493	407.83331	-0.6278	409.51199	-0.60109
408.73721	97.167	410.54503	97.09203	408.73721	-0.638	410.54503	-0.60725
409.77025	97.054	411.57806	96.97913	409.77025	-0.6483	411.57806	-0.61341
410.80329	96.904	412.48197	96.86623	410.80329	-0.6586	412.48197	-0.61957
411.83632	96.791	413.51501	96.7157	411.83632	-0.6668	413.51501	-0.62778
412.74023	96.64	414.54805	96.60281	412.74023	-0.677	414.54805	-0.63599
413.77327	96.49	415.58108	96.48991	413.77327	-0.6853	415.58108	-0.64625
414.80631	96.377	416.48499	96.33938	414.80631	-0.6914	416.48499	-0.65857
415.83934	96.226	417.51803	96.22648	415.83934	-0.6976	417.51803	-0.67294
416.74325	96.076	418.55106	96.07595	416.74325	-0.7017	418.55106	-0.68731
417.77629	95.925	419.5841	95.92542	417.77629	-0.7058	419.5841	-0.70168
418.80932	95.813	420.48801	95.81252	418.80932	-0.7099	420.48801	-0.71605
419.84236	95.662	421.52105	95.66199	419.84236	-0.7161	421.52105	-0.73041
420.74627	95.511	422.55408	95.51146	420.74627	-0.7243	422.55408	-0.74478
421.77931	95.361	423.58712	95.36093	421.77931	-0.7345	423.58712	-0.7612
422.81234	95.21	424.49103	95.2104	422.81234	-0.7489	424.49103	-0.77557
423.84538	95.06	425.52407	95.05987	423.84538	-0.7694	425.52407	-0.78789
424.74929	94.909	426.5571	94.87171	424.74929	-0.7879	426.5571	-0.79815
425.78232	94.759	427.59014	94.72118	425.78232	-0.8105	427.59014	-0.81457
426.81536	94.608	428.49405	94.57065	426.81536	-0.8351	428.49405	-0.82689
427.8484	94.42	429.52708	94.38248	427.8484	-0.8597	429.52708	-0.84126

428.75231	94.232	430.56012	94.23195	428.75231	-0.8844	430.56012	-0.85358
429.78534	94.081	431.59316	94.04379	429.78534	-0.9111	431.59316	-0.86794
430.81838	93.893	432.49707	93.89326	430.81838	-0.9336	432.49707	-0.88642
431.85142	93.705	433.5301	93.7051	431.85142	-0.9521	433.5301	-0.909
432.75532	93.517	434.56314	93.51693	432.75532	-0.9726	434.56314	-0.93363
433.78836	93.291	435.59618	93.32877	433.78836	-0.9952	435.59618	-0.95416
434.8214	93.103	436.50008	93.14061	434.8214	-1.0137	436.50008	-0.97674
435.85444	92.915	437.53312	92.95245	435.85444	-1.0322	437.53312	-1.00547
436.75834	92.689	438.56616	92.72665	436.75834	-1.0506	438.56616	-1.03626
437.79138	92.463	439.5992	92.53849	437.79138	-1.0712	439.5992	-1.06705
438.82442	92.275	440.5031	92.31269	438.82442	-1.0917	440.5031	-1.09169
439.85745	92.049	441.53614	92.0869	439.85745	-1.1163	441.53614	-1.11427
440.76136	91.823	442.56918	91.8611	440.76136	-1.143	442.56918	-1.1389
441.7944	91.598	443.60221	91.63531	441.7944	-1.1717	443.60221	-1.16558
442.82744	91.372	444.50612	91.40951	442.82744	-1.2046	444.50612	-1.19432
443.86047	91.108	445.53916	91.14608	443.86047	-1.2374	445.53916	-1.22306
444.76438	90.883	446.5722	90.92029	444.76438	-1.2703	446.5722	-1.2518
445.79742	90.619	447.60523	90.65686	445.79742	-1.3113	447.60523	-1.28259
446.83045	90.356	448.50914	90.39343	446.83045	-1.3503	448.50914	-1.31543
447.86349	90.055	449.54218	90.13	447.86349	-1.3873	449.54218	-1.35443
448.7674	89.791	450.57521	89.86658	448.7674	-1.4242	450.57521	-1.39138
449.80044	89.49	451.60825	89.56551	449.80044	-1.455	451.60825	-1.43038
450.83347	89.227	452.51216	89.30209	450.83347	-1.4879	452.51216	-1.46733
451.73738	88.926	453.5452	89.00103	451.73738	-1.5228	453.5452	-1.50428
452.77042	88.587	454.57823	88.69997	452.77042	-1.5597	454.57823	-1.54122
453.80345	88.286	455.48214	88.39891	453.80345	-1.5905	455.48214	-1.58023
454.83649	87.985	456.51518	88.06021	454.83649	-1.6254	456.51518	-1.62128
455.7404	87.646	457.54821	87.75915	455.7404	-1.6603	457.54821	-1.66233
456.77344	87.308	458.58125	87.42046	456.77344	-1.7013	458.58125	-1.70749
457.80647	86.969	459.48516	87.08177	457.80647	-1.7465	459.48516	-1.7506
458.83951	86.63	460.5182	86.70544	458.83951	-1.7917	460.5182	-1.79576
459.74342	86.254	461.55123	86.36675	459.74342	-1.8389	461.55123	-1.84502
460.77645	85.878	462.58427	85.99042	460.77645	-1.8881	462.58427	-1.89634
461.80949	85.501	463.48818	85.6141	461.80949	-1.9436	463.48818	-1.94766
462.84253	85.125	464.52121	85.20014	462.84253	-1.999	464.52121	-2.00308
463.74644	84.711	465.55425	84.82381	463.74644	-2.0544	465.55425	-2.05645
464.77947	84.335	466.58729	84.40985	464.77947	-2.1098	466.58729	-2.11187
465.81251	83.883	467.4912	83.99589	465.81251	-2.1714	467.4912	-2.17345
466.84555	83.469	468.52423	83.5443	466.84555	-2.233	468.52423	-2.24324
467.74945	83.017	469.55727	83.09271	467.74945	-2.3007	469.55727	-2.31098
468.78249	82.566	470.59031	82.64112	468.78249	-2.3644	470.59031	-2.37872
469.81553	82.077	471.49421	82.1519	469.81553	-2.4321	471.49421	-2.45262
470.84857	81.587	472.52725	81.66268	470.84857	-2.4998	472.52725	-2.53062
471.75247	81.098	473.56029	81.13582	471.75247	-2.5737	473.56029	-2.61067
472.78551	80.571	474.59333	80.60896	472.78551	-2.6497	474.59333	-2.69073
473.81855	80.044	475.49723	80.08211	473.81855	-2.7174	475.49723	-2.76873
474.85158	79.518	476.53027	79.51762	474.85158	-2.7913	476.53027	-2.83647
475.75549	78.953	477.56331	78.95313	475.75549	-2.8652	477.56331	-2.91037
476.78853	78.389	478.59634	78.35101	476.78853	-2.935	478.59634	-2.99042
477.82157	77.787	479.50025	77.74889	477.82157	-3.013	479.50025	-3.06021
478.8546	77.184	480.53329	77.14677	478.8546	-3.0869	480.53329	-3.13616
479.75851	76.545	481.56633	76.50701	479.75851	-3.1629	481.56633	-3.2039
480.79155	75.943	482.59936	75.86726	480.79155	-3.2367	482.59936	-3.27574
481.82458	75.265	483.50327	75.22751	481.82458	-3.3106	483.50327	-3.34554
482.85762	74.625	484.53631	74.55012	482.85762	-3.3886	484.53631	-3.42559
483.76153	73.91	485.56934	73.87273	483.76153	-3.4605	485.56934	-3.50154
484.79457	73.233	486.60238	73.15771	484.79457	-3.5344	486.60238	-3.57544
485.8276	72.518	487.50629	72.4427	485.8276	-3.6021	487.50629	-3.64728

486.86064	71.803	488.53933	71.72768	486.86064	-3.674	488.53933	-3.71297
487.76455	71.05	489.57236	70.97503	487.76455	-3.7397	489.57236	-3.7766
488.79759	70.298	490.6054	70.22237	488.79759	-3.8053	490.6054	-3.84229
489.83062	69.545	491.50931	69.43209	489.83062	-3.871	491.50931	-3.90797
490.86366	68.755	492.54234	68.64181	490.86366	-3.9326	492.54234	-3.96545
491.76757	67.964	493.57538	67.85152	491.76757	-3.9942	493.57538	-4.01676
492.8006	67.174	494.60842	67.06124	492.8006	-4.0558	494.60842	-4.06398
493.83364	66.346	495.51233	66.23332	493.83364	-4.1153	495.51233	-4.11119
494.73755	65.518	496.54536	65.40541	494.73755	-4.1707	496.54536	-4.1584
495.77059	64.69	497.5784	64.57749	495.77059	-4.2241	497.5784	-4.20151
496.80362	63.825	498.48231	63.71194	496.80362	-4.2754	498.48231	-4.23845
497.83666	62.959	499.51534	62.84639	497.83666	-4.3226	499.51534	-4.2713
498.74057	62.094	500.54838	61.98084	498.74057	-4.3657	500.54838	-4.30414
499.7736	61.191	501.58142	61.11529	499.7736	-4.4068	501.58142	-4.33493
500.80664	60.325	502.48533	60.21211	500.80664	-4.4376	502.48533	-4.36367
501.83968	59.422	503.51836	59.34656	501.83968	-4.4643	503.51836	-4.38625
502.74359	58.519	504.5514	58.44338	502.74359	-4.4889	504.5514	-4.40677
503.77662	57.615	505.58444	57.57783	503.77662	-4.5053	505.58444	-4.4232
504.80966	56.675	506.48834	56.67465	504.80966	-4.5176	506.48834	-4.42935
505.8427	55.771	507.52138	55.77147	505.8427	-4.5258	507.52138	-4.43141
506.7466	54.831	508.55442	54.83065	506.7466	-4.5279	508.55442	-4.42935
507.77964	53.927	509.58746	53.92747	507.77964	-4.5258	509.58746	-4.41909
508.81268	52.987	510.49136	53.02429	508.81268	-4.5238	510.49136	-4.40472
509.84572	52.083	511.5244	52.15874	509.84572	-4.5176	511.5244	-4.38009
510.74962	51.143	512.55744	51.21793	510.74962	-4.4991	512.55744	-4.3493
511.78266	50.202	513.59047	50.31475	511.78266	-4.4807	513.59047	-4.31235
512.8157	49.299	514.49438	49.4492	512.8157	-4.456	514.49438	-4.27745
513.84873	48.358	515.52742	48.54601	513.84873	-4.4273	515.52742	-4.23435
514.75264	47.455	516.56046	47.7181	514.75264	-4.3883	516.56046	-4.18098
515.78568	46.551	517.59349	46.85255	515.78568	-4.3452	517.59349	-4.12145
516.81872	45.686	518.4974	45.987	516.81872	-4.298	518.4974	-4.05987
517.85175	44.783	519.53044	45.15908	517.85175	-4.2426	519.53044	-3.99624
518.75566	43.917	520.56347	44.33117	518.75566	-4.1912	520.56347	-3.9326
519.7887	43.052	521.59651	43.54088	519.7887	-4.1297	521.59651	-3.86076
520.82173	42.224	522.50042	42.7506	520.82173	-4.0681	522.50042	-3.78276
521.85477	41.358	523.53346	41.96031	521.85477	-4.0024	523.53346	-3.70476
522.75868	40.568	524.56649	41.2453	522.75868	-3.9388	524.56649	-3.63086
523.79172	39.74	525.59953	40.49264	523.79172	-3.869	525.59953	-3.5508
524.82475	38.95	526.50344	39.77763	524.82475	-3.7951	526.50344	-3.47691
525.85779	38.159	527.53647	39.06261	525.85779	-3.7232	527.53647	-3.3948
526.7617	37.407	528.56951	38.38522	526.7617	-3.6473	528.56951	-3.31475
527.79473	36.654	529.60255	37.70783	527.79473	-3.5754	529.60255	-3.2388
528.82777	35.939	530.50646	37.03045	528.82777	-3.4954	530.50646	-3.1649
529.86081	35.224	531.53949	36.39069	529.86081	-3.4174	531.53949	-3.0869
530.76472	34.509	532.57253	35.78857	530.76472	-3.3353	532.57253	-3.00889
531.79775	33.832	533.60557	35.14882	531.79775	-3.2614	533.60557	-2.93705
532.83079	33.192	534.50948	34.58433	532.83079	-3.1916	534.50948	-2.8611
533.86383	32.515	535.54251	33.98221	533.86383	-3.1156	535.54251	-2.78926
534.76773	31.912	536.57555	33.45535	534.76773	-3.0417	536.57555	-2.72152
535.80077	31.273	537.60859	32.89087	535.80077	-2.9658	537.60859	-2.65173
536.83381	30.708	538.51249	32.36401	536.83381	-2.8981	538.51249	-2.59015
537.73772	30.106	539.54553	31.83715	537.73772	-2.8303	539.54553	-2.53472
538.77075	29.542	540.57857	31.34793	538.77075	-2.7667	540.57857	-2.4793
539.80379	28.977	541.48248	30.85871	539.80379	-2.703	541.48248	-2.42593
540.83683	28.45	542.51551	30.36948	540.83683	-2.6374	542.51551	-2.37667
541.74073	27.923	543.54855	29.88026	541.74073	-2.5799	543.54855	-2.32946
542.77377	27.397	544.58159	29.42867	542.77377	-2.5265	544.58159	-2.28019
543.80681	26.87	545.48549	28.97708	543.80681	-2.4752	545.48549	-2.23709

544.83985	26.38	546.51853	28.52549	544.83985	-2.428	546.51853	-2.18577
545.74375	25.891	547.55157	28.0739	545.74375	-2.3828	547.55157	-2.14061
546.77679	25.44	548.58461	27.65994	546.77679	-2.3377	548.58461	-2.09545
547.80983	24.95	549.48851	27.20835	547.80983	-2.2966	549.48851	-2.05234
548.84286	24.499	550.52155	26.83202	548.84286	-2.2597	550.52155	-2.00924
549.74677	24.047	551.55459	26.41806	549.74677	-2.2186	551.55459	-1.97024
550.77981	23.596	552.58762	26.04174	550.77981	-2.1796	552.58762	-1.93534
551.81285	23.182	553.49153	25.62778	551.81285	-2.1406	553.49153	-1.89634
552.84588	22.73	554.52457	25.25145	552.84588	-2.1016	554.52457	-1.8635
553.74979	22.316	555.55761	24.91276	553.74979	-2.0626	555.55761	-1.83065
554.78283	21.902	556.59064	24.53644	554.78283	-2.0216	556.59064	-1.79781
555.81586	21.526	557.49455	24.16011	555.81586	-1.9785	557.49455	-1.76291
556.8489	21.112	558.52759	23.82142	556.8489	-1.9395	558.52759	-1.72597
557.75281	20.736	559.56062	23.48272	557.75281	-1.9046	559.56062	-1.68902
558.78585	20.359	560.59366	23.14403	558.78585	-1.8656	560.59366	-1.65207
559.81888	19.983	561.49757	22.80534	559.81888	-1.8224	561.49757	-1.61512
560.85192	19.644	562.53061	22.50428	560.85192	-1.7793	562.53061	-1.57612
561.75583	19.268	563.56364	22.16558	561.75583	-1.7383	563.56364	-1.53507
562.78886	18.929	564.59668	21.86452	562.78886	-1.7013	564.59668	-1.49401
563.8219	18.59	565.50059	21.56346	563.8219	-1.6664	565.50059	-1.45706
564.85494	18.252	566.53362	21.30003	564.85494	-1.6254	566.53362	-1.42012
565.75885	17.951	567.56666	20.99897	565.75885	-1.5802	567.56666	-1.37701
566.79188	17.612	568.5997	20.73555	566.79188	-1.5392	568.5997	-1.3339
567.82492	17.311	569.50361	20.47212	567.82492	-1.5043	569.50361	-1.28874
568.85796	17.01	570.53664	20.20869	568.85796	-1.4694	570.53664	-1.24564
569.76186	16.709	571.56968	19.98289	569.76186	-1.4283	571.56968	-1.20048
570.7949	16.445	572.60272	19.71947	570.7949	-1.3832	572.60272	-1.15737
571.82794	16.182	573.50662	19.49367	571.82794	-1.3339	573.50662	-1.11016
572.86098	15.919	574.53966	19.30551	572.86098	-1.2867	574.53966	-1.06295
573.76488	15.655	575.5727	19.07971	573.76488	-1.2374	575.5727	-1.01574
574.79792	15.392	576.60574	18.89155	574.79792	-1.1861	576.60574	-0.97468
575.83096	15.166	577.50964	18.70339	575.83096	-1.1307	577.50964	-0.93158
576.86399	14.94	578.54268	18.51522	576.86399	-1.0753	578.54268	-0.88847
577.7679	14.752	579.57572	18.32706	577.7679	-1.026	579.57572	-0.84947
578.80094	14.526	580.60875	18.17653	578.80094	-0.9747	580.60875	-0.80431
579.83398	14.338	581.51266	18.026	579.83398	-0.9234	581.51266	-0.7612
580.73788	14.187	582.5457	17.87547	580.73788	-0.8782	582.5457	-0.7222
581.77092	13.999	583.57874	17.72494	581.77092	-0.8331	583.57874	-0.6832
582.80396	13.849	584.48264	17.61204	582.80396	-0.7899	584.48264	-0.6401
583.83699	13.698	585.51568	17.46151	583.83699	-0.7468	585.51568	-0.60109
584.7409	13.548	586.54872	17.34861	584.7409	-0.7037	586.54872	-0.56209
585.77394	13.397	587.58175	17.23572	585.77394	-0.6606	587.58175	-0.52309
586.80698	13.284	588.48566	17.16045	586.80698	-0.6216	588.48566	-0.48614
587.84001	13.171	589.5187	17.04755	587.84001	-0.5826	589.5187	-0.4533
588.74392	13.059	590.55174	16.97229	588.74392	-0.5436	590.55174	-0.42046
589.77696	12.946	591.58477	16.89702	589.77696	-0.5046	591.58477	-0.39172
590.80999	12.833	592.48868	16.82176	590.80999	-0.4677	592.48868	-0.36504
591.84303	12.757	593.52172	16.74649	591.84303	-0.4348	593.52172	-0.33219
592.74694	12.682	594.55475	16.67123	592.74694	-0.404	594.55475	-0.30551
593.77998	12.607	595.58779	16.6336	593.77998	-0.3733	595.58779	-0.28293
594.81301	12.532	596.4917	16.55833	594.81301	-0.3445	596.4917	-0.26035
595.84605	12.456	597.52474	16.5207	595.84605	-0.3137	597.52474	-0.23982
596.74996	12.419	598.55777	16.48307	596.74996	-0.2809	598.55777	-0.21724
597.783	12.343	599.59081	16.44543	597.783	-0.2562	599.59081	-0.19261
598.81603	12.306	600.49472	16.4078	598.81603	-0.2296	600.49472	-0.17414
599.84907	12.268	601.52775	16.37017	599.84907	-0.209	601.52775	-0.15771
600.75298	12.231	602.56079	16.33254	600.75298	-0.1865	602.56079	-0.1454
601.78601	12.193	603.59383	16.33254	601.78601	-0.1618	603.59383	-0.13103

602.81905	12.155	604.49774	16.2949	602.81905	-0.1454	604.49774	-0.11666
603.85209	12.118	605.53077	16.2949	603.85209	-0.131	605.53077	-0.10434
604.756	12.118	606.56381	16.25727	604.756	-0.1249	606.56381	-0.09819
605.78903	12.08	607.59685	16.21964	605.78903	-0.1146	607.59685	-0.09613
606.82207	12.08	608.50075	16.21964	606.82207	-0.1043	608.50075	-0.08998
607.85511	12.042	609.53379	16.21964	607.85511	-0.0941	609.53379	-0.08382
608.75901	12.042	610.56683	16.182	608.75901	-0.0879	610.56683	-0.07766
609.79205	12.005	611.59987	16.182	609.79205	-0.0838	611.59987	-0.0715
610.82509	12.005	612.50377	16.14437	610.82509	-0.0777	612.50377	-0.06945
611.85813	12.005	613.53681	16.14437	611.85813	-0.0715	613.53681	-0.06534
612.76203	11.967	614.56985	16.14437	612.76203	-0.0653	614.56985	-0.05919
613.79507	11.967	615.60288	16.10674	613.79507	-0.0592	615.60288	-0.05303
614.82811	11.967	616.50679	16.10674	614.82811	-0.0551	616.50679	-0.04687
615.86114	11.93	617.53983	16.10674	615.86114	-0.051	617.53983	-0.04482
616.76505	11.93	618.57287	16.10674	616.76505	-0.0448	618.57287	-0.04482
617.79809	11.93	619.6059	16.06911	617.79809	-0.0428	619.6059	-0.04276
618.83113	11.93	620.50981	16.06911	618.83113	-0.0448	620.50981	-0.03866
619.86416	11.892	621.54285	16.06911	619.86416	-0.0469	621.54285	-0.03455
620.76807	11.892	622.57588	16.06911	620.76807	-0.051	622.57588	-0.03455
621.80111	11.892	623.60892	16.06911	621.80111	-0.0551	623.60892	-0.03455
622.83414	11.892	624.51283	16.03147	622.83414	-0.0571	624.51283	-0.03661
623.73805	11.854	625.54587	16.03147	623.73805	-0.0592	625.54587	-0.04071
624.77109	11.854	626.5789	16.03147	624.77109	-0.0653	626.5789	-0.04071
625.80413	11.854	627.48281	16.03147	625.80413	-0.0695	627.48281	-0.04276
626.83716	11.817	628.51585	16.03147	626.83716	-0.0633	628.51585	-0.04687
627.74107	11.817	629.54888	15.99384	627.74107	-0.0571	629.54888	-0.05303
628.77411	11.817	630.58192	15.99384	628.77411	-0.051	630.58192	-0.06124
629.80714	11.779	631.48583	15.99384	629.80714	-0.0448	631.48583	-0.06534
630.84018	11.779	632.51887	15.95621	630.84018	-0.0448	632.51887	-0.06945
631.74409	11.779	633.5519	15.95621	631.74409	-0.0489	633.5519	-0.07355
632.77713	11.779	634.58494	15.95621	632.77713	-0.0448	634.58494	-0.07561
633.81016	11.779	635.48885	15.91858	633.81016	-0.0387	635.48885	-0.07561
634.8432	11.741	636.52188	15.91858	634.8432	-0.0428	636.52188	-0.07561
635.74711	11.741	637.55492	15.88094	635.74711	-0.051	637.55492	-0.07971
636.78014	11.741	638.58796	15.88094	636.78014	-0.0612	638.58796	-0.07766
637.81318	11.704	639.49187	15.88094	637.81318	-0.0653	639.49187	-0.07561
638.84622	11.704	640.5249	15.84331	638.84622	-0.0653	640.5249	-0.07355
639.75013	11.704	641.55794	15.84331	639.75013	-0.0633	641.55794	-0.06945
640.78316	11.666	642.59098	15.80568	640.78316	-0.0633	642.59098	-0.0674
641.8162	11.666	643.49489	15.80568	641.8162	-0.0674	643.49489	-0.0674
642.84924	11.666	644.52792	15.80568	642.84924	-0.0674	644.52792	-0.0674
643.75314	11.628	645.56096	15.76805	643.75314	-0.0633	645.56096	-0.05713
644.78618	11.628	646.594	15.76805	644.78618	-0.0551	646.594	-0.05303
645.81922	11.628	647.4979	15.76805	645.81922	-0.051	647.4979	-0.05303
646.85226	11.591	648.53094	15.73041	646.85226	-0.0489	648.53094	-0.05303
647.75616	11.591	649.56398	15.73041	647.75616	-0.051	649.56398	-0.05303
648.7892	11.591	650.59701	15.73041	648.7892	-0.051	650.59701	-0.05098
649.82224	11.591	651.50092	15.73041	649.82224	-0.0489	651.50092	-0.04687
650.85527	11.553	652.53396	15.69278	650.85527	-0.0469	652.53396	-0.04482
651.75918	11.553	653.567	15.69278	651.75918	-0.0489	653.567	-0.05098
652.79222	11.553	654.60003	15.69278	652.79222	-0.053	654.60003	-0.05713
653.82526	11.553	655.50394	15.69278	653.82526	-0.0551	655.50394	-0.05508
654.85829	11.516	656.53698	15.69278	654.85829	-0.0551	656.53698	-0.05508
655.7622	11.516	657.57002	15.65515	655.7622	-0.053	657.57002	-0.05508
656.79524	11.516	658.60305	15.65515	656.79524	-0.051	658.60305	-0.05713
657.82827	11.478	659.50696	15.61752	657.82827	-0.0489	659.50696	-0.06329
658.86131	11.478	660.54	15.61752	658.86131	-0.0489	660.54	-0.06534
659.76522	11.478	661.57303	15.61752	659.76522	-0.051	661.57303	-0.05919

660.79826	11.478	662.60607	15.57988	660.79826	-0.0489	662.60607	-0.05713
661.83129	11.44	663.50998	15.57988	661.83129	-0.051	663.50998	-0.05919
662.86433	11.44	664.54302	15.57988	662.86433	-0.053	664.54302	-0.06124
663.76824	11.44	665.57605	15.57988	663.76824	-0.0551	665.57605	-0.06124
664.80127	11.44	666.60909	15.54225	664.80127	-0.0571	666.60909	-0.05919
665.83431	11.403	667.513	15.54225	665.83431	-0.0571	667.513	-0.05303
666.73822	11.403	668.54603	15.54225	666.73822	-0.0592	668.54603	-0.05713
667.77126	11.403	669.57907	15.50462	667.77126	-0.0633	669.57907	-0.06329
668.80429	11.365	670.48298	15.50462	668.80429	-0.0674	670.48298	-0.06534
669.83733	11.365	671.51602	15.50462	669.83733	-0.0715	671.51602	-0.06534
670.74124	11.365	672.54905	15.46699	670.74124	-0.0736	672.54905	-0.06534
671.77427	11.327	673.58209	15.46699	671.77427	-0.0756	673.58209	-0.0674
672.80731	11.327	674.486	15.46699	672.80731	-0.0777	674.486	-0.0715
673.84035	11.29	675.51903	15.42935	673.84035	-0.0777	675.51903	-0.07971
674.74426	11.29	676.55207	15.42935	674.74426	-0.0756	676.55207	-0.07971
675.77729	11.252	677.58511	15.39172	675.77729	-0.0695	677.58511	-0.07561
676.81033	11.252	678.48902	15.39172	676.81033	-0.0633	678.48902	-0.0715
677.84337	11.252	679.52205	15.35409	677.84337	-0.0571	679.52205	-0.0715
678.74727	11.215	680.55509	15.35409	678.74727	-0.051	680.55509	-0.06945
679.78031	11.215	681.58813	15.35409	679.78031	-0.0489	681.58813	-0.06534
680.81335	11.215	682.49203	15.31646	680.81335	-0.0428	682.49203	-0.06124
681.84639	11.215	683.52507	15.31646	681.84639	-0.0428	683.52507	-0.05508
682.75029	11.215	684.55811	15.31646	682.75029	-0.0448	684.55811	-0.05303
683.78333	11.177	685.59115	15.31646	683.78333	-0.051	685.59115	-0.05508
684.81637	11.177	686.49505	15.27882	684.81637	-0.0551	686.49505	-0.05919
685.8494	11.177	687.52809	15.27882	685.8494	-0.0551	687.52809	-0.05919
686.75331	11.139	688.56113	15.27882	686.75331	-0.0551	688.56113	-0.05713
687.78635	11.139	689.59416	15.24119	687.78635	-0.0571	689.59416	-0.05508
688.81939	11.139	690.49807	15.24119	688.81939	-0.0592	690.49807	-0.05303
689.85242	11.139	691.53111	15.24119	689.85242	-0.0592	691.53111	-0.05508
690.75633	11.102	692.56415	15.20356	690.75633	-0.0551	692.56415	-0.05713
691.78937	11.102	693.59718	15.20356	691.78937	-0.0489	693.59718	-0.05098
692.8224	11.102	694.50109	15.20356	692.8224	-0.0448	694.50109	-0.04482
693.85544	11.064	695.53413	15.20356	693.85544	-0.0448	695.53413	-0.04687
694.75935	11.064	696.56716	15.16593	694.75935	-0.0469	696.56716	-0.05098
695.79239	11.064	697.6002	15.16593	695.79239	-0.0448	697.6002	-0.05508
696.82542	11.064	698.50411	15.16593	696.82542	-0.0448	698.50411	-0.06124
697.85846	11.064	699.53715	15.16593	697.85846	-0.0469	699.53715	-0.06124
698.76237	11.026	700.57018	15.12829	698.76237	-0.0469	700.57018	-0.05919
699.7954	11.026	701.60322	15.12829	699.7954	-0.051	701.60322	-0.06534
700.82844	11.026	702.50713	15.09066	700.82844	-0.0571	702.50713	-0.06945
701.86148	10.989	703.54016	15.09066	701.86148	-0.0571	703.54016	-0.06945
702.76539	10.989	704.5732	15.09066	702.76539	-0.0551	704.5732	-0.0674
703.79842	10.989	705.60624	15.05303	703.79842	-0.0551	705.60624	-0.06534
704.83146	10.951	706.51015	15.05303	704.83146	-0.0489	706.51015	-0.05919
705.8645	10.951	707.54318	15.05303	705.8645	-0.0428	707.54318	-0.05508
706.76841	10.951	708.57622	15.0154	706.76841	-0.0428	708.57622	-0.05303
707.80144	10.951	709.60926	15.0154	707.80144	-0.0387	709.60926	-0.05098
708.83448	10.951	710.51316	15.0154	708.83448	-0.0346	710.51316	-0.04892
709.73839	10.951	711.5462	15.0154	709.73839	-0.0325	711.5462	-0.04892
710.77142	10.913	712.57924	15.0154	710.77142	-0.0346	712.57924	-0.05098
711.80446	10.913	713.48315	14.97776	711.80446	-0.0325	713.48315	-0.04687
712.8375	10.913	714.51618	14.97776	712.8375	-0.0387	714.51618	-0.04276
713.74141	10.913	715.54922	14.97776	713.74141	-0.0448	715.54922	-0.04071
714.77444	10.913	716.58226	14.94013	714.77444	-0.0469	716.58226	-0.03661
715.80748	10.876	717.48616	14.94013	715.80748	-0.0448	717.48616	-0.03045
716.84052	10.876	718.5192	14.94013	716.84052	-0.0428	718.5192	-0.02429
717.74442	10.876	719.55224	14.94013	717.74442	-0.0387	719.55224	-0.02018

718.77746	10.838	720.58528	14.94013	718.77746	-0.0366	720.58528	-0.01403
719.8105	10.838	721.48918	14.94013	719.8105	-0.0366	721.48918	-0.01608
720.84354	10.838	722.52222	14.94013	720.84354	-0.0305	722.52222	-0.02018
721.74744	10.838	723.55526	14.94013	721.74744	-0.0222	723.55526	-0.02429
722.78048	10.838	724.58829	14.94013	722.78048	-0.0222	724.58829	-0.02634
723.81352	10.838	725.4922	14.94013	723.81352	-0.0243	725.4922	-0.0284
724.84655	10.838	726.52524	14.9025	724.84655	-0.0284	726.52524	-0.02634
725.75046	10.838	727.55828	14.9025	725.75046	-0.0305	727.55828	-0.02429
726.7835	10.838	728.59131	14.9025	726.7835	-0.0263	728.59131	-0.02018
727.81654	10.801	729.49522	14.9025	727.81654	-0.0243	729.49522	-0.02018
728.84957	10.801	730.52826	14.9025	728.84957	-0.0284	730.52826	-0.01608
728.84957	10.801	730.52826	14.9025	728.84957	-0.0284	730.52826	-0.01608
729.75348	10.801	731.56129	14.9025	729.75348	-0.0305	731.56129	-0.01403
730.78652	10.801	732.59433	14.9025	730.78652	-0.0284	732.59433	-0.01403
731.81955	10.801	733.49824	14.9025	731.81955	-0.0243	733.49824	-0.01608
732.85259	10.801	734.53128	14.9025	732.85259	-0.0222	734.53128	-0.02224
733.7565	10.763	735.56431	14.86486	733.7565	-0.0243	735.56431	-0.0284
734.78954	10.763	736.59735	14.86486	734.78954	-0.0305	736.59735	-0.0325
735.82257	10.763	737.50126	14.86486	735.82257	-0.0346	737.50126	-0.03045
736.85561	10.763	738.53429	14.86486	736.85561	-0.0346	738.53429	-0.03045
737.75952	10.763	739.56733	14.86486	737.75952	-0.0325	739.56733	-0.0284
738.79255	10.763	740.60037	14.82723	738.79255	-0.0284	740.60037	-0.02634
739.82559	10.725	741.50428	14.82723	739.82559	-0.0263	741.50428	-0.02429
740.85863	10.725	742.53731	14.82723	740.85863	-0.0243	742.53731	-0.02018
741.76254	10.725	743.57035	14.82723	741.76254	-0.0202	743.57035	-0.01608
742.79557	10.725	744.60339	14.82723	742.79557	-0.012	744.60339	-0.01197
743.82861	10.725	745.50729	14.82723	743.82861	-0.0058	745.50729	-0.01197
744.86165	10.725	746.54033	14.82723	744.86165	0.00034	746.54033	-0.00992
745.76555	10.725	747.57337	14.82723	745.76555	0.00034	747.57337	-0.00787
746.79859	10.725	748.60641	14.82723	746.79859	-0.0038	748.60641	-0.00582
747.83163	10.725	749.51031	14.82723	747.83163	-0.0079	749.51031	-0.00376
748.86467	10.725	750.54335	14.82723	748.86467	-0.0099	750.54335	-0.00376
749.76857	10.725	751.57639	14.82723	749.76857	-0.014	751.57639	-0.00376
750.80161	10.725	752.60942	14.82723	750.80161	-0.0161	752.60942	-0.00376
751.83465	10.725	753.51333	14.82723	751.83465	-0.0181	753.51333	-0.00171
752.73855	10.725	754.54637	14.82723	752.73855	-0.0202	754.54637	-0.00171
753.77159	10.688	755.57941	14.82723	753.77159	-0.0181	755.57941	-0.00171
754.80463	10.688	756.48331	14.82723	754.80463	-0.014	756.48331	0.0003421
755.83767	10.688	757.51635	14.82723	755.83767	-0.012	757.51635	0.0003421
756.74157	10.688	758.54939	14.82723	756.74157	-0.0079	758.54939	0.0065
757.77461	10.688	759.58242	14.82723	757.77461	-0.0017	759.58242	0.01471
758.80765	10.688	760.48633	14.82723	758.80765	0.00239	760.48633	0.01882
759.84068	10.688	761.51937	14.82723	759.84068	0.00034	761.51937	0.01882
760.74459	10.688	762.55241	14.82723	760.74459	-0.0017	762.55241	0.01676
761.77763	10.688	763.58544	14.82723	761.77763	0.00034	763.58544	0.01676
762.81067	10.725	764.48935	14.86486	762.81067	0.00239	764.48935	0.01676
763.8437	10.688	765.52239	14.86486	763.8437	0.00239	765.52239	0.01266
764.74761	10.688	766.55543	14.86486	764.74761	0.00445	766.55543	0.00239
765.78065	10.688	767.58846	14.86486	765.78065	-0.0038	767.58846	-0.00992
766.81368	10.688	768.49237	14.86486	766.81368	-0.0079	768.49237	-0.01608
767.84672	10.688	769.52541	14.86486	767.84672	-0.0038	769.52541	-0.01608
768.75063	10.688	770.55844	14.82723	768.75063	0.00034	770.55844	-0.01403
769.78367	10.688	771.59148	14.82723	769.78367	-0.0017	771.59148	-0.01608
770.8167	10.688	772.49539	14.82723	770.8167	-0.0017	772.49539	-0.02018
771.84974	10.688	773.52843	14.82723	771.84974	-0.0038	773.52843	-0.02018
772.75365	10.688	774.56146	14.82723	772.75365	-0.0017	774.56146	-0.01608
773.78668	10.688	775.5945	14.82723	773.78668	0.00034	775.5945	-0.01197
774.81972	10.688	776.49841	14.82723	774.81972	0.00239	776.49841	-0.00787

775.85276	10.688	777.53144	14.82723	775.85276	0.00034	777.53144	-0.00787
776.75667	10.688	778.56448	14.82723	776.75667	-0.0038	778.56448	-0.00787
777.7897	10.688	779.59752	14.82723	777.7897	-0.0058	779.59752	-0.00787
778.82274	10.688	780.50143	14.82723	778.82274	-0.0038	780.50143	-0.00582
779.85578	10.688	781.53446	14.82723	779.85578	-0.0017	781.53446	0.0003421
780.75968	10.688	782.5675	14.82723	780.75968	-0.0038	782.5675	0.0065
781.79272	10.688	783.60054	14.82723	781.79272	-0.0058	783.60054	0.01266
782.82576	10.688	784.50444	14.82723	782.82576	-0.0079	784.50444	0.01471
783.8588	10.688	785.53748	14.82723	783.8588	-0.0038	785.53748	0.01882
784.7627	10.688	786.57052	14.82723	784.7627	-0.0017	786.57052	0.02087
785.79574	10.688	787.60356	14.82723	785.79574	-0.0017	787.60356	0.01882
786.82878	10.688	788.50746	14.82723	786.82878	-0.0079	788.50746	0.01676
787.86181	10.688	789.5405	14.82723	787.86181	-0.0161	789.5405	0.01266
788.76572	10.688	790.57354	14.82723	788.76572	-0.014	790.57354	0.0065
789.79876	10.688	791.60657	14.82723	789.79876	-0.0099	791.60657	-0.00376
790.8318	10.688	792.51048	14.82723	790.8318	-0.014	792.51048	-0.00992
791.86483	10.688	793.54352	14.82723	791.86483	-0.0079	793.54352	-0.00582
792.76874	10.688	794.57656	14.82723	792.76874	-0.0038	794.57656	-0.00992
793.80178	10.688	795.60959	14.82723	793.80178	-0.0058	795.60959	-0.00787
794.83481	10.65	796.5135	14.82723	794.83481	0.0065	796.5135	0.01676
795.73872	10.688			795.73872	0.00445		
796.77176	10.688			796.77176	-0.0305		

Infrared spectra of Brown coal

Wavenumbers cm-1	Absorbance % Raw coal	Wavenumbers cm-1	Absorbance % Soaked coal
3997.96027	0.00012	3997.96027	-0.00023
3996.03169	-0.00001	3996.03169	-0.00039
3994.1031	-0.00004	3994.1031	-0.00039
3992.17451	0.00001	3992.17451	-0.00032
3990.24593	0.00001	3990.24593	-0.00034
3988.31734	0	3988.31734	-0.00036
3986.38875	0.00012	3986.38875	-0.00028
3984.46017	0.00025	3984.46017	-0.0002
3982.53158	0.00023	3982.53158	-0.0002
3980.60299	0.00015	3980.60299	-0.00018
3978.67441	0.00021	3978.67441	-0.0001
3976.74582	0.00035	3976.74582	-0.00007
3974.81723	0.00036	3974.81723	-0.00013
3972.88865	0.00034	3972.88865	-0.00012
3970.96006	0.0004	3970.96006	-0.00006
3969.03147	0.00042	3969.03147	-0.00006
3967.10289	0.00043	3967.10289	-0.00002
3965.1743	0.00061	3965.1743	0.00014
3963.24571	0.00075	3963.24571	0.00018
3961.31713	0.00063	3961.31713	0.00007
3959.38854	0.00054	3959.38854	0.00008
3957.45995	0.00056	3957.45995	0.00007
3955.53137	0.00056	3955.53137	0.00002
3953.60278	0.00073	3953.60278	0.00018
3951.67419	0.00107	3951.67419	0.00041
3949.74561	0.00099	3949.74561	0.00024
3947.81702	0.00055	3947.81702	0
3945.88843	0.00077	3945.88843	0.0004
3943.95985	0.00125	3943.95985	0.00064
3942.03126	0.00094	3942.03126	0.00026
3940.10267	0.00052	3940.10267	0.00009
3938.17409	0.00066	3938.17409	0.00027
3936.2455	0.00123	3936.2455	0.00068
3934.31691	0.00187	3934.31691	0.00101
3932.38832	0.00163	3932.38832	0.00059
3930.45974	0.00069	3930.45974	-0.00002

3928.53115	0.0007	3928.53115	0.00031
3926.60256	0.00144	3926.60256	0.0008
3924.67398	0.00134	3924.67398	0.00043
3922.74539	0.00097	3922.74539	0.00026
3920.8168	0.00157	3920.8168	0.00089
3918.88822	0.0022	3918.88822	0.00122
3916.95963	0.00176	3916.95963	0.00081
3915.03104	0.00118	3915.03104	0.0006
3913.10246	0.0013	3913.10246	0.0008
3911.17387	0.00142	3911.17387	0.00075
3909.24528	0.00157	3909.24528	0.00082
3907.3167	0.00235	3907.3167	0.00126
3905.38811	0.00285	3905.38811	0.00118
3903.45952	0.00236	3903.45952	0.00085
3901.53094	0.00178	3901.53094	0.00082
3899.60235	0.00098	3899.60235	0.00027
3897.67376	0.0003	3897.67376	0.00001
3895.74518	0.00135	3895.74518	0.00118
3893.81659	0.00307	3893.81659	0.00207
3891.888	0.00254	3891.888	0.00091
3889.95942	0.00134	3889.95942	0.0004
3888.03083	0.00227	3888.03083	0.00159
3886.10224	0.00284	3886.10224	0.00154
3884.17366	0.00221	3884.17366	0.00115
3882.24507	0.00251	3882.24507	0.00154
3880.31648	0.00197	3880.31648	0.00084
3878.3879	0.00109	3878.3879	0.00068
3876.45931	0.00199	3876.45931	0.00158
3874.53072	0.00295	3874.53072	0.00179
3872.60214	0.00323	3872.60214	0.00179
3870.67355	0.00268	3870.67355	0.0011
3868.74496	0.00144	3868.74496	0.00061
3866.81638	0.00208	3866.81638	0.00173
3864.88779	0.00312	3864.88779	0.00205
3862.9592	0.00203	3862.9592	0.00092
3861.03062	0.00074	3861.03062	0.00032
3859.10203	0.00133	3859.10203	0.00125
3857.17344	0.00415	3857.17344	0.00332
3855.24486	0.00671	3855.24486	0.00413
3853.31627	0.00461	3853.31627	0.00176
3851.38768	0.00057	3851.38768	0
3849.4591	0.00014	3849.4591	0.00073
3847.53051	0.00203	3847.53051	0.00205

3845.60192	0.00333	3845.60192	0.00237
3843.67334	0.00333	3843.67334	0.00193
3841.74475	0.00329	3841.74475	0.00197
3839.81616	0.00347	3839.81616	0.00194
3837.88758	0.00256	3837.88758	0.00111
3835.95899	0.00168	3835.95899	0.00103
3834.0304	0.00205	3834.0304	0.00174
3832.10182	0.00238	3832.10182	0.00186
3830.17323	0.00236	3830.17323	0.00185
3828.24464	0.00235	3828.24464	0.00169
3826.31606	0.0022	3826.31606	0.00148
3824.38747	0.0032	3824.38747	0.0024
3822.45888	0.00401	3822.45888	0.00232
3820.5303	0.0024	3820.5303	0.00099
3818.60171	0.00215	3818.60171	0.00184
3816.67312	0.00306	3816.67312	0.00236
3814.74453	0.00191	3814.74453	0.00129
3812.81595	0.00125	3812.81595	0.00137
3810.88736	0.00279	3810.88736	0.00268
3808.95877	0.00402	3808.95877	0.00286
3807.03019	0.0029	3807.03019	0.00142
3805.1016	0.00234	3805.1016	0.00169
3803.17301	0.00356	3803.17301	0.00257
3801.24443	0.00284	3801.24443	0.00138
3799.31584	0.00174	3799.31584	0.00123
3797.38725	0.0023	3797.38725	0.00202
3795.45867	0.00241	3795.45867	0.00199
3793.53008	0.00233	3793.53008	0.00206
3791.60149	0.00278	3791.60149	0.00234
3789.67291	0.00315	3789.67291	0.00247
3787.74432	0.00335	3787.74432	0.00251
3785.81573	0.00306	3785.81573	0.00208
3783.88715	0.00269	3783.88715	0.00191
3781.95856	0.00332	3781.95856	0.00256
3780.02997	0.0036	3780.02997	0.00243
3778.10139	0.00249	3778.10139	0.00161
3776.1728	0.0019	3776.1728	0.00163
3774.24421	0.00273	3774.24421	0.00247
3772.31563	0.00383	3772.31563	0.00297
3770.38704	0.00369	3770.38704	0.00238
3768.45845	0.00284	3768.45845	0.0019
3766.52987	0.00238	3766.52987	0.00176
3764.60128	0.00209	3764.60128	0.00166

3762.67269	0.00278	3762.67269	0.0024
3760.74411	0.00363	3760.74411	0.00261
3758.81552	0.0025	3758.81552	0.00134
3756.88693	0.00129	3756.88693	0.00091
3754.95835	0.00263	3754.95835	0.00236
3753.02976	0.00443	3753.02976	0.00299
3751.10117	0.00281	3751.10117	0.00083
3749.17259	0.00044	3749.17259	0
3747.244	0.00229	3747.244	0.00254
3745.31541	0.00402	3745.31541	0.00297
3743.38683	0.00155	3743.38683	0.00059
3741.45824	0.0001	3741.45824	0.00035
3739.52965	0.00221	3739.52965	0.00219
3737.60107	0.00447	3737.60107	0.00315
3735.67248	0.00407	3735.67248	0.00213
3733.74389	0.00189	3733.74389	0.00066
3731.81531	0.00058	3731.81531	0.00029
3729.88672	0.00124	3729.88672	0.00145
3727.95813	0.00272	3727.95813	0.00243
3726.02955	0.00304	3726.02955	0.00217
3724.10096	0.00242	3724.10096	0.00165
3722.17237	0.00192	3722.17237	0.00139
3720.24379	0.00132	3720.24379	0.00103
3718.3152	0.0008	3718.3152	0.00068
3716.38661	0.00139	3716.38661	0.0013
3714.45803	0.00316	3714.45803	0.0024
3712.52944	0.00368	3712.52944	0.00201
3710.60085	0.00156	3710.60085	0.00025
3708.67227	-0.00022	3708.67227	-0.00021
3706.74368	0.00032	3706.74368	0.00098
3704.81509	0.00218	3704.81509	0.00238
3702.88651	0.00303	3702.88651	0.00237
3700.95792	0.00218	3700.95792	0.00154
3699.02933	0.00153	3699.02933	0.00151
3697.10074	0.00155	3697.10074	0.00168
3695.17216	0.0018	3695.17216	0.00184
3693.24357	0.0028	3693.24357	0.00249
3691.31498	0.00376	3691.31498	0.00258
3689.3864	0.00255	3689.3864	0.001
3687.45781	0.00035	3687.45781	-0.00014
3685.52922	-0.00006	3685.52922	0.00036
3683.60064	0.00069	3683.60064	0.00101
3681.67205	0.00119	3681.67205	0.00104

3679.74346	0.00193	3679.74346	0.00146
3677.81488	0.00301	3677.81488	0.00176
3675.88629	0.00173	3675.88629	0.00004
3673.9577	0	3673.9577	0
3672.02912	0.00181	3672.02912	0.00243
3670.10053	0.00296	3670.10053	0.00257
3668.17194	0.00163	3668.17194	0.0018
3666.24336	0.00175	3666.24336	0.00274
3664.31477	0.00308	3664.31477	0.00379
3662.38618	0.00375	3662.38618	0.00409
3660.4576	0.0044	3660.4576	0.00472
3658.52901	0.00526	3658.52901	0.00537
3656.60042	0.00468	3656.60042	0.00481
3654.67184	0.00406	3654.67184	0.00521
3652.74325	0.00636	3652.74325	0.00749
3650.81466	0.00895	3650.81466	0.00858
3648.88608	0.00794	3648.88608	0.00754
3646.95749	0.006	3646.95749	0.00736
3645.0289	0.00669	3645.0289	0.00924
3643.10032	0.0088	3643.10032	0.01116
3641.17173	0.0102	3641.17173	0.01199
3639.24314	0.0108	3639.24314	0.01244
3637.31456	0.0113	3637.31456	0.01307
3635.38597	0.01168	3635.38597	0.01362
3633.45738	0.0126	3633.45738	0.01481
3631.5288	0.01496	3631.5288	0.01691
3629.60021	0.01605	3629.60021	0.01724
3627.67162	0.01414	3627.67162	0.01631
3625.74304	0.01371	3625.74304	0.01749
3623.81445	0.01611	3623.81445	0.01998
3621.88586	0.01877	3621.88586	0.02166
3619.95728	0.01932	3619.95728	0.02153
3618.02869	0.01833	3618.02869	0.02115
3616.1001	0.01863	3616.1001	0.0222
3614.17152	0.01984	3614.17152	0.02318
3612.24293	0.02073	3612.24293	0.02396
3610.31434	0.02185	3610.31434	0.0252
3608.38576	0.02236	3608.38576	0.02566
3606.45717	0.02219	3606.45717	0.02587
3604.52858	0.02309	3604.52858	0.02707
3602.6	0.02463	3602.6	0.02824
3600.67141	0.02534	3600.67141	0.02863
3598.74282	0.02585	3598.74282	0.02927

3596.81424	0.02677	3596.81424	0.03025
3594.88565	0.02707	3594.88565	0.03058
3592.95706	0.0272	3592.95706	0.03115
3591.02848	0.02892	3591.02848	0.03298
3589.09989	0.03094	3589.09989	0.03423
3587.1713	0.03078	3587.1713	0.0338
3585.24272	0.03023	3585.24272	0.03408
3583.31413	0.03141	3583.31413	0.03571
3581.38554	0.03307	3581.38554	0.03708
3579.45695	0.03409	3579.45695	0.03785
3577.52837	0.03474	3577.52837	0.0385
3575.59978	0.0352	3575.59978	0.03905
3573.67119	0.03552	3573.67119	0.03957
3571.74261	0.0366	3571.74261	0.04073
3569.81402	0.03855	3569.81402	0.04219
3567.88543	0.03939	3567.88543	0.04243
3565.95685	0.0386	3565.95685	0.04215
3564.02826	0.03887	3564.02826	0.04329
3562.09967	0.04058	3562.09967	0.04504
3560.17109	0.04193	3560.17109	0.04608
3558.2425	0.04272	3558.2425	0.04683
3556.31391	0.04375	3556.31391	0.04787
3554.38533	0.04481	3554.38533	0.04872
3552.45674	0.04527	3552.45674	0.0491
3550.52815	0.04588	3550.52815	0.04993
3548.59957	0.04731	3548.59957	0.0513
3546.67098	0.04841	3546.67098	0.05209
3544.74239	0.04866	3544.74239	0.05241
3542.81381	0.04914	3542.81381	0.05326
3540.88522	0.05035	3540.88522	0.05463
3538.95663	0.0517	3538.95663	0.05581
3537.02805	0.05253	3537.02805	0.05642
3535.09946	0.05301	3535.09946	0.05696
3533.17087	0.05378	3533.17087	0.05791
3531.24229	0.05484	3531.24229	0.05893
3529.3137	0.05564	3529.3137	0.05962
3527.38511	0.05624	3527.38511	0.06024
3525.45653	0.05699	3525.45653	0.06105
3523.52794	0.05771	3523.52794	0.06183
3521.59935	0.05841	3521.59935	0.0627
3519.67077	0.05929	3519.67077	0.06368
3517.74218	0.06014	3517.74218	0.06448
3515.81359	0.06081	3515.81359	0.06514

3513.88501	0.06158	3513.88501	0.06593
3511.95642	0.06238	3511.95642	0.0667
3510.02783	0.06282	3510.02783	0.06715
3508.09925	0.0632	3508.09925	0.06774
3506.17066	0.06414	3506.17066	0.06869
3504.24207	0.06504	3504.24207	0.06928
3502.31349	0.06527	3502.31349	0.06956
3500.3849	0.06568	3500.3849	0.07032
3498.45631	0.06661	3498.45631	0.07128
3496.52773	0.06737	3496.52773	0.07188
3494.59914	0.06779	3494.59914	0.0724
3492.67055	0.06834	3492.67055	0.07313
3490.74197	0.06906	3490.74197	0.07386
3488.81338	0.06955	3488.81338	0.07428
3486.88479	0.06988	3486.88479	0.07469
3484.95621	0.07053	3484.95621	0.07541
3483.02762	0.07124	3483.02762	0.07597
3481.09903	0.07155	3481.09903	0.07623
3479.17045	0.07193	3479.17045	0.07678
3477.24186	0.07268	3477.24186	0.07756
3475.31327	0.07329	3475.31327	0.07811
3473.38469	0.07364	3473.38469	0.07851
3471.4561	0.07401	3471.4561	0.07893
3469.52751	0.07447	3469.52751	0.07929
3467.59893	0.0749	3467.59893	0.0796
3465.67034	0.07536	3465.67034	0.08008
3463.74175	0.07588	3463.74175	0.0806
3461.81316	0.07622	3461.81316	0.08094
3459.88458	0.0765	3459.88458	0.08131
3457.95599	0.0769	3457.95599	0.08179
3456.0274	0.07726	3456.0274	0.08216
3454.09882	0.07747	3454.09882	0.08235
3452.17023	0.0777	3452.17023	0.08256
3450.24164	0.0782	3450.24164	0.08298
3448.31306	0.07855	3448.31306	0.08309
3446.38447	0.07844	3446.38447	0.08298
3444.45588	0.07854	3444.45588	0.08332
3442.5273	0.07888	3442.5273	0.08366
3440.59871	0.07902	3440.59871	0.08374
3438.67012	0.07913	3438.67012	0.08379
3436.74154	0.07923	3436.74154	0.08378
3434.81295	0.07926	3434.81295	0.08381
3432.88436	0.07927	3432.88436	0.08386

3430.95578	0.0792	3430.95578	0.08381
3429.02719	0.07909	3429.02719	0.08375
3427.0986	0.07895	3427.0986	0.0837
3425.17002	0.07888	3425.17002	0.08371
3423.24143	0.07888	3423.24143	0.08368
3421.31284	0.07865	3421.31284	0.08339
3419.38426	0.07823	3419.38426	0.08307
3417.45567	0.07801	3417.45567	0.08295
3415.52708	0.07787	3415.52708	0.08278
3413.5985	0.07757	3413.5985	0.08246
3411.66991	0.07718	3411.66991	0.08212
3409.74132	0.07679	3409.74132	0.08179
3407.81274	0.0764	3407.81274	0.08146
3405.88415	0.07603	3405.88415	0.08114
3403.95556	0.07558	3403.95556	0.08072
3402.02698	0.07505	3402.02698	0.08021
3400.09839	0.07463	3400.09839	0.07982
3398.1698	0.07419	3398.1698	0.07943
3396.24122	0.07367	3396.24122	0.07897
3394.31263	0.07321	3394.31263	0.07849
3392.38404	0.07259	3392.38404	0.07787
3390.45546	0.07187	3390.45546	0.07733
3388.52687	0.07135	3388.52687	0.07696
3386.59828	0.07086	3386.59828	0.07651
3384.6697	0.0702	3384.6697	0.07589
3382.74111	0.06947	3382.74111	0.07526
3380.81252	0.0688	3380.81252	0.07465
3378.88394	0.06813	3378.88394	0.07402
3376.95535	0.0674	3376.95535	0.07336
3375.02676	0.06659	3375.02676	0.0727
3373.09818	0.06572	3373.09818	0.07197
3371.16959	0.06486	3371.16959	0.0712
3369.241	0.06404	3369.241	0.07044
3367.31242	0.0632	3367.31242	0.06967
3365.38383	0.06231	3365.38383	0.06895
3363.45524	0.06143	3363.45524	0.06824
3361.52666	0.06055	3361.52666	0.06747
3359.59807	0.05966	3359.59807	0.06674
3357.66948	0.05882	3357.66948	0.06604
3355.7409	0.05795	3355.7409	0.06526
3353.81231	0.05706	3353.81231	0.06445
3351.88372	0.05623	3351.88372	0.06368
3349.95514	0.05544	3349.95514	0.06296

3348.02655	0.05462	3348.02655	0.06228
3346.09796	0.05377	3346.09796	0.06158
3344.16937	0.05294	3344.16937	0.06084
3342.24079	0.05215	3342.24079	0.06009
3340.3122	0.05144	3340.3122	0.05937
3338.38361	0.05078	3338.38361	0.05871
3336.45503	0.05003	3336.45503	0.05803
3334.52644	0.0492	3334.52644	0.05733
3332.59785	0.04844	3332.59785	0.05671
3330.66927	0.04778	3330.66927	0.0561
3328.74068	0.04714	3328.74068	0.05546
3326.81209	0.04647	3326.81209	0.05478
3324.88351	0.04581	3324.88351	0.05411
3322.95492	0.04519	3322.95492	0.05352
3321.02633	0.04459	3321.02633	0.05297
3319.09775	0.044	3319.09775	0.05243
3317.16916	0.04341	3317.16916	0.05189
3315.24057	0.04285	3315.24057	0.05137
3313.31199	0.04231	3313.31199	0.05087
3311.3834	0.04183	3311.3834	0.05038
3309.45481	0.04135	3309.45481	0.04984
3307.52623	0.04081	3307.52623	0.04932
3305.59764	0.04035	3305.59764	0.04892
3303.66905	0.03994	3303.66905	0.04855
3301.74047	0.0395	3301.74047	0.04814
3299.81188	0.03908	3299.81188	0.04773
3297.88329	0.03869	3297.88329	0.0473
3295.95471	0.03832	3295.95471	0.04693
3294.02612	0.038	3294.02612	0.04662
3292.09753	0.03763	3292.09753	0.04627
3290.16895	0.03721	3290.16895	0.04591
3288.24036	0.03684	3288.24036	0.04561
3286.31177	0.03654	3286.31177	0.04533
3284.38319	0.03626	3284.38319	0.04505
3282.4546	0.03595	3282.4546	0.04475
3280.52601	0.03566	3280.52601	0.04447
3278.59743	0.03543	3278.59743	0.04424
3276.66884	0.03524	3276.66884	0.04401
3274.74025	0.03499	3274.74025	0.04374
3272.81167	0.0347	3272.81167	0.04345
3270.88308	0.03445	3270.88308	0.0432
3268.95449	0.03423	3268.95449	0.04296
3267.02591	0.03398	3267.02591	0.04272

3265.09732	0.03369	3265.09732	0.04245
3263.16873	0.03344	3263.16873	0.04219
3261.24015	0.03322	3261.24015	0.04194
3259.31156	0.03302	3259.31156	0.04169
3257.38297	0.03285	3257.38297	0.04147
3255.45439	0.03264	3255.45439	0.04123
3253.5258	0.03231	3253.5258	0.04095
3251.59721	0.03197	3251.59721	0.04069
3249.66863	0.03177	3249.66863	0.04047
3247.74004	0.03165	3247.74004	0.04029
3245.81145	0.03142	3245.81145	0.04001
3243.88287	0.03104	3243.88287	0.03969
3241.95428	0.03074	3241.95428	0.03947
3240.02569	0.03052	3240.02569	0.03926
3238.09711	0.03029	3238.09711	0.03899
3236.16852	0.03006	3236.16852	0.03871
3234.23993	0.02982	3234.23993	0.03843
3232.31135	0.02953	3232.31135	0.03814
3230.38276	0.02919	3230.38276	0.03785
3228.45417	0.02885	3228.45417	0.03753
3226.52558	0.02854	3226.52558	0.0372
3224.597	0.02827	3224.597	0.03693
3222.66841	0.02804	3222.66841	0.03671
3220.73982	0.02777	3220.73982	0.0364
3218.81124	0.02744	3218.81124	0.03606
3216.88265	0.02718	3216.88265	0.0358
3214.95406	0.02694	3214.95406	0.0355
3213.02548	0.02663	3213.02548	0.0352
3211.09689	0.02635	3211.09689	0.03493
3209.1683	0.02605	3209.1683	0.03461
3207.23972	0.02572	3207.23972	0.0343
3205.31113	0.02543	3205.31113	0.03401
3203.38254	0.02514	3203.38254	0.03369
3201.45396	0.02488	3201.45396	0.03339
3199.52537	0.02463	3199.52537	0.0331
3197.59678	0.0243	3197.59678	0.03275
3195.6682	0.02394	3195.6682	0.03243
3193.73961	0.02366	3193.73961	0.03214
3191.81102	0.02341	3191.81102	0.03184
3189.88244	0.02313	3189.88244	0.03152
3187.95385	0.02286	3187.95385	0.03123
3186.02526	0.02255	3186.02526	0.03091
3184.09668	0.02217	3184.09668	0.03055

3182.16809	0.02186	3182.16809	0.03027
3180.2395	0.02165	3180.2395	0.03003
3178.31092	0.02135	3178.31092	0.02967
3176.38233	0.02099	3176.38233	0.02932
3174.45374	0.02073	3174.45374	0.02906
3172.52516	0.02051	3172.52516	0.02877
3170.59657	0.02022	3170.59657	0.02844
3168.66798	0.01991	3168.66798	0.02812
3166.7394	0.01959	3166.7394	0.02781
3164.81081	0.01928	3164.81081	0.02752
3162.88222	0.01899	3162.88222	0.02723
3160.95364	0.01873	3160.95364	0.02694
3159.02505	0.01849	3159.02505	0.02666
3157.09646	0.01826	3157.09646	0.02638
3155.16788	0.01804	3155.16788	0.02608
3153.23929	0.01781	3153.23929	0.02579
3151.3107	0.01754	3151.3107	0.0255
3149.38212	0.01723	3149.38212	0.0252
3147.45353	0.01692	3147.45353	0.0249
3145.52494	0.01665	3145.52494	0.02462
3143.59636	0.01641	3143.59636	0.02433
3141.66777	0.01618	3141.66777	0.02404
3139.73918	0.01594	3139.73918	0.02376
3137.8106	0.01567	3137.8106	0.0235
3135.88201	0.01544	3135.88201	0.02328
3133.95342	0.01518	3133.95342	0.02298
3132.02484	0.01487	3132.02484	0.02266
3130.09625	0.01466	3130.09625	0.02243
3128.16766	0.01449	3128.16766	0.02222
3126.23908	0.01426	3126.23908	0.02195
3124.31049	0.01403	3124.31049	0.02167
3122.3819	0.01379	3122.3819	0.02137
3120.45332	0.01356	3120.45332	0.02112
3118.52473	0.01338	3118.52473	0.0209
3116.59614	0.01316	3116.59614	0.02064
3114.66756	0.01289	3114.66756	0.02038
3112.73897	0.01264	3112.73897	0.02015
3110.81038	0.01243	3110.81038	0.01989
3108.88179	0.0122	3108.88179	0.0196
3106.95321	0.01193	3106.95321	0.0193
3105.02462	0.0117	3105.02462	0.01906
3103.09603	0.0115	3103.09603	0.01883
3101.16745	0.01123	3101.16745	0.01851

3099.23886	0.01096	3099.23886	0.01826
3097.31027	0.01075	3097.31027	0.01804
3095.38169	0.01048	3095.38169	0.01775
3093.4531	0.01021	3093.4531	0.01749
3091.52451	0.01005	3091.52451	0.01728
3089.59593	0.00991	3089.59593	0.01707
3087.66734	0.00973	3087.66734	0.01682
3085.73875	0.00953	3085.73875	0.01655
3083.81017	0.00934	3083.81017	0.0163
3081.88158	0.00915	3081.88158	0.01607
3079.95299	0.00892	3079.95299	0.01583
3078.02441	0.00869	3078.02441	0.0156
3076.09582	0.00849	3076.09582	0.01542
3074.16723	0.00832	3074.16723	0.01527
3072.23865	0.00817	3072.23865	0.01509
3070.31006	0.00804	3070.31006	0.01492
3068.38147	0.00794	3068.38147	0.01474
3066.45289	0.00777	3066.45289	0.01449
3064.5243	0.00752	3064.5243	0.01424
3062.59571	0.0073	3062.59571	0.01408
3060.66713	0.00716	3060.66713	0.01397
3058.73854	0.00705	3058.73854	0.01382
3056.80995	0.00688	3056.80995	0.01357
3054.88137	0.00665	3054.88137	0.01331
3052.95278	0.00651	3052.95278	0.01314
3051.02419	0.00642	3051.02419	0.01301
3049.09561	0.00625	3049.09561	0.0128
3047.16702	0.00604	3047.16702	0.01255
3045.23843	0.00589	3045.23843	0.01235
3043.30985	0.00575	3043.30985	0.01215
3041.38126	0.00555	3041.38126	0.01192
3039.45267	0.00531	3039.45267	0.01169
3037.52409	0.0051	3037.52409	0.01149
3035.5955	0.00496	3035.5955	0.01131
3033.66691	0.00485	3033.66691	0.01114
3031.73833	0.00466	3031.73833	0.0109
3029.80974	0.00442	3029.80974	0.01066
3027.88115	0.00427	3027.88115	0.01049
3025.95257	0.00413	3025.95257	0.01028
3024.02398	0.0039	3024.02398	0.01002
3022.09539	0.00365	3022.09539	0.00977
3020.16681	0.00344	3020.16681	0.00954
3018.23822	0.00325	3018.23822	0.0093

3016.30963	0.00306	3016.30963	0.00903
3014.38105	0.00291	3014.38105	0.00878
3012.45246	0.00272	3012.45246	0.00853
3010.52387	0.00246	3010.52387	0.00826
3008.59529	0.00221	3008.59529	0.00805
3006.6667	0.00202	3006.6667	0.00787
3004.73811	0.00182	3004.73811	0.00764
3002.80953	0.0016	3002.80953	0.00739
3000.88094	0.00142	3000.88094	0.00716
2998.95235	0.00134	2998.95235	0.00701
2997.02377	0.00129	2997.02377	0.00686
2995.09518	0.00126	2995.09518	0.00673
2993.16659	0.00124	2993.16659	0.0066
2991.238	0.00126	2991.238	0.00653
2989.30942	0.0013	2989.30942	0.00654
2987.38083	0.00134	2987.38083	0.00657
2985.45224	0.00144	2985.45224	0.00663
2983.52366	0.00165	2983.52366	0.00675
2981.59507	0.00193	2981.59507	0.00692
2979.66648	0.00223	2979.66648	0.00714
2977.7379	0.0026	2977.7379	0.00743
2975.80931	0.00308	2975.80931	0.00781
2973.88072	0.00362	2973.88072	0.00826
2971.95214	0.00417	2971.95214	0.00877
2970.02355	0.00481	2970.02355	0.00935
2968.09496	0.00549	2968.09496	0.00996
2966.16638	0.00614	2966.16638	0.01054
2964.23779	0.00675	2964.23779	0.01105
2962.3092	0.00729	2962.3092	0.01146
2960.38062	0.00768	2960.38062	0.01176
2958.45203	0.00794	2958.45203	0.01193
2956.52344	0.00811	2956.52344	0.01201
2954.59486	0.00824	2954.59486	0.01204
2952.66627	0.00834	2952.66627	0.01206
2950.73768	0.00843	2950.73768	0.0121
2948.8091	0.00855	2948.8091	0.01217
2946.88051	0.00872	2946.88051	0.01227
2944.95192	0.00894	2944.95192	0.01243
2943.02334	0.00923	2943.02334	0.01269
2941.09475	0.00959	2941.09475	0.01304
2939.16616	0.01007	2939.16616	0.01347
2937.23758	0.01067	2937.23758	0.01399
2935.30899	0.01137	2935.30899	0.01458

2933.3804	0.01213	2933.3804	0.0152
2931.45182	0.01286	2931.45182	0.01582
2929.52323	0.01351	2929.52323	0.01642
2927.59464	0.014	2927.59464	0.01692
2925.66606	0.01433	2925.66606	0.01728
2923.73747	0.01448	2923.73747	0.01746
2921.80888	0.01445	2921.80888	0.01746
2919.8803	0.01427	2919.8803	0.01731
2917.95171	0.01395	2917.95171	0.01701
2916.02312	0.01349	2916.02312	0.01658
2914.09454	0.01294	2914.09454	0.01607
2912.16595	0.01235	2912.16595	0.01556
2910.23736	0.01177	2910.23736	0.01508
2908.30878	0.01122	2908.30878	0.01462
2906.38019	0.01074	2906.38019	0.01419
2904.4516	0.01032	2904.4516	0.01378
2902.52302	0.00993	2902.52302	0.01341
2900.59443	0.00956	2900.59443	0.01305
2898.66584	0.00922	2898.66584	0.0127
2896.73726	0.00889	2896.73726	0.01234
2894.80867	0.00854	2894.80867	0.01198
2892.88008	0.00817	2892.88008	0.01161
2890.9515	0.00781	2890.9515	0.01126
2889.02291	0.00749	2889.02291	0.01094
2887.09432	0.0072	2887.09432	0.01064
2885.16574	0.00695	2885.16574	0.01037
2883.23715	0.00678	2883.23715	0.01014
2881.30856	0.0067	2881.30856	0.00998
2879.37998	0.00671	2879.37998	0.0099
2877.45139	0.0068	2877.45139	0.00988
2875.5228	0.00694	2875.5228	0.00992
2873.59421	0.00711	2873.59421	0.00999
2871.66563	0.00725	2871.66563	0.01009
2869.73704	0.00734	2869.73704	0.01018
2867.80845	0.00745	2867.80845	0.01026
2865.87987	0.00758	2865.87987	0.01032
2863.95128	0.00771	2863.95128	0.01037
2862.02269	0.00786	2862.02269	0.01043
2860.09411	0.008	2860.09411	0.01052
2858.16552	0.00813	2858.16552	0.01061
2856.23693	0.00822	2856.23693	0.01066
2854.30835	0.00826	2854.30835	0.01066
2852.37976	0.00819	2852.37976	0.01055

2850.45117	0.00797	2850.45117	0.01031
2848.52259	0.00761	2848.52259	0.00994
2846.594	0.00713	2846.594	0.00946
2844.66541	0.00658	2844.66541	0.00892
2842.73683	0.00605	2842.73683	0.0084
2840.80824	0.00559	2840.80824	0.00792
2838.87965	0.00516	2838.87965	0.00743
2836.95107	0.00471	2836.95107	0.00694
2835.02248	0.00426	2835.02248	0.0065
2833.09389	0.00386	2833.09389	0.0061
2831.16531	0.00347	2831.16531	0.00574
2829.23672	0.00312	2829.23672	0.00539
2827.30813	0.00283	2827.30813	0.00507
2825.37955	0.00258	2825.37955	0.00478
2823.45096	0.00236	2823.45096	0.00451
2821.52237	0.00215	2821.52237	0.00425
2819.59379	0.00196	2819.59379	0.00401
2817.6652	0.00177	2817.6652	0.00378
2815.73661	0.00158	2815.73661	0.00356
2813.80803	0.00141	2813.80803	0.00336
2811.87944	0.00127	2811.87944	0.00318
2809.95085	0.00114	2809.95085	0.00301
2808.02227	0.001	2808.02227	0.00282
2806.09368	0.00086	2806.09368	0.00264
2804.16509	0.00076	2804.16509	0.00248
2802.23651	0.00069	2802.23651	0.00234
2800.30792	0.00065	2800.30792	0.00219
2798.37933	0.00062	2798.37933	0.00205
2796.45075	0.00057	2796.45075	0.00193
2794.52216	0.00049	2794.52216	0.00184
2792.59357	0.00041	2792.59357	0.00175
2790.66499	0.00035	2790.66499	0.00166
2788.7364	0.00028	2788.7364	0.00154
2786.80781	0.00021	2786.80781	0.00141
2784.87923	0.00017	2784.87923	0.00128
2782.95064	0.00015	2782.95064	0.00118
2781.02205	0.00014	2781.02205	0.00111
2779.09347	0.00012	2779.09347	0.00104
2777.16488	0.00012	2777.16488	0.00098
2775.23629	0.00011	2775.23629	0.0009
2773.30771	0.00008	2773.30771	0.00081
2771.37912	0.00002	2771.37912	0.00072
2769.45053	0	2769.45053	0.00064

2767.52195	0	2767.52195	0.00059
2765.59336	0.00001	2765.59336	0.00054
2763.66477	0	2763.66477	0.0005
2761.73619	-0.00001	2761.73619	0.00046
2759.8076	-0.00001	2759.8076	0.00041
2757.87901	-0.00001	2757.87901	0.00035
2755.95042	0	2755.95042	0.0003
2754.02184	0.00001	2754.02184	0.00027
2752.09325	0.00003	2752.09325	0.00026
2750.16466	0.00006	2750.16466	0.00027
2748.23608	0.00008	2748.23608	0.00026
2746.30749	0.00006	2746.30749	0.00022
2744.3789	0.00004	2744.3789	0.00017
2742.45032	0.00003	2742.45032	0.00014
2740.52173	0.00005	2740.52173	0.00013
2738.59314	0.0001	2738.59314	0.00014
2736.66456	0.00015	2736.66456	0.00014
2734.73597	0.00016	2734.73597	0.00015
2732.80738	0.00015	2732.80738	0.00014
2730.8788	0.00013	2730.8788	0.00011
2728.95021	0.00013	2728.95021	0.00009
2727.02162	0.00014	2727.02162	0.00008
2725.09304	0.00015	2725.09304	0.00009
2723.16445	0.00015	2723.16445	0.00009
2721.23586	0.00015	2721.23586	0.00009
2719.30728	0.00015	2719.30728	0.00008
2717.37869	0.00015	2717.37869	0.00006
2715.4501	0.00013	2715.4501	0.00003
2713.52152	0.00012	2713.52152	0
2711.59293	0.00009	2711.59293	-0.00001
2709.66434	0.00007	2709.66434	-0.00001
2707.73576	0.00005	2707.73576	-0.00002
2705.80717	0.00004	2705.80717	-0.00003
2703.87858	0.00004	2703.87858	-0.00004
2701.95	0.00006	2701.95	-0.00005
2700.02141	0.00007	2700.02141	-0.00005
2698.09282	0.00008	2698.09282	-0.00006
2696.16424	0.00006	2696.16424	-0.00007
2694.23565	0.00003	2694.23565	-0.00007
2692.30706	0	2692.30706	-0.00006
2690.37848	-0.00002	2690.37848	-0.00007
2688.44989	-0.00002	2688.44989	-0.00008
2686.5213	0	2686.5213	-0.00008

2684.59272	0.00003	2684.59272	-0.00007
2682.66413	0.00008	2682.66413	-0.00004
2680.73554	0.00014	2680.73554	-0.00001
2678.80696	0.00017	2678.80696	0
2676.87837	0.00017	2676.87837	0
2674.94978	0.00014	2674.94978	-0.00002
2673.0212	0.0001	2673.0212	-0.00004
2671.09261	0.00006	2671.09261	-0.00004
2669.16402	0.00003	2669.16402	-0.00003
2667.23544	0.00004	2667.23544	-0.00001
2665.30685	0.00005	2665.30685	0.00001
2663.37826	0.00006	2663.37826	0.00002
2661.44968	0.00007	2661.44968	0.00001
2659.52109	0.00008	2659.52109	0
2657.5925	0.00008	2657.5925	0
2655.66392	0.00007	2655.66392	-0.00001
2653.73533	0.00005	2653.73533	-0.00001
2651.80674	0.00002	2651.80674	-0.00001
2649.87816	-0.00002	2649.87816	-0.00002
2647.94957	-0.00005	2647.94957	-0.00003
2646.02098	-0.00004	2646.02098	-0.00005
2644.0924	-0.00001	2644.0924	-0.00005
2642.16381	0.00003	2642.16381	-0.00003
2640.23522	0.00006	2640.23522	-0.00001
2638.30663	0.00009	2638.30663	0
2636.37805	0.00009	2636.37805	-0.00001
2634.44946	0.00007	2634.44946	-0.00003
2632.52087	0.00004	2632.52087	-0.00006
2630.59229	0.00001	2630.59229	-0.00007
2628.6637	0	2628.6637	-0.00006
2626.73511	0.00002	2626.73511	-0.00004
2624.80653	0.00005	2624.80653	-0.00003
2622.87794	0.00008	2622.87794	-0.00004
2620.94935	0.00008	2620.94935	-0.00004
2619.02077	0.00008	2619.02077	-0.00005
2617.09218	0.00008	2617.09218	-0.00006
2615.16359	0.00008	2615.16359	-0.00005
2613.23501	0.00006	2613.23501	-0.00004
2611.30642	0.00004	2611.30642	-0.00003
2609.37783	0.00003	2609.37783	-0.00001
2607.44925	0.00004	2607.44925	0.00001
2605.52066	0.00005	2605.52066	0.00002
2603.59207	0.00006	2603.59207	0.00001

2601.66349	0.0001	2601.66349	0
2599.7349	0.00012	2599.7349	-0.00001
2597.80631	0.00011	2597.80631	0
2595.87773	0.00009	2595.87773	0
2593.94914	0.00005	2593.94914	0
2592.02055	0.00002	2592.02055	-0.00001
2590.09197	0	2590.09197	-0.00001
2588.16338	0.00002	2588.16338	0
2586.23479	0.00005	2586.23479	0.00001
2584.30621	0.00008	2584.30621	0.00001
2582.37762	0.0001	2582.37762	0
2580.44903	0.00008	2580.44903	-0.00001
2578.52045	0.00005	2578.52045	-0.00001
2576.59186	0.00003	2576.59186	-0.00001
2574.66327	0.00002	2574.66327	-0.00001
2572.73469	0.00002	2572.73469	-0.00002
2570.8061	0.00003	2570.8061	-0.00003
2568.87751	0.00003	2568.87751	-0.00002
2566.94893	0.00003	2566.94893	-0.00002
2565.02034	0.00003	2565.02034	-0.00001
2563.09175	0.00002	2563.09175	0
2561.16317	0.00002	2561.16317	0.00001
2559.23458	0.00003	2559.23458	0.00001
2557.30599	0.00007	2557.30599	0.00001
2555.37741	0.00009	2555.37741	0.00001
2553.44882	0.0001	2553.44882	0
2551.52023	0.00007	2551.52023	-0.00001
2549.59165	0.00004	2549.59165	0
2547.66306	0.00001	2547.66306	0.00001
2545.73447	0.00001	2545.73447	0.00001
2543.80589	0.00004	2543.80589	0.00001
2541.8773	0.00006	2541.8773	0.00001
2539.94871	0.00008	2539.94871	0.00002
2538.02013	0.00009	2538.02013	0.00001
2536.09154	0.00008	2536.09154	0.00001
2534.16295	0.00004	2534.16295	0.00001
2532.23437	0.00001	2532.23437	0.00001
2530.30578	-0.00001	2530.30578	0
2528.37719	-0.00002	2528.37719	-0.00002
2526.44861	-0.00001	2526.44861	-0.00003
2524.52002	0.00001	2524.52002	-0.00003
2522.59143	0.00003	2522.59143	-0.00002
2520.66284	0.00005	2520.66284	0

2518.73426	0.00006	2518.73426	0.00001
2516.80567	0.00005	2516.80567	0.00002
2514.87708	0.00002	2514.87708	0.00002
2512.9485	-0.00001	2512.9485	0
2511.01991	-0.00001	2511.01991	-0.00002
2509.09132	-0.00002	2509.09132	-0.00003
2507.16274	-0.00001	2507.16274	-0.00002
2505.23415	0.00001	2505.23415	0
2503.30556	0.00003	2503.30556	0
2501.37698	0.00004	2501.37698	0.00001
2499.44839	0.00004	2499.44839	0.00002
2497.5198	0.00003	2497.5198	0.00001
2495.59122	0.00001	2495.59122	0
2493.66263	0	2493.66263	0
2491.73404	0	2491.73404	0.00001
2489.80546	0.00003	2489.80546	0.00002
2487.87687	0.00007	2487.87687	0.00003
2485.94828	0.00009	2485.94828	0.00002
2484.0197	0.00008	2484.0197	0.00001
2482.09111	0.00004	2482.09111	0
2480.16252	0	2480.16252	0.00001
2478.23394	-0.00001	2478.23394	0.00002
2476.30535	0	2476.30535	0.00003
2474.37676	0.00004	2474.37676	0.00005
2472.44818	0.00009	2472.44818	0.00007
2470.51959	0.00012	2470.51959	0.00008
2468.591	0.00012	2468.591	0.00007
2466.66242	0.00008	2466.66242	0.00007
2464.73383	0.00003	2464.73383	0.00007
2462.80524	0	2462.80524	0.00007
2460.87666	0	2460.87666	0.00009
2458.94807	0.00005	2458.94807	0.00011
2457.01948	0.00011	2457.01948	0.00012
2455.0909	0.00016	2455.0909	0.00012
2453.16231	0.00018	2453.16231	0.00012
2451.23372	0.00017	2451.23372	0.00014
2449.30514	0.00015	2449.30514	0.00015
2447.37655	0.00013	2447.37655	0.00016
2445.44796	0.00012	2445.44796	0.00016
2443.51938	0.00013	2443.51938	0.00017
2441.59079	0.00016	2441.59079	0.00017
2439.6622	0.0002	2439.6622	0.00018
2437.73362	0.00024	2437.73362	0.00021

2435.80503	0.00027	2435.80503	0.00024
2433.87644	0.00029	2433.87644	0.00027
2431.94786	0.00031	2431.94786	0.00029
2430.01927	0.00032	2430.01927	0.00029
2428.09068	0.00034	2428.09068	0.0003
2426.1621	0.00034	2426.1621	0.00031
2424.23351	0.00034	2424.23351	0.00033
2422.30492	0.00035	2422.30492	0.00035
2420.37634	0.00037	2420.37634	0.00036
2418.44775	0.0004	2418.44775	0.00036
2416.51916	0.00043	2416.51916	0.00036
2414.59058	0.00046	2414.59058	0.00037
2412.66199	0.00047	2412.66199	0.00039
2410.7334	0.00047	2410.7334	0.0004
2408.80482	0.00048	2408.80482	0.00042
2406.87623	0.0005	2406.87623	0.00044
2404.94764	0.00053	2404.94764	0.00047
2403.01905	0.00057	2403.01905	0.0005
2401.09047	0.00061	2401.09047	0.00051
2399.16188	0.00063	2399.16188	0.00052
2397.23329	0.00065	2397.23329	0.00053
2395.30471	0.00068	2395.30471	0.00056
2393.37612	0.00073	2393.37612	0.00058
2391.44753	0.00081	2391.44753	0.00058
2389.51895	0.00092	2389.51895	0.00056
2387.59036	0.00108	2387.59036	0.00052
2385.66177	0.00124	2385.66177	0.00052
2383.73319	0.00135	2383.73319	0.00057
2381.8046	0.00139	2381.8046	0.00064
2379.87601	0.00137	2379.87601	0.0007
2377.94743	0.00134	2377.94743	0.00071
2376.01884	0.00126	2376.01884	0.0007
2374.09025	0.00115	2374.09025	0.00068
2372.16167	0.00115	2372.16167	0.00066
2370.23308	0.00113	2370.23308	0.00065
2368.30449	0.00099	2368.30449	0.00066
2366.37591	0.00087	2366.37591	0.00065
2364.44732	0.00083	2364.44732	0.00062
2362.51873	0.00095	2362.51873	0.00071
2360.59015	0.00115	2360.59015	0.00095
2358.66156	0.00093	2358.66156	0.0013
2356.73297	0.00033	2356.73297	0.00165
2354.80439	0	2354.80439	0.00163

2352.8758	0.00056	2352.8758	0.00076
2350.94721	0.0024	2350.94721	0
2349.01863	0.00424	2349.01863	0.00057
2347.09004	0.0039	2347.09004	0.00171
2345.16145	0.00157	2345.16145	0.00173
2343.23287	0.00004	2343.23287	0.00095
2341.30428	0.00042	2341.30428	0.00063
2339.37569	0.00127	2339.37569	0.00073
2337.44711	0.00165	2337.44711	0.00081
2335.51852	0.00165	2335.51852	0.00085
2333.58993	0.00158	2333.58993	0.00097
2331.66135	0.0014	2331.66135	0.001
2329.73276	0.0011	2329.73276	0.00083
2327.80417	0.00103	2327.80417	0.00071
2325.87559	0.00127	2325.87559	0.00082
2323.947	0.00133	2323.947	0.00096
2322.01841	0.00098	2322.01841	0.00089
2320.08983	0.00067	2320.08983	0.00066
2318.16124	0.0008	2318.16124	0.00051
2316.23265	0.00109	2316.23265	0.00057
2314.30407	0.00111	2314.30407	0.00069
2312.37548	0.00086	2312.37548	0.00071
2310.44689	0.00064	2310.44689	0.00066
2308.51831	0.0006	2308.51831	0.00061
2306.58972	0.00069	2306.58972	0.00056
2304.66113	0.00076	2304.66113	0.00052
2302.73255	0.00072	2302.73255	0.00052
2300.80396	0.0006	2300.80396	0.00052
2298.87537	0.00051	2298.87537	0.0005
2296.94679	0.00045	2296.94679	0.00047
2295.0182	0.00041	2295.0182	0.00043
2293.08961	0.00041	2293.08961	0.00039
2291.16103	0.00042	2291.16103	0.00035
2289.23244	0.0004	2289.23244	0.00031
2287.30385	0.00035	2287.30385	0.00027
2285.37526	0.0003	2285.37526	0.00024
2283.44668	0.00028	2283.44668	0.00024
2281.51809	0.00025	2281.51809	0.00024
2279.5895	0.0002	2279.5895	0.00021
2277.66092	0.00018	2277.66092	0.00016
2275.73233	0.00021	2275.73233	0.00013
2273.80374	0.00022	2273.80374	0.00011
2271.87516	0.0002	2271.87516	0.00011

2269.94657	0.00014	2269.94657	0.00013
2268.01798	0.00008	2268.01798	0.00014
2266.0894	0.00004	2266.0894	0.00013
2264.16081	0.00005	2264.16081	0.00011
2262.23222	0.0001	2262.23222	0.00008
2260.30364	0.00016	2260.30364	0.00006
2258.37505	0.0002	2258.37505	0.00005
2256.44646	0.00017	2256.44646	0.00006
2254.51788	0.0001	2254.51788	0.00007
2252.58929	0.00004	2252.58929	0.00007
2250.6607	0	2250.6607	0.00003
2248.73212	0.00001	2248.73212	0.00003
2246.80353	0.00005	2246.80353	0.00001
2244.87494	0.00009	2244.87494	0.00001
2242.94636	0.0001	2242.94636	0.00002
2241.01777	0.0001	2241.01777	0.00004
2239.08918	0.00008	2239.08918	0.00006
2237.1606	0.00005	2237.1606	0.00006
2235.23201	0.00002	2235.23201	0.00004
2233.30342	0	2233.30342	0.00002
2231.37484	0	2231.37484	0
2229.44625	0.00002	2229.44625	0
2227.51766	0.00005	2227.51766	0.00002
2225.58908	0.00008	2225.58908	0.00003
2223.66049	0.00009	2223.66049	0.00004
2221.7319	0.00008	2221.7319	0.00004
2219.80332	0.00006	2219.80332	0.00003
2217.87473	0.00004	2217.87473	0.00001
2215.94614	0.00001	2215.94614	0.00001
2214.01756	0	2214.01756	0.00002
2212.08897	0.00001	2212.08897	0.00004
2210.16038	0.00004	2210.16038	0.00005
2208.2318	0.00008	2208.2318	0.00004
2206.30321	0.00011	2206.30321	0.00003
2204.37462	0.00011	2204.37462	0.00001
2202.44604	0.00008	2202.44604	0.00001
2200.51745	0.00003	2200.51745	0
2198.58886	0	2198.58886	0
2196.66028	0	2196.66028	0.00002
2194.73169	0.00002	2194.73169	0.00004
2192.8031	0.00006	2192.8031	0.00004
2190.87452	0.00008	2190.87452	0.00003
2188.94593	0.00008	2188.94593	0.00001

2187.01734	0.00006	2187.01734	0
2185.08876	0.00002	2185.08876	0.00001
2183.16017	0	2183.16017	0.00002
2181.23158	0	2181.23158	0.00004
2179.303	0.00004	2179.303	0.00003
2177.37441	0.00007	2177.37441	0.00002
2175.44582	0.00007	2175.44582	-0.00001
2173.51724	0.00006	2173.51724	-0.00002
2171.58865	0.00003	2171.58865	-0.00001
2169.66006	0	2169.66006	0.00001
2167.73147	0	2167.73147	0.00003
2165.80289	0.00002	2165.80289	0.00005
2163.8743	0.00006	2163.8743	0.00006
2161.94571	0.00009	2161.94571	0.00004
2160.01713	0.0001	2160.01713	-0.00001
2158.08854	0.00008	2158.08854	-0.00002
2156.15995	0.00004	2156.15995	-0.00001
2154.23137	0.00001	2154.23137	0.00002
2152.30278	0	2152.30278	0.00004
2150.37419	0.00001	2150.37419	0.00005
2148.44561	0.00003	2148.44561	0.00007
2146.51702	0.00006	2146.51702	0.00007
2144.58843	0.00006	2144.58843	0.00005
2142.65985	0.00004	2142.65985	0.00002
2140.73126	0.00003	2140.73126	0.00004
2138.80267	0.00003	2138.80267	0.00009
2136.87409	0.00001	2136.87409	0.00012
2134.9455	0	2134.9455	0.00012
2133.01691	0	2133.01691	0.00011
2131.08833	0.00001	2131.08833	0.00011
2129.15974	0.00001	2129.15974	0.00011
2127.23115	0.00002	2127.23115	0.00013
2125.30257	0.00003	2125.30257	0.00016
2123.37398	0.00003	2123.37398	0.00017
2121.44539	0.00001	2121.44539	0.00015
2119.51681	0	2119.51681	0.00013
2117.58822	0.00001	2117.58822	0.00016
2115.65963	0.00002	2115.65963	0.0002
2113.73105	0.00001	2113.73105	0.00022
2111.80246	0.00002	2111.80246	0.00026
2109.87387	0.00004	2109.87387	0.00029
2107.94529	0.00003	2107.94529	0.00028
2106.0167	0.00001	2106.0167	0.00026

2104.08811	0	2104.08811	0.00024
2102.15953	0.00001	2102.15953	0.00025
2100.23094	0.00003	2100.23094	0.00028
2098.30235	0.00006	2098.30235	0.00033
2096.37377	0.00011	2096.37377	0.00036
2094.44518	0.00013	2094.44518	0.00037
2092.51659	0.00014	2092.51659	0.00036
2090.58801	0.00009	2090.58801	0.00031
2088.65942	0.00001	2088.65942	0.0003
2086.73083	0	2086.73083	0.00037
2084.80225	0.00004	2084.80225	0.00044
2082.87366	0.00012	2082.87366	0.0005
2080.94507	0.0002	2080.94507	0.00051
2079.01649	0.00026	2079.01649	0.0005
2077.0879	0.00026	2077.0879	0.00046
2075.15931	0.00021	2075.15931	0.00046
2073.23073	0.00013	2073.23073	0.00048
2071.30214	0.00006	2071.30214	0.00051
2069.37355	0.00007	2069.37355	0.0006
2067.44497	0.00015	2067.44497	0.00064
2065.51638	0.00017	2065.51638	0.00051
2063.58779	0.00016	2063.58779	0.00044
2061.65921	0.00024	2061.65921	0.00053
2059.73062	0.00027	2059.73062	0.00055
2057.80203	0.00021	2057.80203	0.00057
2055.87345	0.00017	2055.87345	0.00065
2053.94486	0.00018	2053.94486	0.00072
2052.01627	0.00021	2052.01627	0.00074
2050.08768	0.00024	2050.08768	0.0007
2048.1591	0.00026	2048.1591	0.00064
2046.23051	0.00026	2046.23051	0.00063
2044.30192	0.00028	2044.30192	0.00067
2042.37334	0.00026	2042.37334	0.00065
2040.44475	0.00021	2040.44475	0.00065
2038.51616	0.00025	2038.51616	0.00075
2036.58758	0.00033	2036.58758	0.00078
2034.65899	0.00035	2034.65899	0.0007
2032.7304	0.00032	2032.7304	0.00065
2030.80182	0.00029	2030.80182	0.00067
2028.87323	0.00028	2028.87323	0.00073
2026.94464	0.00028	2026.94464	0.00078
2025.01606	0.0003	2025.01606	0.0008
2023.08747	0.00029	2023.08747	0.00078

2021.15888	0.00033	2021.15888	0.00079
2019.2303	0.00038	2019.2303	0.00071
2017.30171	0.00025	2017.30171	0.00047
2015.37312	0.00015	2015.37312	0.0005
2013.44454	0.00026	2013.44454	0.00075
2011.51595	0.00037	2011.51595	0.00085
2009.58736	0.00036	2009.58736	0.00082
2007.65878	0.00031	2007.65878	0.00077
2005.73019	0.00029	2005.73019	0.00072
2003.8016	0.0003	2003.8016	0.0007
2001.87302	0.00036	2001.87302	0.00073
1999.94443	0.00041	1999.94443	0.00074
1998.01584	0.00038	1998.01584	0.00072
1996.08726	0.00044	1996.08726	0.00088
1994.15867	0.00053	1994.15867	0.00086
1992.23008	0.00029	1992.23008	0.00045
1990.3015	0	1990.3015	0.00034
1988.37291	0.00003	1988.37291	0.00054
1986.44432	0.00018	1986.44432	0.00067
1984.51574	0.00032	1984.51574	0.00077
1982.58715	0.00046	1982.58715	0.00082
1980.65856	0.00052	1980.65856	0.00082
1978.72998	0.00052	1978.72998	0.0008
1976.80139	0.00044	1976.80139	0.00077
1974.8728	0.00031	1974.8728	0.00072
1972.94422	0.00018	1972.94422	0.0007
1971.01563	0.0002	1971.01563	0.00083
1969.08704	0.0003	1969.08704	0.00078
1967.15846	0.0002	1967.15846	0.00045
1965.22987	0.00012	1965.22987	0.00047
1963.30128	0.00031	1963.30128	0.00076
1961.3727	0.00042	1961.3727	0.0008
1959.44411	0.00036	1959.44411	0.00078
1957.51552	0.00034	1957.51552	0.00083
1955.58694	0.00032	1955.58694	0.0008
1953.65835	0.00029	1953.65835	0.00078
1951.72976	0.0003	1951.72976	0.00081
1949.80118	0.0003	1949.80118	0.00079
1947.87259	0.00025	1947.87259	0.00076
1945.944	0.00032	1945.944	0.00083
1944.01542	0.00033	1944.01542	0.00061
1942.08683	0.00004	1942.08683	0.00024
1940.15824	0	1940.15824	0.00054

1938.22966	0.00036	1938.22966	0.00097
1936.30107	0.00053	1936.30107	0.00099
1934.37248	0.00047	1934.37248	0.00086
1932.44389	0.0004	1932.44389	0.00082
1930.51531	0.00035	1930.51531	0.00083
1928.58672	0.00031	1928.58672	0.00088
1926.65813	0.0004	1926.65813	0.00103
1924.72955	0.00041	1924.72955	0.00081
1922.80096	0.00012	1922.80096	0.0004
1920.87237	0.00018	1920.87237	0.00066
1918.94379	0.00041	1918.94379	0.00065
1917.0152	0.00019	1917.0152	0.00038
1915.08661	0.00017	1915.08661	0.00079
1913.15803	0.00057	1913.15803	0.00123
1911.22944	0.00069	1911.22944	0.00107
1909.30085	0.00044	1909.30085	0.0007
1907.37227	0.00026	1907.37227	0.0007
1905.44368	0.00037	1905.44368	0.00092
1903.51509	0.00051	1903.51509	0.001
1901.58651	0.00059	1901.58651	0.00099
1899.65792	0.00073	1899.65792	0.0011
1897.72933	0.00088	1897.72933	0.00114
1895.80075	0.00075	1895.80075	0.00091
1893.87216	0.0006	1893.87216	0.00096
1891.94357	0.00071	1891.94357	0.00114
1890.01499	0.00057	1890.01499	0.00076
1888.0864	0.00027	1888.0864	0.00061
1886.15781	0.00046	1886.15781	0.00101
1884.22923	0.00082	1884.22923	0.00122
1882.30064	0.00099	1882.30064	0.00122
1880.37205	0.00107	1880.37205	0.00122
1878.44347	0.00105	1878.44347	0.00118
1876.51488	0.0009	1876.51488	0.0011
1874.58629	0.00077	1874.58629	0.00118
1872.65771	0.00095	1872.65771	0.00144
1870.72912	0.00107	1870.72912	0.00108
1868.80053	0.00051	1868.80053	0.00007
1866.87195	0	1866.87195	0
1864.94336	0.00034	1864.94336	0.00089
1863.01477	0.00091	1863.01477	0.0014
1861.08619	0.00107	1861.08619	0.00129
1859.1576	0.00099	1859.1576	0.00111
1857.22901	0.00095	1857.22901	0.00108

1855.30043	0.00097	1855.30043	0.00114
1853.37184	0.00092	1853.37184	0.00118
1851.44325	0.00079	1851.44325	0.00117
1849.51467	0.00068	1849.51467	0.00117
1847.58608	0.00079	1847.58608	0.00125
1845.65749	0.00074	1845.65749	0.00072
1843.72891	0.00011	1843.72891	-0.00007
1841.80032	-0.00005	1841.80032	0.00042
1839.87173	0.0006	1839.87173	0.0013
1837.94315	0.00093	1837.94315	0.00123
1836.01456	0.00071	1836.01456	0.00092
1834.08597	0.00075	1834.08597	0.00117
1832.15739	0.00094	1832.15739	0.00107
1830.2288	0.00047	1830.2288	0.00014
1828.30021	0.00001	1828.30021	0.00002
1826.37163	0.00021	1826.37163	0.00049
1824.44304	0.00029	1824.44304	0.00047
1822.51445	0.00027	1822.51445	0.00068
1820.58587	0.00061	1820.58587	0.00104
1818.65728	0.00083	1818.65728	0.00098
1816.72869	0.00078	1816.72869	0.00084
1814.8001	0.00083	1814.8001	0.001
1812.87152	0.00088	1812.87152	0.00093
1810.94293	0.00057	1810.94293	0.0005
1809.01434	0.00019	1809.01434	0.00043
1807.08576	0.00021	1807.08576	0.00084
1805.15717	0.00056	1805.15717	0.00126
1803.22858	0.00086	1803.22858	0.00121
1801.3	0.0007	1801.3	0.00062
1799.37141	0.00031	1799.37141	0.00038
1797.44282	0.00041	1797.44282	0.001
1795.51424	0.00102	1795.51424	0.00162
1793.58565	0.00113	1793.58565	0.00101
1791.65706	0.00032	1791.65706	0.00009
1789.72848	0.00009	1789.72848	0.00063
1787.79989	0.00078	1787.79989	0.00173
1785.8713	0.00126	1785.8713	0.00178
1783.94272	0.00114	1783.94272	0.00144
1782.01413	0.00092	1782.01413	0.00111
1780.08554	0.00057	1780.08554	0.00079
1778.15696	0.00043	1778.15696	0.00109
1776.22837	0.00088	1776.22837	0.00167
1774.29978	0.00115	1774.29978	0.00119

1772.3712	0.00051	1772.3712	0
1770.44261	0	1770.44261	0.00012
1768.51402	0.00024	1768.51402	0.00109
1766.58544	0.00075	1766.58544	0.00191
1764.65685	0.00135	1764.65685	0.00235
1762.72826	0.00143	1762.72826	0.00174
1760.79968	0.00104	1760.79968	0.00135
1758.87109	0.00115	1758.87109	0.00177
1756.9425	0.00135	1756.9425	0.00196
1755.01392	0.00161	1755.01392	0.00259
1753.08533	0.00226	1753.08533	0.0031
1751.15674	0.00224	1751.15674	0.00226
1749.22816	0.0015	1749.22816	0.0015
1747.29957	0.00149	1747.29957	0.00222
1745.37098	0.00235	1745.37098	0.0035
1743.4424	0.00332	1743.4424	0.00436
1741.51381	0.00382	1741.51381	0.00457
1739.58522	0.00395	1739.58522	0.00491
1737.65664	0.00483	1737.65664	0.00644
1735.72805	0.00606	1735.72805	0.00673
1733.79946	0.00586	1733.79946	0.00543
1731.87088	0.00559	1731.87088	0.00626
1729.94229	0.00662	1729.94229	0.0082
1728.0137	0.00786	1728.0137	0.0096
1726.08512	0.00875	1726.08512	0.01045
1724.15653	0.00917	1724.15653	0.01084
1722.22794	0.00972	1722.22794	0.01172
1720.29936	0.01082	1720.29936	0.01236
1718.37077	0.01125	1718.37077	0.01142
1716.44218	0.01077	1716.44218	0.01103
1714.5136	0.0112	1714.5136	0.01289
1712.58501	0.01265	1712.58501	0.01531
1710.65642	0.01408	1710.65642	0.01689
1708.72784	0.01501	1708.72784	0.0174
1706.79925	0.01517	1706.79925	0.01706
1704.87066	0.01528	1704.87066	0.01766
1702.94208	0.01642	1702.94208	0.01918
1701.01349	0.01725	1701.01349	0.01906
1699.0849	0.01719	1699.0849	0.01912
1697.15631	0.01774	1697.15631	0.02009
1695.22773	0.01832	1695.22773	0.02086
1693.29914	0.01915	1693.29914	0.02273
1691.37055	0.02024	1691.37055	0.02399

1689.44197	0.02076	1689.44197	0.02435
1687.51338	0.02146	1687.51338	0.02497
1685.58479	0.02197	1685.58479	0.02411
1683.65621	0.02103	1683.65621	0.02292
1681.72762	0.02091	1681.72762	0.02492
1679.79903	0.02259	1679.79903	0.02774
1677.87045	0.02443	1677.87045	0.02903
1675.94186	0.02512	1675.94186	0.02878
1674.01327	0.02539	1674.01327	0.02939
1672.08469	0.02653	1672.08469	0.03089
1670.1561	0.02748	1670.1561	0.03113
1668.22751	0.02794	1668.22751	0.03217
1666.29893	0.02968	1666.29893	0.03509
1664.37034	0.03192	1664.37034	0.03678
1662.44175	0.03309	1662.44175	0.03717
1660.51317	0.03403	1660.51317	0.03887
1658.58458	0.03593	1658.58458	0.04177
1656.65599	0.03844	1656.65599	0.0437
1654.72741	0.03975	1654.72741	0.04249
1652.79882	0.039	1652.79882	0.04137
1650.87023	0.04009	1650.87023	0.04567
1648.94165	0.04338	1648.94165	0.04918
1647.01306	0.04525	1647.01306	0.04901
1645.08447	0.04608	1645.08447	0.05025
1643.15589	0.048	1643.15589	0.05338
1641.2273	0.05031	1641.2273	0.05578
1639.29871	0.05239	1639.29871	0.05708
1637.37013	0.0539	1637.37013	0.05693
1635.44154	0.05444	1635.44154	0.05678
1633.51295	0.05555	1633.51295	0.05959
1631.58437	0.05788	1631.58437	0.0629
1629.65578	0.05974	1629.65578	0.06405
1627.72719	0.06058	1627.72719	0.06436
1625.79861	0.06136	1625.79861	0.06505
1623.87002	0.06192	1623.87002	0.06522
1621.94143	0.06232	1621.94143	0.0661
1620.01285	0.06363	1620.01285	0.06787
1618.08426	0.06456	1618.08426	0.06754
1616.15567	0.06372	1616.15567	0.06618
1614.22709	0.06312	1614.22709	0.06687
1612.2985	0.06352	1612.2985	0.06779
1610.36991	0.06341	1610.36991	0.06717
1608.44133	0.06253	1608.44133	0.06604

1606.51274	0.06173	1606.51274	0.06541
1604.58415	0.06117	1604.58415	0.06494
1602.65557	0.06042	1602.65557	0.06418
1600.72698	0.05948	1600.72698	0.06344
1598.79839	0.05859	1598.79839	0.06282
1596.86981	0.05762	1596.86981	0.06194
1594.94122	0.05645	1594.94122	0.06082
1593.01263	0.0553	1593.01263	0.05987
1591.08405	0.0543	1591.08405	0.05904
1589.15546	0.05323	1589.15546	0.05806
1587.22687	0.05202	1587.22687	0.05699
1585.29829	0.0506	1585.29829	0.05578
1583.3697	0.04896	1583.3697	0.0545
1581.44111	0.04749	1581.44111	0.0536
1579.51252	0.04656	1579.51252	0.05273
1577.58394	0.04496	1577.58394	0.05005
1575.65535	0.04208	1575.65535	0.0471
1573.72676	0.04061	1573.72676	0.04727
1571.79818	0.04054	1571.79818	0.04723
1569.86959	0.03896	1569.86959	0.04457
1567.941	0.03643	1567.941	0.04262
1566.01242	0.03488	1566.01242	0.04217
1564.08383	0.03421	1564.08383	0.04222
1562.15524	0.03419	1562.15524	0.04169
1560.22666	0.03263	1560.22666	0.03735
1558.29807	0.02822	1558.29807	0.03237
1556.36948	0.02608	1556.36948	0.033
1554.4409	0.02645	1554.4409	0.03437
1552.51231	0.02643	1552.51231	0.0339
1550.58372	0.02539	1550.58372	0.03244
1548.65514	0.02414	1548.65514	0.03145
1546.72655	0.02332	1546.72655	0.03092
1544.79796	0.02238	1544.79796	0.02946
1542.86938	0.02088	1542.86938	0.02694
1540.94079	0.01853	1540.94079	0.02329
1539.0122	0.01608	1539.0122	0.02176
1537.08362	0.01638	1537.08362	0.02427
1535.15503	0.01753	1535.15503	0.02479
1533.22644	0.01667	1533.22644	0.02242
1531.29786	0.01549	1531.29786	0.0218
1529.36927	0.01554	1529.36927	0.02217
1527.44068	0.01529	1527.44068	0.02135
1525.5121	0.01462	1525.5121	0.02064

1523.58351	0.01445	1523.58351	0.02014
1521.65492	0.01413	1521.65492	0.01912
1519.72634	0.01414	1519.72634	0.0196
1517.79775	0.01482	1517.79775	0.02037
1515.86916	0.01521	1515.86916	0.02066
1513.94058	0.01566	1513.94058	0.02177
1512.01199	0.01653	1512.01199	0.02307
1510.0834	0.01742	1510.0834	0.02314
1508.15482	0.01732	1508.15482	0.02067
1506.22623	0.01605	1506.22623	0.01891
1504.29764	0.01643	1504.29764	0.0219
1502.36906	0.0185	1502.36906	0.02508
1500.44047	0.01997	1500.44047	0.02558
1498.51188	0.01996	1498.51188	0.02447
1496.5833	0.01924	1496.5833	0.02389
1494.65471	0.01944	1494.65471	0.02538
1492.72612	0.02065	1492.72612	0.02671
1490.79754	0.02101	1490.79754	0.02559
1488.86895	0.02008	1488.86895	0.02419
1486.94036	0.01973	1486.94036	0.02483
1485.01178	0.02042	1485.01178	0.02635
1483.08319	0.02105	1483.08319	0.02693
1481.1546	0.02112	1481.1546	0.02678
1479.22602	0.02121	1479.22602	0.02695
1477.29743	0.02163	1477.29743	0.02709
1475.36884	0.02197	1475.36884	0.02662
1473.44026	0.02186	1473.44026	0.02576
1471.51167	0.02187	1471.51167	0.02652
1469.58308	0.02325	1469.58308	0.02943
1467.6545	0.02529	1467.6545	0.03133
1465.72591	0.02622	1465.72591	0.03106
1463.79732	0.02632	1463.79732	0.03139
1461.86873	0.02734	1461.86873	0.03337
1459.94015	0.02883	1459.94015	0.03398
1458.01156	0.02878	1458.01156	0.0321
1456.08297	0.02781	1456.08297	0.0319
1454.15439	0.02851	1454.15439	0.03464
1452.2258	0.03018	1452.2258	0.0367
1450.29721	0.03122	1450.29721	0.0369
1448.36863	0.0313	1448.36863	0.03624
1446.44004	0.0311	1446.44004	0.03619
1444.51145	0.03122	1444.51145	0.03689
1442.58287	0.03141	1442.58287	0.03751

1440.65428	0.03164	1440.65428	0.03802
1438.72569	0.03168	1438.72569	0.03749
1436.79711	0.03076	1436.79711	0.03587
1434.86852	0.02982	1434.86852	0.03576
1432.93993	0.02999	1432.93993	0.03661
1431.01135	0.02998	1431.01135	0.0362
1429.08276	0.0292	1429.08276	0.03544
1427.15417	0.02858	1427.15417	0.03535
1425.22559	0.02807	1425.22559	0.03492
1423.297	0.02748	1423.297	0.03436
1421.36841	0.02712	1421.36841	0.03366
1419.43983	0.02621	1419.43983	0.03184
1417.51124	0.02483	1417.51124	0.03076
1415.58265	0.02456	1415.58265	0.0314
1413.65407	0.02495	1413.65407	0.03182
1411.72548	0.02495	1411.72548	0.03145
1409.79689	0.02478	1409.79689	0.03116
1407.86831	0.0248	1407.86831	0.03107
1405.93972	0.02462	1405.93972	0.03057
1404.01113	0.02423	1404.01113	0.0304
1402.08255	0.02421	1402.08255	0.03089
1400.15396	0.0242	1400.15396	0.03068
1398.22537	0.02379	1398.22537	0.02997
1396.29679	0.0234	1396.29679	0.02936
1394.3682	0.02311	1394.3682	0.02896
1392.43961	0.0233	1392.43961	0.02959
1390.51103	0.0241	1390.51103	0.03052
1388.58244	0.02477	1388.58244	0.03068
1386.65385	0.02507	1386.65385	0.03109
1384.72527	0.02556	1384.72527	0.0323
1382.79668	0.02598	1382.79668	0.03288
1380.86809	0.02588	1380.86809	0.03241
1378.93951	0.0256	1378.93951	0.03186
1377.01092	0.02543	1377.01092	0.03149
1375.08233	0.02497	1375.08233	0.03081
1373.15375	0.0243	1373.15375	0.03049
1371.22516	0.02407	1371.22516	0.03081
1369.29657	0.02402	1369.29657	0.03074
1367.36799	0.02386	1367.36799	0.03047
1365.4394	0.02383	1365.4394	0.0302
1363.51081	0.02356	1363.51081	0.02936
1361.58223	0.02298	1361.58223	0.02884
1359.65364	0.02286	1359.65364	0.0293

1357.72505	0.02311	1357.72505	0.02974
1355.79647	0.0232	1355.79647	0.02959
1353.86788	0.02313	1353.86788	0.02929
1351.93929	0.02317	1351.93929	0.0291
1350.0107	0.02321	1350.0107	0.02893
1348.08212	0.02313	1348.08212	0.02887
1346.15353	0.023	1346.15353	0.02904
1344.22494	0.02297	1344.22494	0.02936
1342.29636	0.023	1342.29636	0.02937
1340.36777	0.02285	1340.36777	0.02877
1338.43918	0.02267	1338.43918	0.02841
1336.5106	0.02295	1336.5106	0.02888
1334.58201	0.02353	1334.58201	0.0295
1332.65342	0.02393	1332.65342	0.02981
1330.72484	0.02412	1330.72484	0.02997
1328.79625	0.02429	1328.79625	0.03021
1326.86766	0.02454	1326.86766	0.03048
1324.93908	0.02485	1324.93908	0.0308
1323.01049	0.02523	1323.01049	0.03114
1321.0819	0.02559	1321.0819	0.03136
1319.15332	0.02586	1319.15332	0.03152
1317.22473	0.02621	1317.22473	0.03197
1315.29614	0.02672	1315.29614	0.03249
1313.36756	0.02721	1313.36756	0.03278
1311.43897	0.02765	1311.43897	0.03308
1309.51038	0.02811	1309.51038	0.03346
1307.5818	0.02847	1307.5818	0.03379
1305.65321	0.02871	1305.65321	0.03414
1303.72462	0.02892	1303.72462	0.03453
1301.79604	0.02918	1301.79604	0.03487
1299.86745	0.02955	1299.86745	0.03517
1297.93886	0.03	1297.93886	0.03541
1296.01028	0.03046	1296.01028	0.03563
1294.08169	0.03086	1294.08169	0.0359
1292.1531	0.03114	1292.1531	0.03623
1290.22452	0.03128	1290.22452	0.03654
1288.29593	0.0313	1288.29593	0.0368
1286.36734	0.03135	1286.36734	0.03709
1284.43876	0.03155	1284.43876	0.03742
1282.51017	0.03183	1282.51017	0.03768
1280.58158	0.03208	1280.58158	0.03788
1278.653	0.03227	1278.653	0.03805
1276.72441	0.03238	1276.72441	0.03821

1274.79582	0.0324	1274.79582	0.03831
1272.86724	0.03234	1272.86724	0.03827
1270.93865	0.03224	1270.93865	0.03815
1269.01006	0.03219	1269.01006	0.03812
1267.08148	0.03219	1267.08148	0.03816
1265.15289	0.03215	1265.15289	0.03821
1263.2243	0.03209	1263.2243	0.03825
1261.29572	0.03203	1261.29572	0.03825
1259.36713	0.032	1259.36713	0.03828
1257.43854	0.03206	1257.43854	0.03838
1255.50996	0.03218	1255.50996	0.03844
1253.58137	0.03222	1253.58137	0.03845
1251.65278	0.03216	1251.65278	0.03847
1249.7242	0.03204	1249.7242	0.03851
1247.79561	0.03189	1247.79561	0.03854
1245.86702	0.03175	1245.86702	0.03854
1243.93844	0.03169	1243.93844	0.03853
1242.00985	0.03175	1242.00985	0.03856
1240.08126	0.03189	1240.08126	0.03867
1238.15268	0.03207	1238.15268	0.03885
1236.22409	0.03222	1236.22409	0.03903
1234.2955	0.0323	1234.2955	0.03918
1232.36691	0.03234	1232.36691	0.03927
1230.43833	0.03237	1230.43833	0.03934
1228.50974	0.03244	1228.50974	0.03942
1226.58115	0.03254	1226.58115	0.03954
1224.65257	0.03263	1224.65257	0.03969
1222.72398	0.03269	1222.72398	0.03986
1220.79539	0.03273	1220.79539	0.04003
1218.86681	0.03277	1218.86681	0.04012
1216.93822	0.03284	1216.93822	0.04019
1215.00963	0.03299	1215.00963	0.04029
1213.08105	0.03312	1213.08105	0.04042
1211.15246	0.03316	1211.15246	0.04055
1209.22387	0.03313	1209.22387	0.0407
1207.29529	0.03305	1207.29529	0.04078
1205.3667	0.03292	1205.3667	0.04076
1203.43811	0.03285	1203.43811	0.04071
1201.50953	0.03287	1201.50953	0.0407
1199.58094	0.0329	1199.58094	0.04067
1197.65235	0.03301	1197.65235	0.04074
1195.72377	0.03305	1195.72377	0.04082
1193.79518	0.03299	1193.79518	0.04088

1191.86659	0.03288	1191.86659	0.04087
1189.93801	0.03278	1189.93801	0.04082
1188.00942	0.03277	1188.00942	0.04081
1186.08083	0.03287	1186.08083	0.04093
1184.15225	0.03308	1184.15225	0.04123
1182.22366	0.03331	1182.22366	0.04161
1180.29507	0.03338	1180.29507	0.04187
1178.36649	0.03328	1178.36649	0.04194
1176.4379	0.03315	1176.4379	0.04194
1174.50931	0.03312	1174.50931	0.04197
1172.58073	0.03318	1172.58073	0.04205
1170.65214	0.0333	1170.65214	0.0422
1168.72355	0.0334	1168.72355	0.04235
1166.79497	0.03334	1166.79497	0.04236
1164.86638	0.03305	1164.86638	0.04217
1162.93779	0.03256	1162.93779	0.04176
1161.00921	0.03194	1161.00921	0.04118
1159.08062	0.03136	1159.08062	0.04058
1157.15203	0.03094	1157.15203	0.04011
1155.22345	0.03066	1155.22345	0.03978
1153.29486	0.03046	1153.29486	0.03951
1151.36627	0.0302	1151.36627	0.03919
1149.43769	0.02982	1149.43769	0.03874
1147.5091	0.02937	1147.5091	0.03825
1145.58051	0.02897	1145.58051	0.03783
1143.65193	0.02865	1143.65193	0.03754
1141.72334	0.02842	1141.72334	0.03737
1139.79475	0.02825	1139.79475	0.03729
1137.86617	0.02811	1137.86617	0.03722
1135.93758	0.028	1135.93758	0.03716
1134.00899	0.02798	1134.00899	0.03715
1132.08041	0.02808	1132.08041	0.03728
1130.15182	0.02831	1130.15182	0.03756
1128.22323	0.02857	1128.22323	0.03787
1126.29465	0.02879	1126.29465	0.03814
1124.36606	0.02899	1124.36606	0.03838
1122.43747	0.02917	1122.43747	0.03859
1120.50889	0.02931	1120.50889	0.0388
1118.5803	0.02953	1118.5803	0.03908
1116.65171	0.02986	1116.65171	0.03945
1114.72312	0.03023	1114.72312	0.03983
1112.79454	0.03054	1112.79454	0.04008
1110.86595	0.03074	1110.86595	0.04015

1108.93736	0.03083	1108.93736	0.04003
1107.00878	0.03083	1107.00878	0.03983
1105.08019	0.03074	1105.08019	0.03963
1103.1516	0.03065	1103.1516	0.03952
1101.22302	0.0306	1101.22302	0.03952
1099.29443	0.03053	1099.29443	0.03948
1097.36584	0.0304	1097.36584	0.03927
1095.43726	0.03025	1095.43726	0.03891
1093.50867	0.03007	1093.50867	0.03849
1091.58008	0.02986	1091.58008	0.03812
1089.6515	0.02965	1089.6515	0.03788
1087.72291	0.02944	1087.72291	0.03774
1085.79432	0.02919	1085.79432	0.0376
1083.86574	0.02891	1083.86574	0.03741
1081.93715	0.02856	1081.93715	0.0371
1080.00856	0.02812	1080.00856	0.03663
1078.07998	0.02763	1078.07998	0.03611
1076.15139	0.02714	1076.15139	0.03565
1074.2228	0.02672	1074.2228	0.03533
1072.29422	0.02644	1072.29422	0.03515
1070.36563	0.02623	1070.36563	0.03503
1068.43704	0.02605	1068.43704	0.03487
1066.50846	0.0259	1066.50846	0.03467
1064.57987	0.02577	1064.57987	0.03451
1062.65128	0.02567	1062.65128	0.03444
1060.7227	0.0256	1060.7227	0.03447
1058.79411	0.0255	1058.79411	0.03453
1056.86552	0.0254	1056.86552	0.03457
1054.93694	0.0253	1054.93694	0.0345
1053.00835	0.02521	1053.00835	0.03428
1051.07976	0.02514	1051.07976	0.03399
1049.15118	0.02512	1049.15118	0.03376
1047.22259	0.02515	1047.22259	0.03362
1045.294	0.02519	1045.294	0.0336
1043.36542	0.0252	1043.36542	0.03366
1041.43683	0.02516	1041.43683	0.03373
1039.50824	0.02512	1039.50824	0.03375
1037.57966	0.02511	1037.57966	0.0337
1035.65107	0.02512	1035.65107	0.0336
1033.72248	0.0251	1033.72248	0.03344
1031.7939	0.02493	1031.7939	0.0331
1029.86531	0.02443	1029.86531	0.03243
1027.93672	0.0236	1027.93672	0.03142

1026.00814	0.02256	1026.00814	0.03019
1024.07955	0.02145	1024.07955	0.0289
1022.15096	0.02048	1022.15096	0.02782
1020.22238	0.01981	1020.22238	0.02707
1018.29379	0.01943	1018.29379	0.0266
1016.3652	0.01927	1016.3652	0.02629
1014.43662	0.0192	1014.43662	0.02599
1012.50803	0.01901	1012.50803	0.02556
1010.57944	0.01862	1010.57944	0.02493
1008.65086	0.01796	1008.65086	0.02413
1006.72227	0.0171	1006.72227	0.02321
1004.79368	0.01617	1004.79368	0.02225
1002.8651	0.01526	1002.8651	0.02123
1000.93651	0.01435	1000.93651	0.02013
999.00792	0.01343	999.00792	0.01898
997.07933	0.01247	997.07933	0.01779
995.15075	0.01146	995.15075	0.0166
993.22216	0.01051	993.22216	0.01552
991.29357	0.00967	991.29357	0.01459
989.36499	0.00897	989.36499	0.01378
987.4364	0.00839	987.4364	0.01302
985.50781	0.00783	985.50781	0.01218
983.57923	0.00719	983.57923	0.01123
981.65064	0.00645	981.65064	0.01025
979.72205	0.00567	979.72205	0.00933
977.79347	0.00491	977.79347	0.00851
975.86488	0.00428	975.86488	0.00777
973.93629	0.00377	973.93629	0.00706
972.00771	0.00335	972.00771	0.00634
970.07912	0.00299	970.07912	0.00565
968.15053	0.00266	968.15053	0.005
966.22195	0.00233	966.22195	0.00444
964.29336	0.002	964.29336	0.00401
962.36477	0.00166	962.36477	0.00366
960.43619	0.00129	960.43619	0.0033
958.5076	0.00095	958.5076	0.0029
956.57901	0.00067	956.57901	0.00245
954.65043	0.00047	954.65043	0.00201
952.72184	0.00039	952.72184	0.00167
950.79325	0.00039	950.79325	0.00144
948.86467	0.00041	948.86467	0.0013
946.93608	0.00043	946.93608	0.0012
945.00749	0.00037	945.00749	0.00108

943.07891	0.00018	943.07891	0.00091
941.15032	0.00003	941.15032	0.00074
939.22173	0	939.22173	0.00061
937.29315	0.00008	937.29315	0.00053
935.36456	0.00023	935.36456	0.00047
933.43597	0.00032	933.43597	0.0004
931.50739	0.00029	931.50739	0.00028
929.5788	0.00017	929.5788	0.00014
927.65021	0.00001	927.65021	0.00004
925.72163	-0.00006	925.72163	0.00002
923.79304	-0.00007	923.79304	0
921.86445	0.00013	921.86445	0.00004
919.93587	0.00047	919.93587	0.00016
918.00728	0.00077	918.00728	0.00033
916.07869	0.00083	916.07869	0.00047
914.15011	0.00066	914.15011	0.00052
912.22152	0.00039	912.22152	0.00046
910.29293	0.00011	910.29293	0.00028
908.36435	0	908.36435	0.00009
906.43576	0.00013	906.43576	-0.00006
904.50717	0.00035	904.50717	-0.00016
902.57859	0.0005	902.57859	-0.00018
900.65	0.00049	900.65	-0.00012
898.72141	0.00032	898.72141	-0.00004
896.79283	0.00015	896.79283	0.00005
894.86424	0.00016	894.86424	0.00018
892.93565	0.0004	892.93565	0.0003
891.00707	0.00085	891.00707	0.00042
889.07848	0.00136	889.07848	0.00057
887.14989	0.00177	887.14989	0.00077
885.22131	0.00204	885.22131	0.00103
883.29272	0.00219	883.29272	0.00132
881.36413	0.00226	881.36413	0.00155
879.43554	0.00239	879.43554	0.00175
877.50696	0.00265	877.50696	0.00198
875.57837	0.00297	875.57837	0.00223
873.64978	0.00332	873.64978	0.00249
871.7212	0.00359	871.7212	0.00271
869.79261	0.00371	869.79261	0.00286
867.86402	0.00377	867.86402	0.00291
865.93544	0.0038	865.93544	0.00289
864.00685	0.00376	864.00685	0.00283
862.07826	0.00368	862.07826	0.00279

860.14968	0.00357	860.14968	0.00278
858.22109	0.00344	858.22109	0.00278
856.2925	0.00335	856.2925	0.0027
854.36392	0.00326	854.36392	0.00247
852.43533	0.00319	852.43533	0.00214
850.50674	0.00317	850.50674	0.00192
848.57816	0.00306	848.57816	0.00187
846.64957	0.00278	846.64957	0.00186
844.72098	0.00249	844.72098	0.00191
842.7924	0.00231	842.7924	0.00198
840.86381	0.00232	840.86381	0.002
838.93522	0.00256	838.93522	0.00195
837.00664	0.00285	837.00664	0.00188
835.07805	0.00306	835.07805	0.00188
833.14946	0.00314	833.14946	0.00203
831.22088	0.00306	831.22088	0.00232
829.29229	0.00291	829.29229	0.00264
827.3637	0.00292	827.3637	0.00288
825.43512	0.00318	825.43512	0.00299
823.50653	0.00363	823.50653	0.00305
821.57794	0.00416	821.57794	0.0032
819.64936	0.00453	819.64936	0.00348
817.72077	0.0046	817.72077	0.00386
815.79218	0.0045	815.79218	0.00426
813.8636	0.00435	813.8636	0.00453
811.93501	0.00433	811.93501	0.0046
810.00642	0.00456	810.00642	0.00458
808.07784	0.00496	808.07784	0.00459
806.14925	0.0054	806.14925	0.00483
804.22066	0.00579	804.22066	0.00535
802.29208	0.00601	802.29208	0.00598
800.36349	0.00603	800.36349	0.00643
798.4349	0.00593	798.4349	0.00652
796.50632	0.00578	796.50632	0.00622
794.57773	0.00566	794.57773	0.00573
792.64914	0.00557	792.64914	0.00533
790.72056	0.0054	790.72056	0.00515
788.79197	0.00512	788.79197	0.00514
786.86338	0.00486	786.86338	0.00519
784.9348	0.00467	784.9348	0.00513
783.00621	0.00464	783.00621	0.00496
781.07762	0.00477	781.07762	0.00481
779.14904	0.00489	779.14904	0.0047

777.22045	0.00484	777.22045	0.00461
775.29186	0.00456	775.29186	0.00451
773.36328	0.00409	773.36328	0.00431
771.43469	0.00359	771.43469	0.00393
769.5061	0.00322	769.5061	0.00342
767.57752	0.00298	767.57752	0.00289
765.64893	0.00285	765.64893	0.00253
763.72034	0.00275	763.72034	0.00244
761.79175	0.00261	761.79175	0.00254
759.86317	0.00246	759.86317	0.00267
757.93458	0.00234	757.93458	0.00263
756.00599	0.00222	756.00599	0.00236
754.07741	0.00214	754.07741	0.00199
752.14882	0.00211	752.14882	0.00172
750.22023	0.00201	750.22023	0.00161
748.29165	0.00184	748.29165	0.00165
746.36306	0.00165	746.36306	0.00173
744.43447	0.00143	744.43447	0.00166
742.50589	0.00128	742.50589	0.00137
740.5773	0.00122	740.5773	0.00096
738.64871	0.00114	738.64871	0.0006
736.72013	0.00099	736.72013	0.00047
734.79154	0.00077	734.79154	0.00056
732.86295	0.00054	732.86295	0.00069
730.93437	0.00044	730.93437	0.0007
729.00578	0.00049	729.00578	0.00054
727.07719	0.00064	727.07719	0.00024
725.14861	0.00086	725.14861	0
723.22002	0.001	723.22002	0
721.29143	0.00065	721.29143	0.00011
719.36285	0.00022	719.36285	0.00024
717.43426	0.00002	717.43426	0.00032
715.50567	0	715.50567	0.00026
713.57709	0.00015	713.57709	0.00004
711.6485	0.00058	711.6485	-0.00019
709.71991	0.0011	709.71991	-0.00023
707.79133	0.00144	707.79133	-0.00004
705.86274	0.00144	705.86274	0.00028
703.93415	0.00118	703.93415	0.00056
702.00557	0.00094	702.00557	0.00072
700.07698	0.00095	700.07698	0.00077
698.14839	0.00127	698.14839	0.0008
696.21981	0.00181	696.21981	0.00088

694.29122	0.00235	694.29122	0.00104
692.36263	0.00264	692.36263	0.00118
690.43405	0.00266	690.43405	0.00123
688.50546	0.00243	688.50546	0.00112
686.57687	0.00211	686.57687	0.00089
684.64829	0.00192	684.64829	0.00066
682.7197	0.0019	682.7197	0.00054
680.79111	0.002	680.79111	0.00062
678.86253	0.00216	678.86253	0.00083
676.93394	0.00247	676.93394	0.00098
675.00535	0.00338	675.00535	0.00098
673.07677	0.00461	673.07677	0.00113
671.14818	0.00383	671.14818	0.00157
669.21959	0.00065	669.21959	0.00153
667.29101	0	667.29101	0.00087
665.36242	0.003	665.36242	0.00095
663.43383	0.00462	663.43383	0.00158
661.50525	0.0038	661.50525	0.00183
659.57666	0.00296	659.57666	0.00169
657.64807	0.00296	657.64807	0.0015
655.71949	0.00322	655.71949	0.00146
653.7909	0.00338	653.7909	0.00163
651.86231	0.00339	651.86231	0.00195
649.93373	0.00338	649.93373	0.00228
648.00514	0.00351	648.00514	0.00253
646.07655	0.00378	646.07655	0.00268
644.14796	0.00408	644.14796	0.00277
642.21938	0.0043	642.21938	0.00288
640.29079	0.00447	640.29079	0.0031
638.3622	0.00452	638.3622	0.00339
636.43362	0.00443	636.43362	0.00362
634.50503	0.00434	634.50503	0.00369
632.57644	0.00444	632.57644	0.00369
630.64786	0.00479	630.64786	0.00376
628.71927	0.00526	628.71927	0.00395
626.79068	0.00562	626.79068	0.0041
624.8621	0.00574	624.8621	0.00439
622.93351	0.00566	622.93351	0.00484
621.00492	0.00551	621.00492	0.00521
619.07634	0.00531	619.07634	0.00532
617.14775	0.00537	617.14775	0.00526
615.21916	0.00592	615.21916	0.00538
613.29058	0.00663	613.29058	0.00564

611.36199	0.00701	611.36199	0.00578
609.4334	0.00698	609.4334	0.00586
607.50482	0.0067	607.50482	0.00594
605.57623	0.00638	605.57623	0.00598
603.64764	0.00616	603.64764	0.00597
601.71906	0.00607	601.71906	0.00582
599.79047	0.00615	599.79047	0.00569
597.86188	0.00638	597.86188	0.00573
595.9333	0.00658	595.9333	0.00582
594.00471	0.00662	594.00471	0.00583
592.07612	0.00652	592.07612	0.00583
590.14754	0.0065	590.14754	0.00608
588.21895	0.0067	588.21895	0.00638
586.29036	0.00694	586.29036	0.00647
584.36178	0.00709	584.36178	0.00656
582.43319	0.0072	582.43319	0.00672
580.5046	0.00727	580.5046	0.00697
578.57602	0.0073	578.57602	0.00729
576.64743	0.0074	576.64743	0.00747
574.71884	0.00762	574.71884	0.00773
572.79026	0.0079	572.79026	0.00799
570.86167	0.00809	570.86167	0.00793
568.93308	0.00813	568.93308	0.00782
567.0045	0.00816	567.0045	0.00799
565.07591	0.00832	565.07591	0.00842
563.14732	0.00853	563.14732	0.00884
561.21874	0.00871	561.21874	0.00913
559.29015	0.00885	559.29015	0.00926
557.36156	0.00891	557.36156	0.00922
555.43298	0.00885	555.43298	0.00914
553.50439	0.00881	553.50439	0.0092
551.5758	0.00886	551.5758	0.00937
549.64722	0.00903	549.64722	0.00958
547.71863	0.00933	547.71863	0.00985
545.79004	0.00968	545.79004	0.01012
543.86146	0.00996	543.86146	0.0104
541.93287	0.0101	541.93287	0.01063
540.00428	0.01008	540.00428	0.01079
538.0757	0.01003	538.0757	0.01097
536.14711	0.01014	536.14711	0.01116
534.21852	0.01034	534.21852	0.01135
532.28994	0.01046	532.28994	0.01136
530.36135	0.01037	530.36135	0.01112

528.43276	0.00993	528.43276	0.01046
526.50417	0.00919	526.50417	0.00964
524.57559	0.00865	524.57559	0.00956
522.647	0.00856	522.647	0.00993
520.71841	0.0086	520.71841	0.00989
518.78983	0.00856	518.78983	0.00944
516.86124	0.0085	516.86124	0.00918
514.93265	0.0085	514.93265	0.00922
513.00407	0.00835	513.00407	0.00915
511.07548	0.0079	511.07548	0.00892
509.14689	0.00742	509.14689	0.00873
507.21831	0.00725	507.21831	0.00871
505.28972	0.00737	505.28972	0.00876
503.36113	0.0074	503.36113	0.0083
501.43255	0.00727	501.43255	0.00774
499.50396	0.00716	499.50396	0.00766
497.57537	0.00703	497.57537	0.00782
495.64679	0.00682	495.64679	0.0081
493.7182	0.00667	493.7182	0.00832
491.78961	0.00679	491.78961	0.00849
489.86103	0.00727	489.86103	0.00878
487.93244	0.00789	487.93244	0.00898
486.00385	0.00842	486.00385	0.0091
484.07527	0.00897	484.07527	0.00978
482.14668	0.00979	482.14668	0.01133
480.21809	0.01092	480.21809	0.01302
478.28951	0.01204	478.28951	0.01419
476.36092	0.01278	476.36092	0.01471
474.43233	0.01299	474.43233	0.01449
472.50375	0.01292	472.50375	0.01421
470.57516	0.01294	470.57516	0.01463
468.64657	0.01295	468.64657	0.01507
466.71799	0.01255	466.71799	0.0152
464.7894	0.01185	464.7894	0.015
462.86081	0.01122	462.86081	0.01415
460.93223	0.01065	460.93223	0.01264
459.00364	0.00963	459.00364	0.01062
457.07505	0.00829	457.07505	0.00938
455.14647	0.00742	455.14647	0.00934
453.21788	0.00706	453.21788	0.00918
451.28929	0.00685	451.28929	0.00877
449.36071	0.00658	449.36071	0.00819
447.43212	0.00602	447.43212	0.00723

445.50353	0.00525	445.50353	0.00621
443.57495	0.00456	443.57495	0.00556
441.64636	0.00419	441.64636	0.00579
439.71777	0.00428	439.71777	0.00656
437.78919	0.00455	437.78919	0.00665
435.8606	0.00471	435.8606	0.00612
433.93201	0.00473	433.93201	0.00573
432.00343	0.00455	432.00343	0.00564
430.07484	0.00417	430.07484	0.00597
428.14625	0.00385	428.14625	0.0065
426.21767	0.0037	426.21767	0.00672
424.28908	0.00366	424.28908	0.00671
422.36049	0.00349	422.36049	0.00614
420.43191	0.00265	420.43191	0.004
418.50332	0.00153	418.50332	0.00237
416.57473	0.00121	416.57473	0.00302
414.64615	0.00164	414.64615	0.0039
412.71756	0.00159	412.71756	0.00371
410.78897	0.00117	410.78897	0.00308
408.86038	0.00092	408.86038	0.00218
406.9318	0.00077	406.9318	0.0011
405.00321	0.00056	405.00321	0.00053
403.07462	0.0004	403.07462	0.00012
401.14604	0	401.14604	0
399.21745	0	399.21745	0

Infrared spectra of Coking coal

Wavenumbers cm-1	Absorbance % Raw coal	Wavenumbers cm-1	Absorbance % Soaked coal
3997.96027	0.00025	3997.96027	0.0001
3996.03169	0	3996.03169	-0.00002
3994.1031	-0.00003	3994.1031	-0.00003
3992.17451	0.00005	3992.17451	0.00004
3990.24593	0.00005	3990.24593	0.00004
3988.31734	0.00002	3988.31734	0
3986.38875	0.00004	3986.38875	0.00009
3984.46017	0.00004	3984.46017	0.00022
3982.53158	-0.00001	3982.53158	0.00025
3980.60299	0	3980.60299	0.00025
3978.67441	0.00005	3978.67441	0.00036
3976.74582	0.00006	3976.74582	0.0005
3974.81723	0.00002	3974.81723	0.00046
3972.88865	0.00007	3972.88865	0.00038
3970.96006	0.00012	3970.96006	0.00041
3969.03147	0.0001	3969.03147	0.00041
3967.10289	0.00012	3967.10289	0.00047
3965.1743	0.00027	3965.1743	0.00075
3963.24571	0.0003	3963.24571	0.00099
3961.31713	0.00022	3961.31713	0.0009
3959.38854	0.00025	3959.38854	0.00078
3957.45995	0.00023	3957.45995	0.00076
3955.53137	0.00012	3955.53137	0.00074
3953.60278	0.00018	3953.60278	0.00094
3951.67419	0.00033	3951.67419	0.00132
3949.74561	0.00018	3949.74561	0.00122
3947.81702	0	3947.81702	0.00073
3945.88843	0.00043	3945.88843	0.00097
3943.95985	0.00069	3943.95985	0.00148
3942.03126	0.00036	3942.03126	0.00111
3940.10267	0.00019	3940.10267	0.00067
3938.17409	0.00023	3938.17409	0.00088
3936.2455	0.00046	3936.2455	0.00157
3934.31691	0.00073	3934.31691	0.00227
3932.38832	0.00043	3932.38832	0.00196
3930.45974	-0.00007	3930.45974	0.00093

3928.53115	0.00023	3928.53115	0.00094
3926.60256	0.00064	3926.60256	0.00176
3924.67398	0.00024	3924.67398	0.00164
3922.74539	0.00011	3922.74539	0.00123
3920.8168	0.00074	3920.8168	0.0019
3918.88822	0.00113	3918.88822	0.00257
3916.95963	0.00085	3916.95963	0.00203
3915.03104	0.00069	3915.03104	0.00135
3913.10246	0.00077	3913.10246	0.0015
3911.17387	0.00049	3911.17387	0.00177
3909.24528	0.0003	3909.24528	0.00215
3907.3167	0.00053	3907.3167	0.0032
3905.38811	0.00047	3905.38811	0.00378
3903.45952	0.00049	3903.45952	0.00304
3901.53094	0.00092	3901.53094	0.00208
3899.60235	0.00061	3899.60235	0.00101
3897.67376	0.00024	3897.67376	0.00032
3895.74518	0.00108	3895.74518	0.00162
3893.81659	0.00171	3893.81659	0.00356
3891.888	0.00055	3891.888	0.00298
3889.95942	0.00013	3889.95942	0.00169
3888.03083	0.00124	3888.03083	0.0027
3886.10224	0.00111	3886.10224	0.00332
3884.17366	0.00078	3884.17366	0.00262
3882.24507	0.00131	3882.24507	0.00287
3880.31648	0.00071	3880.31648	0.00227
3878.3879	0.00049	3878.3879	0.00144
3876.45931	0.00111	3876.45931	0.00261
3874.53072	0.00109	3874.53072	0.00379
3872.60214	0.00114	3872.60214	0.00404
3870.67355	0.00069	3870.67355	0.00322
3868.74496	0.00039	3868.74496	0.0018
3866.81638	0.00152	3866.81638	0.00253
3864.88779	0.00181	3864.88779	0.00366
3862.9592	0.00072	3862.9592	0.00247
3861.03062	0	3861.03062	0.00118
3859.10203	0.00049	3859.10203	0.00216
3857.17344	0.00208	3857.17344	0.00556
3855.24486	0.00298	3855.24486	0.00832
3853.31627	0.00145	3853.31627	0.00552
3851.38768	0.00028	3851.38768	0.00061
3849.4591	0.00075	3849.4591	0.00007
3847.53051	0.00143	3847.53051	0.00231

3845.60192	0.00124	3845.60192	0.00399
3843.67334	0.0006	3843.67334	0.00423
3841.74475	0.00072	3841.74475	0.00424
3839.81616	0.00096	3839.81616	0.00417
3837.88758	0.00049	3837.88758	0.00281
3835.95899	0.00064	3835.95899	0.00166
3834.0304	0.00137	3834.0304	0.00213
3832.10182	0.00139	3832.10182	0.00264
3830.17323	0.00124	3830.17323	0.00273
3828.24464	0.0009	3828.24464	0.00278
3826.31606	0.00049	3826.31606	0.00276
3824.38747	0.00127	3824.38747	0.00396
3822.45888	0.00125	3822.45888	0.0048
3820.5303	0.00021	3820.5303	0.00288
3818.60171	0.00133	3818.60171	0.00231
3816.67312	0.00198	3816.67312	0.00307
3814.74453	0.00095	3814.74453	0.00185
3812.81595	0.00083	3812.81595	0.00136
3810.88736	0.00175	3810.88736	0.00326
3808.95877	0.00171	3808.95877	0.00472
3807.03019	0.00034	3807.03019	0.00355
3805.1016	0.00077	3805.1016	0.00288
3803.17301	0.00178	3803.17301	0.00401
3801.24443	0.00081	3801.24443	0.00303
3799.31584	0.00087	3799.31584	0.00167
3797.38725	0.00168	3797.38725	0.00221
3795.45867	0.00159	3795.45867	0.00239
3793.53008	0.00156	3793.53008	0.00241
3791.60149	0.00166	3791.60149	0.00301
3789.67291	0.00161	3789.67291	0.00352
3787.74432	0.00156	3787.74432	0.0038
3785.81573	0.00114	3785.81573	0.0035
3783.88715	0.00102	3783.88715	0.00307
3781.95856	0.0017	3781.95856	0.00363
3780.02997	0.00166	3780.02997	0.00372
3778.10139	0.00099	3778.10139	0.00244
3776.1728	0.00103	3776.1728	0.00194
3774.24421	0.00168	3774.24421	0.00308
3772.31563	0.00202	3772.31563	0.0044
3770.38704	0.00149	3770.38704	0.00418
3768.45845	0.00126	3768.45845	0.00308
3766.52987	0.00129	3766.52987	0.00246
3764.60128	0.00118	3764.60128	0.00223

3762.67269	0.00181	3762.67269	0.0031
3760.74411	0.00195	3760.74411	0.00404
3758.81552	0.00075	3758.81552	0.00281
3756.88693	0.00032	3756.88693	0.00157
3754.95835	0.00157	3754.95835	0.00324
3753.02976	0.00215	3753.02976	0.00534
3751.10117	0.00038	3751.10117	0.00347
3749.17259	0	3749.17259	0.00065
3747.244	0.00263	3747.244	0.00247
3745.31541	0.00314	3745.31541	0.00423
3743.38683	0.00086	3743.38683	0.00151
3741.45824	0.00025	3741.45824	0
3739.52965	0.00138	3739.52965	0.00236
3737.60107	0.00197	3737.60107	0.00489
3735.67248	0.00132	3735.67248	0.00431
3733.74389	0.00056	3733.74389	0.00165
3731.81531	0.0005	3731.81531	0.00018
3729.88672	0.00148	3729.88672	0.00125
3727.95813	0.00216	3727.95813	0.00324
3726.02955	0.00182	3726.02955	0.00387
3724.10096	0.00146	3724.10096	0.00332
3722.17237	0.00143	3722.17237	0.0028
3720.24379	0.00118	3720.24379	0.00226
3718.3152	0.00085	3718.3152	0.00195
3716.38661	0.00131	3716.38661	0.00294
3714.45803	0.0023	3714.45803	0.00521
3712.52944	0.00222	3712.52944	0.00603
3710.60085	0.00117	3710.60085	0.00393
3708.67227	0.00116	3708.67227	0.00232
3706.74368	0.00227	3706.74368	0.00344
3704.81509	0.0035	3704.81509	0.00607
3702.88651	0.00365	3702.88651	0.00763
3700.95792	0.00329	3700.95792	0.00739
3699.02933	0.00367	3699.02933	0.00746
3697.10074	0.00401	3697.10074	0.00836
3695.17216	0.00424	3695.17216	0.00956
3693.24357	0.00502	3693.24357	0.01134
3691.31498	0.00555	3691.31498	0.01261
3689.3864	0.00482	3689.3864	0.01112
3687.45781	0.00442	3687.45781	0.00863
3685.52922	0.0051	3685.52922	0.0083
3683.60064	0.00541	3683.60064	0.00933
3681.67205	0.00495	3681.67205	0.00996

3679.74346	0.00492	3679.74346	0.01068
3677.81488	0.00503	3677.81488	0.01141
3675.88629	0.00342	3675.88629	0.00927
3673.9577	0.00348	3673.9577	0.00673
3672.02912	0.00585	3672.02912	0.00838
3670.10053	0.00586	3670.10053	0.00941
3668.17194	0.00494	3668.17194	0.00777
3666.24336	0.00549	3666.24336	0.00775
3664.31477	0.00593	3664.31477	0.00904
3662.38618	0.00554	3662.38618	0.00958
3660.4576	0.00556	3660.4576	0.01
3658.52901	0.00575	3658.52901	0.01063
3656.60042	0.00486	3656.60042	0.0098
3654.67184	0.00487	3654.67184	0.00914
3652.74325	0.00662	3652.74325	0.01161
3650.81466	0.00738	3650.81466	0.01396
3648.88608	0.00648	3648.88608	0.01184
3646.95749	0.00634	3646.95749	0.0088
3645.0289	0.00768	3645.0289	0.0091
3643.10032	0.00878	3643.10032	0.01118
3641.17173	0.00886	3641.17173	0.0124
3639.24314	0.00869	3639.24314	0.01267
3637.31456	0.00875	3637.31456	0.01285
3635.38597	0.0087	3635.38597	0.013
3633.45738	0.00929	3633.45738	0.01385
3631.5288	0.01098	3631.5288	0.01616
3629.60021	0.01126	3629.60021	0.01687
3627.67162	0.01046	3627.67162	0.01444
3625.74304	0.01152	3625.74304	0.01417
3623.81445	0.01365	3623.81445	0.01753
3621.88586	0.01515	3621.88586	0.02135
3619.95728	0.01515	3619.95728	0.02252
3618.02869	0.01493	3618.02869	0.0212
3616.1001	0.0158	3616.1001	0.02065
3614.17152	0.01634	3614.17152	0.02098
3612.24293	0.01672	3612.24293	0.02121
3610.31434	0.01772	3610.31434	0.02191
3608.38576	0.01803	3608.38576	0.02206
3606.45717	0.01808	3606.45717	0.02155
3604.52858	0.01902	3604.52858	0.02236
3602.6	0.01991	3602.6	0.02397
3600.67141	0.02015	3600.67141	0.02471
3598.74282	0.02079	3598.74282	0.02525

3596.81424	0.02185	3596.81424	0.02626
3594.88565	0.02225	3594.88565	0.02663
3592.95706	0.02276	3592.95706	0.02687
3591.02848	0.02448	3591.02848	0.02887
3589.09989	0.02573	3589.09989	0.03101
3587.1713	0.02549	3587.1713	0.03055
3585.24272	0.02593	3585.24272	0.02972
3583.31413	0.02751	3583.31413	0.03106
3581.38554	0.02877	3581.38554	0.03309
3579.45695	0.02949	3579.45695	0.03439
3577.52837	0.03015	3577.52837	0.0352
3575.59978	0.03067	3575.59978	0.03575
3573.67119	0.03106	3573.67119	0.03624
3571.74261	0.03199	3571.74261	0.03757
3569.81402	0.03329	3569.81402	0.03972
3567.88543	0.03359	3567.88543	0.04039
3565.95685	0.0335	3565.95685	0.03911
3564.02826	0.03464	3564.02826	0.03908
3562.09967	0.03616	3562.09967	0.04081
3560.17109	0.03693	3560.17109	0.04229
3558.2425	0.03746	3558.2425	0.04319
3556.31391	0.03832	3556.31391	0.04431
3554.38533	0.03897	3554.38533	0.04535
3552.45674	0.03914	3552.45674	0.04564
3550.52815	0.03975	3550.52815	0.04608
3548.59957	0.0409	3548.59957	0.04739
3546.67098	0.04147	3546.67098	0.04825
3544.74239	0.04165	3544.74239	0.04817
3542.81381	0.0424	3542.81381	0.04855
3540.88522	0.04361	3540.88522	0.04987
3538.95663	0.04452	3538.95663	0.05128
3537.02805	0.04487	3537.02805	0.05198
3535.09946	0.04523	3535.09946	0.0523
3533.17087	0.04603	3533.17087	0.05307
3531.24229	0.04686	3531.24229	0.05416
3529.3137	0.04734	3529.3137	0.05485
3527.38511	0.04776	3527.38511	0.05519
3525.45653	0.04835	3525.45653	0.05565
3523.52794	0.04892	3523.52794	0.05617
3521.59935	0.04963	3521.59935	0.0568
3519.67077	0.05046	3519.67077	0.0577
3517.74218	0.05106	3517.74218	0.05852
3515.81359	0.05147	3515.81359	0.05909

3513.88501	0.052	3513.88501	0.05977
3511.95642	0.05259	3511.95642	0.06056
3510.02783	0.05294	3510.02783	0.06098
3508.09925	0.05343	3508.09925	0.06127
3506.17066	0.0542	3506.17066	0.06209
3504.24207	0.05455	3504.24207	0.06279
3502.31349	0.05465	3502.31349	0.0628
3500.3849	0.0553	3500.3849	0.0631
3498.45631	0.05611	3498.45631	0.06405
3496.52773	0.05654	3496.52773	0.06483
3494.59914	0.0569	3494.59914	0.0653
3492.67055	0.05752	3492.67055	0.06591
3490.74197	0.05817	3490.74197	0.06667
3488.81338	0.0585	3488.81338	0.06715
3486.88479	0.05883	3486.88479	0.06749
3484.95621	0.0595	3484.95621	0.0682
3483.02762	0.06001	3483.02762	0.06893
3481.09903	0.06019	3481.09903	0.06913
3479.17045	0.06065	3479.17045	0.06944
3477.24186	0.06138	3477.24186	0.07026
3475.31327	0.0619	3475.31327	0.071
3473.38469	0.06224	3473.38469	0.07141
3471.4561	0.06255	3471.4561	0.07177
3469.52751	0.06277	3469.52751	0.07212
3467.59893	0.063	3467.59893	0.07247
3465.67034	0.06347	3465.67034	0.07297
3463.74175	0.06404	3463.74175	0.07359
3461.81316	0.06438	3461.81316	0.07398
3459.88458	0.06474	3459.88458	0.0743
3457.95599	0.06522	3457.95599	0.07479
3456.0274	0.0656	3456.0274	0.07527
3454.09882	0.06578	3454.09882	0.07556
3452.17023	0.06597	3452.17023	0.07581
3450.24164	0.06635	3450.24164	0.07628
3448.31306	0.0664	3448.31306	0.07652
3446.38447	0.06624	3446.38447	0.07628
3444.45588	0.06655	3444.45588	0.07634
3442.5273	0.06683	3442.5273	0.07666
3440.59871	0.06682	3440.59871	0.07672
3438.67012	0.06669	3438.67012	0.07662
3436.74154	0.06649	3436.74154	0.07649
3434.81295	0.06638	3434.81295	0.07642
3432.88436	0.06636	3432.88436	0.07642

3430.95578	0.06626	3430.95578	0.0763
3429.02719	0.06612	3429.02719	0.07609
3427.0986	0.066	3427.0986	0.07593
3425.17002	0.06594	3425.17002	0.0759
3423.24143	0.06586	3423.24143	0.07587
3421.31284	0.06548	3421.31284	0.07548
3419.38426	0.06506	3419.38426	0.07485
3417.45567	0.0648	3417.45567	0.07447
3415.52708	0.06444	3415.52708	0.07417
3413.5985	0.06391	3413.5985	0.07369
3411.66991	0.06343	3411.66991	0.07316
3409.74132	0.06301	3409.74132	0.07266
3407.81274	0.06262	3407.81274	0.07218
3405.88415	0.06221	3405.88415	0.07167
3403.95556	0.06158	3403.95556	0.07096
3402.02698	0.06079	3402.02698	0.07012
3400.09839	0.0602	3400.09839	0.06952
3398.1698	0.05975	3398.1698	0.06902
3396.24122	0.05925	3396.24122	0.06839
3394.31263	0.05867	3394.31263	0.06768
3392.38404	0.05792	3392.38404	0.0668
3390.45546	0.05729	3390.45546	0.06596
3388.52687	0.05688	3388.52687	0.06545
3386.59828	0.05637	3386.59828	0.06491
3384.6697	0.05563	3384.6697	0.06408
3382.74111	0.05484	3382.74111	0.06313
3380.81252	0.05404	3380.81252	0.06221
3378.88394	0.05321	3378.88394	0.06129
3376.95535	0.05237	3376.95535	0.06034
3375.02676	0.05152	3375.02676	0.05938
3373.09818	0.05058	3373.09818	0.05832
3371.16959	0.04954	3371.16959	0.05717
3369.241	0.04851	3369.241	0.05604
3367.31242	0.04756	3367.31242	0.05497
3365.38383	0.04675	3365.38383	0.05395
3363.45524	0.04594	3363.45524	0.05294
3361.52666	0.04499	3361.52666	0.05189
3359.59807	0.04409	3359.59807	0.05088
3357.66948	0.04331	3357.66948	0.04995
3355.7409	0.04245	3355.7409	0.04896
3353.81231	0.04146	3353.81231	0.04786
3351.88372	0.04045	3351.88372	0.04678
3349.95514	0.03954	3349.95514	0.04582

3348.02655	0.03876	3348.02655	0.04495
3346.09796	0.03798	3346.09796	0.04404
3344.16937	0.03713	3344.16937	0.04307
3342.24079	0.03628	3342.24079	0.04209
3340.3122	0.03546	3340.3122	0.04115
3338.38361	0.0347	3338.38361	0.0403
3336.45503	0.03398	3336.45503	0.03948
3334.52644	0.0333	3334.52644	0.03867
3332.59785	0.03265	3332.59785	0.03792
3330.66927	0.03198	3330.66927	0.03719
3328.74068	0.03124	3328.74068	0.03641
3326.81209	0.03049	3326.81209	0.03557
3324.88351	0.0298	3324.88351	0.03477
3322.95492	0.02922	3322.95492	0.03408
3321.02633	0.02869	3321.02633	0.03345
3319.09775	0.02811	3319.09775	0.03281
3317.16916	0.02752	3317.16916	0.03216
3315.24057	0.02696	3315.24057	0.03154
3313.31199	0.02641	3313.31199	0.03091
3311.3834	0.02585	3311.3834	0.03025
3309.45481	0.02521	3309.45481	0.02953
3307.52623	0.02458	3307.52623	0.02882
3305.59764	0.02412	3305.59764	0.02833
3303.66905	0.0238	3303.66905	0.028
3301.74047	0.0235	3301.74047	0.02769
3299.81188	0.02317	3299.81188	0.02732
3297.88329	0.02274	3297.88329	0.02685
3295.95471	0.02229	3295.95471	0.02636
3294.02612	0.02192	3294.02612	0.02595
3292.09753	0.02159	3292.09753	0.02556
3290.16895	0.02133	3290.16895	0.0252
3288.24036	0.02113	3288.24036	0.02494
3286.31177	0.02091	3286.31177	0.02471
3284.38319	0.02064	3284.38319	0.02443
3282.4546	0.02034	3282.4546	0.02409
3280.52601	0.02008	3280.52601	0.02376
3278.59743	0.01984	3278.59743	0.02348
3276.66884	0.01955	3276.66884	0.02317
3274.74025	0.0192	3274.74025	0.02278
3272.81167	0.01887	3272.81167	0.02237
3270.88308	0.01864	3270.88308	0.0221
3268.95449	0.01851	3268.95449	0.02196
3267.02591	0.01841	3267.02591	0.02183

3265.09732	0.01825	3265.09732	0.0216
3263.16873	0.01805	3263.16873	0.02133
3261.24015	0.01782	3261.24015	0.02106
3259.31156	0.01755	3259.31156	0.02078
3257.38297	0.01729	3257.38297	0.0205
3255.45439	0.01704	3255.45439	0.02021
3253.5258	0.01682	3253.5258	0.01989
3251.59721	0.01661	3251.59721	0.01959
3249.66863	0.01635	3249.66863	0.01934
3247.74004	0.01607	3247.74004	0.01909
3245.81145	0.01576	3245.81145	0.01878
3243.88287	0.01549	3243.88287	0.01839
3241.95428	0.0153	3241.95428	0.01811
3240.02569	0.01509	3240.02569	0.01788
3238.09711	0.01479	3238.09711	0.01755
3236.16852	0.01444	3236.16852	0.01714
3234.23993	0.01409	3234.23993	0.01675
3232.31135	0.01382	3232.31135	0.01644
3230.38276	0.01361	3230.38276	0.01619
3228.45417	0.01338	3228.45417	0.0159
3226.52558	0.01312	3226.52558	0.01557
3224.597	0.01288	3224.597	0.01526
3222.66841	0.01267	3222.66841	0.01501
3220.73982	0.01237	3220.73982	0.01467
3218.81124	0.01204	3218.81124	0.01426
3216.88265	0.01178	3216.88265	0.01395
3214.95406	0.01148	3214.95406	0.01366
3213.02548	0.01119	3213.02548	0.01335
3211.09689	0.01099	3211.09689	0.01309
3209.1683	0.01072	3209.1683	0.0128
3207.23972	0.01044	3207.23972	0.01245
3205.31113	0.01019	3205.31113	0.01215
3203.38254	0.00993	3203.38254	0.01185
3201.45396	0.00972	3201.45396	0.01159
3199.52537	0.0095	3199.52537	0.01135
3197.59678	0.00919	3197.59678	0.01099
3195.6682	0.00889	3195.6682	0.01058
3193.73961	0.00864	3193.73961	0.01028
3191.81102	0.00839	3191.81102	0.01003
3189.88244	0.00815	3189.88244	0.00976
3187.95385	0.00796	3187.95385	0.00951
3186.02526	0.00777	3186.02526	0.00925
3184.09668	0.00751	3184.09668	0.00893

3182.16809	0.00729	3182.16809	0.00868
3180.2395	0.0071	3180.2395	0.0085
3178.31092	0.00682	3178.31092	0.0082
3176.38233	0.00656	3176.38233	0.00785
3174.45374	0.00641	3174.45374	0.00762
3172.52516	0.00625	3172.52516	0.00745
3170.59657	0.00606	3170.59657	0.00723
3168.66798	0.00586	3168.66798	0.00697
3166.7394	0.00566	3166.7394	0.00673
3164.81081	0.00546	3164.81081	0.0065
3162.88222	0.00526	3162.88222	0.00629
3160.95364	0.00507	3160.95364	0.00607
3159.02505	0.0049	3159.02505	0.00584
3157.09646	0.00472	3157.09646	0.0056
3155.16788	0.00449	3155.16788	0.00534
3153.23929	0.00425	3153.23929	0.00509
3151.3107	0.00408	3151.3107	0.00491
3149.38212	0.00398	3149.38212	0.00479
3147.45353	0.0039	3147.45353	0.00468
3145.52494	0.0038	3145.52494	0.00453
3143.59636	0.00367	3143.59636	0.00433
3141.66777	0.00348	3141.66777	0.00408
3139.73918	0.00328	3139.73918	0.00383
3137.8106	0.00316	3137.8106	0.00367
3135.88201	0.00311	3135.88201	0.00364
3133.95342	0.00298	3133.95342	0.00354
3132.02484	0.00279	3132.02484	0.00328
3130.09625	0.00271	3130.09625	0.00311
3128.16766	0.00267	3128.16766	0.00306
3126.23908	0.00259	3126.23908	0.00295
3124.31049	0.00243	3124.31049	0.00279
3122.3819	0.00219	3122.3819	0.00254
3120.45332	0.00198	3120.45332	0.00229
3118.52473	0.00185	3118.52473	0.00214
3116.59614	0.00173	3116.59614	0.002
3114.66756	0.00169	3114.66756	0.00188
3112.73897	0.0017	3112.73897	0.00183
3110.81038	0.00165	3110.81038	0.00176
3108.88179	0.00153	3108.88179	0.00164
3106.95321	0.00145	3106.95321	0.00155
3105.02462	0.00144	3105.02462	0.00154
3103.09603	0.00138	3103.09603	0.00147
3101.16745	0.00119	3101.16745	0.00126

3099.23886	0.00109	3099.23886	0.00111
3097.31027	0.0011	3097.31027	0.00112
3095.38169	0.00103	3095.38169	0.00106
3093.4531	0.00092	3093.4531	0.00092
3091.52451	0.00081	3091.52451	0.00085
3089.59593	0.00072	3089.59593	0.00082
3087.66734	0.00063	3087.66734	0.00076
3085.73875	0.00053	3085.73875	0.00069
3083.81017	0.00047	3083.81017	0.00066
3081.88158	0.00045	3081.88158	0.00067
3079.95299	0.00042	3079.95299	0.00066
3078.02441	0.00043	3078.02441	0.00068
3076.09582	0.00053	3076.09582	0.0008
3074.16723	0.00064	3074.16723	0.00096
3072.23865	0.00067	3072.23865	0.00105
3070.31006	0.00065	3070.31006	0.00111
3068.38147	0.00063	3068.38147	0.00118
3066.45289	0.00063	3066.45289	0.00123
3064.5243	0.00069	3064.5243	0.00129
3062.59571	0.00082	3062.59571	0.00143
3060.66713	0.00095	3060.66713	0.00164
3058.73854	0.00099	3058.73854	0.00181
3056.80995	0.00092	3056.80995	0.00186
3054.88137	0.00089	3054.88137	0.00188
3052.95278	0.00097	3052.95278	0.00202
3051.02419	0.00108	3051.02419	0.00219
3049.09561	0.00113	3049.09561	0.00225
3047.16702	0.0011	3047.16702	0.00222
3045.23843	0.00105	3045.23843	0.00219
3043.30985	0.00099	3043.30985	0.00219
3041.38126	0.00096	3041.38126	0.00219
3039.45267	0.00099	3039.45267	0.0022
3037.52409	0.00102	3037.52409	0.00217
3035.5955	0.00099	3035.5955	0.00208
3033.66691	0.00094	3033.66691	0.00202
3031.73833	0.00089	3031.73833	0.00197
3029.80974	0.0009	3029.80974	0.00194
3027.88115	0.00097	3027.88115	0.002
3025.95257	0.00099	3025.95257	0.00202
3024.02398	0.00096	3024.02398	0.00195
3022.09539	0.00094	3022.09539	0.00188
3020.16681	0.00089	3020.16681	0.0018
3018.23822	0.00077	3018.23822	0.00169

3016.30963	0.00062	3016.30963	0.00157
3014.38105	0.00054	3014.38105	0.00144
3012.45246	0.0005	3012.45246	0.00132
3010.52387	0.0005	3010.52387	0.00121
3008.59529	0.00058	3008.59529	0.00117
3006.6667	0.00064	3006.6667	0.00114
3004.73811	0.00058	3004.73811	0.00101
3002.80953	0.00045	3002.80953	0.00081
3000.88094	0.00029	3000.88094	0.0006
2998.95235	0.00016	2998.95235	0.00042
2997.02377	0.00006	2997.02377	0.00027
2995.09518	0.00002	2995.09518	0.00015
2993.16659	0	2993.16659	0.00003
2991.238	0.00003	2991.238	0
2989.30942	0.00013	2989.30942	0.00003
2987.38083	0.00017	2987.38083	0.00005
2985.45224	0.00014	2985.45224	0.00008
2983.52366	0.00011	2983.52366	0.00014
2981.59507	0.00011	2981.59507	0.00024
2979.66648	0.0002	2979.66648	0.00042
2977.7379	0.00043	2977.7379	0.00073
2975.80931	0.00075	2975.80931	0.00117
2973.88072	0.00111	2973.88072	0.00171
2971.95214	0.00152	2971.95214	0.00236
2970.02355	0.00197	2970.02355	0.00311
2968.09496	0.00241	2968.09496	0.00391
2966.16638	0.00283	2966.16638	0.00466
2964.23779	0.00323	2964.23779	0.00533
2962.3092	0.00363	2962.3092	0.00592
2960.38062	0.00398	2960.38062	0.0064
2958.45203	0.00422	2958.45203	0.00673
2956.52344	0.00432	2956.52344	0.00694
2954.59486	0.00429	2954.59486	0.00705
2952.66627	0.00423	2952.66627	0.00715
2950.73768	0.00425	2950.73768	0.00726
2948.8091	0.00429	2948.8091	0.00732
2946.88051	0.00432	2946.88051	0.00735
2944.95192	0.00441	2944.95192	0.00746
2943.02334	0.00458	2943.02334	0.0077
2941.09475	0.0048	2941.09475	0.00805
2939.16616	0.00503	2939.16616	0.00844
2937.23758	0.00528	2937.23758	0.00887
2935.30899	0.00561	2935.30899	0.00938

2933.3804	0.00598	2933.3804	0.00994
2931.45182	0.00634	2931.45182	0.01049
2929.52323	0.00672	2929.52323	0.01107
2927.59464	0.00708	2927.59464	0.01167
2925.66606	0.00736	2925.66606	0.01218
2923.73747	0.00753	2923.73747	0.01252
2921.80888	0.00757	2921.80888	0.01268
2919.8803	0.0075	2919.8803	0.01264
2917.95171	0.00733	2917.95171	0.01244
2916.02312	0.00711	2916.02312	0.01209
2914.09454	0.00686	2914.09454	0.01169
2912.16595	0.0066	2912.16595	0.01128
2910.23736	0.00633	2910.23736	0.01089
2908.30878	0.00604	2908.30878	0.0105
2906.38019	0.00577	2906.38019	0.01012
2904.4516	0.00555	2904.4516	0.00975
2902.52302	0.00539	2902.52302	0.00941
2900.59443	0.00526	2900.59443	0.00907
2898.66584	0.00508	2898.66584	0.00873
2896.73726	0.00484	2896.73726	0.0084
2894.80867	0.00463	2894.80867	0.00811
2892.88008	0.00446	2892.88008	0.00786
2890.9515	0.00431	2890.9515	0.00761
2889.02291	0.00419	2889.02291	0.00736
2887.09432	0.00409	2887.09432	0.00712
2885.16574	0.00401	2885.16574	0.00689
2883.23715	0.00393	2883.23715	0.0067
2881.30856	0.00385	2881.30856	0.00656
2879.37998	0.00376	2879.37998	0.00649
2877.45139	0.00369	2877.45139	0.00646
2875.5228	0.00362	2875.5228	0.00646
2873.59421	0.00364	2873.59421	0.00652
2871.66563	0.00379	2871.66563	0.00668
2869.73704	0.004	2869.73704	0.00689
2867.80845	0.00416	2867.80845	0.00708
2865.87987	0.00425	2865.87987	0.00724
2863.95128	0.00429	2863.95128	0.00736
2862.02269	0.0043	2862.02269	0.00744
2860.09411	0.00432	2860.09411	0.0075
2858.16552	0.00439	2858.16552	0.00756
2856.23693	0.00448	2856.23693	0.00758
2854.30835	0.00452	2854.30835	0.00751
2852.37976	0.00446	2852.37976	0.00733

2850.45117	0.00425	2850.45117	0.00702
2848.52259	0.00393	2848.52259	0.00659
2846.594	0.00359	2846.594	0.00612
2844.66541	0.00328	2844.66541	0.00569
2842.73683	0.003	2842.73683	0.00528
2840.80824	0.00272	2840.80824	0.00486
2838.87965	0.00246	2838.87965	0.00447
2836.95107	0.00225	2836.95107	0.00412
2835.02248	0.00207	2835.02248	0.00379
2833.09389	0.00189	2833.09389	0.00348
2831.16531	0.00172	2831.16531	0.00319
2829.23672	0.00155	2829.23672	0.00292
2827.30813	0.00141	2827.30813	0.00265
2825.37955	0.00129	2825.37955	0.00239
2823.45096	0.00117	2823.45096	0.00213
2821.52237	0.00104	2821.52237	0.00189
2819.59379	0.00092	2819.59379	0.00169
2817.6652	0.00079	2817.6652	0.00151
2815.73661	0.00069	2815.73661	0.00135
2813.80803	0.00061	2813.80803	0.0012
2811.87944	0.00052	2811.87944	0.00105
2809.95085	0.00044	2809.95085	0.00093
2808.02227	0.00039	2808.02227	0.00084
2806.09368	0.00037	2806.09368	0.00077
2804.16509	0.00034	2804.16509	0.0007
2802.23651	0.00028	2802.23651	0.00061
2800.30792	0.00022	2800.30792	0.00052
2798.37933	0.00017	2798.37933	0.00043
2796.45075	0.00016	2796.45075	0.00039
2794.52216	0.00015	2794.52216	0.00035
2792.59357	0.00011	2792.59357	0.00031
2790.66499	0.00006	2790.66499	0.00026
2788.7364	0.00005	2788.7364	0.00022
2786.80781	0.0001	2786.80781	0.00021
2784.87923	0.00013	2784.87923	0.00022
2782.95064	0.00013	2782.95064	0.00022
2781.02205	0.0001	2781.02205	0.00021
2779.09347	0.00007	2779.09347	0.00018
2777.16488	0.00002	2777.16488	0.00012
2775.23629	0	2775.23629	0.00006
2773.30771	0.00002	2773.30771	0.00001
2771.37912	0.00006	2771.37912	0
2769.45053	0.00008	2769.45053	0

2767.52195	0.00006	2767.52195	0
2765.59336	0.00003	2765.59336	-0.00001
2763.66477	0.00002	2763.66477	-0.00002
2761.73619	0.00004	2761.73619	-0.00003
2759.8076	0.00005	2759.8076	-0.00003
2757.87901	0.00006	2757.87901	-0.00003
2755.95042	0.00007	2755.95042	-0.00002
2754.02184	0.00006	2754.02184	-0.00003
2752.09325	0.00004	2752.09325	-0.00004
2750.16466	0.00001	2750.16466	-0.00007
2748.23608	0	2748.23608	-0.00008
2746.30749	0.00002	2746.30749	-0.00007
2744.3789	0.00007	2744.3789	-0.00003
2742.45032	0.00009	2742.45032	0.00001
2740.52173	0.00008	2740.52173	0.00003
2738.59314	0.00006	2738.59314	0.00004
2736.66456	0.00006	2736.66456	0.00004
2734.73597	0.00009	2734.73597	0.00005
2732.80738	0.00013	2732.80738	0.00007
2730.8788	0.00016	2730.8788	0.0001
2728.95021	0.00014	2728.95021	0.00012
2727.02162	0.0001	2727.02162	0.00012
2725.09304	0.00008	2725.09304	0.00011
2723.16445	0.0001	2723.16445	0.0001
2721.23586	0.00013	2721.23586	0.0001
2719.30728	0.00014	2719.30728	0.0001
2717.37869	0.00015	2717.37869	0.00009
2715.4501	0.00014	2715.4501	0.00008
2713.52152	0.00012	2713.52152	0.00007
2711.59293	0.00009	2711.59293	0.00005
2709.66434	0.00007	2709.66434	0.00003
2707.73576	0.00006	2707.73576	0.00001
2705.80717	0.00008	2705.80717	0.00001
2703.87858	0.00009	2703.87858	0.00001
2701.95	0.00009	2701.95	0
2700.02141	0.00008	2700.02141	-0.00002
2698.09282	0.00007	2698.09282	-0.00004
2696.16424	0.00006	2696.16424	-0.00006
2694.23565	0.00005	2694.23565	-0.00005
2692.30706	0.00004	2692.30706	-0.00003
2690.37848	0.00004	2690.37848	-0.00002
2688.44989	0.00004	2688.44989	-0.00001
2686.5213	0.00005	2686.5213	-0.00001

2684.59272	0.00004	2684.59272	0
2682.66413	0.00002	2682.66413	0
2680.73554	0	2680.73554	0
2678.80696	-0.00001	2678.80696	-0.00001
2676.87837	-0.00001	2676.87837	0
2674.94978	0	2674.94978	0.00001
2673.0212	0.00001	2673.0212	0.00002
2671.09261	0.00004	2671.09261	0.00002
2669.16402	0.00007	2669.16402	0.00004
2667.23544	0.0001	2667.23544	0.00007
2665.30685	0.00011	2665.30685	0.0001
2663.37826	0.00011	2663.37826	0.0001
2661.44968	0.00009	2661.44968	0.00008
2659.52109	0.00007	2659.52109	0.00007
2657.5925	0.00007	2657.5925	0.00007
2655.66392	0.00008	2655.66392	0.00007
2653.73533	0.00009	2653.73533	0.00008
2651.80674	0.00009	2651.80674	0.00008
2649.87816	0.00008	2649.87816	0.00007
2647.94957	0.00004	2647.94957	0.00005
2646.02098	0	2646.02098	0.00002
2644.0924	-0.00001	2644.0924	0
2642.16381	0.00002	2642.16381	-0.00001
2640.23522	0.00005	2640.23522	-0.00003
2638.30663	0.00006	2638.30663	-0.00004
2636.37805	0.00005	2636.37805	-0.00005
2634.44946	0.00004	2634.44946	-0.00006
2632.52087	0.00002	2632.52087	-0.00005
2630.59229	0	2630.59229	-0.00003
2628.6637	0	2628.6637	-0.00002
2626.73511	0.00003	2626.73511	-0.00003
2624.80653	0.00007	2624.80653	-0.00003
2622.87794	0.00008	2622.87794	-0.00003
2620.94935	0.00007	2620.94935	-0.00003
2619.02077	0.00002	2619.02077	-0.00005
2617.09218	0	2617.09218	-0.00005
2615.16359	0.00001	2615.16359	-0.00004
2613.23501	0.00005	2613.23501	-0.00002
2611.30642	0.00009	2611.30642	-0.00001
2609.37783	0.00012	2609.37783	-0.00001
2607.44925	0.00012	2607.44925	-0.00001
2605.52066	0.00009	2605.52066	0
2603.59207	0.00006	2603.59207	0

2601.66349	0.00004	2601.66349	0
2599.7349	0.00004	2599.7349	-0.00001
2597.80631	0.00007	2597.80631	-0.00002
2595.87773	0.00008	2595.87773	-0.00002
2593.94914	0.00008	2593.94914	-0.00001
2592.02055	0.00006	2592.02055	-0.00001
2590.09197	0.00004	2590.09197	-0.00002
2588.16338	0.00002	2588.16338	-0.00004
2586.23479	0.00002	2586.23479	-0.00004
2584.30621	0.00003	2584.30621	-0.00004
2582.37762	0.00006	2582.37762	-0.00003
2580.44903	0.00008	2580.44903	-0.00003
2578.52045	0.00008	2578.52045	-0.00003
2576.59186	0.00007	2576.59186	-0.00004
2574.66327	0.00003	2574.66327	-0.00005
2572.73469	0.00001	2572.73469	-0.00005
2570.8061	0	2570.8061	-0.00004
2568.87751	0.00001	2568.87751	-0.00003
2566.94893	0.00004	2566.94893	-0.00002
2565.02034	0.00007	2565.02034	-0.00003
2563.09175	0.00008	2563.09175	-0.00004
2561.16317	0.00006	2561.16317	-0.00004
2559.23458	0.00003	2559.23458	-0.00003
2557.30599	0.00001	2557.30599	-0.00001
2555.37741	0	2555.37741	0.00001
2553.44882	0.00001	2553.44882	0.00002
2551.52023	0.00005	2551.52023	0.00001
2549.59165	0.00009	2549.59165	0
2547.66306	0.00009	2547.66306	-0.00001
2545.73447	0.00007	2545.73447	0
2543.80589	0.00003	2543.80589	0.00001
2541.8773	-0.00001	2541.8773	0.00002
2539.94871	-0.00001	2539.94871	0.00004
2538.02013	0.00001	2538.02013	0.00005
2536.09154	0.00005	2536.09154	0.00003
2534.16295	0.00009	2534.16295	0.00003
2532.23437	0.00011	2532.23437	0.00004
2530.30578	0.00009	2530.30578	0.00005
2528.37719	0.00005	2528.37719	0.00006
2526.44861	0.00003	2526.44861	0.00006
2524.52002	0.00005	2524.52002	0.00005
2522.59143	0.00009	2522.59143	0.00003
2520.66284	0.00013	2520.66284	0.00003

2518.73426	0.00015	2518.73426	0.00005
2516.80567	0.00014	2516.80567	0.00008
2514.87708	0.0001	2514.87708	0.00009
2512.9485	0.00008	2512.9485	0.0001
2511.01991	0.00007	2511.01991	0.0001
2509.09132	0.0001	2509.09132	0.00009
2507.16274	0.00013	2507.16274	0.0001
2505.23415	0.00016	2505.23415	0.00011
2503.30556	0.00015	2503.30556	0.00011
2501.37698	0.00012	2501.37698	0.00011
2499.44839	0.00008	2499.44839	0.0001
2497.5198	0.00004	2497.5198	0.00008
2495.59122	0.00002	2495.59122	0.00008
2493.66263	0.00004	2493.66263	0.0001
2491.73404	0.00007	2491.73404	0.00013
2489.80546	0.0001	2489.80546	0.00015
2487.87687	0.0001	2487.87687	0.00017
2485.94828	0.00007	2485.94828	0.00019
2484.0197	0.00003	2484.0197	0.0002
2482.09111	0.00001	2482.09111	0.0002
2480.16252	0	2480.16252	0.00019
2478.23394	0.00002	2478.23394	0.00018
2476.30535	0.00005	2476.30535	0.0002
2474.37676	0.00007	2474.37676	0.00022
2472.44818	0.00006	2472.44818	0.00025
2470.51959	0.00003	2470.51959	0.00027
2468.591	0.00001	2468.591	0.00029
2466.66242	0	2466.66242	0.00032
2464.73383	0.00001	2464.73383	0.00035
2462.80524	0.00006	2462.80524	0.00036
2460.87666	0.00011	2460.87666	0.00037
2458.94807	0.00014	2458.94807	0.00038
2457.01948	0.00015	2457.01948	0.0004
2455.0909	0.00011	2455.0909	0.00043
2453.16231	0.00007	2453.16231	0.00045
2451.23372	0.00004	2451.23372	0.00046
2449.30514	0.00006	2449.30514	0.00049
2447.37655	0.00012	2447.37655	0.00052
2445.44796	0.00019	2445.44796	0.00056
2443.51938	0.00025	2443.51938	0.00058
2441.59079	0.00027	2441.59079	0.0006
2439.6622	0.00024	2439.6622	0.00063
2437.73362	0.00019	2437.73362	0.00068

2435.80503	0.00017	2435.80503	0.00071
2433.87644	0.0002	2433.87644	0.00074
2431.94786	0.00028	2431.94786	0.00075
2430.01927	0.00038	2430.01927	0.00078
2428.09068	0.00044	2428.09068	0.0008
2426.1621	0.00045	2426.1621	0.00082
2424.23351	0.00039	2424.23351	0.00085
2422.30492	0.00031	2422.30492	0.00089
2420.37634	0.00028	2420.37634	0.00093
2418.44775	0.00033	2418.44775	0.00094
2416.51916	0.00041	2416.51916	0.00097
2414.59058	0.0005	2414.59058	0.001
2412.66199	0.00056	2412.66199	0.00104
2410.7334	0.00057	2410.7334	0.00107
2408.80482	0.00054	2408.80482	0.0011
2406.87623	0.00051	2406.87623	0.00113
2404.94764	0.0005	2404.94764	0.00116
2403.01905	0.00054	2403.01905	0.00118
2401.09047	0.0006	2401.09047	0.0012
2399.16188	0.00066	2399.16188	0.00123
2397.23329	0.0007	2397.23329	0.00128
2395.30471	0.00072	2395.30471	0.00131
2393.37612	0.00071	2393.37612	0.00132
2391.44753	0.00069	2391.44753	0.00133
2389.51895	0.00067	2389.51895	0.00134
2387.59036	0.00065	2387.59036	0.00138
2385.66177	0.00066	2385.66177	0.00151
2383.73319	0.00065	2383.73319	0.00172
2381.8046	0.00061	2381.8046	0.00194
2379.87601	0.00051	2379.87601	0.0021
2377.94743	0.00037	2377.94743	0.00219
2376.01884	0.00019	2376.01884	0.00224
2374.09025	0.00003	2374.09025	0.00231
2372.16167	0	2372.16167	0.0024
2370.23308	0.00008	2370.23308	0.00248
2368.30449	0.00024	2368.30449	0.0025
2366.37591	0.0005	2366.37591	0.00247
2364.44732	0.00078	2364.44732	0.00238
2362.51873	0.00108	2362.51873	0.00228
2360.59015	0.00142	2360.59015	0.00219
2358.66156	0.00151	2358.66156	0.00209
2356.73297	0.00143	2356.73297	0.00192
2354.80439	0.00132	2354.80439	0.00146

2352.8758	0.00115	2352.8758	0.00044
2350.94721	0.00137	2350.94721	0
2349.01863	0.00211	2349.01863	0.00127
2347.09004	0.00214	2347.09004	0.0031
2345.16145	0.00093	2345.16145	0.00338
2343.23287	0.00001	2343.23287	0.00233
2341.30428	0.00031	2341.30428	0.00162
2339.37569	0.00095	2339.37569	0.00146
2337.44711	0.00125	2337.44711	0.00143
2335.51852	0.00128	2335.51852	0.00147
2333.58993	0.00131	2333.58993	0.00161
2331.66135	0.00125	2331.66135	0.00157
2329.73276	0.00101	2329.73276	0.00126
2327.80417	0.00089	2327.80417	0.00103
2325.87559	0.00107	2325.87559	0.00109
2323.947	0.00123	2323.947	0.00121
2322.01841	0.00112	2322.01841	0.00112
2320.08983	0.00093	2320.08983	0.00091
2318.16124	0.00091	2318.16124	0.00081
2316.23265	0.00101	2316.23265	0.00093
2314.30407	0.00101	2314.30407	0.0011
2312.37548	0.00088	2312.37548	0.00111
2310.44689	0.00077	2310.44689	0.00101
2308.51831	0.00076	2308.51831	0.00093
2306.58972	0.0008	2306.58972	0.0009
2304.66113	0.00081	2304.66113	0.00093
2302.73255	0.00074	2302.73255	0.00096
2300.80396	0.00063	2300.80396	0.00096
2298.87537	0.00055	2298.87537	0.00094
2296.94679	0.00053	2296.94679	0.00091
2295.0182	0.00052	2295.0182	0.00089
2293.08961	0.00052	2293.08961	0.00087
2291.16103	0.00052	2291.16103	0.00086
2289.23244	0.00049	2289.23244	0.00084
2287.30385	0.00044	2287.30385	0.00081
2285.37526	0.00038	2285.37526	0.00078
2283.44668	0.00035	2283.44668	0.00078
2281.51809	0.00031	2281.51809	0.00078
2279.5895	0.00027	2279.5895	0.00075
2277.66092	0.00023	2277.66092	0.00071
2275.73233	0.00022	2275.73233	0.00067
2273.80374	0.00021	2273.80374	0.00064
2271.87516	0.00019	2271.87516	0.00061

2269.94657	0.00017	2269.94657	0.00059
2268.01798	0.00015	2268.01798	0.00056
2266.0894	0.00014	2266.0894	0.00052
2264.16081	0.00014	2264.16081	0.00049
2262.23222	0.00014	2262.23222	0.00046
2260.30364	0.00015	2260.30364	0.00045
2258.37505	0.00016	2258.37505	0.00044
2256.44646	0.00016	2256.44646	0.00043
2254.51788	0.00014	2254.51788	0.0004
2252.58929	0.0001	2252.58929	0.00036
2250.6607	0.00005	2250.6607	0.00032
2248.73212	0.00005	2248.73212	0.00031
2246.80353	0.00006	2246.80353	0.00029
2244.87494	0.00006	2244.87494	0.00027
2242.94636	0.00007	2242.94636	0.00026
2241.01777	0.00007	2241.01777	0.00025
2239.08918	0.00007	2239.08918	0.00026
2237.1606	0.00005	2237.1606	0.00027
2235.23201	0.00002	2235.23201	0.00026
2233.30342	0.00001	2233.30342	0.00023
2231.37484	0.00001	2231.37484	0.00018
2229.44625	0.00002	2229.44625	0.00013
2227.51766	0.00002	2227.51766	0.00011
2225.58908	0.00001	2225.58908	0.00011
2223.66049	0.00001	2223.66049	0.00013
2221.7319	0.00001	2221.7319	0.00014
2219.80332	0.00001	2219.80332	0.00013
2217.87473	0.00001	2217.87473	0.00012
2215.94614	0.00001	2215.94614	0.00009
2214.01756	0.00001	2214.01756	0.00007
2212.08897	0.00001	2212.08897	0.00007
2210.16038	0	2210.16038	0.00008
2208.2318	0	2208.2318	0.00008
2206.30321	0	2206.30321	0.00006
2204.37462	0	2204.37462	0.00004
2202.44604	0.00002	2202.44604	0.00001
2200.51745	0.00003	2200.51745	0
2198.58886	0.00003	2198.58886	0
2196.66028	0.00001	2196.66028	0.00002
2194.73169	0	2194.73169	0.00003
2192.8031	0.00001	2192.8031	0.00003
2190.87452	0.00002	2190.87452	0.00002
2188.94593	0.00004	2188.94593	0.00001

2187.01734	0.00005	2187.01734	0.00001
2185.08876	0.00005	2185.08876	0.00002
2183.16017	0.00005	2183.16017	0.00003
2181.23158	0.00004	2181.23158	0.00003
2179.303	0.00002	2179.303	0.00002
2177.37441	0	2177.37441	0
2175.44582	0	2175.44582	0
2173.51724	0.00001	2173.51724	0.00002
2171.58865	0.00004	2171.58865	0.00004
2169.66006	0.00006	2169.66006	0.00004
2167.73147	0.00006	2167.73147	0.00003
2165.80289	0.00005	2165.80289	0.00001
2163.8743	0.00003	2163.8743	0.00001
2161.94571	0.00001	2161.94571	0
2160.01713	0	2160.01713	-0.00001
2158.08854	0.00002	2158.08854	-0.00002
2156.15995	0.00005	2156.15995	-0.00001
2154.23137	0.00009	2154.23137	0
2152.30278	0.00011	2152.30278	0.00001
2150.37419	0.00009	2150.37419	0.00001
2148.44561	0.00005	2148.44561	0.00002
2146.51702	0.00003	2146.51702	0.00003
2144.58843	0.00002	2144.58843	0.00003
2142.65985	0.00002	2142.65985	0.00001
2140.73126	0.00007	2140.73126	0.00001
2138.80267	0.00013	2138.80267	0.00004
2136.87409	0.00015	2136.87409	0.00004
2134.9455	0.00012	2134.9455	0.00003
2133.01691	0.00007	2133.01691	0.00002
2131.08833	0.00003	2131.08833	0.00002
2129.15974	0.00001	2129.15974	0.00001
2127.23115	0.00005	2127.23115	0.00001
2125.30257	0.00011	2125.30257	0.00002
2123.37398	0.00016	2123.37398	0.00004
2121.44539	0.00017	2121.44539	0.00005
2119.51681	0.00015	2119.51681	0.00007
2117.58822	0.00012	2117.58822	0.00008
2115.65963	0.0001	2115.65963	0.00007
2113.73105	0.00008	2113.73105	0.00005
2111.80246	0.00009	2111.80246	0.00005
2109.87387	0.00013	2109.87387	0.00008
2107.94529	0.00017	2107.94529	0.00009
2106.0167	0.0002	2106.0167	0.00009

2104.08811	0.00021	2104.08811	0.0001
2102.15953	0.0002	2102.15953	0.00011
2100.23094	0.00018	2100.23094	0.00013
2098.30235	0.00016	2098.30235	0.00014
2096.37377	0.00016	2096.37377	0.00016
2094.44518	0.00019	2094.44518	0.00017
2092.51659	0.00023	2092.51659	0.00018
2090.58801	0.00024	2090.58801	0.00017
2088.65942	0.00026	2088.65942	0.00014
2086.73083	0.00029	2086.73083	0.00016
2084.80225	0.00028	2084.80225	0.00021
2082.87366	0.00024	2082.87366	0.00023
2080.94507	0.00022	2080.94507	0.00023
2079.01649	0.00024	2079.01649	0.00024
2077.0879	0.00029	2077.0879	0.00025
2075.15931	0.00036	2075.15931	0.00027
2073.23073	0.0004	2073.23073	0.00029
2071.30214	0.00039	2071.30214	0.00029
2069.37355	0.00038	2069.37355	0.0003
2067.44497	0.00032	2067.44497	0.00034
2065.51638	0.00016	2065.51638	0.00026
2063.58779	0.00016	2063.58779	0.00017
2061.65921	0.00034	2061.65921	0.00023
2059.73062	0.00045	2059.73062	0.00032
2057.80203	0.00047	2057.80203	0.00033
2055.87345	0.00047	2055.87345	0.00032
2053.94486	0.00041	2053.94486	0.00031
2052.01627	0.00034	2052.01627	0.00032
2050.08768	0.00029	2050.08768	0.00035
2048.1591	0.0003	2048.1591	0.00037
2046.23051	0.00037	2046.23051	0.00039
2044.30192	0.00046	2044.30192	0.00041
2042.37334	0.00043	2042.37334	0.00036
2040.44475	0.00037	2040.44475	0.00027
2038.51616	0.00041	2038.51616	0.00028
2036.58758	0.00041	2036.58758	0.00036
2034.65899	0.00036	2034.65899	0.00038
2032.7304	0.00035	2032.7304	0.00037
2030.80182	0.0004	2030.80182	0.00037
2028.87323	0.00045	2028.87323	0.00038
2026.94464	0.00048	2026.94464	0.00038
2025.01606	0.00045	2025.01606	0.00036
2023.08747	0.00038	2023.08747	0.00031

2021.15888	0.00037	2021.15888	0.00033
2019.2303	0.00031	2019.2303	0.00036
2017.30171	0.00013	2017.30171	0.00022
2015.37312	0.00021	2015.37312	0.0001
2013.44454	0.00046	2013.44454	0.00024
2011.51595	0.00051	2011.51595	0.00038
2009.58736	0.00046	2009.58736	0.00039
2007.65878	0.00042	2007.65878	0.00036
2005.73019	0.0004	2005.73019	0.00033
2003.8016	0.0004	2003.8016	0.00033
2001.87302	0.0004	2001.87302	0.00034
1999.94443	0.00037	1999.94443	0.00032
1998.01584	0.00032	1998.01584	0.00027
1996.08726	0.00045	1996.08726	0.00036
1994.15867	0.00043	1994.15867	0.00049
1992.23008	0.00009	1992.23008	0.00027
1990.3015	0.00008	1990.3015	0
1988.37291	0.00032	1988.37291	0.00008
1986.44432	0.00044	1986.44432	0.00024
1984.51574	0.0005	1984.51574	0.00034
1982.58715	0.00051	1982.58715	0.00039
1980.65856	0.00047	1980.65856	0.00037
1978.72998	0.00044	1978.72998	0.00037
1976.80139	0.0004	1976.80139	0.00037
1974.8728	0.00037	1974.8728	0.00035
1972.94422	0.00037	1972.94422	0.0003
1971.01563	0.00051	1971.01563	0.00036
1969.08704	0.00049	1969.08704	0.00042
1967.15846	0.00021	1967.15846	0.0002
1965.22987	0.00027	1965.22987	0.00004
1963.30128	0.00052	1963.30128	0.00024
1961.3727	0.00048	1961.3727	0.00039
1959.44411	0.00041	1959.44411	0.00039
1957.51552	0.00046	1957.51552	0.00041
1955.58694	0.00049	1955.58694	0.0004
1953.65835	0.00055	1953.65835	0.00035
1951.72976	0.00063	1951.72976	0.00038
1949.80118	0.00061	1949.80118	0.00041
1947.87259	0.00048	1947.87259	0.00041
1945.944	0.00046	1945.944	0.00052
1944.01542	0.00024	1944.01542	0.00051
1942.08683	0	1942.08683	0.00011
1940.15824	0.00043	1940.15824	0.00002

1938.22966	0.0009	1938.22966	0.00042
1936.30107	0.00089	1936.30107	0.00066
1934.37248	0.00073	1934.37248	0.00065
1932.44389	0.00062	1932.44389	0.00061
1930.51531	0.00057	1930.51531	0.0006
1928.58672	0.00058	1928.58672	0.0006
1926.65813	0.00073	1926.65813	0.00074
1924.72955	0.00058	1924.72955	0.00075
1922.80096	0.00028	1922.80096	0.00042
1920.87237	0.00061	1920.87237	0.00045
1918.94379	0.00061	1918.94379	0.00063
1917.0152	0.00032	1917.0152	0.00034
1915.08661	0.00069	1915.08661	0.00034
1913.15803	0.00103	1913.15803	0.00082
1911.22944	0.00084	1911.22944	0.001
1909.30085	0.00053	1909.30085	0.00076
1907.37227	0.00064	1907.37227	0.00062
1905.44368	0.00091	1905.44368	0.00079
1903.51509	0.00097	1903.51509	0.00097
1901.58651	0.00091	1901.58651	0.00102
1899.65792	0.00094	1899.65792	0.00109
1897.72933	0.00092	1897.72933	0.00115
1895.80075	0.0007	1895.80075	0.00098
1893.87216	0.00079	1893.87216	0.00086
1891.94357	0.00102	1891.94357	0.00107
1890.01499	0.0007	1890.01499	0.00099
1888.0864	0.00062	1888.0864	0.00067
1886.15781	0.00101	1886.15781	0.0008
1884.22923	0.00114	1884.22923	0.00109
1882.30064	0.0011	1882.30064	0.00119
1880.37205	0.00109	1880.37205	0.00123
1878.44347	0.00108	1878.44347	0.00123
1876.51488	0.00103	1876.51488	0.00115
1874.58629	0.0011	1874.58629	0.00113
1872.65771	0.00129	1872.65771	0.00141
1870.72912	0.0009	1870.72912	0.00148
1868.80053	0	1868.80053	0.0007
1866.87195	0.00006	1866.87195	0
1864.94336	0.00097	1864.94336	0.00038
1863.01477	0.00142	1863.01477	0.0011
1861.08619	0.00128	1861.08619	0.00134
1859.1576	0.00111	1859.1576	0.00125
1857.22901	0.0011	1857.22901	0.00116

1855.30043	0.00114	1855.30043	0.00116
1853.37184	0.00113	1853.37184	0.00115
1851.44325	0.00107	1851.44325	0.00111
1849.51467	0.00102	1849.51467	0.00112
1847.58608	0.00107	1847.58608	0.00129
1845.65749	0.00062	1845.65749	0.00113
1843.72891	-0.00003	1843.72891	0.00027
1841.80032	0.00055	1841.80032	-0.00003
1839.87173	0.00138	1839.87173	0.00065
1837.94315	0.00128	1837.94315	0.00103
1836.01456	0.001	1836.01456	0.00087
1834.08597	0.00127	1834.08597	0.00101
1832.15739	0.00115	1832.15739	0.00128
1830.2288	0.00026	1830.2288	0.00075
1828.30021	0.00023	1828.30021	0.00017
1826.37163	0.00076	1826.37163	0.00037
1824.44304	0.00078	1824.44304	0.00048
1822.51445	0.00105	1822.51445	0.00053
1820.58587	0.00144	1820.58587	0.00096
1818.65728	0.00138	1818.65728	0.00123
1816.72869	0.00121	1816.72869	0.00115
1814.8001	0.0013	1814.8001	0.00119
1812.87152	0.00114	1812.87152	0.00125
1810.94293	0.00068	1810.94293	0.00092
1809.01434	0.00065	1809.01434	0.00057
1807.08576	0.00108	1807.08576	0.0007
1805.15717	0.00146	1805.15717	0.00117
1803.22858	0.00139	1803.22858	0.00144
1801.3	0.00082	1801.3	0.00105
1799.37141	0.00058	1799.37141	0.00042
1797.44282	0.00109	1797.44282	0.00048
1795.51424	0.00154	1795.51424	0.00118
1793.58565	0.00085	1793.58565	0.00127
1791.65706	0	1791.65706	0.00029
1789.72848	0.00074	1789.72848	0
1787.79989	0.00184	1787.79989	0.00095
1785.8713	0.00187	1785.8713	0.00161
1783.94272	0.00157	1783.94272	0.00154
1782.01413	0.00132	1782.01413	0.00131
1780.08554	0.00105	1780.08554	0.00097
1778.15696	0.00132	1778.15696	0.00093
1776.22837	0.00176	1776.22837	0.00153
1774.29978	0.00115	1774.29978	0.00179

1772.3712	-0.00002	1772.3712	0.00085
1770.44261	0.00026	1770.44261	0.00005
1768.51402	0.00127	1768.51402	0.00029
1766.58544	0.00198	1766.58544	0.00099
1764.65685	0.00226	1764.65685	0.00179
1762.72826	0.00151	1762.72826	0.00186
1760.79968	0.00102	1760.79968	0.00125
1758.87109	0.0013	1758.87109	0.00111
1756.9425	0.00126	1756.9425	0.00112
1755.01392	0.00165	1755.01392	0.00129
1753.08533	0.00191	1753.08533	0.00191
1751.15674	0.00089	1751.15674	0.00164
1749.22816	0	1749.22816	0.00041
1747.29957	0.00048	1747.29957	0
1745.37098	0.00134	1745.37098	0.00061
1743.4424	0.00169	1743.4424	0.00133
1741.51381	0.00138	1741.51381	0.00146
1739.58522	0.00122	1739.58522	0.00117
1737.65664	0.00227	1737.65664	0.00178
1735.72805	0.00211	1735.72805	0.00259
1733.79946	0.00039	1733.79946	0.00148
1731.87088	0.00077	1731.87088	0.00031
1729.94229	0.00209	1729.94229	0.00089
1728.0137	0.00275	1728.0137	0.0019
1726.08512	0.00284	1726.08512	0.00249
1724.15653	0.00249	1724.15653	0.0025
1722.22794	0.00266	1722.22794	0.00264
1720.29936	0.00262	1720.29936	0.00326
1718.37077	0.00102	1718.37077	0.00267
1716.44218	0.00001	1716.44218	0.00069
1714.5136	0.00117	1714.5136	0.00005
1712.58501	0.0028	1712.58501	0.00112
1710.65642	0.00353	1710.65642	0.0024
1708.72784	0.00325	1708.72784	0.00303
1706.79925	0.00218	1706.79925	0.00258
1704.87066	0.00212	1704.87066	0.00197
1702.94208	0.00305	1702.94208	0.00253
1701.01349	0.00235	1701.01349	0.00265
1699.0849	0.00192	1699.0849	0.00157
1697.15631	0.00249	1697.15631	0.00131
1695.22773	0.00284	1695.22773	0.00152
1693.29914	0.00423	1693.29914	0.00239
1691.37055	0.00496	1691.37055	0.00371

1689.44197	0.0048	1689.44197	0.00441
1687.51338	0.00501	1687.51338	0.00521
1685.58479	0.00391	1685.58479	0.00558
1683.65621	0.00266	1683.65621	0.00422
1681.72762	0.00458	1681.72762	0.00396
1679.79903	0.00713	1679.79903	0.00596
1677.87045	0.00808	1677.87045	0.00814
1675.94186	0.00752	1675.94186	0.00889
1674.01327	0.00779	1674.01327	0.009
1672.08469	0.00891	1672.08469	0.00995
1670.1561	0.0087	1670.1561	0.01061
1668.22751	0.00924	1668.22751	0.01076
1666.29893	0.01162	1666.29893	0.01239
1664.37034	0.01267	1664.37034	0.01446
1662.44175	0.01237	1662.44175	0.01509
1660.51317	0.01336	1660.51317	0.01545
1658.58458	0.01551	1658.58458	0.01709
1656.65599	0.01669	1656.65599	0.0195
1654.72741	0.01481	1654.72741	0.02015
1652.79882	0.01312	1652.79882	0.01796
1650.87023	0.01692	1650.87023	0.01805
1648.94165	0.0198	1648.94165	0.02093
1647.01306	0.01884	1647.01306	0.02205
1645.08447	0.01927	1645.08447	0.02192
1643.15589	0.02157	1643.15589	0.02327
1641.2273	0.02308	1641.2273	0.02527
1639.29871	0.02348	1639.29871	0.02687
1637.37013	0.0224	1637.37013	0.02738
1635.44154	0.02128	1635.44154	0.02642
1633.51295	0.02312	1633.51295	0.02646
1631.58437	0.02551	1631.58437	0.02843
1629.65578	0.02573	1629.65578	0.03005
1627.72719	0.0252	1627.72719	0.03045
1625.79861	0.02518	1625.79861	0.03069
1623.87002	0.02468	1623.87002	0.03064
1621.94143	0.02486	1621.94143	0.03052
1620.01285	0.02598	1620.01285	0.03155
1618.08426	0.02503	1618.08426	0.03215
1616.15567	0.02316	1616.15567	0.03079
1614.22709	0.02354	1614.22709	0.02995
1612.2985	0.02426	1612.2985	0.03049
1610.36991	0.02351	1610.36991	0.03056
1608.44133	0.0223	1608.44133	0.02974

1606.51274	0.02161	1606.51274	0.02894
1604.58415	0.02105	1604.58415	0.02842
1602.65557	0.02023	1602.65557	0.02775
1600.72698	0.01953	1600.72698	0.02695
1598.79839	0.01906	1598.79839	0.02623
1596.86981	0.01838	1596.86981	0.02541
1594.94122	0.01747	1594.94122	0.02429
1593.01263	0.0167	1593.01263	0.02314
1591.08405	0.01603	1591.08405	0.02215
1589.15546	0.01526	1589.15546	0.0212
1587.22687	0.01452	1587.22687	0.02027
1585.29829	0.01383	1585.29829	0.01933
1583.3697	0.01321	1583.3697	0.0183
1581.44111	0.01299	1581.44111	0.01751
1579.51252	0.01272	1579.51252	0.01716
1577.58394	0.01075	1577.58394	0.01588
1575.65535	0.0087	1575.65535	0.01316
1573.72676	0.00966	1573.72676	0.01209
1571.79818	0.01028	1571.79818	0.01269
1569.86959	0.00853	1569.86959	0.01184
1567.941	0.00768	1567.941	0.01005
1566.01242	0.00813	1566.01242	0.00933
1564.08383	0.00878	1564.08383	0.00958
1562.15524	0.00874	1562.15524	0.01049
1560.22666	0.00535	1560.22666	0.00955
1558.29807	0.00207	1558.29807	0.00531
1556.36948	0.00417	1556.36948	0.0035
1554.4409	0.00636	1554.4409	0.00466
1552.51231	0.00657	1552.51231	0.00562
1550.58372	0.00586	1550.58372	0.00554
1548.65514	0.00562	1548.65514	0.00522
1546.72655	0.00571	1546.72655	0.00534
1544.79796	0.00485	1544.79796	0.00521
1542.86938	0.00325	1542.86938	0.00422
1540.94079	0.00105	1540.94079	0.00211
1539.0122	0.00102	1539.0122	0
1537.08362	0.00433	1537.08362	0.00097
1535.15503	0.00529	1535.15503	0.00284
1533.22644	0.00354	1533.22644	0.00251
1531.29786	0.00353	1531.29786	0.00178
1529.36927	0.00423	1529.36927	0.00227
1527.44068	0.00352	1527.44068	0.00231
1525.5121	0.00289	1525.5121	0.00171

1523.58351	0.00253	1523.58351	0.0014
1521.65492	0.00173	1521.65492	0.0007
1519.72634	0.00239	1519.72634	0.00027
1517.79775	0.00309	1517.79775	0.00061
1515.86916	0.00307	1515.86916	0.00077
1513.94058	0.00372	1513.94058	0.00118
1512.01199	0.00447	1512.01199	0.00223
1510.0834	0.0041	1510.0834	0.00318
1508.15482	0.00154	1508.15482	0.00251
1506.22623	0	1506.22623	0.00013
1504.29764	0.00291	1504.29764	0
1502.36906	0.00563	1502.36906	0.00215
1500.44047	0.00553	1500.44047	0.00375
1498.51188	0.00386	1498.51188	0.00356
1496.5833	0.00283	1496.5833	0.00251
1494.65471	0.00398	1494.65471	0.00259
1492.72612	0.00511	1492.72612	0.00388
1490.79754	0.00401	1490.79754	0.00417
1488.86895	0.00286	1488.86895	0.00303
1486.94036	0.00369	1486.94036	0.00272
1485.01178	0.00513	1485.01178	0.00381
1483.08319	0.00549	1483.08319	0.00487
1481.1546	0.00516	1481.1546	0.00521
1479.22602	0.00524	1479.22602	0.00541
1477.29743	0.00535	1477.29743	0.0058
1475.36884	0.00488	1475.36884	0.0059
1473.44026	0.00392	1473.44026	0.00535
1471.51167	0.00436	1471.51167	0.00498
1469.58308	0.00671	1469.58308	0.00632
1467.6545	0.00794	1467.6545	0.0084
1465.72591	0.00708	1465.72591	0.00913
1463.79732	0.00695	1463.79732	0.00895
1461.86873	0.00857	1461.86873	0.00996
1459.94015	0.00885	1459.94015	0.0116
1458.01156	0.00672	1458.01156	0.01143
1456.08297	0.00636	1456.08297	0.01018
1454.15439	0.00884	1454.15439	0.01088
1452.2258	0.01055	1452.2258	0.01275
1450.29721	0.01046	1450.29721	0.01386
1448.36863	0.00963	1448.36863	0.01383
1446.44004	0.00949	1446.44004	0.01357
1444.51145	0.01006	1444.51145	0.01382
1442.58287	0.01051	1442.58287	0.0143

1440.65428	0.01089	1440.65428	0.01482
1438.72569	0.01038	1438.72569	0.01499
1436.79711	0.00898	1436.79711	0.01397
1434.86852	0.00915	1434.86852	0.01293
1432.93993	0.01015	1432.93993	0.01323
1431.01135	0.00984	1431.01135	0.01344
1429.08276	0.00925	1429.08276	0.01285
1427.15417	0.00939	1427.15417	0.01245
1425.22559	0.00924	1425.22559	0.01218
1423.297	0.00906	1423.297	0.01185
1421.36841	0.00883	1421.36841	0.01174
1419.43983	0.00763	1419.43983	0.01099
1417.51124	0.00721	1417.51124	0.00974
1415.58265	0.00828	1415.58265	0.00972
1413.65407	0.00893	1413.65407	0.01045
1411.72548	0.00879	1411.72548	0.01075
1409.79689	0.00877	1409.79689	0.0108
1407.86831	0.00892	1407.86831	0.01105
1405.93972	0.00861	1405.93972	0.01107
1404.01113	0.00858	1404.01113	0.01083
1402.08255	0.00914	1402.08255	0.01098
1400.15396	0.00897	1400.15396	0.01103
1398.22537	0.00833	1398.22537	0.01045
1396.29679	0.0078	1396.29679	0.00967
1394.3682	0.00738	1394.3682	0.00893
1392.43961	0.00778	1392.43961	0.00878
1390.51103	0.00829	1390.51103	0.00942
1388.58244	0.00795	1388.58244	0.00992
1386.65385	0.00791	1386.65385	0.01004
1384.72527	0.0088	1384.72527	0.01041
1382.79668	0.00918	1382.79668	0.01076
1380.86809	0.0086	1380.86809	0.01053
1378.93951	0.00795	1378.93951	0.01013
1377.01092	0.00744	1377.01092	0.0099
1375.08233	0.00659	1375.08233	0.0094
1373.15375	0.00612	1373.15375	0.00866
1371.22516	0.00635	1371.22516	0.00833
1369.29657	0.00626	1369.29657	0.0081
1367.36799	0.00605	1367.36799	0.0077
1365.4394	0.00582	1365.4394	0.0074
1363.51081	0.005	1363.51081	0.0068
1361.58223	0.00443	1361.58223	0.00595
1359.65364	0.00475	1359.65364	0.00574

1357.72505	0.00502	1357.72505	0.00604
1355.79647	0.00478	1355.79647	0.00612
1353.86788	0.00454	1353.86788	0.00593
1351.93929	0.00451	1351.93929	0.00579
1350.0107	0.00449	1350.0107	0.00573
1348.08212	0.00449	1348.08212	0.00567
1346.15353	0.00458	1346.15353	0.00568
1344.22494	0.00468	1344.22494	0.00579
1342.29636	0.00443	1342.29636	0.00584
1340.36777	0.00363	1340.36777	0.00547
1338.43918	0.00321	1338.43918	0.00491
1336.5106	0.00369	1336.5106	0.00488
1334.58201	0.00429	1334.58201	0.00531
1332.65342	0.00449	1332.65342	0.00563
1330.72484	0.00443	1330.72484	0.00566
1328.79625	0.00432	1328.79625	0.00555
1326.86766	0.00416	1326.86766	0.00545
1324.93908	0.004	1324.93908	0.00539
1323.01049	0.00388	1323.01049	0.00539
1321.0819	0.00369	1321.0819	0.00533
1319.15332	0.00351	1319.15332	0.00511
1317.22473	0.00362	1317.22473	0.00489
1315.29614	0.00379	1315.29614	0.00479
1313.36756	0.00365	1313.36756	0.00463
1311.43897	0.00348	1311.43897	0.00443
1309.51038	0.00333	1309.51038	0.0043
1307.5818	0.00309	1307.5818	0.00414
1305.65321	0.00291	1305.65321	0.00389
1303.72462	0.0028	1303.72462	0.00363
1301.79604	0.00271	1301.79604	0.0034
1299.86745	0.00264	1299.86745	0.00323
1297.93886	0.00255	1297.93886	0.00311
1296.01028	0.00246	1296.01028	0.00298
1294.08169	0.00238	1294.08169	0.00285
1292.1531	0.00228	1292.1531	0.00274
1290.22452	0.00213	1290.22452	0.00261
1288.29593	0.00195	1288.29593	0.00251
1286.36734	0.00191	1286.36734	0.00252
1284.43876	0.00202	1284.43876	0.00268
1282.51017	0.00215	1282.51017	0.00283
1280.58158	0.00227	1280.58158	0.00286
1278.653	0.00235	1278.653	0.00283
1276.72441	0.00235	1276.72441	0.00283

1274.79582	0.00224	1274.79582	0.00289
1272.86724	0.00205	1272.86724	0.00298
1270.93865	0.00192	1270.93865	0.00303
1269.01006	0.00202	1269.01006	0.00308
1267.08148	0.00224	1267.08148	0.00313
1265.15289	0.00243	1265.15289	0.00312
1263.2243	0.00252	1263.2243	0.0031
1261.29572	0.00247	1261.29572	0.00313
1259.36713	0.0024	1259.36713	0.0032
1257.43854	0.00241	1257.43854	0.00334
1255.50996	0.00245	1255.50996	0.00346
1253.58137	0.0025	1253.58137	0.00348
1251.65278	0.0026	1251.65278	0.00345
1249.7242	0.00271	1249.7242	0.00344
1247.79561	0.00278	1247.79561	0.0035
1245.86702	0.00277	1245.86702	0.00356
1243.93844	0.0027	1243.93844	0.00357
1242.00985	0.00263	1242.00985	0.00355
1240.08126	0.00261	1240.08126	0.00352
1238.15268	0.00265	1238.15268	0.00354
1236.22409	0.0027	1236.22409	0.00361
1234.2955	0.00276	1234.2955	0.0037
1232.36691	0.00281	1232.36691	0.00376
1230.43833	0.00284	1230.43833	0.00377
1228.50974	0.00287	1228.50974	0.00373
1226.58115	0.00289	1226.58115	0.00371
1224.65257	0.00288	1224.65257	0.00373
1222.72398	0.00286	1222.72398	0.00383
1220.79539	0.00288	1220.79539	0.00397
1218.86681	0.00296	1218.86681	0.00409
1216.93822	0.00311	1216.93822	0.00416
1215.00963	0.00332	1215.00963	0.00423
1213.08105	0.00351	1213.08105	0.00434
1211.15246	0.00364	1211.15246	0.00452
1209.22387	0.00372	1209.22387	0.00478
1207.29529	0.00377	1207.29529	0.00506
1205.3667	0.00381	1205.3667	0.00526
1203.43811	0.00395	1203.43811	0.0054
1201.50953	0.00416	1201.50953	0.00552
1199.58094	0.0043	1199.58094	0.00558
1197.65235	0.00445	1197.65235	0.00568
1195.72377	0.00453	1195.72377	0.0058
1193.79518	0.00456	1193.79518	0.00593

1191.86659	0.00462	1191.86659	0.00608
1189.93801	0.00476	1189.93801	0.00629
1188.00942	0.00501	1188.00942	0.00658
1186.08083	0.00535	1186.08083	0.00697
1184.15225	0.00572	1184.15225	0.00748
1182.22366	0.00608	1182.22366	0.00805
1180.29507	0.00634	1180.29507	0.0085
1178.36649	0.00652	1178.36649	0.0088
1176.4379	0.00675	1176.4379	0.00909
1174.50931	0.00706	1174.50931	0.00942
1172.58073	0.00738	1172.58073	0.00977
1170.65214	0.00766	1170.65214	0.01014
1168.72355	0.00788	1168.72355	0.01048
1166.79497	0.00803	1166.79497	0.01076
1164.86638	0.00817	1164.86638	0.01097
1162.93779	0.00833	1162.93779	0.01112
1161.00921	0.00849	1161.00921	0.01119
1159.08062	0.00864	1159.08062	0.01119
1157.15203	0.00874	1157.15203	0.0112
1155.22345	0.00875	1155.22345	0.01123
1153.29486	0.00874	1153.29486	0.0113
1151.36627	0.00878	1151.36627	0.01137
1149.43769	0.00888	1149.43769	0.01137
1147.5091	0.00906	1147.5091	0.01135
1145.58051	0.00928	1145.58051	0.01135
1143.65193	0.00945	1143.65193	0.01139
1141.72334	0.00958	1141.72334	0.01156
1139.79475	0.0097	1139.79475	0.0119
1137.86617	0.00986	1137.86617	0.01238
1135.93758	0.01017	1135.93758	0.01297
1134.00899	0.01064	1134.00899	0.01361
1132.08041	0.01124	1132.08041	0.01425
1130.15182	0.01187	1130.15182	0.01494
1128.22323	0.0124	1128.22323	0.01569
1126.29465	0.0128	1126.29465	0.01653
1124.36606	0.01321	1124.36606	0.01751
1122.43747	0.01369	1122.43747	0.01857
1120.50889	0.01421	1120.50889	0.01957
1118.5803	0.01478	1118.5803	0.02043
1116.65171	0.01528	1116.65171	0.02106
1114.72312	0.01557	1114.72312	0.02132
1112.79454	0.0156	1112.79454	0.02119
1110.86595	0.01545	1110.86595	0.02088

1108.93736	0.01523	1108.93736	0.0206
1107.00878	0.01506	1107.00878	0.02041
1105.08019	0.01497	1105.08019	0.0203
1103.1516	0.01495	1103.1516	0.02024
1101.22302	0.01497	1101.22302	0.02024
1099.29443	0.01497	1099.29443	0.02027
1097.36584	0.01493	1097.36584	0.0203
1095.43726	0.01486	1095.43726	0.02033
1093.50867	0.01477	1093.50867	0.02032
1091.58008	0.01462	1091.58008	0.02031
1089.6515	0.01449	1089.6515	0.02032
1087.72291	0.01445	1087.72291	0.02036
1085.79432	0.01452	1085.79432	0.02049
1083.86574	0.01472	1083.86574	0.02074
1081.93715	0.01492	1081.93715	0.02107
1080.00856	0.01503	1080.00856	0.0214
1078.07998	0.01506	1078.07998	0.0217
1076.15139	0.01504	1076.15139	0.02198
1074.2228	0.01506	1074.2228	0.02226
1072.29422	0.01521	1072.29422	0.02255
1070.36563	0.01544	1070.36563	0.02287
1068.43704	0.01569	1068.43704	0.02325
1066.50846	0.01592	1066.50846	0.02374
1064.57987	0.01608	1064.57987	0.02431
1062.65128	0.01621	1062.65128	0.02492
1060.7227	0.01636	1060.7227	0.02552
1058.79411	0.0166	1058.79411	0.02608
1056.86552	0.01695	1056.86552	0.02666
1054.93694	0.01735	1054.93694	0.02732
1053.00835	0.01771	1053.00835	0.02808
1051.07976	0.01795	1051.07976	0.0289
1049.15118	0.01811	1049.15118	0.02975
1047.22259	0.0183	1047.22259	0.0306
1045.294	0.01862	1045.294	0.0315
1043.36542	0.01908	1043.36542	0.03247
1041.43683	0.01958	1041.43683	0.03356
1039.50824	0.02005	1039.50824	0.03469
1037.57966	0.02038	1037.57966	0.03569
1035.65107	0.02045	1035.65107	0.03635
1033.72248	0.02024	1033.72248	0.03648
1031.7939	0.01981	1031.7939	0.036
1029.86531	0.01921	1029.86531	0.03494
1027.93672	0.01852	1027.93672	0.03353

1026.00814	0.0178	1026.00814	0.03204
1024.07955	0.0171	1024.07955	0.03074
1022.15096	0.0165	1022.15096	0.02978
1020.22238	0.01604	1020.22238	0.02922
1018.29379	0.01574	1018.29379	0.02904
1016.3652	0.01561	1016.3652	0.02914
1014.43662	0.01556	1014.43662	0.02935
1012.50803	0.01545	1012.50803	0.02935
1010.57944	0.01516	1010.57944	0.02888
1008.65086	0.0146	1008.65086	0.02783
1006.72227	0.01378	1006.72227	0.0263
1004.79368	0.01283	1004.79368	0.02449
1002.8651	0.01182	1002.8651	0.0226
1000.93651	0.01081	1000.93651	0.02071
999.00792	0.00987	999.00792	0.01886
997.07933	0.00897	997.07933	0.01702
995.15075	0.00807	995.15075	0.01523
993.22216	0.00724	993.22216	0.01359
991.29357	0.0065	991.29357	0.01216
989.36499	0.0058	989.36499	0.0109
987.4364	0.00511	987.4364	0.0097
985.50781	0.00443	985.50781	0.00846
983.57923	0.00375	983.57923	0.00718
981.65064	0.00312	981.65064	0.00598
979.72205	0.00258	979.72205	0.00492
977.79347	0.00213	977.79347	0.00404
975.86488	0.00175	975.86488	0.00328
973.93629	0.00139	973.93629	0.00257
972.00771	0.001	972.00771	0.00189
970.07912	0.00065	970.07912	0.00129
968.15053	0.00039	968.15053	0.00086
966.22195	0.00025	966.22195	0.00064
964.29336	0.00023	964.29336	0.00055
962.36477	0.00022	962.36477	0.00045
960.43619	0.00017	960.43619	0.00027
958.5076	0.00008	958.5076	0.00008
956.57901	0	956.57901	0
954.65043	-0.00001	954.65043	0.00006
952.72184	0.0001	952.72184	0.00037
950.79325	0.00039	950.79325	0.00084
948.86467	0.00074	948.86467	0.00137
946.93608	0.00106	946.93608	0.00188
945.00749	0.00125	945.00749	0.00232

943.07891	0.00129	943.07891	0.00271
941.15032	0.00128	941.15032	0.00306
939.22173	0.00128	939.22173	0.00331
937.29315	0.00134	937.29315	0.00337
935.36456	0.00144	935.36456	0.00322
933.43597	0.00148	933.43597	0.00294
931.50739	0.00143	931.50739	0.00274
929.5788	0.00136	929.5788	0.0028
927.65021	0.00132	927.65021	0.00312
925.72163	0.00141	925.72163	0.0036
923.79304	0.00167	923.79304	0.0041
921.86445	0.00202	921.86445	0.00454
919.93587	0.00235	919.93587	0.00503
918.00728	0.00267	918.00728	0.00566
916.07869	0.00292	916.07869	0.00632
914.15011	0.00305	914.15011	0.00677
912.22152	0.00306	912.22152	0.00676
910.29293	0.00293	910.29293	0.00625
908.36435	0.00271	908.36435	0.00544
906.43576	0.00243	906.43576	0.00464
904.50717	0.00213	904.50717	0.00405
902.57859	0.00186	902.57859	0.00372
900.65	0.00171	900.65	0.00351
898.72141	0.00169	898.72141	0.00329
896.79283	0.00177	896.79283	0.00305
894.86424	0.00188	894.86424	0.00289
892.93565	0.00191	892.93565	0.0029
891.00707	0.00189	891.00707	0.00305
889.07848	0.00188	889.07848	0.00325
887.14989	0.00193	887.14989	0.00339
885.22131	0.00209	885.22131	0.00346
883.29272	0.00235	883.29272	0.00353
881.36413	0.00258	881.36413	0.00369
879.43554	0.00276	879.43554	0.00401
877.50696	0.00289	877.50696	0.0044
875.57837	0.00293	875.57837	0.00466
873.64978	0.00294	873.64978	0.00463
871.7212	0.00298	871.7212	0.00436
869.79261	0.00298	869.79261	0.00406
867.86402	0.00292	867.86402	0.00391
865.93544	0.00276	865.93544	0.0039
864.00685	0.00249	864.00685	0.00381
862.07826	0.00223	862.07826	0.00347

860.14968	0.00207	860.14968	0.00291
858.22109	0.00197	858.22109	0.00231
856.2925	0.0019	856.2925	0.00189
854.36392	0.00177	854.36392	0.00173
852.43533	0.0015	852.43533	0.00164
850.50674	0.00122	850.50674	0.0014
848.57816	0.00097	848.57816	0.00102
846.64957	0.00082	846.64957	0.00065
844.72098	0.00082	844.72098	0.00041
842.7924	0.00093	842.7924	0.00041
840.86381	0.001	840.86381	0.0006
838.93522	0.001	838.93522	0.00082
837.00664	0.00093	837.00664	0.00091
835.07805	0.00085	835.07805	0.00088
833.14946	0.00086	833.14946	0.00086
831.22088	0.00096	831.22088	0.00097
829.29229	0.00112	829.29229	0.00121
827.3637	0.00132	827.3637	0.00151
825.43512	0.00149	825.43512	0.00173
823.50653	0.00157	823.50653	0.00182
821.57794	0.00166	821.57794	0.00192
819.64936	0.00179	819.64936	0.00218
817.72077	0.00196	817.72077	0.00266
815.79218	0.00222	815.79218	0.00325
813.8636	0.00252	813.8636	0.00373
811.93501	0.00276	811.93501	0.00397
810.00642	0.00294	810.00642	0.004
808.07784	0.00303	808.07784	0.00397
806.14925	0.00304	806.14925	0.00409
804.22066	0.00306	804.22066	0.00444
802.29208	0.0031	802.29208	0.00479
800.36349	0.00318	800.36349	0.00495
798.4349	0.00331	798.4349	0.00483
796.50632	0.00341	796.50632	0.00455
794.57773	0.00345	794.57773	0.00432
792.64914	0.00341	792.64914	0.00426
790.72056	0.00325	790.72056	0.00431
788.79197	0.00298	788.79197	0.00426
786.86338	0.00269	786.86338	0.00397
784.9348	0.00244	784.9348	0.00347
783.00621	0.00231	783.00621	0.00297
781.07762	0.0023	781.07762	0.00267
779.14904	0.00225	779.14904	0.00258

777.22045	0.0021	777.22045	0.00253
775.29186	0.00188	775.29186	0.0024
773.36328	0.00164	773.36328	0.00222
771.43469	0.00154	771.43469	0.00214
769.5061	0.00169	769.5061	0.00232
767.57752	0.00203	767.57752	0.00281
765.64893	0.00241	765.64893	0.00348
763.72034	0.00272	763.72034	0.00411
761.79175	0.00283	761.79175	0.00454
759.86317	0.00282	759.86317	0.00482
757.93458	0.00288	757.93458	0.00513
756.00599	0.00309	756.00599	0.0056
754.07741	0.00345	754.07741	0.00617
752.14882	0.00385	752.14882	0.00662
750.22023	0.00405	750.22023	0.00675
748.29165	0.00394	748.29165	0.00655
746.36306	0.00359	746.36306	0.0062
744.43447	0.00309	744.43447	0.00583
742.50589	0.00268	742.50589	0.00546
740.5773	0.00246	740.5773	0.00499
738.64871	0.00233	738.64871	0.00429
736.72013	0.00214	736.72013	0.0034
734.79154	0.00179	734.79154	0.00249
732.86295	0.00122	732.86295	0.00177
730.93437	0.00062	730.93437	0.00137
729.00578	0.00019	729.00578	0.00119
727.07719	0	727.07719	0.001
725.14861	0.00004	725.14861	0.00072
723.22002	0.00022	723.22002	0.00043
721.29143	0.00019	721.29143	0.00013
719.36285	0.00008	719.36285	0
717.43426	0.00004	717.43426	0.00003
715.50567	0.00002	715.50567	0.00021
713.57709	0.00001	713.57709	0.00026
711.6485	0.00011	711.6485	0.00011
709.71991	0.00028	709.71991	-0.00007
707.79133	0.00047	707.79133	-0.00006
705.86274	0.00063	705.86274	0.00021
703.93415	0.00073	703.93415	0.00062
702.00557	0.00081	702.00557	0.00098
700.07698	0.00092	700.07698	0.00111
698.14839	0.00103	698.14839	0.00104
696.21981	0.00112	696.21981	0.00093

694.29122	0.0012	694.29122	0.00096
692.36263	0.0013	692.36263	0.00114
690.43405	0.0014	690.43405	0.00131
688.50546	0.00144	688.50546	0.00129
686.57687	0.0014	686.57687	0.00107
684.64829	0.00132	684.64829	0.00081
682.7197	0.00124	682.7197	0.00069
680.79111	0.00121	680.79111	0.00078
678.86253	0.00131	678.86253	0.00094
676.93394	0.00154	676.93394	0.00094
675.00535	0.00203	675.00535	0.00076
673.07677	0.00268	673.07677	0.00083
671.14818	0.00238	671.14818	0.0013
669.21959	0.00069	669.21959	0.00123
667.29101	0	667.29101	0.00039
665.36242	0.00151	665.36242	0.00032
663.43383	0.00267	663.43383	0.00081
661.50525	0.00252	661.50525	0.00088
659.57666	0.00211	659.57666	0.00067
657.64807	0.00189	657.64807	0.00062
655.71949	0.00172	655.71949	0.00077
653.7909	0.00157	653.7909	0.00096
651.86231	0.00157	651.86231	0.00106
649.93373	0.00186	649.93373	0.00104
648.00514	0.00236	648.00514	0.00099
646.07655	0.00282	646.07655	0.00112
644.14796	0.003	644.14796	0.00147
642.21938	0.00287	642.21938	0.00185
640.29079	0.00267	640.29079	0.00212
638.3622	0.00262	638.3622	0.00221
636.43362	0.00281	636.43362	0.00211
634.50503	0.00313	634.50503	0.00197
632.57644	0.00346	632.57644	0.00197
630.64786	0.00363	630.64786	0.00218
628.71927	0.00358	628.71927	0.00247
626.79068	0.00333	626.79068	0.00265
624.8621	0.0033	624.8621	0.00277
622.93351	0.00365	622.93351	0.00298
621.00492	0.00419	621.00492	0.00337
619.07634	0.00468	619.07634	0.00386
617.14775	0.00501	617.14775	0.00437
615.21916	0.00528	615.21916	0.00491
613.29058	0.00544	613.29058	0.00536

611.36199	0.00541	611.36199	0.00562
609.4334	0.00544	609.4334	0.00584
607.50482	0.00562	607.50482	0.00613
605.57623	0.00588	605.57623	0.00646
603.64764	0.00612	603.64764	0.00671
601.71906	0.00613	601.71906	0.00678
599.79047	0.006	599.79047	0.00669
597.86188	0.00595	597.86188	0.00662
595.9333	0.00589	595.9333	0.00672
594.00471	0.00579	594.00471	0.00695
592.07612	0.00581	592.07612	0.0072
590.14754	0.0062	590.14754	0.00749
588.21895	0.00669	588.21895	0.0079
586.29036	0.00693	586.29036	0.00835
584.36178	0.0071	584.36178	0.00883
582.43319	0.00726	582.43319	0.00935
580.5046	0.00747	580.5046	0.00986
578.57602	0.00771	578.57602	0.01036
576.64743	0.0079	576.64743	0.01079
574.71884	0.00832	574.71884	0.01117
572.79026	0.00882	572.79026	0.01166
570.86167	0.00904	570.86167	0.01228
568.93308	0.00912	568.93308	0.01292
567.0045	0.00927	567.0045	0.01358
565.07591	0.00953	565.07591	0.01425
563.14732	0.00978	563.14732	0.01482
561.21874	0.01006	561.21874	0.01528
559.29015	0.01045	559.29015	0.01566
557.36156	0.01082	557.36156	0.01596
555.43298	0.011	555.43298	0.01631
553.50439	0.01097	553.50439	0.01677
551.5758	0.01074	551.5758	0.01721
549.64722	0.01048	549.64722	0.01754
547.71863	0.01055	547.71863	0.01784
545.79004	0.01103	545.79004	0.01823
543.86146	0.01169	543.86146	0.01868
541.93287	0.0121	541.93287	0.01899
540.00428	0.01195	540.00428	0.0189
538.0757	0.01129	538.0757	0.01837
536.14711	0.01043	536.14711	0.01749
534.21852	0.00971	534.21852	0.01637
532.28994	0.00922	532.28994	0.01508
530.36135	0.00893	530.36135	0.01362

528.43276	0.00835	528.43276	0.01199
526.50417	0.00726	526.50417	0.01022
524.57559	0.00645	524.57559	0.0087
522.647	0.00585	522.647	0.00757
520.71841	0.00488	520.71841	0.0064
518.78983	0.00389	518.78983	0.00494
516.86124	0.00353	516.86124	0.00362
514.93265	0.0037	514.93265	0.0028
513.00407	0.00367	513.00407	0.00229
511.07548	0.00327	511.07548	0.00183
509.14689	0.00272	509.14689	0.00138
507.21831	0.00236	507.21831	0.00094
505.28972	0.00226	505.28972	0.00056
503.36113	0.00195	503.36113	0.00023
501.43255	0.00179	501.43255	0
499.50396	0.00213	499.50396	0.00004
497.57537	0.00244	497.57537	0.00022
495.64679	0.00256	495.64679	0.00035
493.7182	0.00248	493.7182	0.00058
491.78961	0.00252	491.78961	0.00119
489.86103	0.00306	489.86103	0.00221
487.93244	0.00367	487.93244	0.00338
486.00385	0.00406	486.00385	0.00441
484.07527	0.00468	484.07527	0.00547
482.14668	0.00575	482.14668	0.00685
480.21809	0.00676	480.21809	0.00838
478.28951	0.00739	478.28951	0.00966
476.36092	0.00764	476.36092	0.01053
474.43233	0.00726	474.43233	0.01086
472.50375	0.00674	472.50375	0.01058
470.57516	0.00687	470.57516	0.01015
468.64657	0.00709	468.64657	0.00978
466.71799	0.00711	466.71799	0.0092
464.7894	0.00701	464.7894	0.00831
462.86081	0.00659	462.86081	0.00721
460.93223	0.00571	460.93223	0.006
459.00364	0.00429	459.00364	0.00462
457.07505	0.00353	457.07505	0.00323
455.14647	0.00385	455.14647	0.00233
453.21788	0.00395	453.21788	0.00196
451.28929	0.00373	451.28929	0.00186
449.36071	0.00329	449.36071	0.0018
447.43212	0.00256	447.43212	0.00146

445.50353	0.00204	445.50353	0.00097
443.57495	0.00208	443.57495	0.00081
441.64636	0.00283	441.64636	0.00129
439.71777	0.00372	439.71777	0.00222
437.78919	0.00374	437.78919	0.00298
435.8606	0.00323	435.8606	0.00331
433.93201	0.00299	433.93201	0.00346
432.00343	0.00302	432.00343	0.00359
430.07484	0.00336	430.07484	0.00373
428.14625	0.00384	428.14625	0.00381
426.21767	0.00397	426.21767	0.00353
424.28908	0.00387	424.28908	0.00288
422.36049	0.00329	422.36049	0.00218
420.43191	0.00129	420.43191	0.00141
418.50332	0	418.50332	0.0008
416.57473	0.00128	416.57473	0.001
414.64615	0.00261	414.64615	0.00164
412.71756	0.00258	412.71756	0.00172
410.78897	0.00205	410.78897	0.0013
408.86038	0.00133	408.86038	0.00096
406.9318	0.00049	406.9318	0.00083
405.00321	0.00001	405.00321	0.00064
403.07462	0.00003	403.07462	0.00034
401.14604	0.00002	401.14604	0
399.21745	0	399.21745	0

TG curve of water-immersed brown coal

temperature	***TG***	***TG***	***temperature***	***TG***	***temperature***	***TG***	***temperature***	***TG***
℃	%	%	℃	%	℃	%	℃	%
	Coal	Coal+MgCl2		Coal+TEMPO		Coal+TPPI		Coal+PA
29.991	100	100	29.267	100	28.57	100	31.33	100
30.991	99.93	99.94395	30.267	99.94566	29.57	99.91679	32.33	99.93615
31.991	99.85475	99.94419	31.267	99.89165	30.57	99.82116	33.33	99.85185
32.991	99.77389	99.9521	32.267	99.86799	31.57	99.7286	34.33	99.7638
33.991	99.67647	99.95922	33.267	99.85479	32.57	99.66399	35.33	99.66387
34.991	99.59671	99.96434	34.267	99.85267	33.57	99.6164	36.33	99.58404
35.991	99.53562	99.96798	35.267	99.84905	34.57	99.58812	37.33	99.52375
36.991	99.49634	99.96373	36.267	99.84947	35.57	99.57228	38.33	99.48043
37.991	99.47085	99.95974	37.267	99.84425	36.57	99.55681	39.33	99.45115
38.991	99.44437	99.94919	38.267	99.84077	37.57	99.54284	40.33	99.42147
39.991	99.42041	99.94177	39.267	99.83216	38.57	99.52367	41.33	99.39767
40.991	99.39979	99.93134	40.267	99.8315	39.57	99.50883	42.33	99.37942
41.991	99.38846	99.91901	41.267	99.82576	40.57	99.49184	43.33	99.36538
42.991	99.37311	99.90738	42.267	99.81802	41.57	99.46864	44.33	99.35449
43.991	99.35821	99.89192	43.267	99.81276	42.57	99.44175	45.33	99.34593
44.991	99.34551	99.87666	44.267	99.80548	43.57	99.41441	46.33	99.33608
45.991	99.33944	99.86312	45.267	99.80155	44.57	99.38995	47.33	99.33019
46.991	99.33422	99.8434	46.267	99.80013	45.57	99.36906	48.33	99.31902
47.991	99.32109	99.82079	47.267	99.7928	46.57	99.34251	49.33	99.30976
48.991	99.31165	99.79719	48.267	99.7858	47.57	99.31322	50.33	99.30362
49.991	99.30326	99.7676	49.267	99.78057	48.57	99.28758	51.33	99.29548
50.991	99.29829	99.73678	50.267	99.77214	49.57	99.26138	52.33	99.29196
51.991	99.296	99.70705	51.267	99.76497	50.57	99.237	53.33	99.28971
52.991	99.29142	99.67453	52.267	99.75999	51.57	99.21508	54.33	99.28297
53.991	99.28451	99.63571	53.267	99.75278	52.57	99.19329	55.33	99.27764
54.991	99.28155	99.59549	54.267	99.74304	53.57	99.16692	56.33	99.27743
55.991	99.28221	99.55648	55.267	99.73681	54.57	99.14132	57.33	99.27692
56.991	99.28339	99.51589	56.267	99.73549	55.57	99.12068	58.33	99.27822
57.991	99.28717	99.47198	57.267	99.73371	56.57	99.1022	59.33	99.28101
58.991	99.28971	99.42979	58.267	99.7323	57.57	99.0833	60.33	99.27853
59.991	99.28793	99.38173	59.267	99.73452	58.57	99.06695	61.33	99.27278
60.991	99.28413	99.32814	60.267	99.73298	59.57	99.04831	62.33	99.26837
61.991	99.28283	99.26994	61.267	99.72754	60.57	99.02635	63.33	99.26728
62.991	99.2825	99.20986	62.267	99.7221	61.57	99.00265	64.33	99.26765
63.991	99.28292	99.15021	63.267	99.7172	62.57	98.98184	65.33	99.26805
64.991	99.28353	99.09065	64.267	99.71299	63.57	98.96209	66.33	99.26786

65.991	99.28153	99.02932	65.267	99.70865	64.57	98.94277	67.33	99.2634
66.991	99.2766	98.96882	66.267	99.70324	65.57	98.92305	68.33	99.25935
67.991	99.27195	98.9062	67.267	99.6968	66.57	98.90362	69.33	99.25596
68.991	99.26766	98.84362	68.267	99.68915	67.57	98.88193	70.33	99.25426
69.991	99.26394	98.78304	69.267	99.68252	68.57	98.85995	71.33	99.25253
70.991	99.25919	98.72693	70.267	99.67649	69.57	98.84042	72.33	99.2512
71.991	99.25468	98.67203	71.267	99.67173	70.57	98.81993	73.33	99.24856
72.991	99.2494	98.62272	72.267	99.66789	71.57	98.79979	74.33	99.24506
73.991	99.24403	98.5741	73.267	99.66521	72.57	98.78041	75.33	99.2399
74.991	99.23728	98.52574	74.267	99.6622	73.57	98.76171	76.33	99.2329
75.991	99.22977	98.47816	75.267	99.65795	74.57	98.74268	77.33	99.2277
76.991	99.22359	98.4278	76.267	99.65115	75.57	98.72221	78.33	99.22551
77.991	99.22081	98.37937	77.267	99.64326	76.57	98.6986	79.33	99.22742
78.991	99.21988	98.33062	78.267	99.63573	77.57	98.67656	80.33	99.2298
79.991	99.22056	98.283	79.267	99.63061	78.57	98.65632	81.33	99.2312
80.991	99.21975	98.23321	80.267	99.62755	79.57	98.63929	82.33	99.22963
81.991	99.21825	98.1787	81.267	99.62571	80.57	98.62415	83.33	99.22745
82.991	99.21618	98.11592	82.267	99.62239	81.57	98.61028	84.33	99.2258
83.991	99.21284	98.05139	83.267	99.61704	82.57	98.59323	85.33	99.22684
84.991	99.21247	97.98188	84.267	99.61154	83.57	98.57552	86.33	99.22926
85.991	99.21357	97.90852	85.267	99.60557	84.57	98.55899	87.33	99.2293
86.991	99.21388	97.83798	86.267	99.60258	85.57	98.54367	88.33	99.22818
87.991	99.21368	97.765	87.267	99.60099	86.57	98.52997	89.33	99.22541
88.991	99.21346	97.69377	88.267	99.59849	87.57	98.51546	90.33	99.22154
89.991	99.21425	97.62392	89.267	99.59533	88.57	98.49885	91.33	99.21825
90.991	99.21583	97.55534	90.267	99.59054	89.57	98.48201	92.33	99.21493
91.991	99.21527	97.49142	91.267	99.58434	90.57	98.46302	93.33	99.20974
92.991	99.2139	97.42947	92.267	99.57868	91.57	98.44348	94.33	99.2043
93.991	99.21364	97.36496	93.267	99.57158	92.57	98.42504	95.33	99.19826
94.991	99.21349	97.2994	94.267	99.56323	93.57	98.40507	96.33	99.1926
95.991	99.21329	97.23169	95.267	99.55726	94.57	98.38493	97.33	99.18566
96.991	99.21102	97.16139	96.267	99.55207	95.57	98.3625	98.33	99.17781
97.991	99.20895	97.0861	97.267	99.54771	96.57	98.34248	99.33	99.1707
98.991	99.20712	97.00203	98.267	99.54223	97.57	98.32279	100.33	99.16516
99.991	99.20494	96.91119	99.267	99.53653	98.57	98.3028	101.33	99.16139
100.991	99.20412	96.81526	100.267	99.53132	99.57	98.28477	102.33	99.15876
101.991	99.20396	96.71362	101.267	99.52601	100.57	98.26985	103.33	99.15481
102.991	99.20229	96.60924	102.267	99.52147	101.57	98.2561	104.33	99.15335
103.991	99.20296	96.50376	103.267	99.51801	102.57	98.24477	105.33	99.15283
104.991	99.20287	96.40259	104.267	99.51378	103.57	98.23287	106.33	99.15257
105.991	99.20271	96.30617	105.267	99.51141	104.57	98.22462	107.33	99.1525
106.991	99.20261	96.2186	106.267	99.5067	105.57	98.21997	108.33	99.1505
107.991	99.20021	96.13945	107.267	99.50155	106.57	98.21378	109.33	99.14794

108.991	99.19754	96.06945	108.267	99.49487	107.57	98.20844	110.33	99.14349
109.991	99.19332	96.01062	109.267	99.48793	108.57	98.20071	111.33	99.13861
110.991	99.18916	95.96195	110.267	99.48253	109.57	98.19105	112.33	99.1359
111.991	99.1878	95.92404	111.267	99.47647	110.57	98.17932	113.33	99.13251
112.991	99.18563	95.89787	112.267	99.4709	111.57	98.16554	114.33	99.12701
113.991	99.1823	95.87448	113.267	99.46592	112.57	98.15277	115.33	99.12125
114.991	99.17955	95.85361	114.267	99.45762	113.57	98.13902	116.33	99.11632
115.991	99.17735	95.83292	115.267	99.4489	114.57	98.12025	117.33	99.11351
116.991	99.17618	95.81385	116.267	99.44162	115.57	98.10147	118.33	99.10876
117.991	99.17398	95.79648	117.267	99.43733	116.57	98.08416	119.33	99.10397
118.991	99.17344	95.77643	118.267	99.43221	117.57	98.06998	120.33	99.10011
119.991	99.17442	95.75504	119.267	99.42509	118.57	98.05509	121.33	99.09596
120.991	99.1746	95.7315	120.267	99.41927	119.57	98.04042	122.33	99.08968
121.991	99.17201	95.70556	121.267	99.41363	120.57	98.0273	123.33	99.08343
122.991	99.1707	95.67545	122.267	99.40599	121.57	98.01492	124.33	99.07854
123.991	99.17051	95.64341	123.267	99.39695	122.57	98.00181	125.33	99.07336
124.991	99.16965	95.61089	124.267	99.38982	123.57	97.9908	126.33	99.06752
125.991	99.16775	95.57637	125.267	99.38189	124.57	97.98265	127.33	99.06353
126.991	99.1661	95.5391	126.267	99.3722	125.57	97.97396	128.33	99.06076
127.991	99.1638	95.50389	127.267	99.36312	126.57	97.96471	129.33	99.0583
128.991	99.16116	95.47188	128.267	99.3551	127.57	97.95781	130.33	99.05598
129.991	99.15891	95.43983	129.267	99.34718	128.57	97.95275	131.33	99.0537
130.991	99.15584	95.41087	130.267	99.33917	129.57	97.94818	132.33	99.04871
131.991	99.15032	95.38268	131.267	99.33364	130.57	97.94355	133.33	99.04377
132.991	99.14588	95.35577	132.267	99.32698	131.57	97.93913	134.33	99.03959
133.991	99.14315	95.33139	133.267	99.32106	132.57	97.93164	135.33	99.03666
134.991	99.14363	95.30877	134.267	99.31635	133.57	97.92234	136.33	99.03195
135.991	99.14278	95.28341	135.267	99.31516	134.57	97.91176	137.33	99.02341
136.991	99.13915	95.25211	136.267	99.31397	135.57	97.90156	138.33	99.01235
137.991	99.13383	95.21425	137.267	99.30838	136.57	97.8888	139.33	99.00128
138.991	99.12969	95.17197	138.267	99.29951	137.57	97.87414	140.33	98.99459
139.991	99.13073	95.1282	139.267	99.28955	138.57	97.85822	141.33	98.98996
140.991	99.1337	95.08258	140.267	99.28187	139.57	97.84116	142.33	98.98492
141.991	99.13549	95.03184	141.267	99.27583	140.57	97.82796	143.33	98.97721
142.991	99.13444	94.97651	142.267	99.26984	141.57	97.81627	144.33	98.96829
143.991	99.13095	94.91909	143.267	99.26135	142.57	97.80727	145.33	98.96185
144.991	99.12949	94.86133	144.267	99.25034	143.57	97.79752	146.33	98.95808
145.991	99.1305	94.80782	145.267	99.24128	144.57	97.78773	147.33	98.95554
146.991	99.1323	94.76008	146.267	99.23751	145.57	97.78129	148.33	98.95199
147.991	99.13242	94.71603	147.267	99.23735	146.57	97.77549	149.33	98.94555
148.991	99.13224	94.6754	148.267	99.2374	147.57	97.77114	150.33	98.93671
149.991	99.13172	94.64101	149.267	99.23618	148.57	97.76634	151.33	98.92796
150.991	99.13267	94.6114	150.267	99.2346	149.57	97.75986	152.33	98.9203

151.991	99.13494	94.58706	151.267	99.23346	150.57	97.75218	153.33	98.91295
152.991	99.13727	94.56903	152.267	99.23378	151.57	97.74469	154.33	98.90538
153.991	99.13913	94.55553	153.267	99.23417	152.57	97.73793	155.33	98.89652
154.991	99.13936	94.54358	154.267	99.23458	153.57	97.73088	156.33	98.88598
155.991	99.13786	94.5322	155.267	99.23259	154.57	97.72358	157.33	98.87322
156.991	99.13344	94.51995	156.267	99.22878	155.57	97.71518	158.33	98.86141
157.991	99.12731	94.50666	157.267	99.22274	156.57	97.70512	159.33	98.85085
158.991	99.12123	94.49385	158.267	99.21375	157.57	97.69389	160.33	98.84087
159.991	99.1161	94.48127	159.267	99.20348	158.57	97.68229	161.33	98.83085
160.991	99.11255	94.46807	160.267	99.19256	159.57	97.67126	162.33	98.82113
161.991	99.11095	94.45586	161.267	99.18196	160.57	97.66064	163.33	98.81048
162.991	99.11065	94.44525	162.267	99.17242	161.57	97.65049	164.33	98.80107
163.991	99.11193	94.43658	163.267	99.16377	162.57	97.64149	165.33	98.796
164.991	99.11517	94.42976	164.267	99.1564	163.57	97.63308	166.33	98.79374
165.991	99.11972	94.42622	165.267	99.15027	164.57	97.62565	167.33	98.78827
166.991	99.1231	94.4233	166.267	99.14665	165.57	97.61949	168.33	98.78046
167.991	99.12512	94.41961	167.267	99.14302	166.57	97.61445	169.33	98.77275
168.991	99.12611	94.41691	168.267	99.13879	167.57	97.60802	170.33	98.76591
169.991	99.12585	94.41403	169.267	99.13458	168.57	97.60038	171.33	98.76039
170.991	99.12576	94.40951	170.267	99.13039	169.57	97.59056	172.33	98.75655
171.991	99.12623	94.40348	171.267	99.12616	170.57	97.57922	173.33	98.75066
172.991	99.12665	94.39704	172.267	99.12135	171.57	97.56792	174.33	98.74175
173.991	99.1272	94.39029	173.267	99.11645	172.57	97.55671	175.33	98.73383
174.991	99.12854	94.38288	174.267	99.11174	173.57	97.54623	176.33	98.72829
175.991	99.1309	94.37626	175.267	99.10801	174.57	97.53671	177.33	98.72256
176.991	99.13352	94.37018	176.267	99.1058	175.57	97.52855	178.33	98.71642
177.991	99.13676	94.3644	177.267	99.105	176.57	97.52179	179.33	98.70987
178.991	99.13953	94.35993	178.267	99.10489	177.57	97.51594	180.33	98.7031
179.991	99.14159	94.35706	179.267	99.10489	178.57	97.51179	181.33	98.69595
180.991	99.14289	94.35461	180.267	99.10499	179.57	97.50732	182.33	98.68934
181.991	99.1436	94.35114	181.267	99.10518	180.57	97.50272	183.33	98.6843
182.991	99.1439	94.34615	182.267	99.10456	181.57	97.49801	184.33	98.67988
183.991	99.14354	94.33926	183.267	99.10297	182.57	97.49282	185.33	98.67501
184.991	99.14143	94.33074	184.267	99.10126	183.57	97.48768	186.33	98.66964
185.991	99.13843	94.31997	185.267	99.09735	184.57	97.48256	187.33	98.66383
186.991	99.13512	94.30674	186.267	99.0921	185.57	97.47618	188.33	98.65714
187.991	99.13106	94.29132	187.267	99.08811	186.57	97.46966	189.33	98.65114
188.991	99.12789	94.2734	188.267	99.08314	187.57	97.4622	190.33	98.6466
189.991	99.12625	94.25575	189.267	99.07818	188.57	97.45352	191.33	98.6422
190.991	99.12541	94.23881	190.267	99.075	189.57	97.44571	192.33	98.63799
191.991	99.12525	94.22188	191.267	99.07385	190.57	97.439	193.33	98.63471
192.991	99.12668	94.20388	192.267	99.07225	191.57	97.4329	194.33	98.63039
193.991	99.12739	94.18483	193.267	99.07072	192.57	97.42727	195.33	98.62604

194.991	99.12754	94.16242	194.267	99.06822	193.57	97.4227	196.33	98.62189
195.991	99.12743	94.13906	195.267	99.0632	194.57	97.41717	197.33	98.61675
196.991	99.1266	94.11323	196.267	99.05715	195.57	97.41214	198.33	98.61141
197.991	99.1257	94.08482	197.267	99.05075	196.57	97.40713	199.33	98.60557
198.991	99.12341	94.05433	198.267	99.04523	197.57	97.40236	200.33	98.59941
199.991	99.11961	94.02101	199.267	99.03939	198.57	97.3986	201.33	98.59095
200.991	99.11372	93.9871	200.267	99.03284	199.57	97.39413	202.33	98.58469
201.991	99.11117	93.95138	201.267	99.02561	200.57	97.38775	203.33	98.58137
202.991	99.1114	93.92227	202.267	99.02078	201.57	97.37914	204.33	98.57959
203.991	99.11311	93.89637	203.267	99.0209	202.57	97.37384	205.33	98.57977
204.991	99.11633	93.87467	204.267	99.02272	203.57	97.3708	206.33	98.5796
205.991	99.11948	93.85469	205.267	99.02701	204.57	97.36951	207.33	98.57976
206.991	99.12498	93.83771	206.267	99.02971	205.57	97.36928	208.33	98.5801
207.991	99.1321	93.82542	207.267	99.03177	206.57	97.36686	209.33	98.58147
208.991	99.14025	93.81497	208.267	99.03423	207.57	97.36289	210.33	98.58295
209.991	99.14912	93.80969	209.267	99.0373	208.57	97.35929	211.33	98.58223
210.991	99.15669	93.80305	210.267	99.04058	209.57	97.3571	212.33	98.58058
211.991	99.16294	93.7965	211.267	99.04032	210.57	97.35397	213.33	98.57869
212.991	99.16932	93.79089	212.267	99.04038	211.57	97.34862	214.33	98.57799
213.991	99.17538	93.78618	213.267	99.03966	212.57	97.34195	215.33	98.57733
214.991	99.17982	93.78148	214.267	99.03784	213.57	97.33453	216.33	98.57744
215.991	99.18696	93.77636	215.267	99.03546	214.57	97.32646	217.33	98.57995
216.991	99.196	93.7735	216.267	99.03319	215.57	97.31874	218.33	98.58339
217.991	99.20439	93.77167	217.267	99.03312	216.57	97.31398	219.33	98.58733
218.991	99.21139	93.77068	218.267	99.03468	217.57	97.31353	220.33	98.59163
219.991	99.22036	93.77024	219.267	99.03947	218.57	97.31601	221.33	98.5965
220.991	99.23048	93.77008	220.267	99.04512	219.57	97.32141	222.33	98.59902
221.991	99.23846	93.77	221.267	99.05296	220.57	97.32805	223.33	98.60002
222.991	99.24413	93.76722	222.267	99.06066	221.57	97.33578	224.33	98.60185
223.991	99.24889	93.76337	223.267	99.06614	222.57	97.34286	225.33	98.60339
224.991	99.25351	93.75778	224.267	99.07186	223.57	97.34929	226.33	98.60501
225.991	99.25966	93.75101	225.267	99.07757	224.57	97.35683	227.33	98.60631
226.991	99.26689	93.74292	226.267	99.08301	225.57	97.36375	228.33	98.60757
227.991	99.27335	93.73446	227.267	99.08574	226.57	97.36874	229.33	98.61012
228.991	99.27854	93.72452	228.267	99.08717	227.57	97.37209	230.33	98.61582
229.991	99.2855	93.71411	229.267	99.08712	228.57	97.37562	231.33	98.62417
230.991	99.29638	93.70703	230.267	99.08909	229.57	97.37961	232.33	98.63257
231.991	99.30812	93.70149	231.267	99.09546	230.57	97.38438	233.33	98.64018
232.991	99.31946	93.69754	232.267	99.10286	231.57	97.39119	234.33	98.64594
233.991	99.32945	93.69311	233.267	99.11039	232.57	97.39735	235.33	98.65282
234.991	99.3387	93.68865	234.267	99.11727	233.57	97.40103	236.33	98.66114
235.991	99.34762	93.68398	235.267	99.12411	234.57	97.40366	237.33	98.66699
236.991	99.35617	93.67872	236.267	99.13112	235.57	97.40561	238.33	98.67007

237.991	99.36582	93.67223	237.267	99.13725	236.57	97.40563	239.33	98.67372
238.991	99.37682	93.66545	238.267	99.1433	237.57	97.40315	240.33	98.67648
239.991	99.38742	93.65871	239.267	99.14935	238.57	97.40086	241.33	98.6818
240.991	99.39857	93.65289	240.267	99.15489	239.57	97.4001	242.33	98.69224
241.991	99.41206	93.64897	241.267	99.16043	240.57	97.40195	243.33	98.70161
242.991	99.42605	93.64706	242.267	99.16842	241.57	97.40602	244.33	98.7094
243.991	99.44074	93.64743	243.267	99.17854	242.57	97.41293	245.33	98.71793
244.991	99.45872	93.6515	244.267	99.18979	243.57	97.42241	246.33	98.72801
245.991	99.47843	93.65828	245.267	99.20238	244.57	97.43433	247.33	98.73956
246.991	99.49639	93.6668	246.267	99.21528	245.57	97.45054	248.33	98.75292
247.991	99.51544	93.67717	247.267	99.2297	246.57	97.4681	249.33	98.76544
248.991	99.53432	93.68878	248.267	99.24723	247.57	97.48603	250.33	98.77554
249.991	99.55206	93.70193	249.267	99.26507	248.57	97.50382	251.33	98.78576
250.991	99.5699	93.71636	250.267	99.28128	249.57	97.52076	252.33	98.79784
251.991	99.5898	93.73044	251.267	99.29623	250.57	97.53705	253.33	98.81234
252.991	99.60815	93.74384	252.267	99.31154	251.57	97.55293	254.33	98.82883
253.991	99.62551	93.75915	253.267	99.32903	252.57	97.56835	255.33	98.84358
254.991	99.64344	93.77699	254.267	99.34751	253.57	97.58259	256.33	98.85947
255.991	99.66092	93.79488	255.267	99.36571	254.57	97.59605	257.33	98.87513
256.991	99.67787	93.81387	256.267	99.38228	255.57	97.60877	258.33	98.89155
257.991	99.69442	93.83276	257.267	99.40086	256.57	97.62004	259.33	98.91066
258.991	99.71238	93.85183	258.267	99.42208	257.57	97.6304	260.33	98.92916
259.991	99.73088	93.87175	259.267	99.4443	258.57	97.64104	261.33	98.94738
260.991	99.74938	93.89297	260.267	99.46621	259.57	97.65214	262.33	98.96672
261.991	99.76849	93.9146	261.267	99.48832	260.57	97.66472	263.33	98.98621
262.991	99.78873	93.9364	262.267	99.51039	261.57	97.67777	264.33	99.00411
263.991	99.81067	93.95727	263.267	99.5322	262.57	97.69184	265.33	99.02184
264.991	99.83341	93.97867	264.267	99.55401	263.57	97.70747	266.33	99.04013
265.991	99.85756	94.00007	265.267	99.57435	264.57	97.72536	267.33	99.05847
266.991	99.88217	94.0223	266.267	99.59454	265.57	97.74389	268.33	99.08128
267.991	99.90545	94.04623	267.267	99.61477	266.57	97.76348	269.33	99.10545
268.991	99.92905	94.0733	268.267	99.63552	267.57	97.78213	270.33	99.12795
269.991	99.95314	94.10089	269.267	99.65564	268.57	97.80089	271.33	99.14754
270.991	99.97716	94.12816	270.267	99.67637	269.57	97.8201	272.33	99.16779
271.991	100.00066	94.15568	271.267	99.69789	270.57	97.84072	273.33	99.189
272.991	100.0225	94.18185	272.267	99.71951	271.57	97.86207	274.33	99.21075
273.991	100.04595	94.2055	273.267	99.74144	272.57	97.88285	275.33	99.23143
274.991	100.06964	94.22859	274.267	99.76372	273.57	97.90459	276.33	99.24903
275.991	100.09363	94.24874	275.267	99.78612	274.57	97.92706	277.33	99.26487
276.991	100.11821	94.26685	276.267	99.80924	275.57	97.95073	278.33	99.28295
277.991	100.14512	94.28476	277.267	99.83354	276.57	97.97458	279.33	99.3054
278.991	100.17328	94.3024	278.267	99.85765	277.57	97.99846	280.33	99.33002
279.991	100.20033	94.32041	279.267	99.88166	278.57	98.023	281.33	99.35396

280.991	100.23116	94.34145	280.267	99.90676	279.57	98.0477	282.33	99.37945
281.991	100.26185	94.36533	281.267	99.93421	280.57	98.07229	283.33	99.40779
282.991	100.29345	94.39207	282.267	99.96387	281.57	98.09795	284.33	99.43793
283.991	100.32812	94.41989	283.267	99.99372	282.57	98.12515	285.33	99.46833
284.991	100.36259	94.44918	284.267	100.02536	283.57	98.15189	286.33	99.49922
285.991	100.39431	94.478	285.267	100.0578	284.57	98.17861	287.33	99.52928
286.991	100.42772	94.50485	286.267	100.09173	285.57	98.2054	288.33	99.5599
287.991	100.46256	94.53263	287.267	100.12565	286.57	98.23041	289.33	99.59259
288.991	100.4954	94.56073	288.267	100.16049	287.57	98.25523	290.33	99.62436
289.991	100.52968	94.58853	289.267	100.19634	288.57	98.28038	291.33	99.65581
290.991	100.56383	94.61547	290.267	100.23105	289.57	98.30535	292.33	99.68793
291.991	100.59807	94.64304	291.267	100.2663	290.57	98.33083	293.33	99.71997
292.991	100.63244	94.66868	292.267	100.30193	291.57	98.35832	294.33	99.75322
293.991	100.66838	94.69381	293.267	100.33691	292.57	98.38754	295.33	99.787
294.991	100.70226	94.72107	294.267	100.37132	293.57	98.41937	296.33	99.81959
295.991	100.73782	94.74912	295.267	100.40659	294.57	98.45313	297.33	99.85112
296.991	100.77368	94.77594	296.267	100.44229	295.57	98.48884	298.33	99.88268
297.991	100.80867	94.80112	297.267	100.47667	296.57	98.52573	299.33	99.91598
298.991	100.84237	94.8263	298.267	100.51085	297.57	98.56216	300.33	99.94701
299.991	100.87638	94.85046	299.267	100.54668	298.57	98.59858	301.33	99.97925
300.991	100.91161	94.87814	300.267	100.5823	299.57	98.63378	302.33	100.01166
301.991	100.94549	94.9059	301.267	100.61885	300.57	98.66885	303.33	100.04497
302.991	100.98162	94.93418	302.267	100.65523	301.57	98.70324	304.33	100.07864
303.991	101.01882	94.96302	303.267	100.69262	302.57	98.73766	305.33	100.11428
304.991	101.05576	94.99157	304.267	100.73142	303.57	98.77337	306.33	100.1495
305.991	101.09091	95.01853	305.267	100.76867	304.57	98.80943	307.33	100.1806
306.991	101.12625	95.04431	306.267	100.80541	305.57	98.84447	308.33	100.21278
307.991	101.16022	95.06781	307.267	100.84042	306.57	98.878	309.33	100.24596
308.991	101.19427	95.08867	308.267	100.87311	307.57	98.91126	310.33	100.27895
309.991	101.22863	95.10976	309.267	100.9055	308.57	98.94335	311.33	100.30933
310.991	101.26283	95.12972	310.267	100.93789	309.57	98.97321	312.33	100.33926
311.991	101.2938	95.14766	311.267	100.96932	310.57	99.00311	313.33	100.36653
312.991	101.32508	95.16405	312.267	101.00057	311.57	99.02972	314.33	100.3945
313.991	101.35573	95.18192	313.267	101.03077	312.57	99.05575	315.33	100.42242
314.991	101.38416	95.20118	314.267	101.06222	313.57	99.08258	316.33	100.44845
315.991	101.41394	95.22118	315.267	101.09223	314.57	99.11065	317.33	100.47419
316.991	101.44392	95.24167	316.267	101.12214	315.57	99.13926	318.33	100.49863
317.991	101.47319	95.26157	317.267	101.15269	316.57	99.17005	319.33	100.52591
318.991	101.50128	95.28076	318.267	101.18422	317.57	99.20224	320.33	100.55324
319.991	101.52831	95.30057	319.267	101.21552	318.57	99.23477	321.33	100.58205
320.991	101.55403	95.32055	320.267	101.24666	319.57	99.26727	322.33	100.61062
321.991	101.58274	95.33844	321.267	101.275	320.57	99.30004	323.33	100.64115
322.991	101.61118	95.35394	322.267	101.30263	321.57	99.33257	324.33	100.67025

323.991	101.63861	95.36806	323.267	101.33266	322.57	99.36393	325.33	100.69842
324.991	101.66083	95.37897	324.267	101.36123	323.57	99.39248	326.33	100.72733
325.991	101.68209	95.38731	325.267	101.3857	324.57	99.41905	327.33	100.75118
326.991	101.70238	95.39363	326.267	101.40534	325.57	99.44445	328.33	100.77315
327.991	101.72178	95.39887	327.267	101.42261	326.57	99.46794	329.33	100.79242
328.991	101.73886	95.40418	328.267	101.43829	327.57	99.4903	330.33	100.80937
329.991	101.75232	95.40767	329.267	101.45329	328.57	99.51111	331.33	100.82659
330.991	101.76465	95.41034	330.267	101.46612	329.57	99.52924	332.33	100.84242
331.991	101.77518	95.41039	331.267	101.4752	330.57	99.54451	333.33	100.85532
332.991	101.78549	95.40882	332.267	101.48293	331.57	99.55977	334.33	100.86551
333.991	101.79344	95.40469	333.267	101.49087	332.57	99.57451	335.33	100.87616
334.991	101.79902	95.39886	334.267	101.49979	333.57	99.58772	336.33	100.88627
335.991	101.80288	95.39096	335.267	101.50851	334.57	99.59986	337.33	100.89504
336.991	101.80645	95.38035	336.267	101.51517	335.57	99.60957	338.33	100.90245
337.991	101.80944	95.37004	337.267	101.52101	336.57	99.61708	339.33	100.90643
338.991	101.81004	95.35761	338.267	101.52535	337.57	99.62497	340.33	100.90993
339.991	101.80628	95.34489	339.267	101.52651	338.57	99.63304	341.33	100.91297
340.991	101.80006	95.33033	340.267	101.52558	339.57	99.63872	342.33	100.91487
341.991	101.79145	95.31665	341.267	101.5211	340.57	99.64275	343.33	100.91516
342.991	101.78105	95.30106	342.267	101.51219	341.57	99.64452	344.33	100.91331
343.991	101.76805	95.28355	343.267	101.50038	342.57	99.64551	345.33	100.91116
344.991	101.75305	95.26328	344.267	101.48644	343.57	99.64666	346.33	100.90821
345.991	101.7343	95.24066	345.267	101.47026	344.57	99.64608	347.33	100.9037
346.991	101.71462	95.21621	346.267	101.45169	345.57	99.64243	348.33	100.89668
347.991	101.69559	95.19145	347.267	101.43233	346.57	99.6358	349.33	100.88789
348.991	101.67453	95.16658	348.267	101.41127	347.57	99.62667	350.33	100.87711
349.991	101.65093	95.14041	349.267	101.38845	348.57	99.61491	351.33	100.86348
350.991	101.62472	95.11355	350.267	101.36402	349.57	99.5999	352.33	100.84923
351.991	101.59644	95.08374	351.267	101.3347	350.57	99.58213	353.33	100.83129
352.991	101.56602	95.05203	352.267	101.3021	351.57	99.56139	354.33	100.81194
353.991	101.53455	95.01802	353.267	101.26675	352.57	99.53891	355.33	100.79246
354.991	101.50175	94.98502	354.267	101.22782	353.57	99.51625	356.33	100.77122
355.991	101.46532	94.94855	355.267	101.18778	354.57	99.49258	357.33	100.74606
356.991	101.42856	94.90785	356.267	101.1463	355.57	99.47035	358.33	100.71835
357.991	101.39041	94.86519	357.267	101.10356	356.57	99.44564	359.33	100.68878
358.991	101.34976	94.81997	358.267	101.05898	357.57	99.42097	360.33	100.65791
359.991	101.30752	94.77349	359.267	101.01376	358.57	99.39421	361.33	100.62609
360.991	101.26188	94.72484	360.267	100.96691	359.57	99.36416	362.33	100.59261
361.991	101.21368	94.67266	361.267	100.91601	360.57	99.33292	363.33	100.55596
362.991	101.1614	94.61722	362.267	100.86571	361.57	99.29942	364.33	100.51572
363.991	101.10644	94.56042	363.267	100.81064	362.57	99.26323	365.33	100.47489
364.991	101.04805	94.50227	364.267	100.75462	363.57	99.22486	366.33	100.43377
365.991	100.98628	94.4422	365.267	100.6956	364.57	99.18354	367.33	100.39251

366.991	100.92293	94.3801	366.267	100.63357	365.57	99.13908	368.33	100.34789
367.991	100.8577	94.31661	367.267	100.57002	366.57	99.09226	369.33	100.29988
368.991	100.79144	94.25329	368.267	100.50414	367.57	99.04519	370.33	100.24956
369.991	100.72451	94.18867	369.267	100.4387	368.57	98.99597	371.33	100.19668
370.991	100.65646	94.12227	370.267	100.36871	369.57	98.94389	372.33	100.1427
371.991	100.58342	94.05438	371.267	100.29853	370.57	98.88795	373.33	100.08513
372.991	100.50799	93.98285	372.267	100.22332	371.57	98.82739	374.33	100.02532
373.991	100.43224	93.90862	373.267	100.14653	372.57	98.76777	375.33	99.9642
374.991	100.35453	93.83406	374.267	100.06861	373.57	98.7061	376.33	99.89974
375.991	100.27431	93.76008	375.267	99.98888	374.57	98.64345	377.33	99.83729
376.991	100.19353	93.68242	376.267	99.90559	375.57	98.57869	378.33	99.7726
377.991	100.10968	93.60291	377.267	99.82162	376.57	98.51182	379.33	99.70854
378.991	100.02257	93.52304	378.267	99.73639	377.57	98.44624	380.33	99.64165
379.991	99.93443	93.43706	379.267	99.65111	378.57	98.38228	381.33	99.57083
380.991	99.84365	93.34881	380.267	99.562	379.57	98.31607	382.33	99.50116
381.991	99.75483	93.26126	381.267	99.46745	380.57	98.24664	383.33	99.42753
382.991	99.66569	93.1747	382.267	99.37346	381.57	98.17355	384.33	99.35544
383.991	99.57639	93.08907	383.267	99.28169	382.57	98.10363	385.33	99.28283
384.991	99.48868	92.99885	384.267	99.19073	383.57	98.03459	386.33	99.20909
385.991	99.39789	92.90885	385.267	99.09576	384.57	97.96483	387.33	99.13338
386.991	99.30718	92.81882	386.267	99.00282	385.57	97.89311	388.33	99.05698
387.991	99.21705	92.7286	387.267	98.90669	386.57	97.82027	389.33	98.98568
388.991	99.13034	92.63925	388.267	98.81128	387.57	97.74178	390.33	98.91229
389.991	99.03931	92.54942	389.267	98.71646	388.57	97.66565	391.33	98.8392
390.991	98.94783	92.45673	390.267	98.61956	389.57	97.58735	392.33	98.76495
391.991	98.85497	92.36217	391.267	98.52044	390.57	97.50521	393.33	98.68783
392.991	98.76106	92.26878	392.267	98.42153	391.57	97.42302	394.33	98.60832
393.991	98.66543	92.17401	393.267	98.32148	392.57	97.33923	395.33	98.52972
394.991	98.56966	92.07545	394.267	98.22181	393.57	97.25545	396.33	98.45051
395.991	98.47682	91.97991	395.267	98.12154	394.57	97.17344	397.33	98.37158
396.991	98.38165	91.88139	396.267	98.02336	395.57	97.0929	398.33	98.2907
397.991	98.28704	91.78349	397.267	97.92588	396.57	97.01474	399.33	98.20652
398.991	98.18925	91.68292	398.267	97.82653	397.57	96.93645	400.33	98.12052
399.991	98.08809	91.58131	399.267	97.72672	398.57	96.85767	401.33	98.0356
400.991	97.9831	91.47298	400.267	97.62716	399.57	96.77758	402.33	97.94757
401.991	97.87888	91.36383	401.267	97.52618	400.57	96.69363	403.33	97.85924
402.991	97.77357	91.24972	402.267	97.42233	401.57	96.60466	404.33	97.76932
403.991	97.66768	91.13127	403.267	97.31351	402.57	96.51274	405.33	97.67996
404.991	97.55889	91.00796	404.267	97.20371	403.57	96.41739	406.33	97.58964
405.991	97.4503	90.88046	405.267	97.09036	404.57	96.32013	407.33	97.49995
406.991	97.33893	90.74822	406.267	96.97812	405.57	96.22309	408.33	97.40876
407.991	97.22982	90.6107	407.267	96.86287	406.57	96.12385	409.33	97.31532
408.991	97.11764	90.46879	408.267	96.74302	407.57	96.02356	410.33	97.21998

409.991	97.00316	90.32753	409.267	96.61916	408.57	95.92065	411.33	97.12446
410.991	96.88534	90.18373	410.267	96.497	409.57	95.8183	412.33	97.02153
411.991	96.76466	90.03781	411.267	96.37323	410.57	95.71541	413.33	96.91824
412.991	96.64156	89.88693	412.267	96.2523	411.57	95.61204	414.33	96.81108
413.991	96.51703	89.72862	413.267	96.12895	412.57	95.50759	415.33	96.69947
414.991	96.38888	89.56186	414.267	96.002	413.57	95.3983	416.33	96.58471
415.991	96.25864	89.39021	415.267	95.8755	414.57	95.28747	417.33	96.46847
416.991	96.12394	89.21258	416.267	95.74322	415.57	95.17317	418.33	96.35148
417.991	95.98975	89.02687	417.267	95.60982	416.57	95.06027	419.33	96.23443
418.991	95.8547	88.8355	418.267	95.47751	417.57	94.9433	420.33	96.11545
419.991	95.71633	88.63372	419.267	95.34282	418.57	94.82714	421.33	95.99019
420.991	95.57267	88.42323	420.267	95.20454	419.57	94.70813	422.33	95.8643
421.991	95.4253	88.2103	421.267	95.05981	420.57	94.58888	423.33	95.73806
422.991	95.27763	87.9879	422.267	94.91275	421.57	94.46445	424.33	95.61248
423.991	95.12467	87.76402	423.267	94.76286	422.57	94.33785	425.33	95.4827
424.991	94.97545	87.53855	424.267	94.61159	423.57	94.20986	426.33	95.35285
425.991	94.81532	87.30885	425.267	94.45857	424.57	94.07733	427.33	95.21744
426.991	94.65427	87.07764	426.267	94.30266	425.57	93.94378	428.33	95.08135
427.991	94.49481	86.85221	427.267	94.1418	426.57	93.80757	429.33	94.94467
428.991	94.33415	86.61919	428.267	93.98024	427.57	93.66978	430.33	94.8064
429.991	94.16906	86.39629	429.267	93.81531	428.57	93.53012	431.33	94.66536
430.991	93.99585	86.17309	430.267	93.64276	429.57	93.39258	432.33	94.52402
431.991	93.82222	85.95138	431.267	93.47003	430.57	93.24747	433.33	94.38024
432.991	93.64567	85.73067	432.267	93.29771	431.57	93.10049	434.33	94.23394
433.991	93.46616	85.50644	433.267	93.12095	432.57	92.95159	435.33	94.08439
434.991	93.28072	85.2858	434.267	92.94436	433.57	92.80024	436.33	93.93297
435.991	93.08886	85.05871	435.267	92.76532	434.57	92.64256	437.33	93.77395
436.991	92.89211	84.82304	436.267	92.57889	435.57	92.47867	438.33	93.61041
437.991	92.68986	84.58778	437.267	92.38642	436.57	92.31054	439.33	93.44022
438.991	92.4865	84.34385	438.267	92.19262	437.57	92.13841	440.33	93.2654
439.991	92.28181	84.09751	439.267	91.98938	438.57	91.96146	441.33	93.08403
440.991	92.06711	83.84482	440.267	91.78078	439.57	91.78088	442.33	92.90179
441.991	91.84982	83.58525	441.267	91.56479	440.57	91.59797	443.33	92.71464
442.991	91.62548	83.32392	442.267	91.33813	441.57	91.40891	444.33	92.52496
443.991	91.39927	83.05713	443.267	91.10857	442.57	91.21989	445.33	92.33102
444.991	91.168	82.78509	444.267	90.87422	443.57	91.02512	446.33	92.13446
445.991	90.92787	82.50861	445.267	90.63913	444.57	90.82705	447.33	91.93223
446.991	90.68004	82.22153	446.267	90.39285	445.57	90.62512	448.33	91.72834
447.991	90.42387	81.93051	447.267	90.15048	446.57	90.41797	449.33	91.51521
448.991	90.165	81.6392	448.267	89.8984	447.57	90.20612	450.33	91.29261
449.991	89.89409	81.33503	449.267	89.6394	448.57	89.99221	451.33	91.06262
450.991	89.62104	81.02186	450.267	89.37248	449.57	89.76345	452.33	90.82388
451.991	89.34046	80.6904	451.267	89.09708	450.57	89.52695	453.33	90.58103

452.991	89.04665	80.36283	452.267	88.8133	451.57	89.28648	454.33	90.33597
453.991	88.74935	80.03036	453.267	88.52127	452.57	89.03913	455.33	90.07662
454.991	88.44123	79.69128	454.267	88.22411	453.57	88.78903	456.33	89.81551
455.991	88.12981	79.34095	455.267	87.91125	454.57	88.52371	457.33	89.55094
456.991	87.81092	78.98722	456.267	87.5899	455.57	88.24869	458.33	89.27353
457.991	87.47474	78.62857	457.267	87.27145	456.57	87.96541	459.33	88.98692
458.991	87.1393	78.25959	458.267	86.93923	457.57	87.67051	460.33	88.69502
459.991	86.79324	77.88242	459.267	86.60392	458.57	87.37529	461.33	88.39411
460.991	86.43691	77.49569	460.267	86.2627	459.57	87.07261	462.33	88.09056
461.991	86.06965	77.0995	461.267	85.90734	460.57	86.76132	463.33	87.77462
462.991	85.69783	76.69383	462.267	85.54545	461.57	86.43855	464.33	87.44799
463.991	85.31848	76.28068	463.267	85.17698	462.57	86.11261	465.33	87.11114
464.991	84.92192	75.86022	464.267	84.79945	463.57	85.78183	466.33	86.76751
465.991	84.51029	75.42042	465.267	84.4037	464.57	85.44553	467.33	86.40954
466.991	84.08555	74.97337	466.267	84.00694	465.57	85.09507	468.33	86.04918
467.991	83.64163	74.51428	467.267	83.58582	466.57	84.73828	469.33	85.67799
468.991	83.19519	74.0464	468.267	83.16173	467.57	84.37305	470.33	85.29314
469.991	82.73519	73.57452	469.267	82.72155	468.57	83.99525	471.33	84.90018
470.991	82.25298	73.08293	470.267	82.27501	469.57	83.60332	472.33	84.50294
471.991	81.75851	72.57983	471.267	81.79981	470.57	83.2086	473.33	84.0898
472.991	81.25465	72.06505	472.267	81.31903	471.57	82.78889	474.33	83.66335
473.991	80.72769	71.54919	473.267	80.82111	472.57	82.36847	475.33	83.22268
474.991	80.19572	71.01021	474.267	80.30978	473.57	81.9322	476.33	82.77019
475.991	79.63501	70.46926	475.267	79.78664	474.57	81.48833	477.33	82.29954
476.991	79.07407	69.90215	476.267	79.2443	475.57	81.02408	478.33	81.81009
477.991	78.49283	69.31575	477.267	78.686	476.57	80.55664	479.33	81.31449
478.991	77.89646	68.74181	478.267	78.11597	477.57	80.051	480.33	80.79535
479.991	77.29179	68.14598	479.267	77.54054	478.57	79.54891	481.33	80.26693
480.991	76.67423	67.53495	480.267	76.95082	479.57	79.03504	482.33	79.72704
481.991	76.03493	66.91397	481.267	76.33972	480.57	78.50101	483.33	79.16026
482.991	75.3819	66.28901	482.267	75.71906	481.57	77.94805	484.33	78.58829
483.991	74.71043	65.65232	483.267	75.09153	482.57	77.39711	485.33	78.00325
484.991	74.02598	64.99572	484.267	74.43141	483.57	76.81333	486.33	77.39562
485.991	73.31985	64.34865	485.267	73.76969	484.57	76.22249	487.33	76.79337
486.991	72.6104	63.66753	486.267	73.08185	485.57	75.61617	488.33	76.17484
487.991	71.8799	62.98818	487.267	72.38836	486.57	74.99529	489.33	75.54438
488.991	71.13279	62.29856	488.267	71.67381	487.57	74.3734	490.33	74.88927
489.991	70.3715	61.60004	489.267	70.94254	488.57	73.73146	491.33	74.22495
490.991	69.59194	60.88594	490.267	70.20974	489.57	73.07846	492.33	73.53934
491.991	68.80215	60.17912	491.267	69.45179	490.57	72.40261	493.33	72.85029
492.991	68.00259	59.46603	492.267	68.68103	491.57	71.73797	494.33	72.15689
493.991	67.18402	58.72791	493.267	67.91213	492.57	71.03638	495.33	71.45024
494.991	66.36056	57.99808	494.267	67.1367	493.57	70.33431	496.33	70.72401

495.991	65.52644	57.24946	495.267	66.32801	494.57	69.62231	497.33	69.99128
496.991	64.66276	56.50395	496.267	65.51234	495.57	68.89744	498.33	69.24295
497.991	63.80475	55.7483	497.267	64.69226	496.57	68.16824	499.33	68.49913
498.991	62.93984	54.97428	498.267	63.85441	497.57	67.41444	500.33	67.73872
499.991	62.06007	54.20817	499.267	63.01669	498.57	66.67778	501.33	66.96333
500.991	61.18486	53.43931	500.267	62.17164	499.57	65.92805	502.33	66.19656
501.991	60.2842	52.65713	501.267	61.30884	500.57	65.15744	503.33	65.40907
502.991	59.39719	51.88598	502.267	60.45099	501.57	64.39956	504.33	64.6225
503.991	58.48264	51.10517	503.267	59.57653	502.57	63.63377	505.33	63.82749
504.991	57.57682	50.31959	504.267	58.69415	503.57	62.85088	506.33	63.02305
505.991	56.66039	49.54307	505.267	57.81229	504.57	62.07175	507.33	62.214
506.991	55.74884	48.76005	506.267	56.91549	505.57	61.28238	508.33	61.41253
507.991	54.81368	47.97037	507.267	56.01571	506.57	60.49227	509.33	60.60762
508.991	53.8952	47.18582	508.267	55.11334	507.57	59.69726	510.33	59.78965
509.991	52.98764	46.40977	509.267	54.22314	508.57	58.90374	511.33	58.97916
510.991	52.07611	45.63805	510.267	53.32544	509.57	58.10497	512.33	58.17218
511.991	51.15837	44.87583	511.267	52.43324	510.57	57.31213	513.33	57.35283
512.991	50.25395	44.1071	512.267	51.53391	511.57	56.51599	514.33	56.54687
513.991	49.3521	43.36383	513.267	50.64251	512.57	55.70696	515.33	55.73776
514.991	48.45394	42.61274	514.267	49.77269	513.57	54.91879	516.33	54.92685
515.991	47.58585	41.87951	515.267	48.87381	514.57	54.13077	517.33	54.13087
516.991	46.71489	41.15639	516.267	48.01741	515.57	53.33725	518.33	53.33427
517.991	45.84658	40.44052	517.267	47.16037	516.57	52.55728	519.33	52.53669
518.991	44.99344	39.72563	518.267	46.31123	517.57	51.77634	520.33	51.76294
519.991	44.17314	39.03717	519.267	45.4513	518.57	50.99439	521.33	50.98813
520.991	43.34638	38.36241	520.267	44.62013	519.57	50.23701	522.33	50.22473
521.991	42.53667	37.70144	521.267	43.80302	520.57	49.47291	523.33	49.47995
522.991	41.74366	37.04667	522.267	43.00314	521.57	48.72736	524.33	48.73234
523.991	40.96059	36.41434	523.267	42.20561	522.57	47.98837	525.33	47.99756
524.991	40.19318	35.79244	524.267	41.43216	523.57	47.26188	526.33	47.26402
525.991	39.44416	35.19333	525.267	40.66162	524.57	46.54512	527.33	46.56458
526.991	38.71778	34.61103	526.267	39.92013	525.57	45.84038	528.33	45.84123
527.991	37.98948	34.0233	527.267	39.17672	526.57	45.15825	529.33	45.13847
528.991	37.27854	33.46308	528.267	38.46879	527.57	44.48553	530.33	44.44157
529.991	36.58978	32.92563	529.267	37.7538	528.57	43.80488	531.33	43.76793
530.991	35.92782	32.3825	530.267	37.08	529.57	43.14591	532.33	43.09765
531.991	35.28019	31.86412	531.267	36.41029	530.57	42.49887	533.33	42.4482
532.991	34.64543	31.35178	532.267	35.75741	531.57	41.8638	534.33	41.80016
533.991	34.02149	30.84209	533.267	35.11842	532.57	41.23239	535.33	41.15283
534.991	33.42677	30.34244	534.267	34.49949	533.57	40.61928	536.33	40.52362
535.991	32.82991	29.86257	535.267	33.88858	534.57	40.00395	537.33	39.90801
536.991	32.25026	29.38876	536.267	33.30198	535.57	39.40589	538.33	39.29075
537.991	31.69476	28.92986	537.267	32.72459	536.57	38.81267	539.33	38.69191

538.991	31.13765	28.48036	538.267	32.16099	537.57	38.23268	540.33	38.0867
539.991	30.6041	28.0329	539.267	31.60885	538.57	37.66946	541.33	37.4962
540.991	30.07296	27.60705	540.267	31.06423	539.57	37.0968	542.33	36.91155
541.991	29.55836	27.18664	541.267	30.5393	540.57	36.53898	543.33	36.34828
542.991	29.04439	26.77697	542.267	30.02605	541.57	35.97717	544.33	35.77349
543.991	28.54367	26.37417	543.267	29.51151	542.57	35.44352	545.33	35.21514
544.991	28.04982	25.98665	544.267	29.01506	543.57	34.90468	546.33	34.67161
545.991	27.56463	25.60154	545.267	28.52718	544.57	34.38298	547.33	34.12952
546.991	27.0904	25.22375	546.267	28.05045	545.57	33.86168	548.33	33.59495
547.991	26.62785	24.85733	547.267	27.57964	546.57	33.33863	549.33	33.06732
548.991	26.17989	24.49288	548.267	27.11647	547.57	32.83685	550.33	32.54704
549.991	25.73832	24.14127	549.267	26.66524	548.57	32.33793	551.33	32.03455
550.991	25.30334	23.79183	550.267	26.23024	549.57	31.84161	552.33	31.53385
551.991	24.88079	23.4493	551.267	25.79382	550.57	31.36372	553.33	31.02739
552.991	24.46997	23.11482	552.267	25.3721	551.57	30.88437	554.33	30.52901
553.991	24.05995	22.78658	553.267	24.95159	552.57	30.41111	555.33	30.04286
554.991	23.66061	22.45935	554.267	24.54408	553.57	29.95379	556.33	29.55219
555.991	23.26577	22.14602	555.267	24.13892	554.57	29.49846	557.33	29.07741
556.991	22.87338	21.83099	556.267	23.75294	555.57	29.04428	558.33	28.60267
557.991	22.49836	21.53486	557.267	23.37572	556.57	28.59696	559.33	28.13787
558.991	22.12688	21.23618	558.267	22.99455	557.57	28.15552	560.33	27.67704
559.991	21.76456	20.9466	559.267	22.63043	558.57	27.71397	561.33	27.23647
560.991	21.4087	20.65664	560.267	22.2627	559.57	27.28461	562.33	26.79597
561.991	21.05499	20.38035	561.267	21.91092	560.57	26.85805	563.33	26.35977
562.991	20.72096	20.11639	562.267	21.56816	561.57	26.43137	564.33	25.92872
563.991	20.38768	19.84968	563.267	21.22981	562.57	26.02168	565.33	25.50263
564.991	20.06647	19.58569	564.267	20.89354	563.57	25.62065	566.33	25.09009
565.991	19.7509	19.3286	565.267	20.56538	564.57	25.21872	567.33	24.68832
566.991	19.4518	19.08082	566.267	20.25395	565.57	24.83006	568.33	24.29348
567.991	19.16092	18.8426	567.267	19.94698	566.57	24.45176	569.33	23.89983
568.991	18.87831	18.61241	568.267	19.65024	567.57	24.07897	570.33	23.51223
569.991	18.6013	18.39414	569.267	19.362	568.57	23.71792	571.33	23.14727
570.991	18.33443	18.17678	570.267	19.08625	569.57	23.36774	572.33	22.78951
571.991	18.07894	17.97073	571.267	18.8224	570.57	23.01765	573.33	22.43942
572.991	17.83507	17.77201	572.267	18.56217	571.57	22.68466	574.33	22.09421
573.991	17.59694	17.5812	573.267	18.31306	572.57	22.35594	575.33	21.76169
574.991	17.3751	17.39944	574.267	18.07205	573.57	22.03888	576.33	21.44566
575.991	17.15592	17.22213	575.267	17.84038	574.57	21.73431	577.33	21.1375
576.991	16.94373	17.05528	576.267	17.6232	575.57	21.42931	578.33	20.83934
577.991	16.75347	16.89042	577.267	17.41573	576.57	21.14035	579.33	20.55244
578.991	16.56862	16.74164	578.267	17.21712	577.57	20.85485	580.33	20.27604
579.991	16.39503	16.59994	579.267	17.02655	578.57	20.58385	581.33	20.01345
580.991	16.23406	16.46851	580.267	16.84653	579.57	20.32717	582.33	19.76413

581.991	16.079	16.34618	581.267	16.68215	580.57	20.07946	583.33	19.51402
582.991	15.92772	16.2266	582.267	16.5283	581.57	19.8421	584.33	19.27571
583.991	15.78896	16.11319	583.267	16.37582	582.57	19.61682	585.33	19.05547
584.991	15.66755	16.01157	584.267	16.23115	583.57	19.3971	586.33	18.84406
585.991	15.54706	15.91814	585.267	16.10119	584.57	19.19473	587.33	18.64495
586.991	15.43345	15.82711	586.267	15.9786	585.57	19.00458	588.33	18.45634
587.991	15.32756	15.74627	587.267	15.86533	586.57	18.82203	589.33	18.27387
588.991	15.22542	15.66754	588.267	15.76087	587.57	18.65043	590.33	18.09981
589.991	15.13238	15.59277	589.267	15.65995	588.57	18.48832	591.33	17.94344
590.991	15.05044	15.52784	590.267	15.56865	589.57	18.32787	592.33	17.79128
591.991	14.9716	15.46793	591.267	15.4903	590.57	18.17469	593.33	17.64414
592.991	14.90043	15.41104	592.267	15.41611	591.57	18.03731	594.33	17.50398
593.991	14.83538	15.36133	593.267	15.35057	592.57	17.9019	595.33	17.36362
594.991	14.77087	15.31077	594.267	15.28779	593.57	17.77382	596.33	17.23426
595.991	14.71579	15.2622	595.267	15.22585	594.57	17.65135	597.33	17.1154
596.991	14.6671	15.22436	596.267	15.17331	595.57	17.53117	598.33	17.00289
597.991	14.62217	15.18839	597.267	15.13126	596.57	17.42236	599.33	16.89596
598.991	14.58092	15.15393	598.267	15.09052	597.57	17.3259	600.33	16.79611
599.991	14.54307	15.12081	599.267	15.05107	598.57	17.23475	601.33	16.69723
600.991	14.50538	15.09086	600.267	15.01385	599.57	17.14659	602.33	16.60987
601.991	14.47445	15.06368	601.267	14.97796	600.57	17.06151	603.33	16.53144
602.991	14.45035	15.04529	602.267	14.94877	601.57	16.97885	604.33	16.4567
603.991	14.42705	15.03099	603.267	14.92777	602.57	16.90537	605.33	16.38928
604.991	14.4059	15.0107	604.267	14.90729	603.57	16.84046	606.33	16.32296
605.991	14.38438	14.99338	605.267	14.88611	604.57	16.7759	607.33	16.25985
606.991	14.36446	14.97836	606.267	14.86702	605.57	16.71511	608.33	16.1996
607.991	14.3482	14.96724	607.267	14.85006	606.57	16.65502	609.33	16.1445
608.991	14.33201	14.95529	608.267	14.83595	607.57	16.5983	610.33	16.08705
609.991	14.31379	14.94135	609.267	14.82336	608.57	16.54625	611.33	16.03518
610.991	14.29925	14.92606	610.267	14.81086	609.57	16.49825	612.33	15.9875
611.991	14.28735	14.91295	611.267	14.80004	610.57	16.45027	613.33	15.9376
612.991	14.27627	14.90356	612.267	14.79166	611.57	16.40583	614.33	15.89181
613.991	14.26612	14.89413	613.267	14.78418	612.57	16.36397	615.33	15.8509
614.991	14.25638	14.88499	614.267	14.77724	613.57	16.32366	616.33	15.8123
615.991	14.24339	14.87458	615.267	14.76994	614.57	16.28737	617.33	15.77658
616.991	14.23006	14.86412	616.267	14.76241	615.57	16.25425	618.33	15.74351
617.991	14.2186	14.85456	617.267	14.75605	616.57	16.22219	619.33	15.70966
618.991	14.20475	14.84502	618.267	14.74916	617.57	16.19297	620.33	15.67868
619.991	14.19157	14.8342	619.267	14.73801	618.57	16.16491	621.33	15.64988
620.991	14.17697	14.82473	620.267	14.72716	619.57	16.13733	622.33	15.61854
621.991	14.15927	14.81248	621.267	14.71682	620.57	16.11316	623.33	15.58729
622.991	14.14055	14.79761	622.267	14.70269	621.57	16.08975	624.33	15.55945
623.991	14.12659	14.78272	623.267	14.68858	622.57	16.06416	625.33	15.53608

624.991	14.11716	14.77554	624.267	14.67704	623.57	16.04031	626.33	15.51442
625.991	14.10947	14.77021	625.267	14.66887	624.57	16.02243	627.33	15.49219
626.991	14.10259	14.76341	626.267	14.66185	625.57	16.0088	628.33	15.47094
627.991	14.09433	14.75648	627.267	14.65504	626.57	15.99576	629.33	15.44944
628.991	14.0828	14.7461	628.267	14.6461	627.57	15.98411	630.33	15.43102
629.991	14.07274	14.73266	629.267	14.63555	628.57	15.97165	631.33	15.41133
630.991	14.06227	14.72102	630.267	14.6259	629.57	15.95935	632.33	15.39087
631.991	14.05016	14.70989	631.267	14.61684	630.57	15.95003	633.33	15.37165
632.991	14.03857	14.69671	632.267	14.60599	631.57	15.93852	634.33	15.35227
633.991	14.02593	14.68317	633.267	14.59336	632.57	15.92536	635.33	15.33181
634.991	14.01052	14.66742	634.267	14.57959	633.57	15.91375	636.33	15.31206
635.991	13.99451	14.65071	635.267	14.5634	634.57	15.90108	637.33	15.29277
636.991	13.98142	14.6338	636.267	14.5485	635.57	15.88625	638.33	15.27593
637.991	13.96926	14.62113	637.267	14.53345	636.57	15.8705	639.33	15.26288
638.991	13.95664	14.61087	638.267	14.5193	637.57	15.85634	640.33	15.25214
639.991	13.94356	14.60109	639.267	14.50572	638.57	15.84422	641.33	15.23384
640.991	13.92491	14.5875	640.267	14.49419	639.57	15.83489	642.33	15.21877
641.991	13.90822	14.56948	641.267	14.47832	640.57	15.82536	643.33	15.2099
642.991	13.89479	14.55526	642.267	14.46268	641.57	15.81062	644.33	15.20247
643.991	13.88075	14.5426	643.267	14.45209	642.57	15.79971	645.33	15.19736
644.991	13.86611	14.53232	644.267	14.44232	643.57	15.79233	646.33	15.19294
645.991	13.85244	14.52141	645.267	14.43372	644.57	15.78658	647.33	15.18465
646.991	13.83808	14.50749	646.267	14.42589	645.57	15.78098	648.33	15.17588
647.991	13.82498	14.49147	647.267	14.4166	646.57	15.77468	649.33	15.1764
648.991	13.81902	14.48051	648.267	14.40551	647.57	15.76759	650.33	15.17578
649.991	13.80966	14.47606	649.267	14.40122	648.57	15.76211	651.33	15.16812
650.991	13.79325	14.4663	650.267	14.39711	649.57	15.76489	652.33	15.15837
651.991	13.77617	14.45162	651.267	14.38711	650.57	15.76338	653.33	15.14745
652.991	13.76163	14.43503	652.267	14.37483	651.57	15.75717	654.33	15.13856
653.991	13.74732	14.4197	653.267	14.36213	652.57	15.74831	655.33	15.13387
654.991	13.73616	14.40882	654.267	14.35038	653.57	15.74028	656.33	15.12724
655.991	13.72361	14.3984	655.267	14.34287	654.57	15.73466	657.33	15.11147
656.991	13.70354	14.38269	656.267	14.33571	655.57	15.73218	658.33	15.09598
657.991	13.6858	14.36361	657.267	14.32144	656.57	15.72727	659.33	15.08735
658.991	13.6745	14.34975	658.267	14.3066	657.57	15.71258	660.33	15.08129
659.991	13.66349	14.34137	659.267	14.29528	658.57	15.6995	661.33	15.07565
660.991	13.64999	14.33297	660.267	14.28469	659.57	15.69221	662.33	15.06947
661.991	13.63591	14.32183	661.267	14.27373	660.57	15.68835	663.33	15.06424
662.991	13.62307	14.30761	662.267	14.26202	661.57	15.68443	664.33	15.06424
663.991	13.61503	14.29783	663.267	14.24917	662.57	15.67888	665.33	15.06886
664.991	13.60963	14.29248	664.267	14.24023	663.57	15.67376	666.33	15.07259
665.991	13.60359	14.28631	665.267	14.2361	664.57	15.67344	667.33	15.07219
666.991	13.59328	14.27569	666.267	14.23222	665.57	15.67728	668.33	15.06972

667.991	13.58278	14.26257	667.267	14.22716	666.57	15.67957	669.33	15.06417
668.991	13.57172	14.24858	668.267	14.22111	667.57	15.67764	670.33	15.05859
669.991	13.56352	14.23597	669.267	14.21269	668.57	15.67445	671.33	15.05638
670.991	13.5562	14.2294	670.267	14.20406	669.57	15.67019	672.33	15.04655
671.991	13.53874	14.21678	671.267	14.19909	670.57	15.66893	673.33	15.03434
672.991	13.52126	14.19957	672.267	14.1865	671.57	15.66896	674.33	15.02174
673.991	13.50428	14.18257	673.267	14.17069	672.57	15.66105	675.33	15.01188
674.991	13.49077	14.16711	674.267	14.15356	673.57	15.65265	676.33	15.00363
675.991	13.47888	14.15366	675.267	14.13793	674.57	15.64561	677.33	14.99798
676.991	13.46862	14.14238	676.267	14.12497	675.57	15.6416	678.33	14.9918
677.991	13.45737	14.13198	677.267	14.11453	676.57	15.63687	679.33	14.98003
678.991	13.44317	14.11859	678.267	14.10408	677.57	15.63288	680.33	14.97191
679.991	13.43669	14.10722	679.267	14.08953	678.57	15.62547	681.33	14.96639
680.991	13.4321	14.10019	680.267	14.08032	679.57	15.61222	682.33	14.9624
681.991	13.42761	14.0957	681.267	14.07597	680.57	15.60341	683.33	14.95623
682.991	13.42027	14.09014	682.267	14.07505	681.57	15.59743	684.33	14.94969
683.991	13.41291	14.08101	683.267	14.07286	682.57	15.59249	685.33	14.94523
684.991	13.40699	14.07202	684.267	14.06935	683.57	15.58556	686.33	14.93976
685.991	13.40003	14.06312	685.267	14.06591	684.57	15.58102	687.33	14.93432
686.991	13.3926	14.05261	686.267	14.06361	685.57	15.5786	688.33	14.93217
687.991	13.38634	14.04534	687.267	14.05991	686.57	15.57613	689.33	14.92683
688.991	13.37516	14.03609	688.267	14.0578	687.57	15.57603	690.33	14.92011
689.991	13.36507	14.02115	689.267	14.05036	688.57	15.57825	691.33	14.91846
690.991	13.36166	14.01355	690.267	14.03967	689.57	15.57519	692.33	14.91768
691.991	13.35836	14.0122	691.267	14.03567	690.57	15.57317	693.33	14.91531
692.991	13.35282	14.01128	692.267	14.03422	691.57	15.57715	694.33	14.91615
693.991	13.3492	14.01123	693.267	14.03382	692.57	15.581	695.33	14.91542
694.991	13.34204	14.01163	694.267	14.03507	693.57	15.58352	696.33	14.90907
695.991	13.33157	14.00582	695.267	14.03504	694.57	15.58749	697.33	14.9059
696.991	13.32822	14.00249	696.267	14.02886	695.57	15.58773	698.33	14.90292
697.991	13.32549	14.00396	697.267	14.02622	696.57	15.58524	699.33	14.89524
698.991	13.31875	14.00084	698.267	14.02619	697.57	15.58924	700.33	14.88607
699.991	13.31077	13.99551	699.267	14.0218	698.57	15.59244	701.33	14.87479
700.991	13.30249	13.98815	700.267	14.01585	699.57	15.58992	702.33	14.85995
701.991	13.29229	13.97871	701.267	14.00781	700.57	15.58258	703.33	14.84593
702.991	13.2872	13.96998	702.267	13.99634	701.57	15.57353	704.33	14.83413
703.991	13.28521	13.96583	703.267	13.98717	702.57	15.5622	705.33	14.82176
704.991	13.27887	13.96087	704.267	13.98209	703.57	15.55446	706.33	14.81187
705.991	13.27332	13.95526	705.267	13.97673	704.57	15.54745	707.33	14.80574
706.991	13.269	13.95095	706.267	13.9715	705.57	15.53565	708.33	14.79613
707.991	13.26203	13.94333	707.267	13.96844	706.57	15.52399	709.33	14.7842
708.991	13.25571	13.93421	708.267	13.9639	707.57	15.51563	710.33	14.7758
709.991	13.25255	13.92662	709.267	13.959	708.57	15.50927	711.33	14.77246

710.991	13.25034	13.92116	710.267	13.95686	709.57	15.50374	712.33	14.76803
711.991	13.24716	13.9161	711.267	13.95593	710.57	15.5009	713.33	14.75941
712.991	13.24412	13.90914	712.267	13.95468	711.57	15.4993	714.33	14.74988
713.991	13.24042	13.90276	713.267	13.95051	712.57	15.49712	715.33	14.73773
714.991	13.23471	13.89584	714.267	13.94498	713.57	15.49477	716.33	14.72857
715.991	13.23303	13.8917	715.267	13.936	714.57	15.49076	717.33	14.72269
716.991	13.23455	13.89151	716.267	13.92858	715.57	15.48389	718.33	14.71751
717.991	13.23586	13.89285	717.267	13.9251	716.57	15.47842	719.33	14.71044
718.991	13.23685	13.89345	718.267	13.92263	717.57	15.47433	720.33	14.70524
719.991	13.23793	13.89312	719.267	13.9194	718.57	15.46822	721.33	14.70419
720.991	13.24096	13.89424	720.267	13.91613	719.57	15.46095	722.33	14.69922
721.991	13.243	13.89407	721.267	13.91598	720.57	15.45505	723.33	14.6882
722.991	13.24107	13.8904	722.267	13.91673	721.57	15.45178	724.33	14.6767
723.991	13.23959	13.88343	723.267	13.91489	722.57	15.44945	725.33	14.66989
724.991	13.23938	13.88002	724.267	13.91164	723.57	15.44409	726.33	14.65844
725.991	13.23529	13.8742	725.267	13.91032	724.57	15.43922	727.33	14.64734
726.991	13.23345	13.86768	726.267	13.90457	725.57	15.43345	728.33	14.63694
727.991	13.23384	13.86407	727.267	13.90078	726.57	15.42378	729.33	14.62804
728.991	13.2358	13.86174	728.267	13.8994	727.57	15.41505	730.33	14.62338
729.991	13.23938	13.86249	729.267	13.89954	728.57	15.40531	731.33	14.61905
730.991	13.24444	13.86574	730.267	13.90131	729.57	15.39543	732.33	14.61429
731.991	13.24935	13.8705	731.267	13.90464	730.57	15.38636	733.33	14.60314
732.991	13.24929	13.87206	732.267	13.90788	731.57	15.3789	734.33	14.59212
733.991	13.25002	13.87048	733.267	13.90665	732.57	15.37261	735.33	14.57814
734.991	13.24702	13.86747	734.267	13.90529	733.57	15.36419	736.33	14.56317
735.991	13.24323	13.86228	735.267	13.90184	734.57	15.35711	737.33	14.54366
736.991	13.2379	13.85665	736.267	13.89782	735.57	15.3486	738.33	14.52115
737.991	13.22967	13.84897	737.267	13.89315	736.57	15.33991	739.33	14.5019
738.991	13.22088	13.83831	738.267	13.88576	737.57	15.32867	740.33	14.48546
739.991	13.21247	13.82982	739.267	13.87576	738.57	15.31403	741.33	14.47167
740.991	13.20649	13.82194	740.267	13.86755	739.57	15.29824	742.33	14.45994
741.991	13.20263	13.81812	741.267	13.8613	740.57	15.2828	743.33	14.44994
742.991	13.2004	13.81692	742.267	13.85761	741.57	15.26977	744.33	14.43839
743.991	13.19786	13.81612	743.267	13.85509	742.57	15.25909	745.33	14.42995
744.991	13.19515	13.81515	744.267	13.8518	743.57	15.24864	746.33	14.42221
745.991	13.19396	13.81493	745.267	13.84704	744.57	15.2381	747.33	14.41304
746.991	13.19484	13.81629	746.267	13.84218	745.57	15.22874	748.33	14.40435
747.991	13.19618	13.81733	747.267	13.8411	746.57	15.21905	749.33	14.39414
748.991	13.1969	13.81771	748.267	13.84153	747.57	15.2121	750.33	14.38032
749.991	13.19797	13.81803	749.267	13.8412	748.57	15.20408	751.33	14.36622
750.991	13.19818	13.81827	750.267	13.84158	749.57	15.1936	752.33	14.35336
751.991	13.19805	13.81671	751.267	13.84187	750.57	15.18255	753.33	14.34278
752.991	13.1998	13.81696	752.267	13.84106	751.57	15.17026	754.33	14.32997

753.991	13.19837	13.81644	753.267	13.84335	752.57	15.15609	755.33	14.31825
754.991	13.1952	13.81408	754.267	13.84458	753.57	15.14266	756.33	14.30631
755.991	13.1915	13.81221	755.267	13.84362	754.57	15.12805	757.33	14.29231
756.991	13.18954	13.80953	756.267	13.84171	755.57	15.11142	758.33	14.2825
757.991	13.19038	13.80873	757.267	13.84007	756.57	15.09404	759.33	14.272
758.991	13.19036	13.80831	758.267	13.83926	757.57	15.07858	760.33	14.26039
759.991	13.1904	13.80718	759.267	13.8364	758.57	15.06725	761.33	14.24702
760.991	13.19082	13.80522	760.267	13.8332	759.57	15.05597	762.33	14.23703
761.991	13.19564	13.8054	761.267	13.82985	760.57	15.04626	763.33	14.2305
762.991	13.20353	13.8096	762.267	13.8323	761.57	15.03743	764.33	14.21969
763.991	13.2092	13.81519	763.267	13.83902	762.57	15.03179	765.33	14.20505
764.991	13.20947	13.81609	764.267	13.84598	763.57	15.02876	766.33	14.19043
765.991	13.20662	13.81349	765.267	13.84597	764.57	15.02289	767.33	14.17945
766.991	13.20699	13.8109	766.267	13.84286	765.57	15.01278	768.33	14.17084
767.991	13.20948	13.81062	767.267	13.84332	766.57	14.99903	769.33	14.15805
768.991	13.20778	13.80867	768.267	13.84619	767.57	14.98854	770.33	14.14062
769.991	13.20359	13.80259	769.267	13.84791	768.57	14.97642	771.33	14.121
770.991	13.19731	13.79414	770.267	13.84449	769.57	14.95914	772.33	14.10417
771.991	13.19323	13.7849	771.267	13.83867	770.57	14.94059	773.33	14.09487
772.991	13.1949	13.78077	772.267	13.83329	771.57	14.92099	774.33	14.08766
773.991	13.19811	13.78057	773.267	13.83513	772.57	14.9034	775.33	14.07838
774.991	13.19928	13.77946	774.267	13.84126	773.57	14.89149	776.33	14.06761
775.991	13.19844	13.77706	775.267	13.84363	774.57	14.88162	777.33	14.05526
776.991	13.19798	13.77191	776.267	13.84435	775.57	14.87094	778.33	14.04337
777.991	13.19783	13.76791	777.267	13.8421	776.57	14.86112	779.33	14.03287
778.991	13.1981	13.76413	778.267	13.83959	777.57	14.8537	780.33	14.02681
779.991	13.20093	13.76152	779.267	13.83618	778.57	14.84713	781.33	14.02231
780.991	13.20365	13.76001	780.267	13.83413	779.57	14.83945	782.33	14.01425
781.991	13.20515	13.75635	781.267	13.8323	780.57	14.83579	783.33	14.00588
782.991	13.20727	13.75263	782.267	13.82875	781.57	14.83178	784.33	13.99901
783.991	13.21056	13.75032	783.267	13.82778	782.57	14.82509	785.33	13.99695
784.991	13.21622	13.7515	784.267	13.82859	783.57	14.81675	786.33	13.99647
785.991	13.22274	13.75407	785.267	13.83226	784.57	14.81041	787.33	13.99634
786.991	13.23003	13.75746	786.267	13.83755	785.57	14.8063	788.33	13.99325
787.991	13.23508	13.76035	787.267	13.84395	786.57	14.80234	789.33	13.98887
788.991	13.23972	13.7624	788.267	13.84906	787.57	14.79975	790.33	13.98593
789.991	13.24324	13.76497	789.267	13.85373	788.57	14.79413	791.33	13.9792
790.991	13.24121	13.76464	790.267	13.85789	789.57	14.78764	792.33	13.97138
791.991	13.23716	13.76079	791.267	13.85737	790.57	14.7809	793.33	13.96302
792.991	13.23167	13.75535	792.267	13.85513	791.57	14.77232	794.33	13.95811
793.991	13.22711	13.7514	793.267	13.852	792.57	14.76308	795.33	13.94931
794.991	13.21568	13.74532	794.267	13.84952	793.57	14.7545	796.33	13.93746
795.991	13.19971	13.72837	795.267	13.84343	794.57	14.74459	797.33	13.92503

796.991		13.72142	796.267	13.83219	795.57	14.73232
			797.267	13.83644	796.57	14.72624

TG curve of water-immersed coking coal

temperature	***TG***	***temperature***	***TG***	***temperature***	***TG***
℃	%	℃	%	℃	%
	Coal		Coal+MgCl2		Coal+TEMPO
26.178	100	28.92	100	28.473	100
27.178	99.87946	29.92	99.82986	29.473	99.80961
28.178	99.69266	30.92	99.60687	30.473	99.55911
29.178	99.46293	31.92	99.32489	31.473	99.25302
30.178	99.22927	32.92	99.02081	32.473	98.92115
31.178	99.02982	33.92	98.7427	33.473	98.61621
32.178	98.84653	34.92	98.50293	34.473	98.35715
33.178	98.69483	35.92	98.31379	35.473	98.14612
34.178	98.56873	36.92	98.14986	36.473	97.97644
35.178	98.43646	37.92	97.9865	37.473	97.80448
36.178	98.3193	38.92	97.85157	38.473	97.65645
37.178	98.20494	39.92	97.72177	39.473	97.5153
38.178	98.10178	40.92	97.61065	40.473	97.3936
39.178	98.00162	41.92	97.50572	41.473	97.28057
40.178	97.90572	42.92	97.41033	42.473	97.17568
41.178	97.81582	43.92	97.33442	43.473	97.08698
42.178	97.73796	44.92	97.25371	44.473	97.00571
43.178	97.65421	45.92	97.17444	45.473	96.92077
44.178	97.56738	46.92	97.0983	46.473	96.84146
45.178	97.48324	47.92	97.02968	47.473	96.76887
46.178	97.40186	48.92	96.95235	48.473	96.68945
47.178	97.32075	49.92	96.87904	49.473	96.61208
48.178	97.23234	50.92	96.80268	50.473	96.53667
49.178	97.14606	51.92	96.73088	51.473	96.45907
50.178	97.05414	52.92	96.66959	52.473	96.39287
51.178	96.96977	53.92	96.60275	53.473	96.32658
52.178	96.89283	54.92	96.53669	54.473	96.25615
53.178	96.81214	55.92	96.4641	55.473	96.18372
54.178	96.72884	56.92	96.39792	56.473	96.1083
55.178	96.64382	57.92	96.33792	57.473	96.04401
56.178	96.55627	58.92	96.27348	58.473	95.97962
57.178	96.47909	59.92	96.21091	59.473	95.91505
58.178	96.40389	60.92	96.14149	60.473	95.85288
59.178	96.32906	61.92	96.07018	61.473	95.78675
60.178	96.25489	62.92	95.99367	62.473	95.72112
61.178	96.17847	63.92	95.92364	63.473	95.6633

62.178	96.10016	64.92	95.84721	64.473	95.60915
63.178	96.02962	65.92	95.76209	65.473	95.55499
64.178	95.96331	66.92	95.67158	66.473	95.49773
65.178	95.89448	67.92	95.57692	67.473	95.44236
66.178	95.82467	68.92	95.47698	68.473	95.38622
67.178	95.75506	69.92	95.36868	69.473	95.32913
68.178	95.6866	70.92	95.25533	70.473	95.2678
69.178	95.61912	71.92	95.15358	71.473	95.21416
70.178	95.54743	72.92	95.03662	72.473	95.16897
71.178	95.47619	73.92	94.91464	73.473	95.10092
72.178	95.419	74.92	94.80949	74.473	95.0467
73.178	95.3482	75.92	94.7083	75.473	95.00556
74.178	95.273	76.92	94.60243	76.473	94.95953
75.178	95.21994	77.92	94.4941	77.473	94.90906
76.178	95.16866	78.92	94.3857	78.473	94.85241
77.178	95.11334	79.92	94.30043	79.473	94.80259
78.178	95.05071	80.92	94.21781	80.473	94.77358
79.178	94.98474	81.92	94.11723	81.473	94.72464
80.178	94.94057	82.92	94.03061	82.473	94.67273
81.178	94.90044	83.92	93.95205	83.473	94.63295
82.178	94.83566	84.92	93.8607	84.473	94.58379
83.178	94.78391	85.92	93.77416	85.473	94.52887
84.178	94.73492	86.92	93.69316	86.473	94.48076
85.178	94.67292	87.92	93.62353	87.473	94.44129
86.178	94.61659	88.92	93.55486	88.473	94.40831
87.178	94.56762	89.92	93.47253	89.473	94.36848
88.178	94.52816	90.92	93.39275	90.473	94.31579
89.178	94.49004	91.92	93.32768	91.473	94.27559
90.178	94.43504	92.92	93.26338	92.473	94.24143
91.178	94.383	93.92	93.19401	93.473	94.20589
92.178	94.34271	94.92	93.12305	94.473	94.16894
93.178	94.30522	95.92	93.05356	95.473	94.13059
94.178	94.26421	96.92	92.97956	96.473	94.09173
95.178	94.22272	97.92	92.90084	97.473	94.0535
96.178	94.18018	98.92	92.82047	98.473	94.01596
97.178	94.13764	99.92	92.73565	99.473	93.97942
98.178	94.09616	100.92	92.64395	100.473	93.9441
99.178	94.0568	101.92	92.54093	101.473	93.90953
100.178	94.01984	102.92	92.4359	102.473	93.87321
101.178	93.98403	103.92	92.33155	103.473	93.84354
102.178	93.94577	104.92	92.21551	104.473	93.81398
103.178	93.91168	105.92	92.09708	105.473	93.78271
104.178	93.88235	106.92	91.97587	106.473	93.74573

105.178	93.85103	107.92	91.86091	107.473	93.70705
106.178	93.81771	108.92	91.7508	108.473	93.6707
107.178	93.77869	109.92	91.64515	109.473	93.63417
108.178	93.74463	110.92	91.55617	110.473	93.60107
109.178	93.70858	111.92	91.47237	111.473	93.57012
110.178	93.6719	112.92	91.3881	112.473	93.53183
111.178	93.64214	113.92	91.31504	113.473	93.48948
112.178	93.61181	114.92	91.2509	114.473	93.44945
113.178	93.57158	115.92	91.19836	115.473	93.41496
114.178	93.53403	116.92	91.14829	116.473	93.38496
115.178	93.4993	117.92	91.10283	117.473	93.35367
116.178	93.47114	118.92	91.0628	118.473	93.32551
117.178	93.44083	119.92	91.01652	119.473	93.28968
118.178	93.41221	120.92	90.97083	120.473	93.25046
119.178	93.38512	121.92	90.93172	121.473	93.21413
120.178	93.35021	122.92	90.8932	122.473	93.18025
121.178	93.31382	123.92	90.85841	123.473	93.14683
122.178	93.28114	124.92	90.82347	124.473	93.11125
123.178	93.24868	125.92	90.78873	125.473	93.07299
124.178	93.21547	126.92	90.75572	126.473	93.03544
125.178	93.17884	127.92	90.72335	127.473	92.99772
126.178	93.14213	128.92	90.69167	128.473	92.9643
127.178	93.10456	129.92	90.66158	129.473	92.92961
128.178	93.06979	130.92	90.63359	130.473	92.89566
129.178	93.0356	131.92	90.60493	131.473	92.86053
130.178	93.00529	132.92	90.5752	132.473	92.82561
131.178	92.97544	133.92	90.54525	133.473	92.79515
132.178	92.94472	134.92	90.51627	134.473	92.76587
133.178	92.9118	135.92	90.48258	135.473	92.73504
134.178	92.88196	136.92	90.44216	136.473	92.70131
135.178	92.85141	137.92	90.40555	137.473	92.67125
136.178	92.81919	138.92	90.36271	138.473	92.64018
137.178	92.78669	139.92	90.31833	139.473	92.609
138.178	92.75516	140.92	90.2727	140.473	92.58048
139.178	92.7206	141.92	90.22352	141.473	92.5502
140.178	92.69	142.92	90.16675	142.473	92.5159
141.178	92.66272	143.92	90.10412	143.473	92.47529
142.178	92.63267	144.92	90.04922	144.473	92.43874
143.178	92.59811	145.92	89.99841	145.473	92.40744
144.178	92.56058	146.92	89.94685	146.473	92.37319
145.178	92.53024	147.92	89.89219	147.473	92.33688
146.178	92.49845	148.92	89.84415	148.473	92.30081
147.178	92.46969	149.92	89.79941	149.473	92.27092

148.178	92.43449	150.92	89.75303	150.473	92.23696
149.178	92.40354	151.92	89.71389	151.473	92.20128
150.178	92.3738	152.92	89.67804	152.473	92.17443
151.178	92.34033	153.92	89.63794	153.473	92.1437
152.178	92.31406	154.92	89.59675	154.473	92.11026
153.178	92.28773	155.92	89.56409	155.473	92.08066
154.178	92.25574	156.92	89.53663	156.473	92.06048
155.178	92.22372	157.92	89.49759	157.473	92.03014
156.178	92.20164	158.92	89.46279	158.473	91.99634
157.178	92.17891	159.92	89.43194	159.473	91.9724
158.178	92.1463	160.92	89.4009	160.473	91.95021
159.178	92.11962	161.92	89.3585	161.473	91.91938
160.178	92.09675	162.92	89.31809	162.473	91.88616
161.178	92.07118	163.92	89.27707	163.473	91.85689
162.178	92.03697	164.92	89.23476	164.473	91.82484
163.178	92.00427	165.92	89.19613	165.473	91.79484
164.178	91.96979	166.92	89.15904	166.473	91.7671
165.178	91.93643	167.92	89.12407	167.473	91.74387
166.178	91.90684	168.92	89.08466	168.473	91.71831
167.178	91.88219	169.92	89.0493	169.473	91.69107
168.178	91.85962	170.92	89.02115	170.473	91.6734
169.178	91.83343	171.92	88.99283	171.473	91.65448
170.178	91.81653	172.92	88.96192	172.473	91.63475
171.178	91.80269	173.92	88.92508	173.473	91.61113
172.178	91.78805	174.92	88.88858	174.473	91.58759
173.178	91.77094	175.92	88.85096	175.473	91.56155
174.178	91.75057	176.92	88.81682	176.473	91.53553
175.178	91.73118	177.92	88.78483	177.473	91.51129
176.178	91.70821	178.92	88.75397	178.473	91.48956
177.178	91.68643	179.92	88.72294	179.473	91.46881
178.178	91.66802	180.92	88.69093	180.473	91.44844
179.178	91.65355	181.92	88.6609	181.473	91.42935
180.178	91.63993	182.92	88.63068	182.473	91.41064
181.178	91.62536	183.92	88.60275	183.473	91.39229
182.178	91.60982	184.92	88.57618	184.473	91.37848
183.178	91.59168	185.92	88.54584	185.473	91.36169
184.178	91.57625	186.92	88.51974	186.473	91.34299
185.178	91.56258	187.92	88.4922	187.473	91.32529
186.178	91.5451	188.92	88.45743	188.473	91.30338
187.178	91.5292	189.92	88.43061	189.473	91.2782
188.178	91.51072	190.92	88.40875	190.473	91.26167
189.178	91.48783	191.92	88.38093	191.473	91.24349
190.178	91.47589	192.92	88.34942	192.473	91.2222

191.178	91.46641	193.92	88.32063	193.473	91.19841
192.178	91.45296	194.92	88.2903	194.473	91.17765
193.178	91.4367	195.92	88.25704	195.473	91.154
194.178	91.4249	196.92	88.22957	196.473	91.132
195.178	91.41146	197.92	88.1992	197.473	91.11329
196.178	91.39894	198.92	88.16428	198.473	91.09001
197.178	91.39171	199.92	88.12886	199.473	91.06812
198.178	91.37862	200.92	88.08704	200.473	91.04237
199.178	91.36558	201.92	88.05301	201.473	91.01854
200.178	91.35178	202.92	88.02151	202.473	91.00153
201.178	91.33471	203.92	87.99253	203.473	90.98839
202.178	91.32559	204.92	87.95796	204.473	90.97033
203.178	91.32025	205.92	87.92139	205.473	90.94994
204.178	91.31355	206.92	87.88846	206.473	90.93526
205.178	91.30123	207.92	87.8565	207.473	90.91723
206.178	91.29272	208.92	87.82961	208.473	90.89978
207.178	91.28351	209.92	87.79416	209.473	90.88173
208.178	91.27695	210.92	87.75429	210.473	90.85663
209.178	91.27462	211.92	87.71453	211.473	90.83079
210.178	91.26472	212.92	87.68211	212.473	90.81142
211.178	91.25123	213.92	87.65314	213.473	90.79586
212.178	91.23953	214.92	87.61546	214.473	90.77732
213.178	91.2328	215.92	87.57865	215.473	90.75776
214.178	91.22737	216.92	87.54126	216.473	90.74002
215.178	91.21765	217.92	87.50975	217.473	90.72616
216.178	91.20848	218.92	87.47803	218.473	90.71354
217.178	91.20125	219.92	87.44541	219.473	90.69767
218.178	91.19765	220.92	87.40745	220.473	90.67598
219.178	91.19433	221.92	87.37461	221.473	90.65474
220.178	91.18811	222.92	87.34332	222.473	90.63316
221.178	91.17723	223.92	87.31557	223.473	90.61262
222.178	91.17012	224.92	87.28736	224.473	90.59356
223.178	91.16387	225.92	87.25952	225.473	90.57512
224.178	91.158	226.92	87.23572	226.473	90.55771
225.178	91.15186	227.92	87.20647	227.473	90.53972
226.178	91.14358	228.92	87.18174	228.473	90.51393
227.178	91.13728	229.92	87.16507	229.473	90.50028
228.178	91.12288	230.92	87.14402	230.473	90.48414
229.178	91.11512	231.92	87.12281	231.473	90.46261
230.178	91.10957	232.92	87.10693	232.473	90.43883
231.178	91.0967	233.92	87.08963	233.473	90.41375
232.178	91.08322	234.92	87.07049	234.473	90.38508
233.178	91.07129	235.92	87.05283	235.473	90.35659

234.178	91.0569	236.92	87.03867	236.473	90.33559
235.178	91.04063	237.92	87.01456	237.473	90.30635
236.178	91.02859	238.92	86.99651	238.473	90.27561
237.178	91.01882	239.92	86.97655	239.473	90.24872
238.178	91.00046	240.92	86.95855	240.473	90.22372
239.178	90.98792	241.92	86.93661	241.473	90.1968
240.178	90.97334	242.92	86.91744	242.473	90.16842
241.178	90.95838	243.92	86.90393	243.473	90.14241
242.178	90.93951	244.92	86.89527	244.473	90.12033
243.178	90.92274	245.92	86.88303	245.473	90.09771
244.178	90.9075	246.92	86.86897	246.473	90.07399
245.178	90.89114	247.92	86.85877	247.473	90.05061
246.178	90.87451	248.92	86.83902	248.473	90.02289
247.178	90.8596	249.92	86.82191	249.473	89.98945
248.178	90.8452	250.92	86.80739	250.473	89.96298
249.178	90.82303	251.92	86.78835	251.473	89.9331
250.178	90.80607	252.92	86.76444	252.473	89.89845
251.178	90.78829	253.92	86.74025	253.473	89.86377
252.178	90.76808	254.92	86.7115	254.473	89.82664
253.178	90.74614	255.92	86.67894	255.473	89.78327
254.178	90.72326	256.92	86.65325	256.473	89.74361
255.178	90.69045	257.92	86.63137	257.473	89.71145
256.178	90.65632	258.92	86.60483	258.473	89.67628
257.178	90.62792	259.92	86.5709	259.473	89.63457
258.178	90.60162	260.92	86.53527	260.473	89.58535
259.178	90.56956	261.92	86.50797	261.473	89.53965
260.178	90.52799	262.92	86.48912	262.473	89.50136
261.178	90.48685	263.92	86.46458	263.473	89.46886
262.178	90.44971	264.92	86.43084	264.473	89.42301
263.178	90.42108	265.92	86.39605	265.473	89.36986
264.178	90.38357	266.92	86.36396	266.473	89.31674
265.178	90.34263	267.92	86.33353	267.473	89.26595
266.178	90.29846	268.92	86.30881	268.473	89.21973
267.178	90.25392	269.92	86.2763	269.473	89.17199
268.178	90.21073	270.92	86.23135	270.473	89.11184
269.178	90.17326	271.92	86.18865	271.473	89.04392
270.178	90.1305	272.92	86.14895	272.473	88.9804
271.178	90.07697	273.92	86.10804	273.473	88.92093
272.178	90.02674	274.92	86.06593	274.473	88.85863
273.178	89.97753	275.92	86.02349	275.473	88.79731
274.178	89.92347	276.92	85.97489	276.473	88.73207
275.178	89.87183	277.92	85.92128	277.473	88.65855
276.178	89.82021	278.92	85.8719	278.473	88.58329

277.178	89.75577	279.92	85.82182	279.473	88.51023
278.178	89.68562	280.92	85.77123	280.473	88.43713
279.178	89.61832	281.92	85.71966	281.473	88.3614
280.178	89.54787	282.92	85.66612	282.473	88.28757
281.178	89.4753	283.92	85.61237	283.473	88.21052
282.178	89.40296	284.92	85.54578	284.473	88.1292
283.178	89.32921	285.92	85.48202	285.473	88.04333
284.178	89.25614	286.92	85.42049	286.473	87.96004
285.178	89.17359	287.92	85.35734	287.473	87.87918
286.178	89.0959	288.92	85.29246	288.473	87.79716
287.178	89.01781	289.92	85.22777	289.473	87.71454
288.178	88.93916	290.92	85.15343	290.473	87.62724
289.178	88.85997	291.92	85.08007	291.473	87.52894
290.178	88.7792	292.92	85.0181	292.473	87.44412
291.178	88.68441	293.92	84.94348	293.473	87.35653
292.178	88.59413	294.92	84.85847	294.473	87.25184
293.178	88.50727	295.92	84.78519	295.473	87.15085
294.178	88.41018	296.92	84.70802	296.473	87.05903
295.178	88.30267	297.92	84.6161	297.473	86.95499
296.178	88.20822	298.92	84.52659	298.473	86.84548
297.178	88.10734	299.92	84.43047	299.473	86.73578
298.178	87.99481	300.92	84.33285	300.473	86.62019
299.178	87.88627	301.92	84.23947	301.473	86.50603
300.178	87.77008	302.92	84.15256	302.473	86.40283
301.178	87.65102	303.92	84.04049	303.473	86.29026
302.178	87.53705	304.92	83.92829	304.473	86.16087
303.178	87.42351	305.92	83.82239	305.473	86.03945
304.178	87.28669	306.92	83.7183	306.473	85.92355
305.178	87.15601	307.92	83.61201	307.473	85.80391
306.178	87.0332	308.92	83.50264	308.473	85.68531
307.178	86.9058	309.92	83.39308	309.473	85.56239
308.178	86.7789	310.92	83.2777	310.473	85.43492
309.178	86.64916	311.92	83.16806	311.473	85.30179
310.178	86.51468	312.92	83.05908	312.473	85.17335
311.178	86.37903	313.92	82.941	313.473	85.04198
312.178	86.24422	314.92	82.82149	314.473	84.90326
313.178	86.10923	315.92	82.69353	315.473	84.76383
314.178	85.96343	316.92	82.56341	316.473	84.61556
315.178	85.82034	317.92	82.42645	317.473	84.46368
316.178	85.66677	318.92	82.2874	318.473	84.31192
317.178	85.51296	319.92	82.145	319.473	84.15624
318.178	85.3575	320.92	82.001	320.473	84.00109
319.178	85.19779	321.92	81.85065	321.473	83.83699

320.178	85.03679	322.92	81.70024	322.473	83.67588
321.178	84.86618	323.92	81.55081	323.473	83.5093
322.178	84.69156	324.92	81.39975	324.473	83.34539
323.178	84.5185	325.92	81.2447	325.473	83.1788
324.178	84.34362	326.92	81.09437	326.473	83.00922
325.178	84.16308	327.92	80.93764	327.473	82.83964
326.178	83.98021	328.92	80.7752	328.473	82.66341
327.178	83.79983	329.92	80.61351	329.473	82.48697
328.178	83.61967	330.92	80.44943	330.473	82.30453
329.178	83.43208	331.92	80.27909	331.473	82.11915
330.178	83.24988	332.92	80.10355	332.473	81.93212
331.178	83.06078	333.92	79.92884	333.473	81.7395
332.178	82.86987	334.92	79.75017	334.473	81.54649
333.178	82.67378	335.92	79.57037	335.473	81.35332
334.178	82.47446	336.92	79.38693	336.473	81.15581
335.178	82.2772	337.92	79.19734	337.473	80.95436
336.178	82.07297	338.92	79.00761	338.473	80.75761
337.178	81.86766	339.92	78.81738	339.473	80.55384
338.178	81.65628	340.92	78.62602	340.473	80.35142
339.178	81.44188	341.92	78.43718	341.473	80.14743
340.178	81.22364	342.92	78.23833	342.473	79.93939
341.178	81.00926	343.92	78.03452	343.473	79.72553
342.178	80.7888	344.92	77.8309	344.473	79.51065
343.178	80.56277	345.92	77.62291	345.473	79.29453
344.178	80.33801	346.92	77.41365	346.473	79.07589
345.178	80.11246	347.92	77.19984	347.473	78.8452
346.178	79.87685	348.92	76.98134	348.473	78.61316
347.178	79.63588	349.92	76.7611	349.473	78.3779
348.178	79.39541	350.92	76.53329	350.473	78.13351
349.178	79.14733	351.92	76.3043	351.473	77.8835
350.178	78.89085	352.92	76.07132	352.473	77.63291
351.178	78.63455	353.92	75.84176	353.473	77.38354
352.178	78.36953	354.92	75.60984	354.473	77.12976
353.178	78.10244	355.92	75.37367	355.473	76.86873
354.178	77.83885	356.92	75.12826	356.473	76.61107
355.178	77.56631	357.92	74.87804	357.473	76.33989
356.178	77.28711	358.92	74.62177	358.473	76.06961
357.178	76.99813	359.92	74.36665	359.473	75.79678
358.178	76.70314	360.92	74.11256	360.473	75.51767
359.178	76.41507	361.92	73.85441	361.473	75.23666
360.178	76.12184	362.92	73.58805	362.473	74.94652
361.178	75.81777	363.92	73.32003	363.473	74.65694
362.178	75.51226	364.92	73.04597	364.473	74.35967

363.178	75.19379	365.92	72.77282	365.473	74.04979
364.178	74.86811	366.92	72.4911	366.473	73.73795
365.178	74.54335	367.92	72.20444	367.473	73.41782
366.178	74.21139	368.92	71.90429	368.473	73.08592
367.178	73.86576	369.92	71.60205	369.473	72.74745
368.178	73.50851	370.92	71.29774	370.473	72.40512
369.178	73.15009	371.92	70.99162	371.473	72.06069
370.178	72.77605	372.92	70.68172	372.473	71.70851
371.178	72.39669	373.92	70.37051	373.473	71.35108
372.178	72.01574	374.92	70.05252	374.473	70.99212
373.178	71.62155	375.92	69.73103	375.473	70.62141
374.178	71.22245	376.92	69.40975	376.473	70.24413
375.178	70.81438	377.92	69.07959	377.473	69.85498
376.178	70.39554	378.92	68.74886	378.473	69.45829
377.178	69.96077	379.92	68.40758	379.473	69.05115
378.178	69.51977	380.92	68.06778	380.473	68.62813
379.178	69.05887	381.92	67.71499	381.473	68.18984
380.178	68.57775	382.92	67.3529	382.473	67.7487
381.178	68.09441	383.92	66.99137	383.473	67.29128
382.178	67.59161	384.92	66.63129	384.473	66.82731
383.178	67.06317	385.92	66.25798	385.473	66.35518
384.178	66.52006	386.92	65.87342	386.473	65.85347
385.178	65.97061	387.92	65.48808	387.473	65.3363
386.178	65.39173	388.92	65.10762	388.473	64.81426
387.178	64.79204	389.92	64.72113	389.473	64.27058
388.178	64.1881	390.92	64.33464	390.473	63.72729
389.178	63.56047	391.92	63.92585	391.473	63.14973
390.178	62.9278	392.92	63.50802	392.473	62.54999
391.178	62.26847	393.92	63.09876	393.473	61.94049
392.178	61.58489	394.92	62.68894	394.473	61.33306
393.178	60.86259	395.92	62.26851	395.473	60.70124
394.178	60.14053	396.92	61.83156	396.473	60.04863
395.178	59.40967	397.92	61.38334	397.473	59.37138
396.178	58.65083	398.92	60.94383	398.473	58.66456
397.178	57.85928	399.92	60.48319	399.473	57.94498
398.178	57.029	400.92	60.03947	400.473	57.20467
399.178	56.17539	401.92	59.57054	401.473	56.44572
400.178	55.29725	402.92	59.10188	402.473	55.67672
401.178	54.38403	403.92	58.62272	403.473	54.88334
402.178	53.4516	404.92	58.14898	404.473	54.06477
403.178	52.46768	405.92	57.66665	405.473	53.21705
404.178	51.44531	406.92	57.17574	406.473	52.37019
405.178	50.37715	407.92	56.68032	407.473	51.47493

406.178	49.2815	408.92	56.17323	408.473	50.54788
407.178	48.12728	409.92	55.64724	409.473	49.60745
408.178	46.92731	410.92	55.10428	410.473	48.61815
409.178	45.72158	411.92	54.55295	411.473	47.60906
410.178	44.46745	412.92	54.00525	412.473	46.55833
411.178	43.15855	413.92	53.44187	413.473	45.46941
412.178	41.81476	414.92	52.86571	414.473	44.37764
413.178	40.43598	415.92	52.26325	415.473	43.234
414.178	39.02314	416.92	51.65943	416.473	42.08729
415.178	37.60948	417.92	51.04766	417.473	40.87078
416.178	36.13529	418.92	50.41495	418.473	39.67641
417.178	34.63067	419.92	49.77548	419.473	38.41725
418.178	33.08936	420.92	49.11168	420.473	37.15055
419.178	31.6	421.92	48.42054	421.473	35.88686
420.178	30.08379	422.92	47.71832	422.473	34.59754
421.178	28.55956	423.92	46.98439	423.473	33.27858
422.178	27.09389	424.92	46.23601	424.473	31.95135
423.178	25.6385	425.92	45.48076	425.473	30.67683
424.178	24.26611	426.92	44.70442	426.473	29.38699
425.178	22.9321	427.92	43.89795	427.473	28.13022
426.178	21.66797	428.92	43.06383	428.473	26.91572
427.178	20.49832	429.92	42.2084	429.473	25.69595
428.178	19.39508	430.92	41.33939	430.473	24.54776
429.178	18.35634	431.92	40.43045	431.473	23.44207
430.178	17.36778	432.92	39.5006	432.473	22.37294
431.178	16.45955	433.92	38.52701	433.473	21.35472
432.178	15.60043	434.92	37.51937	434.473	20.37974
433.178	14.80112	435.92	36.50017	435.473	19.43983
434.178	14.03394	436.92	35.45137	436.473	18.55866
435.178	13.31506	437.92	34.40439	437.473	17.73763
436.178	12.66202	438.92	33.31162	438.473	16.94055
437.178	12.05713	439.92	32.23146	439.473	16.1993
438.178	11.49258	440.92	31.13995	440.473	15.49901
439.178	10.95985	441.92	30.03664	441.473	14.82188
440.178	10.46739	442.92	28.92556	442.473	14.16482
441.178	9.99755	443.92	27.82734	443.473	13.56823
442.178	9.56752	444.92	26.74092	444.473	12.96422
443.178	9.17655	445.92	25.65168	445.473	12.40943
444.178	8.81373	446.92	24.60053	446.473	11.86274
445.178	8.49989	447.92	23.55059	447.473	11.35685
446.178	8.21508	448.92	22.53941	448.473	10.87106
447.178	7.96089	449.92	21.56249	449.473	10.41408
448.178	7.73492	450.92	20.62783	450.473	9.99252

449.178	7.53103	451.92	19.72678	451.473	9.59991
450.178	7.35028	452.92	18.89259	452.473	9.24942
451.178	7.19935	453.92	18.10682	453.473	8.93048
452.178	7.06827	454.92	17.38794	454.473	8.65301
453.178	6.94863	455.92	16.71551	455.473	8.40606
454.178	6.85026	456.92	16.10682	456.473	8.19224
455.178	6.7629	457.92	15.54244	457.473	7.99665
456.178	6.68551	458.92	15.02646	458.473	7.824
457.178	6.61317	459.92	14.56498	459.473	7.67535
458.178	6.54797	460.92	14.15135	460.473	7.55007
459.178	6.4884	461.92	13.78082	461.473	7.44051
460.178	6.43647	462.92	13.44695	462.473	7.34221
461.178	6.38871	463.92	13.15084	463.473	7.25332
462.178	6.33951	464.92	12.89425	464.473	7.17911
463.178	6.28839	465.92	12.67158	465.473	7.11603
464.178	6.2398	466.92	12.46863	466.473	7.05753
465.178	6.19763	467.92	12.2868	467.473	7.00079
466.178	6.15646	468.92	12.11978	468.473	6.94452
467.178	6.11252	469.92	11.97435	469.473	6.88746
468.178	6.06536	470.92	11.84511	470.473	6.83299
469.178	6.01692	471.92	11.73172	471.473	6.78594
470.178	5.96652	472.92	11.6281	472.473	6.74122
471.178	5.92174	473.92	11.53312	473.473	6.69839
472.178	5.87933	474.92	11.44908	474.473	6.65765
473.178	5.83945	475.92	11.37151	475.473	6.61707
474.178	5.79723	476.92	11.3002	476.473	6.5736
475.178	5.75407	477.92	11.23407	477.473	6.53732
476.178	5.70782	478.92	11.16724	478.473	6.50215
477.178	5.66592	479.92	11.10071	479.473	6.46453
478.178	5.62787	480.92	11.03154	480.473	6.42514
479.178	5.58933	481.92	10.95941	481.473	6.38212
480.178	5.55128	482.92	10.88616	482.473	6.33579
481.178	5.50676	483.92	10.81876	483.473	6.29359
482.178	5.45664	484.92	10.74974	484.473	6.25923
483.178	5.40905	485.92	10.67662	485.473	6.2194
484.178	5.36874	486.92	10.59973	486.473	6.17568
485.178	5.32754	487.92	10.52275	487.473	6.12909
486.178	5.28035	488.92	10.44632	488.473	6.0848
487.178	5.22872	489.92	10.37335	489.473	6.04626
488.178	5.17745	490.92	10.29951	490.473	6.01023
489.178	5.13188	491.92	10.22108	491.473	5.97093
490.178	5.09384	492.92	10.13676	492.473	5.9291
491.178	5.05519	493.92	10.05312	493.473	5.88662

492.178	5.01569	494.92	9.96924	494.473	5.84673
493.178	4.97196	495.92	9.87947	495.473	5.80868
494.178	4.92841	496.92	9.78544	496.473	5.76964
495.178	4.88797	497.92	9.69197	497.473	5.72346
496.178	4.85155	498.92	9.59452	498.473	5.68409
497.178	4.80825	499.92	9.49295	499.473	5.63709
498.178	4.76959	500.92	9.38936	500.473	5.58828
499.178	4.72893	501.92	9.28112	501.473	5.53854
500.178	4.68569	502.92	9.17508	502.473	5.48939
501.178	4.64368	503.92	9.07333	503.473	5.44625
502.178	4.60443	504.92	8.96817	504.473	5.40241
503.178	4.5719	505.92	8.85389	505.473	5.3567
504.178	4.54136	506.92	8.73621	506.473	5.3009
505.178	4.50877	507.92	8.62108	507.473	5.25074
506.178	4.46726	508.92	8.51186	508.473	5.2044
507.178	4.42646	509.92	8.40208	509.473	5.16291
508.178	4.38852	510.92	8.28582	510.473	5.11786
509.178	4.35661	511.92	8.17368	511.473	5.07015
510.178	4.32082	512.92	8.06678	512.473	5.03179
511.178	4.27865	513.92	7.96335	513.473	4.99503
512.178	4.23944	514.92	7.86533	514.473	4.95739
513.178	4.20391	515.92	7.77497	515.473	4.92161
514.178	4.1675	516.92	7.68371	516.473	4.88978
515.178	4.13396	517.92	7.59942	517.473	4.85428
516.178	4.10647	518.92	7.51446	518.473	4.82193
517.178	4.07535	519.92	7.42763	519.473	4.7828
518.178	4.04832	520.92	7.34653	520.473	4.73779
519.178	4.01639	521.92	7.27887	521.473	4.70345
520.178	3.97925	522.92	7.21615	522.473	4.67574
521.178	3.94646	523.92	7.15414	523.473	4.64664
522.178	3.92455	524.92	7.0919	524.473	4.61251
523.178	3.90294	525.92	7.03621	525.473	4.5802
524.178	3.87503	526.92	6.99199	526.473	4.55155
525.178	3.84444	527.92	6.95139	527.473	4.53262
526.178	3.81535	528.92	6.9164	528.473	4.51754
527.178	3.79686	529.92	6.87712	529.473	4.50041
528.178	3.78063	530.92	6.84066	530.473	4.48004
529.178	3.76613	531.92	6.80842	531.473	4.46136
530.178	3.74387	532.92	6.77786	532.473	4.44262
531.178	3.72212	533.92	6.74761	533.473	4.4237
532.178	3.70152	534.92	6.719	534.473	4.40566
533.178	3.68351	535.92	6.69579	535.473	4.38991
534.178	3.66589	536.92	6.67067	536.473	4.3732

535.178	3.64558	537.92	6.64803	537.473	4.35141
536.178	3.62768	538.92	6.6222	538.473	4.33136
537.178	3.60505	539.92	6.59919	539.473	4.30927
538.178	3.58573	540.92	6.58059	540.473	4.29453
539.178	3.55948	541.92	6.56375	541.473	4.28242
540.178	3.54019	542.92	6.54745	542.473	4.2697
541.178	3.52321	543.92	6.53446	543.473	4.2565
542.178	3.50934	544.92	6.51958	544.473	4.24296
543.178	3.49866	545.92	6.50572	545.473	4.22946
544.178	3.48777	546.92	6.50069	546.473	4.22172
545.178	3.47588	547.92	6.49211	547.473	4.21912
546.178	3.46621	548.92	6.48316	548.473	4.21003
547.178	3.4663	549.92	6.47257	549.473	4.20202
548.178	3.45942	550.92	6.46224	550.473	4.19179
549.178	3.45206	551.92	6.4507	551.473	4.18247
550.178	3.43918	552.92	6.44689	552.473	4.17494
551.178	3.42612	553.92	6.44105	553.473	4.17027
552.178	3.41297	554.92	6.43106	554.473	4.15922
553.178	3.4074	555.92	6.42494	555.473	4.14717
554.178	3.3979	556.92	6.41881	556.473	4.13728
555.178	3.38371	557.92	6.41803	557.473	4.1273
556.178	3.3745	558.92	6.42015	558.473	4.12331
557.178	3.36368	559.92	6.42115	559.473	4.11871
558.178	3.36042	560.92	6.4139	560.473	4.11255
559.178	3.35863	561.92	6.4075	561.473	4.10286
560.178	3.35612	562.92	6.40206	562.473	4.09733
561.178	3.34703	563.92	6.39892	563.473	4.09434
562.178	3.34212	564.92	6.39456	564.473	4.09292
563.178	3.34064	565.92	6.38701	565.473	4.08992
564.178	3.34286	566.92	6.37663	566.473	4.08385
565.178	3.34403	567.92	6.36642	567.473	4.07574
566.178	3.34134	568.92	6.35954	568.473	4.06843
567.178	3.33563	569.92	6.35577	569.473	4.06378
568.178	3.32834	570.92	6.35367	570.473	4.06023
569.178	3.32202	571.92	6.34886	571.473	4.0559
570.178	3.31718	572.92	6.34375	572.473	4.04892
571.178	3.31285	573.92	6.33887	573.473	4.04294
572.178	3.30588	574.92	6.33274	574.473	4.03786
573.178	3.29982	575.92	6.33103	575.473	4.0348
574.178	3.29384	576.92	6.3317	576.473	4.03919
575.178	3.28679	577.92	6.33022	577.473	4.04196
576.178	3.28694	578.92	6.32923	578.473	4.0449
577.178	3.29E+00	579.92	6.32977	579.473	4.05048

578.178	3.29016	580.92	6.33189	580.473	4.05878
579.178	3.29255	581.92	6.33568	581.473	4.0682
580.178	3.29604	582.92	6.33724	582.473	4.07676
581.178	3.30006	583.92	6.33483	583.473	4.0809
582.178	3.30539	584.92	6.32778	584.473	4.07722
583.178	3.31E+00	585.92	6.32241	585.473	4.07391
584.178	3.30705	586.92	6.31543	586.473	4.06974
585.178	3.30309	587.92	6.30593	587.473	4.06251
586.178	3.30028	588.92	6.29741	588.473	4.05479
587.178	3.29266	589.92	6.28894	589.473	4.04911
588.178	3.28307	590.92	6.28438	590.473	4.04413
589.178	3.27616	591.92	6.28327	591.473	4.04423
590.178	3.27025	592.92	6.2842	592.473	4.04673
591.178	3.26905	593.92	6.28372	593.473	4.04961
592.178	3.27041	594.92	6.28534	594.473	4.05115
593.178	3.27184	595.92	6.28984	595.473	4.05337
594.178	3.27065	596.92	6.29357	596.473	4.05569
595.178	3.27125	597.92	6.29383	597.473	4.0555
596.178	3.27284	598.92	6.29236	598.473	4.05399
597.178	3.27182	599.92	6.29027	599.473	4.0512
598.178	3.26764	600.92	6.29	600.473	4.04769
599.178	3.26075	601.92	6.29431	601.473	4.04609
600.178	3.2535	602.92	6.29906	602.473	4.04776
601.178	3.24913	603.92	6.3002	603.473	4.05E+00
602.178	3.24914	604.92	6.29871	604.473	4.04663
603.178	3.24887	605.92	6.30134	605.473	4.04534
604.178	3.24515	606.92	6.30333	606.473	4.04174
605.178	3.24083	607.92	6.30989	607.473	4.0416
606.178	3.23836	608.92	6.31223	608.473	4.04468
607.178	3.23749	609.92	6.30423	609.473	4.04195
608.178	3.24167	610.92	6.29367	610.473	4.03532
609.178	3.24062	611.92	6.28723	611.473	4.03056
610.178	3.23246	612.92	6.28622	612.473	4.03207
611.178	3.2257	613.92	6.28339	613.473	4.03529
612.178	3.22473	614.92	6.27977	614.473	4.04066
613.178	3.22799	615.92	6.2715	615.473	4.04163
614.178	3.23093	616.92	6.2695	616.473	4.04054
615.178	3.23259	617.92	6.27357	617.473	4.04316
616.178	3.23001	618.92	6.27482	618.473	4.0422
617.178	3.23481	619.92	6.27E+00	619.473	4.03752
618.178	3.24138	620.92	6.27E+00	620.473	4.03069
619.178	3.24446	621.92	6.26812	621.473	4.02403
620.178	3.24478	622.92	6.26769	622.473	4.01625

621.178	3.24484	623.92	6.27239	623.473	4.01274
622.178	3.24389	624.92	6.27038	624.473	4.01147
623.178	3.24517	625.92	6.26589	625.473	4.00515
624.178	3.25036	626.92	6.26502	626.473	4.00383
625.178	3.24726	627.92	6.26318	627.473	4.00446
626.178	3.24607	628.92	6.26165	628.473	4.00457
627.178	3.24787	629.92	6.26303	629.473	4.00814
628.178	3.24748	630.92	6.26392	630.473	4.01566
629.178	3.2484	631.92	6.25862	631.473	4.01952
630.178	3.25208	632.92	6.25378	632.473	4.01936
631.178	3.25275	633.92	6.25066	633.473	4.02134
632.178	3.24733	634.92	6.24818	634.473	4.02204
633.178	3.2437	635.92	6.24707	635.473	4.02258
634.178	3.23984	636.92	6.24596	636.473	4.02337
635.178	3.23647	637.92	6.244	637.473	4.0206
636.178	3.23554	638.92	6.25E+00	638.473	4.01761
637.178	3.23367	639.92	6.25E+00	639.473	4.01652
638.178	3.23221	640.92	6.24533	640.473	4.01287
639.178	3.23E+00	641.92	6.24803	641.473	4.01196
640.178	3.23477	642.92	6.24899	642.473	4.01555
641.178	3.23495	643.92	6.24709	643.473	4.0206
642.178	3.23659	644.92	6.25087	644.473	4.02879
643.178	3.23794	645.92	6.24952	645.473	4.03765
644.178	3.24179	646.92	6.24165	646.473	4.03422
645.178	3.24992	647.92	6.23618	647.473	4.02739
646.178	3.2484	648.92	6.23464	648.473	4.02636
647.178	3.24048	649.92	6.23154	649.473	4.02516
648.178	3.23686	650.92	6.23E+00	650.473	4.02836
649.178	3.23667	651.92	6.23576	651.473	4.03138
650.178	3.23936	652.92	6.23426	652.473	4.03086
651.178	3.24483	653.92	6.23688	653.473	4.03294
652.178	3.24553	654.92	6.24107	654.473	4.04401
653.178	3.24481	655.92	6.23842	655.473	4.05387
654.178	3.25137	656.92	6.23212	656.473	4.05791
655.178	3.25947	657.92	6.22994	657.473	4.06039
656.178	3.26183	658.92	6.22631	658.473	4.06273
657.178	3.26199	659.92	6.22444	659.473	4.06447
658.178	3.26533	660.92	6.21965	660.473	4.06653
659.178	3.26692	661.92	6.21024	661.473	4.06182
660.178	3.27005	662.92	6.20327	662.473	4.05534
661.178	3.26786	663.92	6.20E+00	663.473	4.05341
662.178	3.26085	664.92	6.21097	664.473	4.05592
663.178	3.25602	665.92	6.21875	665.473	4.06372

664.178	3.25584	666.92	6.22436	666.473	4.06932
665.178	3.25871	667.92	6.22536	667.473	4.07134
666.178	3.26019	668.92	6.22356	668.473	4.06974
667.178	3.25848	669.92	6.22093	669.473	4.06586
668.178	3.25495	670.92	6.21727	670.473	4.06153
669.178	3.25142	671.92	6.21087	671.473	4.05649
670.178	3.24917	672.92	6.20197	672.473	4.05076
671.178	3.24734	673.92	6.18761	673.473	4.04029
672.178	3.24456	674.92	6.17416	674.473	4.02704
673.178	3.23792	675.92	6.16799	675.473	4.01699
674.178	3.2267	676.92	6.16696	676.473	4.01287
675.178	3.21741	677.92	6.16678	677.473	4.01325
676.178	3.21297	678.92	6.16528	678.473	4.01E+00
677.178	3.21098	679.92	6.16261	679.473	4.01517
678.178	3.20962	680.92	6.15983	680.473	4.0165
679.178	3.20712	681.92	6.15793	681.473	4.01617
680.178	3.20605	682.92	6.15756	682.473	4.01577
681.178	3.20602	683.92	6.15617	683.473	4.01603
682.178	3.2083	684.92	6.15	684.473	4.01571
683.178	3.21296	685.92	6.14125	685.473	4.00972
684.178	3.21782	686.92	6.13493	686.473	4.00335
685.178	3.21807	687.92	6.13296	687.473	4.00178
686.178	3.21652	688.92	6.13294	688.473	4.0037
687.178	3.21815	689.92	6.13232	689.473	4.00773
688.178	3.22189	690.92	6.12884	690.473	4.01206
689.178	3.22644	691.92	6.12412	691.473	4.01E+00
690.178	3.22952	692.92	6.1228	692.473	4.01235
691.178	3.229	693.92	6.13E+00	693.473	4.01527
692.178	3.22663	694.92	6.12889	694.473	4.02094
693.178	3.22728	695.92	6.12996	695.473	4.02374
694.178	3.23E+00	696.92	6.13	696.473	4.02296
695.178	3.23106	697.92	6.13089	697.473	4.02173
696.178	3.2304	698.92	6.13495	698.473	4.02351
697.178	3.22915	699.92	6.13916	699.473	4.02732
698.178	3.23081	700.92	6.14401	700.473	4.03317
699.178	3.2363	701.92	6.1457	701.473	4.03908
700.178	3.24223	702.92	6.14473	702.473	4.03882
701.178	3.24829	703.92	6.15E+00	703.473	4.03636
702.178	3.25106	704.92	6.14866	704.473	4.03727
703.178	3.25267	705.92	6.15005	705.473	4.03897
704.178	3.25675	706.92	6.14542	706.473	4.0382
705.178	3.26155	707.92	6.14	707.473	4.03685
706.178	3.26191	708.92	6.13209	708.473	4.03511

707.178	3.25646	709.92	6.12546	709.473	4.03054
708.178	3.24986	710.92	6.12599	710.473	4.0331
709.178	3.24107	711.92	6.12607	711.473	4.04126
710.178	3.23813	712.92	6.11922	712.473	4.0443
711.178	3.24177	713.92	6.11261	713.473	4.04406
712.178	3.24163	714.92	6.10891	714.473	4.04348
713.178	3.2362	715.92	6.10574	715.473	4.0418
714.178	3.23188	716.92	6.10406	716.473	4.0413
715.178	3.22935	717.92	6.10434	717.473	4.04459
716.178	3.22738	718.92	6.10285	718.473	4.04599
717.178	3.22691	719.92	6.10121	719.473	4.04585
718.178	3.22498	720.92	6.10116	720.473	4.04698
719.178	3.22125	721.92	6.10155	721.473	4.04742
720.178	3.22073	722.92	6.09951	722.473	4.04473
721.178	3.22199	723.92	6.0976	723.473	4.04004
722.178	3.22159	724.92	6.09737	724.473	4.03842
723.178	3.22E+00	725.92	6.09581	725.473	4.0376
724.178	3.21759	726.92	6.09195	726.473	4.037
725.178	3.21983	727.92	6.08615	727.473	4.03369
726.178	3.22192	728.92	6.0804	728.473	4.02818
727.178	3.22079	729.92	6.07622	729.473	4.02551
728.178	3.21729	730.92	6.07733	730.473	4.02803
729.178	3.21585	731.92	6.07959	731.473	4.03559
730.178	3.21883	732.92	6.07713	732.473	4.03757
731.178	3.2285	733.92	6.0751	733.473	4.03597
732.178	3.23547	734.92	6.07324	734.473	4.03412
733.178	3.23702	735.92	6.07225	735.473	4.0323
734.178	3.23746	736.92	6.07282	736.473	4.03251
735.178	3.23768	737.92	6.07296	737.473	4.03E+00
736.178	3.24E+00	738.92	6.07316	738.473	4.03338
737.178	3.23621	739.92	6.07207	739.473	4.03E+00
738.178	3.23446	740.92	6.07329	740.473	4.03532
739.178	3.23333	741.92	6.07722	741.473	4.03932
740.178	3.23414	742.92	6.08021	742.473	4.04357
741.178	3.2384	743.92	6.08E+00	743.473	4.04654
742.178	3.2446	744.92	6.08147	744.473	4.04838
743.178	3.2504	745.92	6.08127	745.473	4.04966
744.178	3.25518	746.92	6.07789	746.473	4.04922
745.178	3.26018	747.92	6.07184	747.473	4.04531
746.178	3.26458	748.92	6.06509	748.473	4.04101
747.178	3.26471	749.92	6.06035	749.473	4.04E+00
748.178	3.26E+00	750.92	6.0578	750.473	4.03998
749.178	3.25662	751.92	6.05743	751.473	4.04244

750.178	3.25525	752.92	6.05767	752.473	4.04613
751.178	3.25483	753.92	6.05852	753.473	4.05036
752.178	3.25594	754.92	6.06026	754.473	4.05539
753.178	3.25746	755.92	6.06434	755.473	4.06122
754.178	3.25956	756.92	6.07037	756.473	4.06751
755.178	3.26235	757.92	6.07299	757.473	4.07226
756.178	3.26675	758.92	6.07178	758.473	4.07349
757.178	3.27142	759.92	6.07144	759.473	4.07447
758.178	3.27246	760.92	6.0733	760.473	4.07545
759.178	3.27262	761.92	6.07619	761.473	4.07637
760.178	3.2728	762.92	6.07838	762.473	4.07705
761.178	3.27298	763.92	6.07925	763.473	4.07777
762.178	3.27E+00	764.92	6.07975	764.473	4.07871
763.178	3.27E+00	765.92	6.08112	765.473	4.0795
764.178	3.27E+00	766.92	6.08644	766.473	4.08056
765.178	3.27E+00	767.92	6.092	767.473	4.08192
766.178	3.27309	768.92	6.09433	768.473	4.08342
767.178	3.27331	769.92	6.09397	769.473	4.08521
768.178	3.27378	770.92	6.0925	770.473	4.08729
769.178	3.27E+00	771.92	6.0918	771.473	4.08938
770.178	3.27541	772.92	6.09183	772.473	4.08889
771.178	3.27652	773.92	6.09E+00	773.473	4.08803
772.178	3.27575	774.92	6.10E+00	774.473	4.09203
773.178	3.2737	775.92	6.09967	775.473	4.09454
774.178	3.28E+00	776.92	6.09712	776.473	4.09373
775.178	3.27843	777.92	6.09451	777.473	4.09214
776.178	3.27806	778.92	6.0912	778.473	4.08951
777.178	3.27665	779.92	6.08672	779.473	4.08414
778.178	3.27492	780.92	6.08511	780.473	4.08035
779.178	3.27061	781.92	6.084	781.473	4.08158
780.178	3.26676	782.92	6.07436	782.473	4.07839
781.178	3.26888	783.92	6.06576	783.473	4.07529
782.178	3.26996	784.92	6.05924	784.473	4.07176
783.178	3.26826	785.92	6.05731	785.473	4.07075
784.178	3.26688	786.92	6.05786	786.473	4.0721
785.178	3.26313	787.92	6.06023	787.473	4.07602
786.178	3.26123	788.92	6.05965	788.473	4.08045
787.178	3.26035	789.92	6.05519	789.473	4.08026
788.178	3.26014	790.92	6.05332	790.473	4.07924
789.178	3.25557	791.92	6.05354	791.473	4.07841
790.178	3.24874	792.92	6.05587	792.473	4.08108
791.178	3.24196	793.92	6.05519	793.473	4.08E+00
792.178	3.23924	794.92	6.05328	794.473	4.08155

793.178	3.24146	795.92	6.05032	795.473	4.0803
794.178	3.24069	796.92	6.04742	796.473	4.07905
795.178	3.24278				
796.178	3.24813				
797.178	3.25352				

temperature	*TG*	*temperature*	*TG*
℃	%	℃	%
	Coal+TPPI		Coal+PA
25.93	100	25.697	100
26.93	99.86463	26.697	99.86066
27.93	99.67144	27.697	99.65965
28.93	99.43928	28.697	99.43382
29.93	99.20568	29.697	99.2092
30.93	98.99822	30.697	99.01851
31.93	98.81751	31.697	98.84167
32.93	98.66945	32.697	98.69317
33.93	98.54532	33.697	98.5692
34.93	98.42242	34.697	98.43388
35.93	98.30774	35.697	98.30669
36.93	98.19764	36.697	98.20015
37.93	98.09467	37.697	98.09409
38.93	98.00486	38.697	97.99723
39.93	97.91837	39.697	97.89731
40.93	97.83543	40.697	97.80646
41.93	97.7649	41.697	97.72845
42.93	97.69294	42.697	97.64788
43.93	97.61635	43.697	97.56278
44.93	97.53908	44.697	97.47446
45.93	97.46505	45.697	97.38996
46.93	97.39409	46.697	97.30738
47.93	97.31647	47.697	97.21966
48.93	97.24432	48.697	97.13032
49.93	97.17088	49.697	97.03798
50.93	97.09902	50.697	96.95057
51.93	97.03635	51.697	96.87133
52.93	96.97376	52.697	96.79596
53.93	96.9094	53.697	96.71585
54.93	96.84085	54.697	96.63382
55.93	96.76762	55.697	96.54715
56.93	96.70339	56.697	96.4673
57.93	96.64119	57.697	96.39167
58.93	96.57225	58.697	96.31587
59.93	96.5077	59.697	96.23982
60.93	96.44409	60.697	96.15846
61.93	96.37967	61.697	96.0806
62.93	96.32027	62.697	95.99921
63.93	96.26703	63.697	95.92918
64.93	96.21148	64.697	95.85818

65.93	96.1516	65.697	95.7854
66.93	96.09339	66.697	95.7127
67.93	96.0351	67.697	95.64135
68.93	95.9788	68.697	95.57055
69.93	95.91679	69.697	95.49631
70.93	95.85479	70.697	95.42083
71.93	95.80497	71.697	95.35394
72.93	95.74653	72.697	95.29025
73.93	95.68017	73.697	95.20677
74.93	95.633	74.697	95.13917
75.93	95.592	75.697	95.08337
76.93	95.54489	76.697	95.02499
77.93	95.49264	77.697	94.95983
78.93	95.43516	78.697	94.88787
79.93	95.39599	79.697	94.82537
80.93	95.36701	80.697	94.78315
81.93	95.31415	81.697	94.7215
82.93	95.26834	82.697	94.65395
83.93	95.23163	83.697	94.60247
84.93	95.18209	84.697	94.54379
85.93	95.13223	85.697	94.47745
86.93	95.08635	86.697	94.41681
87.93	95.04939	87.697	94.36356
88.93	95.01858	88.697	94.31689
89.93	94.97218	89.697	94.26294
90.93	94.92126	90.697	94.19646
91.93	94.8823	91.697	94.14462
92.93	94.84514	92.697	94.10023
93.93	94.80475	93.697	94.05189
94.93	94.76335	94.697	94.00064
95.93	94.72479	95.697	93.94858
96.93	94.68624	96.697	93.89884
97.93	94.64734	97.697	93.85164
98.93	94.60971	98.697	93.80784
99.93	94.57304	99.697	93.76232
100.93	94.53781	100.697	93.71927
101.93	94.5014	101.697	93.67268
102.93	94.46768	102.697	93.62479
103.93	94.43708	103.697	93.58357
104.93	94.40321	104.697	93.54065
105.93	94.37025	105.697	93.49385
106.93	94.33345	106.697	93.44149
107.93	94.2985	107.697	93.39147

108.93	94.26349	108.697	93.34224
109.93	94.22872	109.697	93.2937
110.93	94.1962	110.697	93.25183
111.93	94.16298	111.697	93.21599
112.93	94.12279	112.697	93.1702
113.93	94.08361	113.697	93.12287
114.93	94.04597	114.697	93.07819
115.93	94.0152	115.697	93.03724
116.93	93.98355	116.697	92.99691
117.93	93.95302	117.697	92.95739
118.93	93.92359	118.697	92.92103
119.93	93.88763	119.697	92.87567
120.93	93.8504	120.697	92.82587
121.93	93.81742	121.697	92.778
122.93	93.7865	122.697	92.73525
123.93	93.7557	123.697	92.69453
124.93	93.71975	124.697	92.65405
125.93	93.6809	125.697	92.61264
126.93	93.64034	126.697	92.5677
127.93	93.60254	127.697	92.52264
128.93	93.56873	128.697	92.48091
129.93	93.53715	129.697	92.44118
130.93	93.50692	130.697	92.40499
131.93	93.47294	131.697	92.36565
132.93	93.43746	132.697	92.32616
133.93	93.40839	133.697	92.28891
134.93	93.38351	134.697	92.2526
135.93	93.35986	135.697	92.21925
136.93	93.32993	136.697	92.18117
137.93	93.30348	137.697	92.14925
138.93	93.27443	138.697	92.11516
139.93	93.24488	139.697	92.08194
140.93	93.21733	140.697	92.05165
141.93	93.18725	141.697	92.01875
142.93	93.15423	142.697	91.98194
143.93	93.11314	143.697	91.93825
144.93	93.07975	144.697	91.90108
145.93	93.05134	145.697	91.8681
146.93	93.02111	146.697	91.82994
147.93	92.98246	147.697	91.79052
148.93	92.94989	148.697	91.75034
149.93	92.92223	149.697	91.71683
150.93	92.88806	150.697	91.67931

151.93	92.85454	151.697	91.64112
152.93	92.82363	152.697	91.60892
153.93	92.78608	153.697	91.57061
154.93	92.74912	154.697	91.53031
155.93	92.72181	155.697	91.49636
156.93	92.69791	156.697	91.4702
157.93	92.6642	157.697	91.43497
158.93	92.63584	158.697	91.39938
159.93	92.61162	159.697	91.3732
160.93	92.5881	160.697	91.34902
161.93	92.55318	161.697	91.31501
162.93	92.52038	162.697	91.28022
163.93	92.48501	163.697	91.24547
164.93	92.45081	164.697	91.2081
165.93	92.42066	165.697	91.17469
166.93	92.3908	166.697	91.14518
167.93	92.36225	167.697	91.11967
168.93	92.33204	168.697	91.0903
169.93	92.31174	169.697	91.06429
170.93	92.29739	170.697	91.04721
171.93	92.27764	171.697	91.02974
172.93	92.25978	172.697	91.01118
173.93	92.23481	173.697	90.98997
174.93	92.20858	174.697	90.96668
175.93	92.18182	175.697	90.94092
176.93	92.15751	176.697	90.91598
177.93	92.13332	177.697	90.89334
178.93	92.1093	178.697	90.87363
179.93	92.08903	179.697	90.85622
180.93	92.06976	180.697	90.84029
181.93	92.05028	181.697	90.82338
182.93	92.03008	182.697	90.80531
183.93	92.01182	183.697	90.78719
184.93	91.99613	184.697	90.77413
185.93	91.97422	185.697	90.75541
186.93	91.95442	186.697	90.73571
187.93	91.93571	187.697	90.71678
188.93	91.91069	188.697	90.69034
189.93	91.88772	189.697	90.66578
190.93	91.87074	190.697	90.6516
191.93	91.84927	191.697	90.6352
192.93	91.82836	192.697	90.61494
193.93	91.80902	193.697	90.59636

194.93	91.78899	194.697	90.58159
195.93	91.7661	195.697	90.56324
196.93	91.74725	196.697	90.54966
197.93	91.72788	197.697	90.53866
198.93	91.70722	198.697	90.52168
199.93	91.68963	199.697	90.50629
200.93	91.6623	200.697	90.48547
201.93	91.6421	201.697	90.46993
202.93	91.63014	202.697	90.45893
203.93	91.62003	203.697	90.45212
204.93	91.60483	204.697	90.44113
205.93	91.59214	205.697	90.42511
206.93	91.58593	206.697	90.415
207.93	91.57862	207.697	90.4026
208.93	91.57481	208.697	90.39263
209.93	91.56708	209.697	90.37925
210.93	91.55478	210.697	90.36025
211.93	91.53941	211.697	90.33614
212.93	91.5265	212.697	90.32027
213.93	91.51652	213.697	90.31058
214.93	91.50046	214.697	90.29837
215.93	91.48522	215.697	90.28607
216.93	91.47088	216.697	90.27548
217.93	91.46363	217.697	90.27108
218.93	91.45559	218.697	90.2674
219.93	91.45006	219.697	90.26611
220.93	91.44262	220.697	90.25502
221.93	91.43581	221.697	90.24561
222.93	91.42662	222.697	90.23548
223.93	91.42136	223.697	90.22846
224.93	91.41683	224.697	90.22117
225.93	91.40969	225.697	90.21307
226.93	91.40204	226.697	90.20428
227.93	91.38385	227.697	90.19206
228.93	91.36779	228.697	90.17719
229.93	91.36697	229.697	90.17449
230.93	91.3632	230.697	90.16494
231.93	91.35606	231.697	90.155
232.93	91.34881	232.697	90.14776
233.93	91.33967	233.697	90.13374
234.93	91.32985	234.697	90.11813
235.93	91.32474	235.697	90.10428
236.93	91.32528	236.697	90.10034

237.93	91.30891	237.697	90.08294
238.93	91.2975	238.697	90.0694
239.93	91.28611	239.697	90.05712
240.93	91.27487	240.697	90.04233
241.93	91.25778	241.697	90.03037
242.93	91.24446	242.697	90.01948
243.93	91.23672	243.697	90.00972
244.93	91.22608	244.697	90.00293
245.93	91.21087	245.697	89.99409
246.93	91.2	246.697	89.98392
247.93	91.19054	247.697	89.97201
248.93	91.17262	248.697	89.95348
249.93	91.16015	249.697	89.93425
250.93	91.15294	250.697	89.91802
251.93	91.14018	251.697	89.89914
252.93	91.12388	252.697	89.87453
253.93	91.11182	253.697	89.85409
254.93	91.0888	254.697	89.8275
255.93	91.0616	255.697	89.79747
256.93	91.04223	256.697	89.77219
257.93	91.02485	257.697	89.75359
258.93	91.00004	258.697	89.73357
259.93	90.96798	259.697	89.70438
260.93	90.93895	260.697	89.67397
261.93	90.91317	261.697	89.64497
262.93	90.89867	262.697	89.62464
263.93	90.88081	263.697	89.60473
264.93	90.85019	264.697	89.57396
265.93	90.81908	265.697	89.53922
266.93	90.7894	266.697	89.50465
267.93	90.7603	267.697	89.47299
268.93	90.73478	268.697	89.44436
269.93	90.7064	269.697	89.41435
270.93	90.66531	270.697	89.37791
271.93	90.62665	271.697	89.33647
272.93	90.59076	272.697	89.2965
273.93	90.55384	273.697	89.2581
274.93	90.51202	274.697	89.21849
275.93	90.47405	275.697	89.17914
276.93	90.42935	276.697	89.13564
277.93	90.3801	277.697	89.08484
278.93	90.33163	278.697	89.03138
279.93	90.28128	279.697	88.98212

280.93	90.23483	280.697	88.93449
281.93	90.18555	281.697	88.88161
282.93	90.13691	282.697	88.82858
283.93	90.08786	283.697	88.77228
284.93	90.03016	284.697	88.70716
285.93	89.97115	285.697	88.63982
286.93	89.91588	286.697	88.57539
287.93	89.86099	287.697	88.51396
288.93	89.80118	288.697	88.45016
289.93	89.74256	289.697	88.38974
290.93	89.67463	290.697	88.31836
291.93	89.60867	291.697	88.24122
292.93	89.55278	292.697	88.18067
293.93	89.47954	293.697	88.11313
294.93	89.39802	294.697	88.03061
295.93	89.33015	295.697	87.95446
296.93	89.26178	296.697	87.87911
297.93	89.18221	297.697	87.78858
298.93	89.10346	298.697	87.69922
299.93	89.01708	299.697	87.61121
300.93	88.92665	300.697	87.51582
301.93	88.85301	301.697	87.42318
302.93	88.78089	302.697	87.33811
303.93	88.67693	303.697	87.23857
304.93	88.57022	304.697	87.1309
305.93	88.48087	305.697	87.03652
306.93	88.39035	306.697	86.94229
307.93	88.29905	307.697	86.83917
308.93	88.20247	308.697	86.73364
309.93	88.10242	309.697	86.62997
310.93	87.99419	310.697	86.52143
311.93	87.89602	311.697	86.41207
312.93	87.79936	312.697	86.30234
313.93	87.68927	313.697	86.18249
314.93	87.57782	314.697	86.06115
315.93	87.45939	315.697	85.94083
316.93	87.33906	316.697	85.81748
317.93	87.21292	317.697	85.68577
318.93	87.09113	318.697	85.55335
319.93	86.96903	319.697	85.41683
320.93	86.84602	320.697	85.28136
321.93	86.71773	321.697	85.14221
322.93	86.59267	322.697	84.99975

323.93	86.46215	323.697	84.85717
324.93	86.32897	324.697	84.7096
325.93	86.20057	325.697	84.56223
326.93	86.06789	326.697	84.41556
327.93	85.92861	327.697	84.27008
328.93	85.78456	328.697	84.12306
329.93	85.64132	329.697	83.9757
330.93	85.50106	330.697	83.82199
331.93	85.36052	331.697	83.66268
332.93	85.21197	332.697	83.50527
333.93	85.06425	333.697	83.34618
334.93	84.91121	334.697	83.18514
335.93	84.76489	335.697	83.0204
336.93	84.61077	336.697	82.84741
337.93	84.45163	337.697	82.67192
338.93	84.28846	338.697	82.49808
339.93	84.1212	339.697	82.32533
340.93	83.95141	340.697	82.1471
341.93	83.79062	341.697	81.96578
342.93	83.63212	342.697	81.78121
343.93	83.46495	343.697	81.59342
344.93	83.28641	344.697	81.40544
345.93	83.11009	345.697	81.21314
346.93	82.93044	346.697	81.01517
347.93	82.75559	347.697	80.81968
348.93	82.58078	348.697	80.6253
349.93	82.39712	349.697	80.42637
350.93	82.19821	350.697	80.21501
351.93	82.00421	351.697	79.9998
352.93	81.81822	352.697	79.79
353.93	81.62864	353.697	79.58311
354.93	81.44063	354.697	79.3686
355.93	81.23957	355.697	79.14493
356.93	81.03652	356.697	78.91736
357.93	80.83223	357.697	78.68472
358.93	80.63052	358.697	78.45457
359.93	80.42005	359.697	78.22647
360.93	80.20761	360.697	77.99503
361.93	80.00176	361.697	77.75661
362.93	79.7853	362.697	77.51411
363.93	79.56404	363.697	77.26855
364.93	79.33369	364.697	77.02203
365.93	79.1052	365.697	76.77045

366.93	78.88019	366.697	76.51336
367.93	78.66249	367.697	76.24702
368.93	78.41727	368.697	75.97751
369.93	78.16047	369.697	75.69498
370.93	77.91076	370.697	75.41402
371.93	77.67356	371.697	75.13282
372.93	77.43973	372.697	74.84888
373.93	77.19205	373.697	74.55886
374.93	76.92613	374.697	74.26231
375.93	76.65937	375.697	73.97034
376.93	76.39719	376.697	73.67196
377.93	76.13897	377.697	73.35803
378.93	75.86746	378.697	73.04534
379.93	75.58491	379.697	72.72521
380.93	75.30236	380.697	72.40325
381.93	75.01333	381.697	72.06769
382.93	74.71605	382.697	71.72636
383.93	74.42544	383.697	71.38684
384.93	74.13279	384.697	71.04187
385.93	73.83023	385.697	70.68934
386.93	73.51319	386.697	70.31885
387.93	73.19483	387.697	69.94357
388.93	72.87368	388.697	69.56187
389.93	72.55864	389.697	69.18556
390.93	72.2417	390.697	68.80274
391.93	71.90894	391.697	68.40405
392.93	71.56631	392.697	67.99531
393.93	71.22937	393.697	67.58303
394.93	70.90388	394.697	67.17969
395.93	70.55793	395.697	66.76359
396.93	70.19662	396.697	66.32648
397.93	69.83009	397.697	65.87775
398.93	69.45873	398.697	65.41506
399.93	69.08812	399.697	64.94893
400.93	68.71134	400.697	64.46648
401.93	68.33495	401.697	63.97896
402.93	67.93545	402.697	63.47341
403.93	67.53694	403.697	62.95166
404.93	67.14323	404.697	62.42546
405.93	66.72579	405.697	61.88305
406.93	66.30499	406.697	61.31927
407.93	65.88861	407.697	60.75735
408.93	65.45901	408.697	60.17874

409.93	65.02251	409.697	59.58027
410.93	64.57839	410.697	58.95854
411.93	64.10924	411.697	58.31839
412.93	63.64165	412.697	57.67281
413.93	63.18005	413.697	57.01948
414.93	62.69537	414.697	56.34874
415.93	62.18868	415.697	55.64781
416.93	61.68136	416.697	54.93215
417.93	61.15755	417.697	54.19426
418.93	60.62712	418.697	53.44362
419.93	60.11033	419.697	52.68031
420.93	59.58231	420.697	51.90171
421.93	59.01087	421.697	51.08407
422.93	58.44029	422.697	50.23342
423.93	57.8708	423.697	49.37841
424.93	57.28049	424.697	48.48759
425.93	56.68005	425.697	47.57913
426.93	56.09206	426.697	46.65378
427.93	55.47882	427.697	45.69212
428.93	54.84339	428.697	44.72399
429.93	54.20307	429.697	43.71213
430.93	53.55709	430.697	42.67203
431.93	52.89037	431.697	41.63714
432.93	52.2119	432.697	40.53734
433.93	51.52121	433.697	39.41027
434.93	50.81362	434.697	38.2685
435.93	50.09262	435.697	37.10724
436.93	49.37682	436.697	35.90688
437.93	48.64833	437.697	34.70494
438.93	47.9233	438.697	33.46798
439.93	47.19582	439.697	32.21787
440.93	46.43758	440.697	30.97516
441.93	45.69269	441.697	29.71191
442.93	44.9241	442.697	28.42101
443.93	44.15696	443.697	27.13938
444.93	43.37099	444.697	25.8602
445.93	42.58929	445.697	24.57571
446.93	41.79848	446.697	23.28698
447.93	41.00611	447.697	22.02565
448.93	40.20067	448.697	20.75906
449.93	39.39626	449.697	19.53723
450.93	38.59924	450.697	18.32625
451.93	37.80125	451.697	17.15026

452.93	37.01113	452.697	16.00669
453.93	36.21793	453.697	14.90703
454.93	35.41549	454.697	13.86068
455.93	34.62775	455.697	12.86998
456.93	33.84952	456.697	11.93446
457.93	33.05024	457.697	11.05977
458.93	32.26715	458.697	10.2374
459.93	31.48114	459.697	9.47911
460.93	30.69254	460.697	8.77111
461.93	29.92659	461.697	8.11381
462.93	29.14313	462.697	7.5041
463.93	28.37346	463.697	6.93079
464.93	27.60515	464.697	6.41746
465.93	26.85305	465.697	5.94675
466.93	26.11656	466.697	5.50035
467.93	25.38929	467.697	5.09344
468.93	24.66056	468.697	4.7186
469.93	23.94293	469.697	4.37875
470.93	23.23476	470.697	4.06255
471.93	22.55269	471.697	3.78193
472.93	21.87842	472.697	3.52993
473.93	21.23322	473.697	3.29846
474.93	20.59392	474.697	3.09399
475.93	19.97633	475.697	2.90356
476.93	19.37005	476.697	2.73001
477.93	18.79268	477.697	2.57568
478.93	18.21634	478.697	2.44028
479.93	17.67107	479.697	2.31543
480.93	17.14931	480.697	2.19692
481.93	16.62424	481.697	2.0843
482.93	16.12121	482.697	1.97963
483.93	15.65126	483.697	1.88764
484.93	15.20553	484.697	1.80524
485.93	14.76388	485.697	1.72276
486.93	14.34021	486.697	1.64165
487.93	13.93181	487.697	1.56732
488.93	13.52573	488.697	1.50564
489.93	13.15979	489.697	1.45284
490.93	12.81268	490.697	1.40684
491.93	12.48161	491.697	1.36226
492.93	12.16236	492.697	1.31702
493.93	11.86086	493.697	1.27663
494.93	11.57712	494.697	1.24211

495.93	11.31297	495.697	1.20618
496.93	11.05599	496.697	1.16526
497.93	10.81752	497.697	1.13083
498.93	10.57985	498.697	1.09519
499.93	10.34984	499.697	1.05891
500.93	10.13864	500.697	1.0267
501.93	9.93556	501.697	0.99485
502.93	9.75408	502.697	0.9667
503.93	9.57516	503.697	0.94546
504.93	9.41295	504.697	0.92598
505.93	9.24844	505.697	0.89629
506.93	9.09573	506.697	0.86683
507.93	8.94713	507.697	0.84134
508.93	8.80734	508.697	0.82165
509.93	8.67017	509.697	0.80301
510.93	8.53851	510.697	0.77778
511.93	8.41977	511.697	0.75589
512.93	8.31	512.697	0.73829
513.93	8.20183	513.697	0.71893
514.93	8.09906	514.697	0.6994
515.93	8.00803	515.697	0.68475
516.93	7.91844	516.697	0.66986
517.93	7.83583	517.697	0.65588
518.93	7.75417	518.697	0.63765
519.93	7.6744	519.697	0.61172
520.93	7.60031	520.697	0.58714
521.93	7.53921	521.697	0.57749
522.93	7.48182	522.697	0.5694
523.93	7.42308	523.697	0.55669
524.93	7.36526	524.697	0.54037
525.93	7.30976	525.697	0.52304
526.93	7.26115	526.697	0.51634
527.93	7.21485	527.697	0.5142
528.93	7.17535	528.697	0.51423
529.93	7.13161	529.697	0.50185
530.93	7.0909	530.697	0.48986
531.93	7.05536	531.697	0.48119
532.93	7.02125	532.697	0.47005
533.93	6.98872	533.697	0.46014
534.93	6.95688	534.697	0.45061
535.93	6.92952	535.697	0.4455
536.93	6.8978	536.697	0.43338
537.93	6.87098	537.697	0.42147

538.93	6.83746	538.697	0.40819
539.93	6.80525	539.697	0.3959
540.93	6.77857	540.697	0.39018
541.93	6.7555	541.697	0.38588
542.93	6.73718	542.697	0.38035
543.93	6.72061	543.697	0.37305
544.93	6.70065	544.697	0.36438
545.93	6.68001	545.697	0.35872
546.93	6.66787	546.697	0.3571
547.93	6.65295	547.697	0.35324
548.93	6.63861	548.697	0.34388
549.93	6.62205	549.697	0.33304
550.93	6.60625	550.697	0.31834
551.93	6.5892	551.697	0.3036
552.93	6.57811	552.697	0.29443
553.93	6.56252	553.697	0.28465
554.93	6.54615	554.697	0.27136
555.93	6.53521	555.697	0.25534
556.93	6.52125	556.697	0.23908
557.93	6.5123	557.697	0.2255
558.93	6.50526	558.697	0.22157
559.93	6.4978	559.697	0.21983
560.93	6.4845	560.697	0.21441
561.93	6.47755	561.697	0.2065
562.93	6.47279	562.697	0.1987
563.93	6.4684	563.697	0.19828
564.93	6.46491	564.697	0.19907
565.93	6.45795	565.697	0.19578
566.93	6.44555	566.697	0.18561
567.93	6.43091	567.697	0.17098
568.93	6.42076	568.697	0.15794
569.93	6.41558	569.697	0.15034
570.93	6.41028	570.697	0.14482
571.93	6.40298	571.697	0.13462
572.93	6.39537	572.697	0.12094
573.93	6.38813	573.697	0.10847
574.93	6.38256	574.697	0.0957163
575.93	6.38589	575.697	0.0851198
576.93	6.39178	576.697	0.07937433
577.93	6.39144	577.697	0.07074291
578.93	6.39276	578.697	0.06466443
579.93	6.39673	579.697	0.06206871
5.81E+02	6.40255	580.697	0.06333557

581.93	6.40984	581.697	0.06670986
582.93	6.41169	582.697	0.06884973
583.93	6.4048	583.697	0.06805702
584.93	6.39271	584.697	0.06053823
585.93	6.38309	585.697	0.05404909
586.93	6.36998	586.697	0.04525793
587.93	6.35444	587.697	0.03375819
588.93	6.34003	588.697	0.01955958
589.93	6.32615	589.697	0.0069455
590.93	6.31646	590.697	-0.00416835
591.93	6.31805	591.697	-0.00818021
592.93	6.32252	592.697	-0.00782463
593.93	6.322	593.697	-0.00805894
594.93	6.32055	594.697	-0.01407217
595.93	6.32036	595.697	-0.0220255
596.93	6.31836	596.697	-0.02657753
597.93	6.31204	597.697	-0.03388541
598.93	6.30561	598.697	-0.04273362
599.93	6.29372	599.697	-0.05402433
600.93	6.28088	600.697	-0.06731951
601.93	6.27471	601.697	-0.07730021
602.93	6.27212	602.697	-0.08147027
603.93	6.26698	603.697	-0.08301379
604.93	6.25668	604.697	-0.0872512
605.93	6.25053	605.697	-0.08877532
606.93	6.24638	606.697	-0.09223206
607.93	6.2496	607.697	-0.09135149
608.93	6.24802	608.697	-0.09341988
609.93	6.23771	609.697	-0.10482
610.93	6.22509	610.697	-0.11756
611.93	6.21837	611.697	-0.12669
612.93	6.22161	612.697	-0.13301
613.93	6.22465	613.697	-0.14551
614.93	6.22434	614.697	-0.15264
615.93	6.21563	615.697	-0.16248
616.93	6.21467	616.697	-0.16658
617.93	6.21838	617.697	-0.16264
618.93	6.21967	618.697	-0.16035
619.93	6.21699	619.697	-0.16454
620.93	6.21273	620.697	-0.17116
621.93	6.20772	621.697	-0.1713
622.93	6.2055	622.697	-0.1713
623.93	6.20906	623.697	-0.16974

624.93	6.20383	624.697	-0.17717
625.93	6.19592	625.697	-0.18671
626.93	6.18984	626.697	-0.19133
627.93	6.18414	627.697	-0.19276
628.93	6.18	628.697	-0.19135
629.93	6.17673	629.697	-0.18756
630.93	6.17138	630.697	-0.18697
631.93	6.15997	631.697	-0.1952
632.93	6.15159	632.697	-0.20062
633.93	6.14572	633.697	-0.20665
634.93	6.14125	634.697	-0.214
635.93	6.13648	635.697	-0.22048
636.93	6.12954	636.697	-0.22638
637.93	6.12335	637.697	-0.23262
638.93	6.12172	638.697	-0.23304
639.93	6.11992	639.697	-0.2288
640.93	6.11662	640.697	-0.22625
641.93	6.11475	641.697	-0.2262
642.93	6.11156	642.697	-0.22761
643.93	6.10999	643.697	-0.22909
644.93	6.11472	644.697	-0.22288
645.93	6.11368	645.697	-0.21982
646.93	6.10163	646.697	-0.22957
647.93	6.09093	6.48E+02	-0.24047
648.93	6.08523	648.697	-0.24498
649.93	6.0817	649.697	-0.24444
650.93	6.08171	650.697	-0.23678
651.93	6.07742	651.697	-0.23057
652.93	6.06969	652.697	-0.23129
653.93	6.06721	653.697	-0.229
654.93	6.07063	654.697	-0.21986
655.93	6.06864	655.697	-0.21443
656.93	6.06197	656.697	-0.21445
657.93	6.05841	657.697	-0.21189
658.93	6.05583	658.697	-0.21035
659.93	6.05626	659.697	-0.20989
660.93	6.05278	660.697	-0.21418
661.93	6.0436	661.697	-0.22681
662.93	6.03617	662.697	-0.23901
663.93	6.03491	663.697	-0.24232
664.93	6.03365	664.697	-0.24191
665.93	6.0307	665.697	-0.24211
666.93	6.02471	666.697	-0.24826

667.93	6.01705	667.697	-0.25644
668.93	6.00664	668.697	-0.26368
669.93	5.99471	669.697	-0.26783
670.93	5.98238	670.697	-0.27109
671.93	5.97096	671.697	-0.27742
672.93	5.96167	672.697	-0.28415
673.93	5.94993	673.697	-0.29474
674.93	5.93656	674.697	-0.30554
675.93	5.92504	675.697	-0.31203
676.93	5.91913	676.697	-0.31172
677.93	5.9168	677.697	-0.30962
678.93	5.91317	678.697	-0.30986
679.93	5.90791	679.697	-0.31393
680.93	5.9016	680.697	-0.31771
681.93	5.89411	681.697	-0.31874
682.93	5.88895	682.697	-0.31694
683.93	5.88459	6.84E+02	-0.3141
684.93	5.87629	684.697	-0.31332
685.93	5.86447	685.697	-0.31736
686.93	5.85522	686.697	-0.319
687.93	5.84954	687.697	-0.31402
688.93	5.84364	6.89E+02	-0.31124
689.93	5.83683	689.697	-0.31148
690.93	5.82891	690.697	-0.31004
691.93	5.81941	691.697	-0.30957
692.93	5.81116	692.697	-0.31274
693.93	5.80643	6.94E+02	-0.31316
694.93	5.80138	694.697	-0.31433
695.93	5.79421	695.697	-0.31689
696.93	5.78725	696.697	-0.31577
697.93	5.78335	697.697	-0.3107
698.93	5.78147	698.697	-0.30474
699.93	5.77805	699.697	-0.30286
700.93	5.77354	700.697	-0.2978
701.93	5.76473	701.697	-0.29052
702.93	5.753	702.697	-0.28692
703.93	5.74209	703.697	-0.29037
704.93	5.73587	704.697	-0.293
705.93	5.7289	705.697	-0.29836
706.93	5.71794	706.697	-0.30831
707.93	5.70517	707.697	-0.31566
708.93	5.69161	708.697	-0.32362
709.93	5.68149	709.697	-0.33453

710.93	5.6783	710.697	-0.33681
711.93	5.67459	711.697	-0.32912
712.93	5.66337	712.697	-0.32589
713.93	5.65184	713.697	-0.32309
714.93	5.64202	714.697	-0.32146
715.93	5.63643	715.697	-0.32123
716.93	5.63297	7.17E+02	-0.32081
717.93	5.62909	717.697	-0.3165
718.93	5.62354	718.697	-0.31738
719.93	5.62098	719.697	-0.32172
720.93	5.61961	720.697	-0.32586
721.93	5.61558	721.697	-0.33174
722.93	5.60469	722.697	-0.33684
723.93	5.59502	723.697	-0.33973
724.93	5.58814	724.697	-0.33672
725.93	5.57967	725.697	-0.33565
726.93	5.56371	726.697	-0.33588
727.93	5.54255	727.697	-0.33815
728.93	5.52146	7.29E+02	-0.34142
729.93	5.50915	729.697	-0.34197
730.93	5.50929	730.697	-0.33953
731.93	5.50739	731.697	-0.33501
732.93	5.49647	7.33E+02	-0.33702
733.93	5.48765	733.697	-0.33993
734.93	5.4846	734.697	-0.34275
735.93	5.48365	7.36E+02	-0.34518
736.93	5.48421	736.697	-0.3425
737.93	5.4769	737.697	-0.33939
738.93	5.46308	738.697	-0.33519
739.93	5.45066	739.697	-0.33406
740.93	5.44564	740.697	-0.33322
741.93	5.44607	741.697	-0.32959
742.93	5.44461	742.697	-0.32332
743.93	5.43932	743.697	-0.31922
744.93	5.43089	744.697	-0.3197
745.93	5.42425	7.46E+02	-0.3217
746.93	5.41857	746.697	-0.32663
747.93	5.4136	747.697	-0.32983
748.93	5.40999	7.49E+02	-0.32971
749.93	5.40662	749.697	-0.32874
7.51E+02	5.405	750.697	-0.32711
751.93	5.40612	751.697	-0.32331
752.93	5.40971	752.697	-0.31899

753.93	5.41429	753.697	-0.3138
754.93	5.41974	754.697	-0.30764
755.93	5.42392	755.697	-0.30286
756.93	5.42643	756.697	-0.29937
757.93	5.42467	757.697	-0.29858
758.93	5.42185	758.697	-0.30041
759.93	5.41948	7.60E+02	-0.30173
760.93	5.41794	760.697	-0.30154
761.93	5.41665	761.697	-0.3011
762.93	5.41497	762.697	-0.30224
763.93	5.41242	763.697	-0.30392
764.93	5.40945	764.697	-0.30522
765.93	5.40702	765.697	-0.30651
766.93	5.40492	766.697	-0.3076
767.93	5.40078	767.697	-0.30841
768.93	5.39657	768.697	-0.31055
769.93	5.39254	769.697	-0.31578
770.93	5.38891	770.697	-0.32005
771.93	5.38548	771.697	-0.32251
772.93	5.3823	772.697	-0.32514
773.93	5.38198	773.697	-0.32643
774.93	5.38359	774.697	-0.32216
775.93	5.38223	775.697	-0.32076
776.93	5.3797	776.697	-0.32309
777.93	5.3769	777.697	-0.32316
778.93	5.37178	778.697	-0.32456
779.93	5.36803	779.697	-0.32951
780.93	5.36915	780.697	-0.33357
781.93	5.37123	781.697	-0.33442
782.93	5.36992	782.697	-0.34116
783.93	5.36996	783.697	-0.34655
784.93	5.36727	784.697	-0.35072
785.93	5.37069	785.697	-0.35107
786.93	5.37852	786.697	-0.34908
787.93	5.38074	7.88E+02	-0.34463
788.93	5.37885	788.697	-0.34147
789.93	5.37522	789.697	-0.3433
790.93	5.3804	7.91E+02	-0.34571
791.93	5.38385	791.697	-0.34762
792.93	5.38312	792.697	-0.34576
793.93	5.3817	793.697	-0.34646
794.93	5.38552	794.697	-0.34856
795.93	5.38724	795.697	-0.34994

796.93	5.35638	7.97E+02	-0.35

DTG curve of water-immersed coking coal

temperature	***DTG***	***temperature***	***DTG***	***temperature***	***DTG***
℃	%/min	℃	%/min	℃	%/min
	Coal		Coal+MgCl2		Coal+TEMPO
30.506	-0.24242	29.991	0.007856214	29.267	0.04218523
31.506	-0.16283	30.991	-0.009766552	30.267	-0.05498799
32.506	-0.15636	31.991	0.00672019	31.267	-0.05115572
33.506	-0.1625	32.991	0.01530687	32.267	-0.03375448
34.506	-0.15211	33.991	0.01222574	33.267	-0.01881178
35.506	-0.13162	34.991	0.006075159	34.267	-0.01162745
36.506	-0.09934848	35.991	-0.003919709	35.267	-0.008277789
37.506	-0.08058961	36.991	-0.01279242	36.267	-0.01282438
38.506	-0.06930588	37.991	-0.02341949	37.267	-0.01214255
39.506	-0.06280201	38.991	-0.03327576	38.267	-0.01634539
40.506	-0.05923605	39.991	-0.03998489	39.267	-0.02019482
41.506	-0.05787344	40.991	-0.04958038	40.267	-0.0222548
42.506	-0.05409206	41.991	-0.05467122	41.267	-0.02453207
43.506	-0.04635323	42.991	-0.06221979	42.267	-0.02132082
44.506	-0.04221169	43.991	-0.07360613	43.267	-0.02385612
45.506	-0.04531919	44.991	-0.08388939	44.267	-0.02736206
46.506	-0.0427881	45.991	-0.09718157	45.267	-0.02735115
47.506	-0.03896389	46.991	-0.11067	46.267	-0.02914211
48.506	-0.03326021	47.991	-0.12249	47.267	-0.03036177
49.506	-0.03116919	48.991	-0.13661	48.267	-0.02979662
50.506	-0.03333345	49.991	-0.15515	49.267	-0.03299182
51.506	-0.03075031	50.991	-0.17111	50.267	-0.03784636
52.506	-0.02271844	51.991	-0.18436	51.267	-0.03670683
53.506	-0.01690286	52.991	-0.19802	52.267	-0.03264288
54.506	-0.00935782	53.991	-0.21044	53.267	-0.03179085
55.506	-0.00566971	54.991	-0.22112	54.267	-0.02596791
56.506	-0.00633572	55.991	-0.23709	55.267	-0.02142373
57.506	-0.00582265	56.991	-0.25534	56.267	-0.02070612
58.506	-0.0022705	57.991	-0.27243	57.267	-0.02123993
59.506	0.000306067	58.991	-0.28644	58.267	-0.01942046
60.506	0.001107125	59.991	-0.30023	59.267	-0.01661681
61.506	-0.00023084	60.991	-0.31436	60.267	-0.01660797
62.506	-0.00383058	61.991	-0.32704	61.267	-0.01951019
63.506	-0.00958727	62.991	-0.33954	62.267	-0.02180178
64.506	-0.01289743	63.991	-0.35281	63.267	-0.02640686
65.506	-0.01330774	64.991	-0.36055	64.267	-0.03181929

66.506	-0.01436302	65.991	-0.3622	65.267	-0.03415839
67.506	-0.01671924	66.991	-0.36029	66.267	-0.03375453
68.506	-0.01978719	67.991	-0.35454	67.267	-0.03287444
69.506	-0.02345914	68.991	-0.3478	68.267	-0.03167507
70.506	-0.02763146	69.991	-0.34103	69.267	-0.03094193
71.506	-0.0304621	70.991	-0.33324	70.267	-0.03050863
72.506	-0.03146088	71.991	-0.32663	71.267	-0.03122911
73.506	-0.03085513	72.991	-0.31775	72.267	-0.03192745
74.506	-0.02913158	73.991	-0.30905	73.267	-0.03189021
75.506	-0.02641764	74.991	-0.30175	74.267	-0.03120077
76.506	-0.02367595	75.991	-0.29735	75.267	-0.02964144
77.506	-0.02174798	76.991	-0.29684	76.267	-0.02797384
78.506	-0.01980264	77.991	-0.30358	77.267	-0.02747915
79.506	-0.01836942	78.991	-0.31303	78.267	-0.02894065
80.506	-0.01458818	79.991	-0.32461	79.267	-0.03043739
81.506	-0.00936323	80.991	-0.33885	80.267	-0.0313119
82.506	-0.00551867	81.991	-0.35133	81.267	-0.0289782
83.506	-0.00427861	82.991	-0.36591	82.267	-0.02514517
84.506	-0.00401476	83.991	-0.37981	83.267	-0.02193564
85.506	-0.00408449	84.991	-0.39267	84.267	-0.02106428
86.506	-0.00242954	85.991	-0.40313	85.267	-0.02197617
87.506	-0.00145779	86.991	-0.40789	86.267	-0.02456734
88.506	-0.00091015	87.991	-0.40684	87.267	-0.02574715
89.506	0.000713578	88.991	-0.4055	88.267	-0.02633837
90.506	0.000494058	89.991	-0.40236	89.267	-0.02783852
91.506	-0.00017728	90.991	-0.39793	90.267	-0.02844256
92.506	-0.00133197	91.991	-0.39564	91.267	-0.03029618
93.506	-0.00263888	92.991	-0.39523	92.267	-0.0319361
94.506	-0.00362356	93.991	-0.40091	93.267	-0.03339094
95.506	-0.00576474	94.991	-0.41221	94.267	-0.03482372
96.506	-0.00726986	95.991	-0.42822	95.267	-0.03462133
97.506	-0.00660565	96.991	-0.44983	96.267	-0.03409555
98.506	-0.00686706	97.991	-0.47358	97.267	-0.03346827
99.506	-0.00680715	98.991	-0.49645	98.267	-0.0308649
100.506	-0.0063959	99.991	-0.51785	99.267	-0.0285664
101.506	-0.00647012	100.991	-0.53384	100.267	-0.02673554
102.506	-0.00452095	101.991	-0.54509	101.267	-0.02576425
103.506	-0.00458722	102.991	-0.54639	102.267	-0.02618359
104.506	-0.00474146	103.991	-0.53706	103.267	-0.02604305
105.506	-0.0058182	104.991	-0.5174	104.267	-0.02716719
106.506	-0.00833613	105.991	-0.48802	105.267	-0.02728897
107.506	-0.00948552	106.991	-0.45027	106.267	-0.0277168
108.506	-0.00948526	107.991	-0.40338	107.267	-0.02892135

109.506	-0.0120562	108.991	-0.35266	108.267	-0.02953932
110.506	-0.01333194	109.991	-0.30578	109.267	-0.03140713
111.506	-0.01549897	110.991	-0.26127	110.267	-0.03503153
112.506	-0.01529831	111.991	-0.22144	111.267	-0.03645301
113.506	-0.01487175	112.991	-0.18645	112.267	-0.03675529
114.506	-0.01386642	113.991	-0.1573	113.267	-0.03458372
115.506	-0.01071875	114.991	-0.13671	114.267	-0.03484731
116.506	-0.00833823	115.991	-0.12288	115.267	-0.03527962
117.506	-0.00845023	116.991	-0.1181	116.267	-0.03500401
118.506	-0.00799548	117.991	-0.12003	117.267	-0.03564856
119.506	-0.00636466	118.991	-0.12509	118.267	-0.03682525
120.506	-0.0048886	119.991	-0.13177	119.267	-0.03633484
121.506	-0.00494902	120.991	-0.13948	120.267	-0.03596461
122.506	-0.00494466	121.991	-0.1503	121.267	-0.03762007
123.506	-0.00501442	122.991	-0.16158	122.267	-0.04113451
124.506	-0.0070059	123.991	-0.16898	123.267	-0.04221578
125.506	-0.00907165	124.991	-0.17451	124.267	-0.04309602
126.506	-0.00927951	125.991	-0.17792	125.267	-0.04451815
127.506	-0.01066596	126.991	-0.17761	126.267	-0.04502816
128.506	-0.01380864	127.991	-0.17501	127.267	-0.04293212
129.506	-0.01583594	128.991	-0.17119	128.267	-0.04193665
130.506	-0.01577125	129.991	-0.1646	129.267	-0.04092294
131.506	-0.01427743	130.991	-0.15629	130.267	-0.03739251
132.506	-0.01427603	131.991	-0.15006	131.267	-0.03181033
133.506	-0.01538804	132.991	-0.15042	132.267	-0.02814922
134.506	-0.017187	133.991	-0.15484	133.267	-0.02753888
135.506	-0.01731671	134.991	-0.1624	134.267	-0.02839304
136.506	-0.01279992	135.991	-0.17267	135.267	-0.03027598
137.506	-0.00778502	136.991	-0.1849	136.267	-0.03053876
138.506	-0.00516283	137.991	-0.20033	137.267	-0.03040207
139.506	-0.00614174	138.991	-0.2205	138.267	-0.03105835
140.506	-0.00817974	139.991	-0.24102	139.267	-0.03553167
141.506	-0.00697884	140.991	-0.25793	140.267	-0.04198556
142.506	-0.00285301	141.991	-0.26722	141.267	-0.04406762
143.506	0.001213811	142.991	-0.27079	142.267	-0.0403006
144.506	0.001438752	143.991	-0.27031	143.267	-0.03415643
145.506	-0.00036032	144.991	-0.26623	144.267	-0.0289926
146.506	-0.00216927	145.991	-0.25577	145.267	-0.0260335
147.506	-0.00117707	146.991	-0.24039	146.267	-0.02334055
148.506	0.002255283	147.991	-0.21855	147.267	-0.01908365
149.506	0.005059729	148.991	-0.19326	148.267	-0.01221535
150.506	0.005819238	149.991	-0.16709	149.267	-0.005707304
151.506	0.004528334	150.991	-0.144	150.267	-0.002323037

152.506	0.002771465	151.991	-0.12305	151.267	-0.003493529
153.506	-0.00022963	152.991	-0.10473	152.267	-0.006242297
154.506	-0.00374876	153.991	-0.09075115	153.267	-0.009848873
155.506	-0.00765383	154.991	-0.07925167	154.267	-0.01476374
156.506	-0.01195067	155.991	-0.07114183	155.267	-0.02014089
157.506	-0.01524075	156.991	-0.06713308	156.267	-0.02692342
158.506	-0.01737482	157.991	-0.0652169	157.267	-0.03366271
159.506	-0.01776307	158.991	-0.06400238	158.267	-0.03970405
160.506	-0.01607304	159.991	-0.06189366	159.267	-0.04409775
161.506	-0.01149456	160.991	-0.05831925	160.267	-0.04630558
162.506	-0.00502695	161.991	-0.05198173	161.267	-0.04648759
163.506	0.000669161	162.991	-0.04569712	162.267	-0.04327709
164.506	0.005048406	163.991	-0.04008425	163.267	-0.03938719
165.506	0.008064937	164.991	-0.03346172	164.267	-0.03526176
166.506	0.009009774	165.991	-0.02771051	165.267	-0.03115666
167.506	0.009415758	166.991	-0.0238903	166.267	-0.02771012
168.506	0.009126226	167.991	-0.02202359	167.267	-0.02486081
169.506	0.007688985	168.991	-0.02164011	168.267	-0.02314515
170.506	0.005226528	169.991	-0.02330272	169.267	-0.02213133
171.506	0.003894451	170.991	-0.02609587	170.267	-0.02246859
172.506	0.003998664	171.991	-0.02778733	171.267	-0.02249509
173.506	0.004985555	172.991	-0.02986002	172.267	-0.02105449
174.506	0.007020751	173.991	-0.03162597	173.267	-0.01883822
175.506	0.008784616	174.991	-0.0315627	174.267	-0.01629046
176.506	0.009759859	175.991	-0.02959943	175.267	-0.01368207
177.506	0.01019004	176.991	-0.02719542	176.267	-0.01061118
178.506	0.01030016	177.991	-0.02534942	177.267	-0.007481672
179.506	0.009715483	178.991	-0.02395372	178.267	-0.005046011
180.506	0.007925764	179.991	-0.02414816	179.267	-0.003576091
181.506	0.004894006	180.991	-0.02588912	180.267	-0.003194712
182.506	0.001121089	181.991	-0.02907607	181.267	-0.005272799
183.506	-0.00287121	182.991	-0.03440127	182.267	-0.008303602
184.506	-0.00670282	183.991	-0.04235924	183.267	-0.01084852
185.506	-0.00938171	184.991	-0.05185792	184.267	-0.01405172
186.506	-0.01082282	185.991	-0.06075518	185.267	-0.01708756
187.506	-0.01148899	186.991	-0.06842993	186.267	-0.01856415
188.506	-0.01136152	187.991	-0.07481297	187.267	-0.01827231
189.506	-0.00930029	188.991	-0.08088705	188.267	-0.01835785
190.506	-0.0071308	189.991	-0.08651132	189.267	-0.01708439
191.506	-0.0049969	190.991	-0.09236401	190.267	-0.01557975
192.506	-0.002708	191.991	-0.09763303	191.267	-0.01628494
193.506	-0.00110426	192.991	-0.10305	192.267	-0.0170681
194.506	-0.00055576	193.991	-0.10947	193.267	-0.01784141

195.506	-0.00154653	194.991	-0.11816	194.267	-0.01910323
196.506	-0.00389714	195.991	-0.1282	195.267	-0.02189649
197.506	-0.00829574	196.991	-0.13847	196.267	-0.02510442
198.506	-0.01006802	197.991	-0.14845	197.267	-0.02851605
199.506	-0.0098866	198.991	-0.15269	198.267	-0.02969352
200.506	-0.00870537	199.991	-0.15381	199.267	-0.02654285
201.506	-0.00624262	200.991	-0.15116	200.267	-0.02155899
202.506	-0.00365956	201.991	-0.14595	201.267	-0.0148662
203.506	0.001282775	202.991	-0.1375	202.267	-0.009817774
204.506	0.007821882	203.991	-0.12421	203.267	-0.005020508
205.506	0.01675362	204.991	-0.1095	204.267	0.000563701
206.506	0.02401068	205.991	-0.090231	205.267	0.006993379
207.506	0.02855677	206.991	-0.07591975	206.267	0.01214891
208.506	0.03146419	207.991	-0.06375992	207.267	0.01208988
209.506	0.03343808	208.991	-0.05346729	208.267	0.0110867
210.506	0.03521654	209.991	-0.04397901	209.267	0.008028613
211.506	0.03477266	210.991	-0.03614916	210.267	0.005028276
212.506	0.03493046	211.991	-0.03136353	211.267	0.002288714
213.506	0.03553107	212.991	-0.02631939	212.267	-0.000559111
214.506	0.03511667	213.991	-0.02392576	213.267	-0.002356379
215.506	0.03477687	214.991	-0.02045478	214.267	-0.003380855
216.506	0.03639745	215.991	-0.01661732	215.267	-0.000252773
217.506	0.0387376	216.991	-0.01314623	216.267	0.003256211
218.506	0.03965074	217.991	-0.01036306	217.267	0.008593225
219.506	0.04032779	218.991	-0.009203059	218.267	0.01434637
220.506	0.03900514	219.991	-0.008584855	219.267	0.01916737
221.506	0.03634612	220.991	-0.01012985	220.267	0.02421697
222.506	0.03494396	221.991	-0.01320513	221.267	0.02784204
223.506	0.03498723	222.991	-0.01759736	222.267	0.03026044
224.506	0.03357389	223.991	-0.02258574	223.267	0.02901698
225.506	0.03042012	224.991	-0.02885007	224.267	0.02641855
226.506	0.02990984	225.991	-0.034997	225.267	0.02160698
227.506	0.03310005	226.991	-0.03778758	226.267	0.01813908
228.506	0.03743275	227.991	-0.03875303	227.267	0.01875911
229.506	0.04164816	228.991	-0.03783406	228.267	0.0197568
230.506	0.04383668	229.991	-0.03638969	229.267	0.02085478
231.506	0.04525056	230.991	-0.03428119	230.267	0.02171255
232.506	0.04658402	231.991	-0.0319516	231.267	0.02406796
233.506	0.04870465	232.991	-0.02898058	232.267	0.02750422
234.506	0.05056416	233.991	-0.02672039	233.267	0.03130096
235.506	0.05080748	234.991	-0.02630933	234.267	0.03400423
236.506	0.04992943	235.991	-0.02692423	235.267	0.03384128
237.506	0.04989248	236.991	-0.02803153	236.267	0.03275857

238.506	0.05209277	237.991	-0.02764096	237.267	0.03158891
239.506	0.05484793	238.991	-0.02597793	238.267	0.03229545
240.506	0.0585763	239.991	-0.02274104	239.267	0.03446848
241.506	0.06451711	240.991	-0.016866	240.267	0.03713885
242.506	0.07069949	241.991	-0.00859705	241.267	0.04098193
243.506	0.07519558	242.991	0.000915738	242.267	0.04528761
244.506	0.080298	243.991	0.01153571	243.267	0.05070044
245.506	0.08492934	244.991	0.02244885	244.267	0.05792381
246.506	0.08773801	245.991	0.03316138	245.267	0.06574945
247.506	0.09030118	246.991	0.04333406	246.267	0.07077489
248.506	0.09345955	247.991	0.05185155	247.267	0.07389756
249.506	0.09361349	248.991	0.05789451	248.267	0.07638371
250.506	0.09238053	249.991	0.06331752	249.267	0.07959436
251.506	0.09230324	250.991	0.06894932	250.267	0.08296495
252.506	0.09137279	251.991	0.07378237	251.267	0.08524685
253.506	0.09022974	252.991	0.07827817	252.267	0.08498001
254.506	0.08947824	253.991	0.0818702	253.267	0.08516939
255.506	0.08974404	254.991	0.08494198	254.267	0.08830906
256.506	0.0888391	255.991	0.0886086	255.267	0.09289178
257.506	0.08899752	256.991	0.09337159	256.267	0.09708202
258.506	0.0900222	257.991	0.09725037	257.267	0.10006
259.506	0.09124597	258.991	0.09970672	258.267	0.10218
260.506	0.09432233	259.991	0.10159	259.267	0.10425
261.506	0.09768601	260.991	0.10311	260.267	0.10725
262.506	0.10241	261.991	0.10462	261.267	0.10867
263.506	0.10651	262.991	0.1065	262.267	0.10825
264.506	0.10928	263.991	0.10905	263.267	0.10691
265.506	0.11252	264.991	0.11288	264.267	0.10598
266.506	0.11566	265.991	0.11653	265.267	0.10467
267.506	0.11812	266.991	0.12028	266.267	0.10399
268.506	0.11874	267.991	0.12401	267.267	0.10411
269.506	0.1186	268.991	0.12687	268.267	0.10392
270.506	0.11809	269.991	0.12845	269.267	0.10497
271.506	0.1174	270.991	0.12899	270.267	0.10608
272.506	0.11801	271.991	0.12661	271.267	0.10731
273.506	0.11873	272.991	0.12109	272.267	0.1089
274.506	0.12038	273.991	0.11509	273.267	0.11163
275.506	0.123	274.991	0.10903	274.267	0.11372
276.506	0.12555	275.991	0.10326	275.267	0.11498
277.506	0.1308	276.991	0.10018	276.267	0.11719
278.506	0.1354	277.991	0.10044	277.267	0.12044
279.506	0.14049	278.991	0.10253	278.267	0.12504
280.506	0.147	279.991	0.10726	279.267	0.12973

281.506	0.15275	280.991	0.11405	280.267	0.13505
282.506	0.15623	281.991	0.12065	281.267	0.14
283.506	0.15941	282.991	0.12648	282.267	0.14601
284.506	0.16365	283.991	0.13272	283.267	0.15236
285.506	0.16541	284.991	0.13734	284.267	0.15856
286.506	0.16765	285.991	0.13938	285.267	0.16375
287.506	0.1691	286.991	0.13978	286.267	0.16698
288.506	0.16884	287.991	0.1396	287.267	0.17044
289.506	0.16909	288.991	0.13739	288.267	0.17315
290.506	0.1714	289.991	0.13509	289.267	0.17455
291.506	0.1717	290.991	0.13533	290.267	0.17506
292.506	0.17247	291.991	0.13535	291.267	0.1758
293.506	0.17395	292.991	0.13457	292.267	0.1763
294.506	0.17433	293.991	0.13301	293.267	0.17546
295.506	0.17404	294.991	0.1317	294.267	0.17532
296.506	0.17422	295.991	0.12979	295.267	0.17551
297.506	0.17467	296.991	0.13053	296.267	0.17544
298.506	0.17323	297.991	0.13248	297.267	0.17654
299.506	0.17465	298.991	0.13314	298.267	0.17779
300.506	0.1759	299.991	0.13337	299.267	0.17942
301.506	0.17646	300.991	0.13424	300.267	0.18109
302.506	0.17668	301.991	0.13523	301.267	0.18293
303.506	0.17767	302.991	0.13575	302.267	0.18413
304.506	0.1776	303.991	0.13512	303.267	0.18389
305.506	0.17637	304.991	0.13125	304.267	0.18196
306.506	0.17695	305.991	0.12696	305.267	0.17946
307.506	0.17548	306.991	0.12168	306.267	0.1769
308.506	0.1719	307.991	0.1151	307.267	0.17301
309.506	0.16842	308.991	0.10764	308.267	0.16837
310.506	0.16527	309.991	0.10206	309.267	0.16381
311.506	0.16081	310.991	0.09791305	310.267	0.16089
312.506	0.15818	311.991	0.0955161	311.267	0.15765
313.506	0.15618	312.991	0.09545098	312.267	0.15586
314.506	0.15269	313.991	0.09456124	313.267	0.15465
315.506	0.14897	314.991	0.09395308	314.267	0.15421
316.506	0.14608	315.991	0.09546334	315.267	0.15409
317.506	0.14263	316.991	0.09748461	316.267	0.15394
318.506	0.14156	317.991	0.09749253	317.267	0.15277
319.506	0.14188	318.991	0.09520805	318.267	0.15029
320.506	0.14012	319.991	0.09166277	319.267	0.1499
321.506	0.13527	320.991	0.08541574	320.267	0.14892
322.506	0.13011	321.991	0.07835581	321.267	0.14508
323.506	0.12543	322.991	0.07047356	322.267	0.13764

324.506	0.12059	323.991	0.06114772	323.267	0.12861
325.506	0.11513	324.991	0.05194344	324.267	0.11918
326.506	0.1056	325.991	0.04307158	325.267	0.11081
327.506	0.09517927	326.991	0.03505688	326.267	0.10148
328.506	0.08485214	327.991	0.02628637	327.267	0.08858255
329.506	0.07725246	328.991	0.01860528	328.267	0.07559589
330.506	0.06918474	329.991	0.01085652	329.267	0.06535047
331.506	0.05993312	330.991	0.003246097	330.267	0.05861104
332.506	0.05034026	331.991	-0.004927208	331.267	0.05332146
333.506	0.04196556	332.991	-0.01481929	332.267	0.04768997
334.506	0.03547841	333.991	-0.02337988	333.267	0.04198465
335.506	0.0281946	334.991	-0.03273542	334.267	0.03677258
336.506	0.01921198	335.991	-0.04075431	335.267	0.03185529
337.506	0.009128007	336.991	-0.04866323	336.267	0.02641165
338.506	-0.00123433	337.991	-0.05465053	337.267	0.01858217
339.506	-0.01116813	338.991	-0.06080533	338.267	0.007710554
340.506	-0.02142204	339.991	-0.06676561	339.267	-0.00511039
341.506	-0.0330389	340.991	-0.07271576	340.267	-0.01764047
342.506	-0.04654762	341.991	-0.08032122	341.267	-0.03129059
343.506	-0.05914828	342.991	-0.08789465	342.267	-0.0455198
344.506	-0.06882029	343.991	-0.09527606	343.267	-0.05825452
345.506	-0.07785778	344.991	-0.10173	344.267	-0.07091947
346.506	-0.08707858	345.991	-0.10935	345.267	-0.08204097
347.506	-0.0967865	346.991	-0.11613	346.267	-0.09180583
348.506	-0.10648	347.991	-0.12379	347.267	-0.10265
349.506	-0.11594	348.991	-0.13123	348.267	-0.11439
350.506	-0.12394	349.991	-0.13799	349.267	-0.12644
351.506	-0.1322	350.991	-0.14344	350.267	-0.13877
352.506	-0.14296	351.991	-0.15088	351.267	-0.15157
353.506	-0.15268	352.991	-0.16024	352.267	-0.16431
354.506	-0.16178	353.991	-0.17082	353.267	-0.17677
355.506	-0.17041	354.991	-0.18229	354.267	-0.1889
356.506	-0.17912	355.991	-0.1926	355.267	-0.19913
357.506	-0.1881	356.991	-0.20327	356.267	-0.20786
358.506	-0.19891	357.991	-0.21474	357.267	-0.21694
359.506	-0.21085	358.991	-0.22849	358.267	-0.22447
360.506	-0.22216	359.991	-0.24078	359.267	-0.23361
361.506	-0.23571	360.991	-0.25186	360.267	-0.24239
362.506	-0.2505	361.991	-0.26277	361.267	-0.25275
363.506	-0.26516	362.991	-0.27351	362.267	-0.26367
364.506	-0.27925	363.991	-0.28401	363.267	-0.275
365.506	-0.29205	364.991	-0.29332	364.267	-0.28657
366.506	-0.30351	365.991	-0.30096	365.267	-0.29643

367.506	-0.31375	366.991	-0.3079	366.267	-0.30799
368.506	-0.32472	367.991	-0.31483	367.267	-0.31817
369.506	-0.33538	368.991	-0.32313	368.267	-0.32989
370.506	-0.34445	369.991	-0.33165	369.267	-0.34127
371.506	-0.35292	370.991	-0.33911	370.267	-0.35134
372.506	-0.36206	371.991	-0.3463	371.267	-0.36122
373.506	-0.37188	372.991	-0.3547	372.267	-0.37165
374.506	-0.38201	373.991	-0.36435	373.267	-0.38325
375.506	-0.39305	374.991	-0.37298	374.267	-0.39285
376.506	-0.40336	375.991	-0.38389	375.267	-0.4014
377.506	-0.41238	376.991	-0.3945	376.267	-0.4108
378.506	-0.42023	377.991	-0.40296	377.267	-0.42108
379.506	-0.42761	378.991	-0.41016	378.267	-0.43068
380.506	-0.4332	379.991	-0.41768	379.267	-0.43878
381.506	-0.43787	380.991	-0.4249	380.267	-0.44476
382.506	-0.44171	381.991	-0.43118	381.267	-0.45021
383.506	-0.4448	382.991	-0.43792	382.267	-0.45613
384.506	-0.44511	383.991	-0.44079	383.267	-0.46327
385.506	-0.44375	384.991	-0.44099	384.267	-0.46667
386.506	-0.44498	385.991	-0.44264	385.267	-0.46671
387.506	-0.44624	386.991	-0.44703	386.267	-0.46867
388.506	-0.44888	387.991	-0.45157	387.267	-0.47212
389.506	-0.45225	388.991	-0.45445	388.267	-0.47655
390.506	-0.45528	389.991	-0.45756	389.267	-0.48121
391.506	-0.4577	390.991	-0.46175	390.267	-0.4853
392.506	-0.4606	391.991	-0.46652	391.267	-0.48748
393.506	-0.46474	392.991	-0.47142	392.267	-0.48924
394.506	-0.46711	393.991	-0.47629	393.267	-0.49116
395.506	-0.47169	394.991	-0.48064	394.267	-0.4928
396.506	-0.47671	395.991	-0.48534	395.267	-0.49339
397.506	-0.48246	396.991	-0.49376	396.267	-0.49401
398.506	-0.48916	397.991	-0.50349	397.267	-0.49424
399.506	-0.49535	398.991	-0.51368	398.267	-0.49648
400.506	-0.50255	399.991	-0.52628	399.267	-0.50095
401.506	-0.51091	400.991	-0.54276	400.267	-0.50925
402.506	-0.52056	401.991	-0.56131	401.267	-0.51904
403.506	-0.52842	402.991	-0.58172	402.267	-0.5271
404.506	-0.53437	403.991	-0.60404	403.267	-0.5376
405.506	-0.53948	404.991	-0.62496	404.267	-0.54947
406.506	-0.54527	405.991	-0.645	405.267	-0.56248
407.506	-0.553	406.991	-0.66322	406.267	-0.57431
408.506	-0.56235	407.991	-0.68127	407.267	-0.58471
409.506	-0.5723	408.991	-0.70003	408.267	-0.59098

410.506	-0.58103	409.991	-0.71811	409.267	-0.59775
411.506	-0.59176	410.991	-0.73823	410.267	-0.6063
412.506	-0.60483	411.991	-0.76039	411.267	-0.61387
413.506	-0.61856	412.991	-0.785	412.267	-0.6212
414.506	-0.63089	413.991	-0.81291	413.267	-0.62804
415.506	-0.64244	414.991	-0.84434	414.267	-0.63434
416.506	-0.65398	415.991	-0.87736	415.267	-0.64084
417.506	-0.6663	416.991	-0.91239	416.267	-0.65197
418.506	-0.68073	417.991	-0.94922	417.267	-0.66497
419.506	-0.69365	418.991	-0.98465	418.267	-0.67823
420.506	-0.7061	419.991	-1.01609	419.267	-0.69237
421.506	-0.71877	420.991	-1.04617	420.267	-0.70484
422.506	-0.73331	421.991	-1.07265	421.267	-0.7175
423.506	-0.74778	422.991	-1.09612	422.267	-0.73227
424.506	-0.76073	423.991	-1.11551	423.267	-0.74812
425.506	-0.77206	424.991	-1.12703	424.267	-0.76309
426.506	-0.78468	425.991	-1.13315	425.267	-0.77672
427.506	-0.79927	426.991	-1.13485	426.267	-0.79196
428.506	-0.81357	427.991	-1.13605	427.267	-0.8059
429.506	-0.82838	428.991	-1.13398	428.267	-0.8201
430.506	-0.84236	429.991	-1.13084	429.267	-0.83489
431.506	-0.85876	430.991	-1.12747	430.267	-0.84773
432.506	-0.87877	431.991	-1.12532	431.267	-0.86115
433.506	-0.90151	432.991	-1.12943	432.267	-0.87572
434.506	-0.92253	433.991	-1.13786	433.267	-0.89294
435.506	-0.94162	434.991	-1.14918	434.267	-0.90853
436.506	-0.96259	435.991	-1.16284	435.267	-0.92682
437.506	-0.9849	436.991	-1.18213	436.267	-0.94878
438.506	-1.00868	437.991	-1.20325	437.267	-0.97485
439.506	-1.03288	438.991	-1.22814	438.267	-1.00501
440.506	-1.05561	439.991	-1.25414	439.267	-1.03671
441.506	-1.07721	440.991	-1.27969	440.267	-1.06736
442.506	-1.10134	441.991	-1.30626	441.267	-1.09438
443.506	-1.13095	442.991	-1.33409	442.267	-1.12361
444.506	-1.16095	443.991	-1.36156	443.267	-1.1517
445.506	-1.19152	444.991	-1.38707	444.267	-1.17964
446.506	-1.22363	445.991	-1.41677	445.267	-1.20476
447.506	-1.25668	446.991	-1.45022	446.267	-1.23147
448.506	-1.2914	447.991	-1.48791	447.267	-1.26056
449.506	-1.33014	448.991	-1.52369	448.267	-1.29235
450.506	-1.36849	449.991	-1.55788	449.267	-1.3278
451.506	-1.40471	450.991	-1.59061	450.267	-1.36449
452.506	-1.44191	451.991	-1.63001	451.267	-1.40555

453.506	-1.47942	452.991	-1.67119	452.267	-1.44648
454.506	-1.51944	453.991	-1.7072	453.267	-1.48719
455.506	-1.55934	454.991	-1.74035	454.267	-1.52663
456.506	-1.59942	455.991	-1.7711	455.267	-1.56404
457.506	-1.63815	456.991	-1.80564	456.267	-1.60221
458.506	-1.68304	457.991	-1.84516	457.267	-1.64206
459.506	-1.72475	458.991	-1.88906	458.267	-1.68291
460.506	-1.76643	459.991	-1.92866	459.267	-1.71945
461.506	-1.81467	460.991	-1.96802	460.267	-1.75698
462.506	-1.86576	461.991	-2.01459	461.267	-1.80303
463.506	-1.92136	462.991	-2.06489	462.267	-1.85154
464.506	-1.98009	463.991	-2.11592	463.267	-1.90288
465.506	-2.04124	464.991	-2.16439	464.267	-1.95429
466.506	-2.10158	465.991	-2.21393	465.267	-2.00701
467.506	-2.16865	466.991	-2.26849	466.267	-2.06163
468.506	-2.24275	467.991	-2.32377	467.267	-2.12793
469.506	-2.31573	468.991	-2.37968	468.267	-2.19606
470.506	-2.38623	469.991	-2.43474	469.267	-2.26333
471.506	-2.45779	470.991	-2.48596	470.267	-2.33181
472.506	-2.53107	471.991	-2.54437	471.267	-2.39992
473.506	-2.60281	472.991	-2.60691	472.267	-2.47388
474.506	-2.67772	473.991	-2.67051	473.267	-2.55292
475.506	-2.75319	474.991	-2.72184	474.267	-2.62925
476.506	-2.82879	475.991	-2.77876	475.267	-2.69824
477.506	-2.89452	476.991	-2.84036	476.267	-2.76628
478.506	-2.96697	477.991	-2.89681	477.267	-2.83654
479.506	-3.04021	478.991	-2.95475	478.267	-2.90034
480.506	-3.11473	479.991	-3.0109	479.267	-2.97098
481.506	-3.19126	480.991	-3.06483	480.267	-3.04293
482.506	-3.27167	481.991	-3.1151	481.267	-3.11358
483.506	-3.34388	482.991	-3.17307	482.267	-3.18511
484.506	-3.41625	483.991	-3.22496	483.267	-3.25431
485.506	-3.49868	484.991	-3.27417	484.267	-3.33031
486.506	-3.57965	485.991	-3.32549	485.267	-3.40209
487.506	-3.65651	486.991	-3.37773	486.267	-3.48019
488.506	-3.72864	487.991	-3.42135	487.267	-3.55532
489.506	-3.79806	488.991	-3.46306	488.267	-3.62231
490.506	-3.86292	489.991	-3.50363	489.267	-3.6822
491.506	-3.93182	490.991	-3.54269	490.267	-3.74193
492.506	-3.99905	491.991	-3.58235	491.267	-3.80762
493.506	-4.06027	492.991	-3.61797	492.267	-3.86828
494.506	-4.11449	493.991	-3.64944	493.267	-3.92451
495.506	-4.16473	494.991	-3.67792	494.267	-3.97631

496.506	-4.21033	495.991	-3.70647	495.267	-4.02405
497.506	-4.25711	496.991	-3.73343	496.267	-4.07006
498.506	-4.29693	497.991	-3.75723	497.267	-4.1181
499.506	-4.33389	498.991	-3.77644	498.267	-4.16319
500.506	-4.36806	499.991	-3.79028	499.267	-4.19961
501.506	-4.39647	500.991	-3.80492	500.267	-4.23486
502.506	-4.42286	501.991	-3.81751	501.267	-4.26758
503.506	-4.44658	502.991	-3.82582	502.267	-4.29919
504.506	-4.46936	503.991	-3.83179	503.267	-4.32742
505.506	-4.48311	504.991	-3.83276	504.267	-4.35261
506.506	-4.49313	505.991	-3.82779	505.267	-4.37282
507.506	-4.49367	506.991	-3.81754	506.267	-4.38582
508.506	-4.48983	507.991	-3.80502	507.267	-4.39316
509.506	-4.48092	508.991	-3.78723	508.267	-4.39433
510.506	-4.46659	509.991	-3.76464	509.267	-4.3919
511.506	-4.44403	510.991	-3.73858	510.267	-4.3805
512.506	-4.41149	511.991	-3.70546	511.267	-4.36556
513.506	-4.37687	512.991	-3.66936	512.267	-4.34211
514.506	-4.33966	513.991	-3.63354	513.267	-4.31362
515.506	-4.29818	514.991	-3.5967	514.267	-4.2844
516.506	-4.2463	515.991	-3.54835	515.267	-4.25084
517.506	-4.19031	516.991	-3.49427	516.267	-4.20985
518.506	-4.13132	517.991	-3.43884	517.267	-4.16351
519.506	-4.07122	518.991	-3.37675	518.267	-4.11351
520.506	-4.01279	519.991	-3.31967	519.267	-4.05764
521.506	-3.94886	520.991	-3.25759	520.267	-4.00401
522.506	-3.87887	521.991	-3.18693	521.267	-3.94554
523.506	-3.81069	522.991	-3.1125	522.267	-3.88153
524.506	-3.74413	523.991	-3.04786	523.267	-3.81158
525.506	-3.6733	524.991	-2.98391	524.267	-3.74614
526.506	-3.59853	525.991	-2.91441	525.267	-3.67737
527.506	-3.52416	526.991	-2.84947	526.267	-3.60706
528.506	-3.44792	527.991	-2.77862	527.267	-3.52964
529.506	-3.36735	528.991	-2.71557	528.267	-3.45206
530.506	-3.29544	529.991	-2.66374	529.267	-3.37268
531.506	-3.21506	530.991	-2.61095	530.267	-3.29741
532.506	-3.13767	531.991	-2.55486	531.267	-3.22297
533.506	-3.06704	532.991	-2.50154	532.267	-3.14532
534.506	-2.99523	533.991	-2.45336	533.267	-3.06776
535.506	-2.93099	534.991	-2.40417	534.267	-2.99582
536.506	-2.86172	535.991	-2.35841	535.267	-2.93064
537.506	-2.80169	536.991	-2.30727	536.267	-2.86776
538.506	-2.73816	537.991	-2.25544	537.267	-2.80475

539.506	-2.68894	538.991	-2.20503	538.267	-2.74534
540.506	-2.63932	539.991	-2.1567	539.267	-2.68741
541.506	-2.58799	540.991	-2.10699	540.267	-2.63231
542.506	-2.54635	541.991	-2.05862	541.267	-2.58122
543.506	-2.49821	542.991	-2.01391	542.267	-2.53265
544.506	-2.45305	543.991	-1.96783	543.267	-2.48289
545.506	-2.40879	544.991	-1.92888	544.267	-2.43355
546.506	-2.36546	545.991	-1.88979	545.267	-2.38951
547.506	-2.3159	546.991	-1.8516	546.267	-2.34384
548.506	-2.26757	547.991	-1.81626	547.267	-2.30102
549.506	-2.22041	548.991	-1.78368	548.267	-2.2584
550.506	-2.17172	549.991	-1.75251	549.267	-2.21585
551.506	-2.12948	550.991	-1.72128	550.267	-2.17398
552.506	-2.09034	551.991	-1.69033	551.267	-2.13209
553.506	-2.05176	552.991	-1.65751	552.267	-2.08991
554.506	-2.01072	553.991	-1.62424	553.267	-2.04645
555.506	-1.97461	554.991	-1.59074	554.267	-2.00649
556.506	-1.94087	555.991	-1.55868	555.267	-1.96576
557.506	-1.90683	556.991	-1.5274	556.267	-1.92727
558.506	-1.86968	557.991	-1.49565	557.267	-1.88999
559.506	-1.82906	558.991	-1.45893	558.267	-1.84968
560.506	-1.79053	559.991	-1.42868	559.267	-1.80969
561.506	-1.74817	560.991	-1.39942	560.267	-1.77614
562.506	-1.70889	561.991	-1.37117	561.267	-1.74166
563.506	-1.66577	562.991	-1.34268	562.267	-1.70375
564.506	-1.62012	563.991	-1.30932	563.267	-1.66414
565.506	-1.57522	564.991	-1.27443	564.267	-1.62234
566.506	-1.53147	565.991	-1.23847	565.267	-1.58051
567.506	-1.48765	566.991	-1.2062	566.267	-1.53829
568.506	-1.43939	567.991	-1.16949	567.267	-1.49417
569.506	-1.39199	568.991	-1.12988	568.267	-1.44528
570.506	-1.34268	569.991	-1.09029	569.267	-1.39687
571.506	-1.29467	570.991	-1.04984	570.267	-1.35257
572.506	-1.24854	571.991	-1.01254	571.267	-1.30597
573.506	-1.20176	572.991	-0.97486	572.267	-1.25745
574.506	-1.14959	573.991	-0.93859	573.267	-1.20884
575.506	-1.09823	574.991	-0.89753	574.267	-1.16084
576.506	-1.04845	575.991	-0.856	575.267	-1.11177
577.506	-0.9968	576.991	-0.8154	576.267	-1.06428
578.506	-0.9479	577.991	-0.77224	577.267	-1.01359
579.506	-0.90165	578.991	-0.73269	578.267	-0.95931
580.506	-0.8508	579.991	-0.69414	579.267	-0.91155
581.506	-7.98E-01	580.991	-0.65227	580.267	-0.86574

582.506	-0.75281	581.991	-0.60904	581.267	-0.81778
583.506	-0.71086	582.991	-0.57203	582.267	-0.77086
584.506	-0.66748	583.991	-0.53453	583.267	-0.7248
585.506	-0.63088	584.991	-0.50149	584.267	-0.67743
586.506	-0.59263	585.991	-0.47239	585.267	-0.63797
587.506	-5.50E-01	586.991	-0.43872	586.267	-0.59892
588.506	-0.51413	587.991	-0.40507	587.267	-0.55273
589.506	-0.48188	588.991	-0.37695	588.267	-0.50914
590.506	-0.44766	589.991	-0.35008	589.267	-0.46923
591.506	-0.41658	590.991	-0.32518	590.267	-0.43296
592.506	-0.38473	591.991	-0.30402	591.267	-0.40136
593.506	-0.35099	592.991	-0.2783	592.267	-0.36868
594.506	-0.32129	593.991	-0.2542	593.267	-0.33261
595.506	-0.29591	594.991	-0.23476	594.267	-0.30052
596.506	-0.27027	595.991	-0.21818	595.267	-0.27642
597.506	-0.24898	596.991	-0.20155	596.267	-0.25391
598.506	-0.22748	597.991	-0.18664	597.267	-0.23502
599.506	-0.20283	598.991	-0.16694	598.267	-0.21347
600.506	-0.1825	599.991	-0.14607	599.267	-0.188
601.506	-0.16537	600.991	-0.13468	600.267	-0.16777
602.506	-0.15061	601.991	-0.12305	601.267	-0.15416
603.506	-0.13653	602.991	-0.11069	602.267	-0.14102
604.506	-0.12316	603.991	-0.09722999	603.267	-0.12692
605.506	-0.11024	604.991	-0.08577606	604.267	-1.12E-01
606.506	-0.1016	605.991	-0.07739197	605.267	-0.09789372
607.506	-0.09507804	606.991	-0.07526093	606.267	-0.08710713
608.506	-0.08802421	607.991	-0.07403379	607.267	-0.08036094
609.506	-0.08168536	608.991	-0.06768737	608.267	-0.07290673
610.506	-0.07469582	609.991	-0.06267324	609.267	-0.06463049
611.506	-0.06839401	610.991	-0.05894575	610.267	-0.05686792
612.506	-0.06627211	611.991	-0.05853781	611.267	-0.05074614
613.506	-0.06428333	612.991	-0.05765905	612.267	-0.04669449
614.506	-0.06018297	613.991	-0.05496313	613.267	-0.04257661
615.506	-0.05965751	614.991	-0.0512747	614.267	-0.03932831
616.506	-0.06048778	615.991	-0.04982428	615.267	-0.03953421
617.506	-0.06283941	616.991	-0.04970973	616.267	-0.04085351
618.506	-0.06741172	617.991	-0.0516667	617.267	-0.04268522
619.506	-0.07280922	618.991	-0.05537483	618.267	-0.04719158
620.506	-0.07340328	619.991	-0.0577666	619.267	-0.05145792
621.506	-0.07085222	620.991	-5.55E-02	620.267	-0.05367136
622.506	-0.06841358	621.991	-5.30E-02	621.267	-0.05468854
623.506	-0.06422466	622.991	-0.05138073	622.267	-0.05476994
624.506	-0.06111661	623.991	-0.0490254	623.267	-0.05238303

625.506	-0.05930879	624.991	-0.04973839	624.267	-0.05113249
626.506	-0.05471738	625.991	-0.05070965	625.267	-0.05138704
627.506	-0.04983364	626.991	-0.04862177	626.267	-0.04865804
628.506	-0.04823749	627.991	-0.04621259	627.267	-0.04548329
629.506	-0.04959298	628.991	-0.04962527	628.267	-0.04499428
630.506	-0.05275774	629.991	-0.05471044	629.267	-0.04760354
631.506	-0.05789655	630.991	-0.06039792	630.267	-0.05200242
632.506	-0.06292617	631.991	-0.06654256	631.267	-0.05771017
633.506	-0.06379532	632.991	-0.07055838	632.267	-0.06154697
634.506	-0.06500526	633.991	-0.06987966	633.267	-0.06431353
635.506	-0.06634468	634.991	-0.06902789	634.267	-0.06707053
636.506	-0.06740755	635.991	-0.06822268	635.267	-0.06971224
637.506	-0.07156174	636.991	-0.06878872	636.267	-0.07035406
638.506	-0.07394774	637.991	-0.07143503	637.267	-0.07250118
639.506	-0.07292299	638.991	-0.07055789	638.267	-0.07360363
640.506	-0.07168403	639.991	-6.79E-02	639.267	-0.07002267
641.506	-0.07254699	640.991	-6.38E-02	640.267	-0.06682732
642.506	-0.07343057	641.991	-0.06259666	641.267	-0.06276091
643.506	-7.45E-02	642.991	-0.06490382	642.267	-0.05887256
644.506	-0.07424019	643.991	-0.06880683	643.267	-0.05623568
645.506	-0.06664471	644.991	-0.0670512	644.267	-0.05583581
646.506	-0.06221384	645.991	-0.05870098	645.267	-0.04867592
647.506	-0.06396663	646.991	-0.05604285	646.267	-0.0415708
648.506	-0.06584958	647.991	-0.05742361	647.267	-0.04121421
649.506	-0.06566176	648.991	-0.06115924	648.267	-0.04273987
650.506	-0.06586553	649.991	-0.06382278	649.267	-0.04517713
651.506	-0.06392606	650.991	-0.06201334	650.267	-0.04740003
652.506	-0.06371623	651.991	-5.87E-02	651.267	-0.04636585
653.506	-0.07223759	652.991	-0.06151103	652.267	-0.04406846
654.506	-0.0774992	653.991	-0.07065995	653.267	-0.05012225
655.506	-0.07443548	654.991	-0.072962	654.267	-0.05680572
656.506	-0.07071829	655.991	-0.06909173	655.267	-0.05765815
657.506	-0.07009836	656.991	-0.06408626	656.267	-0.05668005
658.506	-0.07002949	657.991	-0.06154646	657.267	-0.05561722
659.506	-0.07074624	658.991	-0.06330495	658.267	-0.05562253
660.506	-0.06797167	659.991	-0.06284806	659.267	-0.05864111
661.506	-0.05920286	660.991	-0.05666602	660.267	-0.05961907
662.506	-0.05178405	661.991	-0.04857315	661.267	-0.05354956
663.506	-0.0510038	662.991	-0.04658847	662.267	-0.04667635
664.506	-0.05080912	663.991	-0.04953192	663.267	-0.04274486
665.506	-0.04931824	664.991	-5.30E-02	664.267	-0.0400492
666.506	-0.04546934	665.991	-0.05397235	665.267	-0.03855122
667.506	-0.04239333	666.991	-0.04926682	666.267	-0.0364563

668.506	-0.0482098	667.991	-0.05095977	667.267	-0.03175151
669.506	-0.05551873	668.991	-0.05826762	668.267	-0.03397656
670.506	-0.06233485	669.991	-0.06499272	669.267	-0.0410945
671.506	-0.06444606	670.991	-0.06801273	670.267	-0.04938088
672.506	-0.06514194	671.991	-0.06819488	671.267	-0.05588097
673.506	-0.06459044	672.991	-0.06659616	672.267	-0.05999658
674.506	-0.06637686	673.991	-0.06509088	673.267	-0.06134317
675.506	-0.07066333	674.991	-0.06921244	674.267	-0.06257181
676.506	-0.06393494	675.991	-0.06847147	675.267	-0.06841099
677.506	-0.05595857	676.991	-0.06219228	676.267	-0.06638974
678.506	-0.04813195	677.991	-0.0544115	677.267	-0.05914521
679.506	-0.04419957	678.991	-0.04845383	678.267	-0.04922058
680.506	-0.04127004	679.991	-0.04563507	679.267	-4.09E-02
681.506	-0.03860888	680.991	-0.04407551	680.267	-0.03504869
682.506	-0.0361603	681.991	-0.04321812	681.267	-0.03045848
683.506	-0.03193816	682.991	-0.04149627	682.267	-0.025545
684.506	-0.03165553	683.991	-0.03882421	683.267	-0.01891417
685.506	-0.03577726	684.991	-0.04031072	684.267	-0.0142659
686.506	-0.03899741	685.991	-0.04657375	685.267	-0.01636723
687.506	-0.0366147	686.991	-0.04766283	686.267	-0.02210412
688.506	-0.03419565	687.991	-0.0428979	687.267	-0.02317536
689.506	-0.03387872	688.991	-0.03788475	688.267	-0.02190098
690.506	-0.03188248	689.991	-0.03232305	689.267	-0.01996251
691.506	-0.03188804	690.991	-0.02589369	690.267	-0.01774786
692.506	-0.03433374	691.991	-0.02496566	691.267	-0.01565717
693.506	-0.02958388	692.991	-0.02123521	692.267	-1.82E-02
694.506	-0.02503358	693.991	-0.01123553	693.267	-0.0153277
695.506	-0.0268742	694.991	-8.18E-03	694.267	-0.00872353
696.506	-0.02986422	695.991	-0.01050873	695.267	-0.008814008
697.506	-0.03159825	696.991	-0.0145755	696.267	-0.01155101
698.506	-3.55E-02	697.991	-0.0204174	697.267	-0.01643313
699.506	-0.0342927	698.991	-0.02595202	698.267	-0.0242046
700.506	-0.02915024	699.991	-0.02508719	699.267	-0.02995074
701.506	-0.03080994	700.991	-0.02601735	700.267	-0.02932681
702.506	-0.03253334	701.991	-0.0302756	701.267	-0.03087402
703.506	-0.03124381	702.991	-0.0312652	702.267	-0.0339866
704.506	-0.03058471	703.991	-0.03284663	703.267	-0.03332487
705.506	-0.02931937	704.991	-3.40E-02	704.267	-0.03258619
706.506	-0.02498478	705.991	-0.03279107	705.267	-0.03056976
707.506	-0.02301466	706.991	-0.0306338	706.267	-0.02481548
708.506	-0.02375446	707.991	-0.031039	707.267	-0.01953264
709.506	-0.02179871	708.991	-0.0323822	708.267	-0.01716226
710.506	-0.0206572	709.991	-0.03292478	709.267	-0.01655064

711.506	-0.02147271	710.991	-0.03443957	710.267	-0.01682784
712.506	-0.01818115	711.991	-0.03220211	711.267	-0.02050041
713.506	-0.01326735	712.991	-0.02659346	712.267	-0.02215634
714.506	-0.01037931	713.991	-0.02111061	713.267	-0.02124728
715.506	-0.00841321	714.991	-0.01734105	714.267	-0.02135638
716.506	-0.00573213	715.991	-0.01436728	715.267	-0.02275046
717.506	-0.00194048	716.991	-0.009417495	716.267	-0.02398246
718.506	0.001441704	717.991	-0.005500857	717.267	-0.0214885
719.506	0.003652962	718.991	-0.003669179	718.267	-0.01774485
720.506	0.004067345	719.991	-0.005248904	719.267	-0.01344726
721.506	0.002940096	720.991	-0.00721279	720.267	-0.01067735
722.506	-0.00034245	721.991	-0.01175889	721.267	-0.009326095
723.506	-0.0020836	722.991	-0.01605296	722.267	-0.01137386
724.506	-0.00245429	723.991	-0.01810219	723.267	-0.01168739
725.506	-0.00303374	724.991	-0.02010766	724.267	-0.01046141
726.506	-0.00210017	725.991	-0.01954258	725.267	-0.01012059
727.506	2.11E-03	726.991	-0.01522152	726.267	-0.009424979
728.506	0.006072818	727.991	-0.008066277	727.267	-0.006327084
729.506	0.006172424	728.991	-0.005070442	728.267	-0.002341319
730.506	0.008965783	729.991	-0.002501802	729.267	-0.002408912
731.506	0.008272962	730.991	-0.000480169	730.267	0.000180186
732.506	0.005708439	731.991	-0.001370631	731.267	0.000476893
733.506	0.001147572	732.991	-0.003411174	732.267	-0.001123392
734.506	-0.00619371	733.991	-0.008717508	733.267	-0.004104953
735.506	-0.01463519	734.991	-0.01706524	734.267	-0.009884344
736.506	-0.02290954	735.991	-0.02536568	735.267	-0.0180161
737.506	-0.02668493	736.991	-0.0312355	736.267	-0.02509768
738.506	-0.02943736	737.991	-0.03252758	737.267	-0.02829296
739.506	-0.02903767	738.991	-0.03147636	738.267	-2.97E-02
740.506	-2.83E-02	739.991	-0.02882437	739.267	-0.02915836
741.506	-0.02672717	740.991	-0.02593978	740.267	-2.87E-02
742.506	-0.0224017	741.991	-0.02124472	741.267	-0.0288091
743.506	-0.01624003	742.991	-0.01396474	742.267	-0.02731358
744.506	-0.01032713	743.991	-0.007970072	743.267	-0.0217089
745.506	-0.00605864	744.991	-2.79E-03	744.267	-0.01640973
746.506	-0.00291306	745.991	-0.000141028	745.267	-0.01253038
747.506	-0.0014715	746.991	0.00080882	746.267	-0.009975608
748.506	9.61117E-05	747.991	0.000339965	747.267	-0.008289118
749.506	0.002808904	748.991	0.001133457	748.267	-0.006694132
750.506	0.002655333	749.991	0.000847178	749.267	-0.002369828
751.506	0.000173459	750.991	-0.001396963	750.267	1.37E-03
752.506	-2.98E-03	751.991	-0.003176955	751.267	0.001499808
753.506	-0.00452098	752.991	-0.005084567	752.267	4.89376E-05

754.506	-0.00468418	753.991	-0.005728046	753.267	-0.000756956
755.506	-0.00483503	754.991	-0.006196906	754.267	-0.001469124
756.506	-0.0047054	755.991	-0.006025534	755.267	-0.003483599
757.506	-0.00539022	756.991	-0.007296033	756.267	-0.004984308
758.506	-0.00157012	757.991	-0.006827735	757.267	-0.008220625
759.506	0.005240926	758.991	-0.002632147	758.267	-0.007414627
760.506	0.01091219	759.991	0.001843237	759.267	-0.002631473
761.506	0.01223941	760.991	0.003961622	760.267	0.002633521
762.506	0.01011897	761.991	0.002825464	761.267	0.003556056
763.506	0.0103669	762.991	0.001543781	762.267	0.002179874
764.506	0.01176112	763.991	0.002133637	763.267	0.004248676
765.506	0.01041474	764.991	0.001955121	764.267	0.008068688
766.506	4.90E-03	765.991	-0.001876502	765.267	0.01102538
767.506	-3.82E-03	766.991	-0.009682442	766.267	0.007538659
768.506	-9.80E-03	767.991	-0.01877703	767.267	-0.00021065
769.506	-8.96E-03	768.991	-0.02191223	768.267	-0.007682595
770.506	-0.00535268	769.991	-0.02048157	769.267	-0.00660393
771.506	-0.00472947	770.991	-0.01959513	770.267	-0.001009178
772.506	-0.00674453	771.991	-0.02099707	771.267	0.000280336
773.506	-6.14E-03	772.991	-0.02301339	772.267	-0.001175843
774.506	-0.00367644	773.991	-0.02178224	773.267	-0.003614302
775.506	0.000421614	774.991	-1.90E-02	774.267	-0.00322588
776.506	0.004829242	775.991	-1.48E-02	775.267	-0.001780181
777.506	0.005553005	776.991	-0.01303531	776.267	0.000334893
778.506	4.49E-03	777.991	-0.01511329	777.267	-0.001805407
779.506	0.005038534	778.991	-0.01672365	778.267	-0.007586968
780.506	0.007576533	779.991	-0.01661243	779.267	-0.009776017
781.506	0.01142985	780.991	-0.01270761	780.267	-0.009702761
782.506	0.01570177	781.991	-0.008581199	781.267	-0.006015092
783.506	0.01998725	782.991	-0.004115448	782.267	-0.001143603
784.506	0.0213735	783.991	-0.000724703	783.267	0.004912346
785.506	0.02254829	784.991	0.001510249	784.267	0.009353765
786.506	0.02364752	785.991	0.005283075	785.267	0.01333233
787.506	0.0210237	786.991	0.007290934	786.267	0.0179498
788.506	0.01649581	787.991	0.006306032	787.267	0.01829021
789.506	0.009712589	788.991	0.002293052	788.267	0.01642216
790.506	0.002622684	789.991	-0.001692636	789.267	0.01230693
791.506	-0.00926923	790.991	-0.007842298	790.267	0.00741506
792.506	-0.02199623	791.991	-0.02007807	791.267	-0.000488509
793.506	-0.02886594	792.991	-0.02511842	792.267	-0.0103206
794.506	-0.03972852	793.991	-0.02644467	793.267	-0.008583367
795.506	-0.04789687	794.991	-0.02976116	794.267	-1.03E-02
796.506	-0.05458014	795.991	-0.03448211	795.267	-0.009271684

796.991	0.03789078	796.267	-0.001024447
		797.267	0.1631

temperature	DTG	temperature	DTG
℃	%/min	℃	%/min
	Coal+TPPI		Coal+PA
28.57	-0.52598	31.33	0.03909318
29.57	-0.09176765	32.33	-0.15234
30.57	-0.12805	33.33	-0.15188
31.57	-0.13594	34.33	-0.16049
32.57	-0.11478	35.33	-0.15107
33.57	-0.09219189	36.33	-0.1272
34.57	-0.07361996	37.33	-0.10344
35.57	-0.06165938	38.33	-0.08665718
36.57	-0.05785972	39.33	-0.07741984
37.57	-0.05919021	40.33	-0.06774016
38.57	-0.0696934	41.33	-0.06192744
39.57	-0.08112217	42.33	-0.05255377
40.57	-0.09224973	43.33	-0.04680651
41.57	-0.09852165	44.33	-0.03871308
42.57	-0.10579	45.33	-0.03898706
43.57	-0.11834	46.33	-0.03595661
44.57	-0.12486	47.33	-0.03603282
45.57	-0.12934	48.33	-0.03480068
46.57	-0.13009	49.33	-0.03112502
47.57	-0.12886	50.33	-0.03068658
48.57	-0.13043	51.33	-0.03072037
49.57	-0.13578	52.33	-0.02406061
50.57	-0.1372	53.33	-0.02007811
51.57	-0.13254	54.33	-0.01460048
52.57	-0.12875	55.33	-0.0092991
53.57	-0.12524	56.33	-0.00982687
54.57	-0.12132	57.33	-0.01221604
55.57	-0.12128	58.33	-0.01095623
56.57	-0.12356	59.33	-0.00700822
57.57	-0.12345	60.33	-0.00595841
58.57	-0.12011	61.33	-0.00563218
59.57	-0.11775	62.33	-0.00739522
60.57	-0.11805	63.33	-0.01244459
61.57	-0.11922	64.33	-0.01454224
62.57	-0.12142	65.33	-0.01330958
63.57	-0.12516	66.33	-0.0110675
64.57	-0.12594	67.33	-0.01001152
65.57	-0.12482	68.33	-0.01120189
66.57	-0.12315	69.33	-0.0135711
67.57	-0.12225	70.33	-0.01673717

68.57	-0.12194	71.33	-0.02015035
69.57	-0.12165	72.33	-0.02083823
70.57	-0.12215	73.33	-0.02053487
71.57	-0.12397	74.33	-0.0176501
72.57	-0.12442	75.33	-0.01523683
73.57	-0.12373	76.33	-0.01302571
74.57	-0.12176	77.33	-0.01284624
75.57	-0.11869	78.33	-0.01243573
76.57	-0.11477	79.33	-0.01129675
77.57	-0.11278	80.33	-0.0076076
78.57	-0.11192	81.33	-0.00182505
79.57	-0.11029	82.33	0.00142344
80.57	-0.10681	83.33	0.00167512
81.57	-0.10097	84.33	-0.00110988
82.57	-0.09582625	85.33	-0.00496869
83.57	-0.09321066	86.33	-0.00757016
84.57	-0.0934909	87.33	-0.00836071
85.57	-0.095686	88.33	-0.0100898
86.57	-0.09875037	89.33	-0.01256566
87.57	-0.09936713	90.33	-0.01721246
88.57	-0.1006	91.33	-0.02198601
89.57	-0.1028	92.33	-0.02582693
90.57	-0.1068	93.33	-0.0297316
91.57	-0.11102	94.33	-0.03234927
92.57	-0.11345	95.33	-0.03359821
93.57	-0.11558	96.33	-0.03427473
94.57	-0.11615	97.33	-0.03361611
95.57	-0.11366	98.33	-0.03253871
96.57	-0.1101	99.33	-0.03046954
97.57	-0.1058	100.33	-0.02697383
98.57	-0.09991417	101.33	-0.02384416
99.57	-0.09340835	102.33	-0.01902595
100.57	-0.08300028	103.33	-0.01502491
101.57	-0.07376703	104.33	-0.01224386
102.57	-0.06478008	105.33	-0.01129117
103.57	-0.05651536	106.33	-0.01235299
104.57	-0.05140512	107.33	-0.01274833
105.57	-0.0490219	108.33	-0.01198273
106.57	-0.0490976	109.33	-0.01476666
107.57	-0.0503351	110.33	-0.01778513
108.57	-0.05088308	111.33	-0.02126404
109.57	-0.05750455	112.33	-0.02284124
110.57	-0.06581111	113.33	-0.02363701

111.57	-0.07350185	114.33	-0.02483586
112.57	-0.07897057	115.33	-0.02415069
113.57	-0.08235259	116.33	-0.02407553
114.57	-0.08503695	117.33	-0.02568363
115.57	-0.08539726	118.33	-0.02675734
116.57	-0.08480322	119.33	-0.02650117
117.57	-0.08449366	120.33	-0.02607553
118.57	-0.08187866	121.33	-0.02725456
119.57	-0.07583418	122.33	-0.02822728
120.57	-0.06956925	123.33	-0.02699796
121.57	-0.0654929	124.33	-0.02611955
122.57	-0.06176505	125.33	-0.02507866
123.57	-0.05604701	126.33	-0.02301448
124.57	-0.05062108	127.33	-0.02108373
125.57	-0.0454856	128.33	-0.0210236
126.57	-0.04014976	129.33	-0.02080889
127.57	-0.03614025	130.33	-0.01949356
128.57	-0.03538925	131.33	-0.01820641
129.57	-0.03640163	132.33	-0.02017348
130.57	-0.03719858	133.33	-0.02447905
131.57	-0.0386536	134.33	-0.03005969
132.57	-0.04374716	135.33	-0.0350906
133.57	-0.05014798	136.33	-0.03567955
134.57	-0.05738657	137.33	-0.03530847
135.57	-0.0648459	138.33	-0.03615799
136.57	-0.0682483	139.33	-0.0392268
137.57	-0.06966329	140.33	-0.04230134
138.57	-0.06882455	141.33	-0.04107808
139.57	-0.06847478	142.33	-0.03660443
140.57	-0.06684611	143.33	-0.030944
141.57	-0.06174521	144.33	-0.02854117
142.57	-0.05544419	145.33	-0.03001339
143.57	-0.04728615	146.33	-0.0328333
144.57	-0.04161075	147.33	-0.03358212
145.57	-0.03843756	148.33	-0.03276538
146.57	-0.03729453	149.33	-0.03266819
147.57	-0.03578597	150.33	-0.03464326
148.57	-0.03383164	151.33	-0.03868067
149.57	-0.03351582	152.33	-0.04373488
150.57	-0.03439062	153.33	-0.04809694
151.57	-0.03690754	154.33	-0.04997098
152.57	-0.04056234	155.33	-0.0505772
153.57	-0.04364341	156.33	-0.05195657

154.57	-0.0463375	157.33	-0.05338837
155.57	-0.04817033	158.33	-0.05480515
156.57	-0.05047478	159.33	-0.0561467
157.57	-0.05216311	160.33	-0.05512667
158.57	-0.05327291	161.33	-0.0499842
159.57	-0.05319159	162.33	-0.04404473
160.57	-0.05162249	163.33	-0.04120242
161.57	-0.04843053	164.33	-0.03979318
162.57	-0.04439939	165.33	-0.03815934
163.57	-0.04154099	166.33	-0.03633726
164.57	-0.03967919	167.33	-0.03293767
165.57	-0.03967387	168.33	-0.02913655
166.57	-0.0409509	169.33	-0.0293551
167.57	-0.04255236	170.33	-0.03364368
168.57	-0.04477733	171.33	-0.03519504
169.57	-0.04700345	172.33	-0.03388803
170.57	-0.04965188	173.33	-0.03261697
171.57	-0.05072995	174.33	-0.03207635
172.57	-0.05027306	175.33	-0.03248972
173.57	-0.04779285	176.33	-0.03422466
174.57	-0.04339637	177.33	-0.03538272
175.57	-0.03913381	178.33	-0.03399646
176.57	-0.03503498	179.33	-0.03184958
177.57	-0.03145304	180.33	-0.03091233
178.57	-0.02861113	181.33	-0.03054232
179.57	-0.02655192	182.33	-0.03013368
180.57	-0.02553769	183.33	-0.02984435
181.57	-0.02566888	184.33	-0.02977662
182.57	-0.02708301	185.33	-0.0289501
183.57	-0.02915919	186.33	-0.02746107
184.57	-0.0316054	187.33	-0.02691871
185.57	-0.03350635	188.33	-0.02673238
186.57	-0.03437669	189.33	-0.02578971
187.57	-0.03489135	190.33	-0.02520645
188.57	-0.03516949	191.33	-0.02425491
189.57	-0.0342233	192.33	-0.02283914
190.57	-0.03355859	193.33	-0.02221723
191.57	-0.03204867	194.33	-0.02264524
192.57	-0.03000235	195.33	-0.0235168
193.57	-0.02775273	196.33	-0.02484667
194.57	-0.02583904	197.33	-0.02798285
195.57	-0.02494841	198.33	-0.02891314
196.57	-0.02553082	199.33	-0.02817517

197.57	-0.02791925	200.33	-0.02659427
198.57	-0.02755098	201.33	-0.02329869
199.57	-0.02610983	202.33	-0.0200438
200.57	-0.02371618	203.33	-0.01639277
201.57	-0.02093523	204.33	-0.01245852
202.57	-0.02020084	205.33	-0.00631135
203.57	-0.01993084	206.33	-0.00141344
204.57	-0.01837658	207.33	0.00027738
205.57	-0.01440924	208.33	0.00044235
206.57	-0.01286511	209.33	-0.00067662
207.57	-0.01432802	210.33	-0.00105456
208.57	-0.01747526	211.33	-0.00150028
209.57	-0.02201658	212.33	-0.0015534
210.57	-0.0257343	213.33	-0.00064864
211.57	-0.02792369	214.33	0.00045334
212.57	-0.02834906	215.33	0.0032932
213.57	-0.02699946	216.33	0.00697189
214.57	-0.0236118	217.33	0.01113735
215.57	-0.01681441	218.33	0.01301598
216.57	-0.00866303	219.33	0.01412147
217.57	0.000620196	220.33	0.0152948
218.57	0.01009769	221.33	0.01473308
219.57	0.01894789	222.33	0.01369868
220.57	0.02674662	223.33	0.01202785
221.57	0.03141298	224.33	0.01019333
222.57	0.0330164	225.33	0.00893187
223.57	0.03185335	226.33	0.01081413
224.57	0.02998081	227.33	0.01543776
225.57	0.02771437	228.33	0.01953198
226.57	0.026333	229.33	0.02315934
227.57	0.02652798	230.33	0.02572322
228.57	0.0256229	231.33	0.02923021
229.57	0.02348555	232.33	0.03343621
230.57	0.02190743	233.33	0.03551377
231.57	0.02096059	234.33	0.03411955
232.57	0.01866884	235.33	0.0311612
233.57	0.01462456	236.33	0.02758274
234.57	0.01031963	237.33	0.02641747
235.57	0.005803029	238.33	0.02925886
236.57	0.003149959	239.33	0.03076714
237.57	0.003397304	240.33	0.03049766
238.57	0.006234376	241.33	0.03202966
239.57	0.01081536	242.33	0.03634093

240.57	0.01829898	243.33	0.04139384
241.57	0.02989739	244.33	0.04787467
242.57	0.042125	245.33	0.05237483
243.57	0.05386126	246.33	0.05229316
244.57	0.06385842	247.33	0.05281683
245.57	0.07202888	248.33	0.05567303
246.57	0.07760697	249.33	0.05943102
247.57	0.08178589	250.33	0.06329562
248.57	0.08394391	251.33	0.06548646
249.57	0.08291504	252.33	0.06717393
250.57	0.08033452	253.33	0.06891065
251.57	0.07716694	254.33	0.07292466
252.57	0.07295262	255.33	0.07832217
253.57	0.06867195	256.33	0.082308
254.57	0.06534079	257.33	0.08486242
255.57	0.06234754	258.33	0.08677906
256.57	0.06061837	259.33	0.08949268
257.57	0.05987156	260.33	0.09064749
258.57	0.06015295	261.33	0.09202508
259.57	0.06181641	262.33	0.09307155
260.57	0.06606086	263.33	0.09285988
261.57	0.07105933	264.33	0.09560153
262.57	0.07672691	265.33	0.09906099
263.57	0.08133853	266.33	0.10088
264.57	0.08515809	267.33	0.10091
265.57	0.08888955	268.33	0.10249
266.57	0.09302884	269.33	0.10474
267.57	0.09681197	270.33	0.10702
268.57	0.09850472	271.33	0.10833
269.57	0.10059	272.33	0.10504
270.57	0.10235	273.33	0.10003
271.57	0.10547	274.33	0.09749063
272.57	0.10881	275.33	0.09907655
273.57	0.11187	276.33	0.10175
274.57	0.11437	277.33	0.10343
275.57	0.1163	278.33	0.10581
276.57	0.11864	279.33	0.11053
277.57	0.12123	280.33	0.11815
278.57	0.1242	281.33	0.1271
279.57	0.12605	282.33	0.13483
280.57	0.12781	283.33	0.13987
281.57	0.12935	284.33	0.1437
282.57	0.12965	285.33	0.14916

283.57	0.12968	286.33	0.15285
284.57	0.13004	287.33	0.15457
285.57	0.12966	288.33	0.15588
286.57	0.12853	289.33	0.15695
287.57	0.12936	290.33	0.1587
288.57	0.1309	291.33	0.16085
289.57	0.13428	292.33	0.16209
290.57	0.1396	293.33	0.16116
291.57	0.14638	294.33	0.16144
292.57	0.15341	295.33	0.16249
293.57	0.16055	296.33	0.16227
294.57	0.16737	297.33	0.16268
295.57	0.17205	298.33	0.16204
296.57	0.17577	299.33	0.16157
297.57	0.17723	300.33	0.16235
298.57	0.1778	301.33	0.16484
299.57	0.17787	302.33	0.16658
300.57	0.17746	303.33	0.1659
301.57	0.17638	304.33	0.16624
302.57	0.17476	305.33	0.16643
303.57	0.17347	306.33	0.16673
304.57	0.17114	307.33	0.16527
305.57	0.16865	308.33	0.16283
306.57	0.16542	309.33	0.15781
307.57	0.15983	310.33	0.1533
308.57	0.15367	311.33	0.15073
309.57	0.14867	312.33	0.14698
310.57	0.14515	313.33	0.14235
311.57	0.14211	314.33	0.13699
312.57	0.14149	315.33	0.13495
313.57	0.14263	316.33	0.13328
314.57	0.14463	317.33	0.13402
315.57	0.1482	318.33	0.13486
316.57	0.15234	319.33	0.13632
317.57	0.15569	320.33	0.13803
318.57	0.15747	321.33	0.13968
319.57	0.15765	322.33	0.14219
320.57	0.15509	323.33	0.13996
321.57	0.15086	324.33	0.13654
322.57	0.14505	325.33	0.1308
323.57	0.13872	326.33	0.12324
324.57	0.13115	327.33	0.1152
325.57	0.12224	328.33	0.10692

326.57	0.11243	329.33	0.0974946
327.57	0.10389	330.33	0.08604585
328.57	0.09658756	331.33	0.07773489
329.57	0.0889596	332.33	0.07042081
330.57	0.08188914	333.33	0.06367268
331.57	0.0740771	334.33	0.05772676
332.57	0.06586531	335.33	0.04956106
333.57	0.05960074	336.33	0.0419731
334.57	0.05488963	337.33	0.03576385
335.57	0.04906373	338.33	0.03059812
336.57	0.04228479	339.33	0.02421283
337.57	0.03518406	340.33	0.01673692
338.57	0.02837375	341.33	0.00996674
339.57	0.02300988	342.33	0.00360131
340.57	0.01798984	343.33	-0.0017602
341.57	0.01077813	344.33	-0.00825674
342.57	0.001768451	345.33	-0.01549925
343.57	-0.007583619	346.33	-0.02343668
344.57	-0.01713508	347.33	-0.03198274
345.57	-0.02768206	348.33	-0.03975082
346.57	-0.03931689	349.33	-0.04956655
347.57	-0.05278687	350.33	-0.05972668
348.57	-0.0665374	351.33	-0.06915445
349.57	-0.0783311	352.33	-0.07793453
350.57	-0.08855081	353.33	-0.08792214
351.57	-0.09698055	354.33	-0.09853423
352.57	-0.1049	355.33	-0.10853
353.57	-0.11118	356.33	-0.11913
354.57	-0.11687	357.33	-0.12754
355.57	-0.12247	358.33	-0.13638
356.57	-0.12782	359.33	-0.14716
357.57	-0.13455	360.33	-0.15853
358.57	-0.14267	361.33	-0.16826
359.57	-0.15246	362.33	-0.17648
360.57	-0.16304	363.33	-0.18381
361.57	-0.17462	364.33	-0.19197
362.57	-0.18716	365.33	-0.20221
363.57	-0.19782	366.33	-0.21252
364.57	-0.20914	367.33	-0.22257
365.57	-0.22064	368.33	-0.23198
366.57	-0.23317	369.33	-0.24264
367.57	-0.24647	370.33	-0.25391
368.57	-0.25834	371.33	-0.26672

369.57	-0.26907	372.33	-0.27777
370.57	-0.2788	373.33	-0.28755
371.57	-0.28996	374.33	-0.29632
372.57	-0.30048	375.33	-0.30312
373.57	-0.30903	376.33	-0.3107
374.57	-0.31436	377.33	-0.31892
375.57	-0.31804	378.33	-0.32643
376.57	-0.32391	379.33	-0.33232
377.57	-0.33078	380.33	-0.33839
378.57	-0.33574	381.33	-0.34433
379.57	-0.33861	382.33	-0.35022
380.57	-0.34057	383.33	-0.35817
381.57	-0.34408	384.33	-0.36357
382.57	-0.34983	385.33	-0.3643
383.57	-0.35707	386.33	-0.36541
384.57	-0.36142	387.33	-0.36645
385.57	-0.3644	388.33	-0.36796
386.57	-0.37131	389.33	-0.37074
387.57	-0.3798	390.33	-0.37377
388.57	-0.38827	391.33	-0.3752
389.57	-0.3959	392.33	-0.37703
390.57	-0.40129	393.33	-0.38138
391.57	-0.40295	394.33	-0.38695
392.57	-0.40389	395.33	-0.39388
393.57	-0.40441	396.33	-0.40128
394.57	-0.40197	397.33	-0.40617
395.57	-0.4013	398.33	-0.41139
396.57	-0.40217	399.33	-0.41733
397.57	-0.40469	400.33	-0.42386
398.57	-0.41046	401.33	-0.43061
399.57	-0.4197	402.33	-0.43541
400.57	-0.4316	403.33	-0.43934
401.57	-0.44378	404.33	-0.44288
402.57	-0.45689	405.33	-0.44794
403.57	-0.46922	406.33	-0.45285
404.57	-0.48028	407.33	-0.45856
405.57	-0.48963	408.33	-0.46617
406.57	-0.49702	409.33	-0.47488
407.57	-0.50237	410.33	-0.48612
408.57	-0.50753	411.33	-0.49846
409.57	-0.51481	412.33	-0.51291
410.57	-0.52239	413.33	-0.52695
411.57	-0.52987	414.33	-0.54081

412.57	-0.53705	415.33	-0.55268
413.57	-0.54582	416.33	-0.56373
414.57	-0.55395	417.33	-0.57711
415.57	-0.56253	418.33	-0.58885
416.57	-0.57156	419.33	-0.5986
417.57	-0.58097	420.33	-0.6056
418.57	-0.59092	421.33	-0.61398
419.57	-0.60059	422.33	-0.62326
420.57	-0.61153	423.33	-0.63478
421.57	-0.62223	424.33	-0.64578
422.57	-0.63446	425.33	-0.6521
423.57	-0.64736	426.33	-0.65993
424.57	-0.65916	427.33	-0.66844
425.57	-0.66884	428.33	-0.67845
426.57	-0.67917	429.33	-0.68814
427.57	-0.69078	430.33	-0.6971
428.57	-0.70194	431.33	-0.70522
429.57	-0.71403	432.33	-0.71488
430.57	-0.7273	433.33	-0.7297
431.57	-0.74296	434.33	-0.74557
432.57	-0.76191	435.33	-0.76522
433.57	-0.78308	436.33	-0.78639
434.57	-0.80365	437.33	-0.8086
435.57	-0.82487	438.33	-0.83158
436.57	-0.84674	439.33	-0.85631
437.57	-0.86787	440.33	-0.88082
438.57	-0.88819	441.33	-0.90353
439.57	-0.90792	442.33	-0.92441
440.57	-0.92585	443.33	-0.94336
441.57	-0.94469	444.33	-0.96226
442.57	-0.96438	445.33	-0.98399
443.57	-0.98367	446.33	-1.00907
444.57	-1.00416	447.33	-1.03736
445.57	-1.02884	448.33	-1.06709
446.57	-1.05785	449.33	-1.09707
447.57	-1.08878	450.33	-1.12888
448.57	-1.11945	451.33	-1.16364
449.57	-1.14964	452.33	-1.20055
450.57	-1.184	453.33	-1.23347
451.57	-1.22373	454.33	-1.26745
452.57	-1.26627	455.33	-1.30164
453.57	-1.30524	456.33	-1.33583
454.57	-1.34343	457.33	-1.37119

455.57	-1.38063	458.33	-1.41121
456.57	-1.42092	459.33	-1.4494
457.57	-1.46798	460.33	-1.48754
458.57	-1.51076	461.33	-1.53469
459.57	-1.54594	462.33	-1.5786
460.57	-1.57843	463.33	-1.62258
461.57	-1.61676	464.33	-1.66657
462.57	-1.65759	465.33	-1.71259
463.57	-1.69941	466.33	-1.75854
464.57	-1.74079	467.33	-1.80668
465.57	-1.78233	468.33	-1.85679
466.57	-1.82698	469.33	-1.90306
467.57	-1.87822	470.33	-1.95128
468.57	-1.93418	471.33	-2.00555
469.57	-1.98796	472.33	-2.06408
470.57	-2.03971	473.33	-2.12611
471.57	-2.09867	474.33	-2.19226
472.57	-2.16069	475.33	-2.26093
473.57	-2.22811	476.33	-2.33358
474.57	-2.29258	477.33	-2.40659
475.57	-2.36167	478.33	-2.48388
476.57	-2.42815	479.33	-2.55701
477.57	-2.50116	480.33	-2.62953
478.57	-2.57435	481.33	-2.70371
479.57	-2.64846	482.33	-2.77387
480.57	-2.72199	483.33	-2.84539
481.57	-2.7901	484.33	-2.90783
482.57	-2.85955	485.33	-2.97551
483.57	-2.92553	486.33	-3.04284
484.57	-2.99286	487.33	-3.10729
485.57	-3.06125	488.33	-3.17499
486.57	-3.12991	489.33	-3.23323
487.57	-3.19146	490.33	-3.29355
488.57	-3.25305	491.33	-3.35259
489.57	-3.31136	492.33	-3.41709
490.57	-3.37155	493.33	-3.4769
491.57	-3.43273	494.33	-3.52919
492.57	-3.49234	495.33	-3.58209
493.57	-3.54602	496.33	-3.6254
494.57	-3.59546	497.33	-3.6739
495.57	-3.64109	498.33	-3.7185
496.57	-3.68238	499.33	-3.7608
497.57	-3.72519	500.33	-3.79809

498.57	-3.75706	501.33	-3.8313
499.57	-3.78407	502.33	-3.86482
500.57	-3.81066	503.33	-3.89433
501.57	-3.83346	504.33	-3.92252
502.57	-3.85295	505.33	-3.94537
503.57	-3.87176	506.33	-3.9626
504.57	-3.88849	507.33	-3.97501
505.57	-3.90086	508.33	-3.98448
506.57	-3.91193	509.33	-3.99115
507.57	-3.91895	510.33	-3.99325
508.57	-3.9232	511.33	-3.99216
509.57	-3.92342	512.33	-3.98686
510.57	-3.92125	513.33	-3.97763
511.57	-3.91386	514.33	-3.96726
512.57	-3.89963	515.33	-3.95099
513.57	-3.88539	516.33	-3.92654
514.57	-3.87205	517.33	-3.89815
515.57	-3.85198	518.33	-3.8644
516.57	-3.82273	519.33	-3.82657
517.57	-3.79155	520.33	-3.7885
518.57	-3.75524	521.33	-3.74575
519.57	-3.71781	522.33	-3.69953
520.57	-3.67672	523.33	-3.65495
521.57	-3.62862	524.33	-3.61539
522.57	-3.57129	525.33	-3.57318
523.57	-3.51648	526.33	-3.52858
524.57	-3.46612	527.33	-3.48651
525.57	-3.41142	528.33	-3.43704
526.57	-3.35598	529.33	-3.38929
527.57	-3.30122	530.33	-3.34518
528.57	-3.25207	531.33	-3.30177
529.57	-3.19996	532.33	-3.24749
530.57	-3.15839	533.33	-3.19982
531.57	-3.11138	534.33	-3.15627
532.57	-3.06329	535.33	-3.109
533.57	-3.0179	536.33	-3.0747
534.57	-2.97397	537.33	-3.03493
535.57	-2.93505	538.33	-2.99599
536.57	-2.89024	539.33	-2.95093
537.57	-2.85451	540.33	-2.92081
538.57	-2.81208	541.33	-2.88361
539.57	-2.77594	542.33	-2.84482
540.57	-2.73806	543.33	-2.81151

541.57	-2.70462	544.33	-2.77012
542.57	-2.67668	545.33	-2.73681
543.57	-2.63744	546.33	-2.69854
544.57	-2.60164	547.33	-2.66909
545.57	-2.56685	548.33	-2.62786
546.57	-2.53253	549.33	-2.59991
547.57	-2.49839	550.33	-2.56997
548.57	-2.46451	551.33	-2.53876
549.57	-2.42646	552.33	-2.50868
550.57	-2.38795	553.33	-2.47566
551.57	-2.35811	554.33	-2.44904
552.57	-2.32717	555.33	-2.42129
553.57	-2.29606	556.33	-2.39579
554.57	-2.26779	557.33	-2.3565
555.57	-2.23922	558.33	-2.32149
556.57	-2.21379	559.33	-2.28711
557.57	-2.19055	560.33	-2.255
558.57	-2.16261	561.33	-2.22392
559.57	-2.13074	562.33	-2.1857
560.57	-2.101	563.33	-2.14443
561.57	-2.06795	564.33	-2.10438
562.57	-2.03157	565.33	-2.07126
563.57	-1.99277	566.33	-2.03516
564.57	-1.95143	567.33	-1.9945
565.57	-1.90883	568.33	-1.95107
566.57	-1.86663	569.33	-1.9036
567.57	-1.82463	570.33	-1.85964
568.57	-1.78147	571.33	-1.81593
569.57	-1.73602	572.33	-1.76721
570.57	-1.68996	573.33	-1.71688
571.57	-1.64644	574.33	-1.66291
572.57	-1.60285	575.33	-1.61068
573.57	-1.5595	576.33	-1.55802
574.57	-1.51393	577.33	-1.50431
575.57	-1.46478	578.33	-1.44819
576.57	-1.41572	579.33	-1.39633
577.57	-1.36577	580.33	-1.34732
578.57	-1.31609	581.33	-1.29301
579.57	-1.26677	582.33	-1.23969
580.57	-1.21178	583.33	-1.18661
581.57	-1.15191	584.33	-1.1327
582.57	-1.09696	585.33	-1.0843
5.84E+02	-1.04389	586.33	-1.0351

584.57	-0.99152	587.33	-0.98009
585.57	-0.9449	588.33	-0.92738
586.57	-0.8984	589.33	-0.8812
587.57	-0.84914	590.33	-0.83876
588.57	-0.80752	591.33	-0.80206
589.57	-0.77006	592.33	-0.76536
590.57	-0.73408	593.33	-0.72493
591.57	-0.70205	594.33	-0.68739
592.57	-0.66727	595.33	-0.65542
593.57	-0.62779	596.33	-0.62364
594.57	-0.59024	597.33	-0.59336
595.57	-0.55875	598.33	-0.56071
596.57	-0.52758	599.33	-0.52233
597.57	-0.50001	600.33	-0.48823
598.57	-0.46924	601.33	-0.45651
599.57	-0.43505	602.33	-0.42796
600.57	-0.40741	603.33	-0.40076
601.57	-0.38481	604.33	-0.37451
602.57	-0.36515	605.33	-0.34885
603.57	-0.34535	606.33	-0.32899
604.57	-0.32381	607.33	-0.31298
605.57	-0.30295	608.33	-0.29631
606.57	-0.28617	609.33	-0.28432
607.57	-0.27302	610.33	-0.27164
608.57	-0.25899	611.33	-0.25735
609.57	-0.24597	612.33	-0.24424
610.57	-0.23124	613.33	-0.23157
611.57	-0.21663	614.33	-0.21702
612.57	-0.20461	615.33	-0.2051
613.57	-0.19204	616.33	-0.19461
614.57	-0.17973	617.33	-0.18197
615.57	-0.1691	618.33	-0.17266
616.57	-0.15795	619.33	-0.16639
617.57	-0.14757	620.33	-0.15969
618.57	-0.14074	621.33	-0.1516
619.57	-0.13469	622.33	-0.14459
620.57	-0.12561	623.33	-0.13768
621.57	-0.11571	624.33	-0.13121
622.57	-0.10646	625.33	-0.12626
623.57	-0.09650239	626.33	-0.11857
624.57	-0.08916092	627.33	-0.11119
625.57	-0.08220074	628.33	-0.10617
626.57	-0.07230696	629.33	-0.1036

627.57	-0.06457098	630.33	-0.10236
628.57	-0.06136776	631.33	-0.1009
629.57	-0.05984736	632.33	-0.10009
630.57	-0.05967958	633.33	-0.09871695
631.57	-0.06182523	634.33	-0.09742998
632.57	-0.06375554	635.33	-0.09322537
633.57	-0.06484368	636.33	-0.08754215
634.57	-0.06634459	637.33	-0.08693495
635.57	-0.06513139	638.33	-0.08392367
636.57	-0.06300781	639.33	-0.07669676
637.57	-0.06487214	640.33	-0.0689879
638.57	-0.06375318	641.33	-0.06000565
639.57	-0.05891653	642.33	-0.05215279
640.57	-0.05267518	643.33	-0.0492663
641.57	-0.04740327	644.33	-0.04769868
642.57	-0.04366313	645.33	-0.03613961
643.57	-0.04218533	646.33	-0.02719211
644.57	-0.03943721	647.33	-0.02658306
645.57	-0.02875736	648.33	-0.02801595
646.57	-0.02295793	649.33	-0.03145935
647.57	-0.02224567	650.33	-0.03409606
648.57	-0.02418601	651.33	-0.03200313
649.57	-0.02568431	652.33	-0.03084877
650.57	-0.02510749	6.53E+02	-0.04084025
651.57	-0.02225753	654.33	-0.05003987
652.57	-0.02209136	655.33	-0.05051966
653.57	-0.03262944	656.33	-0.04823925
654.57	-0.04002244	657.33	-0.04510561
655.57	-0.04063164	658.33	-0.04335221
656.57	-0.03756276	659.33	-0.04337395
657.57	-0.0349561	660.33	-0.03921257
658.57	-0.03498363	661.33	-0.02676187
659.57	-0.03633709	662.33	-0.01490303
660.57	-0.03356422	663.33	-0.00969017
661.57	-0.02233755	664.33	-0.00757762
662.57	-0.01282214	665.33	-0.00763641
663.57	-0.009353066	666.33	-0.00709714
664.57	-0.008835358	667.33	-0.00522565
665.57	-0.009043142	668.33	-0.01130169
666.57	-0.006206725	669.33	-0.0215409
667.57	-0.003314345	670.33	-0.03167191
668.57	-0.007892128	671.33	-0.03771479
669.57	-0.01549549	672.33	-0.04120331

670.57	-0.02118161	673.33	-0.04132806
671.57	-0.02259603	674.33	-0.04188239
672.57	-0.02355484	675.33	-0.04779324
673.57	-0.02345233	676.33	-0.04679082
674.57	-0.0272786	677.33	-0.04265212
675.57	-0.03557866	678.33	-0.03743202
676.57	-0.03622692	679.33	-0.03502688
677.57	-0.03456357	680.33	-0.03389053
678.57	-0.03325099	681.33	-0.03299049
679.57	-0.03498211	682.33	-0.03268928
680.57	-0.03481826	683.33	-0.02879742
681.57	-0.03379927	684.33	-0.02488873
682.57	-0.03086551	685.33	-0.0248866
683.57	-0.02265748	686.33	-0.02636879
684.57	-0.01583591	687.33	-0.02359189
685.57	-0.01406179	688.33	-0.020047
686.57	-0.0120933	6.89E+02	-0.01865487
687.57	-0.005305378	690.33	-0.01479563
688.57	-1.99574E-05	691.33	-0.01200814
689.57	0.003088873	692.33	-0.01448434
690.57	0.007053855	693.33	-0.01321195
691.57	0.007210455	6.94E+02	-0.01104725
692.57	0.004437205	695.33	-0.01469434
693.57	0.008632125	696.33	-0.01999231
694.57	0.01190324	697.33	-0.02559569
695.57	0.007810588	698.33	-0.03514879
696.57	0.000835815	6.99E+02	-0.04347839
697.57	-0.006586977	700.33	-0.04703421
698.57	-0.01572682	701.33	-0.05256755
699.57	-0.02079772	702.33	-0.05669122
700.57	-0.02380278	703.33	-0.05587405
701.57	-0.03333209	704.33	-0.05635086
702.57	-0.04270944	705.33	-0.05683761
703.57	-0.04638836	706.33	-0.05272243
704.57	-0.04586302	707.33	-0.04588496
705.57	-0.04361584	708.33	-0.04154384
706.57	-0.03840062	709.33	-0.03922594
707.57	-0.03447833	710.33	-0.03901729
708.57	-0.03149464	711.33	-0.04273999
709.57	-0.02580937	712.33	-0.0424482
710.57	-0.02107013	713.33	-0.03858783
711.57	-0.02004222	714.33	-0.0365452
712.57	-0.0194723	715.33	-0.03872301

713.57	-0.01856535	716.33	-0.03915704
714.57	-0.02060595	717.33	-0.03464456
715.57	-0.02401077	718.33	-0.03197294
716.57	-0.02628496	719.33	-0.03138449
717.57	-0.02677265	720.33	-0.03250816
718.57	-0.02592076	721.33	-0.03308719
719.57	-0.02504156	7.22E+02	-0.03707633
720.57	-0.02454243	723.33	-0.03957147
721.57	-0.02569052	724.33	-0.04266672
722.57	-0.02793765	725.33	-0.04738309
723.57	-0.02890767	726.33	-0.04730619
724.57	-0.03119065	727.33	-0.04330847
725.57	-0.03528709	728.33	-0.03920196
726.57	-0.03941182	729.33	-0.04183826
727.57	-0.04078358	730.33	-0.04173775
728.57	-0.04166115	731.33	-0.04353653
729.57	-0.04331827	732.33	-0.04652288
730.57	-0.04182436	733.33	-0.05305617
731.57	-0.04169892	7.34E+02	-0.06408347
732.57	-0.04113718	735.33	-0.07320527
733.57	-0.04202789	736.33	-0.08034496
734.57	-0.04553566	737.33	-0.08220851
735.57	-0.05056714	7.38E+02	-0.08245661
736.57	-0.05624194	739.33	-0.08017257
737.57	-0.05910983	740.33	-0.07800622
738.57	-0.06125131	7.41E+02	-0.07130983
739.57	-0.06250783	742.33	-0.06215427
740.57	-0.06359821	743.33	-0.05572368
741.57	-0.06259382	744.33	-0.05104593
742.57	-0.05950996	745.33	-0.0487508
743.57	-0.05401113	746.33	-0.0501625
744.57	-0.04947311	747.33	-0.05262858
745.57	-0.04784053	748.33	-0.05329375
746.57	-0.04804209	749.33	-0.0546472
747.57	-0.0492472	750.33	-0.057784
748.57	-0.05139406	7.51E+02	-0.05937001
749.57	-0.05391357	752.33	-0.06157704
750.57	-0.05703267	753.33	-0.06388401
751.57	-0.06300898	7.54E+02	-0.06131072
752.57	-0.06898416	755.33	-0.05908883
7.54E+02	-0.07182256	756.33	-0.05840229
754.57	-0.07202347	757.33	-0.05997974
755.57	-0.07140606	758.33	-0.05814436

756.57	-0.06868958	759.33	-0.05495645
757.57	-0.06573476	760.33	-0.05435839
758.57	-0.06012775	761.33	-0.05474872
759.57	-0.05178277	762.33	-0.05770242
760.57	-0.04469898	763.33	-0.05795682
761.57	-0.04159014	764.33	-0.05609491
762.57	-0.04287606	7.65E+02	-0.05590969
763.57	-0.04240221	766.33	-0.06041314
764.57	-0.04405368	767.33	-0.06842545
765.57	-0.04921445	768.33	-0.07222915
766.57	-0.05703876	769.33	-0.06887106
767.57	-0.06732915	770.33	-0.06425022
768.57	-0.07461172	771.33	-0.06320429
769.57	-0.07562085	772.33	-0.06447574
770.57	-0.07340579	773.33	-0.06444236
771.57	-0.07335745	774.33	-0.06099723
772.57	-0.0720373	775.33	-0.05542459
773.57	-0.06596671	776.33	-0.04841545
774.57	-0.05876589	777.33	-0.04532412
775.57	-0.05119537	778.33	-0.04588668
776.57	-0.04247384	779.33	-0.04519589
777.57	-0.03755383	780.33	-0.04284801
778.57	-0.03561059	781.33	-0.03633186
779.57	-0.03408382	782.33	-0.02936167
780.57	-0.03181794	783.33	-0.02287379
781.57	-0.02965758	784.33	-0.02103582
782.57	-0.02800279	785.33	-0.02092923
783.57	-0.02492226	786.33	-0.01785464
784.57	-0.02606599	787.33	-0.01698313
785.57	-0.02758495	788.33	-0.01748501
786.57	-0.02773903	789.33	-0.02107752
787.57	-0.02794407	790.33	-0.02397995
788.57	-0.02966504	791.33	-0.02964849
789.57	-0.03249204	792.33	-0.03478832
790.57	-0.03622766	7.93E+02	-0.04051173
791.57	-0.04227266	794.33	-0.04603986
792.57	-0.04214587	795.33	-0.04897867
793.57	-0.03074976	7.96E+02	-0.06272254
794.57	-0.02790916	797.33	-0.13168
795.57	-0.01861554		
796.57	0.0261212		

DTG of water-immersed brown coal

temperature	***DTG***	***temperature***	***DTG***	***temperature***	***DTG***
℃	%/min	℃	%/min	℃	%/min
	Coal		Coal+MgCl2		Coal+TEMPO
26.178	-0.33804	28.92	-0.7517	28.473	-0.65646
27.178	-0.25363	29.92	-0.40606	29.473	-0.42749
28.178	-0.3261	30.92	-0.4646	30.473	-0.5247
29.178	-0.3936	31.92	-0.49888	31.473	-0.54041
30.178	-0.4277	32.92	-0.5099	32.473	-0.56912
31.178	-0.42844	33.92	-0.493	33.473	-0.50163
32.178	-0.41541	34.92	-0.46269	34.473	-0.53352
33.178	-0.41745	35.92	-0.4522	35.473	-0.46488
34.178	-0.41079	36.92	-0.43466	36.473	-0.43302
35.178	-0.40923	37.92	-0.42479	37.473	-0.48304
36.178	-0.41178	38.92	-0.41938	38.473	-0.44496
37.178	-0.41725	39.92	-0.40782	39.473	-0.45243
38.178	-0.40963	40.92	-0.38427	40.473	-0.41846
39.178	-0.40399	41.92	-0.37238	41.473	-0.42916
40.178	-0.40541	42.92	-0.36163	42.473	-0.40297
41.178	-0.40653	43.92	-0.35306	43.473	-0.33957
42.178	-0.40788	44.92	-0.3485	44.473	-0.375
43.178	-0.41056	45.92	-0.34627	45.473	-0.36843
44.178	-0.41843	46.92	-0.35255	46.473	-0.35817
45.178	-0.42789	47.92	-0.35969	47.473	-0.34282
46.178	-0.44527	48.92	-0.35597	48.473	-0.39417
47.178	-0.45295	49.92	-0.35647	49.473	-0.37489
48.178	-0.4549	50.92	-0.35977	50.473	-0.40482
49.178	-0.45977	51.92	-0.36861	51.473	-0.37862
50.178	-0.46787	52.92	-0.36566	52.473	-0.33882
51.178	-0.48016	53.92	-0.36327	53.473	-0.37139
52.178	-0.48118	54.92	-0.36216	54.473	-0.38516
53.178	-0.47821	55.92	-0.36118	55.473	-0.42906
54.178	-0.47486	56.92	-0.36954	56.473	-0.3811
55.178	-0.4693	57.92	-0.38166	57.473	-0.35988
56.178	-0.46925	58.92	-0.38779	58.473	-0.37423
57.178	-0.47315	59.92	-0.39537	59.473	-0.35852
58.178	-0.47012	60.92	-0.40527	60.473	-0.38269
59.178	-0.46304	61.92	-0.42397	61.473	-0.38699
60.178	-0.45312	62.92	-0.44702	62.473	-0.38586
61.178	-0.44647	63.92	-0.47004	63.473	-0.31575

62.178	-0.44315	64.92	-0.49838	64.473	-0.32211
63.178	-0.44036	65.92	-0.52651	65.473	-0.3391
64.178	-0.43724	66.92	-0.54758	66.473	-0.33176
65.178	-0.43625	67.92	-0.57171	67.473	-0.33995
66.178	-0.43347	68.92	-0.60473	68.473	-0.33347
67.178	-0.42051	69.92	-0.6236	69.473	-0.36622
68.178	-0.42148	70.92	-0.63345	70.473	-0.36936
69.178	-0.4255	71.92	-0.64313	71.473	-0.26232
70.178	-0.41695	72.92	-0.65174	72.473	-0.36511
71.178	-0.40392	73.92	-0.65722	73.473	-0.38641
72.178	-0.39562	74.92	-0.64473	74.473	-0.26599
73.178	-0.39137	75.92	-0.62537	75.473	-0.25929
74.178	-0.38902	76.92	-0.6247	76.473	-0.29438
75.178	-0.37308	77.92	-0.60634	77.473	-0.3337
76.178	-0.3539	78.92	-0.58176	78.473	-0.3521
77.178	-0.35685	79.92	-0.57399	79.473	-0.22038
78.178	-0.34469	80.92	-0.56475	80.473	-0.18985
79.178	-0.32852	81.92	-0.54957	81.473	-0.35338
80.178	-0.33267	82.92	-0.5259	82.473	-0.26019
81.178	-0.33644	83.92	-0.5025	83.473	-0.24991
82.178	-0.33209	84.92	-0.5009	84.473	-0.34965
83.178	-0.31805	85.92	-0.49849	85.473	-0.30134
84.178	-0.301	86.92	-0.47739	86.473	-0.27293
85.178	-0.30639	87.92	-0.46409	87.473	-0.21381
86.178	-0.31231	88.92	-0.45575	88.473	-0.19267
87.178	-0.29808	89.92	-0.44257	89.473	-0.30696
88.178	-0.28873	90.92	-0.43234	90.473	-0.28916
89.178	-0.28336	91.92	-0.4285	91.473	-0.21839
90.178	-0.27013	92.92	-0.43221	92.473	-0.20724
91.178	-0.26149	93.92	-0.43739	93.473	-0.21437
92.178	-0.25716	94.92	-0.43585	94.473	-0.22191
93.178	-0.25815	95.92	-0.44208	95.473	-0.2295
94.178	-0.25744	96.92	-0.46203	96.473	-0.23363
95.178	-0.24505	97.92	-0.48516	97.473	-0.21753
96.178	-0.23542	98.92	-0.50445	98.473	-0.21906
97.178	-0.23469	99.92	-0.52811	99.473	-0.21338
98.178	-0.23264	100.92	-0.55433	100.473	-0.19791
99.178	-0.2246	101.92	-0.58059	101.473	-0.21747
100.178	-0.21716	102.92	-0.60299	102.473	-0.19264
101.178	-0.20989	103.92	-0.61965	103.473	-0.15965
102.178	-0.20642	104.92	-0.631	104.473	-0.17359
103.178	-0.20296	105.92	-0.63025	105.473	-0.19264
104.178	-0.19999	106.92	-0.6162	106.473	-0.22605

105.178	-0.19979	107.92	-0.60275	107.473	-0.21092
106.178	-0.19683	108.92	-0.58215	108.473	-0.20549
107.178	-0.19045	109.92	-0.55143	109.473	-0.20842
108.178	-0.1928	110.92	-0.51218	110.473	-0.17296
109.178	-0.19764	111.92	-0.46837	111.473	-0.18704
110.178	-0.2001	112.92	-0.42741	112.473	-0.24659
111.178	-0.19623	113.92	-0.38497	113.473	-0.23141
112.178	-0.19017	114.92	-0.35054	114.473	-0.21498
113.178	-0.18681	115.92	-0.32579	115.473	-0.17133
114.178	-0.17989	116.92	-0.29969	116.473	-0.17315
115.178	-0.17784	117.92	-0.27382	117.473	-0.16637
116.178	-0.18243	118.92	-0.25341	118.473	-0.16341
117.178	-0.18124	119.92	-0.2376	119.473	-0.21758
118.178	-0.17644	120.92	-0.22727	120.473	-0.21337
119.178	-0.1735	121.92	-0.21796	121.473	-0.18551
120.178	-0.17558	122.92	-0.21226	122.473	-0.19006
121.178	-0.18048	123.92	-0.20408	123.473	-0.18111
122.178	-0.18437	124.92	-0.19455	124.473	-0.20693
123.178	-0.19084	125.92	-0.1857	125.473	-0.20644
124.178	-0.19213	126.92	-0.17892	126.473	-0.20495
125.178	-0.18956	127.92	-0.17381	127.473	-0.19441
126.178	-0.18634	128.92	-0.171	128.473	-0.18709
127.178	-0.18512	129.92	-0.16701	129.473	-0.1865
128.178	-0.18476	130.92	-0.16428	130.473	-0.18457
129.178	-0.18177	131.92	-0.16666	131.473	-0.18985
130.178	-0.17742	132.92	-0.17038	132.473	-0.18189
131.178	-0.17326	133.92	-0.17459	133.473	-0.15484
132.178	-0.17121	134.92	-0.18397	134.473	-0.16468
133.178	-0.16833	135.92	-0.19337	135.473	-0.17184
134.178	-0.1689	136.92	-0.20371	136.473	-0.18328
135.178	-0.17161	137.92	-0.21677	137.473	-0.15905
136.178	-0.16994	138.92	-0.23527	138.473	-0.16881
137.178	-0.16723	139.92	-0.25348	139.473	-0.16318
138.178	-0.16752	140.92	-0.26113	140.473	-0.14986
139.178	-0.17128	141.92	-0.27075	141.473	-0.17733
140.178	-0.17392	142.92	-0.27684	142.473	-0.19745
141.178	-0.17105	143.92	-0.28232	143.473	-0.21928
142.178	-0.17166	144.92	-0.28388	144.473	-0.17379
143.178	-0.16883	145.92	-0.28185	145.473	-0.18167
144.178	-0.17015	146.92	-0.27642	146.473	-0.17464
145.178	-0.17112	147.92	-0.26005	147.473	-0.2158
146.178	-0.17218	148.92	-0.24681	148.473	-0.16587
147.178	-0.17167	149.92	-0.24075	149.473	-0.15683

148.178	-0.16365	150.92	-0.23292	150.473	-0.20124
149.178	-0.16061	151.92	-0.21824	151.473	-0.15387
150.178	-0.16172	152.92	-0.20503	152.473	-0.15026
151.178	-0.16209	153.92	-0.20131	153.473	-0.17089
152.178	-0.15315	154.92	-0.1928	154.473	-0.17693
153.178	-0.14922	155.92	-0.1853	155.473	-0.13023
154.178	-0.15136	156.92	-0.18449	156.473	-0.11687
155.178	-0.14584	157.92	-0.18626	157.473	-0.186
156.178	-0.14168	158.92	-0.18486	158.473	-0.14809
157.178	-0.14395	159.92	-0.18832	159.473	-0.12616
158.178	-0.14572	160.92	-0.19736	160.473	-0.12245
159.178	-0.14547	161.92	-0.19775	161.473	-0.18362
160.178	-0.15119	162.92	-0.197	162.473	-0.15889
161.178	-0.15865	163.92	-0.19962	163.473	-0.15921
162.178	-0.15659	164.92	-0.20551	164.473	-0.16435
163.178	-0.15335	165.92	-0.20068	165.473	-0.15122
164.178	-0.15312	166.92	-0.19236	166.473	-0.13118
165.178	-0.15399	167.92	-0.18486	167.473	-0.12145
166.178	-0.14227	168.92	-0.17815	168.473	-0.15297
167.178	-0.13073	169.92	-0.17638	169.473	-0.10991
168.178	-0.11898	170.92	-0.1759	170.473	-0.09121197
169.178	-0.10918	171.92	-0.1769	171.473	-0.10229
170.178	-0.10275	172.92	-0.1742	172.473	-0.10707
171.178	-0.09899002	173.92	-0.17059	173.473	-0.12494
172.178	-0.09931853	174.92	-0.17196	174.473	-0.12269
173.178	-0.09631755	175.92	-0.17343	175.473	-0.14214
174.178	-0.09562745	176.92	-0.17403	176.473	-0.12708
175.178	-0.09533729	177.92	-0.17047	177.473	-0.12193
176.178	-0.09480838	178.92	-0.16629	178.473	-0.1075
177.178	-0.09363537	179.92	-0.1599	179.473	-0.10707
178.178	-0.09079301	180.92	-0.15477	180.473	-0.10605
179.178	-0.09022864	181.92	-0.15349	181.473	-0.088634
180.178	-0.08518101	182.92	-0.15019	182.473	-0.09880078
181.178	-0.080355	183.92	-0.14861	183.473	-0.08383444
182.178	-0.07916926	184.92	-0.15088	184.473	-0.06867764
183.178	-0.07970914	185.92	-0.14723	185.473	-0.10067
184.178	-0.0835241	186.92	-0.1429	186.473	-0.09342027
185.178	-0.08798537	187.92	-0.14263	187.473	-0.09763539
186.178	-0.08532579	188.92	-0.14593	188.473	-0.12862
187.178	-0.08008238	189.92	-0.14472	189.473	-0.10783
188.178	-0.07893474	190.92	-0.14732	190.473	-0.08399611
189.178	-0.08043961	191.92	-0.15094	191.473	-0.09536896
190.178	-0.0768852	192.92	-0.14629	192.473	-0.12285

191.178	-0.07562505	193.92	-0.14794	193.473	-0.10676
192.178	-0.07164838	194.92	-0.15598	194.473	-0.11374
193.178	-0.06231934	195.92	-0.16156	195.473	-0.12713
194.178	-0.06228346	196.92	-0.16782	196.473	-0.10512
195.178	-0.06451878	197.92	-0.17065	197.473	-0.10222
196.178	-0.06550282	198.92	-0.1707	198.473	-0.12182
197.178	-0.06544845	199.92	-0.16827	199.473	-0.12092
198.178	-0.06325512	200.92	-0.17339	200.473	-0.14255
199.178	-0.05811954	201.92	-0.17688	201.473	-0.0912094
200.178	-0.05448824	202.92	-0.17548	202.473	-0.0733918
201.178	-0.05750108	203.92	-0.17353	203.473	-0.08139227
202.178	-0.05475808	204.92	-0.16465	204.473	-0.09446999
203.178	-0.05220468	205.92	-0.16523	205.473	-0.09485616
204.178	-0.04781205	206.92	-0.17048	206.473	-0.08179048
205.178	-0.03853694	207.92	-0.17688	207.473	-0.08970851
206.178	-0.03899931	208.92	-0.17502	208.473	-0.08599348
207.178	-0.04395234	209.92	-0.17031	209.473	-0.11902
208.178	-0.04689308	210.92	-0.17344	210.473	-0.12116
209.178	-0.04352488	211.92	-0.17602	211.473	-0.12377
210.178	-0.04141273	212.92	-0.18201	212.473	-0.08774347
211.178	-0.04201384	213.92	-0.18007	213.473	-0.07331235
212.178	-0.04314813	214.92	-0.17503	214.473	-0.10676
213.178	-0.0458419	215.92	-0.17053	215.473	-0.09537887
214.178	-0.04250275	216.92	-0.17374	216.473	-0.08041804
215.178	-0.03622613	217.92	-0.17622	217.473	-0.05911543
216.178	-0.03309204	218.92	-0.1723	218.473	-0.07498694
217.178	-0.03515936	219.92	-0.16665	219.473	-0.07824182
218.178	-0.03632796	220.92	-0.16067	220.473	-0.12502
219.178	-0.03405994	221.92	-0.15785	221.473	-0.1017
220.178	-0.03210378	222.92	-0.15311	222.473	-0.1134
221.178	-0.03132709	223.92	-0.15154	223.473	-0.09639406
222.178	-0.03405319	224.92	-0.14231	224.473	-0.09430231
223.178	-0.03601992	225.92	-0.13209	225.473	-0.09498253
224.178	-0.04166433	226.92	-0.12594	226.473	-0.08206488
225.178	-0.03895289	227.92	-0.12127	227.473	-0.11827
226.178	-0.03839455	228.92	-0.11362	228.473	-0.1057
227.178	-0.04251488	229.92	-0.10736	229.473	-0.06055792
228.178	-0.04716727	230.92	-0.10411	230.473	-0.10115
229.178	-0.05074938	231.92	-0.09688375	231.473	-0.11784
230.178	-0.05485661	232.92	-0.09045425	232.473	-0.12265
231.178	-0.06088674	233.92	-0.09472694	233.473	-0.13814
232.178	-0.05924033	234.92	-0.09293871	234.473	-0.14532
233.178	-0.06071553	235.92	-0.09220335	235.473	-0.13543

234.178	-0.06857471	236.92	-0.0935689	236.473	-0.09638667
235.178	-0.06849355	237.92	-0.09643254	237.473	-0.17646
236.178	-0.06914705	238.92	-0.09621096	238.473	-0.13478
237.178	-0.07130665	239.92	-0.09322216	239.473	-0.13792
238.178	-0.07378496	240.92	-0.09010231	240.473	-0.11647
239.178	-0.07435141	241.92	-0.0829295	241.473	-0.14868
240.178	-0.07588719	242.92	-0.08000668	242.473	-0.13857
241.178	-0.08052107	243.92	-0.07426794	243.473	-0.12341
242.178	-0.07923983	244.92	-0.07523621	244.473	-0.11139
243.178	-0.08064719	245.92	-0.07236374	245.473	-0.11318
244.178	-0.08074597	246.92	-0.0696053	246.473	-0.11912
245.178	-0.0852272	247.92	-0.07293182	247.473	-0.11939
246.178	-0.08414665	248.92	-0.08228808	248.473	-0.16971
247.178	-0.08448443	249.92	-0.08971668	249.473	-0.14545
248.178	-0.08750999	250.92	-0.09901431	250.473	-0.135
249.178	-0.09112307	251.92	-0.11264	251.473	-0.159
250.178	-0.09556826	252.92	-0.11668	252.473	-0.17715
251.178	-0.10639	253.92	-0.11941	253.473	-0.17266
252.178	-0.11862	254.92	-0.12691	254.473	-0.20774
253.178	-0.12234	255.92	-0.13661	255.473	-0.21468
254.178	-0.12795	256.92	-0.1436	256.473	-0.18281
255.178	-0.13755	257.92	-0.14542	257.473	-0.16065
256.178	-0.15119	258.92	-0.13944	258.473	-0.18825
257.178	-0.16271	259.92	-0.13491	259.473	-0.23747
258.178	-0.17068	260.92	-0.13955	260.473	-0.25187
259.178	-0.16933	261.92	-0.14729	261.473	-0.2146
260.178	-0.17093	262.92	-0.15069	262.473	-0.16021
261.178	-0.17905	263.92	-0.14854	263.473	-0.19055
262.178	-0.1902	264.92	-0.14226	264.473	-0.25527
263.178	-0.19799	265.92	-0.14549	265.473	-0.26656
264.178	-0.19841	266.92	-0.16108	266.473	-0.25993
265.178	-0.19657	267.92	-0.17268	267.473	-0.24985
266.178	-0.20026	268.92	-0.17659	268.473	-0.21688
267.178	-0.21497	269.92	-0.18051	269.473	-0.2666
268.178	-0.22357	270.92	-0.18678	270.473	-0.34444
269.178	-0.22836	271.92	-0.19435	271.473	-0.32576
270.178	-0.23435	272.92	-0.20849	272.473	-0.29945
271.178	-0.23862	273.92	-0.22161	273.473	-0.30738
272.178	-0.24501	274.92	-0.22551	274.473	-0.3058
273.178	-0.26081	275.92	-0.22978	275.473	-0.31127
274.178	-0.27795	276.92	-0.23637	276.473	-0.35302
275.178	-0.28736	277.92	-0.24269	277.473	-0.37287
276.178	-0.29909	278.92	-0.2496	278.473	-0.36014

277.178	-0.31398	279.92	-0.25712	279.473	-0.36968
278.178	-0.32565	280.92	-0.26844	280.473	-0.36913
279.178	-0.33882	281.92	-0.27489	281.473	-0.37877
280.178	-0.35228	282.92	-0.28228	282.473	-0.38873
281.178	-0.36377	283.92	-0.28995	283.473	-0.37434
282.178	-0.36898	284.92	-0.29877	284.473	-0.43613
283.178	-0.37409	285.92	-0.30685	285.473	-0.42268
284.178	-0.37972	286.92	-0.31983	286.473	-0.40651
285.178	-0.38371	287.92	-0.33149	287.473	-0.40143
286.178	-0.38877	288.92	-0.3295	288.473	-0.42693
287.178	-0.40212	289.92	-0.33654	289.473	-0.41438
288.178	-0.41245	290.92	-0.35026	290.473	-0.47014
289.178	-0.41443	291.92	-0.35657	291.473	-0.47473
290.178	-0.42746	292.92	-0.36447	292.473	-0.3998
291.178	-0.44464	293.92	-0.38147	293.473	-0.46959
292.178	-0.45458	294.92	-0.39183	294.473	-0.54132
293.178	-0.46899	295.92	-0.40473	295.473	-0.47476
294.178	-0.48766	296.92	-0.42663	296.473	-0.47402
295.178	-0.49727	297.92	-0.43772	297.473	-0.54389
296.178	-0.51327	298.92	-0.43957	298.473	-0.5387
297.178	-0.53401	299.92	-0.46409	299.473	-0.55169
298.178	-0.54239	300.92	-0.48669	300.473	-0.58654
299.178	-0.54808	301.92	-0.49363	301.473	-0.55208
300.178	-0.57402	302.92	-0.50307	302.473	-0.49627
301.178	-0.59223	303.92	-0.51039	303.473	-0.62767
302.178	-0.59923	304.92	-0.51651	304.473	-0.62708
303.178	-0.61059	305.92	-0.52803	305.473	-0.56642
304.178	-0.61672	306.92	-0.54389	306.473	-0.59926
305.178	-0.62321	307.92	-0.54296	307.473	-0.59148
306.178	-0.63615	308.92	-0.53944	308.473	-0.60052
307.178	-0.65011	309.92	-0.54907	309.473	-0.61851
308.178	-0.64787	310.92	-0.55813	310.473	-0.65374
309.178	-0.65022	311.92	-0.57073	311.473	-0.64362
310.178	-0.6639	312.92	-0.58536	312.473	-0.63697
311.178	-0.67454	313.92	-0.60095	313.473	-0.68316
312.178	-0.69073	314.92	-0.61708	314.473	-0.68115
313.178	-0.70576	315.92	-0.63731	315.473	-0.71966
314.178	-0.71914	316.92	-0.65895	316.473	-0.74484
315.178	-0.73273	317.92	-0.67832	317.473	-0.75281
316.178	-0.75095	318.92	-0.69616	318.473	-0.7604
317.178	-0.7719	319.92	-0.71074	319.473	-0.76656
318.178	-0.79003	320.92	-0.72304	320.473	-0.77943
319.178	-0.80721	321.92	-0.73368	321.473	-0.81163

320.178	-0.82175	322.92	-0.74162	322.473	-0.81344
321.178	-0.83763	323.92	-0.74953	323.473	-0.82743
322.178	-0.85372	324.92	-0.7605	324.473	-0.82725
323.178	-0.86666	325.92	-0.76731	325.473	-0.83395
324.178	-0.87915	326.92	-0.77674	326.473	-0.83994
325.178	-0.8887	327.92	-0.78952	327.473	-0.85258
326.178	-0.89487	328.92	-0.8036	328.473	-0.88263
327.178	-0.90385	329.92	-0.81901	329.473	-0.88365
328.178	-0.91293	330.92	-0.83431	330.473	-0.91802
329.178	-0.92281	331.92	-0.84924	331.473	-0.92392
330.178	-0.93289	332.92	-0.86242	332.473	-0.94381
331.178	-0.94483	333.92	-0.87927	333.473	-0.96824
332.178	-0.9583	334.92	-0.89543	334.473	-0.95464
333.178	-0.97283	335.92	-0.90814	335.473	-0.96657
334.178	-0.98934	336.92	-0.91839	336.473	-0.99093
335.178	-1.00529	337.92	-0.92601	337.473	-1.00009
336.178	-1.02159	338.92	-0.93858	338.473	-0.99471
337.178	-1.03447	339.92	-0.95315	339.473	-1.01213
338.178	-1.04676	340.92	-0.96598	340.473	-1.01652
339.178	-1.06432	341.92	-0.97795	341.473	-1.00367
340.178	-1.0789	342.92	-0.99047	342.473	-1.0514
341.178	-1.09201	343.92	-1.00527	343.473	-1.06754
342.178	-1.10701	344.92	-1.02313	344.473	-1.06483
343.178	-1.12321	345.92	-1.04336	345.473	-1.08758
344.178	-1.13942	346.92	-1.06139	346.473	-1.11514
345.178	-1.15919	347.92	-1.07851	347.473	-1.14313
346.178	-1.18231	348.92	-1.09511	348.473	-1.16692
347.178	-1.20441	349.92	-1.10974	349.473	-1.19453
348.178	-1.22624	350.92	-1.12287	350.473	-1.23408
349.178	-1.24824	351.92	-1.13778	351.473	-1.23984
350.178	-1.26962	352.92	-1.15296	352.473	-1.24139
351.178	-1.28957	353.92	-1.17181	353.473	-1.25371
352.178	-1.31189	354.92	-1.18974	354.473	-1.2811
353.178	-1.3371	355.92	-1.20482	355.473	-1.29736
354.178	-1.362	356.92	-1.22043	356.473	-1.32134
355.178	-1.38033	357.92	-1.23849	357.473	-1.35959
356.178	-1.40088	358.92	-1.25916	358.473	-1.35481
357.178	-1.42629	359.92	-1.27923	359.473	-1.37812
358.178	-1.45255	360.92	-1.29936	360.473	-1.38804
359.178	-1.48079	361.92	-1.31387	361.473	-1.4258
360.178	-1.50945	362.92	-1.32912	362.473	-1.43774
361.178	-1.53462	363.92	-1.34996	363.473	-1.47014
362.178	-1.55885	364.92	-1.37768	364.473	-1.5198

363.178	-1.5939	365.92	-1.40455	365.473	-1.54126
364.178	-1.63254	366.92	-1.42912	366.473	-1.587
365.178	-1.67054	367.92	-1.4512	367.473	-1.62713
366.178	-1.712	368.92	-1.47223	368.473	-1.67762
367.178	-1.75142	369.92	-1.49598	369.473	-1.70766
368.178	-1.78984	370.92	-1.51983	370.473	-1.72984
369.178	-1.83179	371.92	-1.53893	371.473	-1.74162
370.178	-1.87675	372.92	-1.55398	372.473	-1.7712
371.178	-1.91903	373.92	-1.57003	373.473	-1.79761
372.178	-1.96086	374.92	-1.59102	374.473	-1.83308
373.178	-2.00488	375.92	-1.61217	375.473	-1.87638
374.178	-2.04924	376.92	-1.6334	376.473	-1.93576
375.178	-2.10343	377.92	-1.65797	377.473	-1.97726
376.178	-2.1642	378.92	-1.68457	378.473	-2.03361
377.178	-2.228	379.92	-1.71163	379.473	-2.08566
378.178	-2.29673	380.92	-1.73631	380.473	-2.13636
379.178	-2.37254	381.92	-1.76407	381.473	-2.21794
380.178	-2.45073	382.92	-1.79356	382.473	-2.29189
381.178	-2.52863	383.92	-1.8217	383.473	-2.29134
382.178	-2.61759	384.92	-1.84662	384.473	-2.38445
383.178	-2.70826	385.92	-1.86703	385.473	-2.45718
384.178	-2.79604	386.92	-1.88466	386.473	-2.58114
385.178	-2.88396	387.92	-1.91315	387.473	-2.6604
386.178	-2.97103	388.92	-1.94714	388.473	-2.70791
387.178	-3.05787	389.92	-1.97194	389.473	-2.74702
388.178	-3.15338	390.92	-1.98551	390.473	-2.81433
389.178	-3.26461	391.92	-2.00744	391.473	-2.99175
390.178	-3.36486	392.92	-2.04143	392.473	-3.09155
391.178	-3.45774	393.92	-2.07914	393.473	-3.16061
392.178	-3.56114	394.92	-2.11724	394.473	-3.16959
393.178	-3.68076	395.92	-2.1434	395.473	-3.24517
394.178	-3.80724	396.92	-2.16537	396.473	-3.42934
395.178	-3.94281	397.92	-2.19423	397.473	-3.54423
396.178	-4.07904	398.92	-2.23344	398.473	-3.6795
397.178	-4.21278	399.92	-2.2687	399.473	-3.72401
398.178	-4.36057	400.92	-2.29307	400.473	-3.81551
399.178	-4.5276	401.92	-2.31881	401.473	-3.91453
400.178	-4.71399	402.92	-2.34373	402.473	-4.05021
401.178	-4.90159	403.92	-2.37137	403.473	-4.17805
402.178	-5.09864	404.92	-2.40578	404.473	-4.26256
403.178	-5.31359	405.92	-2.44837	405.473	-4.41013
404.178	-5.53557	406.92	-2.49537	406.473	-4.56123
405.178	-5.76884	407.92	-2.54481	407.473	-4.67274

406.178	-6.00554	408.92	-2.59301	408.473	-4.8188
407.178	-6.25347	409.92	-2.64458	409.473	-5.00538
408.178	-6.49105	410.92	-2.7043	410.473	-5.23976
409.178	-6.71316	411.92	-2.77638	411.473	-5.39075
410.178	-6.91783	412.92	-2.84132	412.473	-5.5612
411.178	-7.09318	413.92	-2.90772	413.473	-5.68167
412.178	-7.23823	414.92	-2.96586	414.473	-5.81617
413.178	-7.35824	415.92	-3.02919	415.473	-6.01421
414.178	-7.44283	416.92	-3.11046	416.473	-6.17386
415.178	-7.4788	417.92	-3.19716	417.473	-6.29948
416.178	-7.46156	418.92	-3.29073	418.473	-6.39424
417.178	-7.39366	419.92	-3.38486	419.473	-6.45702
418.178	-7.27758	420.92	-3.48645	420.473	-6.45492
419.178	-7.13142	421.92	-3.58638	421.473	-6.50744
420.178	-6.9432	422.92	-3.69268	422.473	-6.50042
421.178	-6.70362	423.92	-3.81502	423.473	-6.43139
422.178	-6.42891	424.92	-3.93856	424.473	-6.33528
423.178	-6.12501	425.92	-4.06166	425.473	-6.11628
424.178	-5.81623	426.92	-4.18601	426.473	-5.90326
425.178	-5.5051	427.92	-4.31508	427.473	-5.6805
426.178	-5.19149	428.92	-4.44766	428.473	-5.49819
427.178	-4.88256	429.92	-4.58987	429.473	-5.26997
428.178	-4.59122	430.92	-4.73767	430.473	-4.99315
429.178	-4.33068	431.92	-4.88399	431.473	-4.79226
430.178	-4.09111	432.92	-5.01502	432.473	-4.59229
431.178	-3.87734	433.92	-5.13402	433.473	-4.39807
432.178	-3.66559	434.92	-5.24614	434.473	-4.22805
433.178	-3.46014	435.92	-5.33394	435.473	-4.0779
434.178	-3.26666	436.92	-5.40104	436.473	-3.80743
435.178	-3.07816	437.92	-5.44868	437.473	-3.69074
436.178	-2.9003	438.92	-5.47374	438.473	-3.44817
437.178	-2.72944	439.92	-5.46863	439.473	-3.32443
438.178	-2.55781	440.92	-5.43839	440.473	-3.22595
439.178	-2.3874	441.92	-5.39029	441.473	-3.09474
440.178	-2.23391	442.92	-5.31972	442.473	-2.91698
441.178	-2.07028	443.92	-5.22841	443.473	-2.81479
442.178	-1.9148	444.92	-5.12358	444.473	-2.74684
443.178	-1.75933	445.92	-4.98979	445.473	-2.59262
444.178	-1.60341	446.92	-4.83797	446.473	-2.49901
445.178	-1.45396	447.92	-4.66847	447.473	-2.35675
446.178	-1.30962	448.92	-4.47911	448.473	-2.24213
447.178	-1.17116	449.92	-4.26937	449.473	-2.0468
448.178	-1.03983	450.92	-4.05079	450.473	-1.92955

449.178	-0.92634	451.92	-3.8119	451.473	-1.73251
450.178	-0.81449	452.92	-3.56789	452.473	-1.59288
451.178	-0.71715	453.92	-3.31964	453.473	-1.4222
452.178	-0.62949	454.92	-3.07649	454.473	-1.23443
453.178	-0.55513	455.92	-2.84121	455.473	-1.08976
454.178	-0.49221	456.92	-2.61467	456.473	-0.96889
455.178	-0.43925	457.92	-2.39387	457.473	-0.8842
456.178	-0.39157	458.92	-2.18304	458.473	-0.76785
457.178	-0.34956	459.92	-1.99242	459.473	-0.65917
458.178	-0.32028	460.92	-1.80762	460.473	-0.56981
459.178	-0.29981	461.92	-1.63042	461.473	-0.5002
460.178	-0.28274	462.92	-1.4617	462.473	-0.46675
461.178	-0.26456	463.92	-1.31283	463.473	-0.40517
462.178	-0.24984	464.92	-1.17843	464.473	-0.33881
463.178	-0.24033	465.92	-1.05873	465.473	-0.30522
464.178	-0.23743	466.92	-0.94547	466.473	-0.28336
465.178	-0.23759	467.92	-0.84526	467.473	-0.28095
466.178	-0.23855	468.92	-0.7588	468.473	-0.28812
467.178	-0.23431	469.92	-0.68776	469.473	-0.29341
468.178	-0.23004	470.92	-0.62076	470.473	-0.25401
469.178	-0.2281	471.92	-0.55916	471.473	-0.24001
470.178	-0.22878	472.92	-0.50473	472.473	-0.22472
471.178	-0.22857	473.92	-0.45721	473.473	-0.21022
472.178	-0.22791	474.92	-0.42217	474.473	-0.2088
473.178	-0.22396	475.92	-0.39508	475.473	-0.21767
474.178	-0.21588	476.92	-0.37593	476.473	-0.20336
475.178	-0.21137	477.92	-0.36323	477.473	-0.17706
476.178	-0.20871	478.92	-0.3561	478.473	-0.18508
477.178	-0.21194	479.92	-0.35062	479.473	-0.1939
478.178	-0.2168	480.92	-0.34897	480.473	-0.21528
479.178	-0.21914	481.92	-0.35419	481.473	-0.23109
480.178	-0.21553	482.92	-0.3611	482.473	-0.22738
481.178	-0.21449	483.92	-0.36791	483.473	-0.19892
482.178	-0.22058	484.92	-0.37185	484.473	-0.18012
483.178	-0.22865	485.92	-0.37252	485.473	-0.21287
484.178	-0.23666	486.92	-0.37398	486.473	-0.23774
485.178	-0.23691	487.92	-0.38046	487.473	-0.22923
486.178	-0.22958	488.92	-0.39094	488.473	-0.20999
487.178	-0.22387	489.92	-0.39739	489.473	-0.18722
488.178	-0.22295	490.92	-0.40233	490.473	-0.18876
489.178	-0.22528	491.92	-0.40906	491.473	-0.20598
490.178	-0.22301	492.92	-0.42078	492.473	-0.21693
491.178	-0.21575	493.92	-0.43289	493.473	-0.20982

492.178	-0.20657	494.92	-0.44848	494.473	-0.19404
493.178	-0.20503	495.92	-0.46316	495.473	-0.19108
494.178	-0.20499	496.92	-0.47583	496.473	-0.22372
495.178	-0.20644	497.92	-0.49064	497.473	-0.20907
496.178	-0.20842	498.92	-0.50315	498.473	-0.22057
497.178	-0.20748	499.92	-0.51201	499.473	-0.24068
498.178	-0.20429	500.92	-0.51915	500.473	-0.25086
499.178	-0.19902	501.92	-0.53147	501.473	-0.25143
500.178	-0.19538	502.92	-0.54369	502.473	-0.23686
501.178	-0.18937	503.92	-0.55206	503.473	-0.21514
502.178	-0.19073	504.92	-0.55523	504.473	-0.22067
503.178	-0.19073	505.92	-0.55641	505.473	-0.26344
504.178	-0.18719	506.92	-0.56156	506.473	-0.2734
505.178	-0.18068	507.92	-0.56756	507.473	-0.24773
506.178	-0.17885	508.92	-0.56782	508.473	-0.21105
507.178	-0.18474	509.92	-0.56128	509.473	-0.21767
508.178	-0.18985	510.92	-0.54883	510.473	-0.23738
509.178	-0.19183	511.92	-0.53235	511.473	-0.21969
510.178	-0.18893	512.92	-0.51966	512.473	-0.18234
511.178	-0.18405	513.92	-0.5036	513.473	-0.18798
512.178	-0.17776	514.92	-0.48524	514.473	-0.19251
513.178	-0.17686	515.92	-0.4679	515.473	-0.15901
514.178	-0.17149	516.92	-0.45155	516.473	-0.1812
515.178	-0.16594	517.92	-0.42888	517.473	-0.16527
516.178	-0.16411	518.92	-0.40625	518.473	-0.182
517.178	-0.16176	519.92	-0.38853	519.473	-0.2189
518.178	-0.15289	520.92	-0.37077	520.473	-0.21497
519.178	-0.14548	521.92	-0.35188	521.473	-0.15034
520.178	-0.14571	522.92	-0.32699	522.473	-0.13654
521.178	-0.14578	523.92	-0.29852	523.473	-0.15923
522.178	-0.14633	524.92	-0.26937	524.473	-0.16664
523.178	-0.13785	525.92	-0.25219	525.473	-0.15969
524.178	-0.12507	526.92	-0.2355	526.473	-0.1112
525.178	-0.11363	527.92	-0.21646	527.473	-0.08984933
526.178	-0.11335	528.92	-0.19728	528.473	-0.0638183
527.178	-0.11353	529.92	-0.18155	529.473	-0.10059
528.178	-0.10921	530.92	-0.17108	530.473	-0.10234
529.178	-0.10122	531.92	-0.16036	531.473	-0.0928437
530.178	-0.09416297	532.92	-0.15431	532.473	-0.09778339
531.178	-0.09478332	533.92	-0.14383	533.473	-0.09026262
532.178	-0.09589465	534.92	-0.1372	534.473	-0.08761079
533.178	-0.10094	535.92	-0.13125	535.473	-0.06753459
534.178	-0.09964302	536.92	-0.12369	536.473	-0.10851

535.178	-0.1023	537.92	-0.11536	537.473	-0.098469
536.178	-0.10112	538.92	-0.10759	538.473	-0.11286
537.178	-0.10034	539.92	-0.10112	539.473	-0.09045413
538.178	-0.0979735	540.92	-0.09490379	540.473	-0.06359394
539.178	-0.09211672	541.92	-0.08917352	541.473	-0.06457293
540.178	-0.08777089	542.92	-0.07643967	542.473	-0.0631862
541.178	-0.08115587	543.92	-0.06736511	543.473	-0.06847169
542.178	-0.07471681	544.92	-0.06132684	544.473	-0.06914018
543.178	-0.05890166	545.92	-0.05739727	545.473	-0.06146635
544.178	-0.05079094	546.92	-0.05364657	546.473	-0.009912189
545.178	-0.04504951	547.92	-0.05241163	547.473	-0.03537189
546.178	-0.04433879	548.92	-0.04560462	548.473	-0.04134097
547.178	-0.04569346	549.92	-0.04078683	549.473	-0.04847123
548.178	-0.04694749	550.92	-0.0434237	550.473	-0.04216667
549.178	-0.0430696	551.92	-0.04223512	551.473	-0.0569149
550.178	-0.04300241	552.92	-0.04025545	552.473	-0.01821231
551.178	-0.05144064	553.92	-0.0339581	553.473	-0.0380059
552.178	-0.05334268	554.92	-0.02628537	554.473	-0.0698691
553.178	-0.05517909	555.92	-0.01886198	555.473	-0.0468216
554.178	-0.04924994	556.92	-0.02081524	556.473	-0.05542061
555.178	-0.04229636	557.92	-0.02132318	557.473	-0.03348721
556.178	-0.03587232	558.92	-0.01855108	558.473	-0.02069966
557.178	-0.03779594	559.92	-0.01647922	559.473	-0.01597906
558.178	-0.0350915	560.92	-0.0154635	560.473	-0.05414943
559.178	-0.02704389	561.92	-0.01953198	561.473	-0.03387193
560.178	-0.019743	562.92	-0.02727772	562.473	-0.02293686
561.178	-0.01254302	563.92	-0.03418134	563.473	-0.008433762
562.178	-0.01197331	564.92	-0.03416194	564.473	-0.01032828
563.178	-0.01449225	565.92	-0.03235471	565.473	-0.02240795
564.178	-0.0175296	566.92	-0.03024905	566.473	-0.03668036
565.178	-0.01588527	567.92	-0.03128739	567.473	-0.04057641
566.178	-0.01568223	568.92	-0.03184475	568.473	-0.02898089
567.178	-0.01745318	569.92	-0.03022528	569.473	-0.01580535
568.178	-0.02305179	570.92	-0.02769513	570.473	-0.01794698
569.178	-0.02763796	571.92	-0.0221216	571.473	-0.03187066
570.178	-0.02981984	572.92	-0.01747417	572.473	-0.03171149
571.178	-0.03067315	573.92	-0.01599184	573.473	-0.03030731
572.178	-0.02578897	574.92	-0.01521744	574.473	-0.02729015
573.178	-0.02049179	575.92	-0.01198159	575.473	0.007115982
574.178	-0.0168929	576.92	-0.00738289	576.473	0.02133769
575.178	-0.01273186	577.92	-0.002156805	577.473	0.005019416
576.178	-0.00626584	578.92	0.002620665	578.473	0.0242371
577.178	1.37E-04	579.92	0.002136057	579.473	0.03391413

578.178	0.007110881	580.92	-0.002596468	580.473	0.04411834
579.178	0.01350447	581.92	-0.00506002	581.473	0.05526909
580.178	0.01235479	582.92	-0.008940729	582.473	0.03008476
581.178	0.008530744	583.92	-0.01504346	583.473	0.002145046
582.178	0.006183907	584.92	-0.02158945	584.473	-0.02426079
583.178	-8.94E-05	585.92	-0.02904378	585.473	-0.01302801
584.178	-0.00809199	586.92	-0.03275317	586.473	-0.03038236
585.178	-0.01492512	587.92	-0.03213528	587.473	-0.04014294
586.178	-0.02173484	588.92	-0.02722353	588.473	-0.03262253
587.178	-0.02446763	589.92	-0.02411165	589.473	-0.03168244
588.178	-0.0227695	590.92	-0.01869268	590.473	-0.009379481
589.178	-0.0195003	591.92	-0.01021811	591.473	0.007235218
590.178	-0.01846657	592.92	-0.002543457	592.473	0.01761378
591.178	-0.01348376	593.92	0.002622267	593.473	0.007212883
592.178	-0.0065829	594.92	0.004784946	594.473	0.009434496
593.178	-0.00298374	595.92	0.004284134	595.473	0.01236323
594.178	-0.0019763	596.92	0.003718053	596.473	0.005995628
595.178	-0.00541973	597.92	0.006713578	597.473	-0.00444735
596.178	-0.01061566	598.92	0.008662796	598.473	-0.01081279
597.178	-0.01405456	599.92	0.006478467	599.473	-0.01662608
598.178	-0.01339438	600.92	0.00335249	600.473	-0.01538772
599.178	-0.01388275	601.92	0.00466555	601.473	0.003758934
600.178	-0.01725516	602.92	0.007008019	602.473	0.009297511
601.178	-0.01930997	603.92	0.01225038	603.473	-9.51E-04
602.178	-0.01840305	604.92	0.01359861	604.473	-0.0158829
603.178	-0.01449114	605.92	0.006044797	605.473	-0.006930921
604.178	-0.00739486	606.92	-0.003395407	606.473	-0.02135097
605.178	-0.00554687	607.92	-0.008117482	607.473	0.01382566
606.178	-0.01050047	608.92	-0.007834467	608.473	0.009268228
607.178	-0.01443432	609.92	-0.01112121	609.473	-0.02882617
608.178	-0.0127497	610.92	-0.0147804	610.473	-0.03332653
609.178	-0.00797192	611.92	-0.02368497	611.473	-0.01079053
610.178	-0.00461731	612.92	-0.0264078	612.473	0.01962113
611.178	-0.00311066	613.92	-0.01934833	613.473	0.01773368
612.178	-0.00707509	614.92	-0.01198283	614.473	0.0273466
613.178	-0.00354406	615.92	-0.008907044	615.473	-0.01638595
614.178	0.005463953	616.92	-0.009770599	616.473	0.007008961
615.178	0.01152794	617.92	-0.009606604	617.473	0.009504315
616.178	0.01247297	618.92	-0.007518468	618.473	-0.01493291
617.178	0.01049509	619.92	3.51E-04	619.473	-0.03024439
618.178	0.008196794	620.92	3.87E-04	620.473	-0.03362436
619.178	0.007988699	621.92	-0.004659774	621.473	-0.03739448
620.178	0.01250755	622.92	-0.006093209	622.473	-0.03387098

621.178	0.007822239	623.92	-0.006340725	623.473	0.004942607
622.178	0.003089925	624.92	-0.005618873	624.473	-0.03167054
623.178	0.002170711	625.92	-0.003145895	625.473	-0.02192649
624.178	0.001691233	626.92	-0.00240537	626.473	0.004317063
625.178	0.002294771	627.92	-0.008530515	627.473	-0.00187836
626.178	0.005097162	628.92	-0.01051241	628.473	0.01063181
627.178	0.004664276	629.92	-0.00960304	629.473	0.03050027
628.178	-0.00185153	630.92	-0.01048276	630.473	0.03547182
629.178	-0.00245161	631.92	-0.01007619	631.473	0.002242123
630.178	-0.00398877	632.92	-0.009845155	632.473	0.007969488
631.178	-0.00708561	633.92	-0.01174476	633.473	0.004427382
632.178	-0.00749192	634.92	-0.01164126	634.473	0.002122827
633.178	-0.00914766	635.92	-0.00800989	635.473	0.005991143
634.178	-0.01215284	636.92	-0.005267075	636.473	-0.007696623
635.178	-0.01148288	637.92	-0.00163285	637.473	-0.01640978
636.178	-0.00798497	638.92	3.15E-04	638.473	-0.009830968
637.178	-0.00548225	639.92	1.03E-04	639.473	-0.01255686
638.178	-0.00208385	640.92	0.003110615	640.473	-0.01296639
639.178	8.76E-04	641.92	0.003184927	641.473	0.008801208
640.178	0.004159913	642.92	-0.002213724	642.473	0.02230793
641.178	0.01010647	643.92	-0.006143217	643.473	0.03092946
642.178	0.009783951	644.92	-0.006778357	644.473	0.05304003
643.178	0.003803835	645.92	-0.01011341	645.473	0.01335168
644.178	0.001273586	646.92	-0.009625498	646.473	-0.03152301
645.178	0.001092935	647.92	-0.007106885	647.473	-0.02277226
646.178	0.001903836	648.92	-0.01016724	648.473	-0.002796379
647.178	0.00435176	649.92	-0.007795641	649.473	-0.002190407
648.178	0.002447423	650.92	-6.58E-04	650.473	0.02278825
649.178	-0.00288715	651.92	0.001235323	651.473	0.001222517
650.178	0.001937545	652.92	-0.00166358	652.473	-0.001232997
651.178	0.01162494	653.92	-0.00114445	653.473	0.03321674
652.178	0.01544137	654.92	-0.004313971	654.473	0.06024702
653.178	0.01581379	655.92	-0.007049224	655.473	0.03582556
654.178	0.01623519	656.92	-0.009289348	656.473	0.009285925
655.178	0.01390291	657.92	-0.01659198	657.473	0.02501705
656.178	0.01516224	658.92	-0.02330239	658.473	0.003041958
657.178	0.01413988	659.92	-0.02089126	659.473	0.01488153
658.178	0.005925576	660.92	-0.01296914	660.473	-0.004839868
659.178	-0.00196951	661.92	-0.006805671	661.473	-0.03392298
660.178	-0.00365904	662.92	-0.001261057	662.473	-0.02505201
661.178	-0.00193567	663.92	4.99E-04	663.473	0.005859465
662.178	-0.00312666	664.92	0.002118325	664.473	0.0246155
663.178	-0.00526532	665.92	0.006276986	665.473	0.03787737

664.178	-0.00936019	666.92	0.008352196	666.473	0.02319292
665.178	-0.01038969	667.92	0.003598737	667.473	0.00163234
666.178	-0.00747633	668.92	-0.005819618	668.473	-0.01662259
667.178	-0.00551797	669.92	-0.01949948	669.473	-0.02075318
668.178	-0.00709154	670.92	-0.03114682	670.473	-0.02349557
669.178	-0.01299215	671.92	-0.03558487	671.473	-0.02680763
670.178	-0.02107423	672.92	-0.03514554	672.473	-0.0356294
671.178	-0.02567811	673.92	-0.03364073	673.473	-0.06488845
672.178	-0.02615227	674.92	-0.03238094	674.473	-0.0653462
673.178	-0.02516436	675.92	-0.03014544	675.473	-0.03513069
674.178	-0.02461968	676.92	-0.02629537	676.473	-0.005246619
675.178	-0.02502889	677.92	-0.01874649	677.473	0.006204212
676.178	-0.02395061	678.92	-0.01061023	678.473	7.53E-04
677.178	-0.02006142	679.92	-0.007533607	679.473	0.00685412
678.178	-0.01142621	680.92	-0.01075348	680.473	0.003914565
679.178	-0.00278028	681.92	-0.01611321	681.473	-0.006825158
680.178	0.002993006	682.92	-0.01893477	682.473	0.003115838
681.178	0.004321916	683.92	-0.01846073	683.473	0.002321737
682.178	0.004239548	684.92	-0.01673308	684.473	-0.01726524
683.178	0.006909356	685.92	-0.01599583	685.473	-0.03444963
684.178	0.009933705	686.92	-0.01793665	686.473	-0.01882261
685.178	0.01284151	687.92	-0.02002276	687.473	0.003129604
686.178	0.01330727	688.92	-0.01696146	688.473	0.014112
687.178	0.01004107	689.92	-0.009639644	689.473	0.02577324
688.178	0.005586454	690.92	-0.003826494	690.473	0.01562712
689.178	0.005709377	691.92	-0.001885385	691.473	-6.53E-06
690.178	0.008278405	692.92	-0.001822177	692.473	0.001227488
691.178	0.008070627	693.92	-8.14E-04	693.473	0.02439641
692.178	0.005378399	694.92	0.00374738	694.473	0.02602217
693.178	0.00174826	695.92	0.009324089	695.473	0.002859244
694.178	9.91E-04	696.92	0.01328572	696.473	-0.006192906
695.178	0.004631075	697.92	0.01233673	697.473	-0.003621032
696.178	0.009743966	698.92	0.009842166	698.473	0.01533155
697.178	0.01316033	699.92	0.009452852	699.473	0.02340589
698.178	0.01324015	700.92	0.0116359	700.473	0.0350206
699.178	0.0134769	701.92	0.01182761	701.473	0.01174938
700.178	0.01645481	702.92	0.006418027	702.473	-0.009366922
701.178	0.02011905	703.92	4.72E-04	703.473	-0.008058963
702.178	0.01918282	704.92	-0.007452583	704.473	0.009288582
703.178	0.01251828	705.92	-0.01259939	705.473	0.006772468
704.178	0.004754315	706.92	-0.0116176	706.473	-0.008356733
705.178	-0.00443187	707.92	-0.01185119	707.473	-0.00665231
706.178	-0.00786003	708.92	-0.01835843	708.473	-0.0191875

707.178	-0.00663893	709.92	-0.02333547	709.473	-0.009000905
708.178	-0.00941859	710.92	-0.02283803	710.473	0.03490434
709.178	-0.01574463	711.92	-0.02144175	711.473	0.03782632
710.178	-0.018701	712.92	-0.01760424	712.473	0.002072889
711.178	-0.01696877	713.92	-0.01321516	713.473	-0.001730508
712.178	-0.01415222	714.92	-0.01433218	714.473	-0.006237733
713.178	-0.00905488	715.92	-0.01544665	715.473	-0.007270346
714.178	-0.00832286	716.92	-0.01133943	716.473	0.00614563
715.178	-0.01271226	717.92	-0.007049444	717.473	0.01649692
716.178	-0.01299728	718.92	-0.005978076	718.473	-0.003181793
717.178	-0.00898477	719.92	-0.005113959	719.473	0.001358413
718.178	-0.00656714	720.92	-0.0042044	720.473	0.004288938
719.178	-0.00712677	721.92	-0.005378875	721.473	-0.003394745
720.178	-0.00606902	722.92	-0.006936147	722.473	-0.02297501
721.178	-0.00430993	723.92	-0.009560058	723.473	-0.01651336
722.178	-0.00193915	724.92	-0.01302645	724.473	-0.00385462
723.178	-4.48E-04	725.92	-0.01570775	725.473	-0.002935998
724.178	-0.00214625	726.92	-0.01371248	726.473	-0.006794186
725.178	-0.00371268	727.92	-0.0112458	727.473	-0.02595917
726.178	-0.0015162	728.92	-0.01262567	728.473	-0.02121376
727.178	0.006518973	729.92	-0.01290939	729.473	-0.003664656
728.178	0.01115117	730.92	-0.01176838	730.473	0.03161108
729.178	0.01074064	731.92	-0.008831524	731.473	0.03290372
730.178	0.009671532	732.92	-0.004824269	732.473	-0.004014817
731.178	0.01038594	733.92	-0.002109355	733.473	-0.009297504
732.178	0.01211499	734.92	-0.002582174	734.473	-0.009297521
733.178	0.01255708	735.92	-0.004629011	735.473	-0.004914555
734.178	0.009713169	736.92	-0.002318335	736.473	0.00329379
735.178	0.003294457	737.92	0.00138082	737.473	3.71E-04
736.178	-6.08E-04	738.92	0.004277836	738.473	0.002308615
737.178	0.001038016	739.92	0.005512661	739.473	2.08E-04
738.178	0.004602307	740.92	0.005384543	740.473	0.01588455
739.178	0.008013103	741.92	0.005112066	741.473	0.02169414
740.178	0.0113709	742.92	0.002802975	742.473	0.02150184
741.178	0.01495746	743.92	-3.79E-04	743.473	0.01057448
742.178	0.0186757	744.92	-0.005213557	744.473	0.007786667
743.178	0.01936622	745.92	-0.01046779	745.473	0.005075486
744.178	0.01648894	746.92	-0.01383741	746.473	-0.01281692
745.178	0.01138578	747.92	-0.01466216	747.473	-0.02169056
746.178	0.006706663	748.92	-0.01472658	748.473	-0.01986468
747.178	0.002916206	749.92	-0.01407439	749.473	8.04E-04
748.178	6.39E-04	750.92	-0.01098	750.473	0.006236934
749.178	-0.00150135	751.92	-0.004605378	751.473	0.01711357

750.178	-0.00294724	752.92	0.003247476	752.473	0.01988618
751.178	-0.00141618	753.92	0.00768434	753.473	0.02297868
752.178	0.003694092	754.92	0.00856571	754.473	0.02684783
753.178	0.009127516	755.92	0.008712913	755.473	0.03095227
754.178	0.01064433	756.92	0.009758586	756.473	0.03162396
755.178	0.01104907	757.92	0.01105231	757.473	0.01323096
756.178	0.01049198	758.92	0.01130249	758.473	0.004958677
757.178	0.009666921	759.92	0.009362237	759.473	0.004958678
758.178	0.008420913	760.92	0.005921215	760.473	0.004958735
759.178	0.00659663	761.92	0.005159943	761.473	0.004121524
760.178	0.003987519	762.92	0.009171227	762.473	0.003049936
761.178	0.001141612	763.92	0.0128198	763.473	0.004943685
762.178	4.05E-04	764.92	0.01310199	764.473	0.004250197
763.178	4.52E-04	765.92	0.01102394	765.473	0.004292525
764.178	6.41E-04	766.92	0.008774874	766.473	0.006102026
765.178	9.68E-04	767.92	0.007795758	767.473	0.007357351
766.178	0.001485411	768.92	0.007525772	768.473	0.008037826
767.178	0.00228034	769.92	0.008323331	769.473	0.009898665
768.178	0.001627882	770.92	0.008308036	770.473	0.0111297
769.178	3.29E-04	771.92	0.00481431	771.473	0.004658968
770.178	0.001398586	772.92	0.001751075	772.473	-0.004423229
771.178	0.003176207	773.92	2.51E-04	773.473	0.00286701
772.178	0.002635383	774.92	-9.05E-04	774.473	0.02498104
773.178	0.001328428	775.92	-0.003269704	775.473	0.001259628
774.178	-3.70E-04	776.92	-0.004126718	776.473	-0.00727768
775.178	-0.00376645	777.92	-0.006469057	777.473	-0.008573823
776.178	-0.0056054	778.92	-0.01580123	778.473	-0.02151061
777.178	-0.00297416	779.92	-0.0212228	779.473	-0.02794923
778.178	-0.0031383	780.92	-0.0236258	780.473	-0.005147118
779.178	-0.00623042	781.92	-0.02311785	781.473	0.003156598
780.178	-0.00708883	782.92	-0.02075938	782.473	-0.02553923
781.178	-0.00844051	783.92	-0.01644408	783.473	-0.01207328
782.178	-0.00853586	784.92	-0.01591104	784.473	-0.01562195
783.178	-0.00646592	785.92	-0.01797674	785.473	0.00209834
784.178	-0.00422104	786.92	-0.01328812	786.473	0.01446175
785.178	-0.00832793	787.92	-0.00774156	787.473	0.02294847
786.178	-0.0133282	788.92	-0.002234	788.473	0.01272947
787.178	-0.01646087	789.92	-0.001393383	789.473	-0.005267694
788.178	-0.01711304	790.92	-0.00289571	790.473	-0.004853214
789.178	-0.01351034	791.92	-0.006133046	791.473	0.001100435
790.178	-0.01273515	792.92	-0.00772703	792.473	0.01676916
791.178	-0.01082468	793.92	-0.005799907	793.473	-7.23E-04
792.178	-0.00703375	794.92	-0.008890457	794.473	-0.005807855

793.178	-0.00131298	795.92	-0.0141611	795.473	-0.00634137
794.178	0.007969071	796.92	-0.01515785	796.473	-0.006170727
795.178	0.01459468				
796.178	0.0243132				
797.178	0.01064176				

temperature	*DTG*	*temperature*	*DTG*
℃	%/min	℃	%/min
	Coal+TPPI		Coal+PA
25.93	-0.7089	25.697	-0.30683
26.93	-0.29258	26.697	-0.29172
27.93	-0.35325	27.697	-0.34575
28.93	-0.41031	28.697	-0.39796
29.93	-0.42664	29.697	-0.41883
30.93	-0.42046	30.697	-0.4107
31.93	-0.40022	31.697	-0.40174
32.93	-0.39356	32.697	-0.41267
33.93	-0.38733	33.697	-0.40666
34.93	-0.38756	34.697	-0.41132
35.93	-0.38451	35.697	-0.41347
36.93	-0.38801	36.697	-0.42143
37.93	-0.3812	37.697	-0.41474
38.93	-0.37008	38.697	-0.40328
39.93	-0.36365	39.697	-0.40381
40.93	-0.36214	40.697	-0.4061
41.93	-0.36208	41.697	-0.41139
42.93	-0.36308	42.697	-0.41442
43.93	-0.36813	43.697	-0.42153
44.93	-0.3718	44.697	-0.43244
45.93	-0.38233	45.697	-0.45058
46.93	-0.38863	46.697	-0.46248
47.93	-0.3876	47.697	-0.46459
48.93	-0.38437	48.697	-0.46591
49.93	-0.38468	49.697	-0.46933
50.93	-0.39195	50.697	-0.47756
51.93	-0.39219	51.697	-0.48117
52.93	-0.38826	52.697	-0.47707
53.93	-0.3895	53.697	-0.47387
54.93	-0.38801	54.697	-0.46854
55.93	-0.38791	55.697	-0.46835
56.93	-0.39115	56.697	-0.47377
57.93	-0.3927	57.697	-0.47901
58.93	-0.38796	58.697	-0.47543
59.93	-0.3811	59.697	-0.4703
60.93	-0.37444	60.697	-0.46408
61.93	-0.37265	61.697	-0.46267
62.93	-0.37077	62.697	-0.46196
63.93	-0.36486	63.697	-0.45837
64.93	-0.36359	64.697	-0.45769

65.93	-0.36302	65.697	-0.45576
66.93	-0.35465	66.697	-0.44799
67.93	-0.35378	67.697	-0.43839
68.93	-0.36189	68.697	-0.44634
69.93	-0.3569	69.697	-0.44498
70.93	-0.34582	70.697	-0.43358
71.93	-0.33816	71.697	-0.4246
72.93	-0.33437	72.697	-0.41936
73.93	-0.33439	73.697	-0.42012
74.93	-0.32089	74.697	-0.41314
75.93	-0.30035	75.697	-0.39263
76.93	-0.30128	76.697	-0.38853
77.93	-0.29321	77.697	-0.38938
78.93	-0.27462	78.697	-0.37032
79.93	-0.27493	79.697	-0.36383
80.93	-0.27999	80.697	-0.37041
81.93	-0.27927	81.697	-0.37079
82.93	-0.26904	82.697	-0.36322
83.93	-0.25326	83.697	-0.34719
84.93	-0.25716	84.697	-0.34179
85.93	-0.26965	85.697	-0.35538
86.93	-0.26034	86.697	-0.34851
87.93	-0.25558	87.697	-0.33556
88.93	-0.25724	88.697	-0.33268
89.93	-0.25072	89.697	-0.32553
90.93	-0.24389	90.697	-0.31648
91.93	-0.23893	91.697	-0.30959
92.93	-0.24088	92.697	-0.30644
93.93	-0.24375	93.697	-0.3047
94.93	-0.23508	94.697	-0.29572
95.93	-0.22631	95.697	-0.28217
96.93	-0.22463	96.697	-0.27842
97.93	-0.22218	97.697	-0.28021
98.93	-0.21513	98.697	-0.27536
99.93	-0.21	99.697	-0.26782
100.93	-0.20661	100.697	-0.26349
101.93	-0.20356	101.697	-0.26301
102.93	-0.20102	102.697	-0.26588
103.93	-0.19868	103.697	-0.26811
104.93	-0.19739	104.697	-0.27002
105.93	-0.19612	105.697	-0.26995
106.93	-0.193	106.697	-0.2621
107.93	-0.1952	107.697	-0.25799

108.93	-0.20026	108.697	-0.26223
109.93	-0.20262	109.697	-0.2621
110.93	-0.20184	110.697	-0.25842
111.93	-0.19735	111.697	-0.24941
112.93	-0.19549	112.697	-0.24347
113.93	-0.19133	113.697	-0.23547
114.93	-0.18984	114.697	-0.23035
115.93	-0.19242	115.697	-0.23682
116.93	-0.19193	116.697	-0.24293
117.93	-0.18535	117.697	-0.24016
118.93	-0.18026	118.697	-0.23744
119.93	-0.1788	119.697	-0.23468
120.93	-0.1833	120.697	-0.23547
121.93	-0.18764	121.697	-0.23592
122.93	-0.19443	122.697	-0.24095
123.93	-0.19537	123.697	-0.24239
124.93	-0.19303	124.697	-0.23748
125.93	-0.18913	125.697	-0.22978
126.93	-0.18956	126.697	-0.225
127.93	-0.19191	127.697	-0.22328
128.93	-0.19133	128.697	-0.22276
129.93	-0.18461	129.697	-0.21904
130.93	-0.17419	130.697	-0.2136
131.93	-0.16584	131.697	-0.20684
132.93	-0.16161	132.697	-0.20347
133.93	-0.15864	133.697	-0.19695
134.93	-0.15824	134.697	-0.19518
135.93	-0.15541	135.697	-0.19141
136.93	-0.15046	136.697	-0.1854
137.93	-0.14997	137.697	-0.1826
138.93	-0.15584	138.697	-0.18451
139.93	-0.16617	139.697	-0.18969
140.93	-0.16678	140.697	-0.18803
141.93	-0.16805	141.697	-0.18823
142.93	-0.16925	142.697	-0.19079
143.93	-0.17517	143.697	-0.19528
144.93	-0.17643	144.697	-0.19881
145.93	-0.17597	145.697	-0.20038
146.93	-0.17817	146.697	-0.20244
147.93	-0.17296	147.697	-0.19807
148.93	-0.16976	148.697	-0.19367
149.93	-0.17596	149.697	-0.19763
150.93	-0.1789	150.697	-0.19847

151.93	-0.17234	151.697	-0.19442
152.93	-0.16605	152.697	-0.18502
153.93	-0.16945	153.697	-0.18618
154.93	-0.16579	154.697	-0.1839
155.93	-0.15846	155.697	-0.17537
156.93	-0.15595	156.697	-0.17082
157.93	-0.15583	157.697	-0.17012
158.93	-0.15142	158.697	-0.16586
159.93	-0.15523	159.697	-0.16532
160.93	-0.16147	160.697	-0.17092
161.93	-0.15997	161.697	-0.17099
162.93	-0.15899	162.697	-0.16539
163.93	-0.16144	163.697	-0.16427
164.93	-0.16594	164.697	-0.1678
165.93	-0.15577	165.697	-0.1623
166.93	-0.14463	166.697	-0.15098
167.93	-0.13543	167.697	-0.14041
168.93	-0.12497	168.697	-0.12881
169.93	-0.12156	169.697	-0.121
170.93	-0.1194	170.697	-0.11671
171.93	-0.11783	171.697	-0.11642
172.93	-0.11412	172.697	-0.11417
173.93	-0.11468	173.697	-0.11034
174.93	-0.12076	174.697	-0.11136
175.93	-0.12083	175.697	-0.11105
176.93	-0.12202	176.697	-0.10982
177.93	-0.11933	177.697	-0.10718
178.93	-0.11545	178.697	-0.10441
179.93	-0.1094	179.697	-0.09927458
180.93	-0.10422	180.697	-0.09178883
181.93	-0.10327	181.697	-0.08968513
182.93	-0.09986906	182.697	-0.08889769
183.93	-0.09901621	183.697	-0.09033783
184.93	-0.10274	184.697	-0.09708625
185.93	-0.10384	185.697	-0.1007
186.93	-0.10248	186.697	-0.09831306
187.93	-0.10425	187.697	-0.09740721
188.93	-0.10756	188.697	-0.10186
189.93	-0.10598	189.697	-0.10152
190.93	-0.10605	190.697	-0.09868119
191.93	-0.10874	191.697	-0.09840795
192.93	-0.10519	192.697	-0.09057935
193.93	-0.10254	193.697	-0.08194891

194.93	-0.10419	194.697	-0.08286945
195.93	-0.10259	195.697	-0.08275442
196.93	-0.10659	196.697	-0.08347473
197.93	-0.10603	197.697	-0.08049471
198.93	-0.10058	198.697	-0.07773572
199.93	-0.09326562	199.697	-0.07094827
200.93	-0.09066309	200.697	-0.06920267
201.93	-0.08592028	201.697	-0.07197914
202.93	-0.07712566	202.697	-0.06836946
203.93	-0.07033892	203.697	-0.06619272
204.93	-0.05630934	204.697	-0.0598475
205.93	-0.04837276	205.697	-0.05822248
206.93	-0.04832674	206.697	-0.06324675
207.93	-0.05157561	207.697	-0.07349957
208.93	-0.0503427	208.697	-0.07660604
209.93	-0.04811362	209.697	-0.07246904
210.93	-0.05449027	210.697	-0.07378893
211.93	-0.05926466	211.697	-0.07365496
212.93	-0.06502419	212.697	-0.07352112
213.93	-0.06518936	213.697	-0.0682764
214.93	-0.06267872	214.697	-0.05881881
215.93	-0.05669224	215.697	-0.04516838
216.93	-0.05296316	216.697	-0.04159586
217.93	-0.05118243	217.697	-0.0411541
218.93	-0.04681294	218.697	-0.04009147
219.93	-0.04048808	219.697	-0.03661798
220.93	-0.03441134	220.697	-0.03435338
221.93	-0.03410551	221.697	-0.03650167
222.93	-0.03431605	222.697	-0.03973923
223.93	-0.04251274	223.697	-0.04682711
224.93	-0.04672144	224.697	-0.04900145
225.93	-0.04325008	225.697	-0.04479727
226.93	-0.0399186	226.697	-0.04469298
227.93	-0.04105366	227.697	-0.04608951
228.93	-0.04265788	228.697	-0.04636504
229.93	-0.04407633	229.697	-0.05028723
230.93	-0.04529044	230.697	-0.0544798
231.93	-0.0374574	231.697	-0.05546926
232.93	-0.02735393	232.697	-0.04856193
233.93	-0.03650547	233.697	-0.05750832
234.93	-0.04146149	234.697	-0.0601357
235.93	-0.04403814	235.697	-0.0616413
236.93	-0.04673895	236.697	-0.06604642

237.93	-0.05188092	237.697	-0.06498935
238.93	-0.05375691	238.697	-0.06195412
239.93	-0.05482527	239.697	-0.05929758
240.93	-0.06230427	240.697	-0.06082204
241.93	-0.06192495	241.697	-0.0560187
242.93	-0.06103021	242.697	-0.05368516
243.93	-0.06026243	243.697	-0.05378229
244.93	-0.06451854	244.697	-0.05630214
245.93	-0.06125804	245.697	-0.06059311
246.93	-0.05741266	246.697	-0.06389788
247.93	-0.06074142	247.697	-0.06950702
248.93	-0.06420742	248.697	-0.08039817
249.93	-0.06258134	249.697	-0.08773542
250.93	-0.07011779	250.697	-0.09829605
251.93	-0.08091117	251.697	-0.10938
252.93	-0.081889	252.697	-0.11347
253.93	-0.08480364	253.697	-0.11292
254.93	-0.09620813	254.697	-0.11559
255.93	-0.10805	255.697	-0.12215
256.93	-0.11576	256.697	-0.12619
257.93	-0.1239	257.697	-0.13098
258.93	-0.1193	258.697	-0.12712
259.93	-0.11378	259.697	-0.12105
260.93	-0.12029	260.697	-0.12442
261.93	-0.12881	261.697	-0.13447
262.93	-0.1318	262.697	-0.1432
263.93	-0.13035	263.697	-0.14505
264.93	-0.12826	264.697	-0.14374
265.93	-0.13016	265.697	-0.14437
266.93	-0.14618	266.697	-0.15407
267.93	-0.15884	267.697	-0.16803
268.93	-0.1626	268.697	-0.17357
269.93	-0.16631	269.697	-0.17614
270.93	-0.17373	270.697	-0.17936
271.93	-0.17934	271.697	-0.18383
272.93	-0.1906	272.697	-0.19304
273.93	-0.20382	273.697	-0.20597
274.93	-0.20887	274.697	-0.21677
275.93	-0.21615	275.697	-0.22123
276.93	-0.22259	276.697	-0.22648
277.93	-0.23011	277.697	-0.23502
278.93	-0.23459	278.697	-0.24373
279.93	-0.24113	279.697	-0.25459

280.93	-0.24946	280.697	-0.26801
281.93	-0.25561	281.697	-0.27855
282.93	-0.25938	282.697	-0.28461
283.93	-0.26223	283.697	-0.29261
284.93	-0.27053	284.697	-0.30239
285.93	-0.27641	285.697	-0.30739
286.93	-0.28818	286.697	-0.31903
287.93	-0.29823	287.697	-0.3303
288.93	-0.29819	288.697	-0.32844
289.93	-0.3067	289.697	-0.32862
290.93	-0.32306	290.697	-0.34007
291.93	-0.33035	291.697	-0.34835
292.93	-0.33568	292.697	-0.35587
293.93	-0.34875	293.697	-0.37431
294.93	-0.35632	294.697	-0.38506
295.93	-0.36847	295.697	-0.39306
296.93	-0.38884	296.697	-0.41327
297.93	-0.38981	297.697	-0.42932
298.93	-0.38377	298.697	-0.43113
299.93	-0.40628	299.697	-0.44603
300.93	-0.42952	300.697	-0.46505
301.93	-0.43553	301.697	-0.46798
302.93	-0.44229	302.697	-0.4722
303.93	-0.44667	303.697	-0.48037
304.93	-0.45046	304.697	-0.48663
305.93	-0.46636	305.697	-0.49347
306.93	-0.48865	306.697	-0.50747
307.93	-0.48612	307.697	-0.51437
308.93	-0.47944	308.697	-0.51602
309.93	-0.49207	309.697	-0.53078
310.93	-0.50581	310.697	-0.54712
311.93	-0.52152	311.697	-0.5583
312.93	-0.53697	312.697	-0.57043
313.93	-0.55224	313.697	-0.58617
314.93	-0.56112	314.697	-0.60169
315.93	-0.57576	315.697	-0.61728
316.93	-0.59294	316.697	-0.63309
317.93	-0.60374	317.697	-0.64636
318.93	-0.61283	318.697	-0.65838
319.93	-0.61967	319.697	-0.67372
320.93	-0.62613	320.697	-0.68804
321.93	-0.6289	321.697	-0.69919
322.93	-0.63511	322.697	-0.70752

323.93	-0.64582	323.697	-0.7126
324.93	-0.65933	324.697	-0.72084
325.93	-0.66829	325.697	-0.7258
326.93	-0.67765	326.697	-0.73262
327.93	-0.68587	327.697	-0.7411
328.93	-0.69411	328.697	-0.7478
329.93	-0.70733	329.697	-0.75466
330.93	-0.71832	330.697	-0.76305
331.93	-0.72408	331.697	-0.77752
332.93	-0.72965	332.697	-0.79218
333.93	-0.73984	333.697	-0.80847
334.93	-0.75185	334.697	-0.82099
335.93	-0.76708	335.697	-0.83022
336.93	-0.78232	336.697	-0.84326
337.93	-0.78865	337.697	-0.85737
338.93	-0.79355	338.697	-0.87293
339.93	-0.80527	339.697	-0.88618
340.93	-0.82122	340.697	-0.89673
341.93	-0.83364	341.697	-0.90837
342.93	-0.84277	342.697	-0.92265
343.93	-0.84827	343.697	-0.93656
344.93	-0.85162	344.697	-0.94661
345.93	-0.86641	345.697	-0.95685
346.93	-0.89123	346.697	-0.97182
347.93	-0.9076	347.697	-0.98872
348.93	-0.91126	348.697	-1.00191
349.93	-0.91709	349.697	-1.0113
350.93	-0.92622	350.697	-1.02268
351.93	-0.93885	351.697	-1.0402
352.93	-0.9573	352.697	-1.05946
353.93	-0.96955	353.697	-1.08119
354.93	-0.97202	354.697	-1.09505
355.93	-0.98379	355.697	-1.10328
356.93	-0.99905	356.697	-1.11731
357.93	-1.01097	357.697	-1.13579
358.93	-1.02704	358.697	-1.15307
359.93	-1.04283	359.697	-1.16725
360.93	-1.05913	360.697	-1.1823
361.93	-1.07539	361.697	-1.19386
362.93	-1.09021	362.697	-1.21078
363.93	-1.09587	363.697	-1.23413
364.93	-1.11548	364.697	-1.25838
365.93	-1.14772	365.697	-1.28477

366.93	-1.1681	366.697	-1.31055
367.93	-1.17667	367.697	-1.33323
368.93	-1.18161	368.697	-1.35417
369.93	-1.19359	369.697	-1.37786
370.93	-1.21661	370.697	-1.40057
371.93	-1.24504	371.697	-1.41878
372.93	-1.25819	372.697	-1.43841
373.93	-1.25917	373.697	-1.45752
374.93	-1.27317	374.697	-1.47723
375.93	-1.30304	375.697	-1.49963
376.93	-1.33347	376.697	-1.52609
377.93	-1.35783	377.697	-1.55569
378.93	-1.37857	378.697	-1.58327
379.93	-1.39412	379.697	-1.61351
380.93	-1.41407	380.697	-1.64029
381.93	-1.44086	381.697	-1.66878
382.93	-1.46797	382.697	-1.70591
383.93	-1.49001	383.697	-1.74376
384.93	-1.51115	384.697	-1.77588
385.93	-1.5303	385.697	-1.8018
386.93	-1.54375	386.697	-1.83028
387.93	-1.56918	387.697	-1.86572
388.93	-1.60034	388.697	-1.90751
389.93	-1.62208	389.697	-1.94523
390.93	-1.63045	390.697	-1.96708
391.93	-1.64686	391.697	-1.99176
392.93	-1.67219	392.697	-2.02987
393.93	-1.7013	393.697	-2.07785
394.93	-1.73579	394.697	-2.12926
395.93	-1.76118	395.697	-2.17428
396.93	-1.7836	396.697	-2.21923
397.93	-1.81179	397.697	-2.26692
398.93	-1.85664	398.697	-2.32892
399.93	-1.88927	399.697	-2.39563
400.93	-1.91519	400.697	-2.4556
401.93	-1.94346	401.697	-2.51662
402.93	-1.9739	402.697	-2.58182
403.93	-2.00386	403.697	-2.64567
404.93	-2.03352	404.697	-2.70963
405.93	-2.07121	405.697	-2.77958
406.93	-2.10355	406.697	-2.85887
407.93	-2.151	407.697	-2.93576
408.93	-2.19645	408.697	-3.01528

409.93	-2.23334	409.697	-3.087
410.93	-2.2744	410.697	-3.15589
411.93	-2.32753	411.697	-3.24484
412.93	-2.38105	412.697	-3.3355
413.93	-2.4299	413.697	-3.42878
414.93	-2.48512	414.697	-3.51298
415.93	-2.51449	415.697	-3.59452
416.93	-2.55168	416.697	-3.68087
417.93	-2.61584	417.697	-3.78736
418.93	-2.67351	418.697	-3.90553
419.93	-2.71849	419.697	-4.01896
420.93	-2.76671	420.697	-4.1326
421.93	-2.82089	421.697	-4.24467
422.93	-2.85107	422.697	-4.36402
423.93	-2.91334	423.697	-4.48917
424.93	-2.98309	424.697	-4.62426
425.93	-3.02895	425.697	-4.75734
426.93	-3.07163	426.697	-4.87909
427.93	-3.12998	427.697	-5.00163
428.93	-3.1871	428.697	-5.12394
429.93	-3.23776	429.697	-5.25772
430.93	-3.31419	430.697	-5.39091
431.93	-3.38176	431.697	-5.52276
432.93	-3.42892	432.697	-5.65404
433.93	-3.47816	433.697	-5.77335
434.93	-3.53224	434.697	-5.88499
435.93	-3.5746	435.697	-5.99289
436.93	-3.61577	436.697	-6.08142
437.93	-3.65722	437.697	-6.1573
438.93	-3.68953	438.697	-6.22276
439.93	-3.71247	439.697	-6.26801
440.93	-3.75133	440.697	-6.28773
441.93	-3.7858	441.697	-6.29467
442.93	-3.81588	442.697	-6.28744
443.93	-3.84505	443.697	-6.25378
444.93	-3.87438	444.697	-6.20878
445.93	-3.89942	445.697	-6.13757
446.93	-3.91751	446.697	-6.04075
447.93	-3.93023	447.697	-5.92554
448.93	-3.93156	448.697	-5.78953
449.93	-3.93507	449.697	-5.62527
450.93	-3.93291	450.697	-5.43973
451.93	-3.9265	451.697	-5.23793

452.93	-3.91292	452.697	-5.01403
453.93	-3.90023	453.697	-4.77675
454.93	-3.8928	454.697	-4.52717
455.93	-3.88448	455.697	-4.27561
456.93	-3.87685	456.697	-4.02194
457.93	-3.86352	457.697	-3.7757
458.93	-3.84738	458.697	-3.53131
459.93	-3.83638	459.697	-3.30334
460.93	-3.82142	460.697	-3.08449
461.93	-3.79834	461.697	-2.8744
462.93	-3.75767	462.697	-2.67336
463.93	-3.71725	463.697	-2.49064
464.93	-3.67819	464.697	-2.3199
465.93	-3.63766	465.697	-2.15836
466.93	-3.59423	466.697	-2.00187
467.93	-3.53263	467.697	-1.84715
468.93	-3.46353	468.697	-1.70293
469.93	-3.39569	469.697	-1.57032
470.93	-3.3326	470.697	-1.4363
471.93	-3.26343	471.697	-1.31131
472.93	-3.18447	472.697	-1.19819
473.93	-3.09843	473.697	-1.08712
474.93	-3.00868	474.697	-0.98549
475.93	-2.93136	475.697	-0.89372
476.93	-2.85012	476.697	-0.81501
477.93	-2.77229	477.697	-0.74614
478.93	-2.69442	478.697	-0.68648
479.93	-2.60617	479.697	-0.62712
480.93	-2.50958	480.697	-0.57348
481.93	-2.43039	481.697	-0.53192
482.93	-2.34751	482.697	-0.49884
483.93	-2.26753	483.697	-0.46916
484.93	-2.19631	484.697	-0.43435
485.93	-2.1019	485.697	-0.39681
486.93	-2.00962	486.697	-0.36092
487.93	-1.92905	487.697	-0.33164
488.93	-1.85611	488.697	-0.30912
489.93	-1.77041	489.697	-0.28265
490.93	-1.68887	490.697	-0.25441
491.93	-1.59967	491.697	-0.2299
492.93	-1.51242	492.697	-0.21661
493.93	-1.43861	493.697	-0.2046
494.93	-1.37309	494.697	-0.19789

495.93	-1.31167	495.697	-0.19274
496.93	-1.24766	496.697	-0.18415
497.93	-1.18828	497.697	-0.17854
498.93	-1.12792	498.697	-0.17395
499.93	-1.0754	499.697	-0.16506
500.93	-1.01895	500.697	-0.15235
501.93	-0.97339	501.697	-0.14922
502.93	-0.9213	502.697	-0.14539
503.93	-0.87267	503.697	-0.13855
504.93	-0.8281	504.697	-0.13045
505.93	-0.78873	505.697	-0.12226
506.93	-0.75722	506.697	-0.12008
507.93	-0.72046	507.697	-0.11962
508.93	-0.68869	508.697	-0.11861
509.93	-0.65333	509.697	-0.11329
510.93	-0.6227	510.697	-0.10628
511.93	-0.58735	511.697	-0.09902295
512.93	-0.5568	512.697	-0.09590074
513.93	-0.52256	513.697	-0.09303883
514.93	-0.49239	514.697	-0.08952753
515.93	-0.46857	515.697	-0.09194107
516.93	-0.44516	516.697	-0.09531304
517.93	-0.4168	517.697	-0.08889978
518.93	-0.3883	518.697	-0.08222893
519.93	-0.36802	519.697	-0.08093516
520.93	-0.34837	520.697	-0.08193165
521.93	-0.33172	521.697	-0.08359005
522.93	-0.31081	522.697	-0.07635464
523.93	-0.28927	523.697	-0.0620373
524.93	-0.26803	524.697	-0.04662344
525.93	-0.25691	525.697	-0.04790035
526.93	-0.24637	526.697	-0.05010664
527.93	-0.23191	527.697	-0.04793485
528.93	-0.21693	528.697	-0.04493654
529.93	-0.20258	529.697	-0.04032823
530.93	-0.19188	530.697	-0.04110384
531.93	-0.17996	531.697	-0.04299583
532.93	-0.17485	532.697	-0.05078078
533.93	-0.16458	533.697	-0.05093563
534.93	-0.15997	534.697	-0.05172018
535.93	-0.15759	535.697	-0.05349611
536.93	-0.15272	536.697	-0.05019097
537.93	-0.1465	537.697	-0.04659685

538.93	-0.13804	538.697	-0.04419886
539.93	-0.13158	539.697	-0.04549747
540.93	-0.1243	540.697	-0.04370636
541.93	-0.12004	541.697	-0.0395405
542.93	-0.10678	542.697	-0.03228432
543.93	-0.09623648	543.697	-0.02719843
544.93	-0.08832579	544.697	-0.02920053
545.93	-0.08437255	545.697	-0.03355063
546.93	-0.08257363	546.697	-0.03925761
547.93	-0.08254723	547.697	-0.04378387
548.93	-0.07746873	548.697	-0.04401532
549.93	-0.07433032	549.697	-0.04662068
550.93	-0.07637267	550.697	-0.05369929
551.93	-0.07420761	551.697	-0.06157602
552.93	-0.0738526	552.697	-0.06586148
553.93	-0.06908177	553.697	-0.06743842
554.93	-0.06352791	554.697	-0.06051403
555.93	-0.05786924	555.697	-0.05258079
556.93	-0.05875415	556.697	-0.05005498
557.93	-0.05354445	557.697	-0.04907311
558.93	-0.04617094	558.697	-0.04556807
559.93	-0.04201494	559.697	-0.03583123
560.93	-0.0356868	560.697	-0.0253263
561.93	-0.03426224	561.697	-0.01902698
562.93	-0.03797722	562.697	-0.02294796
563.93	-0.04211211	563.697	-0.03068991
564.93	-0.04020072	564.697	-0.03560618
565.93	-0.03872964	565.697	-0.03527256
566.93	-0.03914552	566.697	-0.03387208
567.93	-0.04099202	567.697	-0.0397799
568.93	-0.04344989	568.697	-0.04879056
569.93	-0.04379322	569.697	-0.05469116
570.93	-0.03953734	570.697	-0.05655289
571.93	-0.02808255	571.697	-0.05383341
572.93	-0.01817459	572.697	-0.0493165
573.93	-0.0151338	573.697	-0.04973652
574.93	-0.01094286	574.697	-0.05002172
575.93	-0.003963065	575.697	-0.04551616
576.93	0.00444897	576.697	-0.03612059
577.93	0.01325161	577.697	-0.02614712
578.93	0.01780501	578.697	-0.01698674
579.93	0.01155669	579.697	-0.01102191
5.81E+02	0.00053835	580.697	-0.0120973

581.93	-0.005580656	581.697	-0.0109096
582.93	-0.01459701	582.697	-0.01262775
583.93	-0.02647626	583.697	-0.01811227
584.93	-0.03915357	584.697	-0.02749216
585.93	-0.05208597	585.697	-0.03729787
586.93	-0.05907481	586.697	-0.04534364
587.93	-0.05385862	587.697	-0.04738058
588.93	-0.04391856	588.697	-0.04258186
589.93	-0.03826163	589.697	-0.03880412
590.93	-0.0310567	590.697	-0.0373281
591.93	-0.02185216	591.697	-0.03505795
592.93	-0.01401355	592.697	-0.02917788
593.93	-0.009362889	593.697	-0.02594358
594.93	-0.007209347	594.697	-0.02456069
595.93	-0.01545829	595.697	-0.02885571
596.93	-0.02592372	596.697	-0.03708572
597.93	-0.02943347	597.697	-0.04318274
598.93	-0.03012149	598.697	-0.04203097
599.93	-0.03336809	599.697	-0.03811875
600.93	-0.03853777	600.697	-0.03777601
601.93	-0.03853223	601.697	-0.03436622
602.93	-0.03680444	602.697	-0.03079702
603.93	-0.02758783	603.697	-0.02342596
604.93	-0.02102432	604.697	-0.01687479
605.93	-0.02348093	605.697	-0.01760697
606.93	-0.02947911	606.697	-0.02264925
607.93	-0.03034176	607.697	-0.02727401
608.93	-0.02191795	608.697	-0.02874298
609.93	-0.01618539	609.697	-0.03531809
610.93	-0.01398835	610.697	-0.0377731
611.93	-0.02113253	611.697	-0.04424844
612.93	-0.0207218	612.697	-0.0453192
613.93	-0.01232929	613.697	-0.03597408
614.93	-0.003803171	614.697	-0.02695866
615.93	-0.001074255	615.697	-0.02391275
616.93	-0.005600819	616.697	-0.02388605
617.93	-0.01053597	617.697	-0.01646578
618.93	-0.01165479	618.697	-0.01167941
619.93	-0.004285589	619.697	-0.00491301
620.93	-0.006875236	620.697	-0.00691475
621.93	-0.0140748	621.697	-0.01497578
622.93	-0.01863876	622.697	-0.01919475
623.93	-0.02064278	623.697	-0.01768513

624.93	-0.02047253	624.697	-0.01256474
625.93	-0.01951686	625.697	-0.00993945
626.93	-0.02147917	626.697	-0.00992397
627.93	-0.03052649	627.697	-0.01588761
628.93	-0.03281859	628.697	-0.01502891
629.93	-0.03144272	629.697	-0.01277929
630.93	-0.03041027	630.697	-0.01435036
631.93	-0.02984479	631.697	-0.01738847
632.93	-0.0315951	632.697	-0.02186095
633.93	-0.03330982	633.697	-0.02783928
634.93	-0.03093652	634.697	-0.02842287
635.93	-0.02530443	635.697	-0.02111388
636.93	-0.02187301	636.697	-0.01596157
637.93	-0.0194656	637.697	-0.01231642
638.93	-0.01863356	638.697	-0.00872054
639.93	-0.01647272	639.697	-0.00533861
640.93	-0.009283252	640.697	0.00212845
641.93	-0.006391031	641.697	0.00752748
642.93	-0.01284844	642.697	0.00182208
643.93	-0.01820216	643.697	-0.00729109
644.93	-0.01967824	644.697	-0.01159419
645.93	-0.02051633	645.697	-0.01111473
646.93	-0.01866604	646.697	-0.00553174
647.93	-0.02043222	6.48E+02	-0.00093993
648.93	-0.02797357	648.697	-0.0049652
649.93	-0.02882035	649.697	-0.00550522
650.93	-0.01952709	650.697	0.0059882
651.93	-0.0142172	651.697	0.0159396
652.93	-0.01472974	652.697	0.01886984
653.93	-0.01457914	653.697	0.02029213
654.93	-0.01604233	654.697	0.01661935
655.93	-0.01328332	655.697	0.01285872
656.93	-0.01088662	656.697	0.01027486
657.93	-0.01482948	657.697	0.00112222
658.93	-0.02130298	658.697	-0.01176298
659.93	-0.0211035	659.697	-0.017168
660.93	-0.01772274	660.697	-0.01702178
661.93	-0.01737743	661.697	-0.01874965
662.93	-0.01951588	662.697	-0.02368775
663.93	-0.02450638	663.697	-0.02898238
664.93	-0.02907091	664.697	-0.03094996
665.93	-0.03093989	665.697	-0.02583044
666.93	-0.03379972	666.697	-0.02031244

667.93	-0.03983774	667.697	-0.02193324
668.93	-0.04482574	668.697	-0.02635709
669.93	-0.05052338	669.697	-0.03292846
670.93	-0.0551366	670.697	-0.03585331
671.93	-0.05737925	671.697	-0.03469502
672.93	-0.05450885	672.697	-0.02984912
673.93	-0.0485839	673.697	-0.02591289
674.93	-0.04333636	674.697	-0.02421818
675.93	-0.03945806	675.697	-0.02292534
676.93	-0.03768692	676.697	-0.02106202
677.93	-0.03498747	677.697	-0.01522087
678.93	-0.02993654	678.697	-0.00726671
679.93	-0.02542099	679.697	-0.00129267
680.93	-0.02698335	680.697	-0.0010759
681.93	-0.03279539	681.697	-0.00487073
682.93	-0.03624309	682.697	-0.00561905
683.93	-0.03650358	6.84E+02	-7.8547E-05
684.93	-0.0363211	684.697	0.00390506
685.93	-0.03588696	685.697	0.00448929
686.93	-0.03753116	686.697	0.00438282
687.93	-0.04079546	687.697	0.00276138
688.93	-0.04082261	6.89E+02	0.00030978
689.93	-0.03641054	689.697	0.00251568
690.93	-0.03373996	690.697	0.00283752
691.93	-0.03466058	691.697	-0.00162199
692.93	-0.035268	692.697	-0.00269428
693.93	-0.03339379	6.94E+02	0.00069066
694.93	-0.02975896	694.697	0.00341381
695.93	-0.02604353	695.697	0.00422192
696.93	-0.0236546	696.697	0.00944613
697.93	-0.026334	697.697	0.01416583
698.93	-0.03050276	698.697	0.01685472
699.93	-0.03264621	699.697	0.01630981
700.93	-0.03214334	700.697	0.01408647
701.93	-0.03407699	701.697	0.00761648
702.93	-0.03974737	702.697	-0.00235556
703.93	-0.04557509	703.697	-0.00806079
704.93	-0.05120884	704.697	-0.01620776
705.93	-0.05191678	705.697	-0.0272841
706.93	-0.04663738	706.697	-0.03068549
707.93	-0.04237153	707.697	-0.02401412
708.93	-0.0454371	708.697	-0.02043149
709.93	-0.0482972	709.697	-0.015453

710.93	-0.04755512	710.697	-0.00837625
711.93	-0.04311173	711.697	-0.00365604
712.93	-0.03687947	712.697	0.0016535
713.93	-0.03290183	713.697	0.01102879
714.93	-0.03406602	714.697	0.01194985
715.93	-0.03338506	715.697	0.00479275
716.93	-0.02758963	7.17E+02	-7.7606E-05
717.93	-0.02303575	717.697	-0.00535965
718.93	-0.0237266	718.697	-0.00963655
719.93	-0.02591119	719.697	-0.01137328
720.93	-0.02803562	720.697	-0.00975871
721.93	-0.0311026	721.697	-0.01177152
722.93	-0.03761808	722.697	-0.01160061
723.93	-0.04932888	723.697	-0.01046127
724.93	-0.06132472	724.697	-0.00981795
725.93	-0.06615309	725.697	-0.00653124
726.93	-0.05928435	726.697	-0.00166394
727.93	-0.05482944	727.697	0.00283131
728.93	-0.05728291	7.29E+02	-0.00017356
729.93	-0.05738159	729.697	-0.00267902
730.93	-0.04960909	730.697	-0.00437044
731.93	-0.03706034	731.697	-0.00439208
732.93	-0.02369297	7.33E+02	-0.00064831
733.93	-0.02057018	733.697	0.00173304
734.93	-0.02889912	734.697	0.00287362
735.93	-0.03548958	7.36E+02	0.00072094
736.93	-0.03183547	736.697	0.00241177
737.93	-0.02599483	737.697	0.00645604
738.93	-0.02497516	738.697	0.01216926
739.93	-0.02764715	739.697	0.01610151
740.93	-0.03320946	740.697	0.01418485
741.93	-0.03304022	741.697	0.01095397
742.93	-0.02825009	742.697	0.00535185
743.93	-0.02334779	743.697	0.00259038
744.93	-0.02230807	744.697	0.00219672
745.93	-0.02451761	7.46E+02	0.00068853
746.93	-0.02458573	746.697	-0.00211217
747.93	-0.02070469	747.697	-0.00237378
748.93	-0.01325192	7.49E+02	0.00054635
749.93	-0.006186739	749.697	0.00494799
7.51E+02	0.000687749	750.697	0.01172608
751.93	0.006374458	751.697	0.01676511
752.93	0.01010717	752.697	0.0188479

753.93	0.01103189	753.697	0.01868323
754.93	0.01031944	754.697	0.01652128
755.93	0.008297333	755.697	0.01346203
756.93	0.005164103	756.697	0.01091405
757.93	0.001574752	757.697	0.00802077
758.93	-0.002832549	758.697	0.00347799
759.93	-0.007125867	7.60E+02	-0.00054144
760.93	-0.01056814	760.697	-0.00352841
761.93	-0.01108335	761.697	-0.00496969
762.93	-0.01066172	762.697	-0.004521
763.93	-0.01176906	763.697	-0.00417191
764.93	-0.01338362	764.697	-0.0056831
765.93	-0.0150418	765.697	-0.00922676
766.93	-0.01625806	766.697	-0.01115863
767.93	-0.01687824	767.697	-0.01163295
768.93	-0.01694774	768.697	-0.01245222
769.93	-0.01539891	769.697	-0.0122796
770.93	-0.01330673	770.697	-0.00896922
771.93	-0.01165152	771.697	-0.00776981
772.93	-0.01060356	772.697	-0.00788895
773.93	-0.009929599	773.697	-0.00470095
774.93	-0.01083363	774.697	-0.00298109
775.93	-0.01089535	775.697	-0.00457424
776.93	-0.008098017	776.697	-0.00529225
777.93	-0.006694179	777.697	-0.00511317
778.93	-0.008425346	778.697	-0.01183091
779.93	-0.007767511	779.697	-0.01607971
780.93	-0.007737573	780.697	-0.01718169
781.93	-0.003729359	781.697	-0.01726425
782.93	0.004019313	782.697	-0.01517286
783.93	0.007799297	783.697	-0.00943999
784.93	0.005920369	784.697	-0.00499649
785.93	0.002511829	785.697	-0.00557759
786.93	0.006774529	786.697	-0.0030688
787.93	0.008495978	7.88E+02	-0.000714
788.93	0.009791917	788.697	0.00297073
789.93	0.006884757	789.697	0.00280556
790.93	0.004904153	7.91E+02	0.0003565
791.93	0.001890014	791.697	-0.00313075
792.93	-0.01037007	792.697	-0.00524267
793.93	0.01593114	793.697	-0.00414392
794.93	0.01535211	794.697	-0.00239928
795.93	0.02826274	795.697	-0.00435305

796.93	0.22106	7.97E+02	-3.0846E-05

DSC curve of water-immersed brown coal

temperature	***DSC***	***temperature***	***DSC***	***temperature***	***DSC***
℃	mW/mg	℃	mW/mg	℃	mW/mg
	Coal		Coal+MgCl2		Coal+TEMPO
26.178	0.0407041	28.92	0.003669648	28.473	-0.06133812
27.178	-0.02216885	29.92	-0.05468266	29.473	-0.09580251
28.178	-0.07609614	30.92	-0.1042	30.473	-0.13307
29.178	-0.12143	31.92	-0.14522	31.473	-0.16638
30.178	-0.14722	32.92	-0.16743	32.473	-0.18607
31.178	-0.16002	33.92	-0.17478	33.473	-0.1917
32.178	-0.16646	34.92	-0.17452	34.473	-0.19016
33.178	-0.16978	35.92	-0.17185	35.473	-0.18646
34.178	-0.17197	36.92	-0.16889	36.473	-0.18275
35.178	-0.17373	37.92	-0.16619	37.473	-0.17931
36.178	-0.17527	38.92	-0.16395	38.473	-0.17666
37.178	-0.17676	39.92	-0.16203	39.473	-0.17447
38.178	-0.17829	40.92	-0.16042	40.473	-0.17289
39.178	-0.17979	41.92	-0.15909	41.473	-0.1716
40.178	-0.18127	42.92	-0.15801	42.473	-0.17064
41.178	-0.18282	43.92	-0.15714	43.473	-0.16994
42.178	-0.18442	44.92	-0.15646	44.473	-0.16935
43.178	-0.18604	45.92	-0.1559	45.473	-0.16885
44.178	-0.1876	46.92	-0.15542	46.473	-0.16837
45.178	-0.18907	47.92	-0.15499	47.473	-0.16786
46.178	-0.1905	48.92	-0.1546	48.473	-0.16736
47.178	-0.19176	49.92	-0.15421	49.473	-0.16682
48.178	-0.19289	50.92	-0.15379	50.473	-0.16614
49.178	-0.19384	51.92	-0.15337	51.473	-0.16531
50.178	-0.19458	52.92	-0.15289	52.473	-0.16444
51.178	-0.19516	53.92	-0.15237	53.473	-0.16337
52.178	-0.1955	54.92	-0.15192	54.473	-0.16217
53.178	-0.19561	55.92	-0.15162	55.473	-0.1608
54.178	-0.19547	56.92	-0.15167	56.473	-0.15919
55.178	-0.19509	57.92	-0.15225	57.473	-0.15736
56.178	-0.19444	58.92	-0.15373	58.473	-0.15532
57.178	-0.19344	59.92	-0.15615	59.473	-0.15309
58.178	-0.19221	60.92	-0.15985	60.473	-0.15064
59.178	-0.19065	61.92	-0.16475	61.473	-0.14783
60.178	-0.18879	62.92	-0.17109	62.473	-0.14498
61.178	-0.18657	63.92	-0.17853	63.473	-0.14164

62.178	-0.18408	64.92	-0.18717	64.473	-0.13824
63.178	-0.18116	65.92	-0.19685	65.473	-0.13458
64.178	-0.17802	66.92	-0.20718	66.473	-0.13073
65.178	-0.17449	67.92	-0.21732	67.473	-0.12669
66.178	-0.17081	68.92	-0.22716	68.473	-0.12241
67.178	-0.16675	69.92	-0.2361	69.473	-0.11799
68.178	-0.1624	70.92	-0.24374	70.473	-0.11334
69.178	-0.15783	71.92	-0.24949	71.473	-0.1086
70.178	-0.15296	72.92	-0.2533	72.473	-0.10358
71.178	-0.14777	73.92	-0.25482	73.473	-0.09843391
72.178	-0.14224	74.92	-0.25395	74.473	-0.09326989
73.178	-0.1367	75.92	-0.2508	75.473	-0.08784898
74.178	-0.13072	76.92	-0.24549	76.473	-0.08216682
75.178	-0.12467	77.92	-0.23835	77.473	-0.07656158
76.178	-0.1182	78.92	-0.22953	78.473	-0.07087283
77.178	-0.11185	79.92	-0.21959	79.473	-0.0650136
78.178	-0.10524	80.92	-0.20866	80.473	-0.05909513
79.178	-0.09844523	81.92	-0.19757	81.473	-0.05303918
80.178	-0.09179006	82.92	-0.18615	82.473	-0.04726519
81.178	-0.08498937	83.92	-0.17512	83.473	-0.04125436
82.178	-0.07799196	84.92	-0.16413	84.473	-0.03541483
83.178	-0.07114697	85.92	-0.15357	85.473	-0.02940084
84.178	-0.06398704	86.92	-0.14322	86.473	-0.02347553
85.178	-0.05706904	87.92	-0.13338	87.473	-0.0176302
86.178	-0.05023246	88.92	-0.12366	88.473	-0.0117336
87.178	-0.04351367	89.92	-0.11453	89.473	-0.005912084
88.178	-0.03672598	90.92	-0.1058	90.473	-0.000126453
89.178	-0.02989085	91.92	-0.09802463	91.473	0.005671159
90.178	-0.02309003	92.92	-0.09161111	92.473	0.01150708
91.178	-0.01651984	93.92	-0.08699769	93.473	0.01717152
92.178	-0.00974109	94.92	-0.08454736	94.473	0.02284347
93.178	-0.00313001	95.92	-0.08452455	95.473	0.02849853
94.178	0.003256431	96.92	-0.08714089	96.473	0.0341291
95.178	0.00963993	97.92	-0.09256392	97.473	0.0396229
96.178	0.01604644	98.92	-0.10047	98.473	0.04507489
97.178	0.0223734	99.92	-0.11051	99.473	0.05050047
98.178	0.02862925	100.92	-0.12241	100.473	0.05594962
99.178	0.03475161	101.92	-0.13511	101.473	0.06128431
100.178	0.0409102	102.92	-0.14739	102.473	0.0667939
101.178	0.04702038	103.92	-0.15765	103.473	0.07221172
102.178	0.0530861	104.92	-0.16467	104.473	0.07768394
103.178	0.05905875	105.92	-0.16672	105.473	0.08302219
104.178	0.06500465	106.92	-0.16328	106.473	0.08837331

105.178	0.07095536	107.92	-0.15433	107.473	0.09378626
106.178	0.07674927	108.92	-0.1404	108.473	0.09922277
107.178	0.08262835	109.92	-0.12168	109.473	0.1046
108.178	0.08838707	110.92	-0.09954326	110.473	0.10996
109.178	0.09421971	111.92	-0.0738848	111.473	0.11539
110.178	0.09998346	112.92	-0.04667018	112.473	0.12086
111.178	0.10573	113.92	-0.01934845	113.473	0.12636
112.178	0.11146	114.92	0.006793171	114.473	0.13196
113.178	0.1173	115.92	0.03074504	115.473	0.13756
114.178	0.12305	116.92	0.05239447	116.473	0.14327
115.178	0.12893	117.92	0.07216417	117.473	0.14894
116.178	0.1349	118.92	0.08959293	118.473	0.15468
117.178	0.14081	119.92	0.1056	119.473	0.16067
118.178	0.14695	120.92	0.12051	120.473	0.16671
119.178	0.15288	121.92	0.1337	121.473	0.17274
120.178	0.15907	122.92	0.1463	122.473	0.1789
121.178	0.16522	123.92	0.15791	123.473	0.18502
122.178	0.17152	124.92	0.16892	124.473	0.19136
123.178	0.17783	125.92	0.17914	125.473	0.19782
124.178	0.1842	126.92	0.18875	126.473	0.20438
125.178	0.19061	127.92	0.19805	127.473	0.2111
126.178	0.19706	128.92	0.20659	128.473	0.21786
127.178	0.20356	129.92	0.2144	129.473	0.22463
128.178	0.21023	130.92	0.22141	130.473	0.23167
129.178	0.21685	131.92	0.22762	131.473	0.23876
130.178	0.22361	132.92	0.23285	132.473	0.24592
131.178	0.23039	133.92	0.2372	133.473	0.25331
132.178	0.23715	134.92	0.24051	134.473	0.26075
133.178	0.24438	135.92	0.24288	135.473	0.26849
134.178	0.25152	136.92	0.24455	136.473	0.27627
135.178	0.25886	137.92	0.24568	137.473	0.28424
136.178	0.26637	138.92	0.24652	138.473	0.2924
137.178	0.274	139.92	0.24751	139.473	0.30062
138.178	0.28187	140.92	0.24898	140.473	0.30922
139.178	0.28977	141.92	0.25116	141.473	0.31771
140.178	0.29792	142.92	0.25448	142.473	0.32641
141.178	0.30605	143.92	0.25925	143.473	0.33533
142.178	0.31439	144.92	0.26581	144.473	0.34438
143.178	0.32288	145.92	0.27406	145.473	0.35368
144.178	0.33155	146.92	0.28432	146.473	0.36306
145.178	0.3403	147.92	0.29615	147.473	0.37284
146.178	0.34946	148.92	0.30923	148.473	0.38249
147.178	0.35859	149.92	0.32363	149.473	0.39255

148.178	0.36771	150.92	0.33881	150.473	0.40288
149.178	0.37732	151.92	0.35435	151.473	0.41347
150.178	0.38707	152.92	0.37012	152.473	0.42408
151.178	0.39683	153.92	0.38528	153.473	0.43495
152.178	0.40695	154.92	0.39985	154.473	0.44602
153.178	0.41702	155.92	0.41384	155.473	0.4571
154.178	0.42754	156.92	0.42695	156.473	0.46853
155.178	0.43816	157.92	0.43926	157.473	0.48006
156.178	0.44887	158.92	0.45082	158.473	0.49185
157.178	0.45982	159.92	0.46181	159.473	0.5039
158.178	0.47077	160.92	0.47223	160.473	0.51601
159.178	0.4821	161.92	0.48222	161.473	0.5286
160.178	0.49358	162.92	0.49173	162.473	0.54128
161.178	0.50501	163.92	0.50091	163.473	0.55436
162.178	0.51684	164.92	0.51014	164.473	0.56762
163.178	0.52895	165.92	0.51943	165.473	0.58112
164.178	0.54126	166.92	0.52918	166.473	0.59526
165.178	0.5537	167.92	0.53938	167.473	0.60921
166.178	0.56651	168.92	0.55038	168.473	0.62363
167.178	0.57923	169.92	0.56221	169.473	0.63787
168.178	0.59228	170.92	0.57454	170.473	0.65261
169.178	0.60537	171.92	0.58748	171.473	0.66753
170.178	0.619	172.92	0.6009	172.473	0.68258
171.178	0.63217	173.92	0.61487	173.473	0.69805
172.178	0.64578	174.92	0.62889	174.473	0.71371
173.178	0.65944	175.92	0.64278	175.473	0.72966
174.178	0.6733	176.92	0.65625	176.473	0.74557
175.178	0.68757	177.92	0.66969	177.473	0.762
176.178	0.70186	178.92	0.68281	178.473	0.77839
177.178	0.71638	179.92	0.69581	179.473	0.79535
178.178	0.73102	180.92	0.70921	180.473	0.81229
179.178	0.74633	181.92	0.72189	181.473	0.82989
180.178	0.76132	182.92	0.73484	182.473	0.8474
181.178	0.77683	183.92	0.74783	183.473	0.86507
182.178	0.79253	184.92	0.76082	184.473	0.8829
183.178	0.80804	185.92	0.7738	185.473	0.90104
184.178	0.82418	186.92	0.78691	186.473	0.91947
185.178	0.84024	187.92	0.80014	187.473	0.93845
186.178	0.85672	188.92	0.81315	188.473	0.95717
187.178	0.87367	189.92	0.82637	189.473	0.97633
188.178	0.88998	190.92	0.83943	190.473	0.99565
189.178	0.90725	191.92	0.85277	191.473	1.01525
190.178	0.9242	192.92	0.86581	192.473	1.03492

191.178	0.9419	193.92	0.87881	193.473	1.05498
192.178	0.95929	194.92	0.89171	194.473	1.07525
193.178	0.97712	195.92	0.90474	195.473	1.09535
194.178	0.9953	196.92	0.91778	196.473	1.11561
195.178	1.01342	197.92	0.9308	197.473	1.13641
196.178	1.03173	198.92	0.94385	198.473	1.15732
197.178	1.04994	199.92	0.95727	199.473	1.17832
198.178	1.06899	200.92	0.97068	200.473	1.19903
199.178	1.08812	201.92	0.98426	201.473	1.22033
200.178	1.10745	202.92	0.99869	202.473	1.2417
201.178	1.12672	203.92	1.01344	203.473	1.26315
202.178	1.14648	204.92	1.02812	204.473	1.28486
203.178	1.16655	205.92	1.04369	205.473	1.30675
204.178	1.18666	206.92	1.05975	206.473	1.32853
205.178	1.20713	207.92	1.07595	207.473	1.35006
206.178	1.22787	208.92	1.09276	208.473	1.37274
207.178	1.24869	209.92	1.10993	209.473	1.39466
208.178	1.26917	210.92	1.12728	210.473	1.4171
209.178	1.29064	211.92	1.14482	211.473	1.43937
210.178	1.31223	212.92	1.16272	212.473	1.46173
211.178	1.33354	213.92	1.18054	213.473	1.48364
212.178	1.35517	214.92	1.19873	214.473	1.50639
213.178	1.37761	215.92	1.21672	215.473	1.52877
214.178	1.3996	216.92	1.2352	216.473	1.55091
215.178	1.42206	217.92	1.25429	217.473	1.57363
216.178	1.44482	218.92	1.27426	218.473	1.59639
217.178	1.46774	219.92	1.29543	219.473	1.61908
218.178	1.49103	220.92	1.31732	220.473	1.64206
219.178	1.51451	221.92	1.34044	221.473	1.6646
220.178	1.53841	222.92	1.36499	222.473	1.6873
221.178	1.56228	223.92	1.38978	223.473	1.71054
222.178	1.58705	224.92	1.41548	224.473	1.73358
223.178	1.6118	225.92	1.44124	225.473	1.75698
224.178	1.6368	226.92	1.46656	226.473	1.78006
225.178	1.66174	227.92	1.49168	227.473	1.80374
226.178	1.68736	228.92	1.51622	228.473	1.82753
227.178	1.71328	229.92	1.54047	229.473	1.85147
228.178	1.73923	230.92	1.5641	230.473	1.87572
229.178	1.76568	231.92	1.58815	231.473	1.90049
230.178	1.79232	232.92	1.6118	232.473	1.92494
231.178	1.81885	233.92	1.63533	233.473	1.94987
232.178	1.84622	234.92	1.65905	234.473	1.97527
233.178	1.87385	235.92	1.68314	235.473	2.00097

234.178	1.90099	236.92	1.70697	236.473	2.02733
235.178	1.92919	237.92	1.73127	237.473	2.05328
236.178	1.95776	238.92	1.75562	238.473	2.07987
237.178	1.98622	239.92	1.78034	239.473	2.10689
238.178	2.01483	240.92	1.80503	240.473	2.13426
239.178	2.04424	241.92	1.83026	241.473	2.16222
240.178	2.07349	242.92	1.85577	242.473	2.19035
241.178	2.10305	243.92	1.88135	243.473	2.21895
242.178	2.13343	244.92	1.90706	244.473	2.24841
243.178	2.16347	245.92	1.93347	245.473	2.27857
244.178	2.19396	246.92	1.96021	246.473	2.30852
245.178	2.22485	247.92	1.98686	247.473	2.33945
246.178	2.2561	248.92	2.014	248.473	2.37092
247.178	2.28741	249.92	2.04083	249.473	2.40261
248.178	2.31945	250.92	2.06812	250.473	2.43434
249.178	2.3515	251.92	2.09622	251.473	2.46744
250.178	2.38375	252.92	2.1243	252.473	2.50047
251.178	2.41672	253.92	2.1529	253.473	2.53393
252.178	2.44957	254.92	2.18106	254.473	2.56835
253.178	2.48257	255.92	2.20997	255.473	2.6026
254.178	2.51557	256.92	2.23887	256.473	2.63675
255.178	2.55012	257.92	2.26792	257.473	2.67136
256.178	2.58436	258.92	2.29764	258.473	2.70734
257.178	2.6193	259.92	2.3276	259.473	2.7426
258.178	2.65414	260.92	2.35778	260.473	2.7784
259.178	2.68938	261.92	2.38812	261.473	2.81466
260.178	2.72447	262.92	2.41872	262.473	2.85139
261.178	2.7599	263.92	2.4498	263.473	2.88757
262.178	2.79576	264.92	2.48107	264.473	2.92436
263.178	2.8304	265.92	2.51251	265.473	2.96137
264.178	2.86666	266.92	2.54426	266.473	2.99884
265.178	2.90113	267.92	2.57617	267.473	3.03586
266.178	2.93639	268.92	2.60762	268.473	3.07315
267.178	2.97165	269.92	2.63993	269.473	3.11005
268.178	3.00787	270.92	2.67264	270.473	3.14779
269.178	3.04344	271.92	2.70496	271.473	3.18524
270.178	3.08002	272.92	2.73745	272.473	3.22299
271.178	3.11648	273.92	2.77019	273.473	3.26075
272.178	3.15346	274.92	2.80342	274.473	3.2975
273.178	3.19012	275.92	2.83661	275.473	3.33426
274.178	3.22708	276.92	2.87021	276.473	3.37084
275.178	3.264	277.92	2.9037	277.473	3.40788
276.178	3.30037	278.92	2.93687	278.473	3.44449

277.178	3.33795	279.92	2.97043	279.473	3.48072
278.178	3.37489	280.92	3.0042	280.473	3.51705
279.178	3.41112	281.92	3.0382	281.473	3.55276
280.178	3.44855	282.92	3.0719	282.473	3.58806
281.178	3.48504	283.92	3.10523	283.473	3.62367
282.178	3.52152	284.92	3.1389	284.473	3.65921
283.178	3.55774	285.92	3.17262	285.473	3.69438
284.178	3.59309	286.92	3.20601	286.473	3.72944
285.178	3.62813	287.92	3.24021	287.473	3.764
286.178	3.66217	288.92	3.27416	288.473	3.79731
287.178	3.69659	289.92	3.30844	289.473	3.83075
288.178	3.73	290.92	3.34244	290.473	3.86372
289.178	3.76305	291.92	3.37611	291.473	3.8967
290.178	3.79601	292.92	3.40956	292.473	3.92879
291.178	3.82894	293.92	3.44279	293.473	3.96056
292.178	3.86165	294.92	3.47555	294.473	3.99175
293.178	3.89535	295.92	3.50832	295.473	4.02206
294.178	3.92727	296.92	3.54051	296.473	4.05121
295.178	3.9603	297.92	3.57243	297.473	4.08028
296.178	3.99223	298.92	3.60344	298.473	4.10869
297.178	4.02312	299.92	3.63534	299.473	4.13689
298.178	4.05398	300.92	3.66601	300.473	4.16388
299.178	4.0836	301.92	3.69677	301.473	4.19052
300.178	4.11272	302.92	3.7267	302.473	4.21682
301.178	4.14133	303.92	3.7561	303.473	4.24268
302.178	4.16948	304.92	3.7846	304.473	4.26788
303.178	4.19654	305.92	3.81416	305.473	4.29256
304.178	4.22284	306.92	3.84176	306.473	4.3161
305.178	4.24836	307.92	3.86943	307.473	4.33907
306.178	4.27333	308.92	3.89682	308.473	4.36105
307.178	4.29722	309.92	3.92312	309.473	4.3829
308.178	4.32047	310.92	3.94957	310.473	4.40386
309.178	4.34281	311.92	3.97512	311.473	4.42442
310.178	4.36449	312.92	4.00026	312.473	4.44449
311.178	4.38545	313.92	4.02451	313.473	4.46374
312.178	4.4064	314.92	4.04817	314.473	4.48242
313.178	4.42659	315.92	4.07171	315.473	4.50049
314.178	4.44631	316.92	4.09432	316.473	4.51809
315.178	4.46522	317.92	4.11693	317.473	4.53516
316.178	4.48415	318.92	4.13871	318.473	4.55172
317.178	4.50244	319.92	4.16017	319.473	4.56825
318.178	4.52038	320.92	4.18115	320.473	4.58418
319.178	4.53783	321.92	4.20181	321.473	4.60014

320.178	4.55455	322.92	4.22214	322.473	4.61551
321.178	4.57113	323.92	4.24189	323.473	4.63091
322.178	4.58763	324.92	4.26115	324.473	4.64571
323.178	4.60405	325.92	4.28042	325.473	4.66021
324.178	4.62016	326.92	4.29894	326.473	4.67424
325.178	4.63608	327.92	4.31793	327.473	4.68783
326.178	4.65157	328.92	4.33682	328.473	4.70117
327.178	4.66684	329.92	4.35584	329.473	4.71437
328.178	4.68169	330.92	4.37452	330.473	4.72762
329.178	4.69707	331.92	4.39329	331.473	4.74099
330.178	4.71212	332.92	4.41208	332.473	4.75471
331.178	4.72758	333.92	4.43053	333.473	4.76886
332.178	4.74324	334.92	4.44938	334.473	4.7834
333.178	4.75924	335.92	4.46795	335.473	4.79815
334.178	4.7757	336.92	4.48647	336.473	4.81309
335.178	4.79233	337.92	4.5051	337.473	4.82793
336.178	4.80947	338.92	4.52347	338.473	4.84231
337.178	4.82684	339.92	4.54229	339.473	4.85709
338.178	4.84496	340.92	4.56183	340.473	4.87167
339.178	4.86368	341.92	4.58186	341.473	4.88695
340.178	4.88324	342.92	4.60278	342.473	4.90309
341.178	4.90389	343.92	4.6245	343.473	4.92011
342.178	4.92655	344.92	4.64694	344.473	4.93863
343.178	4.95017	345.92	4.67017	345.473	4.95887
344.178	4.97495	346.92	4.69388	346.473	4.9806
345.178	5.00083	347.92	4.71827	347.473	5.00467
346.178	5.02818	348.92	4.74323	348.473	5.02987
347.178	5.05639	349.92	4.76874	349.473	5.05628
348.178	5.08481	350.92	4.79512	350.473	5.08388
349.178	5.11431	351.92	4.822	351.473	5.11255
350.178	5.14501	352.92	4.85012	352.473	5.14231
351.178	5.17604	353.92	4.87874	353.473	5.17245
352.178	5.20979	354.92	4.90848	354.473	5.20374
353.178	5.2456	355.92	4.93895	355.473	5.23674
354.178	5.2829	356.92	4.97074	356.473	5.27067
355.178	5.32339	357.92	5.0026	357.473	5.3075
356.178	5.36662	358.92	5.03581	358.473	5.34612
357.178	5.41247	359.92	5.07	359.473	5.38736
358.178	5.46084	360.92	5.10523	360.473	5.43136
359.178	5.51144	361.92	5.14117	361.473	5.4776
360.178	5.56482	362.92	5.1782	362.473	5.52734
361.178	5.62167	363.92	5.21536	363.473	5.57875
362.178	5.68095	364.92	5.25362	364.473	5.63288

363.178	5.74473	365.92	5.29289	365.473	5.69064
364.178	5.81208	366.92	5.33375	366.473	5.7504
365.178	5.88247	367.92	5.37492	367.473	5.81305
366.178	5.95743	368.92	5.41717	368.473	5.87918
367.178	6.03655	369.92	5.46014	369.473	5.9483
368.178	6.12036	370.92	5.50451	370.473	6.02048
369.178	6.20707	371.92	5.54987	371.473	6.09637
370.178	6.29989	372.92	5.59621	372.473	6.17677
371.178	6.39822	373.92	5.64278	373.473	6.26184
372.178	6.50109	374.92	5.69055	374.473	6.35034
373.178	6.61072	375.92	5.73967	375.473	6.44492
374.178	6.72532	376.92	5.78898	376.473	6.54372
375.178	6.84674	377.92	5.83971	377.473	6.64825
376.178	6.97553	378.92	5.89031	378.473	6.75789
377.178	7.11313	379.92	5.94374	379.473	6.87324
378.178	7.25687	380.92	5.99784	380.473	6.99661
379.178	7.40862	381.92	6.05398	381.473	7.12711
380.178	7.57044	382.92	6.11148	382.473	7.26048
381.178	7.73702	383.92	6.16984	383.473	7.40397
382.178	7.9132	384.92	6.22872	384.473	7.55505
383.178	8.10103	385.92	6.28879	385.473	7.71053
384.178	8.29933	386.92	6.34931	386.473	7.87756
385.178	8.50632	387.92	6.41008	387.473	8.04968
386.178	8.72481	388.92	6.47014	388.473	8.22977
387.178	8.95414	389.92	6.53033	389.473	8.42067
388.178	9.19216	390.92	6.58911	390.473	8.61887
389.178	9.44557	391.92	6.64731	391.473	8.82833
390.178	9.70703	392.92	6.70395	392.473	9.04581
391.178	9.98498	393.92	6.75879	393.473	9.27073
392.178	10.27424	394.92	6.81305	394.473	9.5016
393.178	10.57876	395.92	6.86543	395.473	9.74875
394.178	10.89141	396.92	6.91628	396.473	10.00107
395.178	11.21843	397.92	6.9667	397.473	10.26275
396.178	11.56199	398.92	7.01542	398.473	10.53337
397.178	11.91895	399.92	7.06692	399.473	10.81302
398.178	12.29716	400.92	7.11698	400.473	11.10374
399.178	12.69061	401.92	7.17118	401.473	11.40438
400.178	13.10372	402.92	7.22675	402.473	11.71121
401.178	13.54261	403.92	7.28676	403.473	12.02625
402.178	14.00021	404.92	7.35006	404.473	12.35395
403.178	14.49647	405.92	7.41838	405.473	12.69686
404.178	15.02031	406.92	7.49394	406.473	13.03935
405.178	15.58576	407.92	7.57747	407.473	13.40352

406.178	16.17799	408.92	7.66876	408.473	13.78412
407.178	16.80691	409.92	7.76929	409.473	14.17061
408.178	17.46296	410.92	7.87988	410.473	14.57409
409.178	18.11467	411.92	8.00216	411.473	14.98309
410.178	18.76793	412.92	8.1355	412.473	15.40829
411.178	19.40543	413.92	8.28228	413.473	15.84498
412.178	20.00114	414.92	8.44089	414.473	16.27801
413.178	20.54112	415.92	8.61476	415.473	16.71497
414.178	21.00831	416.92	8.80333	416.473	17.12872
415.178	21.37994	417.92	9.00512	417.473	17.53412
416.178	21.65318	418.92	9.23013	418.473	17.89459
417.178	21.80398	419.92	9.47294	419.473	18.22279
418.178	21.82166	420.92	9.73444	420.473	18.49211
419.178	21.70469	421.92	10.02016	421.473	18.68893
420.178	21.44893	422.92	10.32419	422.473	18.80674
421.178	21.05067	423.92	10.65506	423.473	18.83534
422.178	20.53795	424.92	11.00558	424.473	18.76488
423.178	19.90703	425.92	11.37234	425.473	18.59872
424.178	19.20384	426.92	11.7591	426.473	18.32811
425.178	18.41923	427.92	12.16073	427.473	17.96471
426.178	17.58562	428.92	12.57413	428.473	17.52504
427.178	16.74705	429.92	12.99563	429.473	17.00565
428.178	15.91405	430.92	13.42089	430.473	16.45147
429.178	15.10708	431.92	13.84928	431.473	15.86475
430.178	14.31783	432.92	14.26938	432.473	15.26524
431.178	13.58296	433.92	14.68231	433.473	14.6762
432.178	12.88201	434.92	15.07767	434.473	14.10426
433.178	12.23157	435.92	15.43817	435.473	13.56
434.178	11.60294	436.92	15.76604	436.473	13.04739
435.178	11.00871	437.92	16.0467	437.473	12.56917
436.178	10.44552	438.92	16.28427	438.473	12.11134
437.178	9.907	439.92	16.46218	439.473	11.6836
438.178	9.37964	440.92	16.58229	440.473	11.28428
439.178	8.85947	441.92	16.63926	441.473	10.89444
440.178	8.3556	442.92	16.63053	442.473	10.50484
441.178	7.84532	443.92	16.5535	443.473	10.13115
442.178	7.34073	444.92	16.40611	444.473	9.74104
443.178	6.84201	445.92	16.18278	445.473	9.35158
444.178	6.34393	446.92	15.88932	446.473	8.94225
445.178	5.85608	447.92	15.5128	447.473	8.52834
446.178	5.3786	448.92	15.06618	448.473	8.09596
447.178	4.91398	449.92	14.54693	449.473	7.64414
448.178	4.47083	450.92	13.96385	450.473	7.17817

449.178	4.04434	451.92	13.31634	451.473	6.70056
450.178	3.63864	452.92	12.63178	452.473	6.22233
451.178	3.28044	453.92	11.90778	453.473	5.74403
452.178	2.96255	454.92	11.18146	454.473	5.2744
453.178	2.68311	455.92	10.4355	455.473	4.80718
454.178	2.44606	456.92	9.71069	456.473	4.36814
455.178	2.24409	457.92	8.99538	457.473	3.94787
456.178	2.0783	458.92	8.30706	458.473	3.55921
457.178	1.9465	459.92	7.66645	459.473	3.21054
458.178	1.84313	460.92	7.05921	460.473	2.9017
459.178	1.76287	461.92	6.48976	461.473	2.63125
460.178	1.70013	462.92	5.9553	462.473	2.40564
461.178	1.65256	463.92	5.46439	463.473	2.2178
462.178	1.61501	464.92	5.00981	464.473	2.06799
463.178	1.58547	465.92	4.59269	465.473	1.94924
464.178	1.56136	466.92	4.20527	466.473	1.85717
465.178	1.54105	467.92	3.86329	467.473	1.7857
466.178	1.52378	468.92	3.54752	468.473	1.73145
467.178	1.50836	469.92	3.27487	469.473	1.69069
468.178	1.49446	470.92	3.02928	470.473	1.65829
469.178	1.48138	471.92	2.8199	471.473	1.63274
470.178	1.46935	472.92	2.64183	472.473	1.61151
471.178	1.45821	473.92	2.49424	473.473	1.5932
472.178	1.44795	474.92	2.37528	474.473	1.57719
473.178	1.43847	475.92	2.28019	475.473	1.5625
474.178	1.4296	476.92	2.20652	476.473	1.54906
475.178	1.42149	477.92	2.15148	477.473	1.53633
476.178	1.41383	478.92	2.11187	478.473	1.52456
477.178	1.40697	479.92	2.08512	479.473	1.51337
478.178	1.4006	480.92	2.06857	480.473	1.50265
479.178	1.39479	481.92	2.06004	481.473	1.49246
480.178	1.38936	482.92	2.05792	482.473	1.4828
481.178	1.38386	483.92	2.06111	483.473	1.47397
482.178	1.3781	484.92	2.06851	484.473	1.46593
483.178	1.37207	485.92	2.07907	485.473	1.45871
484.178	1.36585	486.92	2.09236	486.473	1.45239
485.178	1.3588	487.92	2.1079	487.473	1.44685
486.178	1.35086	488.92	2.12549	488.473	1.44218
487.178	1.34166	489.92	2.1444	489.473	1.43832
488.178	1.33139	490.92	2.16455	490.473	1.43499
489.178	1.31976	491.92	2.18513	491.473	1.4319
490.178	1.30721	492.92	2.2065	492.473	1.42855
491.178	1.29351	493.92	2.22776	493.473	1.42478

492.178	1.27863	494.92	2.24899	494.473	1.42018
493.178	1.26242	495.92	2.27062	495.473	1.41443
494.178	1.24504	496.92	2.29135	496.473	1.40723
495.178	1.22639	497.92	2.3119	497.473	1.39818
496.178	1.20742	498.92	2.33152	498.473	1.38757
497.178	1.18761	499.92	2.3504	499.473	1.37478
498.178	1.16753	500.92	2.36823	500.473	1.3602
499.178	1.14706	501.92	2.38445	501.473	1.34377
500.178	1.1265	502.92	2.39867	502.473	1.32574
501.178	1.10622	503.92	2.4098	503.473	1.30608
502.178	1.08574	504.92	2.41749	504.473	1.28512
503.178	1.06542	505.92	2.42048	505.473	1.26255
504.178	1.0455	506.92	2.41816	506.473	1.23927
505.178	1.02552	507.92	2.40981	507.473	1.21488
506.178	1.00552	508.92	2.39476	508.473	1.1899
507.178	0.98567	509.92	2.37231	509.473	1.16472
508.178	0.96574	510.92	2.34119	510.473	1.13929
509.178	0.94627	511.92	2.30274	511.473	1.1137
510.178	0.92716	512.92	2.25587	512.473	1.08819
511.178	0.90785	513.92	2.20184	513.473	1.06309
512.178	0.88912	514.92	2.14221	514.473	1.03778
513.178	0.87067	515.92	2.07679	515.473	1.01234
514.178	0.85194	516.92	2.00628	516.473	0.98724
515.178	0.83359	517.92	1.93425	517.473	0.96207
516.178	0.81543	518.92	1.85958	518.473	0.93676
517.178	0.79701	519.92	1.78445	519.473	0.91094
518.178	0.77885	520.92	1.71032	520.473	0.88475
519.178	0.7605	521.92	1.63652	521.473	0.85896
520.178	0.74155	522.92	1.56555	522.473	0.83257
521.178	0.7227	523.92	1.4967	523.473	0.80622
522.178	0.70387	524.92	1.42894	524.473	0.77891
523.178	0.68454	525.92	1.36414	525.473	0.75219
524.178	0.66509	526.92	1.30216	526.473	0.72537
525.178	0.64543	527.92	1.24172	527.473	0.69857
526.178	0.62563	528.92	1.18375	528.473	0.6717
527.178	0.60569	529.92	1.12814	529.473	0.64503
528.178	0.58574	530.92	1.07345	530.473	0.6183
529.178	0.5657	531.92	1.02013	531.473	0.59204
530.178	0.54505	532.92	0.96867	532.473	0.56619
531.178	0.52467	533.92	0.91779	533.473	0.54126
532.178	0.50454	534.92	0.86728	534.473	0.5165
533.178	0.48402	535.92	0.81714	535.473	0.49225
534.178	0.46353	536.92	0.76803	536.473	0.46912

535.178	0.44287	537.92	0.71825	537.473	0.4458
536.178	0.42221	538.92	0.66926	538.473	0.42368
537.178	0.4018	539.92	0.62033	539.473	0.40215
538.178	0.3814	540.92	0.57258	540.473	0.38134
539.178	0.36101	541.92	0.52582	541.473	0.36146
540.178	0.34106	542.92	0.47959	542.473	0.3422
541.178	0.32123	543.92	0.43452	543.473	0.32373
542.178	0.30198	544.92	0.39132	544.473	0.30624
543.178	0.28315	545.92	0.34936	545.473	0.28919
544.178	0.26435	546.92	0.31054	546.473	0.27311
545.178	0.24621	547.92	0.27334	547.473	0.25792
546.178	0.22857	548.92	0.23999	548.473	0.24317
547.178	0.21153	549.92	0.20903	549.473	0.22921
548.178	0.1947	550.92	0.18113	550.473	0.21586
549.178	0.17832	551.92	0.15642	551.473	0.20312
550.178	0.16221	552.92	0.13479	552.473	0.19143
551.178	0.14635	553.92	0.11619	553.473	0.18036
552.178	0.1312	554.92	0.09997382	554.473	0.16978
553.178	0.11671	555.92	0.08650044	555.473	0.15987
554.178	0.10266	556.92	0.07503123	556.473	0.15041
555.178	0.0890555	557.92	0.06534744	557.473	0.14155
556.178	0.07644855	558.92	0.05693498	558.473	0.13322
557.178	0.06399772	559.92	0.04986654	559.473	0.12518
558.178	0.05247306	560.92	0.04357394	560.473	0.11741
559.178	0.04124294	561.92	0.03809655	561.473	0.10992
560.178	0.03063075	562.92	0.03337574	562.473	0.10292
561.178	0.02054594	563.92	0.02906887	563.473	0.09647834
562.178	0.01108987	564.92	0.02499872	564.473	0.09020579
563.178	0.002197617	565.92	0.02133533	565.473	0.08431454
564.178	-0.00632052	566.92	0.01772285	566.473	0.07865329
565.178	-0.01429066	567.92	0.01429633	567.473	0.07334605
566.178	-0.02201354	568.92	0.01109958	568.473	0.06837705
567.178	-0.02924854	569.92	0.008086258	569.473	0.06373381
568.178	-0.03600675	570.92	0.005240372	570.473	0.05928824
569.178	-0.04241261	571.92	0.002362138	571.473	0.05503139
570.178	-0.04856487	572.92	-0.000301923	572.473	0.05099663
571.178	-0.05429911	573.92	-0.003025998	573.473	0.04712548
572.178	-0.05989157	574.92	-0.005656576	574.473	0.04351729
573.178	-0.0650051	575.92	-0.008116424	575.473	0.04010132
574.178	-0.07000155	576.92	-0.01040774	576.473	0.03703083
575.178	-0.07479374	577.92	-0.01250688	577.473	0.0342916
576.178	-0.07922893	578.92	-0.01465675	578.473	0.03178654
577.178	-8.35E-02	579.92	-0.01666422	579.473	0.02933031

578.178	-0.08744509	580.92	-0.01847188	580.473	0.02701928
579.178	-0.09120257	581.92	-0.02019399	581.473	0.02491657
580.178	-0.09473183	582.92	-0.0219258	582.473	0.02287638
581.178	-0.09800934	583.92	-0.02368839	583.473	0.02087732
582.178	-0.101	584.92	-0.02554216	584.473	0.01888007
583.178	-1.04E-01	585.92	-0.02739964	585.473	0.01683843
584.178	-0.10675	586.92	-0.02926557	586.473	0.01474266
585.178	-0.10949	587.92	-0.03103086	587.473	0.01298212
586.178	-0.11208	588.92	-0.03287083	588.473	0.01135426
587.178	-0.11456	589.92	-0.03473598	589.473	0.009809884
588.178	-0.1169	590.92	-0.0364402	590.473	0.008363905
589.178	-0.11912	591.92	-0.03796412	591.473	0.007073724
590.178	-0.1213	592.92	-0.0392309	592.473	0.006011946
591.178	-0.12316	593.92	-0.0404995	593.473	0.00491662
592.178	-0.1248	594.92	-0.04170391	594.473	0.003925142
593.178	-0.12619	595.92	-0.04289002	595.473	0.002782077
594.178	-0.12765	596.92	-0.04406197	596.473	0.00167011
595.178	-0.12902	597.92	-0.04517469	597.473	0.000502246
596.178	-0.13032	598.92	-0.04626323	598.473	-0.000563004
597.178	-0.13164	599.92	-0.04728768	599.473	-0.001672855
598.178	-0.13283	600.92	-0.04839473	600.473	-0.002945974
599.178	-0.13403	601.92	-0.04926912	601.473	-0.004017238
600.178	-0.13529	602.92	-0.05013798	602.473	-0.005029718
601.178	-0.13665	603.92	-0.05108768	603.473	-6.05E-03
602.178	-0.13791	604.92	-0.05191181	604.473	-0.007153037
603.178	-0.13918	605.92	-0.05271547	605.473	-0.00823267
604.178	-0.14055	606.92	-0.05330416	606.473	-0.009352281
605.178	-0.14189	607.92	-0.05372257	607.473	-0.01016917
606.178	-0.14324	608.92	-0.05423706	608.473	-0.01086119
607.178	-0.14442	609.92	-0.05500698	609.473	-0.0117511
608.178	-0.14539	610.92	-0.05570643	610.473	-0.01274976
609.178	-0.14642	611.92	-0.05621203	611.473	-0.01370407
610.178	-0.1476	612.92	-0.05656897	612.473	-0.014328
611.178	-0.14862	613.92	-0.05689033	613.473	-0.01486398
612.178	-0.14941	614.92	-0.05741221	614.473	-0.01545324
613.178	-0.15015	615.92	-0.05788985	615.473	-0.01621983
614.178	-0.15095	616.92	-0.05809152	616.473	-0.01694781
615.178	-0.15191	617.92	-0.05822759	617.473	-0.01751132
616.178	-0.15285	618.92	-0.0585207	618.473	-0.01820253
617.178	-0.15356	619.92	-5.89E-02	619.473	-0.01896581
618.178	-0.15422	620.92	-5.93E-02	620.473	-0.01971676
619.178	-0.1551	621.92	-0.05967029	621.473	-0.0204822
620.178	-0.15593	622.92	-0.05993933	622.473	-0.0211338

621.178	-0.15671	623.92	-0.06018688	623.473	-0.0217001
622.178	-0.15748	624.92	-0.06065992	624.473	-0.02239854
623.178	-0.15804	625.92	-0.06104605	625.473	-0.02315189
624.178	-0.15869	626.92	-0.06121871	626.473	-0.02366514
625.178	-0.1594	627.92	-0.06132746	627.473	-0.0240681
626.178	-0.15991	628.92	-0.06144931	628.473	-0.02449478
627.178	-0.16037	629.92	-0.06145023	629.473	-0.0247766
628.178	-0.16083	630.92	-0.06131017	630.473	-0.02495915
629.178	-0.16115	631.92	-0.06142045	631.473	-0.02521305
630.178	-0.16135	632.92	-0.06154816	632.473	-0.02550133
631.178	-0.16162	633.92	-0.06162306	633.473	-0.02577794
632.178	-0.16204	634.92	-0.06163483	634.473	-0.02598991
633.178	-0.16252	635.92	-0.06158195	635.473	-0.02606814
634.178	-0.16289	636.92	-0.06153935	636.473	-0.02618896
635.178	-0.16309	637.92	-0.06147371	637.473	-0.02625543
636.178	-0.16322	638.92	-6.15E-02	638.473	-0.02638285
637.178	-0.16345	639.92	-6.15E-02	639.473	-0.02644534
638.178	-0.1636	640.92	-0.06142762	640.473	-0.02645135
639.178	-1.64E-01	641.92	-0.06132331	641.473	-0.02641008
640.178	-0.16384	642.92	-0.0613602	642.473	-0.02636892
641.178	-0.16388	643.92	-0.06142998	643.473	-0.02661659
642.178	-0.16398	644.92	-0.06124699	644.473	-0.02671117
643.178	-0.16411	645.92	-0.06120765	645.473	-0.02683379
644.178	-0.16412	646.92	-0.0614385	646.473	-0.02728143
645.178	-0.16403	647.92	-0.06148185	647.473	-0.02795473
646.178	-0.16425	648.92	-0.06157946	648.473	-0.02846898
647.178	-0.16463	649.92	-0.06158794	649.473	-0.02884711
648.178	-0.1648	650.92	-6.15E-02	650.473	-0.02910823
649.178	-0.16485	651.92	-0.06138171	651.473	-0.02923276
650.178	-0.16475	652.92	-0.06149436	652.473	-0.02945964
651.178	-0.16472	653.92	-0.06137342	653.473	-0.02966287
652.178	-0.16477	654.92	-0.06099258	654.473	-0.029646
653.178	-0.16484	655.92	-0.06091977	655.473	-0.02950195
654.178	-0.16464	656.92	-0.06081179	656.473	-0.02953533
655.178	-0.1642	657.92	-0.06060821	657.473	-0.02955878
656.178	-0.16396	658.92	-0.06038957	658.473	-0.02964366
657.178	-0.16377	659.92	-0.06022635	659.473	-0.02957099
658.178	-0.16355	660.92	-0.0602288	660.473	-0.02959181
659.178	-0.16341	661.92	-0.06038463	661.473	-0.02980273
660.178	-0.16338	662.92	-0.06046981	662.473	-0.0299165
661.178	-0.16355	663.92	-6.01E-02	663.473	-0.02987242
662.178	-0.1638	664.92	-0.05958921	664.473	-0.02958396
663.178	-0.164	665.92	-0.0591016	665.473	-0.02905788

664.178	-0.16383	666.92	-0.05865445	666.473	-0.02855466
665.178	-0.16352	667.92	-0.05821719	667.473	-0.02819403
666.178	-0.16325	668.92	-0.05763952	668.473	-0.02794347
667.178	-0.16295	669.92	-0.05709174	669.473	-0.0276419
668.178	-0.1625	670.92	-0.05648904	670.473	-0.02725698
669.178	-0.16204	671.92	-0.05600255	671.473	-0.02684547
670.178	-0.16152	672.92	-0.05570802	672.473	-0.02649992
671.178	-0.16109	673.92	-0.05542096	673.473	-0.0266098
672.178	-0.16094	674.92	-0.05484517	674.473	-0.02667141
673.178	-0.16109	675.92	-0.05398816	675.473	-0.02646138
674.178	-0.16125	676.92	-0.0530787	676.473	-0.0259815
675.178	-0.16112	677.92	-0.05212293	677.473	-0.02547575
676.178	-0.16078	678.92	-0.05129566	678.473	-2.51E-02
677.178	-0.16042	679.92	-0.05043849	679.473	-0.02473626
678.178	-0.16007	680.92	-0.04950406	680.473	-0.02442462
679.178	-0.15971	681.92	-0.0484368	681.473	-0.02386305
680.178	-0.15925	682.92	-0.04758344	682.473	-0.02322735
681.178	-0.15856	683.92	-0.04686752	683.473	-0.02279706
682.178	-0.15774	684.92	-0.04622394	684.473	-0.02234438
683.178	-0.15703	685.92	-0.04561569	685.473	-0.02202778
684.178	-0.15642	686.92	-0.04492145	686.473	-0.02173227
685.178	-0.15594	687.92	-0.04408669	687.473	-0.02130774
686.178	-0.15552	688.92	-0.04314996	688.473	-0.02073639
687.178	-0.15507	689.92	-0.04224842	689.473	-0.02011359
688.178	-0.15447	690.92	-0.04154714	690.473	-0.01969594
689.178	-0.15396	691.92	-0.04104671	691.473	-1.93E-02
690.178	-0.15338	692.92	-0.04046499	692.473	-0.01904989
691.178	-0.15304	693.92	-3.97E-02	693.473	-0.01871473
692.178	-0.15282	694.92	-0.03888229	694.473	-0.01804256
693.178	-0.15253	695.92	-0.03819358	695.473	-0.01752113
694.178	-1.52E-01	696.92	-0.03760728	696.473	-0.01716932
695.178	-0.15154	697.92	-0.03693583	697.473	-0.01704714
696.178	-0.15116	698.92	-0.03607778	698.473	-0.01666283
697.178	-0.15087	699.92	-0.03496434	699.473	-0.01625702
698.178	-0.15064	700.92	-0.03380019	700.473	-0.0156848
699.178	-0.15036	701.92	-0.0327261	701.473	-0.01510613
700.178	-0.14979	702.92	-0.03186284	702.473	-0.01482426
701.178	-0.14911	703.92	-3.09E-02	703.473	-0.01449345
702.178	-0.14867	704.92	-0.02988707	704.473	-0.01398753
703.178	-0.14828	705.92	-0.02886263	705.473	-0.01329655
704.178	-0.14772	706.92	-0.02791032	706.473	-0.01278276
705.178	-0.14702	707.92	-0.0270449	707.473	-0.01227013
706.178	-0.14641	708.92	-0.02610987	708.473	-0.01197533

707.178	-0.14574	709.92	-0.02504959	709.473	-0.01144252
708.178	-0.14529	710.92	-0.02379021	710.473	-0.01060714
709.178	-0.1448	711.92	-0.02253752	711.473	-0.009676342
710.178	-0.14404	712.92	-0.02126039	712.473	-0.008934753
711.178	-0.14308	713.92	-0.02007511	713.473	-0.008325343
712.178	-0.14229	714.92	-0.0189112	714.473	-0.007733058
713.178	-0.14159	715.92	-0.01759996	715.473	-0.007016647
714.178	-0.1409	716.92	-0.01637137	716.473	-0.006178741
715.178	-0.14008	717.92	-0.01507807	717.473	-0.005473336
716.178	-0.13905	718.92	-0.01380993	718.473	-0.004876515
717.178	-0.13807	719.92	-0.01241641	719.473	-0.004256283
718.178	-0.13712	720.92	-0.01104864	720.473	-0.003574339
719.178	-0.13619	721.92	-0.009754266	721.473	-0.002765763
720.178	-0.13523	722.92	-0.008483383	722.473	-0.001995425
721.178	-0.13421	723.92	-0.00733137	723.473	-0.00136322
722.178	-0.13334	724.92	-0.006084914	724.473	-0.000681199
723.178	-1.33E-01	725.92	-0.005019023	725.473	4.92215E-05
724.178	-0.13191	726.92	-0.00386131	726.473	0.000795077
725.178	-0.1312	727.92	-0.002909773	727.473	0.00142999
726.178	-0.13062	728.92	-0.00193573	728.473	0.001902173
727.178	-0.13012	729.92	-0.000759563	729.473	0.002445746
728.178	-0.1297	730.92	0.000697578	730.473	0.003209975
729.178	-0.12918	731.92	0.002186937	731.473	0.004096886
730.178	-0.12835	732.92	0.003563732	732.473	0.004855354
731.178	-0.12758	733.92	0.004974437	733.473	0.005586771
732.178	-0.1268	734.92	0.006254584	734.473	0.0061851
733.178	-0.12603	735.92	0.007713513	735.473	0.006828289
734.178	-0.12525	736.92	0.009084011	736.473	0.007695003
735.178	-0.12442	737.92	0.01038513	737.473	8.48E-03
736.178	-1.23E-01	738.92	0.01146793	738.473	0.009197659
737.178	-0.12265	739.92	0.01269013	739.473	9.85E-03
738.178	-0.12189	740.92	0.01409104	740.473	0.01063289
739.178	-0.12109	741.92	0.01547881	741.473	0.01146402
740.178	-0.12019	742.92	0.01685459	742.473	0.01235617
741.178	-0.11933	743.92	1.80E-02	743.473	0.01313502
742.178	-0.11844	744.92	0.01929084	744.473	0.01382475
743.178	-0.11769	745.92	0.0204138	745.473	0.01446679
744.178	-0.11707	746.92	0.02163161	746.473	0.01504764
745.178	-0.11635	747.92	0.0227842	747.473	0.01569188
746.178	-0.11575	748.92	0.0238676	748.473	0.01643211
747.178	-0.11515	749.92	0.0250475	749.473	1.72E-02
748.178	-1.15E-01	750.92	0.02636935	750.473	0.01799409
749.178	-0.11382	751.92	0.02793719	751.473	0.01889313

750.178	-0.11305	752.92	0.02936469	752.473	0.01969848
751.178	-0.11213	753.92	0.03102414	753.473	0.02066905
752.178	-0.11122	754.92	0.03263491	754.473	0.02174375
753.178	-0.11033	755.92	0.0343319	755.473	0.02276234
754.178	-0.10931	756.92	0.0361778	756.473	0.02375837
755.178	-0.10831	757.92	0.03776859	757.473	0.02466222
756.178	-0.10712	758.92	0.03913945	758.473	0.02549028
757.178	-0.10618	759.92	0.04053974	759.473	0.02640783
758.178	-0.10544	760.92	0.04204283	760.473	0.02748798
759.178	-0.10484	761.92	0.04351541	761.473	0.02858195
760.178	-0.10402	762.92	0.04492784	762.473	0.02956048
761.178	-0.10308	763.92	0.04633932	763.473	0.03044273
762.178	-1.02E-01	764.92	0.04770433	764.473	0.03127837
763.178	-1.01E-01	765.92	0.04930251	765.473	0.03224853
764.178	-1.00E-01	766.92	0.0510253	766.473	0.03333969
765.178	-9.96E-02	767.92	0.05263047	767.473	0.03427644
766.178	-0.09854703	768.92	0.05408306	768.473	0.03530038
767.178	-0.09731988	769.92	0.05538764	769.473	0.036211
768.178	-0.09618799	770.92	0.05689917	770.473	0.0372098
769.178	-9.50E-02	771.92	0.05841185	771.473	0.03821147
770.178	-0.09394083	772.92	0.05978837	772.473	0.03910467
771.178	-0.0928998	773.92	6.14E-02	773.473	0.03999682
772.178	-0.09183828	774.92	6.32E-02	774.473	0.04113662
773.178	-0.09083753	775.92	0.06505583	775.473	0.04233666
774.178	-8.94E-02	776.92	0.06710121	776.473	0.04347371
775.178	-0.08803574	777.92	0.06922961	777.473	0.04477318
776.178	-0.08680786	778.92	0.07124593	778.473	0.04601338
777.178	-0.08554099	779.92	0.07323298	779.473	0.04732417
778.178	-0.08424424	780.92	0.07546674	780.473	0.04883606
779.178	-0.08302076	781.92	0.07739885	781.473	0.05018531
780.178	-0.08180553	782.92	0.07918872	782.473	0.05130159
781.178	-0.08050515	783.92	0.08095625	783.473	0.05230985
782.178	-0.07957875	784.92	0.08276366	784.473	0.05332736
783.178	-0.07872614	785.92	0.08477506	785.473	0.05439142
784.178	-0.07784524	786.92	0.08689039	786.473	0.05563455
785.178	-0.07687366	787.92	0.08918805	787.473	0.05698653
786.178	-0.07574467	788.92	0.09143789	788.473	0.05819693
787.178	-0.07452691	789.92	0.09370778	789.473	0.05943227
788.178	-0.07317796	790.92	0.09601988	790.473	0.06071664
789.178	-0.07188465	791.92	0.09852154	791.473	0.06208782
790.178	-0.07058897	792.92	0.10095	792.473	0.06347904
791.178	-0.06919307	793.92	0.1031	793.473	6.46E-02
792.178	-0.06763972	794.92	0.10527	794.473	0.06563882

793.178	-0.06625367	795.92	0.10732	795.473	0.06669937
794.178	-0.06505684	796.92	0.10886	796.473	0.06765549
795.178	-0.06385747				
796.178	-0.0625541				
797.178	-0.06239067				

temperature	*DSC*	*temperature*	*DSC*
°C	mW/mg	°C	mW/mg
	Coal+TPPI		Coal+PA
25.93	0.01523975	25.697	-0.04451909
26.93	-0.03698369	26.697	-0.06271276
27.93	-0.08044707	27.697	-0.09116381
28.93	-0.11601	28.697	-0.11983
29.93	-0.13743	29.697	-0.14014
30.93	-0.1474	30.697	-0.15154
31.93	-0.15142	31.697	-0.15747
32.93	-0.15272	32.697	-0.16103
33.93	-0.15304	33.697	-0.16345
34.93	-0.15309	34.697	-0.16541
35.93	-0.15306	35.697	-0.16723
36.93	-0.15309	36.697	-0.16886
37.93	-0.15321	37.697	-0.17057
38.93	-0.15345	38.697	-0.17223
39.93	-0.15378	39.697	-0.17393
40.93	-0.15418	40.697	-0.17564
41.93	-0.15457	41.697	-0.17736
42.93	-0.15496	42.697	-0.179
43.93	-0.15532	43.697	-0.18064
44.93	-0.15564	44.697	-0.18221
45.93	-0.15589	45.697	-0.18364
46.93	-0.15601	46.697	-0.18494
47.93	-0.15602	47.697	-0.18613
48.93	-0.15588	48.697	-0.18719
49.93	-0.1556	49.697	-0.18812
50.93	-0.1552	50.697	-0.18882
51.93	-0.15468	51.697	-0.18942
52.93	-0.15393	52.697	-0.18975
53.93	-0.15293	53.697	-0.18982
54.93	-0.15177	54.697	-0.18966
55.93	-0.15038	55.697	-0.18932
56.93	-0.14877	56.697	-0.18864
57.93	-0.14695	57.697	-0.18776
58.93	-0.14482	58.697	-0.18657
59.93	-0.14247	59.697	-0.18505
60.93	-0.13982	60.697	-0.18317
61.93	-0.13689	61.697	-0.18105
62.93	-0.13379	62.697	-0.17854
63.93	-0.13038	63.697	-0.17573
64.93	-0.1267	64.697	-0.17263

65.93	-0.12274	65.697	-0.1692
66.93	-0.11866	66.697	-0.16554
67.93	-0.11434	67.697	-0.16148
68.93	-0.10977	68.697	-0.15705
69.93	-0.10507	69.697	-0.15238
70.93	-0.10021	70.697	-0.14746
71.93	-0.09505946	71.697	-0.14229
72.93	-0.08981203	72.697	-0.13685
73.93	-0.08447912	73.697	-0.13122
74.93	-0.07899895	74.697	-0.12544
75.93	-0.07336855	75.697	-0.11926
76.93	-0.06760378	76.697	-0.1131
77.93	-0.06187502	77.697	-0.1067
78.93	-0.0560205	78.697	-0.10028
79.93	-0.05009043	79.697	-0.09370806
80.93	-0.04406956	80.697	-0.08710518
81.93	-0.03821252	81.697	-0.08051844
82.93	-0.03219463	82.697	-0.07384492
83.93	-0.02615913	83.697	-0.06714576
84.93	-0.02013904	84.697	-0.06055195
85.93	-0.01415618	85.697	-0.0538762
86.93	-0.008309627	86.697	-0.04727911
87.93	-0.002404121	87.697	-0.04067329
88.93	0.003411934	88.697	-0.0341257
89.93	0.009291254	89.697	-0.02758586
90.93	0.01495976	90.697	-0.02117585
91.93	0.02075746	91.697	-0.01476352
92.93	0.0264714	92.697	-0.00833663
93.93	0.03187018	93.697	-0.00213264
94.93	0.03747087	94.697	0.00417614
95.93	0.04296687	95.697	0.01044209
96.93	0.04842511	96.697	0.01664736
97.93	0.053744	97.697	0.02272126
98.93	0.05901674	98.697	0.02859955
99.93	0.06428822	99.697	0.03473644
100.93	0.06953333	100.697	0.04044942
101.93	0.07482258	101.697	0.04635743
102.93	0.08000809	102.697	0.05220843
103.93	0.08518679	103.697	0.05797614
104.93	0.09039274	104.697	0.06382151
105.93	0.09533425	105.697	0.06961853
106.93	0.10047	106.697	0.07535917
107.93	0.10559	107.697	0.08102909

108.93	0.11073	108.697	0.08684027
109.93	0.11575	109.697	0.09263546
110.93	0.1209	110.697	0.09844064
111.93	0.12595	111.697	0.10416
112.93	0.13098	112.697	0.11013
113.93	0.13607	113.697	0.11601
114.93	0.14133	114.697	0.12199
115.93	0.14662	115.697	0.12802
116.93	0.15195	116.697	0.13409
117.93	0.1573	117.697	0.14009
118.93	0.1626	118.697	0.14627
119.93	0.16806	119.697	0.15248
120.93	0.17366	120.697	0.1587
121.93	0.17919	121.697	0.16503
122.93	0.18482	122.697	0.17136
123.93	0.19045	123.697	0.17776
124.93	0.19604	124.697	0.18421
125.93	0.20183	125.697	0.19073
126.93	0.20763	126.697	0.19748
127.93	0.21365	127.697	0.2043
128.93	0.21964	128.697	0.21122
129.93	0.22587	129.697	0.21812
130.93	0.23203	130.697	0.22518
131.93	0.23837	131.697	0.23246
132.93	0.24479	132.697	0.23962
133.93	0.25135	133.697	0.2471
134.93	0.25801	134.697	0.25487
135.93	0.26478	135.697	0.26235
136.93	0.27167	136.697	0.26998
137.93	0.27846	137.697	0.2779
138.93	0.28557	138.697	0.28599
139.93	0.29293	139.697	0.29403
140.93	0.30023	140.697	0.3023
141.93	0.30771	141.697	0.3107
142.93	0.31505	142.697	0.31915
143.93	0.32276	143.697	0.32773
144.93	0.33043	144.697	0.33645
145.93	0.33844	145.697	0.34538
146.93	0.34661	146.697	0.35448
147.93	0.35474	147.697	0.36353
148.93	0.36305	148.697	0.37291
149.93	0.37155	149.697	0.3822
150.93	0.38018	150.697	0.39182

151.93	0.389	151.697	0.40171
152.93	0.39801	152.697	0.41153
153.93	0.40725	153.697	0.42158
154.93	0.41636	154.697	0.43167
155.93	0.42556	155.697	0.44175
156.93	0.43514	156.697	0.45243
157.93	0.44484	157.697	0.46292
158.93	0.45455	158.697	0.4737
159.93	0.46452	159.697	0.48442
160.93	0.47467	160.697	0.49549
161.93	0.48486	161.697	0.50648
162.93	0.49545	162.697	0.51782
163.93	0.50601	163.697	0.52917
164.93	0.51682	164.697	0.54094
165.93	0.5278	165.697	0.5525
166.93	0.53899	166.697	0.56445
167.93	0.55013	167.697	0.5763
168.93	0.56138	168.697	0.58853
169.93	0.57306	169.697	0.60075
170.93	0.58459	170.697	0.61312
171.93	0.59644	171.697	0.62577
172.93	0.60821	172.697	0.63857
173.93	0.62036	173.697	0.65108
174.93	0.63259	174.697	0.66408
175.93	0.64491	175.697	0.67721
176.93	0.65752	176.697	0.69034
177.93	0.67042	177.697	0.70367
178.93	0.68323	178.697	0.71712
179.93	0.69647	179.697	0.73092
180.93	0.70961	180.697	0.74468
181.93	0.72296	181.697	0.7588
182.93	0.73659	182.697	0.77263
183.93	0.75045	183.697	0.78711
184.93	0.76418	184.697	0.80151
185.93	0.77788	185.697	0.816
186.93	0.79197	186.697	0.83089
187.93	0.8063	187.697	0.84585
188.93	0.8207	188.697	0.86098
189.93	0.83538	189.697	0.87643
190.93	0.84992	190.697	0.89179
191.93	0.86487	191.697	0.90735
192.93	0.88017	192.697	0.923
193.93	0.89569	193.697	0.93888

194.93	0.91118	194.697	0.95469
195.93	0.92696	195.697	0.97068
196.93	0.94273	196.697	0.98691
197.93	0.95903	197.697	1.00324
198.93	0.97543	198.697	1.01992
199.93	0.99168	199.697	1.03648
200.93	1.00836	200.697	1.05326
201.93	1.02525	201.697	1.07017
202.93	1.0424	202.697	1.08741
203.93	1.0595	203.697	1.1048
204.93	1.07713	204.697	1.12251
205.93	1.09459	205.697	1.14032
206.93	1.11241	206.697	1.15811
207.93	1.13027	207.697	1.17617
208.93	1.14861	208.697	1.19428
209.93	1.16721	209.697	1.21295
210.93	1.18624	210.697	1.23143
211.93	1.20523	211.697	1.2502
212.93	1.22464	212.697	1.26875
213.93	1.24453	213.697	1.28803
214.93	1.2646	214.697	1.30718
215.93	1.28481	215.697	1.32636
216.93	1.30566	216.697	1.34603
217.93	1.32626	217.697	1.36562
218.93	1.34757	218.697	1.38544
219.93	1.36848	219.697	1.40561
220.93	1.38957	220.697	1.42562
221.93	1.41098	221.697	1.44607
222.93	1.43234	222.697	1.46686
223.93	1.45437	223.697	1.48812
224.93	1.47623	224.697	1.50986
225.93	1.49867	225.697	1.53142
226.93	1.52129	226.697	1.55336
227.93	1.54437	227.697	1.5754
228.93	1.5677	228.697	1.59772
229.93	1.59136	229.697	1.62004
230.93	1.61518	230.697	1.64279
231.93	1.63944	231.697	1.66532
232.93	1.66378	232.697	1.6884
233.93	1.6887	233.697	1.71146
234.93	1.71382	234.697	1.7346
235.93	1.73938	235.697	1.75857
236.93	1.76512	236.697	1.78288

237.93	1.79188	237.697	1.8074
238.93	1.81796	238.697	1.83239
239.93	1.84507	239.697	1.8573
240.93	1.87225	240.697	1.88308
241.93	1.89956	241.697	1.90885
242.93	1.92729	242.697	1.93443
243.93	1.95507	243.697	1.96026
244.93	1.98287	244.697	1.98648
245.93	2.01131	245.697	2.01285
246.93	2.03962	246.697	2.03952
247.93	2.06831	247.697	2.06605
248.93	2.09696	248.697	2.09351
249.93	2.12639	249.697	2.12123
250.93	2.15561	250.697	2.14912
251.93	2.18455	251.697	2.17745
252.93	2.21412	252.697	2.20614
253.93	2.24401	253.697	2.23467
254.93	2.27404	254.697	2.26335
255.93	2.30413	255.697	2.29236
256.93	2.3345	256.697	2.32172
257.93	2.36565	257.697	2.35083
258.93	2.3966	258.697	2.37964
259.93	2.42866	259.697	2.40909
260.93	2.46052	260.697	2.43801
261.93	2.49283	261.697	2.46801
262.93	2.52474	262.697	2.49776
263.93	2.55688	263.697	2.52842
264.93	2.58952	264.697	2.55899
265.93	2.62182	265.697	2.58999
266.93	2.65434	266.697	2.62148
267.93	2.68649	267.697	2.65319
268.93	2.71842	268.697	2.68489
269.93	2.7513	269.697	2.71722
270.93	2.78398	270.697	2.74935
271.93	2.81662	271.697	2.78081
272.93	2.84874	272.697	2.813
273.93	2.88099	273.697	2.84469
274.93	2.91345	274.697	2.87652
275.93	2.9455	275.697	2.90902
276.93	2.97832	276.697	2.94125
277.93	3.01059	277.697	2.97378
278.93	3.04299	278.697	3.00633
279.93	3.07491	279.697	3.03875

280.93	3.10697	280.697	3.07102
281.93	3.13867	281.697	3.10379
282.93	3.16971	282.697	3.13573
283.93	3.20068	283.697	3.16798
284.93	3.23149	284.697	3.20003
285.93	3.26178	285.697	3.2318
286.93	3.29271	286.697	3.26362
287.93	3.32344	287.697	3.29412
288.93	3.35434	288.697	3.32479
289.93	3.38563	289.697	3.35544
290.93	3.41709	290.697	3.3857
291.93	3.44827	291.697	3.41658
292.93	3.47855	292.697	3.44665
293.93	3.50839	293.697	3.47706
294.93	3.53725	294.697	3.50727
295.93	3.56626	295.697	3.53752
296.93	3.59412	296.697	3.56699
297.93	3.62079	297.697	3.59636
298.93	3.64606	298.697	3.62532
299.93	3.6709	299.697	3.65348
300.93	3.69498	300.697	3.68116
301.93	3.7185	301.697	3.70777
302.93	3.74223	302.697	3.7343
303.93	3.76504	303.697	3.76026
304.93	3.78755	304.697	3.78631
305.93	3.8102	305.697	3.81183
306.93	3.83278	306.697	3.83663
307.93	3.85466	307.697	3.86183
308.93	3.87584	308.697	3.88659
309.93	3.8961	309.697	3.91086
310.93	3.91573	310.697	3.93516
311.93	3.93482	311.697	3.95871
312.93	3.95382	312.697	3.98192
313.93	3.97145	313.697	4.00425
314.93	3.98815	314.697	4.02562
315.93	4.00488	315.697	4.04668
316.93	4.0216	316.697	4.06672
317.93	4.0384	317.697	4.08626
318.93	4.05449	318.697	4.1046
319.93	4.06964	319.697	4.12256
320.93	4.08394	320.697	4.14015
321.93	4.09764	321.697	4.15736
322.93	4.11041	322.697	4.17455

323.93	4.12296	323.697	4.19106
324.93	4.135	324.697	4.20735
325.93	4.14643	325.697	4.22316
326.93	4.15774	326.697	4.23892
327.93	4.16906	327.697	4.25492
328.93	4.18052	328.697	4.2702
329.93	4.1922	329.697	4.28515
330.93	4.20337	330.697	4.29985
331.93	4.21415	331.697	4.31447
332.93	4.22437	332.697	4.32894
333.93	4.23343	333.697	4.34366
334.93	4.2424	334.697	4.35835
335.93	4.25108	335.697	4.37257
336.93	4.26015	336.697	4.3872
337.93	4.2696	337.697	4.4023
338.93	4.27956	338.697	4.41799
339.93	4.29025	339.697	4.43386
340.93	4.30223	340.697	4.44988
341.93	4.31519	341.697	4.46614
342.93	4.32844	342.697	4.48269
343.93	4.3413	343.697	4.49947
344.93	4.35387	344.697	4.51622
345.93	4.36667	345.697	4.53289
346.93	4.38083	346.697	4.5499
347.93	4.3951	347.697	4.56718
348.93	4.40941	348.697	4.58506
349.93	4.42313	349.697	4.60406
350.93	4.43682	350.697	4.62437
351.93	4.45114	351.697	4.64601
352.93	4.46655	352.697	4.66908
353.93	4.48279	353.697	4.69357
354.93	4.49892	354.697	4.71942
355.93	4.51632	355.697	4.74626
356.93	4.53405	356.697	4.77399
357.93	4.55308	357.697	4.80207
358.93	4.57352	358.697	4.83076
359.93	4.59502	359.697	4.86016
360.93	4.61868	360.697	4.89045
361.93	4.64329	361.697	4.92207
362.93	4.669	362.697	4.95493
363.93	4.69522	363.697	4.98862
364.93	4.72232	364.697	5.02308
365.93	4.74979	365.697	5.05951

366.93	4.77774	366.697	5.09697
367.93	4.80536	367.697	5.13574
368.93	4.83357	368.697	5.17503
369.93	4.86103	369.697	5.21639
370.93	4.88993	370.697	5.2584
371.93	4.92089	371.697	5.30207
372.93	4.95255	372.697	5.34739
373.93	4.98498	373.697	5.39445
374.93	5.0194	374.697	5.44331
375.93	5.05556	375.697	5.49308
376.93	5.09443	376.697	5.54427
377.93	5.1347	377.697	5.59876
378.93	5.17586	378.697	5.65405
379.93	5.2172	379.697	5.71193
380.93	5.25884	380.697	5.77039
381.93	5.30199	381.697	5.83168
382.93	5.34601	382.697	5.89583
383.93	5.39005	383.697	5.96129
384.93	5.43441	384.697	6.02967
385.93	5.4796	385.697	6.10066
386.93	5.52624	386.697	6.17479
387.93	5.57409	387.697	6.25149
388.93	5.62462	388.697	6.33262
389.93	5.67675	389.697	6.41585
390.93	5.73194	390.697	6.50223
391.93	5.78906	391.697	6.59155
392.93	5.84779	392.697	6.68329
393.93	5.90809	393.697	6.77802
394.93	5.96991	394.697	6.87586
395.93	6.03402	395.697	6.9779
396.93	6.09987	396.697	7.08404
397.93	6.16737	397.697	7.19374
398.93	6.23627	398.697	7.30805
399.93	6.30688	399.697	7.42661
400.93	6.37911	400.697	7.55077
401.93	6.4523	401.697	7.67906
402.93	6.52865	402.697	7.81416
403.93	6.60727	403.697	7.95501
404.93	6.68599	404.697	8.10101
405.93	6.76909	405.697	8.25366
406.93	6.85279	406.697	8.41415
407.93	6.93838	407.697	8.58102
408.93	7.02894	408.697	8.75606

409.93	7.12038	409.697	8.93927
410.93	7.21409	410.697	9.12801
411.93	7.31054	411.697	9.32529
412.93	7.40943	412.697	9.52766
413.93	7.51131	413.697	9.73996
414.93	7.61806	414.697	9.96152
415.93	7.72744	415.697	10.19007
416.93	7.83705	416.697	10.42581
417.93	7.95162	417.697	10.67197
418.93	8.0663	418.697	10.92802
419.93	8.18611	419.697	11.1954
420.93	8.30825	420.697	11.47151
421.93	8.43337	421.697	11.75932
422.93	8.55816	422.697	12.05731
423.93	8.68637	423.697	12.35947
424.93	8.81912	424.697	12.67692
425.93	8.95141	425.697	13.00584
426.93	9.08511	426.697	13.3397
427.93	9.21711	427.697	13.68333
428.93	9.34836	428.697	14.02164
429.93	9.4784	429.697	14.36552
430.93	9.6075	430.697	14.71022
431.93	9.73419	431.697	15.04177
432.93	9.85905	432.697	15.3767
433.93	9.98342	433.697	15.70313
434.93	10.10597	434.697	16.02063
435.93	10.22676	435.697	16.32943
436.93	10.34653	436.697	16.63089
437.93	10.46489	437.697	16.91401
438.93	10.57661	438.697	17.18125
439.93	10.68238	439.697	17.4222
440.93	10.78448	440.697	17.6306
441.93	10.87619	441.697	17.80078
442.93	10.96004	442.697	17.9245
443.93	11.03304	443.697	17.99244
444.93	11.09721	444.697	18.00196
445.93	11.15449	445.697	17.95065
446.93	11.2051	446.697	17.83593
447.93	11.24808	447.697	17.65642
448.93	11.28327	448.697	17.40343
449.93	11.31024	449.697	17.08118
450.93	11.32925	450.697	16.67983
451.93	11.34044	451.697	16.2061

452.93	11.34348	452.697	15.66361
453.93	11.33848	453.697	15.05765
454.93	11.3271	454.697	14.40826
455.93	11.31107	455.697	13.72392
456.93	11.29029	456.697	13.0166
457.93	11.26326	457.697	12.30761
458.93	11.23051	458.697	11.60057
459.93	11.18902	459.697	10.9075
460.93	11.13637	460.697	10.2283
461.93	11.07179	461.697	9.56938
462.93	10.99042	462.697	8.93698
463.93	10.89761	463.697	8.33514
464.93	10.79152	464.697	7.77681
465.93	10.67375	465.697	7.24724
466.93	10.54137	466.697	6.73968
467.93	10.39547	467.697	6.26905
468.93	10.23643	468.697	5.82279
469.93	10.06743	469.697	5.40869
470.93	9.88882	470.697	5.0109
471.93	9.69965	471.697	4.6386
472.93	9.49504	472.697	4.2933
473.93	9.28565	473.697	3.96639
474.93	9.06683	474.697	3.66744
475.93	8.84733	475.697	3.38714
476.93	8.62394	476.697	3.13061
477.93	8.39638	477.697	2.89355
478.93	8.15823	478.697	2.68167
479.93	7.92356	479.697	2.48651
480.93	7.68597	480.697	2.31545
481.93	7.44494	481.697	2.16308
482.93	7.2062	482.697	2.02661
483.93	6.96764	483.697	1.90532
484.93	6.72872	484.697	1.79925
485.93	6.4945	485.697	1.7042
486.93	6.26182	486.697	1.61914
487.93	6.03804	487.697	1.54315
488.93	5.81542	488.697	1.47486
489.93	5.59809	489.697	1.41237
490.93	5.38853	490.697	1.35649
491.93	5.18173	491.697	1.30378
492.93	4.97887	492.697	1.25633
493.93	4.77891	493.697	1.21178
494.93	4.5853	494.697	1.17025

495.93	4.39774	495.697	1.13139
496.93	4.21545	496.697	1.09552
497.93	4.03952	497.697	1.06201
498.93	3.87055	498.697	1.03095
499.93	3.70601	499.697	1.00206
500.93	3.55116	500.697	0.97536
501.93	3.39974	501.697	0.95083
502.93	3.25702	502.697	0.92828
503.93	3.11902	503.697	0.90718
504.93	2.98785	504.697	0.88804
505.93	2.86224	505.697	0.8695
506.93	2.74298	506.697	0.85233
507.93	2.62976	507.697	0.83628
508.93	2.52278	508.697	0.82097
509.93	2.4216	509.697	0.80622
510.93	2.32574	510.697	0.79225
511.93	2.23491	511.697	0.77876
512.93	2.1493	512.697	0.76628
513.93	2.06598	513.697	0.75403
514.93	1.9892	514.697	0.74222
515.93	1.91664	515.697	0.73114
516.93	1.84856	516.697	0.72065
517.93	1.78366	517.697	0.71043
518.93	1.72305	518.697	0.70052
519.93	1.66591	519.697	0.6906
520.93	1.61116	520.697	0.68077
521.93	1.56007	521.697	0.67142
522.93	1.51105	522.697	0.66232
523.93	1.46439	523.697	0.65332
524.93	1.42008	524.697	0.64407
525.93	1.37792	525.697	0.63501
526.93	1.33718	526.697	0.62646
527.93	1.29876	527.697	0.61823
528.93	1.26216	528.697	0.61013
529.93	1.22687	529.697	0.60206
530.93	1.19317	530.697	0.59421
531.93	1.16047	531.697	0.58664
532.93	1.12948	532.697	0.57919
533.93	1.1	533.697	0.57205
534.93	1.07154	534.697	0.56496
535.93	1.04419	535.697	0.55811
536.93	1.0185	536.697	0.55127
537.93	0.99379	537.697	0.54436

538.93	0.9703	538.697	0.53769
539.93	0.94766	539.697	0.53119
540.93	0.92633	540.697	0.52493
541.93	0.90569	541.697	0.5187
542.93	0.88638	542.697	0.51276
543.93	0.86814	543.697	0.50739
544.93	0.85032	544.697	0.5019
545.93	0.83358	545.697	0.49658
546.93	0.81755	546.697	0.49136
547.93	0.80226	547.697	0.48619
548.93	0.78752	548.697	0.48099
549.93	0.77337	549.697	0.47584
550.93	0.75976	550.697	0.47056
551.93	0.74687	551.697	0.46482
552.93	0.73469	552.697	0.45957
553.93	0.72255	553.697	0.45452
554.93	0.71075	554.697	0.44964
555.93	0.69952	555.697	0.44491
556.93	0.68856	556.697	0.44029
557.93	0.67817	557.697	0.43599
558.93	0.66778	558.697	0.43189
559.93	0.65811	559.697	0.42807
560.93	0.64834	560.697	0.42398
561.93	0.63929	561.697	0.41994
562.93	0.63059	562.697	0.41591
563.93	0.62219	563.697	0.41215
564.93	0.61414	564.697	0.40829
565.93	0.60639	565.697	0.40435
566.93	0.59873	566.697	0.40021
567.93	0.59113	567.697	0.3961
568.93	0.58386	568.697	0.39226
569.93	0.57662	569.697	0.3886
570.93	0.56951	570.697	0.38511
571.93	0.56222	571.697	0.38139
572.93	0.55526	572.697	0.3776
573.93	0.54852	573.697	0.37374
574.93	0.54195	574.697	0.36976
575.93	0.53549	575.697	0.36583
576.93	0.52898	576.697	0.36182
577.93	0.52267	577.697	0.35785
578.93	0.5163	578.697	0.3538
579.93	0.51037	579.697	0.34978
5.81E+02	0.50458	580.697	0.34606

581.93	0.49883	581.697	0.34233
582.93	0.49298	582.697	0.33847
583.93	0.48728	583.697	0.33434
584.93	0.48165	584.697	0.32998
585.93	0.47613	585.697	0.3254
586.93	0.4704	586.697	0.32067
587.93	0.46462	587.697	0.31587
588.93	0.45893	588.697	0.3108
589.93	0.45302	589.697	0.30563
590.93	0.44722	590.697	0.3005
591.93	0.44179	591.697	0.29551
592.93	0.43619	592.697	0.29071
593.93	0.4306	593.697	0.28575
594.93	0.42503	594.697	0.28067
595.93	0.41944	595.697	0.27535
596.93	0.41375	596.697	0.26981
597.93	0.40818	597.697	0.26403
598.93	0.40259	598.697	0.25832
599.93	0.3968	599.697	0.25237
600.93	0.39093	600.697	0.24613
601.93	0.38501	601.697	0.24002
602.93	0.37921	602.697	0.23382
603.93	0.37325	603.697	0.22751
604.93	0.36716	604.697	0.2212
605.93	0.36072	605.697	0.21484
606.93	0.35442	606.697	0.20843
607.93	0.34833	607.697	0.20196
608.93	0.34203	608.697	0.19511
609.93	0.33552	609.697	0.18782
610.93	0.32884	610.697	0.18047
611.93	0.32243	611.697	0.17322
612.93	0.31616	612.697	0.16601
613.93	0.30995	613.697	0.15862
614.93	0.3035	614.697	0.15134
615.93	0.29707	615.697	0.14402
616.93	0.29044	616.697	0.13713
617.93	0.28416	617.697	0.1305
618.93	0.27763	618.697	0.12365
619.93	0.27101	619.697	0.11651
620.93	0.2645	620.697	0.10938
621.93	0.25783	621.697	0.10245
622.93	0.25116	622.697	0.09565678
623.93	0.24419	623.697	0.08890695

624.93	0.23692	624.697	0.08181706
625.93	0.22966	625.697	0.07475078
626.93	0.22238	626.697	0.0681961
627.93	0.21508	627.697	0.06197719
628.93	0.20762	628.697	0.05595091
629.93	0.20025	629.697	0.05008698
630.93	0.19278	630.697	0.04440701
631.93	0.18521	631.697	0.03855115
632.93	0.17764	632.697	0.03282166
633.93	0.16998	633.697	0.02718049
634.93	0.16207	634.697	0.02155775
635.93	0.15413	635.697	0.01606548
636.93	0.14607	636.697	0.01040011
637.93	0.13826	637.697	0.00490276
638.93	0.13005	638.697	-0.00046151
639.93	0.12175	639.697	-0.00566931
640.93	0.11359	640.697	-0.01087806
641.93	0.10544	641.697	-0.0161165
642.93	0.0972168	642.697	-0.02120256
643.93	0.08906823	643.697	-0.02631672
644.93	0.08112727	644.697	-0.03114935
645.93	0.07283566	645.697	-0.03610375
646.93	0.06424195	646.697	-0.04143485
647.93	0.05583457	6.48E+02	-0.04666974
648.93	0.04734013	648.697	-0.05165407
649.93	0.03902428	649.697	-0.05648634
650.93	0.03061112	650.697	-0.06124323
651.93	0.02221718	651.697	-0.06597593
652.93	0.01339384	652.697	-0.07094771
653.93	0.004565906	653.697	-0.07575293
654.93	-0.00387647	654.697	-0.08036477
655.93	-0.01277639	655.697	-0.08515541
656.93	-0.02168422	656.697	-0.09014277
657.93	-0.03078784	657.697	-0.0950587
658.93	-0.03992399	658.697	-0.0999032
659.93	-0.04921434	659.697	-0.10475
660.93	-0.05865334	660.697	-0.10971
661.93	-0.06802442	661.697	-0.11488
662.93	-0.07732469	662.697	-0.11996
663.93	-0.08624861	663.697	-0.12473
664.93	-0.09513454	664.697	-0.12927
665.93	-0.10394	665.697	-0.13389
666.93	-0.1127	666.697	-0.13859

667.93	-0.12145	667.697	-0.14327
668.93	-0.13037	668.697	-0.14782
669.93	-0.13927	669.697	-0.15232
670.93	-0.14828	670.697	-0.15674
671.93	-0.15718	671.697	-0.1614
672.93	-0.16645	672.697	-0.16608
673.93	-0.17565	673.697	-0.1707
674.93	-0.18485	674.697	-0.17514
675.93	-0.19365	675.697	-0.17918
676.93	-0.20233	676.697	-0.18315
677.93	-0.21114	677.697	-0.187
678.93	-0.22012	678.697	-0.19089
679.93	-0.22913	679.697	-0.19477
680.93	-0.23793	680.697	-0.19849
681.93	-0.24641	681.697	-0.20206
682.93	-0.2549	682.697	-0.20575
683.93	-0.26378	6.84E+02	-0.20969
684.93	-0.2725	684.697	-0.21346
685.93	-0.28123	685.697	-0.21737
686.93	-0.28961	686.697	-0.22104
687.93	-0.29796	687.697	-0.22442
688.93	-0.30662	6.89E+02	-0.2277
689.93	-0.3154	689.697	-0.231
690.93	-0.32427	690.697	-0.23413
691.93	-0.33319	691.697	-0.23727
692.93	-0.34193	692.697	-0.24047
693.93	-0.35059	6.94E+02	-0.24357
694.93	-0.35921	694.697	-0.24651
695.93	-0.36807	695.697	-0.24942
696.93	-0.3771	696.697	-0.25208
697.93	-0.38587	697.697	-0.25466
698.93	-0.39454	698.697	-0.25715
699.93	-0.40325	699.697	-0.25954
700.93	-0.41189	700.697	-0.26171
701.93	-0.42033	701.697	-0.2636
702.93	-0.4293	702.697	-0.26578
703.93	-0.43789	703.697	-0.26793
704.93	-0.44629	704.697	-0.27004
705.93	-0.45467	705.697	-0.27194
706.93	-0.46293	706.697	-0.27369
707.93	-0.47118	707.697	-0.2754
708.93	-0.47953	708.697	-0.27706
709.93	-0.48779	709.697	-0.27859

710.93	-0.49568	710.697	-0.2797
711.93	-0.50384	711.697	-0.28066
712.93	-0.51223	712.697	-0.28158
713.93	-0.52038	713.697	-0.28263
714.93	-0.52849	714.697	-0.28378
715.93	-0.53646	715.697	-0.2848
716.93	-0.54427	7.17E+02	-0.28558
717.93	-0.55199	717.697	-0.28625
718.93	-0.55957	718.697	-0.28691
719.93	-0.56645	719.697	-0.28743
720.93	-0.57305	720.697	-0.28789
721.93	-0.57945	721.697	-0.28812
722.93	-0.5858	722.697	-0.28824
723.93	-0.59213	723.697	-0.28818
724.93	-0.59798	724.697	-0.28797
725.93	-0.60361	725.697	-0.28781
726.93	-0.60914	726.697	-0.28768
727.93	-0.61457	727.697	-0.28762
728.93	-0.61974	7.29E+02	-0.28787
729.93	-0.62429	729.697	-0.28808
730.93	-0.62819	730.697	-0.28789
731.93	-0.63179	731.697	-0.28756
732.93	-0.63506	7.33E+02	-0.28714
733.93	-0.63797	733.697	-0.28659
734.93	-0.64041	734.697	-0.28615
735.93	-0.64225	7.36E+02	-0.28535
736.93	-0.64396	736.697	-0.28412
737.93	-0.64575	737.697	-0.28273
738.93	-0.64728	738.697	-0.28146
739.93	-0.64813	739.697	-0.28017
740.93	-0.64832	740.697	-0.27882
741.93	-0.64798	741.697	-0.27749
742.93	-0.64733	742.697	-0.27585
743.93	-0.64674	743.697	-0.27444
744.93	-0.6457	744.697	-0.27288
745.93	-0.6441	7.46E+02	-0.27141
746.93	-0.64216	746.697	-0.2698
747.93	-0.63988	747.697	-0.26821
748.93	-0.63725	7.49E+02	-0.26664
749.93	-0.63433	749.697	-0.26491
7.51E+02	-0.63098	750.697	-0.26316
751.93	-0.62708	751.697	-0.26126
752.93	-0.62293	752.697	-0.25948

753.93	-0.6183	753.697	-0.25745
754.93	-0.61336	754.697	-0.2555
755.93	-0.60847	755.697	-0.25346
756.93	-0.6035	756.697	-0.25144
757.93	-0.5986	757.697	-0.24953
758.93	-0.59352	758.697	-0.24764
759.93	-0.58818	7.60E+02	-0.24566
760.93	-0.58263	760.697	-0.24361
761.93	-0.57701	761.697	-0.24164
762.93	-0.57137	762.697	-0.2398
763.93	-0.56548	763.697	-0.23787
764.93	-0.55949	764.697	-0.23602
765.93	-0.55335	765.697	-0.23409
766.93	-0.54715	766.697	-0.23207
767.93	-0.54103	767.697	-0.23033
768.93	-0.53486	768.697	-0.22858
769.93	-0.52851	769.697	-0.22684
770.93	-0.52221	770.697	-0.22498
771.93	-0.51616	771.697	-0.22331
772.93	-0.51006	772.697	-0.22173
773.93	-0.5038	773.697	-0.22008
774.93	-0.49725	774.697	-0.21833
775.93	-0.49094	775.697	-0.21658
776.93	-0.48459	776.697	-0.21471
777.93	-0.47822	777.697	-0.21282
778.93	-0.47185	778.697	-0.21107
779.93	-0.46562	779.697	-0.20943
780.93	-0.45947	780.697	-0.20773
781.93	-0.45372	781.697	-0.20617
782.93	-0.44812	782.697	-0.20473
783.93	-0.44285	783.697	-0.2034
784.93	-0.43761	784.697	-0.20206
785.93	-0.43251	785.697	-0.20055
786.93	-0.42764	786.697	-0.19894
787.93	-0.42291	7.88E+02	-0.19734
788.93	-0.4182	788.697	-0.19575
789.93	-0.41342	789.697	-0.19415
790.93	-0.40848	7.91E+02	-0.19255
791.93	-0.4036	791.697	-0.19099
792.93	-0.39922	792.697	-0.18931
793.93	-0.39525	793.697	-0.18823
794.93	-0.39076	794.697	-0.18691
795.93	-0.3867	795.697	-0.18557

796.93	-0.38444	7.97E+02	-0.18461

DSC curve of water-immersed coking coal

temperature	***DSC***	***temperature***	***DSC***	***temperature***	***DSC***
℃	mW/mg	℃	mW/mg	℃	mW/mg
	Coal		Coal+MgCl2		Coal+TEMPO
30.506	-0.021533	29.991	-0.03604448	29.267	0.01075589
31.506	-0.02094389	30.991	-0.02415524	30.267	-0.01794163
32.506	-0.02463488	31.991	-0.02046756	31.267	-0.02098051
33.506	-0.02768784	32.991	-0.01933847	32.267	-0.01801932
34.506	-0.02876193	33.991	-0.01914941	33.267	-0.01525743
35.506	-0.02727391	34.991	-0.01927272	34.267	-0.01306464
36.506	-0.02501183	35.991	-0.01958764	35.267	-0.01143796
37.506	-0.02281233	36.991	-0.02020901	36.267	-0.01018184
38.506	-0.02108246	37.991	-0.02129562	37.267	-0.009192485
39.506	-0.01968273	38.991	-0.02292072	38.267	-0.008333957
40.506	-0.01856453	39.991	-0.02503361	39.267	-0.007590628
41.506	-0.01769955	40.991	-0.02767234	40.267	-0.00681102
42.506	-0.01686539	41.991	-0.03072207	41.267	-0.006034637
43.506	-0.01608546	42.991	-0.03405016	42.267	-0.005215472
44.506	-0.01529745	43.991	-0.03792406	43.267	-0.004364536
45.506	-0.01450364	44.991	-0.04179703	44.267	-0.003474161
46.506	-0.01370278	45.991	-0.04585601	45.267	-0.002541089
47.506	-0.0128633	46.991	-0.05012414	46.267	-0.001486867
48.506	-0.01204969	47.991	-0.0544959	47.267	-0.000495693
49.506	-0.0112307	48.991	-0.0589227	48.267	0.000545758
50.506	-0.01037067	49.991	-0.06370275	49.267	0.001561309
51.506	-0.00955167	50.991	-0.0687663	50.267	0.002610656
52.506	-0.00862213	51.991	-0.07386013	51.267	0.003652195
53.506	-0.00769208	52.991	-0.07921169	52.267	0.004730456
54.506	-0.00664391	53.991	-0.08488525	53.267	0.005898534
55.506	-0.00556024	54.991	-0.09084355	54.267	0.007113828
56.506	-0.00452175	55.991	-0.0969295	55.267	0.008332805
57.506	-0.00352176	56.991	-0.10336	56.267	0.009528446
58.506	-0.0024249	57.991	-0.11004	57.267	0.01074651
59.506	-0.00126465	58.991	-0.11678	58.267	0.01199476
60.506	-0.00012194	59.991	-0.12383	59.267	0.01330005
61.506	0.001041982	60.991	-0.13066	60.267	0.01458582
62.506	0.002250743	61.991	-0.13732	61.267	0.01591777
63.506	0.003435645	62.991	-0.14352	62.267	0.01724602
64.506	0.004670272	63.991	-0.14888	63.267	0.01859272
65.506	0.005971655	64.991	-0.1531	64.267	0.01992874

66.506	0.00731243	65.991	-0.15617	65.267	0.02124101
67.506	0.008659702	66.991	-0.15771	66.267	0.02265826
68.506	0.01001408	67.991	-0.15745	67.267	0.02404854
69.506	0.01139354	68.991	-0.15496	68.267	0.02544671
70.506	0.01278561	69.991	-0.15019	69.267	0.02690531
71.506	0.01411712	70.991	-0.14391	70.267	0.02836544
72.506	0.01553566	71.991	-0.13676	71.267	0.02980746
73.506	0.01699665	72.991	-0.12959	72.267	0.03114932
74.506	0.01850222	73.991	-0.12283	73.267	0.03255422
75.506	0.01998738	74.991	-0.11708	74.267	0.03393625
76.506	0.02156409	75.991	-0.11256	75.267	0.03537801
77.506	0.02308439	76.991	-0.10967	76.267	0.03680834
78.506	0.02462048	77.991	-0.10899	77.267	0.03830052
79.506	0.02624649	78.991	-0.11095	78.267	0.03977369
80.506	0.0279179	79.991	-0.11547	79.267	0.0412888
81.506	0.02957226	80.991	-0.12197	80.267	0.04278459
82.506	0.0311806	81.991	-0.12989	81.267	0.04439746
83.506	0.0327855	82.991	-0.13903	82.267	0.0460187
84.506	0.0345672	83.991	-0.14841	83.267	0.04765897
85.506	0.03634516	84.991	-0.15788	84.267	0.04929534
86.506	0.03816248	85.991	-0.1658	85.267	0.05096485
87.506	0.03999814	86.991	-0.17021	86.267	0.05269442
88.506	0.04179401	87.991	-0.17046	87.267	0.05445758
89.506	0.04366536	88.991	-0.16782	88.267	0.05620203
90.506	0.0455872	89.991	-0.16372	89.267	0.05803204
91.506	0.04759146	90.991	-0.15907	90.267	0.05985966
92.506	0.04960264	91.991	-0.15441	91.267	0.06175833
93.506	0.0516534	92.991	-0.1501	92.267	0.06370068
94.506	0.05366435	93.991	-0.14654	93.267	0.06568279
95.506	0.05573747	94.991	-0.14447	94.267	0.06770241
96.506	0.05785344	95.991	-0.14554	95.267	0.06969822
97.506	0.05998224	96.991	-0.15157	96.267	0.07175543
98.506	0.06208056	97.991	-0.16188	97.267	0.07387669
99.506	0.06427391	98.991	-0.17473	98.267	0.07599585
100.506	0.06646484	99.991	-0.18867	99.267	0.07812881
101.506	0.06867505	100.991	-0.20275	100.267	0.08036865
102.506	0.07084046	101.991	-0.21596	101.267	0.08262337
103.506	0.07304452	102.991	-0.22671	102.267	0.08484406
104.506	0.07527761	103.991	-0.23156	103.267	0.08710545
105.506	0.07747451	104.991	-0.2273	104.267	0.0893694
106.506	0.07974855	105.991	-0.21435	105.267	0.0917367
107.506	0.08205824	106.991	-0.19498	106.267	0.09406789
108.506	0.08444662	107.991	-0.16977	107.267	0.09646074

109.506	0.08683059	108.991	-0.14016	108.267	0.09891311
110.506	0.08922329	109.991	-0.1075	109.267	0.10141
111.506	0.09168796	110.991	-0.07325944	110.267	0.10392
112.506	0.09416158	111.991	-0.03828533	111.267	0.10639
113.506	0.09666727	112.991	-0.00750946	112.267	0.10895
114.506	0.09917904	113.991	0.01648508	113.267	0.11158
115.506	0.1017	114.991	0.03314942	114.267	0.11418
116.506	0.10424	115.991	0.04463084	115.267	0.11694
117.506	0.10681	116.991	0.05185118	116.267	0.11967
118.506	0.10946	117.991	0.05600213	117.267	0.12234
119.506	0.11218	118.991	0.0576999	118.267	0.12507
120.506	0.11487	119.991	0.0575238	119.267	0.12801
121.506	0.11757	120.991	0.05606616	120.267	0.13093
122.506	0.12027	121.991	0.05391021	121.267	0.13385
123.506	0.12304	122.991	0.05160305	122.267	0.1368
124.506	0.12583	123.991	0.04965271	123.267	0.1398
125.506	0.12867	124.991	0.0483913	124.267	0.14273
126.506	0.13155	125.991	0.04846946	125.267	0.14579
127.506	0.13445	126.991	0.05034299	126.267	0.14889
128.506	0.13731	127.991	0.0541331	127.267	0.15198
129.506	0.14027	128.991	0.05962148	128.267	0.15504
130.506	0.14333	129.991	0.06666312	129.267	0.15816
131.506	0.14637	130.991	0.07447966	130.267	0.16132
132.506	0.14944	131.991	0.08245622	131.267	0.16444
133.506	0.15256	132.991	0.08929691	132.267	0.16767
134.506	0.15575	133.991	0.09430648	133.267	0.17084
135.506	0.15883	134.991	0.09695596	134.267	0.17412
136.506	0.16199	135.991	0.09697518	135.267	0.17745
137.506	0.1652	136.991	0.09443559	136.267	0.18074
138.506	0.16836	137.991	0.08977764	137.267	0.18412
139.506	0.17165	138.991	0.08334095	138.267	0.18743
140.506	0.17487	139.991	0.07622688	139.267	0.19086
141.506	0.17823	140.991	0.06943277	140.267	0.19428
142.506	0.18165	141.991	0.06386025	141.267	0.1978
143.506	0.18503	142.991	0.06037454	142.267	0.2013
144.506	0.18841	143.991	0.0594235	143.267	0.20482
145.506	0.19197	144.991	0.06175321	144.267	0.20838
146.506	0.19552	145.991	0.06813155	145.267	0.212
147.506	0.19908	146.991	0.07836357	146.267	0.21572
148.506	0.20272	147.991	0.09188326	147.267	0.21931
149.506	0.2064	148.991	0.10774	148.267	0.22313
150.506	0.21014	149.991	0.12529	149.267	0.22702
151.506	0.21397	150.991	0.14338	150.267	0.23085

152.506	0.21787	151.991	0.16218	151.267	0.2348
153.506	0.22188	152.991	0.1802	152.267	0.2388
154.506	0.22585	153.991	0.19674	153.267	0.24284
155.506	0.22999	154.991	0.21137	154.267	0.24696
156.506	0.23411	155.991	0.22367	155.267	0.25104
157.506	0.23831	156.991	0.23462	156.267	0.25525
158.506	0.24251	157.991	0.24439	157.267	0.25949
159.506	0.24675	158.991	0.2534	158.267	0.26368
160.506	0.25101	159.991	0.26203	159.267	0.26804
161.506	0.25532	160.991	0.27019	160.267	0.27243
162.506	0.2598	161.991	0.27792	161.267	0.27683
163.506	0.2643	162.991	0.28516	162.267	0.28131
164.506	0.26879	163.991	0.29209	163.267	0.28587
165.506	0.27338	164.991	0.29876	164.267	0.2905
166.506	0.27795	165.991	0.30509	165.267	0.29511
167.506	0.28273	166.991	0.31137	166.267	0.29981
168.506	0.28768	167.991	0.31766	167.267	0.30465
169.506	0.29251	168.991	0.32391	168.267	0.30947
170.506	0.29747	169.991	0.32991	169.267	0.31438
171.506	0.30247	170.991	0.33589	170.267	0.31921
172.506	0.30749	171.991	0.34192	171.267	0.324
173.506	0.31269	172.991	0.34805	172.267	0.32893
174.506	0.31787	173.991	0.35411	173.267	0.334
175.506	0.32318	174.991	0.36019	174.267	0.33901
176.506	0.3285	175.991	0.36632	175.267	0.34404
177.506	0.33374	176.991	0.37251	176.267	0.34914
178.506	0.33926	177.991	0.37868	177.267	0.35437
179.506	0.34472	178.991	0.38501	178.267	0.35963
180.506	0.35029	179.991	0.39132	179.267	0.36489
181.506	0.35591	180.991	0.39762	180.267	0.3703
182.506	0.36162	181.991	0.40372	181.267	0.37577
183.506	0.36738	182.991	0.40979	182.267	0.38125
184.506	0.37309	183.991	0.41569	183.267	0.38693
185.506	0.37887	184.991	0.42145	184.267	0.39252
186.506	0.38484	185.991	0.42713	185.267	0.39814
187.506	0.39068	186.991	0.43275	186.267	0.40393
188.506	0.39669	187.991	0.43809	187.267	0.40969
189.506	0.4028	188.991	0.44353	188.267	0.41555
190.506	0.40875	189.991	0.44885	189.267	0.4215
191.506	0.41496	190.991	0.4541	190.267	0.42758
192.506	0.42127	191.991	0.45938	191.267	0.43365
193.506	0.42755	192.991	0.46482	192.267	0.43984
194.506	0.43392	193.991	0.47025	193.267	0.44606

195.506	0.44049	194.991	0.47606	194.267	0.45231
196.506	0.44709	195.991	0.48194	195.267	0.45863
197.506	0.45375	196.991	0.48824	196.267	0.46514
198.506	0.46048	197.991	0.49523	197.267	0.47149
199.506	0.46739	198.991	0.50292	198.267	0.47808
200.506	0.47421	199.991	0.51161	199.267	0.48474
201.506	0.48138	200.991	0.52148	200.267	0.49139
202.506	0.4885	201.991	0.53311	201.267	0.49815
203.506	0.4959	202.991	0.54648	202.267	0.50508
204.506	0.5032	203.991	0.56144	203.267	0.51213
205.506	0.5109	204.991	0.57693	204.267	0.51941
206.506	0.51851	205.991	0.59296	205.267	0.52668
207.506	0.5262	206.991	0.60891	206.267	0.53401
208.506	0.53404	207.991	0.6244	207.267	0.54145
209.506	0.54183	208.991	0.63899	208.267	0.54905
210.506	0.54996	209.991	0.65252	209.267	0.55664
211.506	0.55812	210.991	0.66459	210.267	0.56453
212.506	0.56624	211.991	0.67551	211.267	0.57228
213.506	0.57468	212.991	0.68557	212.267	0.58007
214.506	0.58321	213.991	0.69536	213.267	0.58819
215.506	0.59186	214.991	0.70512	214.267	0.5964
216.506	0.60071	215.991	0.71495	215.267	0.60491
217.506	0.60968	216.991	0.72483	216.267	0.6132
218.506	0.61872	217.991	0.73487	217.267	0.62184
219.506	0.62792	218.991	0.74511	218.267	0.63071
220.506	0.63746	219.991	0.75565	219.267	0.63958
221.506	0.64697	220.991	0.76626	220.267	0.64864
222.506	0.65681	221.991	0.77734	221.267	0.6578
223.506	0.6666	222.991	0.78889	222.267	0.6672
224.506	0.67667	223.991	0.80028	223.267	0.67679
225.506	0.68696	224.991	0.81212	224.267	0.68638
226.506	0.6973	225.991	0.82408	225.267	0.6963
227.506	0.70797	226.991	0.83661	226.267	0.70654
228.506	0.71864	227.991	0.84902	227.267	0.71681
229.506	0.72971	228.991	0.86139	228.267	0.72735
230.506	0.74062	229.991	0.87461	229.267	0.73815
231.506	0.75196	230.991	0.88763	230.267	0.74922
232.506	0.76327	231.991	0.90095	231.267	0.76042
233.506	0.77479	232.991	0.91448	232.267	0.77206
234.506	0.78678	233.991	0.92815	233.267	0.7837
235.506	0.79865	234.991	0.94216	234.267	0.79573
236.506	0.81087	235.991	0.95631	235.267	0.80798
237.506	0.82321	236.991	0.97059	236.267	0.82024

238.506	0.83588	237.991	0.98509	237.267	0.83261
239.506	0.84867	238.991	1.00009	238.267	0.84519
240.506	0.86188	239.991	1.01522	239.267	0.85822
241.506	0.8751	240.991	1.03058	240.267	0.8711
242.506	0.88886	241.991	1.04607	241.267	0.88441
243.506	0.90289	242.991	1.06168	242.267	0.89799
244.506	0.91694	243.991	1.07716	243.267	0.91187
245.506	0.93122	244.991	1.09263	244.267	0.92574
246.506	0.94607	245.991	1.10781	245.267	0.94029
247.506	0.96086	246.991	1.1226	246.267	0.95475
248.506	0.97635	247.991	1.13708	247.267	0.96952
249.506	0.99201	248.991	1.15144	248.267	0.98478
250.506	1.00778	249.991	1.16543	249.267	0.99993
251.506	1.02378	250.991	1.1795	250.267	1.01558
252.506	1.04027	251.991	1.19348	251.267	1.0313
253.506	1.057	252.991	1.20764	252.267	1.04756
254.506	1.07395	253.991	1.22208	253.267	1.0641
255.506	1.09106	254.991	1.23725	254.267	1.08122
256.506	1.10881	255.991	1.25211	255.267	1.0986
257.506	1.12672	256.991	1.26764	256.267	1.11602
258.506	1.14479	257.991	1.28316	257.267	1.13443
259.506	1.16313	258.991	1.29903	258.267	1.15298
260.506	1.18225	259.991	1.31516	259.267	1.17161
261.506	1.20132	260.991	1.33179	260.267	1.19074
262.506	1.22094	261.991	1.34877	261.267	1.21037
263.506	1.24111	262.991	1.36626	262.267	1.23042
264.506	1.26122	263.991	1.38367	263.267	1.25101
265.506	1.28234	264.991	1.40188	264.267	1.27188
266.506	1.30375	265.991	1.42045	265.267	1.29298
267.506	1.32544	266.991	1.43951	266.267	1.31461
268.506	1.34826	267.991	1.4588	267.267	1.33675
269.506	1.3709	268.991	1.47855	268.267	1.35954
270.506	1.39405	269.991	1.49878	269.267	1.38291
271.506	1.41798	270.991	1.51932	270.267	1.40661
272.506	1.44257	271.991	1.54046	271.267	1.43061
273.506	1.46711	272.991	1.56226	272.267	1.45565
274.506	1.49281	273.991	1.58391	273.267	1.48062
275.506	1.51863	274.991	1.60633	274.267	1.50675
276.506	1.54516	275.991	1.62877	275.267	1.53334
277.506	1.57193	276.991	1.65157	276.267	1.56046
278.506	1.59965	277.991	1.67512	277.267	1.58791
279.506	1.62762	278.991	1.69877	278.267	1.61638
280.506	1.65615	279.991	1.72292	279.267	1.64565

281.506	1.68581	280.991	1.74725	280.267	1.6757
282.506	1.71599	281.991	1.77118	281.267	1.70644
283.506	1.74645	282.991	1.79631	282.267	1.73811
284.506	1.77811	283.991	1.82133	283.267	1.77028
285.506	1.81065	284.991	1.84667	284.267	1.8034
286.506	1.84292	285.991	1.87254	285.267	1.83717
287.506	1.87649	286.991	1.89846	286.267	1.87198
288.506	1.91124	287.991	1.92469	287.267	1.90716
289.506	1.94555	288.991	1.95129	288.267	1.94326
290.506	1.98087	289.991	1.97825	289.267	1.98005
291.506	2.01695	290.991	2.00529	290.267	2.01759
292.506	2.054	291.991	2.03243	291.267	2.05575
293.506	2.09118	292.991	2.05964	292.267	2.09446
294.506	2.12889	293.991	2.08721	293.267	2.13466
295.506	2.16746	294.991	2.11449	294.267	2.17519
296.506	2.20685	295.991	2.14201	295.267	2.21662
297.506	2.24653	296.991	2.16917	296.267	2.25881
298.506	2.28689	297.991	2.19651	297.267	2.3015
299.506	2.32751	298.991	2.22416	298.267	2.34451
300.506	2.36844	299.991	2.25117	299.267	2.38876
301.506	2.4097	300.991	2.27913	300.267	2.43284
302.506	2.45145	301.991	2.30591	301.267	2.47761
303.506	2.49301	302.991	2.33269	302.267	2.5225
304.506	2.53444	303.991	2.35997	303.267	2.567
305.506	2.57608	304.991	2.38658	304.267	2.61268
306.506	2.61727	305.991	2.41296	305.267	2.65717
307.506	2.658	306.991	2.43888	306.267	2.7024
308.506	2.69848	307.991	2.46453	307.267	2.74653
309.506	2.73957	308.991	2.49017	308.267	2.79032
310.506	2.77872	309.991	2.51534	309.267	2.83374
311.506	2.81804	310.991	2.54039	310.267	2.87654
312.506	2.85559	311.991	2.56534	311.267	2.91846
313.506	2.89262	312.991	2.58965	312.267	2.96009
314.506	2.92931	313.991	2.61375	313.267	3.00008
315.506	2.96472	314.991	2.63753	314.267	3.03863
316.506	2.99888	315.991	2.66093	315.267	3.07615
317.506	3.03186	316.991	2.68311	316.267	3.11242
318.506	3.06399	317.991	2.70501	317.267	3.14763
319.506	3.09513	318.991	2.72628	318.267	3.18109
320.506	3.12475	319.991	2.74644	319.267	3.21312
321.506	3.15308	320.991	2.76644	320.267	3.24423
322.506	3.18026	321.991	2.78584	321.267	3.27344
323.506	3.2057	322.991	2.80474	322.267	3.30117

324.506	3.23055	323.991	2.82326	323.267	3.328
325.506	3.2539	324.991	2.84146	324.267	3.35321
326.506	3.27662	325.991	2.85878	325.267	3.37706
327.506	3.2979	326.991	2.87571	326.267	3.39973
328.506	3.31832	327.991	2.89211	327.267	3.42141
329.506	3.33773	328.991	2.90771	328.267	3.44183
330.506	3.35625	329.991	2.92242	329.267	3.46111
331.506	3.3739	330.991	2.93697	330.267	3.47926
332.506	3.3904	331.991	2.95112	331.267	3.49632
333.506	3.40619	332.991	2.965	332.267	3.51223
334.506	3.42073	333.991	2.97858	333.267	3.52695
335.506	3.4345	334.991	2.99207	334.267	3.54071
336.506	3.44724	335.991	3.0052	335.267	3.55345
337.506	3.45888	336.991	3.01804	336.267	3.5654
338.506	3.47002	337.991	3.0307	337.267	3.57649
339.506	3.4805	338.991	3.04309	338.267	3.58659
340.506	3.49032	339.991	3.05481	339.267	3.59641
341.506	3.49959	340.991	3.06629	340.267	3.60545
342.506	3.50873	341.991	3.07721	341.267	3.61406
343.506	3.51749	342.991	3.08769	342.267	3.62206
344.506	3.52623	343.991	3.09796	343.267	3.62972
345.506	3.53446	344.991	3.10802	344.267	3.63705
346.506	3.54266	345.991	3.11798	345.267	3.64388
347.506	3.5506	346.991	3.12763	346.267	3.65042
348.506	3.55851	347.991	3.13716	347.267	3.65669
349.506	3.56636	348.991	3.14659	348.267	3.66271
350.506	3.57419	349.991	3.15582	349.267	3.66884
351.506	3.58212	350.991	3.16507	350.267	3.6748
352.506	3.59012	351.991	3.17428	351.267	3.68086
353.506	3.59816	352.991	3.18359	352.267	3.68684
354.506	3.60633	353.991	3.19332	353.267	3.69302
355.506	3.61459	354.991	3.20316	354.267	3.69932
356.506	3.62297	355.991	3.21327	355.267	3.70579
357.506	3.63143	356.991	3.2239	356.267	3.71227
358.506	3.64022	357.991	3.23453	357.267	3.71891
359.506	3.64893	358.991	3.24543	358.267	3.72562
360.506	3.65758	359.991	3.25642	359.267	3.73227
361.506	3.66655	360.991	3.26746	360.267	3.73903
362.506	3.67552	361.991	3.27864	361.267	3.74589
363.506	3.68474	362.991	3.28975	362.267	3.75264
364.506	3.694	363.991	3.30077	363.267	3.7595
365.506	3.70342	364.991	3.31184	364.267	3.76642
366.506	3.71295	365.991	3.32289	365.267	3.77353

367.506	3.72257	366.991	3.33392	366.267	3.78091
368.506	3.7323	367.991	3.34494	367.267	3.78844
369.506	3.7422	368.991	3.35574	368.267	3.7962
370.506	3.75212	369.991	3.36672	369.267	3.80407
371.506	3.76195	370.991	3.3776	370.267	3.81209
372.506	3.77208	371.991	3.38857	371.267	3.81998
373.506	3.78216	372.991	3.39963	372.267	3.82807
374.506	3.79216	373.991	3.41089	373.267	3.83589
375.506	3.80227	374.991	3.42224	374.267	3.84365
376.506	3.81254	375.991	3.43363	375.267	3.85126
377.506	3.82256	376.991	3.44534	376.267	3.8589
378.506	3.83271	377.991	3.45687	377.267	3.86623
379.506	3.84285	378.991	3.46832	378.267	3.87374
380.506	3.85272	379.991	3.47982	379.267	3.88117
381.506	3.86265	380.991	3.4911	380.267	3.88849
382.506	3.87245	381.991	3.50216	381.267	3.89603
383.506	3.88221	382.991	3.51303	382.267	3.9035
384.506	3.89186	383.991	3.52346	383.267	3.91095
385.506	3.90136	384.991	3.53423	384.267	3.91838
386.506	3.91097	385.991	3.54506	385.267	3.92627
387.506	3.92034	386.991	3.55605	386.267	3.93385
388.506	3.92983	387.991	3.56752	387.267	3.94164
389.506	3.93906	388.991	3.57915	388.267	3.9496
390.506	3.94841	389.991	3.59075	389.267	3.95765
391.506	3.95788	390.991	3.60236	390.267	3.96592
392.506	3.96744	391.991	3.61393	391.267	3.97435
393.506	3.97714	392.991	3.62546	392.267	3.98283
394.506	3.98734	393.991	3.63698	393.267	3.99147
395.506	3.99785	394.991	3.64864	394.267	4.00037
396.506	4.00851	395.991	3.66002	395.267	4.00957
397.506	4.01986	396.991	3.67251	396.267	4.01897
398.506	4.03158	397.991	3.68528	397.267	4.02895
399.506	4.04376	398.991	3.69869	398.267	4.03929
400.506	4.05665	399.991	3.71277	399.267	4.05017
401.506	4.07031	400.991	3.72776	400.267	4.06137
402.506	4.08418	401.991	3.74303	401.267	4.0732
403.506	4.09878	402.991	3.75912	402.267	4.08546
404.506	4.11394	403.991	3.77631	403.267	4.0986
405.506	4.12977	404.991	3.79465	404.267	4.11192
406.506	4.14565	405.991	3.81396	405.267	4.12604
407.506	4.16223	406.991	3.83448	406.267	4.14077
408.506	4.17889	407.991	3.85608	407.267	4.15592
409.506	4.19615	408.991	3.8789	408.267	4.17197

410.506	4.21371	409.991	3.90191	409.267	4.18884
411.506	4.2318	410.991	3.92589	410.267	4.20604
412.506	4.25041	411.991	3.94982	411.267	4.22406
413.506	4.26959	412.991	3.97387	412.267	4.24265
414.506	4.28965	413.991	3.99832	413.267	4.26204
415.506	4.31052	414.991	4.02281	414.267	4.28222
416.506	4.33215	415.991	4.04706	415.267	4.30267
417.506	4.3551	416.991	4.0719	416.267	4.32428
418.506	4.37878	417.991	4.09771	417.267	4.34671
419.506	4.40326	418.991	4.12418	418.267	4.36975
420.506	4.42852	419.991	4.15214	419.267	4.394
421.506	4.455	420.991	4.1814	420.267	4.4191
422.506	4.48223	421.991	4.21186	421.267	4.44541
423.506	4.51022	422.991	4.24534	422.267	4.47279
424.506	4.53972	423.991	4.28192	423.267	4.50138
425.506	4.56898	424.991	4.32155	424.267	4.53133
426.506	4.60068	425.991	4.36531	425.267	4.56236
427.506	4.63339	426.991	4.41329	426.267	4.59442
428.506	4.66683	427.991	4.46379	427.267	4.62804
429.506	4.7013	428.991	4.52059	428.267	4.66233
430.506	4.73664	429.991	4.57838	429.267	4.69817
431.506	4.77386	430.991	4.63904	430.267	4.73557
432.506	4.81168	431.991	4.70112	431.267	4.77414
433.506	4.85068	432.991	4.76442	432.267	4.81346
434.506	4.89104	433.991	4.82964	433.267	4.85458
435.506	4.93281	434.991	4.89465	434.267	4.8968
436.506	4.97592	435.991	4.96216	435.267	4.93986
437.506	5.02011	436.991	5.03187	436.267	4.98468
438.506	5.06649	437.991	5.10157	437.267	5.03052
439.506	5.11431	438.991	5.17465	438.267	5.07724
440.506	5.16262	439.991	5.24915	439.267	5.1264
441.506	5.21365	440.991	5.32514	440.267	5.17672
442.506	5.26601	441.991	5.40354	441.267	5.22842
443.506	5.32047	442.991	5.48203	442.267	5.28257
444.506	5.37608	443.991	5.56183	443.267	5.33781
445.506	5.43399	444.991	5.64204	444.267	5.39539
446.506	5.49434	445.991	5.72198	445.267	5.45476
447.506	5.55602	446.991	5.80346	446.267	5.51722
448.506	5.62025	447.991	5.88591	447.267	5.57926
449.506	5.68564	448.991	5.9685	448.267	5.64397
450.506	5.75455	449.991	6.05293	449.267	5.71128
451.506	5.82395	450.991	6.13883	450.267	5.78017
452.506	5.89526	451.991	6.22882	451.267	5.85056

453.506	5.96894	452.991	6.31882	452.267	5.92311
454.506	6.04451	453.991	6.41033	453.267	5.99836
455.506	6.12313	454.991	6.5031	454.267	6.07503
456.506	6.20305	455.991	6.59543	455.267	6.15479
457.506	6.28548	456.991	6.6856	456.267	6.23706
458.506	6.37147	457.991	6.77518	457.267	6.31955
459.506	6.45785	458.991	6.86516	458.267	6.40549
460.506	6.54751	459.991	6.95481	459.267	6.49286
461.506	6.64027	460.991	7.04439	460.267	6.58151
462.506	6.73494	461.991	7.13471	461.267	6.67349
463.506	6.83308	462.991	7.22608	462.267	6.76723
464.506	6.93492	463.991	7.31944	463.267	6.86388
465.506	7.04071	464.991	7.41407	464.267	6.96329
466.506	7.15006	465.991	7.51139	465.267	7.06555
467.506	7.2628	466.991	7.60872	466.267	7.16756
468.506	7.3804	467.991	7.7082	467.267	7.2756
469.506	7.49938	468.991	7.8092	468.267	7.38482
470.506	7.62194	469.991	7.90983	469.267	7.49821
471.506	7.7488	470.991	8.01143	470.267	7.61273
472.506	7.87597	471.991	8.11639	471.267	7.73178
473.506	8.00256	472.991	8.22274	472.267	7.85127
474.506	8.13475	473.991	8.32716	473.267	7.97401
475.506	8.26669	474.991	8.43598	474.267	8.09742
476.506	8.40354	475.991	8.5413	475.267	8.2226
477.506	8.53858	476.991	8.6488	476.267	8.34791
478.506	8.67668	477.991	8.75564	477.267	8.47225
479.506	8.81424	478.991	8.85942	478.267	8.59576
480.506	8.95086	479.991	8.96192	479.267	8.71721
481.506	9.09069	480.991	9.06259	480.267	8.83888
482.506	9.23067	481.991	9.16175	481.267	8.96047
483.506	9.36855	482.991	9.25704	482.267	9.08183
484.506	9.50449	483.991	9.34897	483.267	9.19943
485.506	9.63807	484.991	9.43888	484.267	9.31823
486.506	9.76924	485.991	9.52303	485.267	9.43341
487.506	9.8972	486.991	9.60679	486.267	9.54956
488.506	10.02311	487.991	9.68582	487.267	9.66313
489.506	10.14413	488.991	9.76114	488.267	9.77405
490.506	10.26046	489.991	9.83195	489.267	9.88202
491.506	10.37347	490.991	9.89842	490.267	9.98431
492.506	10.4811	491.991	9.95873	491.267	10.08369
493.506	10.5831	492.991	10.0132	492.267	10.17855
494.506	10.67905	493.991	10.06223	493.267	10.26725
495.506	10.76698	494.991	10.10285	494.267	10.34953

496.506	10.84709	495.991	10.13624	495.267	10.42746
497.506	10.92095	496.991	10.16159	496.267	10.4986
498.506	10.98496	497.991	10.17942	497.267	10.56234
499.506	11.03967	498.991	10.18985	498.267	10.61916
500.506	11.08499	499.991	10.19257	499.267	10.66743
501.506	11.11943	500.991	10.18797	500.267	10.70731
502.506	11.14436	501.991	10.17578	501.267	10.73834
503.506	11.15814	502.991	10.15607	502.267	10.75994
504.506	11.16137	503.991	10.12832	503.267	10.77255
505.506	11.1532	504.991	10.09272	504.267	10.77601
506.506	11.13378	505.991	10.0497	505.267	10.77054
507.506	11.1037	506.991	9.99798	506.267	10.75623
508.506	11.0624	507.991	9.93764	507.267	10.73319
509.506	11.01157	508.991	9.86971	508.267	10.70147
510.506	10.95202	509.991	9.79505	509.267	10.66166
511.506	10.88302	510.991	9.71346	510.267	10.61229
512.506	10.80541	511.991	9.62622	511.267	10.55395
513.506	10.72082	512.991	9.53118	512.267	10.48588
514.506	10.62839	513.991	9.43277	513.267	10.40966
515.506	10.52793	514.991	9.32742	514.267	10.32744
516.506	10.42275	515.991	9.2175	515.267	10.23488
517.506	10.31009	516.991	9.1026	516.267	10.13921
518.506	10.19087	517.991	8.98275	517.267	10.03706
519.506	10.0663	518.991	8.85792	518.267	9.92996
520.506	9.93886	519.991	8.73114	519.267	9.81571
521.506	9.80408	520.991	8.60203	520.267	9.69886
522.506	9.66612	521.991	8.47196	521.267	9.57826
523.506	9.52486	522.991	8.33807	522.267	9.45358
524.506	9.37982	523.991	8.20643	523.267	9.32287
525.506	9.23192	524.991	8.07315	524.267	9.19034
526.506	9.08148	525.991	7.93959	525.267	9.05177
527.506	8.93185	526.991	7.80666	526.267	8.91225
528.506	8.77676	527.991	7.6709	527.267	8.766
529.506	8.61979	528.991	7.53889	528.267	8.62256
530.506	8.4633	529.991	7.40845	529.267	8.47309
531.506	8.30954	530.991	7.2753	530.267	8.32767
532.506	8.15833	531.991	7.14577	531.267	8.17822
533.506	8.00691	532.991	7.01721	532.267	8.02962
534.506	7.85926	533.991	6.8895	533.267	7.88175
535.506	7.71469	534.991	6.76294	534.267	7.73681
536.506	7.56973	535.991	6.63935	535.267	7.59234
537.506	7.43014	536.991	6.51697	536.267	7.45206
538.506	7.2949	537.991	6.3981	537.267	7.31281

539.506	7.1615	538.991	6.28097	538.267	7.17683
540.506	7.03294	539.991	6.16428	539.267	7.04423
541.506	6.90643	540.991	6.05171	540.267	6.91272
542.506	6.78396	541.991	5.94057	541.267	6.78465
543.506	6.66415	542.991	5.83214	542.267	6.65937
544.506	6.54689	543.991	5.72505	543.267	6.53309
545.506	6.43101	544.991	5.62101	544.267	6.41099
546.506	6.31782	545.991	5.51792	545.267	6.29104
547.506	6.20457	546.991	5.41738	546.267	6.17451
548.506	6.09191	547.991	5.31923	547.267	6.0593
549.506	5.982	548.991	5.22274	548.267	5.94505
550.506	5.87182	549.991	5.12965	549.267	5.83421
551.506	5.76115	550.991	5.0361	550.267	5.72645
552.506	5.65221	551.991	4.94319	551.267	5.61812
553.506	5.54538	552.991	4.85024	552.267	5.51212
554.506	5.43815	553.991	4.75703	553.267	5.40485
555.506	5.33329	554.991	4.66182	554.267	5.29973
556.506	5.22778	555.991	4.5666	555.267	5.19355
557.506	5.12088	556.991	4.46746	556.267	5.08891
558.506	5.01387	557.991	4.37082	557.267	4.98456
559.506	4.90536	558.991	4.27023	558.267	4.87668
560.506	4.79638	559.991	4.1709	559.267	4.77052
561.506	4.68489	560.991	4.0696	560.267	4.66033
562.506	4.5687	561.991	3.97008	561.267	4.55125
563.506	4.45415	562.991	3.87035	562.267	4.44049
564.506	4.3363	563.991	3.76836	563.267	4.32683
565.506	4.21659	564.991	3.66431	564.267	4.21169
566.506	4.09403	565.991	3.55875	565.267	4.09434
567.506	3.971	566.991	3.45239	566.267	3.97688
568.506	3.84605	567.991	3.34432	567.267	3.85608
569.506	3.72038	568.991	3.2362	568.267	3.73361
570.506	3.59216	569.991	3.12841	569.267	3.61014
571.506	3.46313	570.991	3.01778	570.267	3.48608
572.506	3.33355	571.991	2.90847	571.267	3.3616
573.506	3.20471	572.991	2.79778	572.267	3.2334
574.506	3.07467	573.991	2.68711	573.267	3.10567
575.506	2.94869	574.991	2.57757	574.267	2.97876
576.506	2.82161	575.991	2.4669	575.267	2.8493
577.506	2.69461	576.991	2.35981	576.267	2.72152
578.506	2.57282	577.991	2.25123	577.267	2.59495
579.506	2.4496	578.991	2.14589	578.267	2.46804
580.506	2.33145	579.991	2.04237	579.267	2.34052
581.506	2.22E+00	580.991	1.94152	580.267	2.21657

582.506	2.10292	581.991	1.84183	581.267	2.09531
583.506	1.99274	582.991	1.74491	582.267	1.9761
584.506	1.88368	583.991	1.65194	583.267	1.85917
585.506	1.78129	584.991	1.5619	584.267	1.74369
586.506	1.68149	585.991	1.47532	585.267	1.63313
587.506	1.59E+00	586.991	1.39102	586.267	1.52472
588.506	1.49109	587.991	1.31096	587.267	1.41968
589.506	1.40139	588.991	1.23438	588.267	1.31792
590.506	1.31511	589.991	1.16118	589.267	1.221
591.506	1.23325	590.991	1.09142	590.267	1.128
592.506	1.15444	591.991	1.0242	591.267	1.03831
593.506	1.07996	592.991	0.9608	592.267	0.95187
594.506	1.0098	593.991	0.90192	593.267	0.87143
595.506	0.94309	594.991	0.84479	594.267	0.79417
596.506	0.88006	595.991	0.79146	595.267	0.72119
597.506	0.82063	596.991	0.74183	596.267	0.65217
598.506	0.76605	597.991	0.6955	597.267	0.58815
599.506	0.71569	598.991	0.6528	598.267	0.52692
600.506	0.66752	599.991	0.6127	599.267	0.47069
601.506	0.62304	600.991	0.57616	600.267	0.41791
602.506	0.58282	601.991	0.54241	601.267	0.36925
603.506	0.54552	602.991	0.51144	602.267	0.32405
604.506	0.51163	603.991	0.48376	603.267	0.28287
605.506	0.48096	604.991	0.45726	604.267	2.45E-01
606.506	0.45291	605.991	0.43346	605.267	0.20966
607.506	0.4276	606.991	0.41203	606.267	0.17779
608.506	0.4051	607.991	0.3927	607.267	0.14885
609.506	0.38483	608.991	0.3752	608.267	0.12253
610.506	0.36645	609.991	0.35929	609.267	0.09934741
611.506	0.3495	610.991	0.34512	610.267	0.0778489
612.506	0.33413	611.991	0.33199	611.267	0.05846575
613.506	0.31991	612.991	0.32036	612.267	0.04115288
614.506	0.30676	613.991	0.30974	613.267	0.02532009
615.506	0.29462	614.991	0.3	614.267	0.0107369
616.506	0.283	615.991	0.29112	615.267	-0.002408004
617.506	0.27223	616.991	0.2829	616.267	-0.01464685
618.506	0.26247	617.991	0.27508	617.267	-0.02602981
619.506	0.25363	618.991	0.26801	618.267	-0.0363429
620.506	0.24603	619.991	0.26172	619.267	-0.0459662
621.506	0.23939	620.991	2.56E-01	620.267	-0.05475845
622.506	0.23362	621.991	2.50E-01	621.267	-0.06296462
623.506	0.22862	622.991	0.24533	622.267	-0.07070889
624.506	0.22463	623.991	0.2405	623.267	-0.07789367

625.506	0.22111	624.991	0.23619	624.267	-0.08440957
626.506	0.21811	625.991	0.23195	625.267	-0.09061524
627.506	0.21561	626.991	0.22791	626.267	-0.09638003
628.506	0.21319	627.991	0.22402	627.267	-0.10163
629.506	0.21097	628.991	0.22032	628.267	-0.10662
630.506	0.20885	629.991	0.21691	629.267	-0.11126
631.506	0.2068	630.991	0.21352	630.267	-0.1157
632.506	0.20487	631.991	0.21023	631.267	-0.11974
633.506	0.2032	632.991	0.20731	632.267	-0.12363
634.506	0.20164	633.991	0.20444	633.267	-0.12721
635.506	0.20008	634.991	0.20172	634.267	-0.13065
636.506	0.19857	635.991	0.19902	635.267	-0.13393
637.506	0.19731	636.991	0.19635	636.267	-0.13709
638.506	0.19608	637.991	0.19388	637.267	-0.13998
639.506	0.19456	638.991	0.19154	638.267	-0.14269
640.506	0.19297	639.991	1.89E-01	639.267	-0.14535
641.506	0.19145	640.991	1.87E-01	640.267	-0.14787
642.506	0.18995	641.991	0.18528	641.267	-0.15029
643.506	1.88E-01	642.991	0.18353	642.267	-0.15266
644.506	0.18715	643.991	0.18201	643.267	-0.15506
645.506	0.18579	644.991	0.18084	644.267	-0.15722
646.506	0.1779	645.991	0.17976	645.267	-0.15929
647.506	0.1531	646.991	0.17879	646.267	-0.16135
648.506	0.13262	647.991	0.17778	647.267	-0.16343
649.506	0.1136	648.991	0.17698	648.267	-0.16541
650.506	0.09561464	649.991	0.1762	649.267	-0.16736
651.506	0.07766708	650.991	0.17556	650.267	-0.16923
652.506	0.05992127	651.991	1.75E-01	651.267	-0.17103
653.506	0.04215663	652.991	0.17422	652.267	-0.17286
654.506	0.02849811	653.991	0.17348	653.267	-0.17476
655.506	0.03535201	654.991	0.17292	654.267	-0.17651
656.506	0.03792232	655.991	0.17251	655.267	-0.17821
657.506	0.03863653	656.991	0.17208	656.267	-0.17985
658.506	0.03861738	657.991	0.17167	657.267	-0.18148
659.506	0.03830298	658.991	0.17142	658.267	-0.18319
660.506	0.0379225	659.991	0.17107	659.267	-0.18462
661.506	0.03764057	660.991	0.17091	660.267	-0.18605
662.506	0.0373453	661.991	0.17068	661.267	-0.18756
663.506	0.03729107	662.991	0.17046	662.267	-0.18916
664.506	0.03732411	663.991	0.17057	663.267	-0.1906
665.506	0.0375285	664.991	1.71E-01	664.267	-0.192
666.506	0.0377482	665.991	0.17101	665.267	-0.19328
667.506	0.03807089	666.991	0.17119	666.267	-0.19459

668.506	0.03850824	667.991	0.17145	667.267	-0.19589
669.506	0.03894463	668.991	0.17178	668.267	-0.19711
670.506	0.03943975	669.991	0.17234	669.267	-0.19828
671.506	0.03989344	670.991	0.17305	670.267	-0.19933
672.506	0.04028952	671.991	0.17363	671.267	-0.20025
673.506	0.0406715	672.991	0.17419	672.267	-0.20113
674.506	0.04082991	673.991	0.17473	673.267	-0.2019
675.506	0.04109344	674.991	0.17524	674.267	-0.20267
676.506	0.04147397	675.991	0.17586	675.267	-0.20342
677.506	0.04199406	676.991	0.17657	676.267	-0.2042
678.506	0.04235645	677.991	0.17723	677.267	-0.20474
679.506	0.04272643	678.991	0.17802	678.267	-0.20537
680.506	0.04316308	679.991	0.17891	679.267	-2.06E-01
681.506	0.04375989	680.991	0.17991	680.267	-0.20658
682.506	0.04456775	681.991	0.18097	681.267	-0.20721
683.506	0.04533368	682.991	0.18218	682.267	-0.20788
684.506	0.04612308	683.991	0.18355	683.267	-0.20858
685.506	0.04681049	684.991	0.18507	684.267	-0.20906
686.506	0.04767074	685.991	0.18669	685.267	-0.2097
687.506	0.04851518	686.991	0.18816	686.267	-0.21013
688.506	0.04937986	687.991	0.18973	687.267	-0.21078
689.506	0.05022433	688.991	0.19126	688.267	-0.21131
690.506	0.05103471	689.991	0.19291	689.267	-0.2119
691.506	0.05193217	690.991	0.19465	690.267	-0.21251
692.506	0.05283982	691.991	0.19643	691.267	-0.21301
693.506	0.05370363	692.991	0.19809	692.267	-2.13E-01
694.506	0.05452661	693.991	0.19976	693.267	-0.21403
695.506	0.05527893	694.991	2.01E-01	694.267	-0.21456
696.506	0.0560165	695.991	0.2029	695.267	-0.21505
697.506	0.0567392	696.991	0.20459	696.267	-0.21563
698.506	5.76E-02	697.991	0.20621	697.267	-0.21609
699.506	0.05847846	698.991	0.20773	698.267	-0.21631
700.506	0.05920896	699.991	0.20926	699.267	-0.21643
701.506	0.05993969	700.991	0.21069	700.267	-0.21674
702.506	0.06070534	701.991	0.2121	701.267	-0.21707
703.506	0.06153433	702.991	0.21353	702.267	-0.2175
704.506	0.06246117	703.991	0.21493	703.267	-0.21765
705.506	0.0634146	704.991	2.16E-01	704.267	-0.2178
706.506	0.06433327	705.991	0.2176	705.267	-0.21808
707.506	0.06523502	706.991	0.21912	706.267	-0.21845
708.506	0.06607464	707.991	0.22055	707.267	-0.21887
709.506	0.06703363	708.991	0.22196	708.267	-0.21934
710.506	0.06793843	709.991	0.22339	709.267	-0.21977

711.506	0.0689888	710.991	0.22485	710.267	-0.22008
712.506	0.06994506	711.991	0.22646	711.267	-0.22046
713.506	0.0708672	712.991	0.22805	712.267	-0.22087
714.506	0.07164735	713.991	0.22965	713.267	-0.22128
715.506	0.07249815	714.991	0.23108	714.267	-0.22174
716.506	0.07331302	715.991	0.23245	715.267	-0.22232
717.506	0.07400573	716.991	0.23392	716.267	-0.22288
718.506	0.07462769	717.991	0.23528	717.267	-0.22324
719.506	0.07521977	718.991	0.23663	718.267	-0.22375
720.506	0.07588866	719.991	0.23799	719.267	-0.22448
721.506	0.07650569	720.991	0.23931	720.267	-0.22515
722.506	0.07711693	721.991	0.2405	721.267	-0.22576
723.506	0.07762969	722.991	0.2416	722.267	-0.22645
724.506	0.07836835	723.991	0.24295	723.267	-0.22714
725.506	0.07919438	724.991	0.24446	724.267	-0.22771
726.506	0.08013747	725.991	0.24597	725.267	-0.22843
727.506	8.09E-02	726.991	0.24772	726.267	-0.22909
728.506	0.08172756	727.991	0.24941	727.267	-0.2295
729.506	0.08263199	728.991	0.25123	728.267	-0.22993
730.506	0.0836514	729.991	0.25328	729.267	-0.23043
731.506	0.08467307	730.991	0.25543	730.267	-0.23082
732.506	0.0856235	731.991	0.25747	731.267	-0.2311
733.506	0.08633326	732.991	0.25923	732.267	-0.23149
734.506	0.08714675	733.991	0.26105	733.267	-0.23186
735.506	0.08790893	734.991	0.26262	734.267	-0.23208
736.506	0.08854863	735.991	0.26419	735.267	-0.23242
737.506	0.08904224	736.991	0.26546	736.267	-0.23281
738.506	0.08926154	737.991	0.26646	737.267	-0.23324
739.506	0.08941234	738.991	0.26737	738.267	-2.34E-01
740.506	8.96E-02	739.991	0.26831	739.267	-0.23432
741.506	0.08970339	740.991	0.26929	740.267	-2.35E-01
742.506	0.08975399	741.991	0.2703	741.267	-0.23524
743.506	0.08990421	742.991	0.27134	742.267	-0.23579
744.506	0.0900276	743.991	0.27223	743.267	-0.23649
745.506	0.09017584	744.991	2.73E-01	744.267	-0.23718
746.506	0.09042988	745.991	0.27437	745.267	-0.23777
747.506	0.0907789	746.991	0.27561	746.267	-0.23815
748.506	0.09098645	747.991	0.27663	747.267	-0.23851
749.506	0.09129453	748.991	0.27761	748.267	-0.23896
750.506	0.09167994	749.991	0.27857	749.267	-0.23929
751.506	0.09175307	750.991	0.27947	750.267	-2.40E-01
752.506	9.20E-02	751.991	0.28029	751.267	-0.23998
753.506	0.0920521	752.991	0.28116	752.267	-0.24041

754.506	0.09211357	753.991	0.28197	753.267	-0.24084
755.506	0.09215266	754.991	0.28264	754.267	-0.24163
756.506	0.09238246	755.991	0.28347	755.267	-0.24243
757.506	0.09259636	756.991	0.28425	756.267	-0.2432
758.506	0.0926789	757.991	0.28503	757.267	-0.24415
759.506	0.09296259	758.991	0.28578	758.267	-0.24521
760.506	0.09307286	759.991	0.28647	759.267	-0.24628
761.506	0.09331968	760.991	0.287	760.267	-0.24742
762.506	0.09357431	761.991	0.28753	761.267	-0.24876
763.506	0.09382273	762.991	0.28813	762.267	-0.25012
764.506	0.09379636	763.991	0.28871	763.267	-0.25144
765.506	0.09362342	764.991	0.28915	764.267	-0.25297
766.506	9.35E-02	765.991	0.28942	765.267	-0.25468
767.506	9.34E-02	766.991	0.28993	766.267	-0.25656
768.506	9.32E-02	767.991	0.29069	767.267	-0.25834
769.506	9.30E-02	768.991	0.29154	768.267	-0.25992
770.506	0.09259656	769.991	0.29246	769.267	-0.26164
771.506	0.09204642	770.991	0.29332	770.267	-0.26337
772.506	0.09165766	771.991	0.29408	771.267	-0.26514
773.506	9.15E-02	772.991	0.29487	772.267	-0.26687
774.506	0.09116963	773.991	0.29579	773.267	-0.26841
775.506	0.09071575	774.991	2.96E-01	774.267	-0.26995
776.506	0.09019548	775.991	2.97E-01	775.267	-0.27165
777.506	0.089674	776.991	0.29711	776.267	-0.27354
778.506	8.92E-02	777.991	0.29731	777.267	-0.27539
779.506	0.08877667	778.991	0.29763	778.267	-0.27722
780.506	0.08825129	779.991	0.29796	779.267	-0.27899
781.506	0.08762134	780.991	0.29823	780.267	-0.28073
782.506	0.08716438	781.991	0.29853	781.267	-0.28271
783.506	0.08678669	782.991	0.29895	782.267	-0.28447
784.506	0.08643106	783.991	0.29934	783.267	-0.28602
785.506	0.08613876	784.991	0.29971	784.267	-0.28753
786.506	0.08568272	785.991	0.29987	785.267	-0.2891
787.506	0.08521784	786.991	0.29997	786.267	-0.29075
788.506	0.08491098	787.991	0.3001	787.267	-0.29232
789.506	0.08472958	788.991	0.30048	788.267	-0.29398
790.506	0.08442518	789.991	0.30073	789.267	-0.29542
791.506	0.08402502	790.991	0.30074	790.267	-0.29711
792.506	0.08372565	791.991	0.30084	791.267	-0.29911
793.506	0.0836226	792.991	0.30103	792.267	-0.30112
794.506	0.08374073	793.991	0.30152	793.267	-0.30279
795.506	0.08366289	794.991	0.30174	794.267	-3.04E-01
796.506	0.083532	795.991	0.30172	795.267	-0.30644

796.991	0.30319	796.267	-0.30824
		797.267	-0.3084

temperature	*DSC*	*temperature*	*DSC*
℃	mW/mg	℃	mW/mg
	Coal+TPPI		Coal+PA
28.57	0.01305348	31.33	0.01487779
29.57	-0.01911443	32.33	-0.00422805
30.57	-0.03498374	33.33	-0.01911349
31.57	-0.03503613	34.33	-0.02871818
32.57	-0.03179253	35.33	-0.0308762
33.57	-0.02894751	36.33	-0.02894415
34.57	-0.02692779	37.33	-0.02619821
35.57	-0.02566529	38.33	-0.02364301
36.57	-0.0248827	39.33	-0.02179846
37.57	-0.02442732	40.33	-0.02037929
38.57	-0.02412143	41.33	-0.01925351
39.57	-0.0238807	42.33	-0.01828821
40.57	-0.02362957	43.33	-0.0173974
41.57	-0.02338544	44.33	-0.01643848
42.57	-0.02306205	45.33	-0.01550174
43.57	-0.022726	46.33	-0.01457588
44.57	-0.02232399	47.33	-0.01358392
45.57	-0.02187708	48.33	-0.01258842
46.57	-0.02142413	49.33	-0.01156882
47.57	-0.02089238	50.33	-0.01049459
48.57	-0.02023776	51.33	-0.00943486
49.57	-0.01952309	52.33	-0.00832693
50.57	-0.01883237	53.33	-0.00710057
51.57	-0.01804191	54.33	-0.00577637
52.57	-0.01721677	55.33	-0.00448176
53.57	-0.01626074	56.33	-0.00317213
54.57	-0.01527091	57.33	-0.00188884
55.57	-0.01416496	58.33	-0.00050209
56.57	-0.01312797	59.33	0.00092199
57.57	-0.01203231	60.33	0.00236244
58.57	-0.01094079	61.33	0.00386642
59.57	-0.009733536	62.33	0.00537313
60.57	-0.008502529	63.33	0.00681833
61.57	-0.007262382	64.33	0.00829879
62.57	-0.005991586	65.33	0.00987682
63.57	-0.004711326	66.33	0.01148548
64.57	-0.003400197	67.33	0.01308644
65.57	-0.001994429	68.33	0.01468571
66.57	-0.000558315	69.33	0.01632663
67.57	0.000933176	70.33	0.01792133

68.57	0.002428275	71.33	0.01948298
69.57	0.003935527	72.33	0.02105757
70.57	0.005511815	73.33	0.02274078
71.57	0.007028568	74.33	0.0244158
72.57	0.008564477	75.33	0.0261067
73.57	0.01020062	76.33	0.02783687
74.57	0.01180181	77.33	0.02954298
75.57	0.01342654	78.33	0.0313142
76.57	0.01510453	79.33	0.03310983
77.57	0.01676461	80.33	0.03498063
78.57	0.01847453	81.33	0.0369157
79.57	0.02025406	82.33	0.03879874
80.57	0.02209546	83.33	0.04074737
81.57	0.0239324	84.33	0.04263005
82.57	0.0258552	85.33	0.0446583
83.57	0.02778223	86.33	0.0466892
84.57	0.02976729	87.33	0.04871832
85.57	0.03180624	88.33	0.05078048
86.57	0.03389201	89.33	0.05290903
87.57	0.03599426	90.33	0.05498787
88.57	0.03824144	91.33	0.0571468
89.57	0.04047821	92.33	0.05931671
90.57	0.04275398	93.33	0.06158574
91.57	0.04515546	94.33	0.06382735
92.57	0.04761015	95.33	0.06612821
93.57	0.05008188	96.33	0.06850056
94.57	0.05257688	97.33	0.07086162
95.57	0.05516077	98.33	0.07320764
96.57	0.0577333	99.33	0.07565167
97.57	0.06035742	100.33	0.0780639
98.57	0.06293137	101.33	0.0805297
99.57	0.06556688	102.33	0.08299707
100.57	0.06817391	103.33	0.08549609
101.57	0.0709802	104.33	0.08795593
102.57	0.07357022	105.33	0.0904546
103.57	0.07626675	106.33	0.09298184
104.57	0.07899647	107.33	0.09553138
105.57	0.08177666	108.33	0.09815077
106.57	0.08462766	109.33	0.10074
107.57	0.08746564	110.33	0.10343
108.57	0.09041666	111.33	0.10605
109.57	0.09348081	112.33	0.10875
110.57	0.09652936	113.33	0.11149

111.57	0.099734	114.33	0.11428
112.57	0.10289	115.33	0.11711
113.57	0.10613	116.33	0.11994
114.57	0.10954	117.33	0.12273
115.57	0.11289	118.33	0.12561
116.57	0.11636	119.33	0.12857
117.57	0.11982	120.33	0.13155
118.57	0.12335	121.33	0.13451
119.57	0.12692	122.33	0.13748
120.57	0.13048	123.33	0.14058
121.57	0.13417	124.33	0.14362
122.57	0.13776	125.33	0.14674
123.57	0.14141	126.33	0.14992
124.57	0.14508	127.33	0.15311
125.57	0.14888	128.33	0.15634
126.57	0.15272	129.33	0.1596
127.57	0.1566	130.33	0.16293
128.57	0.16049	131.33	0.16627
129.57	0.16446	132.33	0.16949
130.57	0.1685	133.33	0.17283
131.57	0.17243	134.33	0.17623
132.57	0.17648	135.33	0.17969
133.57	0.18056	136.33	0.18302
134.57	0.18465	137.33	0.18641
135.57	0.18879	138.33	0.1898
136.57	0.19288	139.33	0.19321
137.57	0.19699	140.33	0.19661
138.57	0.20114	141.33	0.20015
139.57	0.20533	142.33	0.20361
140.57	0.20948	143.33	0.20711
141.57	0.21382	144.33	0.21056
142.57	0.21802	145.33	0.21415
143.57	0.22233	146.33	0.21771
144.57	0.22673	147.33	0.22144
145.57	0.23103	148.33	0.22517
146.57	0.23555	149.33	0.22902
147.57	0.24012	150.33	0.23291
148.57	0.24478	151.33	0.23683
149.57	0.24955	152.33	0.24103
150.57	0.25436	153.33	0.24505
151.57	0.2592	154.33	0.24928
152.57	0.26418	155.33	0.25353
153.57	0.26916	156.33	0.25779

154.57	0.27433	157.33	0.26209
155.57	0.27937	158.33	0.26643
156.57	0.28458	159.33	0.27092
157.57	0.2898	160.33	0.27532
158.57	0.29492	161.33	0.27987
159.57	0.3002	162.33	0.28444
160.57	0.30554	163.33	0.28906
161.57	0.31103	164.33	0.29367
162.57	0.31645	165.33	0.2984
163.57	0.32208	166.33	0.30315
164.57	0.32785	167.33	0.30802
165.57	0.33356	168.33	0.31293
166.57	0.3394	169.33	0.318
167.57	0.34535	170.33	0.3229
168.57	0.35146	171.33	0.32793
169.57	0.35751	172.33	0.33296
170.57	0.36361	173.33	0.33817
171.57	0.36975	174.33	0.34321
172.57	0.37586	175.33	0.34841
173.57	0.38215	176.33	0.3535
174.57	0.38829	177.33	0.35881
175.57	0.39456	178.33	0.36421
176.57	0.40067	179.33	0.36973
177.57	0.40698	180.33	0.37519
178.57	0.41317	181.33	0.38085
179.57	0.41964	182.33	0.38649
180.57	0.42603	183.33	0.39222
181.57	0.43243	184.33	0.3981
182.57	0.43895	185.33	0.40386
183.57	0.44548	186.33	0.4098
184.57	0.45195	187.33	0.4157
185.57	0.45863	188.33	0.4217
186.57	0.4652	189.33	0.42778
187.57	0.47181	190.33	0.43389
188.57	0.4786	191.33	0.44009
189.57	0.48531	192.33	0.44632
190.57	0.49223	193.33	0.4526
191.57	0.4991	194.33	0.45895
192.57	0.50592	195.33	0.46546
193.57	0.51277	196.33	0.47189
194.57	0.51971	197.33	0.47844
195.57	0.52655	198.33	0.48506
196.57	0.53336	199.33	0.49193

197.57	0.54017	200.33	0.49873
198.57	0.54713	201.33	0.50573
199.57	0.55391	202.33	0.5129
200.57	0.56074	203.33	0.52014
201.57	0.56774	204.33	0.5275
202.57	0.57486	205.33	0.53515
203.57	0.5822	206.33	0.54276
204.57	0.58946	207.33	0.55053
205.57	0.597	208.33	0.55852
206.57	0.60473	209.33	0.56634
207.57	0.61262	210.33	0.57461
208.57	0.6206	211.33	0.58276
209.57	0.62888	212.33	0.59095
210.57	0.6374	213.33	0.59942
211.57	0.64591	214.33	0.60802
212.57	0.65466	215.33	0.61677
213.57	0.66359	216.33	0.6256
214.57	0.67247	217.33	0.63456
215.57	0.68161	218.33	0.64374
216.57	0.69062	219.33	0.65284
217.57	0.69972	220.33	0.66227
218.57	0.70898	221.33	0.67188
219.57	0.71814	222.33	0.6814
220.57	0.72748	223.33	0.69128
221.57	0.73699	224.33	0.7011
222.57	0.74652	225.33	0.71142
223.57	0.7561	226.33	0.72161
224.57	0.7661	227.33	0.73205
225.57	0.77613	228.33	0.74255
226.57	0.78636	229.33	0.75324
227.57	0.7967	230.33	0.76438
228.57	0.80725	231.33	0.77546
229.57	0.818	232.33	0.78676
230.57	0.82877	233.33	0.79834
231.57	0.83992	234.33	0.81036
232.57	0.85141	235.33	0.82241
233.57	0.86293	236.33	0.83492
234.57	0.87475	237.33	0.84734
235.57	0.8865	238.33	0.86008
236.57	0.89858	239.33	0.87324
237.57	0.91081	240.33	0.88661
238.57	0.92342	241.33	0.90002
239.57	0.93596	242.33	0.91386

240.57	0.9489	243.33	0.92804
241.57	0.96187	244.33	0.94244
242.57	0.97481	245.33	0.95685
243.57	0.98824	246.33	0.97179
244.57	1.00177	247.33	0.98677
245.57	1.01547	248.33	1.00257
246.57	1.02911	249.33	1.01832
247.57	1.04299	250.33	1.03465
248.57	1.05718	251.33	1.05129
249.57	1.07135	252.33	1.06828
250.57	1.08631	253.33	1.08584
251.57	1.10104	254.33	1.10419
252.57	1.1164	255.33	1.12247
253.57	1.13183	256.33	1.14151
254.57	1.14757	257.33	1.16119
255.57	1.16374	258.33	1.18113
256.57	1.18023	259.33	1.20168
257.57	1.19716	260.33	1.22268
258.57	1.21449	261.33	1.24381
259.57	1.23196	262.33	1.26573
260.57	1.25007	263.33	1.28759
261.57	1.26827	264.33	1.31024
262.57	1.2871	265.33	1.33285
263.57	1.30639	266.33	1.35615
264.57	1.3256	267.33	1.37926
265.57	1.34563	268.33	1.40273
266.57	1.3658	269.33	1.42635
267.57	1.3865	270.33	1.45046
268.57	1.40754	271.33	1.47445
269.57	1.42895	272.33	1.49872
270.57	1.45058	273.33	1.52307
271.57	1.47268	274.33	1.54781
272.57	1.49534	275.33	1.57289
273.57	1.51851	276.33	1.5982
274.57	1.54228	277.33	1.62349
275.57	1.56633	278.33	1.649
276.57	1.59086	279.33	1.67514
277.57	1.61588	280.33	1.70145
278.57	1.6415	281.33	1.72788
279.57	1.66778	282.33	1.75465
280.57	1.69466	283.33	1.78184
281.57	1.7219	284.33	1.80965
282.57	1.74984	285.33	1.83765

283.57	1.77857	286.33	1.86648
284.57	1.80758	287.33	1.89525
285.57	1.8376	288.33	1.92466
286.57	1.86783	289.33	1.95426
287.57	1.899	290.33	1.98466
288.57	1.93052	291.33	2.01569
289.57	1.96223	292.33	2.04687
290.57	1.99477	293.33	2.07822
291.57	2.02691	294.33	2.10981
292.57	2.05997	295.33	2.14197
293.57	2.09328	296.33	2.17411
294.57	2.12706	297.33	2.20658
295.57	2.16108	298.33	2.23859
296.57	2.19566	299.33	2.2716
297.57	2.22984	300.33	2.30328
298.57	2.26452	301.33	2.33475
299.57	2.29929	302.33	2.36667
300.57	2.33407	303.33	2.39853
301.57	2.36932	304.33	2.4297
302.57	2.40431	305.33	2.4608
303.57	2.43933	306.33	2.492
304.57	2.47448	307.33	2.522
305.57	2.50933	308.33	2.55198
306.57	2.54387	309.33	2.58174
307.57	2.5781	310.33	2.61087
308.57	2.61279	311.33	2.63923
309.57	2.64589	312.33	2.66705
310.57	2.67966	313.33	2.69406
311.57	2.71206	314.33	2.72089
312.57	2.74393	315.33	2.74684
313.57	2.7749	316.33	2.77153
314.57	2.80496	317.33	2.79586
315.57	2.83435	318.33	2.81903
316.57	2.86261	319.33	2.84125
317.57	2.8897	320.33	2.86264
318.57	2.91545	321.33	2.8833
319.57	2.94016	322.33	2.90276
320.57	2.96371	323.33	2.92191
321.57	2.98638	324.33	2.94005
322.57	3.00808	325.33	2.95736
323.57	3.02847	326.33	2.97414
324.57	3.0483	327.33	2.99003
325.57	3.06708	328.33	3.00527

326.57	3.08535	329.33	3.01958
327.57	3.10287	330.33	3.03334
328.57	3.11969	331.33	3.04653
329.57	3.13575	332.33	3.05904
330.57	3.15077	333.33	3.07078
331.57	3.16534	334.33	3.08193
332.57	3.17896	335.33	3.09249
333.57	3.19188	336.33	3.10241
334.57	3.20421	337.33	3.11212
335.57	3.2155	338.33	3.12155
336.57	3.22632	339.33	3.13058
337.57	3.23651	340.33	3.13937
338.57	3.24631	341.33	3.14783
339.57	3.25561	342.33	3.15609
340.57	3.26474	343.33	3.16437
341.57	3.27344	344.33	3.17247
342.57	3.28199	345.33	3.18027
343.57	3.29052	346.33	3.18798
344.57	3.29857	347.33	3.19549
345.57	3.30662	348.33	3.20295
346.57	3.31442	349.33	3.2105
347.57	3.32212	350.33	3.218
348.57	3.32981	351.33	3.22565
349.57	3.33747	352.33	3.23326
350.57	3.34516	353.33	3.2411
351.57	3.35295	354.33	3.24921
352.57	3.36082	355.33	3.2576
353.57	3.36858	356.33	3.2663
354.57	3.37664	357.33	3.2754
355.57	3.38438	358.33	3.2848
356.57	3.3924	359.33	3.29455
357.57	3.40013	360.33	3.30445
358.57	3.40807	361.33	3.31478
359.57	3.41609	362.33	3.32503
360.57	3.42423	363.33	3.33529
361.57	3.43246	364.33	3.34557
362.57	3.441	365.33	3.3558
363.57	3.44973	366.33	3.36587
364.57	3.45871	367.33	3.37602
365.57	3.46802	368.33	3.38617
366.57	3.47757	369.33	3.39645
367.57	3.48726	370.33	3.40695
368.57	3.49703	371.33	3.41772

369.57	3.50693	372.33	3.42875
370.57	3.51696	373.33	3.44019
371.57	3.52724	374.33	3.45182
372.57	3.53731	375.33	3.4631
373.57	3.54757	376.33	3.47511
374.57	3.55778	377.33	3.48653
375.57	3.5678	378.33	3.4982
376.57	3.57784	379.33	3.50959
377.57	3.5875	380.33	3.52092
378.57	3.59693	381.33	3.53223
379.57	3.60626	382.33	3.5432
380.57	3.61507	383.33	3.55483
381.57	3.62371	384.33	3.56601
382.57	3.63194	385.33	3.57733
383.57	3.64012	386.33	3.58842
384.57	3.64813	387.33	3.59923
385.57	3.6563	388.33	3.61007
386.57	3.66439	389.33	3.62045
387.57	3.67298	390.33	3.63089
388.57	3.68166	391.33	3.64107
389.57	3.69086	392.33	3.65131
390.57	3.70026	393.33	3.66146
391.57	3.70984	394.33	3.672
392.57	3.71973	395.33	3.68267
393.57	3.72967	396.33	3.69377
394.57	3.73961	397.33	3.70509
395.57	3.74969	398.33	3.71649
396.57	3.75972	399.33	3.72824
397.57	3.76995	400.33	3.74004
398.57	3.78044	401.33	3.75206
399.57	3.79097	402.33	3.76429
400.57	3.80205	403.33	3.77657
401.57	3.81387	404.33	3.78921
402.57	3.82599	405.33	3.80207
403.57	3.8387	406.33	3.81571
404.57	3.85192	407.33	3.82959
405.57	3.86571	408.33	3.84428
406.57	3.88017	409.33	3.8599
407.57	3.89492	410.33	3.87661
408.57	3.91049	411.33	3.8937
409.57	3.92655	412.33	3.9123
410.57	3.9431	413.33	3.93131
411.57	3.96013	414.33	3.95103

412.57	3.97747	415.33	3.97147
413.57	3.99547	416.33	3.99229
414.57	4.01374	417.33	4.0136
415.57	4.03266	418.33	4.03513
416.57	4.05153	419.33	4.05711
417.57	4.07117	420.33	4.0794
418.57	4.0912	421.33	4.10209
419.57	4.11243	422.33	4.12559
420.57	4.13405	423.33	4.14998
421.57	4.1567	424.33	4.17523
422.57	4.18037	425.33	4.2012
423.57	4.20517	426.33	4.22733
424.57	4.23133	427.33	4.25487
425.57	4.2584	428.33	4.28318
426.57	4.28672	429.33	4.31315
427.57	4.31606	430.33	4.34358
428.57	4.34662	431.33	4.37549
429.57	4.37772	432.33	4.4082
430.57	4.41057	433.33	4.44205
431.57	4.44424	434.33	4.47755
432.57	4.47894	435.33	4.51483
433.57	4.51446	436.33	4.55297
434.57	4.55156	437.33	4.59238
435.57	4.58977	438.33	4.63279
436.57	4.62845	439.33	4.67421
437.57	4.66796	440.33	4.71677
438.57	4.70891	441.33	4.76147
439.57	4.75051	442.33	4.80714
440.57	4.79262	443.33	4.85422
441.57	4.8367	444.33	4.90239
442.57	4.88135	445.33	4.9521
443.57	4.92743	446.33	5.00344
444.57	4.9749	447.33	5.05703
445.57	5.02363	448.33	5.11168
446.57	5.07426	449.33	5.16821
447.57	5.12693	450.33	5.22676
448.57	5.18052	451.33	5.28686
449.57	5.23724	452.33	5.34951
450.57	5.29561	453.33	5.41411
451.57	5.35507	454.33	5.48006
452.57	5.41717	455.33	5.54967
453.57	5.48116	456.33	5.61958
454.57	5.54831	457.33	5.69218

455.57	5.617	458.33	5.76801
456.57	5.68767	459.33	5.8468
457.57	5.76253	460.33	5.92812
458.57	5.83832	461.33	6.01357
459.57	5.91647	462.33	6.09891
460.57	5.99634	463.33	6.18819
461.57	6.07811	464.33	6.27988
462.57	6.16154	465.33	6.37195
463.57	6.24789	466.33	6.46689
464.57	6.33552	467.33	6.56746
465.57	6.42473	468.33	6.66851
466.57	6.51496	469.33	6.77142
467.57	6.6073	470.33	6.87731
468.57	6.70237	471.33	6.98563
469.57	6.79918	472.33	7.0948
470.57	6.89532	473.33	7.20881
471.57	6.99757	474.33	7.3254
472.57	7.10008	475.33	7.44216
473.57	7.20694	476.33	7.5588
474.57	7.31409	477.33	7.67788
475.57	7.42288	478.33	7.7975
476.57	7.53	479.33	7.91557
477.57	7.64283	480.33	8.03463
478.57	7.75391	481.33	8.15332
479.57	7.86342	482.33	8.27036
480.57	7.97429	483.33	8.38955
481.57	8.0853	484.33	8.50495
482.57	8.19234	485.33	8.61764
483.57	8.30134	486.33	8.73034
484.57	8.40892	487.33	8.83772
485.57	8.51697	488.33	8.9449
486.57	8.62362	489.33	9.0487
487.57	8.72738	490.33	9.15115
488.57	8.83064	491.33	9.25064
489.57	8.93161	492.33	9.34758
490.57	9.03257	493.33	9.44135
491.57	9.12858	494.33	9.53001
492.57	9.22524	495.33	9.61418
493.57	9.31778	496.33	9.69312
494.57	9.40567	497.33	9.76523
495.57	9.48833	498.33	9.83146
496.57	9.56448	499.33	9.88966
497.57	9.63524	500.33	9.94177

498.57	9.69573	501.33	9.98646
499.57	9.74864	502.33	10.02262
500.57	9.79327	503.33	10.05151
501.57	9.82719	504.33	10.0723
502.57	9.8518	505.33	10.0857
503.57	9.86718	506.33	10.09125
504.57	9.87355	507.33	10.08842
505.57	9.87182	508.33	10.07744
506.57	9.86226	509.33	10.05825
507.57	9.84497	510.33	10.03053
508.57	9.82	511.33	9.9954
509.57	9.78763	512.33	9.95369
510.57	9.74833	513.33	9.9051
511.57	9.7016	514.33	9.85133
512.57	9.64688	515.33	9.79125
513.57	9.58601	516.33	9.72519
514.57	9.5187	517.33	9.65545
515.57	9.44472	518.33	9.58175
516.57	9.36624	519.33	9.50425
517.57	9.28278	520.33	9.42501
518.57	9.19446	521.33	9.34141
519.57	9.1037	522.33	9.25496
520.57	9.00686	523.33	9.16677
521.57	8.908	524.33	9.07462
522.57	8.80499	525.33	8.97981
523.57	8.69921	526.33	8.88134
524.57	8.5904	527.33	8.78457
525.57	8.47905	528.33	8.68281
526.57	8.36721	529.33	8.58155
527.57	8.25509	530.33	8.47957
528.57	8.14065	531.33	8.3807
529.57	8.02829	532.33	8.28098
530.57	7.91744	533.33	8.18541
531.57	7.80921	534.33	8.09138
532.57	7.70368	535.33	7.99843
533.57	7.60079	536.33	7.90702
534.57	7.50057	537.33	7.81825
535.57	7.40319	538.33	7.72919
536.57	7.30819	539.33	7.64149
537.57	7.21606	540.33	7.5532
538.57	7.12734	541.33	7.46436
539.57	7.03881	542.33	7.37511
540.57	6.95248	543.33	7.28606

541.57	6.86655	544.33	7.19611
542.57	6.78448	545.33	7.10651
543.57	6.70185	546.33	7.01864
544.57	6.62058	547.33	6.93101
545.57	6.53947	548.33	6.84362
546.57	6.45804	549.33	6.75742
547.57	6.37712	550.33	6.67037
548.57	6.29513	551.33	6.58327
549.57	6.21246	552.33	6.49458
550.57	6.13066	553.33	6.40442
551.57	6.04683	554.33	6.31228
552.57	5.96138	555.33	6.21977
553.57	5.87622	556.33	6.1228
554.57	5.78965	557.33	6.02491
555.57	5.70145	558.33	5.92409
556.57	5.6118	559.33	5.82213
557.57	5.52118	560.33	5.71736
558.57	5.42808	561.33	5.61097
559.57	5.33444	562.33	5.50108
560.57	5.23857	563.33	5.38763
561.57	5.13912	564.33	5.27231
562.57	5.03874	565.33	5.15483
563.57	4.93588	566.33	5.03521
564.57	4.82887	567.33	4.91342
565.57	4.72047	568.33	4.78921
566.57	4.60942	569.33	4.66089
567.57	4.49489	570.33	4.52954
568.57	4.37977	571.33	4.39912
569.57	4.2639	572.33	4.26616
570.57	4.14328	573.33	4.12975
571.57	4.02412	574.33	3.99087
572.57	3.90279	575.33	3.85192
573.57	3.78032	576.33	3.71329
574.57	3.65846	577.33	3.57453
575.57	3.533	578.33	3.4347
576.57	3.41083	579.33	3.29562
577.57	3.28712	580.33	3.15839
578.57	3.16392	581.33	3.02299
579.57	3.04244	582.33	2.89066
580.57	2.92181	583.33	2.75783
581.57	2.80094	584.33	2.62822
582.57	2.68287	585.33	2.50231
5.84E+02	2.56573	586.33	2.37739

584.57	2.45128	587.33	2.25763
585.57	2.33794	588.33	2.14081
586.57	2.22759	589.33	2.02857
587.57	2.12168	590.33	1.91909
588.57	2.01941	591.33	1.8157
589.57	1.92033	592.33	1.71523
590.57	1.82373	593.33	1.61857
591.57	1.73279	594.33	1.527
592.57	1.64454	595.33	1.43934
593.57	1.56016	596.33	1.35663
594.57	1.48029	597.33	1.27829
595.57	1.40383	598.33	1.20451
596.57	1.33054	599.33	1.13474
597.57	1.26254	600.33	1.06952
598.57	1.19755	601.33	1.00683
599.57	1.13597	602.33	0.94925
600.57	1.07754	603.33	0.89436
601.57	1.02287	604.33	0.84295
602.57	0.97146	605.33	0.79582
603.57	0.92315	606.33	0.75073
604.57	0.87691	607.33	0.7092
605.57	0.83422	608.33	0.66945
606.57	0.79368	609.33	0.63286
607.57	0.75571	610.33	0.59797
608.57	0.71953	611.33	0.56577
609.57	0.68681	612.33	0.53543
610.57	0.65495	613.33	0.50629
611.57	0.62521	614.33	0.47964
612.57	0.59718	615.33	0.45429
613.57	0.57039	616.33	0.42986
614.57	0.54541	617.33	0.40688
615.57	0.52198	618.33	0.38522
616.57	0.49964	619.33	0.36438
617.57	0.4787	620.33	0.34498
618.57	0.45885	621.33	0.32617
619.57	0.44033	622.33	0.30807
620.57	0.42274	623.33	0.29069
621.57	0.40595	624.33	0.27417
622.57	0.38997	625.33	0.25774
623.57	0.37454	626.33	0.24242
624.57	0.35979	627.33	0.2275
625.57	0.34571	628.33	0.21296
626.57	0.33224	629.33	0.19874

627.57	0.31912	630.33	0.1852
628.57	0.30626	631.33	0.17153
629.57	0.29349	632.33	0.1584
630.57	0.28145	633.33	0.14579
631.57	0.26963	634.33	0.13366
632.57	0.25859	635.33	0.12154
633.57	0.24773	636.33	0.10988
634.57	0.23716	637.33	0.09848219
635.57	0.22701	638.33	0.08771902
636.57	0.21687	639.33	0.07696045
637.57	0.20716	640.33	0.06667091
638.57	0.19784	641.33	0.0563237
639.57	0.18861	642.33	0.046376
640.57	0.17956	643.33	0.03659406
641.57	0.17095	644.33	0.02738817
642.57	0.16258	645.33	0.01838529
643.57	0.15433	646.33	0.00966363
644.57	0.1464	647.33	0.00128184
645.57	0.1389	648.33	-0.00696747
646.57	0.13156	649.33	-0.01476507
647.57	0.12459	650.33	-0.02226879
648.57	0.11788	651.33	-0.02943189
649.57	0.11145	652.33	-0.03659896
650.57	0.10537	6.53E+02	-0.04367951
651.57	0.09988842	654.33	-0.05037853
652.57	0.09462697	655.33	-0.05672538
653.57	0.08960972	656.33	-0.06274742
654.57	0.08470058	657.33	-0.06868066
655.57	0.08005153	658.33	-0.07437794
656.57	0.0756763	659.33	-0.07993101
657.57	0.07119708	660.33	-0.08541874
658.57	0.06688987	661.33	-0.09074425
659.57	0.06244809	662.33	-0.09592538
660.57	0.05810785	663.33	-0.10092
661.57	0.05393806	664.33	-0.10584
662.57	0.04975533	665.33	-0.11077
663.57	0.04581403	666.33	-0.11555
664.57	0.04194612	667.33	-0.12051
665.57	0.03812381	668.33	-0.12534
666.57	0.0341624	669.33	-0.13006
667.57	0.03034238	670.33	-0.1346
668.57	0.02661532	671.33	-0.13912
669.57	0.02281318	672.33	-0.14359

670.57	0.0192013	673.33	-0.14793
671.57	0.01548649	674.33	-0.15225
672.57	0.01166883	675.33	-0.15642
673.57	0.007960826	676.33	-0.16073
674.57	0.003850635	677.33	-0.16495
675.57	-0.000192766	678.33	-0.16935
676.57	-0.004259276	679.33	-0.17367
677.57	-0.008045224	680.33	-0.17777
678.57	-0.01184573	681.33	-0.182
679.57	-0.01548113	682.33	-0.18616
680.57	-0.01908846	683.33	-0.19031
681.57	-0.02260612	684.33	-0.19426
682.57	-0.02592018	685.33	-0.19829
683.57	-0.02910564	686.33	-0.20227
684.57	-0.03227197	687.33	-0.20624
685.57	-0.03558428	688.33	-0.21014
686.57	-0.03895845	6.89E+02	-0.21392
687.57	-0.04244595	690.33	-0.21769
688.57	-0.04591134	691.33	-0.22144
689.57	-0.04927293	692.33	-0.22517
690.57	-0.0526612	693.33	-0.22875
691.57	-0.05594011	6.94E+02	-0.23224
692.57	-0.05916395	695.33	-0.23582
693.57	-0.06240206	696.33	-0.2396
694.57	-0.06556431	697.33	-0.24328
695.57	-0.06879317	698.33	-0.24689
696.57	-0.07198983	6.99E+02	-0.25048
697.57	-0.07503322	700.33	-0.25409
698.57	-0.07797446	701.33	-0.25779
699.57	-0.08087459	702.33	-0.2614
700.57	-0.08398912	703.33	-0.26472
701.57	-0.08692867	704.33	-0.26776
702.57	-0.08984973	705.33	-0.27081
703.57	-0.09254218	706.33	-0.27398
704.57	-0.09512321	707.33	-0.27722
705.57	-0.09793046	708.33	-0.28044
706.57	-0.10054	709.33	-0.28363
707.57	-0.103	710.33	-0.28676
708.57	-0.10541	711.33	-0.28978
709.57	-0.10795	712.33	-0.29309
710.57	-0.1106	713.33	-0.29667
711.57	-0.11331	714.33	-0.3002
712.57	-0.11621	715.33	-0.30375

713.57	-0.11893	716.33	-0.30738
714.57	-0.12192	717.33	-0.31098
715.57	-0.12517	718.33	-0.31469
716.57	-0.12859	719.33	-0.31853
717.57	-0.13194	720.33	-0.32207
718.57	-0.13524	721.33	-0.32558
719.57	-0.13852	7.22E+02	-0.3292
720.57	-0.14169	723.33	-0.33268
721.57	-0.14492	724.33	-0.33595
722.57	-0.14813	725.33	-0.33919
723.57	-0.15111	726.33	-0.34239
724.57	-0.15396	727.33	-0.34561
725.57	-0.15692	728.33	-0.34896
726.57	-0.15975	729.33	-0.35226
727.57	-0.16257	730.33	-0.35516
728.57	-0.1654	731.33	-0.35801
729.57	-0.16818	732.33	-0.36091
730.57	-0.17079	733.33	-0.36378
731.57	-0.17341	7.34E+02	-0.36645
732.57	-0.17615	735.33	-0.369
733.57	-0.179	736.33	-0.37145
734.57	-0.18192	737.33	-0.37407
735.57	-0.18478	7.38E+02	-0.37699
736.57	-0.18781	739.33	-0.37989
737.57	-0.19074	740.33	-0.3824
738.57	-0.19393	7.41E+02	-0.38471
739.57	-0.19719	742.33	-0.38704
740.57	-0.20032	743.33	-0.38938
741.57	-0.20325	744.33	-0.39163
742.57	-0.20612	745.33	-0.39361
743.57	-0.20886	746.33	-0.39526
744.57	-0.21142	747.33	-0.39684
745.57	-0.21394	748.33	-0.39854
746.57	-0.21621	749.33	-0.40018
747.57	-0.21844	750.33	-0.4018
748.57	-0.22061	7.51E+02	-0.40337
749.57	-0.22247	752.33	-0.40495
750.57	-0.22413	753.33	-0.40642
751.57	-0.22598	7.54E+02	-0.40795
752.57	-0.22776	755.33	-0.40926
7.54E+02	-0.22943	756.33	-0.4104
754.57	-0.23119	757.33	-0.41174
755.57	-0.2328	758.33	-0.41312

756.57	-0.2343	759.33	-0.41446
757.57	-0.23593	760.33	-0.41586
758.57	-0.23758	761.33	-0.41734
759.57	-0.23909	762.33	-0.41876
760.57	-0.24072	763.33	-0.42024
761.57	-0.24242	764.33	-0.42129
762.57	-0.24395	7.65E+02	-0.42223
763.57	-0.2455	766.33	-0.42305
764.57	-0.24697	767.33	-0.4238
765.57	-0.24849	768.33	-0.4243
766.57	-0.24991	769.33	-0.42465
767.57	-0.25095	770.33	-0.42508
768.57	-0.25185	771.33	-0.42544
769.57	-0.25271	772.33	-0.42652
770.57	-0.25351	773.33	-0.42745
771.57	-0.25417	774.33	-0.42858
772.57	-0.25475	775.33	-0.42977
773.57	-0.25512	776.33	-0.43118
774.57	-0.25582	777.33	-0.43269
775.57	-0.25668	778.33	-0.43419
776.57	-0.25772	779.33	-0.43572
777.57	-0.25889	780.33	-0.4369
778.57	-0.26019	781.33	-0.43825
779.57	-0.26155	782.33	-0.43947
780.57	-0.26302	783.33	-0.44052
781.57	-0.26448	784.33	-0.44136
782.57	-0.26561	785.33	-0.44221
783.57	-0.26672	786.33	-0.44304
784.57	-0.26772	787.33	-0.444
785.57	-0.26867	788.33	-0.44516
786.57	-0.2695	789.33	-0.44622
787.57	-0.27031	790.33	-0.44726
788.57	-0.27089	791.33	-0.44833
789.57	-0.27166	792.33	-0.44951
790.57	-0.27264	7.93E+02	-0.45051
791.57	-0.2739	794.33	-0.4514
792.57	-0.27512	795.33	-0.45214
793.57	-0.27608	7.96E+02	-0.45277
794.57	-0.27705	797.33	-0.45253
795.57	-0.27877		
796.57	-0.27985		

Fitting results of functional group peak area of water-immersed brown coal after adding inhibitors

	Coal	Coal+MgCl2	Coal+TEMPO	Coal+TPPI	Coal+PA
A	15.6837	8.77311	13.90633	34.12923	18.78033
B	55.67533	62.27562	54.62574	39.12424	48.80427
C	17.81735	13.93079	18.51645	18.20266	20.34377
D	10.82362	15.02048	12.95147	8.54386	12.07163
E	11.98714	15.58739	11.8069	12.68244	12.01585
F	15.84243	16.65908	16.32014	15.9578	17.70915
G	21.14509	17.11385	20.85371	25.98046	18.24164
H	4.34658	4.25978	4.59593	6.33448	3.16124
I	7.16092	4.71784	6.01887	8.39895	6.14573
J	1.42606	1.77968	2.4498	3.12574	2.14186
K	25.02002	22.41775	22.01647	17.79196	22.03717
L	12.07176	16.46463	15.93818	12.02817	18.54735
M	20.86445	19.20625	19.36748	17.89123	18.7068
N	10.16799	9.80992	8.99256	7.61891	8.66803
O	13.99435	18.78494	12.41506	11.35625	15.04539
P	37.60633	34.05968	41.07331	38.92269	37.07519
Q	19.36688	18.13921	18.15159	18.25714	20.50458
R	2.25644	2.19329	1.64115	1.9551	1.67103
S	7.06566	9.01865	7.25176	6.9621	7.16916
T	14.54684	16.46884	12.46181	13.15332	13.71345
U	30.72892	35.36475	33.27796	32.72136	30.79905
V	45.40214	36.95447	45.36731	45.20812	46.64731

Fitting results of functional group peak area of water-immersed coking coal after adding inhibitors

	Coal	Coal+MgCl2	Coal+TEMPO	Coal+TPPI	Coal+PA
B	45.9323	53.47828	53.63326	52.10853	51.36536
C	20.12751	14.10579	17.95182	10.71447	17.25204
D	33.94019	32.41593	28.41491	37.177	38.21261
E	25.4423	23.35391	24.46357	24.03292	24.97235
F	13.28862	11.04319	15.60602	13.91941	15.63289
G	12.50764	6.48843	10.57709	15.03122	13.86373
H	7.24681	6.61037	5.67384	6.98002	7.6293
I	10.71042	10.83322	10.2658	11.24753	11.28428
J	7.02129	7.70701	6.44949	6.61358	8.30122
K	15.26791	24.54419	14.85628	13.20132	13.30175
L	8.41501	9.41967	11.0479	8.474	10.21748
M	24.18659	24.76253	23.43831	26.39028	23.46869
N	11.2995	10.91695	10.79539	8.89952	13.90645
O	16.17195	16.26295	13.91247	16.99651	14.59293
P	34.12535	35.75215	39.52926	36.537	28.20418
Q	14.21661	12.30542	13.32456	11.17668	18.82775
R	1.35728	4.03479	1.83249	2.80927	3.93127
S	4.09591	5.69274	3.76054	4.94256	4.34641
T	12.87505	13.43831	12.50764	12.8225	12.15927
U	40.98895	47.48203	39.48093	37.86025	36.6384
V	41.68281	29.35213	42.66677	40.78543	42.57628

Fitting results of IR spectra of water-immersed brown coal with inhibitor

700-900	X Observed	Y Generated Coal	X Observed	Y Generated Coal+MgCl2	X Observed	Y Generated Coal+TEMPO
	715.50567	2.76241E-05	715.50567	1.77774E-06	715.50567	0.00003571
	717.43426	4.03313E-05	717.43426	3.05289E-06	717.43426	4.93753E-05
	719.36284	0.000057749	719.36284	5.10115E-06	719.36284	6.71234E-05
	721.29143	0.000081095	721.29143	8.2935E-06	721.29143	8.97192E-05
	723.22002	0.000111684	723.22002	1.31196E-05	723.22002	0.000117909
	725.1486	0.000150848	725.1486	2.01938E-05	725.1486	0.000152355
	727.07719	0.000199819	727.07719	3.02433E-05	727.07719	0.000193566
	729.00578	0.000259588	729.00578	4.40711E-05	729.00578	0.000241807
	730.93436	0.000330744	730.93436	6.24875E-05	730.93436	0.000297028
	732.86295	0.000413301	732.86295	8.62085E-05	732.86295	0.000358795
	734.79154	0.000506553	734.79154	0.000115725	734.79154	0.000426251
	736.72012	0.000608975	736.72012	0.00015116	736.72012	0.000498126
	738.64871	0.000718201	738.64871	0.000192131	738.64871	0.000572793
	740.5773	0.000831115	740.5773	0.000237657	740.5773	0.000648402
	742.50588	0.000944076	742.50588	0.00028614	742.50588	0.000723081
	744.43447	0.00105	744.43447	0.000335461	744.43447	0.000795208
	746.36306	0.00116	746.36306	0.000383217	746.36306	0.000863734
	748.29164	0.00125	748.29164	0.000427116	748.29164	0.000928518
	750.22023	0.00133	750.22023	0.000465522	750.22023	0.000990622
	752.14882	0.0014	752.14882	0.000498105	752.14882	0.00105
	754.0774	0.00147	754.0774	0.000526507	754.0774	0.00112
	756.00599	0.00154	756.00599	0.00055487	756.00599	0.00119
	757.93458	0.00162	757.93458	0.000590015	757.93458	0.00128
	759.86316	0.00172	759.86316	0.000641026	759.86316	0.00139
	761.79175	0.00185	761.79175	0.000718023	761.79175	0.00152
	763.72034	0.00201	763.72034	0.0008301	763.72034	0.00167
	765.64892	0.0022	765.64892	0.000982638	765.64892	0.00185
	767.57751	0.00241	767.57751	0.00117	767.57751	0.00204
	769.5061	0.00264	769.5061	0.0014	769.5061	0.00224
	771.43468	0.00287	771.43468	0.00163	771.43468	0.00243
	773.36327	0.00308	773.36327	0.00186	773.36327	0.00261
	775.29186	0.00326	775.29186	0.00205	775.29186	0.00276
	777.22044	0.0034	777.22044	0.00219	777.22044	0.00288
	779.14903	0.0035	779.14903	0.00226	779.14903	0.00297
	781.07762	0.00357	781.07762	0.00229	781.07762	0.00305
	783.0062	0.00363	783.0062	0.00229	783.0062	0.00312
	784.93479	0.0037	784.93479	0.00229	784.93479	0.00319
	786.86338	0.00378	786.86338	0.00232	786.86338	0.00329
	788.79196	0.00389	788.79196	0.0024	788.79196	0.0034
	790.72055	0.00401	790.72055	0.00253	790.72055	0.00353
	792.64914	0.00413	792.64914	0.00269	792.64914	0.00365
	794.57772	0.00422	794.57772	0.00284	794.57772	0.00373
	796.50631	0.00425	796.50631	0.00294	796.50631	0.00376
	798.4349	0.00421	798.4349	0.00296	798.4349	0.0037
	800.36348	0.00409	800.36348	0.00287	800.36348	0.00356
	802.29207	0.00389	802.29207	0.00269	802.29207	0.00335
	804.22066	0.00363	804.22066	0.00244	804.22066	0.00309
	806.14924	0.00334	806.14924	0.00216	806.14924	0.00281
	808.07783	0.00304	808.07783	0.00188	808.07783	0.00254
	810.00642	0.00276	810.00642	0.00164	810.00642	0.00229

811.93501	0.00251	811.93501	0.00145	811.93501	0.00208
813.86359	0.0023	813.86359	0.0013	813.86359	0.00191
815.79218	0.00212	815.79218	0.00118	815.79218	0.00177
817.72077	0.00197	817.72077	0.00108	817.72077	0.00163
819.64935	0.00183	819.64935	0.000968555	819.64935	0.0015
821.57794	0.00168	821.57794	0.000849459	821.57794	0.00137
823.50653	0.00153	823.50653	0.000720754	823.50653	0.00122
825.43511	0.00138	825.43511	0.000589915	825.43511	0.00108
827.3637	0.00124	827.3637	0.000467205	827.3637	0.000952209
829.29229	0.00111	829.29229	0.000362036	829.29229	0.000838096
831.22087	0.000997003	831.22087	0.000280464	831.22087	0.000746643
833.14946	0.000915008	833.14946	0.000224266	833.14946	0.000679767
835.07805	0.000858797	835.07805	0.000191373	835.07805	0.00063592
837.00663	0.000824467	837.00663	0.000177135	837.00663	0.000611072
838.93522	0.000805973	838.93522	0.000175829	838.93522	0.000600101
840.86381	0.000796856	840.86381	0.000182018	840.86381	0.000598155
842.79239	0.000791799	842.79239	0.000191595	842.79239	0.000601697
844.72098	0.000787742	844.72098	0.00020247	844.72098	0.000609103
846.64957	0.000784439	846.64957	0.000214932	846.64957	0.000620778
848.57815	0.000784479	848.57815	0.000231677	848.57815	0.000638861
850.50674	0.000792755	850.50674	0.000257428	850.50674	0.000666573
852.43533	0.000815437	852.43533	0.00029807	852.43533	0.000707323
854.36391	0.000858489	854.36391	0.000359285	854.36391	0.000763687
856.2925	0.000925886	856.2925	0.000444793	856.2925	0.000836394
858.22109	0.00102	858.22109	0.000554536	858.22109	0.000923541
860.14967	0.00113	860.14967	0.000683311	860.14967	0.00102
862.07826	0.00125	862.07826	0.000820396	862.07826	0.00112
864.00685	0.00137	864.00685	0.000950591	864.00685	0.00121
865.93543	0.00146	865.93543	0.00106	865.93543	0.00128
867.86402	0.00151	867.86402	0.00112	867.86402	0.00132
869.79261	0.00152	869.79261	0.00114	869.79261	0.00133
871.72119	0.00146	871.72119	0.0011	871.72119	0.0013
873.64978	0.00135	873.64978	0.00101	873.64978	0.00123
875.57837	0.0012	875.57837	0.000884768	875.57837	0.00112
877.50695	0.00103	877.50695	0.000736758	877.50695	0.0009937
879.43554	0.000835829	879.43554	0.000583655	879.43554	0.000850154
881.36413	0.000652017	881.36413	0.00043983	881.36413	0.000702809
883.29271	0.000486561	883.29271	0.000315275	883.29271	0.00056125
885.2213	0.000347259	885.2213	0.000214959	885.2213	0.000432882
887.14989	0.000236999	887.14989	0.000139404	887.14989	0.000322416
889.07847	0.00015466	889.07847	8.59886E-05	889.07847	0.000231875
891.00706	9.64996E-05	891.00706	5.04489E-05	891.00706	0.000161008
892.93565	5.75695E-05	892.93565	2.81517E-05	892.93565	0.000107938
894.86423	3.28393E-05	894.86423	1.49417E-05	894.86423	6.98589E-05
896.79282	0.000017913	896.79282	7.54281E-06	896.79282	4.36487E-05
898.72141	9.34485E-06	898.72141	3.62166E-06	898.72141	2.63278E-05

X Observed	Y Generated Coal+TPPI	X Observed	Y Generated Coal+PA
715.50567	3.4968E-05	715.50567	2.8631E-05
717.43426	5.5561E-05	717.43426	4.0783E-05
719.36284	8.6115E-05	719.36284	5.7061E-05
721.29143	0.0001302	721.29143	7.8422E-05
723.22002	0.00019201	723.22002	0.00010587
725.1486	0.00027622	725.1486	0.00014038
727.07719	0.00038762	727.07719	0.00018285
729.00578	0.00053059	729.00578	0.00023394
730.93436	0.00070848	730.93436	0.00029402
732.86295	0.0009228	732.86295	0.00036298
734.79154	0.00117	734.79154	0.00044022
736.72012	0.00145	736.72012	0.00052452
738.64871	0.00176	738.64871	0.00061409
740.5773	0.00207	740.5773	0.00070658
742.50588	0.00238	742.50588	0.0007993
744.43447	0.00267	744.43447	0.00088944
746.36306	0.00293	746.36306	0.00097443
748.29164	0.00313	748.29164	0.00105
750.22023	0.00326	750.22023	0.00112
752.14882	0.00333	752.14882	0.00118
754.0774	0.00331	754.0774	0.00124
756.00599	0.00324	756.00599	0.0013
757.93458	0.00311	757.93458	0.00135
759.86316	0.00296	759.86316	0.00142
761.79175	0.00281	761.79175	0.0015
763.72034	0.00269	763.72034	0.0016
765.64892	0.00262	765.64892	0.00172
767.57751	0.00261	767.57751	0.00185
769.5061	0.00267	769.5061	0.00199
771.43468	0.00278	771.43468	0.00214
773.36327	0.00292	773.36327	0.00228
775.29186	0.00306	775.29186	0.00241
777.22044	0.00316	777.22044	0.00252
779.14903	0.00321	779.14903	0.00262
781.07762	0.00322	781.07762	0.00271
783.0062	0.00319	783.0062	0.00278
784.93479	0.00314	784.93479	0.00285
786.86338	0.00311	786.86338	0.00291
788.79196	0.0031	788.79196	0.00297
790.72055	0.00312	790.72055	0.00301
792.64914	0.00316	792.64914	0.00303
794.57772	0.00321	794.57772	0.00299
796.50631	0.00324	796.50631	0.00291
798.4349	0.00324	798.4349	0.00277
800.36348	0.00318	800.36348	0.00259
802.29207	0.00308	802.29207	0.00238
804.22066	0.00294	804.22066	0.00216
806.14924	0.00278	806.14924	0.00197
808.07783	0.0026	808.07783	0.0018
810.00642	0.00242	810.00642	0.00168

811.93501	0.00224	811.93501	0.00159
813.86359	0.00206	813.86359	0.00153
815.79218	0.00188	815.79218	0.00148
817.72077	0.00171	817.72077	0.00142
819.64935	0.00153	819.64935	0.00135
821.57794	0.00135	821.57794	0.00126
823.50653	0.00118	823.50653	0.00115
825.43511	0.00103	825.43511	0.00103
827.3637	0.00088995	827.3637	0.00091568
829.29229	0.00077532	829.29229	0.0008133
831.22087	0.00068619	831.22087	0.00073076
833.14946	0.0006221	833.14946	0.00067104
835.07805	0.0005802	835.07805	0.0006331
837.00663	0.00055603	837.00663	0.00061292
838.93522	0.0005444	838.93522	0.00060501
840.86381	0.00054029	840.86381	0.00060393
842.79239	0.00053968	842.79239	0.00060539
844.72098	0.0005401	844.72098	0.00060704
846.64957	0.00054107	846.64957	0.00060865
848.57815	0.00054421	848.57815	0.00061201
850.50674	0.00055306	850.50674	0.00062041
852.43533	0.00057255	852.43533	0.00063784
854.36391	0.00060799	854.36391	0.00066806
856.2925	0.00066366	856.2925	0.00071347
858.22109	0.00074122	858.22109	0.00077426
860.14967	0.00083829	860.14967	0.00084762
862.07826	0.00094761	862.07826	0.00092772
864.00685	0.00106	864.00685	0.00101
865.93543	0.00115	865.93543	0.00107
867.86402	0.00122	867.86402	0.00112
869.79261	0.00124	869.79261	0.00113
871.72119	0.00121	871.72119	0.00111
873.64978	0.00114	873.64978	0.00106
875.57837	0.00102	875.57837	0.00097254
877.50695	0.0008761	877.50695	0.00086208
879.43554	0.00071738	879.43554	0.00073686
881.36413	0.00056068	881.36413	0.000607
883.29271	0.00041813	883.29271	0.00048175
885.2213	0.00029747	885.2213	0.00036826
887.14989	0.00020186	887.14989	0.0002711
889.07847	0.00013065	889.07847	0.00019217
891.00706	8.065E-05	891.00706	0.00013115
892.93565	4.7484E-05	892.93565	8.6174E-05
894.86423	2.6667E-05	894.86423	5.451E-05
896.79282	1.4288E-05	896.79282	3.3193E-05
898.72141	7.3044E-06	898.72141	1.9457E-05

Fitting results of IR spectra of water-immersed brown coal with inhibitor

**1000-1800**	X Observed	Y Generated Coal	X Observed	Y Generated Coal+MgCl2	X Observed	Y Generated Coal+TEMPO
	1000.9365	0.00994	1000.9365	0.01274	1000.9365	0.00726
	1002.86509	0.01066	1002.86509	0.0135	1002.86509	0.00781
	1004.79367	0.01139	1004.79367	0.01425	1004.79367	0.00836
	1006.72226	0.01211	1006.72226	0.01501	1006.72226	0.00891
	1008.65085	0.01283	1008.65085	0.01575	1008.65085	0.00947
	1010.57943	0.01355	1010.57943	0.01647	1010.57943	0.01003
	1012.50802	0.01424	1012.50802	0.01717	1012.50802	0.01057
	1014.43661	0.01492	1014.43661	0.01785	1014.43661	0.01111
	1016.36519	0.01557	1016.36519	0.0185	1016.36519	0.01163
	1018.29378	0.01619	1018.29378	0.01911	1018.29378	0.01213
	1020.22237	0.01677	1020.22237	0.01969	1020.22237	0.01261
	1022.15095	0.01732	1022.15095	0.02022	1022.15095	0.01306
	1024.07954	0.01782	1024.07954	0.02071	1024.07954	0.01349
	1026.00813	0.01828	1026.00813	0.02116	1026.00813	0.01388
	1027.93671	0.01869	1027.93671	0.02155	1027.93671	0.01424
	1029.8653	0.01905	1029.8653	0.0219	1029.8653	0.01457
	1031.79389	0.01937	1031.79389	0.02221	1031.79389	0.01487
	1033.72247	0.01963	1033.72247	0.02246	1033.72247	0.01512
	1035.65106	0.01985	1035.65106	0.02267	1035.65106	0.01535
	1037.57965	0.02003	1037.57965	0.02283	1037.57965	0.01554
	1039.50823	0.02016	1039.50823	0.02296	1039.50823	0.0157
	1041.43682	0.02026	1041.43682	0.02304	1041.43682	0.01583
	1043.36541	0.02033	1043.36541	0.02309	1043.36541	0.01594
	1045.29399	0.02037	1045.29399	0.02312	1045.29399	0.01602
	1047.22258	0.02039	1047.22258	0.02311	1047.22258	0.01609
	1049.15117	0.02039	1049.15117	0.02309	1049.15117	0.01614
	1051.07975	0.02038	1051.07975	0.02305	1051.07975	0.01617
	1053.00834	0.02036	1053.00834	0.023	1053.00834	0.0162
	1054.93693	0.02035	1054.93693	0.02295	1054.93693	0.01623
	1056.86551	0.02034	1056.86551	0.02289	1056.86551	0.01626
	1058.7941	0.02034	1058.7941	0.02284	1058.7941	0.01629
	1060.72269	0.02036	1060.72269	0.02279	1060.72269	0.01633
	1062.65127	0.02039	1062.65127	0.02275	1062.65127	0.01638
	1064.57986	0.02044	1064.57986	0.02273	1064.57986	0.01644
	1066.50845	0.02052	1066.50845	0.02273	1066.50845	0.01652
	1068.43703	0.02062	1068.43703	0.02274	1068.43703	0.01661
	1070.36562	0.02075	1070.36562	0.02276	1070.36562	0.01671
	1072.29421	0.02089	1072.29421	0.02281	1072.29421	0.01684
	1074.22279	0.02106	1074.22279	0.02287	1074.22279	0.01697
	1076.15138	0.02125	1076.15138	0.02295	1076.15138	0.01712
	1078.07997	0.02146	1078.07997	0.02305	1078.07997	0.01729
	1080.00855	0.02168	1080.00855	0.02315	1080.00855	0.01746
	1081.93714	0.02191	1081.93714	0.02326	1081.93714	0.01764
	1083.86573	0.02214	1083.86573	0.02338	1083.86573	0.01782
	1085.79431	0.02237	1085.79431	0.0235	1085.79431	0.018
	1087.7229	0.0226	1087.7229	0.02361	1087.7229	0.01818
	1089.65149	0.02282	1089.65149	0.02372	1089.65149	0.01835
	1091.58007	0.02303	1091.58007	0.02382	1091.58007	0.01851
	1093.50866	0.02322	1093.50866	0.0239	1093.50866	0.01865
	1095.43725	0.02338	1095.43725	0.02397	1095.43725	0.01878

1097.36584	0.02352	1097.36584	0.02401	1097.36584	0.01889
1099.29442	0.02363	1099.29442	0.02403	1099.29442	0.01898
1101.22301	0.02371	1101.22301	0.02402	1101.22301	0.01904
1103.1516	0.02375	1103.1516	0.02398	1103.1516	0.01907
1105.08018	0.02375	1105.08018	0.0239	1105.08018	0.01908
1107.00877	0.02372	1107.00877	0.0238	1107.00877	0.01906
1108.93736	0.02366	1108.93736	0.02367	1108.93736	0.01901
1110.86594	0.02356	1110.86594	0.0235	1110.86594	0.01893
1112.79453	0.02342	1112.79453	0.02331	1112.79453	0.01883
1114.72312	0.02325	1114.72312	0.02308	1114.72312	0.01871
1116.6517	0.02306	1116.6517	0.02283	1116.6517	0.01856
1118.58029	0.02284	1118.58029	0.02256	1118.58029	0.01838
1120.50888	0.02259	1120.50888	0.02227	1120.50888	0.0182
1122.43746	0.02233	1122.43746	0.02196	1122.43746	0.01799
1124.36605	0.02205	1124.36605	0.02164	1124.36605	0.01778
1126.29464	0.02177	1126.29464	0.0213	1126.29464	0.01756
1128.22322	0.02147	1128.22322	0.02097	1128.22322	0.01733
1130.15181	0.02118	1130.15181	0.02063	1130.15181	0.0171
1132.0804	0.02089	1132.0804	0.0203	1132.0804	0.01687
1134.00898	0.0206	1134.00898	0.01997	1134.00898	0.01664
1135.93757	0.02032	1135.93757	0.01964	1135.93757	0.01642
1137.86616	0.02005	1137.86616	0.01933	1137.86616	0.0162
1139.79474	0.0198	1139.79474	0.01903	1139.79474	0.016
1141.72333	0.01956	1141.72333	0.01875	1141.72333	0.0158
1143.65192	0.01934	1143.65192	0.01847	1143.65192	0.01562
1145.5805	0.01913	1145.5805	0.01822	1145.5805	0.01545
1147.50909	0.01894	1147.50909	0.01798	1147.50909	0.0153
1149.43768	0.01877	1149.43768	0.01775	1149.43768	0.01515
1151.36626	0.01862	1151.36626	0.01754	1151.36626	0.01502
1153.29485	0.01847	1153.29485	0.01734	1153.29485	0.0149
1155.22344	0.01834	1155.22344	0.01715	1155.22344	0.01478
1157.15202	0.01822	1157.15202	0.01697	1157.15202	0.01468
1159.08061	0.01811	1159.08061	0.0168	1159.08061	0.01458
1161.0092	0.018	1161.0092	0.01662	1161.0092	0.01448
1162.93778	0.0179	1162.93778	0.01645	1162.93778	0.01439
1164.86637	0.01779	1164.86637	0.01628	1164.86637	0.01429
1166.79496	0.01769	1166.79496	0.0161	1166.79496	0.0142
1168.72354	0.01757	1168.72354	0.01592	1168.72354	0.0141
1170.65213	0.01745	1170.65213	0.01572	1170.65213	0.01399
1172.58072	0.01733	1172.58072	0.01552	1172.58072	0.01388
1174.5093	0.01718	1174.5093	0.0153	1174.5093	0.01376
1176.43789	0.01703	1176.43789	0.01507	1176.43789	0.01363
1178.36648	0.01687	1178.36648	0.01483	1178.36648	0.0135
1180.29506	0.01669	1180.29506	0.01458	1180.29506	0.01335
1182.22365	0.01649	1182.22365	0.01431	1182.22365	0.01319
1184.15224	0.01629	1184.15224	0.01403	1184.15224	0.01303
1186.08082	0.01607	1186.08082	0.01373	1186.08082	0.01285
1188.00941	0.01584	1188.00941	0.01343	1188.00941	0.01267
1189.938	0.0156	1189.938	0.01311	1189.938	0.01248
1191.86658	0.01536	1191.86658	0.01279	1191.86658	0.01228
1193.79517	0.0151	1193.79517	0.01247	1193.79517	0.01208
1195.72376	0.01485	1195.72376	0.01214	1195.72376	0.01188
1197.65234	0.01459	1197.65234	0.01182	1197.65234	0.01168
1199.58093	0.01433	1199.58093	0.01149	1199.58093	0.01148
1201.50952	0.01408	1201.50952	0.01118	1201.50952	0.01128
1203.4381	0.01383	1203.4381	0.01087	1203.4381	0.01108

1205.36669	0.01359	1205.36669	0.01057	1205.36669	0.01089
1207.29528	0.01335	1207.29528	0.01028	1207.29528	0.0107
1209.22386	0.01313	1209.22386	0.01	1209.22386	0.01053
1211.15245	0.01292	1211.15245	0.00974	1211.15245	0.01036
1213.08104	0.01273	1213.08104	0.0095	1213.08104	0.0102
1215.00962	0.01254	1215.00962	0.00927	1215.00962	0.01005
1216.93821	0.01238	1216.93821	0.00905	1216.93821	0.0099
1218.8668	0.01222	1218.8668	0.00886	1218.8668	0.00977
1220.79538	0.01208	1220.79538	0.00868	1220.79538	0.00965
1222.72397	0.01195	1222.72397	0.00852	1222.72397	0.00953
1224.65256	0.01183	1224.65256	0.00837	1224.65256	0.00942
1226.58114	0.01173	1226.58114	0.00824	1226.58114	0.00932
1228.50973	0.01163	1228.50973	0.00812	1228.50973	0.00922
1230.43832	0.01154	1230.43832	0.00801	1230.43832	0.00913
1232.3669	0.01146	1232.3669	0.00791	1232.3669	0.00904
1234.29549	0.01138	1234.29549	0.00782	1234.29549	0.00895
1236.22408	0.0113	1236.22408	0.00773	1236.22408	0.00887
1238.15266	0.01122	1238.15266	0.00765	1238.15266	0.00878
1240.08125	0.01115	1240.08125	0.00756	1240.08125	0.00869
1242.00984	0.01107	1242.00984	0.00748	1242.00984	0.0086
1243.93842	0.01098	1243.93842	0.0074	1243.93842	0.0085
1245.86701	0.0109	1245.86701	0.00732	1245.86701	0.0084
1247.7956	0.0108	1247.7956	0.00724	1247.7956	0.0083
1249.72418	0.0107	1249.72418	0.00715	1249.72418	0.00819
1251.65277	0.0106	1251.65277	0.00706	1251.65277	0.00808
1253.58136	0.01049	1253.58136	0.00696	1253.58136	0.00796
1255.50994	0.01037	1255.50994	0.00686	1255.50994	0.00784
1257.43853	0.01024	1257.43853	0.00676	1257.43853	0.00771
1259.36712	0.01011	1259.36712	0.00665	1259.36712	0.00758
1261.2957	0.00998	1261.2957	0.00655	1261.2957	0.00745
1263.22429	0.00984	1263.22429	0.00643	1263.22429	0.00732
1265.15288	0.0097	1265.15288	0.00632	1265.15288	0.00719
1267.08146	0.00955	1267.08146	0.00621	1267.08146	0.00706
1269.01005	0.00941	1269.01005	0.0061	1269.01005	0.00693
1270.93864	0.00926	1270.93864	0.00599	1270.93864	0.0068
1272.86722	0.00912	1272.86722	0.00588	1272.86722	0.00668
1274.79581	0.00898	1274.79581	0.00578	1274.79581	0.00656
1276.7244	0.00884	1276.7244	0.00568	1276.7244	0.00644
1278.65298	0.0087	1278.65298	0.00558	1278.65298	0.00632
1280.58157	0.00857	1280.58157	0.00549	1280.58157	0.00621
1282.51016	0.00844	1282.51016	0.0054	1282.51016	0.00611
1284.43874	0.00832	1284.43874	0.00532	1284.43874	0.00601
1286.36733	0.00819	1286.36733	0.00524	1286.36733	0.00591
1288.29592	0.00807	1288.29592	0.00517	1288.29592	0.00581
1290.22451	0.00796	1290.22451	0.00509	1290.22451	0.00572
1292.15309	0.00784	1292.15309	0.00503	1292.15309	0.00563
1294.08168	0.00773	1294.08168	0.00496	1294.08168	0.00554
1296.01027	0.00761	1296.01027	0.00489	1296.01027	0.00545
1297.93885	0.0075	1297.93885	0.00483	1297.93885	0.00537
1299.86744	0.00738	1299.86744	0.00477	1299.86744	0.00528
1301.79603	0.00726	1301.79603	0.0047	1301.79603	0.00519
1303.72461	0.00714	1303.72461	0.00464	1303.72461	0.0051
1305.6532	0.00702	1305.6532	0.00457	1305.6532	0.005
1307.58179	0.00689	1307.58179	0.0045	1307.58179	0.00491
1309.51037	0.00676	1309.51037	0.00443	1309.51037	0.00481
1311.43896	0.00663	1311.43896	0.00436	1311.43896	0.00472

1313.36755	0.0065	1313.36755	0.00429	1313.36755	0.00462
1315.29613	0.00637	1315.29613	0.00422	1315.29613	0.00452
1317.22472	0.00624	1317.22472	0.00415	1317.22472	0.00443
1319.15331	0.00612	1319.15331	0.00409	1319.15331	0.00434
1321.08189	0.00599	1321.08189	0.00402	1321.08189	0.00425
1323.01048	0.00588	1323.01048	0.00396	1323.01048	0.00417
1324.93907	0.00577	1324.93907	0.00391	1324.93907	0.0041
1326.86765	0.00567	1326.86765	0.00386	1326.86765	0.00403
1328.79624	0.00558	1328.79624	0.00382	1328.79624	0.00398
1330.72483	0.00551	1330.72483	0.00378	1330.72483	0.00394
1332.65341	0.00545	1332.65341	0.00376	1332.65341	0.00391
1334.582	0.00542	1334.582	0.00375	1334.582	0.00389
1336.51059	0.0054	1336.51059	0.00376	1336.51059	0.00389
1338.43917	0.0054	1338.43917	0.00377	1338.43917	0.00391
1340.36776	0.00542	1340.36776	0.0038	1340.36776	0.00394
1342.29635	0.00546	1342.29635	0.00385	1342.29635	0.00399
1344.22493	0.00552	1344.22493	0.00391	1344.22493	0.00406
1346.15352	0.0056	1346.15352	0.00398	1346.15352	0.00414
1348.08211	0.00571	1348.08211	0.00406	1348.08211	0.00424
1350.01069	0.00583	1350.01069	0.00416	1350.01069	0.00436
1351.93928	0.00597	1351.93928	0.00427	1351.93928	0.00448
1353.86787	0.00613	1353.86787	0.00439	1353.86787	0.00462
1355.79645	0.00629	1355.79645	0.00452	1355.79645	0.00477
1357.72504	0.00647	1357.72504	0.00466	1357.72504	0.00492
1359.65363	0.00666	1359.65363	0.0048	1359.65363	0.00508
1361.58221	0.00685	1361.58221	0.00495	1361.58221	0.00524
1363.5108	0.00704	1363.5108	0.0051	1363.5108	0.00541
1365.43939	0.00723	1365.43939	0.00525	1365.43939	0.00557
1367.36797	0.00742	1367.36797	0.0054	1367.36797	0.00572
1369.29656	0.00761	1369.29656	0.00554	1369.29656	0.00587
1371.22515	0.00778	1371.22515	0.00569	1371.22515	0.00601
1373.15373	0.00794	1373.15373	0.00583	1373.15373	0.00614
1375.08232	0.00809	1375.08232	0.00596	1375.08232	0.00626
1377.01091	0.00823	1377.01091	0.00609	1377.01091	0.00636
1378.93949	0.00835	1378.93949	0.0062	1378.93949	0.00645
1380.86808	0.00845	1380.86808	0.00631	1380.86808	0.00653
1382.79667	0.00854	1382.79667	0.00641	1382.79667	0.00659
1384.72525	0.00861	1384.72525	0.00651	1384.72525	0.00663
1386.65384	0.00867	1386.65384	0.00659	1386.65384	0.00667
1388.58243	0.00871	1388.58243	0.00667	1388.58243	0.00669
1390.51101	0.00874	1390.51101	0.00674	1390.51101	0.0067
1392.4396	0.00876	1392.4396	0.0068	1392.4396	0.0067
1394.36819	0.00877	1394.36819	0.00685	1394.36819	0.00669
1396.29677	0.00877	1396.29677	0.00691	1396.29677	0.00667
1398.22536	0.00876	1398.22536	0.00695	1398.22536	0.00665
1400.15395	0.00875	1400.15395	0.007	1400.15395	0.00663
1402.08253	0.00874	1402.08253	0.00704	1402.08253	0.00661
1404.01112	0.00873	1404.01112	0.00708	1404.01112	0.00659
1405.93971	0.00872	1405.93971	0.00712	1405.93971	0.00657
1407.86829	0.00871	1407.86829	0.00716	1407.86829	0.00655
1409.79688	0.0087	1409.79688	0.0072	1409.79688	0.00654
1411.72547	0.0087	1411.72547	0.00724	1411.72547	0.00654
1413.65405	0.00871	1413.65405	0.00728	1413.65405	0.00654
1415.58264	0.00872	1415.58264	0.00732	1415.58264	0.00655
1417.51123	0.00873	1417.51123	0.00737	1417.51123	0.00657
1419.43981	0.00876	1419.43981	0.00741	1419.43981	0.00659

1421.3684	0.00878	1421.3684	0.00745	1421.3684	0.00662
1423.29699	0.00881	1423.29699	0.00748	1423.29699	0.00665
1425.22557	0.00884	1425.22557	0.00751	1425.22557	0.00669
1427.15416	0.00887	1427.15416	0.00754	1427.15416	0.00673
1429.08275	0.0089	1429.08275	0.00756	1429.08275	0.00676
1431.01133	0.00893	1431.01133	0.00758	1431.01133	0.0068
1432.93992	0.00896	1432.93992	0.00758	1432.93992	0.00683
1434.86851	0.00897	1434.86851	0.00757	1434.86851	0.00686
1436.79709	0.00899	1436.79709	0.00755	1436.79709	0.00689
1438.72568	0.00899	1438.72568	0.00752	1438.72568	0.0069
1440.65427	0.00898	1440.65427	0.00747	1440.65427	0.00691
1442.58285	0.00895	1442.58285	0.00741	1442.58285	0.0069
1444.51144	0.00892	1444.51144	0.00733	1444.51144	0.00688
1446.44003	0.00886	1446.44003	0.00724	1446.44003	0.00685
1448.36861	0.0088	1448.36861	0.00713	1448.36861	0.00681
1450.2972	0.00871	1450.2972	0.00701	1450.2972	0.00675
1452.22579	0.00861	1452.22579	0.00687	1452.22579	0.00668
1454.15437	0.0085	1454.15437	0.00671	1454.15437	0.00659
1456.08296	0.00836	1456.08296	0.00655	1456.08296	0.00649
1458.01155	0.00821	1458.01155	0.00637	1458.01155	0.00637
1459.94013	0.00805	1459.94013	0.00618	1459.94013	0.00625
1461.86872	0.00787	1461.86872	0.00598	1461.86872	0.00611
1463.79731	0.00768	1463.79731	0.00577	1463.79731	0.00597
1465.72589	0.00748	1465.72589	0.00556	1465.72589	0.00581
1467.65448	0.00726	1467.65448	0.00535	1467.65448	0.00565
1469.58307	0.00705	1469.58307	0.00513	1469.58307	0.00549
1471.51165	0.00682	1471.51165	0.00491	1471.51165	0.00532
1473.44024	0.00659	1473.44024	0.0047	1473.44024	0.00515
1475.36883	0.00637	1475.36883	0.00449	1475.36883	0.00498
1477.29741	0.00614	1477.29741	0.00429	1477.29741	0.00481
1479.226	0.00592	1479.226	0.00409	1479.226	0.00464
1481.15459	0.00571	1481.15459	0.0039	1481.15459	0.00448
1483.08318	0.00551	1483.08318	0.00372	1483.08318	0.00433
1485.01176	0.00532	1485.01176	0.00355	1485.01176	0.00418
1486.94035	0.00515	1486.94035	0.00339	1486.94035	0.00404
1488.86894	0.00499	1488.86894	0.00325	1488.86894	0.00391
1490.79752	0.00485	1490.79752	0.00312	1490.79752	0.00379
1492.72611	0.00473	1492.72611	0.003	1492.72611	0.00368
1494.6547	0.00463	1494.6547	0.00289	1494.6547	0.00358
1496.58328	0.00456	1496.58328	0.0028	1496.58328	0.00349
1498.51187	0.00451	1498.51187	0.00272	1498.51187	0.00341
1500.44046	0.00448	1500.44046	0.00265	1500.44046	0.00334
1502.36904	0.00448	1502.36904	0.0026	1502.36904	0.00329
1504.29763	0.0045	1504.29763	0.00256	1504.29763	0.00324
1506.22622	0.00454	1506.22622	0.00253	1506.22622	0.0032
1508.1548	0.00461	1508.1548	0.00252	1508.1548	0.00318
1510.08339	0.00469	1510.08339	0.00251	1510.08339	0.00316
1512.01198	0.00479	1512.01198	0.00252	1512.01198	0.00315
1513.94056	0.00491	1513.94056	0.00255	1513.94056	0.00315
1515.86915	0.00504	1515.86915	0.00258	1515.86915	0.00316
1517.79774	0.00519	1517.79774	0.00263	1517.79774	0.00317
1519.72632	0.00535	1519.72632	0.00269	1519.72632	0.0032
1521.65491	0.00553	1521.65491	0.00276	1521.65491	0.00323
1523.5835	0.00571	1523.5835	0.00285	1523.5835	0.00328
1525.51208	0.00591	1525.51208	0.00295	1525.51208	0.00333
1527.44067	0.00612	1527.44067	0.00307	1527.44067	0.00339

1529.36926	0.00634	1529.36926	0.00321	1529.36926	0.00347
1531.29784	0.00657	1531.29784	0.00337	1531.29784	0.00356
1533.22643	0.00682	1533.22643	0.00355	1533.22643	0.00367
1535.15502	0.00709	1535.15502	0.00375	1535.15502	0.00379
1537.0836	0.00738	1537.0836	0.00398	1537.0836	0.00393
1539.01219	0.00769	1539.01219	0.00424	1539.01219	0.0041
1540.94078	0.00802	1540.94078	0.00453	1540.94078	0.00429
1542.86936	0.00839	1542.86936	0.00485	1542.86936	0.0045
1544.79795	0.00878	1544.79795	0.0052	1544.79795	0.00475
1546.72654	0.00921	1546.72654	0.00559	1546.72654	0.00503
1548.65512	0.00967	1548.65512	0.00602	1548.65512	0.00534
1550.58371	0.01017	1550.58371	0.00649	1550.58371	0.00569
1552.5123	0.01071	1552.5123	0.007	1552.5123	0.00608
1554.44088	0.01129	1554.44088	0.00756	1554.44088	0.00651
1556.36947	0.01191	1556.36947	0.00816	1556.36947	0.00699
1558.29806	0.01257	1558.29806	0.00881	1558.29806	0.00752
1560.22664	0.01327	1560.22664	0.0095	1560.22664	0.0081
1562.15523	0.01401	1562.15523	0.01025	1562.15523	0.00872
1564.08382	0.01479	1564.08382	0.01104	1564.08382	0.0094
1566.0124	0.01561	1566.0124	0.01188	1566.0124	0.01012
1567.94099	0.01646	1567.94099	0.01277	1567.94099	0.0109
1569.86958	0.01733	1569.86958	0.0137	1569.86958	0.01172
1571.79816	0.01823	1571.79816	0.01467	1571.79816	0.01259
1573.72675	0.01916	1573.72675	0.01568	1573.72675	0.0135
1575.65534	0.0201	1575.65534	0.01673	1575.65534	0.01445
1577.58392	0.02105	1577.58392	0.01782	1577.58392	0.01544
1579.51251	0.02201	1579.51251	0.01893	1579.51251	0.01646
1581.4411	0.02297	1581.4411	0.02006	1581.4411	0.0175
1583.36968	0.02394	1583.36968	0.02122	1583.36968	0.01856
1585.29827	0.02489	1585.29827	0.02238	1585.29827	0.01964
1587.22686	0.02583	1587.22686	0.02356	1587.22686	0.02072
1589.15544	0.02676	1589.15544	0.02473	1589.15544	0.0218
1591.08403	0.02766	1591.08403	0.0259	1591.08403	0.02287
1593.01262	0.02854	1593.01262	0.02706	1593.01262	0.02392
1594.9412	0.02939	1594.9412	0.02819	1594.9412	0.02494
1596.86979	0.0302	1596.86979	0.02931	1596.86979	0.02593
1598.79838	0.03097	1598.79838	0.03039	1598.79838	0.02688
1600.72696	0.0317	1600.72696	0.03143	1600.72696	0.02778
1602.65555	0.03239	1602.65555	0.03242	1602.65555	0.02862
1604.58414	0.03302	1604.58414	0.03337	1604.58414	0.02939
1606.51272	0.0336	1606.51272	0.03426	1606.51272	0.0301
1608.44131	0.03413	1608.44131	0.03508	1608.44131	0.03073
1610.3699	0.03459	1610.3699	0.03584	1610.3699	0.03128
1612.29848	0.035	1612.29848	0.03654	1612.29848	0.03175
1614.22707	0.03535	1614.22707	0.03715	1614.22707	0.03213
1616.15566	0.03563	1616.15566	0.03769	1616.15566	0.03242
1618.08424	0.03585	1618.08424	0.03815	1618.08424	0.03263
1620.01283	0.03601	1620.01283	0.03853	1620.01283	0.03274
1621.94142	0.0361	1621.94142	0.03882	1621.94142	0.03277
1623.87	0.03613	1623.87	0.03903	1623.87	0.03271
1625.79859	0.0361	1625.79859	0.03916	1625.79859	0.03257
1627.72718	0.03599	1627.72718	0.0392	1627.72718	0.03235
1629.65576	0.03583	1629.65576	0.03916	1629.65576	0.03205
1631.58435	0.0356	1631.58435	0.03904	1631.58435	0.03168
1633.51294	0.03532	1633.51294	0.03883	1633.51294	0.03124
1635.44152	0.03497	1635.44152	0.03855	1635.44152	0.03075

1637.37011	0.03457	1637.37011	0.03819	1637.37011	0.03019
1639.2987	0.03411	1639.2987	0.03776	1639.2987	0.02959
1641.22728	0.03359	1641.22728	0.03725	1641.22728	0.02894
1643.15587	0.03303	1643.15587	0.03668	1643.15587	0.02826
1645.08446	0.03241	1645.08446	0.03605	1645.08446	0.02754
1647.01304	0.03175	1647.01304	0.03536	1647.01304	0.0268
1648.94163	0.03105	1648.94163	0.0346	1648.94163	0.02603
1650.87022	0.0303	1650.87022	0.0338	1650.87022	0.02525
1652.7988	0.02952	1652.7988	0.03295	1652.7988	0.02446
1654.72739	0.0287	1654.72739	0.03206	1654.72739	0.02365
1656.65598	0.02786	1656.65598	0.03112	1656.65598	0.02285
1658.58456	0.02698	1658.58456	0.03015	1658.58456	0.02204
1660.51315	0.02609	1660.51315	0.02915	1660.51315	0.02124
1662.44174	0.02517	1662.44174	0.02813	1662.44174	0.02044
1664.37032	0.02423	1664.37032	0.02708	1664.37032	0.01965
1666.29891	0.02328	1666.29891	0.02602	1666.29891	0.01887
1668.2275	0.02233	1668.2275	0.02494	1668.2275	0.01809
1670.15608	0.02136	1670.15608	0.02385	1670.15608	0.01734
1672.08467	0.0204	1672.08467	0.02276	1672.08467	0.01659
1674.01326	0.01944	1674.01326	0.02167	1674.01326	0.01586
1675.94185	0.01848	1675.94185	0.02059	1675.94185	0.01514
1677.87043	0.01753	1677.87043	0.01951	1677.87043	0.01445
1679.79902	0.01659	1679.79902	0.01845	1679.79902	0.01376
1681.72761	0.01566	1681.72761	0.0174	1681.72761	0.01309
1683.65619	0.01476	1683.65619	0.01637	1683.65619	0.01244
1685.58478	0.01387	1685.58478	0.01536	1685.58478	0.01181
1687.51337	0.013	1687.51337	0.01438	1687.51337	0.01119
1689.44195	0.01216	1689.44195	0.01343	1689.44195	0.0106
1691.37054	0.01135	1691.37054	0.01251	1691.37054	0.01001
1693.29913	0.01056	1693.29913	0.01162	1693.29913	0.00945
1695.22771	0.00981	1695.22771	0.01076	1695.22771	0.0089
1697.1563	0.00909	1697.1563	0.00994	1697.1563	0.00838
1699.08489	0.00839	1699.08489	0.00916	1699.08489	0.00787
1701.01347	0.00773	1701.01347	0.00842	1701.01347	0.00738
1702.94206	0.00711	1702.94206	0.00771	1702.94206	0.0069
1704.87065	0.00652	1704.87065	0.00705	1704.87065	0.00645
1706.79923	0.00596	1706.79923	0.00642	1706.79923	0.00601
1708.72782	0.00543	1708.72782	0.00584	1708.72782	0.0056
1710.65641	0.00494	1710.65641	0.00529	1710.65641	0.0052
1712.58499	0.00448	1712.58499	0.00478	1712.58499	0.00482
1714.51358	0.00405	1714.51358	0.0043	1714.51358	0.00446
1716.44217	0.00366	1716.44217	0.00386	1716.44217	0.00412
1718.37075	0.00329	1718.37075	0.00346	1718.37075	0.00379
1720.29934	0.00295	1720.29934	0.00309	1720.29934	0.00349
1722.22793	0.00264	1722.22793	0.00275	1722.22793	0.0032
1724.15651	0.00236	1724.15651	0.00244	1724.15651	0.00293
1726.0851	0.0021	1726.0851	0.00216	1726.0851	0.00267
1728.01369	0.00186	1728.01369	0.0019	1728.01369	0.00243
1729.94227	0.00164	1729.94227	0.00167	1729.94227	0.00221
1731.87086	0.00145	1731.87086	0.00147	1731.87086	0.00201
1733.79945	0.00128	1733.79945	0.00128	1733.79945	0.00182
1735.72803	0.00112	1735.72803	0.00112	1735.72803	0.00164
1737.65662	0.00097895	1737.65662	0.000969225	1737.65662	0.00148
1739.58521	0.00085373	1739.58521	0.000839069	1739.58521	0.00133
1741.51379	0.00074237	1741.51379	0.00072412	1741.51379	0.00119
1743.44238	0.00064367	1743.44238	0.000622957	1743.44238	0.00106

1745.37097	0.00055648	1745.37097	0.000534242	1745.37097	0.00094964
1747.29955	0.00047969	1747.29955	0.000456715	1747.29955	0.000845286
1749.22814	0.00041229	1749.22814	0.000389205	1749.22814	0.000750599
1751.15673	0.00035332	1751.15673	0.000330623	1751.15673	0.000664919
1753.08531	0.0003019	1753.08531	0.000279968	1753.08531	0.000587602
1755.0139	0.0002572	1755.0139	0.000236319	1755.0139	0.000518024
1756.94249	0.00021848	1756.94249	0.000198841	1756.94249	0.000455581
1758.87107	0.00018503	1758.87107	0.000166772	1758.87107	0.000399696
1760.79966	0.00015625	1760.79966	0.000139428	1760.79966	0.000349816
1762.72825	0.00013155	1762.72825	0.000116195	1762.72825	0.000305418
1764.65683	0.00011042	1764.65683	9.65227E-05	1764.65683	0.000266008
1766.58542	9.2415E-05	1766.58542	7.99239E-05	1766.58542	0.000231119
1768.51401	7.7114E-05	1768.51401	6.59671E-05	1768.51401	0.000200318
1770.44259	6.4155E-05	1770.44259	5.42724E-05	1770.44259	0.000173198
1772.37118	5.3213E-05	1772.37118	4.45073E-05	1772.37118	0.000149385
1774.29977	4.4006E-05	1774.29977	3.63817E-05	1774.29977	0.000128532
1776.22835	3.6283E-05	1776.22835	2.96437E-05	1776.22835	0.00011032
1778.15694	2.9826E-05	1778.15694	2.40757E-05	1778.15694	9.44576E-05
1780.08553	2.4444E-05	1780.08553	1.94904E-05	1780.08553	8.06783E-05
1782.01411	1.9973E-05	1782.01411	1.57274E-05	1782.01411	6.87408E-05
1783.9427	1.6271E-05	1783.9427	1.26499E-05	1783.9427	5.84265E-05
1785.87129	1.3215E-05	1785.87129	1.01417E-05	1785.87129	4.95385E-05
1787.79987	1.0701E-05	1787.79987	8.10455E-06	1787.79987	4.18999E-05
1789.72846	8.6391E-06	1789.72846	6.45561E-06	1789.72846	3.53524E-05
1791.65705	6.9533E-06	1791.65705	5.1255E-06	1791.65705	2.97552E-05
1793.58563	5.5796E-06	1793.58563	4.05627E-06	1793.58563	2.49829E-05
1795.51422	4.4638E-06	1795.51422	3.19968E-06	1795.51422	2.09247E-05
1797.44281	3.5603E-06	1797.44281	2.5158E-06	1797.44281	1.74828E-05
1799.37139	2.8311E-06	1799.37139	1.97167E-06	1799.37139	1.45714E-05

X Observed	Y Generated Coal+TPPI	X Observed	Y Generated Coal+PA
1000.9365	0.0081	1000.9365	0.00649
1002.86509	0.00862	1002.8651	0.00704
1004.79367	0.00913	1004.7937	0.00761
1006.72226	0.00964	1006.7223	0.00818
1008.65085	0.01014	1008.6509	0.00876
1010.57943	0.01064	1010.5794	0.00934
1012.50802	0.01112	1012.508	0.00991
1014.43661	0.01159	1014.4366	0.01047
1016.36519	0.01205	1016.3652	0.01101
1018.29378	0.01248	1018.2938	0.01153
1020.22237	0.01289	1020.2224	0.01203
1022.15095	0.01327	1022.151	0.0125
1024.07954	0.01363	1024.0795	0.01293
1026.00813	0.01396	1026.0081	0.01333
1027.93671	0.01426	1027.9367	0.01368
1029.8653	0.01453	1029.8653	0.014
1031.79389	0.01478	1031.7939	0.01427
1033.72247	0.01499	1033.7225	0.0145
1035.65106	0.01518	1035.6511	0.01469
1037.57965	0.01535	1037.5797	0.01484
1039.50823	0.01549	1039.5082	0.01495
1041.43682	0.01561	1041.4368	0.01502
1043.36541	0.01571	1043.3654	0.01507
1045.29399	0.0158	1045.294	0.01508
1047.22258	0.01588	1047.2226	0.01508
1049.15117	0.01594	1049.1512	0.01505
1051.07975	0.01601	1051.0798	0.01501
1053.00834	0.01607	1053.0083	0.01497
1054.93693	0.01613	1054.9369	0.01492
1056.86551	0.01619	1056.8655	0.01487
1058.7941	0.01626	1058.7941	0.01483
1060.72269	0.01635	1060.7227	0.0148
1062.65127	0.01644	1062.6513	0.01479
1064.57986	0.01654	1064.5799	0.01479
1066.50845	0.01666	1066.5085	0.01481
1068.43703	0.01679	1068.437	0.01485
1070.36562	0.01694	1070.3656	0.01491
1072.29421	0.0171	1072.2942	0.01499
1074.22279	0.01727	1074.2228	0.0151
1076.15138	0.01746	1076.1514	0.01522
1078.07997	0.01765	1078.08	0.01535
1080.00855	0.01784	1080.0086	0.0155
1081.93714	0.01805	1081.9371	0.01566
1083.86573	0.01825	1083.8657	0.01582
1085.79431	0.01845	1085.7943	0.01599
1087.7229	0.01864	1087.7229	0.01615
1089.65149	0.01883	1089.6515	0.01631
1091.58007	0.019	1091.5801	0.01646
1093.50866	0.01916	1093.5087	0.0166
1095.43725	0.0193	1095.4373	0.01672

1097.36584	0.01942	1097.3658	0.01682
1099.29442	0.01952	1099.2944	0.0169
1101.22301	0.0196	1101.223	0.01695
1103.1516	0.01965	1103.1516	0.01698
1105.08018	0.01967	1105.0802	0.01698
1107.00877	0.01967	1107.0088	0.01695
1108.93736	0.01965	1108.9374	0.01689
1110.86594	0.0196	1110.8659	0.01681
1112.79453	0.01952	1112.7945	0.01669
1114.72312	0.01943	1114.7231	0.01656
1116.6517	0.01932	1116.6517	0.0164
1118.58029	0.01919	1118.5803	0.01622
1120.50888	0.01904	1120.5089	0.01603
1122.43746	0.01888	1122.4375	0.01582
1124.36605	0.01872	1124.3661	0.0156
1126.29464	0.01855	1126.2946	0.01537
1128.22322	0.01837	1128.2232	0.01514
1130.15181	0.0182	1130.1518	0.01491
1132.0804	0.01803	1132.0804	0.01468
1134.00898	0.01786	1134.009	0.01445
1135.93757	0.0177	1135.9376	0.01423
1137.86616	0.01755	1137.8662	0.01401
1139.79474	0.01741	1139.7947	0.01381
1141.72333	0.01728	1141.7233	0.01362
1143.65192	0.01716	1143.6519	0.01344
1145.5805	0.01705	1145.5805	0.01328
1147.50909	0.01696	1147.5091	0.01313
1149.43768	0.01687	1149.4377	0.01299
1151.36626	0.0168	1151.3663	0.01286
1153.29485	0.01673	1153.2949	0.01275
1155.22344	0.01666	1155.2234	0.01264
1157.15202	0.0166	1157.152	0.01254
1159.08061	0.01654	1159.0806	0.01244
1161.0092	0.01648	1161.0092	0.01235
1162.93778	0.01642	1162.9378	0.01226
1164.86637	0.01635	1164.8664	0.01217
1166.79496	0.01627	1166.795	0.01207
1168.72354	0.01618	1168.7235	0.01197
1170.65213	0.01608	1170.6521	0.01187
1172.58072	0.01597	1172.5807	0.01175
1174.5093	0.01584	1174.5093	0.01162
1176.43789	0.0157	1176.4379	0.01148
1178.36648	0.01554	1178.3665	0.01133
1180.29506	0.01537	1180.2951	0.01117
1182.22365	0.01517	1182.2237	0.011
1184.15224	0.01497	1184.1522	0.01081
1186.08082	0.01474	1186.0808	0.01061
1188.00941	0.01451	1188.0094	0.0104
1189.938	0.01426	1189.938	0.01018
1191.86658	0.014	1191.8666	0.00996
1193.79517	0.01373	1193.7952	0.00973
1195.72376	0.01346	1195.7238	0.00949
1197.65234	0.01318	1197.6523	0.00925
1199.58093	0.0129	1199.5809	0.00901
1201.50952	0.01262	1201.5095	0.00878
1203.4381	0.01234	1203.4381	0.00854

1205.36669	0.01207	1205.3667	0.00832
1207.29528	0.0118	1207.2953	0.0081
1209.22386	0.01154	1209.2239	0.00789
1211.15245	0.01129	1211.1525	0.00769
1213.08104	0.01105	1213.081	0.0075
1215.00962	0.01082	1215.0096	0.00732
1216.93821	0.01061	1216.9382	0.00715
1218.8668	0.0104	1218.8668	0.007
1220.79538	0.01021	1220.7954	0.00686
1222.72397	0.01002	1222.724	0.00673
1224.65256	0.00985	1224.6526	0.00661
1226.58114	0.00969	1226.5811	0.0065
1228.50973	0.00954	1228.5097	0.0064
1230.43832	0.00939	1230.4383	0.00631
1232.3669	0.00925	1232.3669	0.00623
1234.29549	0.00911	1234.2955	0.00615
1236.22408	0.00898	1236.2241	0.00607
1238.15266	0.00884	1238.1527	0.006
1240.08125	0.00871	1240.0813	0.00593
1242.00984	0.00858	1242.0098	0.00586
1243.93842	0.00845	1243.9384	0.00579
1245.86701	0.00831	1245.867	0.00572
1247.7956	0.00817	1247.7956	0.00565
1249.72418	0.00803	1249.7242	0.00557
1251.65277	0.00788	1251.6528	0.00549
1253.58136	0.00773	1253.5814	0.00541
1255.50994	0.00757	1255.5099	0.00533
1257.43853	0.00742	1257.4385	0.00524
1259.36712	0.00726	1259.3671	0.00515
1261.2957	0.00709	1261.2957	0.00506
1263.22429	0.00693	1263.2243	0.00497
1265.15288	0.00677	1265.1529	0.00488
1267.08146	0.0066	1267.0815	0.00479
1269.01005	0.00644	1269.0101	0.0047
1270.93864	0.00629	1270.9386	0.00462
1272.86722	0.00613	1272.8672	0.00453
1274.79581	0.00598	1274.7958	0.00445
1276.7244	0.00584	1276.7244	0.00437
1278.65298	0.0057	1278.653	0.0043
1280.58157	0.00557	1280.5816	0.00423
1282.51016	0.00545	1282.5102	0.00417
1284.43874	0.00533	1284.4387	0.00411
1286.36733	0.00522	1286.3673	0.00405
1288.29592	0.00512	1288.2959	0.004
1290.22451	0.00502	1290.2245	0.00395
1292.15309	0.00493	1292.1531	0.0039
1294.08168	0.00484	1294.0817	0.00385
1296.01027	0.00476	1296.0103	0.0038
1297.93885	0.00469	1297.9389	0.00375
1299.86744	0.00461	1299.8674	0.0037
1301.79603	0.00455	1301.796	0.00365
1303.72461	0.00448	1303.7246	0.0036
1305.6532	0.00442	1305.6532	0.00354
1307.58179	0.00436	1307.5818	0.00348
1309.51037	0.0043	1309.5104	0.00342
1311.43896	0.00424	1311.439	0.00335

1313.36755	0.00419	1313.3676	0.00328
1315.29613	0.00414	1315.2961	0.00321
1317.22472	0.00409	1317.2247	0.00313
1319.15331	0.00404	1319.1533	0.00305
1321.08189	0.00401	1321.0819	0.00296
1323.01048	0.00397	1323.0105	0.00288
1324.93907	0.00394	1324.9391	0.00279
1326.86765	0.00392	1326.8677	0.00271
1328.79624	0.00391	1328.7962	0.00263
1330.72483	0.0039	1330.7248	0.00256
1332.65341	0.00391	1332.6534	0.0025
1334.582	0.00392	1334.582	0.00244
1336.51059	0.00394	1336.5106	0.0024
1338.43917	0.00398	1338.4392	0.00237
1340.36776	0.00403	1340.3678	0.00236
1342.29635	0.00409	1342.2964	0.00237
1344.22493	0.00416	1344.2249	0.0024
1346.15352	0.00424	1346.1535	0.00245
1348.08211	0.00433	1348.0821	0.00252
1350.01069	0.00443	1350.0107	0.00261
1351.93928	0.00453	1351.9393	0.00273
1353.86787	0.00465	1353.8679	0.00287
1355.79645	0.00477	1355.7965	0.00302
1357.72504	0.0049	1357.725	0.0032
1359.65363	0.00503	1359.6536	0.00339
1361.58221	0.00517	1361.5822	0.00359
1363.5108	0.0053	1363.5108	0.0038
1365.43939	0.00543	1365.4394	0.00401
1367.36797	0.00556	1367.368	0.00422
1369.29656	0.00569	1369.2966	0.00443
1371.22515	0.00581	1371.2252	0.00462
1373.15373	0.00593	1373.1537	0.0048
1375.08232	0.00604	1375.0823	0.00496
1377.01091	0.00614	1377.0109	0.0051
1378.93949	0.00624	1378.9395	0.00521
1380.86808	0.00633	1380.8681	0.0053
1382.79667	0.0064	1382.7967	0.00536
1384.72525	0.00647	1384.7253	0.00539
1386.65384	0.00653	1386.6538	0.0054
1388.58243	0.00659	1388.5824	0.00539
1390.51101	0.00664	1390.511	0.00535
1392.4396	0.00668	1392.4396	0.0053
1394.36819	0.00671	1394.3682	0.00524
1396.29677	0.00674	1396.2968	0.00516
1398.22536	0.00677	1398.2254	0.00509
1400.15395	0.00679	1400.154	0.00501
1402.08253	0.00682	1402.0825	0.00494
1404.01112	0.00684	1404.0111	0.00488
1405.93971	0.00686	1405.9397	0.00483
1407.86829	0.00688	1407.8683	0.0048
1409.79688	0.00691	1409.7969	0.00478
1411.72547	0.00693	1411.7255	0.00478
1413.65405	0.00696	1413.6541	0.0048
1415.58264	0.00699	1415.5826	0.00484
1417.51123	0.00702	1417.5112	0.00489
1419.43981	0.00705	1419.4398	0.00496

1421.3684	0.00709	1421.3684	0.00505
1423.29699	0.00712	1423.297	0.00514
1425.22557	0.00716	1425.2256	0.00524
1427.15416	0.00719	1427.1542	0.00534
1429.08275	0.00722	1429.0828	0.00545
1431.01133	0.00725	1431.0113	0.00555
1432.93992	0.00728	1432.9399	0.00564
1434.86851	0.00729	1434.8685	0.00573
1436.79709	0.00731	1436.7971	0.00581
1438.72568	0.00731	1438.7257	0.00587
1440.65427	0.00731	1440.6543	0.00592
1442.58285	0.0073	1442.5829	0.00595
1444.51144	0.00727	1444.5114	0.00596
1446.44003	0.00724	1446.44	0.00595
1448.36861	0.0072	1448.3686	0.00592
1450.2972	0.00714	1450.2972	0.00587
1452.22579	0.00707	1452.2258	0.00579
1454.15437	0.00699	1454.1544	0.0057
1456.08296	0.0069	1456.083	0.00559
1458.01155	0.0068	1458.0116	0.00546
1459.94013	0.00669	1459.9401	0.00532
1461.86872	0.00657	1461.8687	0.00516
1463.79731	0.00644	1463.7973	0.00499
1465.72589	0.0063	1465.7259	0.0048
1467.65448	0.00616	1467.6545	0.00462
1469.58307	0.00601	1469.5831	0.00442
1471.51165	0.00585	1471.5117	0.00422
1473.44024	0.00569	1473.4402	0.00402
1475.36883	0.00553	1475.3688	0.00383
1477.29741	0.00537	1477.2974	0.00364
1479.226	0.00521	1479.226	0.00345
1481.15459	0.00506	1481.1546	0.00328
1483.08318	0.0049	1483.0832	0.00312
1485.01176	0.00475	1485.0118	0.00297
1486.94035	0.00461	1486.9404	0.00283
1488.86894	0.00447	1488.8689	0.00271
1490.79752	0.00434	1490.7975	0.00261
1492.72611	0.00422	1492.7261	0.00253
1494.6547	0.0041	1494.6547	0.00247
1496.58328	0.004	1496.5833	0.00242
1498.51187	0.0039	1498.5119	0.0024
1500.44046	0.00382	1500.4405	0.00239
1502.36904	0.00374	1502.369	0.00241
1504.29763	0.00368	1504.2976	0.00243
1506.22622	0.00362	1506.2262	0.00248
1508.1548	0.00358	1508.1548	0.00253
1510.08339	0.00355	1510.0834	0.0026
1512.01198	0.00353	1512.012	0.00268
1513.94056	0.00352	1513.9406	0.00277
1515.86915	0.00353	1515.8692	0.00286
1517.79774	0.00355	1517.7977	0.00296
1519.72632	0.00358	1519.7263	0.00306
1521.65491	0.00363	1521.6549	0.00316
1523.5835	0.0037	1523.5835	0.00326
1525.51208	0.00378	1525.5121	0.00336
1527.44067	0.00389	1527.4407	0.00346

1529.36926	0.00401	1529.3693	0.00355
1531.29784	0.00415	1531.2978	0.00365
1533.22643	0.00432	1533.2264	0.00376
1535.15502	0.00452	1535.155	0.00386
1537.0836	0.00474	1537.0836	0.00397
1539.01219	0.00499	1539.0122	0.0041
1540.94078	0.00528	1540.9408	0.00423
1542.86936	0.00559	1542.8694	0.00438
1544.79795	0.00595	1544.798	0.00456
1546.72654	0.00634	1546.7265	0.00475
1548.65512	0.00677	1548.6551	0.00498
1550.58371	0.00724	1550.5837	0.00524
1552.5123	0.00776	1552.5123	0.00553
1554.44088	0.00832	1554.4409	0.00586
1556.36947	0.00892	1556.3695	0.00624
1558.29806	0.00956	1558.2981	0.00666
1560.22664	0.01025	1560.2266	0.00713
1562.15523	0.01098	1562.1552	0.00765
1564.08382	0.01176	1564.0838	0.00822
1566.0124	0.01257	1566.0124	0.00884
1567.94099	0.01342	1567.941	0.0095
1569.86958	0.0143	1569.8696	0.01022
1571.79816	0.01521	1571.7982	0.01098
1573.72675	0.01615	1573.7268	0.01178
1575.65534	0.01711	1575.6553	0.01261
1577.58392	0.01808	1577.5839	0.01349
1579.51251	0.01907	1579.5125	0.01439
1581.4411	0.02005	1581.4411	0.01531
1583.36968	0.02104	1583.3697	0.01626
1585.29827	0.02202	1585.2983	0.01721
1587.22686	0.02298	1587.2269	0.01817
1589.15544	0.02393	1589.1554	0.01913
1591.08403	0.02484	1591.084	0.02008
1593.01262	0.02572	1593.0126	0.02101
1594.9412	0.02656	1594.9412	0.02193
1596.86979	0.02736	1596.8698	0.02281
1598.79838	0.0281	1598.7984	0.02366
1600.72696	0.02879	1600.727	0.02448
1602.65555	0.02941	1602.6556	0.02524
1604.58414	0.02997	1604.5841	0.02596
1606.51272	0.03047	1606.5127	0.02663
1608.44131	0.0309	1608.4413	0.02724
1610.3699	0.03125	1610.3699	0.02779
1612.29848	0.03153	1612.2985	0.02827
1614.22707	0.03174	1614.2271	0.02869
1616.15566	0.03188	1616.1557	0.02905
1618.08424	0.03195	1618.0842	0.02934
1620.01283	0.03195	1620.0128	0.02957
1621.94142	0.03188	1621.9414	0.02973
1623.87	0.03175	1623.87	0.02982
1625.79859	0.03155	1625.7986	0.02986
1627.72718	0.0313	1627.7272	0.02983
1629.65576	0.03099	1629.6558	0.02974
1631.58435	0.03063	1631.5844	0.02959
1633.51294	0.03022	1633.5129	0.02939
1635.44152	0.02976	1635.4415	0.02914

1637.37011	0.02927	1637.3701	0.02883
1639.2987	0.02874	1639.2987	0.02848
1641.22728	0.02818	1641.2273	0.02808
1643.15587	0.02759	1643.1559	0.02763
1645.08446	0.02697	1645.0845	0.02715
1647.01304	0.02633	1647.013	0.02662
1648.94163	0.02567	1648.9416	0.02606
1650.87022	0.02499	1650.8702	0.02546
1652.7988	0.0243	1652.7988	0.02483
1654.72739	0.02359	1654.7274	0.02417
1656.65598	0.02288	1656.656	0.02348
1658.58456	0.02215	1658.5846	0.02277
1660.51315	0.02142	1660.5132	0.02203
1662.44174	0.02068	1662.4417	0.02127
1664.37032	0.01995	1664.3703	0.02049
1666.29891	0.0192	1666.2989	0.0197
1668.2275	0.01846	1668.2275	0.0189
1670.15608	0.01772	1670.1561	0.01808
1672.08467	0.01698	1672.0847	0.01726
1674.01326	0.01625	1674.0133	0.01644
1675.94185	0.01552	1675.9419	0.01562
1677.87043	0.0148	1677.8704	0.0148
1679.79902	0.01408	1679.799	0.01398
1681.72761	0.01338	1681.7276	0.01318
1683.65619	0.01269	1683.6562	0.01238
1685.58478	0.012	1685.5848	0.0116
1687.51337	0.01134	1687.5134	0.01084
1689.44195	0.01068	1689.442	0.0101
1691.37054	0.01005	1691.3705	0.00938
1693.29913	0.00943	1693.2991	0.00869
1695.22771	0.00882	1695.2277	0.00802
1697.1563	0.00824	1697.1563	0.00738
1699.08489	0.00768	1699.0849	0.00678
1701.01347	0.00714	1701.0135	0.0062
1702.94206	0.00662	1702.9421	0.00565
1704.87065	0.00613	1704.8707	0.00513
1706.79923	0.00566	1706.7992	0.00465
1708.72782	0.00521	1708.7278	0.0042
1710.65641	0.00478	1710.6564	0.00378
1712.58499	0.00438	1712.585	0.00339
1714.51358	0.004	1714.5136	0.00303
1716.44217	0.00365	1716.4422	0.0027
1718.37075	0.00331	1718.3708	0.00239
1720.29934	0.003	1720.2993	0.00212
1722.22793	0.00271	1722.2279	0.00187
1724.15651	0.00245	1724.1565	0.00164
1726.0851	0.0022	1726.0851	0.00144
1728.01369	0.00197	1728.0137	0.00125
1729.94227	0.00176	1729.9423	0.00109
1731.87086	0.00157	1731.8709	0.00094383
1733.79945	0.0014	1733.7995	0.0008149
1735.72803	0.00124	1735.728	0.00070112
1737.65662	0.0011	1737.6566	0.0006011
1739.58521	0.00096689	1739.5852	0.00051354
1741.51379	0.00085024	1741.5138	0.00043718
1743.44238	0.00074556	1743.4424	0.00037086

1745.37097	0.00065194	1745.371	0.00031349
1747.29955	0.00056846	1747.2996	0.00026406
1749.22814	0.00049427	1749.2281	0.00022163
1751.15673	0.00042856	1751.1567	0.00018536
1753.08531	0.00037052	1753.0853	0.00015447
1755.0139	0.00031944	1755.0139	0.00012827
1756.94249	0.00027462	1756.9425	0.00010614
1758.87107	0.00023542	1758.8711	8.7511E-05
1760.79966	0.00020124	1760.7997	7.1895E-05
1762.72825	0.00017153	1762.7283	5.8856E-05
1764.65683	0.00014579	1764.6568	4.8009E-05
1766.58542	0.00012356	1766.5854	3.9021E-05
1768.51401	0.00010442	1768.514	3.1603E-05
1770.44259	8.7994E-05	1770.4426	2.5503E-05
1772.37118	7.3941E-05	1772.3712	2.0507E-05
1774.29977	6.1954E-05	1774.2998	1.6431E-05
1776.22835	5.1762E-05	1776.2284	1.3118E-05
1778.15694	4.3123E-05	1778.1569	1.0436E-05
1780.08553	3.5823E-05	1780.0855	8.2718E-06
1782.01411	2.9674E-05	1782.0141	6.5333E-06
1783.9427	0.00002451	1783.9427	5.1416E-06
1785.87129	2.0187E-05	1785.8713	4.032E-06
1787.79987	1.6578E-05	1787.7999	3.1504E-06
1789.72846	1.3576E-05	1789.7285	2.4528E-06
1791.65705	1.1086E-05	1791.6571	1.9029E-06
1793.58563	9.0262E-06	1793.5856	1.4709E-06
1795.51422	7.3283E-06	1795.5142	1.1329E-06
1797.44281	5.9327E-06	1797.4428	8.6949E-07
1799.37139	4.7891E-06	1799.3714	6.6492E-07

Fitting results of IR spectra of water-immersed brown coal with inhibitor

2800-3000

X Observed	Y Generated Coal	X Observed	Y Generated Coal+MgCl2	X Observed	Y Generated Coal+TEMPO
2800.3079	6.8017E-05	2800.3079	6.06155E-06	2800.3079	4.01369E-05
2802.23649	9.2564E-05	2802.23649	1.09918E-05	2802.23649	5.78182E-05
2804.16507	0.00012276	2804.16507	1.90367E-05	2804.16507	8.10095E-05
2806.09366	0.0001587	2806.09366	3.14954E-05	2806.09366	0.0001104
2808.02225	0.00020009	2808.02225	4.97939E-05	2808.02225	0.00014635
2809.95083	0.00024618	2809.95083	7.52625E-05	2809.95083	0.000188731
2811.87942	0.00029588	2811.87942	0.000108829	2811.87942	0.00023681
2813.80801	0.00034785	2813.80801	0.000150698	2813.80801	0.000289193
2815.73659	0.00040083	2815.73659	0.000200119	2815.73659	0.000343898
2817.66518	0.00045394	2817.66518	0.000255392	2817.66518	0.00039856
2819.59377	0.00050713	2819.59377	0.000314191	2819.59377	0.000450805
2821.52235	0.0005615	2821.52235	0.000374242	2821.52235	0.00049877
2823.45094	0.00061959	2823.45094	0.000434217	2823.45094	0.000541717
2825.37953	0.00068545	2825.37953	0.000494646	2825.37953	0.000580652
2827.30811	0.00076437	2827.30811	0.000558523	2827.30811	0.000618829
2829.2367	0.0008624	2829.2367	0.000631364	2829.2367	0.000661982
2831.16529	0.00098551	2831.16529	0.000720569	2831.16529	0.000718124
2833.09387	0.00114	2833.09387	0.000834144	2833.09387	0.000796809
2835.02246	0.00132	2835.02246	0.000979014	2835.02246	0.000907793
2836.95105	0.00154	2836.95105	0.00116	2836.95105	0.00106
2838.87963	0.00179	2838.87963	0.00137	2838.87963	0.00126
2840.80822	0.00205	2840.80822	0.00162	2840.80822	0.0015
2842.73681	0.00232	2842.73681	0.00189	2842.73681	0.00177
2844.66539	0.00258	2844.66539	0.00216	2844.66539	0.00207
2846.59398	0.00282	2846.59398	0.00242	2846.59398	0.00236
2848.52257	0.00302	2848.52257	0.00266	2848.52257	0.00263
2850.45115	0.00317	2850.45115	0.00285	2850.45115	0.00286
2852.37974	0.00327	2852.37974	0.00299	2852.37974	0.00302
2854.30833	0.00331	2854.30833	0.00308	2854.30833	0.00311
2856.23691	0.0033	2856.23691	0.00311	2856.23691	0.00312
2858.1655	0.00324	2858.1655	0.00308	2858.1655	0.00306
2860.09409	0.00316	2860.09409	0.00302	2860.09409	0.00296
2862.02267	0.00305	2862.02267	0.00293	2862.02267	0.00283
2863.95126	0.00293	2863.95126	0.00282	2863.95126	0.00268
2865.87985	0.00282	2865.87985	0.00271	2865.87985	0.00254
2867.80843	0.00271	2867.80843	0.00261	2867.80843	0.00242
2869.73702	0.00261	2869.73702	0.00253	2869.73702	0.00231
2871.66561	0.00253	2871.66561	0.00246	2871.66561	0.00222
2873.59419	0.00246	2873.59419	0.00242	2873.59419	0.00214
2875.52278	0.00241	2875.52278	0.0024	2875.52278	0.00207
2877.45137	0.00237	2877.45137	0.0024	2877.45137	0.00202
2879.37995	0.00235	2879.37995	0.00242	2879.37995	0.00198
2881.30854	0.00235	2881.30854	0.00245	2881.30854	0.00196
2883.23713	0.00237	2883.23713	0.0025	2883.23713	0.00196
2885.16571	0.00241	2885.16571	0.00256	2885.16571	0.00199
2887.0943	0.00248	2887.0943	0.00263	2887.0943	0.00205
2889.02289	0.00257	2889.02289	0.0027	2889.02289	0.00213
2890.95147	0.00267	2890.95147	0.00279	2890.95147	0.00224
2892.88006	0.00279	2892.88006	0.00289	2892.88006	0.00237
2894.80865	0.00292	2894.80865	0.00299	2894.80865	0.00251

2896.73724	0.00306	2896.73724	0.00311	2896.73724	0.00265
2898.66582	0.0032	2898.66582	0.00323	2898.66582	0.00281
2900.59441	0.00336	2900.59441	0.00337	2900.59441	0.00297
2902.523	0.00353	2902.523	0.00353	2902.523	0.00314
2904.45158	0.00371	2904.45158	0.00371	2904.45158	0.00333
2906.38017	0.00393	2906.38017	0.00392	2906.38017	0.00354
2908.30876	0.00417	2908.30876	0.00414	2908.30876	0.00378
2910.23734	0.00443	2910.23734	0.00438	2910.23734	0.00405
2912.16593	0.00472	2912.16593	0.00462	2912.16593	0.00433
2914.09452	0.00501	2914.09452	0.00486	2914.09452	0.00462
2916.0231	0.00529	2916.0231	0.00507	2916.0231	0.0049
2917.95169	0.00554	2917.95169	0.00524	2917.95169	0.00516
2919.88028	0.00573	2919.88028	0.00536	2919.88028	0.00536
2921.80886	0.00584	2921.80886	0.0054	2921.80886	0.00549
2923.73745	0.00587	2923.73745	0.00537	2923.73745	0.00554
2925.66604	0.0058	2925.66604	0.00526	2925.66604	0.0055
2927.59462	0.00562	2927.59462	0.00507	2927.59462	0.00536
2929.52321	0.00536	2929.52321	0.00482	2929.52321	0.00513
2931.4518	0.00503	2931.4518	0.00452	2931.4518	0.00483
2933.38038	0.00464	2933.38038	0.0042	2933.38038	0.00448
2935.30897	0.00424	2935.30897	0.00387	2935.30897	0.00409
2937.23756	0.00385	2937.23756	0.00356	2937.23756	0.00371
2939.16614	0.0035	2939.16614	0.00329	2939.16614	0.00334
2941.09473	0.00321	2941.09473	0.00306	2941.09473	0.00303
2943.02332	0.003	2943.02332	0.00289	2943.02332	0.00279
2944.9519	0.00287	2944.9519	0.00277	2944.9519	0.00263
2946.88049	0.00283	2946.88049	0.00269	2946.88049	0.00256
2948.80908	0.00286	2948.80908	0.00265	2948.80908	0.00257
2950.73766	0.00294	2950.73766	0.00263	2950.73766	0.00265
2952.66625	0.00304	2952.66625	0.00262	2952.66625	0.00277
2954.59484	0.00313	2954.59484	0.0026	2954.59484	0.00292
2956.52342	0.00319	2956.52342	0.00256	2956.52342	0.00305
2958.45201	0.0032	2958.45201	0.0025	2958.45201	0.00314
2960.3806	0.00314	2960.3806	0.0024	2960.3806	0.00316
2962.30918	0.003	2962.30918	0.00228	2962.30918	0.00311
2964.23777	0.00279	2964.23777	0.00212	2964.23777	0.00297
2966.16636	0.00253	2966.16636	0.00193	2966.16636	0.00276
2968.09494	0.00223	2968.09494	0.00173	2968.09494	0.00249
2970.02353	0.0019	2970.02353	0.00152	2970.02353	0.00217
2971.95212	0.00157	2971.95212	0.00131	2971.95212	0.00183
2973.8807	0.00127	2973.8807	0.0011	2973.8807	0.0015
2975.80929	0.00098719	2975.80929	0.000911536	2975.80929	0.00119
2977.73788	0.00074667	2977.73788	0.000737769	2977.73788	0.000908046
2979.66646	0.00054772	2979.66646	0.000584918	2979.66646	0.000673105
2981.59505	0.00038964	2981.59505	0.000454233	2981.59505	0.000482785
2983.52364	0.00026881	2983.52364	0.000345508	2983.52364	0.000335043
2985.45222	0.00017983	2985.45222	0.00025741	2985.45222	0.000224963
2987.38081	0.00011667	2987.38081	0.000187834	2987.38081	0.000146142
2989.3094	7.3395E-05	2989.3094	0.000134246	2989.3094	9.18515E-05
2991.23798	4.4774E-05	2991.23798	9.39735E-05	2991.23798	5.58521E-05
2993.16657	2.6486E-05	2993.16657	6.44294E-05	2993.16657	3.28573E-05
2995.09516	1.5193E-05	2995.09516	4.32649E-05	2995.09516	1.87008E-05
2997.02374	8.4511E-06	2997.02374	2.84552E-05	2997.02374	1.02973E-05
2998.95233	4.5584E-06	2998.95233	1.83298E-05	2998.95233	5.48557E-06
1201.50952	0.01408	1201.50952	0.01118	1201.50952	0.01128
1203.4381	0.01383	1203.4381	0.01087	1203.4381	0.01108

1205.36669	0.01359	1205.36669	0.01057	1205.36669	0.01089
1207.29528	0.01335	1207.29528	0.01028	1207.29528	0.0107
1209.22386	0.01313	1209.22386	0.01	1209.22386	0.01053
1211.15245	0.01292	1211.15245	0.00974	1211.15245	0.01036
1213.08104	0.01273	1213.08104	0.0095	1213.08104	0.0102
1215.00962	0.01254	1215.00962	0.00927	1215.00962	0.01005
1216.93821	0.01238	1216.93821	0.00905	1216.93821	0.0099
1218.8668	0.01222	1218.8668	0.00886	1218.8668	0.00977
1220.79538	0.01208	1220.79538	0.00868	1220.79538	0.00965
1222.72397	0.01195	1222.72397	0.00852	1222.72397	0.00953
1224.65256	0.01183	1224.65256	0.00837	1224.65256	0.00942
1226.58114	0.01173	1226.58114	0.00824	1226.58114	0.00932
1228.50973	0.01163	1228.50973	0.00812	1228.50973	0.00922
1230.43832	0.01154	1230.43832	0.00801	1230.43832	0.00913
1232.3669	0.01146	1232.3669	0.00791	1232.3669	0.00904
1234.29549	0.01138	1234.29549	0.00782	1234.29549	0.00895
1236.22408	0.0113	1236.22408	0.00773	1236.22408	0.00887
1238.15266	0.01122	1238.15266	0.00765	1238.15266	0.00878
1240.08125	0.01115	1240.08125	0.00756	1240.08125	0.00869
1242.00984	0.01107	1242.00984	0.00748	1242.00984	0.0086
1243.93842	0.01098	1243.93842	0.0074	1243.93842	0.0085
1245.86701	0.0109	1245.86701	0.00732	1245.86701	0.0084
1247.7956	0.0108	1247.7956	0.00724	1247.7956	0.0083
1249.72418	0.0107	1249.72418	0.00715	1249.72418	0.00819
1251.65277	0.0106	1251.65277	0.00706	1251.65277	0.00808
1253.58136	0.01049	1253.58136	0.00696	1253.58136	0.00796
1255.50994	0.01037	1255.50994	0.00686	1255.50994	0.00784
1257.43853	0.01024	1257.43853	0.00676	1257.43853	0.00771
1259.36712	0.01011	1259.36712	0.00665	1259.36712	0.00758
1261.2957	0.00998	1261.2957	0.00655	1261.2957	0.00745
1263.22429	0.00984	1263.22429	0.00643	1263.22429	0.00732
1265.15288	0.0097	1265.15288	0.00632	1265.15288	0.00719
1267.08146	0.00955	1267.08146	0.00621	1267.08146	0.00706
1269.01005	0.00941	1269.01005	0.0061	1269.01005	0.00693
1270.93864	0.00926	1270.93864	0.00599	1270.93864	0.0068
1272.86722	0.00912	1272.86722	0.00588	1272.86722	0.00668
1274.79581	0.00898	1274.79581	0.00578	1274.79581	0.00656
1276.7244	0.00884	1276.7244	0.00568	1276.7244	0.00644
1278.65298	0.0087	1278.65298	0.00558	1278.65298	0.00632
1280.58157	0.00857	1280.58157	0.00549	1280.58157	0.00621
1282.51016	0.00844	1282.51016	0.0054	1282.51016	0.00611
1284.43874	0.00832	1284.43874	0.00532	1284.43874	0.00601
1286.36733	0.00819	1286.36733	0.00524	1286.36733	0.00591
1288.29592	0.00807	1288.29592	0.00517	1288.29592	0.00581
1290.22451	0.00796	1290.22451	0.00509	1290.22451	0.00572
1292.15309	0.00784	1292.15309	0.00503	1292.15309	0.00563
1294.08168	0.00773	1294.08168	0.00496	1294.08168	0.00554
1296.01027	0.00761	1296.01027	0.00489	1296.01027	0.00545
1297.93885	0.0075	1297.93885	0.00483	1297.93885	0.00537
1299.86744	0.00738	1299.86744	0.00477	1299.86744	0.00528
1301.79603	0.00726	1301.79603	0.0047	1301.79603	0.00519
1303.72461	0.00714	1303.72461	0.00464	1303.72461	0.0051
1305.6532	0.00702	1305.6532	0.00457	1305.6532	0.005
1307.58179	0.00689	1307.58179	0.0045	1307.58179	0.00491
1309.51037	0.00676	1309.51037	0.00443	1309.51037	0.00481
1311.43896	0.00663	1311.43896	0.00436	1311.43896	0.00472

1313.36755	0.0065	1313.36755	0.00429	1313.36755	0.00462
1315.29613	0.00637	1315.29613	0.00422	1315.29613	0.00452
1317.22472	0.00624	1317.22472	0.00415	1317.22472	0.00443
1319.15331	0.00612	1319.15331	0.00409	1319.15331	0.00434
1321.08189	0.00599	1321.08189	0.00402	1321.08189	0.00425
1323.01048	0.00588	1323.01048	0.00396	1323.01048	0.00417
1324.93907	0.00577	1324.93907	0.00391	1324.93907	0.0041
1326.86765	0.00567	1326.86765	0.00386	1326.86765	0.00403
1328.79624	0.00558	1328.79624	0.00382	1328.79624	0.00398
1330.72483	0.00551	1330.72483	0.00378	1330.72483	0.00394
1332.65341	0.00545	1332.65341	0.00376	1332.65341	0.00391
1334.582	0.00542	1334.582	0.00375	1334.582	0.00389
1336.51059	0.0054	1336.51059	0.00376	1336.51059	0.00389
1338.43917	0.0054	1338.43917	0.00377	1338.43917	0.00391
1340.36776	0.00542	1340.36776	0.0038	1340.36776	0.00394
1342.29635	0.00546	1342.29635	0.00385	1342.29635	0.00399
1344.22493	0.00552	1344.22493	0.00391	1344.22493	0.00406
1346.15352	0.0056	1346.15352	0.00398	1346.15352	0.00414
1348.08211	0.00571	1348.08211	0.00406	1348.08211	0.00424
1350.01069	0.00583	1350.01069	0.00416	1350.01069	0.00436
1351.93928	0.00597	1351.93928	0.00427	1351.93928	0.00448
1353.86787	0.00613	1353.86787	0.00439	1353.86787	0.00462
1355.79645	0.00629	1355.79645	0.00452	1355.79645	0.00477
1357.72504	0.00647	1357.72504	0.00466	1357.72504	0.00492
1359.65363	0.00666	1359.65363	0.0048	1359.65363	0.00508
1361.58221	0.00685	1361.58221	0.00495	1361.58221	0.00524
1363.5108	0.00704	1363.5108	0.0051	1363.5108	0.00541
1365.43939	0.00723	1365.43939	0.00525	1365.43939	0.00557
1367.36797	0.00742	1367.36797	0.0054	1367.36797	0.00572
1369.29656	0.00761	1369.29656	0.00554	1369.29656	0.00587
1371.22515	0.00778	1371.22515	0.00569	1371.22515	0.00601
1373.15373	0.00794	1373.15373	0.00583	1373.15373	0.00614
1375.08232	0.00809	1375.08232	0.00596	1375.08232	0.00626
1377.01091	0.00823	1377.01091	0.00609	1377.01091	0.00636
1378.93949	0.00835	1378.93949	0.0062	1378.93949	0.00645
1380.86808	0.00845	1380.86808	0.00631	1380.86808	0.00653
1382.79667	0.00854	1382.79667	0.00641	1382.79667	0.00659
1384.72525	0.00861	1384.72525	0.00651	1384.72525	0.00663
1386.65384	0.00867	1386.65384	0.00659	1386.65384	0.00667
1388.58243	0.00871	1388.58243	0.00667	1388.58243	0.00669
1390.51101	0.00874	1390.51101	0.00674	1390.51101	0.0067
1392.4396	0.00876	1392.4396	0.0068	1392.4396	0.0067
1394.36819	0.00877	1394.36819	0.00685	1394.36819	0.00669
1396.29677	0.00877	1396.29677	0.00691	1396.29677	0.00667
1398.22536	0.00876	1398.22536	0.00695	1398.22536	0.00665
1400.15395	0.00875	1400.15395	0.007	1400.15395	0.00663
1402.08253	0.00874	1402.08253	0.00704	1402.08253	0.00661
1404.01112	0.00873	1404.01112	0.00708	1404.01112	0.00659
1405.93971	0.00872	1405.93971	0.00712	1405.93971	0.00657
1407.86829	0.00871	1407.86829	0.00716	1407.86829	0.00655
1409.79688	0.0087	1409.79688	0.0072	1409.79688	0.00654
1411.72547	0.0087	1411.72547	0.00724	1411.72547	0.00654
1413.65405	0.00871	1413.65405	0.00728	1413.65405	0.00654
1415.58264	0.00872	1415.58264	0.00732	1415.58264	0.00655
1417.51123	0.00873	1417.51123	0.00737	1417.51123	0.00657
1419.43981	0.00876	1419.43981	0.00741	1419.43981	0.00659

1421.3684	0.00878	1421.3684	0.00745	1421.3684	0.00662
1423.29699	0.00881	1423.29699	0.00748	1423.29699	0.00665
1425.22557	0.00884	1425.22557	0.00751	1425.22557	0.00669
1427.15416	0.00887	1427.15416	0.00754	1427.15416	0.00673
1429.08275	0.0089	1429.08275	0.00756	1429.08275	0.00676
1431.01133	0.00893	1431.01133	0.00758	1431.01133	0.0068
1432.93992	0.00896	1432.93992	0.00758	1432.93992	0.00683
1434.86851	0.00897	1434.86851	0.00757	1434.86851	0.00686
1436.79709	0.00899	1436.79709	0.00755	1436.79709	0.00689
1438.72568	0.00899	1438.72568	0.00752	1438.72568	0.0069
1440.65427	0.00898	1440.65427	0.00747	1440.65427	0.00691
1442.58285	0.00895	1442.58285	0.00741	1442.58285	0.0069
1444.51144	0.00892	1444.51144	0.00733	1444.51144	0.00688
1446.44003	0.00886	1446.44003	0.00724	1446.44003	0.00685
1448.36861	0.0088	1448.36861	0.00713	1448.36861	0.00681
1450.2972	0.00871	1450.2972	0.00701	1450.2972	0.00675
1452.22579	0.00861	1452.22579	0.00687	1452.22579	0.00668
1454.15437	0.0085	1454.15437	0.00671	1454.15437	0.00659
1456.08296	0.00836	1456.08296	0.00655	1456.08296	0.00649
1458.01155	0.00821	1458.01155	0.00637	1458.01155	0.00637
1459.94013	0.00805	1459.94013	0.00618	1459.94013	0.00625
1461.86872	0.00787	1461.86872	0.00598	1461.86872	0.00611
1463.79731	0.00768	1463.79731	0.00577	1463.79731	0.00597
1465.72589	0.00748	1465.72589	0.00556	1465.72589	0.00581
1467.65448	0.00726	1467.65448	0.00535	1467.65448	0.00565
1469.58307	0.00705	1469.58307	0.00513	1469.58307	0.00549
1471.51165	0.00682	1471.51165	0.00491	1471.51165	0.00532
1473.44024	0.00659	1473.44024	0.0047	1473.44024	0.00515
1475.36883	0.00637	1475.36883	0.00449	1475.36883	0.00498
1477.29741	0.00614	1477.29741	0.00429	1477.29741	0.00481
1479.226	0.00592	1479.226	0.00409	1479.226	0.00464
1481.15459	0.00571	1481.15459	0.0039	1481.15459	0.00448
1483.08318	0.00551	1483.08318	0.00372	1483.08318	0.00433
1485.01176	0.00532	1485.01176	0.00355	1485.01176	0.00418
1486.94035	0.00515	1486.94035	0.00339	1486.94035	0.00404
1488.86894	0.00499	1488.86894	0.00325	1488.86894	0.00391
1490.79752	0.00485	1490.79752	0.00312	1490.79752	0.00379
1492.72611	0.00473	1492.72611	0.003	1492.72611	0.00368
1494.6547	0.00463	1494.6547	0.00289	1494.6547	0.00358
1496.58328	0.00456	1496.58328	0.0028	1496.58328	0.00349
1498.51187	0.00451	1498.51187	0.00272	1498.51187	0.00341
1500.44046	0.00448	1500.44046	0.00265	1500.44046	0.00334
1502.36904	0.00448	1502.36904	0.0026	1502.36904	0.00329
1504.29763	0.0045	1504.29763	0.00256	1504.29763	0.00324
1506.22622	0.00454	1506.22622	0.00253	1506.22622	0.0032
1508.1548	0.00461	1508.1548	0.00252	1508.1548	0.00318
1510.08339	0.00469	1510.08339	0.00251	1510.08339	0.00316
1512.01198	0.00479	1512.01198	0.00252	1512.01198	0.00315
1513.94056	0.00491	1513.94056	0.00255	1513.94056	0.00315
1515.86915	0.00504	1515.86915	0.00258	1515.86915	0.00316
1517.79774	0.00519	1517.79774	0.00263	1517.79774	0.00317
1519.72632	0.00535	1519.72632	0.00269	1519.72632	0.0032
1521.65491	0.00553	1521.65491	0.00276	1521.65491	0.00323
1523.5835	0.00571	1523.5835	0.00285	1523.5835	0.00328
1525.51208	0.00591	1525.51208	0.00295	1525.51208	0.00333
1527.44067	0.00612	1527.44067	0.00307	1527.44067	0.00339

1529.36926	0.00634	1529.36926	0.00321	1529.36926	0.00347
1531.29784	0.00657	1531.29784	0.00337	1531.29784	0.00356
1533.22643	0.00682	1533.22643	0.00355	1533.22643	0.00367
1535.15502	0.00709	1535.15502	0.00375	1535.15502	0.00379
1537.0836	0.00738	1537.0836	0.00398	1537.0836	0.00393
1539.01219	0.00769	1539.01219	0.00424	1539.01219	0.0041
1540.94078	0.00802	1540.94078	0.00453	1540.94078	0.00429
1542.86936	0.00839	1542.86936	0.00485	1542.86936	0.0045
1544.79795	0.00878	1544.79795	0.0052	1544.79795	0.00475
1546.72654	0.00921	1546.72654	0.00559	1546.72654	0.00503
1548.65512	0.00967	1548.65512	0.00602	1548.65512	0.00534
1550.58371	0.01017	1550.58371	0.00649	1550.58371	0.00569
1552.5123	0.01071	1552.5123	0.007	1552.5123	0.00608
1554.44088	0.01129	1554.44088	0.00756	1554.44088	0.00651
1556.36947	0.01191	1556.36947	0.00816	1556.36947	0.00699
1558.29806	0.01257	1558.29806	0.00881	1558.29806	0.00752
1560.22664	0.01327	1560.22664	0.0095	1560.22664	0.0081
1562.15523	0.01401	1562.15523	0.01025	1562.15523	0.00872
1564.08382	0.01479	1564.08382	0.01104	1564.08382	0.0094
1566.0124	0.01561	1566.0124	0.01188	1566.0124	0.01012
1567.94099	0.01646	1567.94099	0.01277	1567.94099	0.0109
1569.86958	0.01733	1569.86958	0.0137	1569.86958	0.01172
1571.79816	0.01823	1571.79816	0.01467	1571.79816	0.01259
1573.72675	0.01916	1573.72675	0.01568	1573.72675	0.0135
1575.65534	0.0201	1575.65534	0.01673	1575.65534	0.01445
1577.58392	0.02105	1577.58392	0.01782	1577.58392	0.01544
1579.51251	0.02201	1579.51251	0.01893	1579.51251	0.01646
1581.4411	0.02297	1581.4411	0.02006	1581.4411	0.0175
1583.36968	0.02394	1583.36968	0.02122	1583.36968	0.01856
1585.29827	0.02489	1585.29827	0.02238	1585.29827	0.01964
1587.22686	0.02583	1587.22686	0.02356	1587.22686	0.02072
1589.15544	0.02676	1589.15544	0.02473	1589.15544	0.0218
1591.08403	0.02766	1591.08403	0.0259	1591.08403	0.02287
1593.01262	0.02854	1593.01262	0.02706	1593.01262	0.02392
1594.9412	0.02939	1594.9412	0.02819	1594.9412	0.02494
1596.86979	0.0302	1596.86979	0.02931	1596.86979	0.02593
1598.79838	0.03097	1598.79838	0.03039	1598.79838	0.02688
1600.72696	0.0317	1600.72696	0.03143	1600.72696	0.02778
1602.65555	0.03239	1602.65555	0.03242	1602.65555	0.02862
1604.58414	0.03302	1604.58414	0.03337	1604.58414	0.02939
1606.51272	0.0336	1606.51272	0.03426	1606.51272	0.0301
1608.44131	0.03413	1608.44131	0.03508	1608.44131	0.03073
1610.3699	0.03459	1610.3699	0.03584	1610.3699	0.03128
1612.29848	0.035	1612.29848	0.03654	1612.29848	0.03175
1614.22707	0.03535	1614.22707	0.03715	1614.22707	0.03213
1616.15566	0.03563	1616.15566	0.03769	1616.15566	0.03242
1618.08424	0.03585	1618.08424	0.03815	1618.08424	0.03263
1620.01283	0.03601	1620.01283	0.03853	1620.01283	0.03274
1621.94142	0.0361	1621.94142	0.03882	1621.94142	0.03277
1623.87	0.03613	1623.87	0.03903	1623.87	0.03271
1625.79859	0.0361	1625.79859	0.03916	1625.79859	0.03257
1627.72718	0.03599	1627.72718	0.0392	1627.72718	0.03235
1629.65576	0.03583	1629.65576	0.03916	1629.65576	0.03205
1631.58435	0.0356	1631.58435	0.03904	1631.58435	0.03168
1633.51294	0.03532	1633.51294	0.03883	1633.51294	0.03124
1635.44152	0.03497	1635.44152	0.03855	1635.44152	0.03075

1637.37011	0.03457	1637.37011	0.03819	1637.37011	0.03019
1639.2987	0.03411	1639.2987	0.03776	1639.2987	0.02959
1641.22728	0.03359	1641.22728	0.03725	1641.22728	0.02894
1643.15587	0.03303	1643.15587	0.03668	1643.15587	0.02826
1645.08446	0.03241	1645.08446	0.03605	1645.08446	0.02754
1647.01304	0.03175	1647.01304	0.03536	1647.01304	0.0268
1648.94163	0.03105	1648.94163	0.0346	1648.94163	0.02603
1650.87022	0.0303	1650.87022	0.0338	1650.87022	0.02525
1652.7988	0.02952	1652.7988	0.03295	1652.7988	0.02446
1654.72739	0.0287	1654.72739	0.03206	1654.72739	0.02365
1656.65598	0.02786	1656.65598	0.03112	1656.65598	0.02285
1658.58456	0.02698	1658.58456	0.03015	1658.58456	0.02204
1660.51315	0.02609	1660.51315	0.02915	1660.51315	0.02124
1662.44174	0.02517	1662.44174	0.02813	1662.44174	0.02044
1664.37032	0.02423	1664.37032	0.02708	1664.37032	0.01965
1666.29891	0.02328	1666.29891	0.02602	1666.29891	0.01887
1668.2275	0.02233	1668.2275	0.02494	1668.2275	0.01809
1670.15608	0.02136	1670.15608	0.02385	1670.15608	0.01734
1672.08467	0.0204	1672.08467	0.02276	1672.08467	0.01659
1674.01326	0.01944	1674.01326	0.02167	1674.01326	0.01586
1675.94185	0.01848	1675.94185	0.02059	1675.94185	0.01514
1677.87043	0.01753	1677.87043	0.01951	1677.87043	0.01445
1679.79902	0.01659	1679.79902	0.01845	1679.79902	0.01376
1681.72761	0.01566	1681.72761	0.0174	1681.72761	0.01309
1683.65619	0.01476	1683.65619	0.01637	1683.65619	0.01244
1685.58478	0.01387	1685.58478	0.01536	1685.58478	0.01181
1687.51337	0.013	1687.51337	0.01438	1687.51337	0.01119
1689.44195	0.01216	1689.44195	0.01343	1689.44195	0.0106
1691.37054	0.01135	1691.37054	0.01251	1691.37054	0.01001
1693.29913	0.01056	1693.29913	0.01162	1693.29913	0.00945
1695.22771	0.00981	1695.22771	0.01076	1695.22771	0.0089
1697.1563	0.00909	1697.1563	0.00994	1697.1563	0.00838
1699.08489	0.00839	1699.08489	0.00916	1699.08489	0.00787
1701.01347	0.00773	1701.01347	0.00842	1701.01347	0.00738
1702.94206	0.00711	1702.94206	0.00771	1702.94206	0.0069
1704.87065	0.00652	1704.87065	0.00705	1704.87065	0.00645
1706.79923	0.00596	1706.79923	0.00642	1706.79923	0.00601
1708.72782	0.00543	1708.72782	0.00584	1708.72782	0.0056
1710.65641	0.00494	1710.65641	0.00529	1710.65641	0.0052
1712.58499	0.00448	1712.58499	0.00478	1712.58499	0.00482
1714.51358	0.00405	1714.51358	0.0043	1714.51358	0.00446
1716.44217	0.00366	1716.44217	0.00386	1716.44217	0.00412
1718.37075	0.00329	1718.37075	0.00346	1718.37075	0.00379
1720.29934	0.00295	1720.29934	0.00309	1720.29934	0.00349
1722.22793	0.00264	1722.22793	0.00275	1722.22793	0.0032
1724.15651	0.00236	1724.15651	0.00244	1724.15651	0.00293
1726.0851	0.0021	1726.0851	0.00216	1726.0851	0.00267
1728.01369	0.00186	1728.01369	0.0019	1728.01369	0.00243
1729.94227	0.00164	1729.94227	0.00167	1729.94227	0.00221
1731.87086	0.00145	1731.87086	0.00147	1731.87086	0.00201
1733.79945	0.00128	1733.79945	0.00128	1733.79945	0.00182
1735.72803	0.00112	1735.72803	0.00112	1735.72803	0.00164
1737.65662	0.00097895	1737.65662	0.000969225	1737.65662	0.00148
1739.58521	0.00085373	1739.58521	0.000839069	1739.58521	0.00133
1741.51379	0.00074237	1741.51379	0.00072412	1741.51379	0.00119
1743.44238	0.00064367	1743.44238	0.000622957	1743.44238	0.00106

1745.37097	0.00055648	1745.37097	0.000534242	1745.37097	0.00094964
1747.29955	0.00047969	1747.29955	0.000456715	1747.29955	0.000845286
1749.22814	0.00041229	1749.22814	0.000389205	1749.22814	0.000750599
1751.15673	0.00035332	1751.15673	0.000330623	1751.15673	0.000664919
1753.08531	0.0003019	1753.08531	0.000279968	1753.08531	0.000587602
1755.0139	0.0002572	1755.0139	0.000236319	1755.0139	0.000518024
1756.94249	0.00021848	1756.94249	0.000198841	1756.94249	0.000455581
1758.87107	0.00018503	1758.87107	0.000166772	1758.87107	0.000399696
1760.79966	0.00015625	1760.79966	0.000139428	1760.79966	0.000349816
1762.72825	0.00013155	1762.72825	0.000116195	1762.72825	0.000305418
1764.65683	0.00011042	1764.65683	9.65227E-05	1764.65683	0.000266008
1766.58542	9.2415E-05	1766.58542	7.99239E-05	1766.58542	0.000231119
1768.51401	7.7114E-05	1768.51401	6.59671E-05	1768.51401	0.000200318
1770.44259	6.4155E-05	1770.44259	5.42724E-05	1770.44259	0.000173198
1772.37118	5.3213E-05	1772.37118	4.45073E-05	1772.37118	0.000149385
1774.29977	4.4006E-05	1774.29977	3.63817E-05	1774.29977	0.000128532
1776.22835	3.6283E-05	1776.22835	2.96437E-05	1776.22835	0.00011032
1778.15694	2.9826E-05	1778.15694	2.40757E-05	1778.15694	9.44576E-05
1780.08553	2.4444E-05	1780.08553	1.94904E-05	1780.08553	8.06783E-05
1782.01411	1.9973E-05	1782.01411	1.57274E-05	1782.01411	6.87408E-05
1783.9427	1.6271E-05	1783.9427	1.26499E-05	1783.9427	5.84265E-05
1785.87129	1.3215E-05	1785.87129	1.01417E-05	1785.87129	4.95385E-05
1787.79987	1.0701E-05	1787.79987	8.10455E-06	1787.79987	4.18999E-05
1789.72846	8.6391E-06	1789.72846	6.45561E-06	1789.72846	3.53524E-05
1791.65705	6.9533E-06	1791.65705	5.1255E-06	1791.65705	2.97552E-05
1793.58563	5.5796E-06	1793.58563	4.05627E-06	1793.58563	2.49829E-05
1795.51422	4.4638E-06	1795.51422	3.19968E-06	1795.51422	2.09247E-05
1797.44281	3.5603E-06	1797.44281	2.5158E-06	1797.44281	1.74828E-05
1799.37139	2.8311E-06	1799.37139	1.97167E-06	1799.37139	1.45714E-05

X Observed	Y Generated Coal+TPPI	X Observed	Y Generated Coal+PA
2800.3079	4.7786E-05	2800.3079	1.5563E-06
2802.23649	6.7328E-05	2802.2365	3.5422E-06
2804.16507	9.2375E-05	2804.1651	7.4979E-06
2806.09366	0.00012343	2806.0937	1.4761E-05
2808.02225	0.00016065	2808.0223	2.7033E-05
2809.95083	0.00020375	2809.9508	4.6075E-05
2811.87942	0.00025191	2811.8794	7.3139E-05
2813.80801	0.00030385	2813.808	0.00010827
2815.73659	0.00035795	2815.7366	0.00014977
2817.66518	0.00041253	2817.6652	0.00019426
2819.59377	0.00046626	2819.5938	0.00023758
2821.52235	0.00051862	2821.5224	0.0002764
2823.45094	0.00057038	2823.4509	0.00031013
2825.37953	0.00062401	2825.3795	0.00034214
2827.30811	0.0006838	2827.3081	0.00037983
2829.2367	0.00075575	2829.2367	0.0004333
2831.16529	0.00084703	2831.1653	0.00051319
2833.09387	0.00096499	2833.0939	0.00062819
2835.02246	0.00112	2835.0225	0.00078318
2836.95105	0.0013	2836.9511	0.00097818
2838.87963	0.00153	2838.8796	0.00121
2840.80822	0.00178	2840.8082	0.00146
2842.73681	0.00206	2842.7368	0.00173
2844.66539	0.00233	2844.6654	0.00199
2846.59398	0.0026	2846.594	0.00223
2848.52257	0.00283	2848.5226	0.00244
2850.45115	0.003	2850.4512	0.00259
2852.37974	0.00311	2852.3797	0.00269
2854.30833	0.00316	2854.3083	0.00273
2856.23691	0.00313	2856.2369	0.00271
2858.1655	0.00304	2858.1655	0.00264
2860.09409	0.00292	2860.0941	0.00253
2862.02267	0.00277	2862.0227	0.00241
2863.95126	0.00262	2863.9513	0.00228
2865.87985	0.00248	2865.8799	0.00215
2867.80843	0.00236	2867.8084	0.00204
2869.73702	0.00227	2869.737	0.00195
2871.66561	0.00219	2871.6656	0.00189
2873.59419	0.00214	2873.5942	0.00184
2875.52278	0.00209	2875.5228	0.00181
2877.45137	0.00205	2877.4514	0.0018
2879.37995	0.00202	2879.38	0.00181
2881.30854	0.00201	2881.3085	0.00183
2883.23713	0.00202	2883.2371	0.00187
2885.16571	0.00206	2885.1657	0.00192
2887.0943	0.00213	2887.0943	0.00199
2889.02289	0.00223	2889.0229	0.00208
2890.95147	0.00236	2890.9515	0.00218
2892.88006	0.0025	2892.8801	0.00228
2894.80865	0.00264	2894.8087	0.0024

2896.73724	0.00279	2896.7372	0.00251
2898.66582	0.00293	2898.6658	0.00263
2900.59441	0.00307	2900.5944	0.00275
2902.523	0.00322	2902.523	0.00288
2904.45158	0.00339	2904.4516	0.00303
2906.38017	0.00358	2906.3802	0.0032
2908.30876	0.00382	2908.3088	0.00341
2910.23734	0.00409	2910.2373	0.00364
2912.16593	0.0044	2912.1659	0.0039
2914.09452	0.00471	2914.0945	0.00417
2916.0231	0.00502	2916.0231	0.00445
2917.95169	0.0053	2917.9517	0.00471
2919.88028	0.00552	2919.8803	0.00492
2921.80886	0.00566	2921.8089	0.00507
2923.73745	0.0057	2923.7375	0.00513
2925.66604	0.00564	2925.666	0.00511
2927.59462	0.00547	2927.5946	0.00498
2929.52321	0.00521	2929.5232	0.00476
2931.4518	0.00487	2931.4518	0.00446
2933.38038	0.00448	2933.3804	0.00412
2935.30897	0.00407	2935.309	0.00375
2937.23756	0.00367	2937.2376	0.00338
2939.16614	0.00331	2939.1661	0.00305
2941.09473	0.00301	2941.0947	0.00279
2943.02332	0.0028	2943.0233	0.00259
2944.9519	0.00268	2944.9519	0.00249
2946.88049	0.00265	2946.8805	0.00246
2948.80908	0.0027	2948.8091	0.00251
2950.73766	0.00282	2950.7377	0.00262
2952.66625	0.00297	2952.6663	0.00274
2954.59484	0.00312	2954.5948	0.00287
2956.52342	0.00323	2956.5234	0.00298
2958.45201	0.00329	2958.452	0.00303
2960.3806	0.00326	2960.3806	0.00302
2962.30918	0.00315	2962.3092	0.00294
2964.23777	0.00295	2964.2378	0.00278
2966.16636	0.00268	2966.1664	0.00256
2968.09494	0.00235	2968.0949	0.00229
2970.02353	0.002	2970.0235	0.00199
2971.95212	0.00165	2971.9521	0.00168
2973.8807	0.00131	2973.8807	0.00138
2975.80929	0.00101	2975.8093	0.0011
2977.73788	0.00075087	2977.7379	0.0008465
2979.66646	0.00054001	2979.6665	0.00063473
2981.59505	0.00037542	2981.5951	0.00046198
2983.52364	0.0002523	2983.5236	0.00032638
2985.45222	0.0001639	2985.4522	0.00022381
2987.38081	0.00010292	2987.3808	0.00014897
2989.3094	6.2467E-05	2989.3094	9.6243E-05
2991.23798	3.6649E-05	2991.238	6.0352E-05
2993.16657	2.0784E-05	2993.1666	3.6734E-05
2995.09516	1.1393E-05	2995.0952	2.1702E-05
2997.02374	6.0362E-06	2997.0237	1.2445E-05
2998.95233	3.0914E-06	2998.9523	6.9264E-06
1201.50952	0.01262	1201.5095	0.00878
1203.4381	0.01234	1203.4381	0.00854

1205.36669	0.01207	1205.3667	0.00832
1207.29528	0.0118	1207.2953	0.0081
1209.22386	0.01154	1209.2239	0.00789
1211.15245	0.01129	1211.1525	0.00769
1213.08104	0.01105	1213.081	0.0075
1215.00962	0.01082	1215.0096	0.00732
1216.93821	0.01061	1216.9382	0.00715
1218.8668	0.0104	1218.8668	0.007
1220.79538	0.01021	1220.7954	0.00686
1222.72397	0.01002	1222.724	0.00673
1224.65256	0.00985	1224.6526	0.00661
1226.58114	0.00969	1226.5811	0.0065
1228.50973	0.00954	1228.5097	0.0064
1230.43832	0.00939	1230.4383	0.00631
1232.3669	0.00925	1232.3669	0.00623
1234.29549	0.00911	1234.2955	0.00615
1236.22408	0.00898	1236.2241	0.00607
1238.15266	0.00884	1238.1527	0.006
1240.08125	0.00871	1240.0813	0.00593
1242.00984	0.00858	1242.0098	0.00586
1243.93842	0.00845	1243.9384	0.00579
1245.86701	0.00831	1245.867	0.00572
1247.7956	0.00817	1247.7956	0.00565
1249.72418	0.00803	1249.7242	0.00557
1251.65277	0.00788	1251.6528	0.00549
1253.58136	0.00773	1253.5814	0.00541
1255.50994	0.00757	1255.5099	0.00533
1257.43853	0.00742	1257.4385	0.00524
1259.36712	0.00726	1259.3671	0.00515
1261.2957	0.00709	1261.2957	0.00506
1263.22429	0.00693	1263.2243	0.00497
1265.15288	0.00677	1265.1529	0.00488
1267.08146	0.0066	1267.0815	0.00479
1269.01005	0.00644	1269.0101	0.0047
1270.93864	0.00629	1270.9386	0.00462
1272.86722	0.00613	1272.8672	0.00453
1274.79581	0.00598	1274.7958	0.00445
1276.7244	0.00584	1276.7244	0.00437
1278.65298	0.0057	1278.653	0.0043
1280.58157	0.00557	1280.5816	0.00423
1282.51016	0.00545	1282.5102	0.00417
1284.43874	0.00533	1284.4387	0.00411
1286.36733	0.00522	1286.3673	0.00405
1288.29592	0.00512	1288.2959	0.004
1290.22451	0.00502	1290.2245	0.00395
1292.15309	0.00493	1292.1531	0.0039
1294.08168	0.00484	1294.0817	0.00385
1296.01027	0.00476	1296.0103	0.0038
1297.93885	0.00469	1297.9389	0.00375
1299.86744	0.00461	1299.8674	0.0037
1301.79603	0.00455	1301.796	0.00365
1303.72461	0.00448	1303.7246	0.0036
1305.6532	0.00442	1305.6532	0.00354
1307.58179	0.00436	1307.5818	0.00348
1309.51037	0.0043	1309.5104	0.00342
1311.43896	0.00424	1311.439	0.00335

1313.36755	0.00419	1313.3676	0.00328
1315.29613	0.00414	1315.2961	0.00321
1317.22472	0.00409	1317.2247	0.00313
1319.15331	0.00404	1319.1533	0.00305
1321.08189	0.00401	1321.0819	0.00296
1323.01048	0.00397	1323.0105	0.00288
1324.93907	0.00394	1324.9391	0.00279
1326.86765	0.00392	1326.8677	0.00271
1328.79624	0.00391	1328.7962	0.00263
1330.72483	0.0039	1330.7248	0.00256
1332.65341	0.00391	1332.6534	0.0025
1334.582	0.00392	1334.582	0.00244
1336.51059	0.00394	1336.5106	0.0024
1338.43917	0.00398	1338.4392	0.00237
1340.36776	0.00403	1340.3678	0.00236
1342.29635	0.00409	1342.2964	0.00237
1344.22493	0.00416	1344.2249	0.0024
1346.15352	0.00424	1346.1535	0.00245
1348.08211	0.00433	1348.0821	0.00252
1350.01069	0.00443	1350.0107	0.00261
1351.93928	0.00453	1351.9393	0.00273
1353.86787	0.00465	1353.8679	0.00287
1355.79645	0.00477	1355.7965	0.00302
1357.72504	0.0049	1357.725	0.0032
1359.65363	0.00503	1359.6536	0.00339
1361.58221	0.00517	1361.5822	0.00359
1363.5108	0.0053	1363.5108	0.0038
1365.43939	0.00543	1365.4394	0.00401
1367.36797	0.00556	1367.368	0.00422
1369.29656	0.00569	1369.2966	0.00443
1371.22515	0.00581	1371.2252	0.00462
1373.15373	0.00593	1373.1537	0.0048
1375.08232	0.00604	1375.0823	0.00496
1377.01091	0.00614	1377.0109	0.0051
1378.93949	0.00624	1378.9395	0.00521
1380.86808	0.00633	1380.8681	0.0053
1382.79667	0.0064	1382.7967	0.00536
1384.72525	0.00647	1384.7253	0.00539
1386.65384	0.00653	1386.6538	0.0054
1388.58243	0.00659	1388.5824	0.00539
1390.51101	0.00664	1390.511	0.00535
1392.4396	0.00668	1392.4396	0.0053
1394.36819	0.00671	1394.3682	0.00524
1396.29677	0.00674	1396.2968	0.00516
1398.22536	0.00677	1398.2254	0.00509
1400.15395	0.00679	1400.154	0.00501
1402.08253	0.00682	1402.0825	0.00494
1404.01112	0.00684	1404.0111	0.00488
1405.93971	0.00686	1405.9397	0.00483
1407.86829	0.00688	1407.8683	0.0048
1409.79688	0.00691	1409.7969	0.00478
1411.72547	0.00693	1411.7255	0.00478
1413.65405	0.00696	1413.6541	0.0048
1415.58264	0.00699	1415.5826	0.00484
1417.51123	0.00702	1417.5112	0.00489
1419.43981	0.00705	1419.4398	0.00496

1421.3684	0.00709	1421.3684	0.00505
1423.29699	0.00712	1423.297	0.00514
1425.22557	0.00716	1425.2256	0.00524
1427.15416	0.00719	1427.1542	0.00534
1429.08275	0.00722	1429.0828	0.00545
1431.01133	0.00725	1431.0113	0.00555
1432.93992	0.00728	1432.9399	0.00564
1434.86851	0.00729	1434.8685	0.00573
1436.79709	0.00731	1436.7971	0.00581
1438.72568	0.00731	1438.7257	0.00587
1440.65427	0.00731	1440.6543	0.00592
1442.58285	0.0073	1442.5829	0.00595
1444.51144	0.00727	1444.5114	0.00596
1446.44003	0.00724	1446.44	0.00595
1448.36861	0.0072	1448.3686	0.00592
1450.2972	0.00714	1450.2972	0.00587
1452.22579	0.00707	1452.2258	0.00579
1454.15437	0.00699	1454.1544	0.0057
1456.08296	0.0069	1456.083	0.00559
1458.01155	0.0068	1458.0116	0.00546
1459.94013	0.00669	1459.9401	0.00532
1461.86872	0.00657	1461.8687	0.00516
1463.79731	0.00644	1463.7973	0.00499
1465.72589	0.0063	1465.7259	0.0048
1467.65448	0.00616	1467.6545	0.00462
1469.58307	0.00601	1469.5831	0.00442
1471.51165	0.00585	1471.5117	0.00422
1473.44024	0.00569	1473.4402	0.00402
1475.36883	0.00553	1475.3688	0.00383
1477.29741	0.00537	1477.2974	0.00364
1479.226	0.00521	1479.226	0.00345
1481.15459	0.00506	1481.1546	0.00328
1483.08318	0.0049	1483.0832	0.00312
1485.01176	0.00475	1485.0118	0.00297
1486.94035	0.00461	1486.9404	0.00283
1488.86894	0.00447	1488.8689	0.00271
1490.79752	0.00434	1490.7975	0.00261
1492.72611	0.00422	1492.7261	0.00253
1494.6547	0.0041	1494.6547	0.00247
1496.58328	0.004	1496.5833	0.00242
1498.51187	0.0039	1498.5119	0.0024
1500.44046	0.00382	1500.4405	0.00239
1502.36904	0.00374	1502.369	0.00241
1504.29763	0.00368	1504.2976	0.00243
1506.22622	0.00362	1506.2262	0.00248
1508.1548	0.00358	1508.1548	0.00253
1510.08339	0.00355	1510.0834	0.0026
1512.01198	0.00353	1512.012	0.00268
1513.94056	0.00352	1513.9406	0.00277
1515.86915	0.00353	1515.8692	0.00286
1517.79774	0.00355	1517.7977	0.00296
1519.72632	0.00358	1519.7263	0.00306
1521.65491	0.00363	1521.6549	0.00316
1523.5835	0.0037	1523.5835	0.00326
1525.51208	0.00378	1525.5121	0.00336
1527.44067	0.00389	1527.4407	0.00346

1529.36926	0.00401	1529.3693	0.00355
1531.29784	0.00415	1531.2978	0.00365
1533.22643	0.00432	1533.2264	0.00376
1535.15502	0.00452	1535.155	0.00386
1537.0836	0.00474	1537.0836	0.00397
1539.01219	0.00499	1539.0122	0.0041
1540.94078	0.00528	1540.9408	0.00423
1542.86936	0.00559	1542.8694	0.00438
1544.79795	0.00595	1544.798	0.00456
1546.72654	0.00634	1546.7265	0.00475
1548.65512	0.00677	1548.6551	0.00498
1550.58371	0.00724	1550.5837	0.00524
1552.5123	0.00776	1552.5123	0.00553
1554.44088	0.00832	1554.4409	0.00586
1556.36947	0.00892	1556.3695	0.00624
1558.29806	0.00956	1558.2981	0.00666
1560.22664	0.01025	1560.2266	0.00713
1562.15523	0.01098	1562.1552	0.00765
1564.08382	0.01176	1564.0838	0.00822
1566.0124	0.01257	1566.0124	0.00884
1567.94099	0.01342	1567.941	0.0095
1569.86958	0.0143	1569.8696	0.01022
1571.79816	0.01521	1571.7982	0.01098
1573.72675	0.01615	1573.7268	0.01178
1575.65534	0.01711	1575.6553	0.01261
1577.58392	0.01808	1577.5839	0.01349
1579.51251	0.01907	1579.5125	0.01439
1581.4411	0.02005	1581.4411	0.01531
1583.36968	0.02104	1583.3697	0.01626
1585.29827	0.02202	1585.2983	0.01721
1587.22686	0.02298	1587.2269	0.01817
1589.15544	0.02393	1589.1554	0.01913
1591.08403	0.02484	1591.084	0.02008
1593.01262	0.02572	1593.0126	0.02101
1594.9412	0.02656	1594.9412	0.02193
1596.86979	0.02736	1596.8698	0.02281
1598.79838	0.0281	1598.7984	0.02366
1600.72696	0.02879	1600.727	0.02448
1602.65555	0.02941	1602.6556	0.02524
1604.58414	0.02997	1604.5841	0.02596
1606.51272	0.03047	1606.5127	0.02663
1608.44131	0.0309	1608.4413	0.02724
1610.3699	0.03125	1610.3699	0.02779
1612.29848	0.03153	1612.2985	0.02827
1614.22707	0.03174	1614.2271	0.02869
1616.15566	0.03188	1616.1557	0.02905
1618.08424	0.03195	1618.0842	0.02934
1620.01283	0.03195	1620.0128	0.02957
1621.94142	0.03188	1621.9414	0.02973
1623.87	0.03175	1623.87	0.02982
1625.79859	0.03155	1625.7986	0.02986
1627.72718	0.0313	1627.7272	0.02983
1629.65576	0.03099	1629.6558	0.02974
1631.58435	0.03063	1631.5844	0.02959
1633.51294	0.03022	1633.5129	0.02939
1635.44152	0.02976	1635.4415	0.02914

1637.37011	0.02927	1637.3701	0.02883
1639.2987	0.02874	1639.2987	0.02848
1641.22728	0.02818	1641.2273	0.02808
1643.15587	0.02759	1643.1559	0.02763
1645.08446	0.02697	1645.0845	0.02715
1647.01304	0.02633	1647.013	0.02662
1648.94163	0.02567	1648.9416	0.02606
1650.87022	0.02499	1650.8702	0.02546
1652.7988	0.0243	1652.7988	0.02483
1654.72739	0.02359	1654.7274	0.02417
1656.65598	0.02288	1656.656	0.02348
1658.58456	0.02215	1658.5846	0.02277
1660.51315	0.02142	1660.5132	0.02203
1662.44174	0.02068	1662.4417	0.02127
1664.37032	0.01995	1664.3703	0.02049
1666.29891	0.0192	1666.2989	0.0197
1668.2275	0.01846	1668.2275	0.0189
1670.15608	0.01772	1670.1561	0.01808
1672.08467	0.01698	1672.0847	0.01726
1674.01326	0.01625	1674.0133	0.01644
1675.94185	0.01552	1675.9419	0.01562
1677.87043	0.0148	1677.8704	0.0148
1679.79902	0.01408	1679.799	0.01398
1681.72761	0.01338	1681.7276	0.01318
1683.65619	0.01269	1683.6562	0.01238
1685.58478	0.012	1685.5848	0.0116
1687.51337	0.01134	1687.5134	0.01084
1689.44195	0.01068	1689.442	0.0101
1691.37054	0.01005	1691.3705	0.00938
1693.29913	0.00943	1693.2991	0.00869
1695.22771	0.00882	1695.2277	0.00802
1697.1563	0.00824	1697.1563	0.00738
1699.08489	0.00768	1699.0849	0.00678
1701.01347	0.00714	1701.0135	0.0062
1702.94206	0.00662	1702.9421	0.00565
1704.87065	0.00613	1704.8707	0.00513
1706.79923	0.00566	1706.7992	0.00465
1708.72782	0.00521	1708.7278	0.0042
1710.65641	0.00478	1710.6564	0.00378
1712.58499	0.00438	1712.585	0.00339
1714.51358	0.004	1714.5136	0.00303
1716.44217	0.00365	1716.4422	0.0027
1718.37075	0.00331	1718.3708	0.00239
1720.29934	0.003	1720.2993	0.00212
1722.22793	0.00271	1722.2279	0.00187
1724.15651	0.00245	1724.1565	0.00164
1726.0851	0.0022	1726.0851	0.00144
1728.01369	0.00197	1728.0137	0.00125
1729.94227	0.00176	1729.9423	0.00109
1731.87086	0.00157	1731.8709	0.00094383
1733.79945	0.0014	1733.7995	0.0008149
1735.72803	0.00124	1735.728	0.00070112
1737.65662	0.0011	1737.6566	0.0006011
1739.58521	0.00096689	1739.5852	0.00051354
1741.51379	0.00085024	1741.5138	0.00043718
1743.44238	0.00074556	1743.4424	0.00037086

1745.37097	0.00065194	1745.371	0.00031349
1747.29955	0.00056846	1747.2996	0.00026406
1749.22814	0.00049427	1749.2281	0.00022163
1751.15673	0.00042856	1751.1567	0.00018536
1753.08531	0.00037052	1753.0853	0.00015447
1755.0139	0.00031944	1755.0139	0.00012827
1756.94249	0.00027462	1756.9425	0.00010614
1758.87107	0.00023542	1758.8711	8.7511E-05
1760.79966	0.00020124	1760.7997	7.1895E-05
1762.72825	0.00017153	1762.7283	5.8856E-05
1764.65683	0.00014579	1764.6568	4.8009E-05
1766.58542	0.00012356	1766.5854	3.9021E-05
1768.51401	0.00010442	1768.514	3.1603E-05
1770.44259	8.7994E-05	1770.4426	2.5503E-05
1772.37118	7.3941E-05	1772.3712	2.0507E-05
1774.29977	6.1954E-05	1774.2998	1.6431E-05
1776.22835	5.1762E-05	1776.2284	1.3118E-05
1778.15694	4.3123E-05	1778.1569	1.0436E-05
1780.08553	3.5823E-05	1780.0855	8.2718E-06
1782.01411	2.9674E-05	1782.0141	6.5333E-06
1783.9427	0.00002451	1783.9427	5.1416E-06
1785.87129	2.0187E-05	1785.8713	4.032E-06
1787.79987	1.6578E-05	1787.7999	3.1504E-06
1789.72846	1.3576E-05	1789.7285	2.4528E-06
1791.65705	1.1086E-05	1791.6571	1.9029E-06
1793.58563	9.0262E-06	1793.5856	1.4709E-06
1795.51422	7.3283E-06	1795.5142	1.1329E-06
1797.44281	5.9327E-06	1797.4428	8.6949E-07
1799.37139	4.7891E-06	1799.3714	6.6492E-07

Fitting results of IR spectra of water-immersed brown coal with inhibitor

3000-3600

X Observed	Y Generated Coal	X Observed	Y Generated Coal+MgCl2	X Observed	Y Generated Coal+TEMPO
3000.8809	0.00071962	3000.8809	0.000944944	3000.8809	0.000223771
3002.80949	0.00077144	3002.80949	0.00102	3002.80949	0.000249823
3004.73807	0.00082582	3004.73807	0.00109	3004.73807	0.000278293
3006.66666	0.0008828	3006.66666	0.00117	3006.66666	0.000309325
3008.59525	0.00094238	3008.59525	0.00125	3008.59525	0.000343061
3010.52383	0.001	3010.52383	0.00134	3010.52383	0.000379639
3012.45242	0.00107	3012.45242	0.00143	3012.45242	0.000419194
3014.38101	0.00114	3014.38101	0.00153	3014.38101	0.000461855
3016.30959	0.00121	3016.30959	0.00163	3016.30959	0.000507742
3018.23818	0.00128	3018.23818	0.00173	3018.23818	0.000556966
3020.16677	0.00135	3020.16677	0.00184	3020.16677	0.000609627
3022.09535	0.00143	3022.09535	0.00195	3022.09535	0.00066581
3024.02394	0.00151	3024.02394	0.00207	3024.02394	0.000725586
3025.95253	0.00159	3025.95253	0.00218	3025.95253	0.00078901
3027.88111	0.00168	3027.88111	0.00231	3027.88111	0.000856115
3029.8097	0.00176	3029.8097	0.00243	3029.8097	0.000926915
3031.73829	0.00185	3031.73829	0.00256	3031.73829	0.001
3033.66687	0.00194	3033.66687	0.0027	3033.66687	0.00108
3035.59546	0.00203	3035.59546	0.00283	3035.59546	0.00116
3037.52405	0.00213	3037.52405	0.00297	3037.52405	0.00125
3039.45263	0.00222	3039.45263	0.00311	3039.45263	0.00134
3041.38122	0.00232	3041.38122	0.00325	3041.38122	0.00143
3043.30981	0.00241	3043.30981	0.0034	3043.30981	0.00152
3045.23839	0.00251	3045.23839	0.00355	3045.23839	0.00162
3047.16698	0.00261	3047.16698	0.0037	3047.16698	0.00172
3049.09557	0.00271	3049.09557	0.00385	3049.09557	0.00182
3051.02415	0.0028	3051.02415	0.004	3051.02415	0.00193
3052.95274	0.0029	3052.95274	0.00415	3052.95274	0.00204
3054.88133	0.003	3054.88133	0.00431	3054.88133	0.00215
3056.80991	0.0031	3056.80991	0.00446	3056.80991	0.00226
3058.7385	0.0032	3058.7385	0.00462	3058.7385	0.00237
3060.66709	0.0033	3060.66709	0.00477	3060.66709	0.00248
3062.59567	0.00339	3062.59567	0.00492	3062.59567	0.00259
3064.52426	0.00349	3064.52426	0.00508	3064.52426	0.0027
3066.45285	0.00358	3066.45285	0.00523	3066.45285	0.00281
3068.38143	0.00368	3068.38143	0.00539	3068.38143	0.00292
3070.31002	0.00377	3070.31002	0.00554	3070.31002	0.00303
3072.23861	0.00386	3072.23861	0.00569	3072.23861	0.00314
3074.16719	0.00395	3074.16719	0.00584	3074.16719	0.00325
3076.09578	0.00404	3076.09578	0.00599	3076.09578	0.00335
3078.02437	0.00413	3078.02437	0.00614	3078.02437	0.00345
3079.95295	0.00421	3079.95295	0.0063	3079.95295	0.00355
3081.88154	0.0043	3081.88154	0.00645	3081.88154	0.00365
3083.81013	0.00438	3083.81013	0.0066	3083.81013	0.00374
3085.73871	0.00447	3085.73871	0.00675	3085.73871	0.00384
3087.6673	0.00455	3087.6673	0.0069	3087.6673	0.00392
3089.59589	0.00463	3089.59589	0.00705	3089.59589	0.00401
3091.52447	0.00472	3091.52447	0.0072	3091.52447	0.0041
3093.45306	0.0048	3093.45306	0.00736	3093.45306	0.00418
3095.38165	0.00488	3095.38165	0.00752	3095.38165	0.00426

3097.31024	0.00496	3097.31024	0.00768	3097.31024	0.00433
3099.23882	0.00505	3099.23882	0.00785	3099.23882	0.00441
3101.16741	0.00514	3101.16741	0.00802	3101.16741	0.00449
3103.096	0.00522	3103.096	0.00819	3103.096	0.00456
3105.02458	0.00532	3105.02458	0.00837	3105.02458	0.00464
3106.95317	0.00541	3106.95317	0.00856	3106.95317	0.00471
3108.88176	0.00551	3108.88176	0.00875	3108.88176	0.00479
3110.81034	0.00561	3110.81034	0.00896	3110.81034	0.00487
3112.73893	0.00571	3112.73893	0.00917	3112.73893	0.00495
3114.66752	0.00582	3114.66752	0.00939	3114.66752	0.00504
3116.5961	0.00593	3116.5961	0.00962	3116.5961	0.00513
3118.52469	0.00605	3118.52469	0.00986	3118.52469	0.00522
3120.45328	0.00618	3120.45328	0.01012	3120.45328	0.00532
3122.38186	0.00631	3122.38186	0.01039	3122.38186	0.00543
3124.31045	0.00645	3124.31045	0.01067	3124.31045	0.00555
3126.23904	0.0066	3126.23904	0.01096	3126.23904	0.00567
3128.16762	0.00675	3128.16762	0.01127	3128.16762	0.0058
3130.09621	0.00692	3130.09621	0.01159	3130.09621	0.00594
3132.0248	0.00709	3132.0248	0.01193	3132.0248	0.00609
3133.95338	0.00727	3133.95338	0.01228	3133.95338	0.00625
3135.88197	0.00745	3135.88197	0.01265	3135.88197	0.00642
3137.81056	0.00765	3137.81056	0.01303	3137.81056	0.0066
3139.73914	0.00785	3139.73914	0.01343	3139.73914	0.0068
3141.66773	0.00807	3141.66773	0.01385	3141.66773	0.007
3143.59632	0.00829	3143.59632	0.01428	3143.59632	0.00722
3145.5249	0.00852	3145.5249	0.01473	3145.5249	0.00745
3147.45349	0.00876	3147.45349	0.01519	3147.45349	0.00769
3149.38208	0.00901	3149.38208	0.01566	3149.38208	0.00794
3151.31066	0.00927	3151.31066	0.01615	3151.31066	0.0082
3153.23925	0.00953	3153.23925	0.01664	3153.23925	0.00847
3155.16784	0.00981	3155.16784	0.01716	3155.16784	0.00875
3157.09642	0.01008	3157.09642	0.01768	3157.09642	0.00904
3159.02501	0.01037	3159.02501	0.01821	3159.02501	0.00934
3160.9536	0.01066	3160.9536	0.01875	3160.9536	0.00965
3162.88218	0.01096	3162.88218	0.01929	3162.88218	0.00997
3164.81077	0.01126	3164.81077	0.01984	3164.81077	0.01029
3166.73936	0.01156	3166.73936	0.0204	3166.73936	0.01061
3168.66794	0.01187	3168.66794	0.02095	3168.66794	0.01094
3170.59653	0.01218	3170.59653	0.02151	3170.59653	0.01128
3172.52512	0.01249	3172.52512	0.02207	3172.52512	0.01161
3174.4537	0.01281	3174.4537	0.02263	3174.4537	0.01195
3176.38229	0.01312	3176.38229	0.02318	3176.38229	0.01228
3178.31088	0.01343	3178.31088	0.02373	3178.31088	0.01262
3180.23946	0.01374	3180.23946	0.02428	3180.23946	0.01295
3182.16805	0.01405	3182.16805	0.02481	3182.16805	0.01327
3184.09664	0.01436	3184.09664	0.02534	3184.09664	0.01359
3186.02522	0.01466	3186.02522	0.02586	3186.02522	0.01391
3187.95381	0.01496	3187.95381	0.02637	3187.95381	0.01422
3189.8824	0.01526	3189.8824	0.02686	3189.8824	0.01452
3191.81098	0.01555	3191.81098	0.02735	3191.81098	0.01481
3193.73957	0.01583	3193.73957	0.02782	3193.73957	0.01509
3195.66816	0.01611	3195.66816	0.02827	3195.66816	0.01537
3197.59674	0.01638	3197.59674	0.02872	3197.59674	0.01563
3199.52533	0.01665	3199.52533	0.02915	3199.52533	0.01588
3201.45392	0.01691	3201.45392	0.02956	3201.45392	0.01613
3203.3825	0.01716	3203.3825	0.02996	3203.3825	0.01636

3205.31109	0.0174	3205.31109	0.03035	3205.31109	0.01658
3207.23968	0.01764	3207.23968	0.03073	3207.23968	0.01679
3209.16826	0.01788	3209.16826	0.03109	3209.16826	0.01699
3211.09685	0.0181	3211.09685	0.03144	3211.09685	0.01718
3213.02544	0.01832	3213.02544	0.03179	3213.02544	0.01736
3214.95402	0.01854	3214.95402	0.03212	3214.95402	0.01754
3216.88261	0.01875	3216.88261	0.03245	3216.88261	0.01771
3218.8112	0.01895	3218.8112	0.03277	3218.8112	0.01787
3220.73978	0.01915	3220.73978	0.03308	3220.73978	0.01802
3222.66837	0.01935	3222.66837	0.0334	3222.66837	0.01817
3224.59696	0.01955	3224.59696	0.03371	3224.59696	0.01832
3226.52554	0.01974	3226.52554	0.03402	3226.52554	0.01847
3228.45413	0.01993	3228.45413	0.03434	3228.45413	0.01862
3230.38272	0.02012	3230.38272	0.03467	3230.38272	0.01877
3232.3113	0.02031	3232.3113	0.035	3232.3113	0.01892
3234.23989	0.02051	3234.23989	0.03534	3234.23989	0.01907
3236.16848	0.0207	3236.16848	0.03569	3236.16848	0.01923
3238.09706	0.0209	3238.09706	0.03605	3238.09706	0.0194
3240.02565	0.0211	3240.02565	0.03643	3240.02565	0.01958
3241.95424	0.02131	3241.95424	0.03682	3241.95424	0.01976
3243.88282	0.02152	3243.88282	0.03723	3243.88282	0.01996
3245.81141	0.02174	3245.81141	0.03767	3245.81141	0.02017
3247.74	0.02196	3247.74	0.03812	3247.74	0.02039
3249.66858	0.02219	3249.66858	0.0386	3249.66858	0.02063
3251.59717	0.02244	3251.59717	0.03909	3251.59717	0.02088
3253.52576	0.02268	3253.52576	0.03962	3253.52576	0.02114
3255.45434	0.02294	3255.45434	0.04016	3255.45434	0.02143
3257.38293	0.02321	3257.38293	0.04074	3257.38293	0.02173
3259.31152	0.02349	3259.31152	0.04133	3259.31152	0.02204
3261.2401	0.02378	3261.2401	0.04196	3261.2401	0.02238
3263.16869	0.02408	3263.16869	0.04261	3263.16869	0.02273
3265.09728	0.02439	3265.09728	0.04328	3265.09728	0.02311
3267.02586	0.02472	3267.02586	0.04398	3267.02586	0.0235
3268.95445	0.02505	3268.95445	0.04471	3268.95445	0.02391
3270.88304	0.0254	3270.88304	0.04546	3270.88304	0.02433
3272.81162	0.02576	3272.81162	0.04623	3272.81162	0.02478
3274.74021	0.02613	3274.74021	0.04702	3274.74021	0.02524
3276.6688	0.02651	3276.6688	0.04784	3276.6688	0.02572
3278.59738	0.02691	3278.59738	0.04867	3278.59738	0.02622
3280.52597	0.02732	3280.52597	0.04953	3280.52597	0.02673
3282.45456	0.02774	3282.45456	0.0504	3282.45456	0.02725
3284.38314	0.02818	3284.38314	0.05129	3284.38314	0.0278
3286.31173	0.02863	3286.31173	0.05219	3286.31173	0.02835
3288.24032	0.02909	3288.24032	0.05311	3288.24032	0.02892
3290.16891	0.02956	3290.16891	0.05405	3290.16891	0.0295
3292.09749	0.03004	3292.09749	0.05499	3292.09749	0.03009
3294.02608	0.03054	3294.02608	0.05595	3294.02608	0.03069
3295.95467	0.03105	3295.95467	0.05692	3295.95467	0.0313
3297.88325	0.03158	3297.88325	0.05789	3297.88325	0.03192
3299.81184	0.03212	3299.81184	0.05888	3299.81184	0.03256
3301.74043	0.03267	3301.74043	0.05988	3301.74043	0.03319
3303.66901	0.03323	3303.66901	0.06089	3303.66901	0.03384
3305.5976	0.03381	3305.5976	0.0619	3305.5976	0.0345
3307.52619	0.03441	3307.52619	0.06292	3307.52619	0.03516
3309.45477	0.03502	3309.45477	0.06396	3309.45477	0.03583
3311.38336	0.03564	3311.38336	0.065	3311.38336	0.0365

3313.31195	0.03628	3313.31195	0.06605	3313.31195	0.03719
3315.24053	0.03693	3315.24053	0.06712	3315.24053	0.03788
3317.16912	0.0376	3317.16912	0.06819	3317.16912	0.03857
3319.09771	0.03829	3319.09771	0.06928	3319.09771	0.03928
3321.02629	0.03899	3321.02629	0.07038	3321.02629	0.03999
3322.95488	0.03971	3322.95488	0.0715	3322.95488	0.04071
3324.88347	0.04044	3324.88347	0.07263	3324.88347	0.04143
3326.81205	0.0412	3326.81205	0.07378	3326.81205	0.04217
3328.74064	0.04197	3328.74064	0.07494	3328.74064	0.04291
3330.66923	0.04276	3330.66923	0.07613	3330.66923	0.04366
3332.59781	0.04357	3332.59781	0.07733	3332.59781	0.04442
3334.5264	0.04439	3334.5264	0.07856	3334.5264	0.04519
3336.45499	0.04523	3336.45499	0.0798	3336.45499	0.04596
3338.38357	0.04609	3338.38357	0.08107	3338.38357	0.04675
3340.31216	0.04697	3340.31216	0.08236	3340.31216	0.04755
3342.24075	0.04787	3342.24075	0.08367	3342.24075	0.04836
3344.16933	0.04878	3344.16933	0.085	3344.16933	0.04918
3346.09792	0.04971	3346.09792	0.08636	3346.09792	0.05001
3348.02651	0.05065	3348.02651	0.08773	3348.02651	0.05085
3349.95509	0.05161	3349.95509	0.08913	3349.95509	0.0517
3351.88368	0.05258	3351.88368	0.09055	3351.88368	0.05256
3353.81227	0.05356	3353.81227	0.09198	3353.81227	0.05343
3355.74085	0.05456	3355.74085	0.09343	3355.74085	0.05431
3357.66944	0.05557	3357.66944	0.0949	3357.66944	0.05519
3359.59803	0.05658	3359.59803	0.09638	3359.59803	0.05609
3361.52661	0.0576	3361.52661	0.09786	3361.52661	0.05699
3363.4552	0.05863	3363.4552	0.09936	3363.4552	0.0579
3365.38379	0.05967	3365.38379	0.10086	3365.38379	0.05882
3367.31237	0.0607	3367.31237	0.10235	3367.31237	0.05974
3369.24096	0.06173	3369.24096	0.10385	3369.24096	0.06066
3371.16955	0.06277	3371.16955	0.10533	3371.16955	0.06159
3373.09813	0.0638	3373.09813	0.10681	3373.09813	0.06251
3375.02672	0.06482	3375.02672	0.10826	3375.02672	0.06344
3376.95531	0.06583	3376.95531	0.1097	3376.95531	0.06436
3378.88389	0.06683	3378.88389	0.11111	3378.88389	0.06527
3380.81248	0.06782	3380.81248	0.11249	3380.81248	0.06618
3382.74107	0.0688	3382.74107	0.11384	3382.74107	0.06708
3384.66965	0.06975	3384.66965	0.11515	3384.66965	0.06798
3386.59824	0.07069	3386.59824	0.11641	3386.59824	0.06886
3388.52683	0.0716	3388.52683	0.11763	3388.52683	0.06972
3390.45541	0.0725	3390.45541	0.1188	3390.45541	0.07057
3392.384	0.07336	3392.384	0.1199	3392.384	0.0714
3394.31259	0.07419	3394.31259	0.12095	3394.31259	0.07222
3396.24117	0.075	3396.24117	0.12194	3396.24117	0.07301
3398.16976	0.07577	3398.16976	0.12285	3398.16976	0.07378
3400.09835	0.07651	3400.09835	0.1237	3400.09835	0.07452
3402.02693	0.07721	3402.02693	0.12447	3402.02693	0.07524
3403.95552	0.07788	3403.95552	0.12517	3403.95552	0.07593
3405.88411	0.07851	3405.88411	0.12579	3405.88411	0.07659
3407.81269	0.07909	3407.81269	0.12633	3407.81269	0.07722
3409.74128	0.07964	3409.74128	0.12679	3409.74128	0.07781
3411.66987	0.08015	3411.66987	0.12717	3411.66987	0.07838
3413.59845	0.08062	3413.59845	0.12747	3413.59845	0.07891
3415.52704	0.08104	3415.52704	0.12769	3415.52704	0.0794
3417.45563	0.08142	3417.45563	0.12782	3417.45563	0.07986
3419.38421	0.08176	3419.38421	0.12787	3419.38421	0.08028

3421.3128	0.08205	3421.3128	0.12785	3421.3128	0.08067
3423.24139	0.08231	3423.24139	0.12774	3423.24139	0.08101
3425.16997	0.08252	3425.16997	0.12756	3425.16997	0.08132
3427.09856	0.08269	3427.09856	0.12731	3427.09856	0.0816
3429.02715	0.08282	3429.02715	0.12698	3429.02715	0.08183
3430.95573	0.08291	3430.95573	0.12659	3430.95573	0.08203
3432.88432	0.08296	3432.88432	0.12613	3432.88432	0.08219
3434.81291	0.08298	3434.81291	0.12561	3434.81291	0.08231
3436.74149	0.08296	3436.74149	0.12503	3436.74149	0.0824
3438.67008	0.0829	3438.67008	0.1244	3438.67008	0.08245
3440.59867	0.08281	3440.59867	0.12371	3440.59867	0.08247
3442.52725	0.08268	3442.52725	0.12298	3442.52725	0.08245
3444.45584	0.08253	3444.45584	0.1222	3444.45584	0.0824
3446.38443	0.08234	3446.38443	0.12138	3446.38443	0.08232
3448.31301	0.08213	3448.31301	0.12053	3448.31301	0.08221
3450.2416	0.08189	3450.2416	0.11965	3450.2416	0.08206
3452.17019	0.08163	3452.17019	0.11874	3452.17019	0.08188
3454.09877	0.08134	3454.09877	0.1178	3454.09877	0.08168
3456.02736	0.08103	3456.02736	0.11684	3456.02736	0.08145
3457.95595	0.0807	3457.95595	0.11587	3457.95595	0.08119
3459.88453	0.08035	3459.88453	0.11488	3459.88453	0.08091
3461.81312	0.07998	3461.81312	0.11387	3461.81312	0.0806
3463.74171	0.0796	3463.74171	0.11286	3463.74171	0.08027
3465.67029	0.0792	3465.67029	0.11185	3465.67029	0.07992
3467.59888	0.07878	3467.59888	0.11082	3467.59888	0.07954
3469.52747	0.07835	3469.52747	0.1098	3469.52747	0.07915
3471.45605	0.07791	3471.45605	0.10877	3471.45605	0.07873
3473.38464	0.07746	3473.38464	0.10775	3473.38464	0.0783
3475.31323	0.077	3475.31323	0.10672	3475.31323	0.07785
3477.24181	0.07652	3477.24181	0.1057	3477.24181	0.07738
3479.1704	0.07604	3479.1704	0.10468	3479.1704	0.0769
3481.09899	0.07554	3481.09899	0.10367	3481.09899	0.07639
3483.02758	0.07504	3483.02758	0.10265	3483.02758	0.07588
3484.95616	0.07452	3484.95616	0.10164	3484.95616	0.07535
3486.88475	0.074	3486.88475	0.10064	3486.88475	0.0748
3488.81334	0.07347	3488.81334	0.09964	3488.81334	0.07424
3490.74192	0.07293	3490.74192	0.09863	3490.74192	0.07366
3492.67051	0.07237	3492.67051	0.09763	3492.67051	0.07308
3494.5991	0.07181	3494.5991	0.09663	3494.5991	0.07247
3496.52768	0.07124	3496.52768	0.09563	3496.52768	0.07186
3498.45627	0.07066	3498.45627	0.09463	3498.45627	0.07123
3500.38486	0.07007	3500.38486	0.09362	3500.38486	0.07059
3502.31344	0.06947	3502.31344	0.09261	3502.31344	0.06994
3504.24203	0.06886	3504.24203	0.09159	3504.24203	0.06927
3506.17062	0.06823	3506.17062	0.09056	3506.17062	0.06859
3508.0992	0.06759	3508.0992	0.08953	3508.0992	0.0679
3510.02779	0.06695	3510.02779	0.08848	3510.02779	0.06719
3511.95638	0.06628	3511.95638	0.08743	3511.95638	0.06647
3513.88496	0.06561	3513.88496	0.08636	3513.88496	0.06574
3515.81355	0.06492	3515.81355	0.08527	3515.81355	0.065
3517.74214	0.06421	3517.74214	0.08418	3517.74214	0.06424
3519.67072	0.06349	3519.67072	0.08306	3519.67072	0.06347
3521.59931	0.06276	3521.59931	0.08193	3521.59931	0.06269
3523.5279	0.06201	3523.5279	0.08078	3523.5279	0.0619
3525.45648	0.06125	3525.45648	0.07961	3525.45648	0.06109
3527.38507	0.06047	3527.38507	0.07843	3527.38507	0.06027

3529.31366	0.05968	3529.31366	0.07723	3529.31366	0.05943
3531.24224	0.05887	3531.24224	0.076	3531.24224	0.05859
3533.17083	0.05804	3533.17083	0.07476	3533.17083	0.05773
3535.09942	0.0572	3535.09942	0.0735	3535.09942	0.05686
3537.028	0.05635	3537.028	0.07222	3537.028	0.05598
3538.95659	0.05548	3538.95659	0.07092	3538.95659	0.05509
3540.88518	0.0546	3540.88518	0.06961	3540.88518	0.05419
3542.81376	0.0537	3542.81376	0.06827	3542.81376	0.05328
3544.74235	0.05279	3544.74235	0.06693	3544.74235	0.05236
3546.67094	0.05187	3546.67094	0.06556	3546.67094	0.05142
3548.59952	0.05093	3548.59952	0.06418	3548.59952	0.05048
3550.52811	0.04999	3550.52811	0.06279	3550.52811	0.04954
3552.4567	0.04903	3552.4567	0.06139	3552.4567	0.04858
3554.38528	0.04807	3554.38528	0.05998	3554.38528	0.04762
3556.31387	0.04709	3556.31387	0.05856	3556.31387	0.04665
3558.24246	0.04611	3558.24246	0.05713	3558.24246	0.04568
3560.17104	0.04512	3560.17104	0.0557	3560.17104	0.0447
3562.09963	0.04412	3562.09963	0.05426	3562.09963	0.04372
3564.02822	0.04312	3564.02822	0.05282	3564.02822	0.04273
3565.9568	0.04211	3565.9568	0.05138	3565.9568	0.04175
3567.88539	0.0411	3567.88539	0.04994	3567.88539	0.04076
3569.81398	0.04009	3569.81398	0.0485	3569.81398	0.03977
3571.74256	0.03908	3571.74256	0.04707	3571.74256	0.03879
3573.67115	0.03807	3573.67115	0.04564	3573.67115	0.0378
3575.59974	0.03706	3575.59974	0.04423	3575.59974	0.03682
3577.52832	0.03605	3577.52832	0.04282	3577.52832	0.03584
3579.45691	0.03505	3579.45691	0.04142	3579.45691	0.03486
3581.3855	0.03405	3581.3855	0.04004	3581.3855	0.03389
3583.31408	0.03306	3583.31408	0.03867	3583.31408	0.03293
3585.24267	0.03207	3585.24267	0.03732	3585.24267	0.03197
3587.17126	0.03109	3587.17126	0.03599	3587.17126	0.03103
3589.09984	0.03012	3589.09984	0.03467	3589.09984	0.03009
3591.02843	0.02916	3591.02843	0.03338	3591.02843	0.02915
3592.95702	0.02821	3592.95702	0.0321	3592.95702	0.02823
3594.8856	0.02727	3594.8856	0.03085	3594.8856	0.02733
3596.81419	0.02635	3596.81419	0.02963	3596.81419	0.02643
3598.74278	0.02544	3598.74278	0.02842	3598.74278	0.02554
1600.72696	0.0317	1600.72696	0.03143	1600.72696	0.02778
1602.65555	0.03239	1602.65555	0.03242	1602.65555	0.02862
1604.58414	0.03302	1604.58414	0.03337	1604.58414	0.02939
1606.51272	0.0336	1606.51272	0.03426	1606.51272	0.0301
1608.44131	0.03413	1608.44131	0.03508	1608.44131	0.03073
1610.3699	0.03459	1610.3699	0.03584	1610.3699	0.03128
1612.29848	0.035	1612.29848	0.03654	1612.29848	0.03175
1614.22707	0.03535	1614.22707	0.03715	1614.22707	0.03213
1616.15566	0.03563	1616.15566	0.03769	1616.15566	0.03242
1618.08424	0.03585	1618.08424	0.03815	1618.08424	0.03263
1620.01283	0.03601	1620.01283	0.03853	1620.01283	0.03274
1621.94142	0.0361	1621.94142	0.03882	1621.94142	0.03277
1623.87	0.03613	1623.87	0.03903	1623.87	0.03271
1625.79859	0.0361	1625.79859	0.03916	1625.79859	0.03257
1627.72718	0.03599	1627.72718	0.0392	1627.72718	0.03235
1629.65576	0.03583	1629.65576	0.03916	1629.65576	0.03205
1631.58435	0.0356	1631.58435	0.03904	1631.58435	0.03168
1633.51294	0.03532	1633.51294	0.03883	1633.51294	0.03124
1635.44152	0.03497	1635.44152	0.03855	1635.44152	0.03075

1637.37011	0.03457	1637.37011	0.03819	1637.37011	0.03019
1639.2987	0.03411	1639.2987	0.03776	1639.2987	0.02959
1641.22728	0.03359	1641.22728	0.03725	1641.22728	0.02894
1643.15587	0.03303	1643.15587	0.03668	1643.15587	0.02826
1645.08446	0.03241	1645.08446	0.03605	1645.08446	0.02754
1647.01304	0.03175	1647.01304	0.03536	1647.01304	0.0268
1648.94163	0.03105	1648.94163	0.0346	1648.94163	0.02603
1650.87022	0.0303	1650.87022	0.0338	1650.87022	0.02525
1652.7988	0.02952	1652.7988	0.03295	1652.7988	0.02446
1654.72739	0.0287	1654.72739	0.03206	1654.72739	0.02365
1656.65598	0.02786	1656.65598	0.03112	1656.65598	0.02285
1658.58456	0.02698	1658.58456	0.03015	1658.58456	0.02204
1660.51315	0.02609	1660.51315	0.02915	1660.51315	0.02124
1662.44174	0.02517	1662.44174	0.02813	1662.44174	0.02044
1664.37032	0.02423	1664.37032	0.02708	1664.37032	0.01965
1666.29891	0.02328	1666.29891	0.02602	1666.29891	0.01887
1668.2275	0.02233	1668.2275	0.02494	1668.2275	0.01809
1670.15608	0.02136	1670.15608	0.02385	1670.15608	0.01734
1672.08467	0.0204	1672.08467	0.02276	1672.08467	0.01659
1674.01326	0.01944	1674.01326	0.02167	1674.01326	0.01586
1675.94185	0.01848	1675.94185	0.02059	1675.94185	0.01514
1677.87043	0.01753	1677.87043	0.01951	1677.87043	0.01445
1679.79902	0.01659	1679.79902	0.01845	1679.79902	0.01376
1681.72761	0.01566	1681.72761	0.0174	1681.72761	0.01309
1683.65619	0.01476	1683.65619	0.01637	1683.65619	0.01244
1685.58478	0.01387	1685.58478	0.01536	1685.58478	0.01181
1687.51337	0.013	1687.51337	0.01438	1687.51337	0.01119
1689.44195	0.01216	1689.44195	0.01343	1689.44195	0.0106
1691.37054	0.01135	1691.37054	0.01251	1691.37054	0.01001
1693.29913	0.01056	1693.29913	0.01162	1693.29913	0.00945
1695.22771	0.00981	1695.22771	0.01076	1695.22771	0.0089
1697.1563	0.00909	1697.1563	0.00994	1697.1563	0.00838
1699.08489	0.00839	1699.08489	0.00916	1699.08489	0.00787
1701.01347	0.00773	1701.01347	0.00842	1701.01347	0.00738
1702.94206	0.00711	1702.94206	0.00771	1702.94206	0.0069
1704.87065	0.00652	1704.87065	0.00705	1704.87065	0.00645
1706.79923	0.00596	1706.79923	0.00642	1706.79923	0.00601
1708.72782	0.00543	1708.72782	0.00584	1708.72782	0.0056
1710.65641	0.00494	1710.65641	0.00529	1710.65641	0.0052
1712.58499	0.00448	1712.58499	0.00478	1712.58499	0.00482
1714.51358	0.00405	1714.51358	0.0043	1714.51358	0.00446
1716.44217	0.00366	1716.44217	0.00386	1716.44217	0.00412
1718.37075	0.00329	1718.37075	0.00346	1718.37075	0.00379
1720.29934	0.00295	1720.29934	0.00309	1720.29934	0.00349
1722.22793	0.00264	1722.22793	0.00275	1722.22793	0.0032
1724.15651	0.00236	1724.15651	0.00244	1724.15651	0.00293
1726.0851	0.0021	1726.0851	0.00216	1726.0851	0.00267
1728.01369	0.00186	1728.01369	0.0019	1728.01369	0.00243
1729.94227	0.00164	1729.94227	0.00167	1729.94227	0.00221
1731.87086	0.00145	1731.87086	0.00147	1731.87086	0.00201
1733.79945	0.00128	1733.79945	0.00128	1733.79945	0.00182
1735.72803	0.00112	1735.72803	0.00112	1735.72803	0.00164
1737.65662	0.00097895	1737.65662	0.000969225	1737.65662	0.00148
1739.58521	0.00085373	1739.58521	0.000839069	1739.58521	0.00133
1741.51379	0.00074237	1741.51379	0.00072412	1741.51379	0.00119
1743.44238	0.00064367	1743.44238	0.000622957	1743.44238	0.00106

1745.37097	0.00055648	1745.37097	0.000534242	1745.37097	0.00094964
1747.29955	0.00047969	1747.29955	0.000456715	1747.29955	0.000845286
1749.22814	0.00041229	1749.22814	0.000389205	1749.22814	0.000750599
1751.15673	0.00035332	1751.15673	0.000330623	1751.15673	0.000664919
1753.08531	0.0003019	1753.08531	0.000279968	1753.08531	0.000587602
1755.0139	0.0002572	1755.0139	0.000236319	1755.0139	0.000518024
1756.94249	0.00021848	1756.94249	0.000198841	1756.94249	0.000455581
1758.87107	0.00018503	1758.87107	0.000166772	1758.87107	0.000399696
1760.79966	0.00015625	1760.79966	0.000139428	1760.79966	0.000349816
1762.72825	0.00013155	1762.72825	0.000116195	1762.72825	0.000305418
1764.65683	0.00011042	1764.65683	9.65227E-05	1764.65683	0.000266008
1766.58542	9.2415E-05	1766.58542	7.99239E-05	1766.58542	0.000231119
1768.51401	7.7114E-05	1768.51401	6.59671E-05	1768.51401	0.000200318
1770.44259	6.4155E-05	1770.44259	5.42724E-05	1770.44259	0.000173198
1772.37118	5.3213E-05	1772.37118	4.45073E-05	1772.37118	0.000149385
1774.29977	4.4006E-05	1774.29977	3.63817E-05	1774.29977	0.000128532
1776.22835	3.6283E-05	1776.22835	2.96437E-05	1776.22835	0.00011032
1778.15694	2.9826E-05	1778.15694	2.40757E-05	1778.15694	9.44576E-05
1780.08553	2.4444E-05	1780.08553	1.94904E-05	1780.08553	8.06783E-05
1782.01411	1.9973E-05	1782.01411	1.57274E-05	1782.01411	6.87408E-05
1783.9427	1.6271E-05	1783.9427	1.26499E-05	1783.9427	5.84265E-05
1785.87129	1.3215E-05	1785.87129	1.01417E-05	1785.87129	4.95385E-05
1787.79987	1.0701E-05	1787.79987	8.10455E-06	1787.79987	4.18999E-05
1789.72846	8.6391E-06	1789.72846	6.45561E-06	1789.72846	3.53524E-05
1791.65705	6.9533E-06	1791.65705	5.1255E-06	1791.65705	2.97552E-05
1793.58563	5.5796E-06	1793.58563	4.05627E-06	1793.58563	2.49829E-05
1795.51422	4.4638E-06	1795.51422	3.19968E-06	1795.51422	2.09247E-05
1797.44281	3.5603E-06	1797.44281	2.5158E-06	1797.44281	1.74828E-05
1799.37139	2.8311E-06	1799.37139	1.97167E-06	1799.37139	1.45714E-05

X Observed	Y Generated Coal+TPPI	X Observed	Y Generated Coal+PA
3000.8809	0.00074157	3000.8809	0.00033185
3002.80949	0.00078187	3002.8095	0.00036266
3004.73807	0.00082349	3004.7381	0.00039565
3006.66666	0.00086642	3006.6667	0.0004309
3008.59525	0.00091063	3008.5953	0.00046849
3010.52383	0.0009561	3010.5238	0.00050849
3012.45242	0.001	3012.4524	0.00055095
3014.38101	0.00105	3014.381	0.00059595
3016.30959	0.0011	3016.3096	0.00064352
3018.23818	0.00115	3018.2382	0.00069372
3020.16677	0.0012	3020.1668	0.00074657
3022.09535	0.00125	3022.0954	0.00080209
3024.02394	0.00131	3024.0239	0.0008603
3025.95253	0.00136	3025.9525	0.00092119
3027.88111	0.00142	3027.8811	0.00098475
3029.8097	0.00147	3029.8097	0.00105
3031.73829	0.00153	3031.7383	0.00112
3033.66687	0.00158	3033.6669	0.00119
3035.59546	0.00164	3035.5955	0.00126
3037.52405	0.0017	3037.5241	0.00134
3039.45263	0.00176	3039.4526	0.00142
3041.38122	0.00182	3041.3812	0.0015
3043.30981	0.00188	3043.3098	0.00158
3045.23839	0.00194	3045.2384	0.00167
3047.16698	0.002	3047.167	0.00176
3049.09557	0.00206	3049.0956	0.00184
3051.02415	0.00212	3051.0242	0.00193
3052.95274	0.00218	3052.9527	0.00202
3054.88133	0.00224	3054.8813	0.00212
3056.80991	0.0023	3056.8099	0.00221
3058.7385	0.00236	3058.7385	0.00231
3060.66709	0.00242	3060.6671	0.0024
3062.59567	0.00248	3062.5957	0.0025
3064.52426	0.00254	3064.5243	0.00259
3066.45285	0.00259	3066.4529	0.00269
3068.38143	0.00265	3068.3814	0.00278
3070.31002	0.00271	3070.31	0.00288
3072.23861	0.00278	3072.2386	0.00298
3074.16719	0.00284	3074.1672	0.00307
3076.09578	0.0029	3076.0958	0.00317
3078.02437	0.00296	3078.0244	0.00326
3079.95295	0.00302	3079.953	0.00336
3081.88154	0.00308	3081.8815	0.00345
3083.81013	0.00315	3083.8101	0.00354
3085.73871	0.00321	3085.7387	0.00364
3087.6673	0.00328	3087.6673	0.00373
3089.59589	0.00335	3089.5959	0.00382
3091.52447	0.00342	3091.5245	0.00391
3093.45306	0.00349	3093.4531	0.00401
3095.38165	0.00357	3095.3817	0.0041

3097.31024	0.00365	3097.3102	0.00419
3099.23882	0.00373	3099.2388	0.00429
3101.16741	0.00382	3101.1674	0.00438
3103.096	0.0039	3103.096	0.00448
3105.02458	0.004	3105.0246	0.00457
3106.95317	0.00409	3106.9532	0.00467
3108.88176	0.0042	3108.8818	0.00477
3110.81034	0.0043	3110.8103	0.00488
3112.73893	0.00442	3112.7389	0.00498
3114.66752	0.00454	3114.6675	0.00509
3116.5961	0.00466	3116.5961	0.00521
3118.52469	0.00479	3118.5247	0.00532
3120.45328	0.00493	3120.4533	0.00545
3122.38186	0.00507	3122.3819	0.00557
3124.31045	0.00523	3124.3105	0.00571
3126.23904	0.00539	3126.239	0.00584
3128.16762	0.00555	3128.1676	0.00599
3130.09621	0.00573	3130.0962	0.00613
3132.0248	0.00591	3132.0248	0.00629
3133.95338	0.0061	3133.9534	0.00645
3135.88197	0.00629	3135.882	0.00662
3137.81056	0.0065	3137.8106	0.00679
3139.73914	0.00671	3139.7391	0.00698
3141.66773	0.00693	3141.6677	0.00716
3143.59632	0.00716	3143.5963	0.00736
3145.5249	0.00739	3145.5249	0.00756
3147.45349	0.00764	3147.4535	0.00777
3149.38208	0.00788	3149.3821	0.00798
3151.31066	0.00814	3151.3107	0.0082
3153.23925	0.0084	3153.2393	0.00843
3155.16784	0.00866	3155.1678	0.00866
3157.09642	0.00894	3157.0964	0.0089
3159.02501	0.00921	3159.025	0.00914
3160.9536	0.00949	3160.9536	0.00939
3162.88218	0.00977	3162.8822	0.00964
3164.81077	0.01005	3164.8108	0.00989
3166.73936	0.01034	3166.7394	0.01014
3168.66794	0.01063	3168.6679	0.0104
3170.59653	0.01091	3170.5965	0.01066
3172.52512	0.0112	3172.5251	0.01092
3174.4537	0.01149	3174.4537	0.01118
3176.38229	0.01177	3176.3823	0.01144
3178.31088	0.01206	3178.3109	0.0117
3180.23946	0.01234	3180.2395	0.01196
3182.16805	0.01262	3182.1681	0.01222
3184.09664	0.01289	3184.0966	0.01247
3186.02522	0.01316	3186.0252	0.01272
3187.95381	0.01342	3187.9538	0.01297
3189.8824	0.01368	3189.8824	0.01322
3191.81098	0.01393	3191.811	0.01346
3193.73957	0.01418	3193.7396	0.0137
3195.66816	0.01442	3195.6682	0.01393
3197.59674	0.01465	3197.5967	0.01416
3199.52533	0.01488	3199.5253	0.01439
3201.45392	0.0151	3201.4539	0.01461
3203.3825	0.01531	3203.3825	0.01483

3205.31109	0.01552	3205.3111	0.01504
3207.23968	0.01572	3207.2397	0.01525
3209.16826	0.01591	3209.1683	0.01546
3211.09685	0.0161	3211.0969	0.01567
3213.02544	0.01628	3213.0254	0.01587
3214.95402	0.01646	3214.954	0.01608
3216.88261	0.01663	3216.8826	0.01628
3218.8112	0.0168	3218.8112	0.01648
3220.73978	0.01697	3220.7398	0.01668
3222.66837	0.01713	3222.6684	0.01689
3224.59696	0.01729	3224.597	0.0171
3226.52554	0.01745	3226.5255	0.01731
3228.45413	0.0176	3228.4541	0.01752
3230.38272	0.01776	3230.3827	0.01774
3232.3113	0.01792	3232.3113	0.01797
3234.23989	0.01808	3234.2399	0.0182
3236.16848	0.01825	3236.1685	0.01844
3238.09706	0.01841	3238.0971	0.01868
3240.02565	0.01859	3240.0257	0.01894
3241.95424	0.01876	3241.9542	0.01921
3243.88282	0.01895	3243.8828	0.01948
3245.81141	0.01913	3245.8114	0.01977
3247.74	0.01933	3247.74	0.02007
3249.66858	0.01953	3249.6686	0.02038
3251.59717	0.01975	3251.5972	0.02071
3253.52576	0.01997	3253.5258	0.02104
3255.45434	0.0202	3255.4543	0.02139
3257.38293	0.02044	3257.3829	0.02176
3259.31152	0.0207	3259.3115	0.02213
3261.2401	0.02096	3261.2401	0.02252
3263.16869	0.02123	3263.1687	0.02293
3265.09728	0.02152	3265.0973	0.02334
3267.02586	0.02182	3267.0259	0.02377
3268.95445	0.02213	3268.9545	0.02421
3270.88304	0.02245	3270.883	0.02467
3272.81162	0.02279	3272.8116	0.02513
3274.74021	0.02314	3274.7402	0.02561
3276.6688	0.0235	3276.6688	0.0261
3278.59738	0.02388	3278.5974	0.0266
3280.52597	0.02426	3280.526	0.02711
3282.45456	0.02466	3282.4546	0.02763
3284.38314	0.02508	3284.3831	0.02815
3286.31173	0.02551	3286.3117	0.02869
3288.24032	0.02595	3288.2403	0.02923
3290.16891	0.0264	3290.1689	0.02978
3292.09749	0.02687	3292.0975	0.03033
3294.02608	0.02735	3294.0261	0.0309
3295.95467	0.02785	3295.9547	0.03146
3297.88325	0.02836	3297.8833	0.03204
3299.81184	0.02888	3299.8118	0.03262
3301.74043	0.02942	3301.7404	0.0332
3303.66901	0.02997	3303.669	0.03379
3305.5976	0.03054	3305.5976	0.03438
3307.52619	0.03112	3307.5262	0.03498
3309.45477	0.03172	3309.4548	0.03559
3311.38336	0.03234	3311.3834	0.0362

3313.31195	0.03297	3313.312	0.03681
3315.24053	0.03361	3315.2405	0.03743
3317.16912	0.03427	3317.1691	0.03806
3319.09771	0.03495	3319.0977	0.03869
3321.02629	0.03565	3321.0263	0.03933
3322.95488	0.03636	3322.9549	0.03998
3324.88347	0.03709	3324.8835	0.04064
3326.81205	0.03784	3326.8121	0.0413
3328.74064	0.03861	3328.7406	0.04198
3330.66923	0.03939	3330.6692	0.04266
3332.59781	0.0402	3332.5978	0.04336
3334.5264	0.04101	3334.5264	0.04407
3336.45499	0.04185	3336.455	0.04478
3338.38357	0.04271	3338.3836	0.04552
3340.31216	0.04358	3340.3122	0.04626
3342.24075	0.04446	3342.2408	0.04701
3344.16933	0.04537	3344.1693	0.04778
3346.09792	0.04628	3346.0979	0.04857
3348.02651	0.04721	3348.0265	0.04936
3349.95509	0.04816	3349.9551	0.05017
3351.88368	0.04912	3351.8837	0.05099
3353.81227	0.05008	3353.8123	0.05182
3355.74085	0.05106	3355.7409	0.05267
3357.66944	0.05205	3357.6694	0.05353
3359.59803	0.05304	3359.598	0.05439
3361.52661	0.05404	3361.5266	0.05527
3363.4552	0.05504	3363.4552	0.05615
3365.38379	0.05604	3365.3838	0.05704
3367.31237	0.05704	3367.3124	0.05794
3369.24096	0.05804	3369.241	0.05884
3371.16955	0.05904	3371.1696	0.05975
3373.09813	0.06002	3373.0981	0.06065
3375.02672	0.061	3375.0267	0.06156
3376.95531	0.06197	3376.9553	0.06246
3378.88389	0.06293	3378.8839	0.06335
3380.81248	0.06387	3380.8125	0.06424
3382.74107	0.06479	3382.7411	0.06512
3384.66965	0.06569	3384.6697	0.06599
3386.59824	0.06657	3386.5982	0.06685
3388.52683	0.06743	3388.5268	0.06769
3390.45541	0.06826	3390.4554	0.06851
3392.384	0.06907	3392.384	0.06931
3394.31259	0.06984	3394.3126	0.0701
3396.24117	0.07059	3396.2412	0.07085
3398.16976	0.0713	3398.1698	0.07159
3400.09835	0.07197	3400.0984	0.07229
3402.02693	0.07262	3402.0269	0.07297
3403.95552	0.07322	3403.9555	0.07362
3405.88411	0.07379	3405.8841	0.07423
3407.81269	0.07432	3407.8127	0.07481
3409.74128	0.07481	3409.7413	0.07536
3411.66987	0.07526	3411.6699	0.07587
3413.59845	0.07567	3413.5985	0.07634
3415.52704	0.07604	3415.527	0.07678
3417.45563	0.07637	3417.4556	0.07718
3419.38421	0.07666	3419.3842	0.07754

3421.3128	0.07692	3421.3128	0.07786
3423.24139	0.07713	3423.2414	0.07815
3425.16997	0.0773	3425.17	0.0784
3427.09856	0.07744	3427.0986	0.07861
3429.02715	0.07754	3429.0272	0.07878
3430.95573	0.07761	3430.9557	0.07891
3432.88432	0.07764	3432.8843	0.07901
3434.81291	0.07764	3434.8129	0.07908
3436.74149	0.0776	3436.7415	0.07911
3438.67008	0.07754	3438.6701	0.07911
3440.59867	0.07744	3440.5987	0.07907
3442.52725	0.07732	3442.5273	0.07901
3444.45584	0.07717	3444.4558	0.07891
3446.38443	0.077	3446.3844	0.07878
3448.31301	0.0768	3448.313	0.07863
3450.2416	0.07658	3450.2416	0.07846
3452.17019	0.07634	3452.1702	0.07825
3454.09877	0.07608	3454.0988	0.07803
3456.02736	0.0758	3456.0274	0.07778
3457.95595	0.07551	3457.956	0.07751
3459.88453	0.0752	3459.8845	0.07723
3461.81312	0.07487	3461.8131	0.07692
3463.74171	0.07453	3463.7417	0.0766
3465.67029	0.07418	3465.6703	0.07626
3467.59888	0.07381	3467.5989	0.07591
3469.52747	0.07344	3469.5275	0.07554
3471.45605	0.07305	3471.4561	0.07516
3473.38464	0.07265	3473.3846	0.07477
3475.31323	0.07224	3475.3132	0.07437
3477.24181	0.07183	3477.2418	0.07395
3479.1704	0.0714	3479.1704	0.07352
3481.09899	0.07096	3481.099	0.07309
3483.02758	0.07052	3483.0276	0.07264
3484.95616	0.07006	3484.9562	0.07218
3486.88475	0.0696	3486.8848	0.07172
3488.81334	0.06912	3488.8133	0.07124
3490.74192	0.06864	3490.7419	0.07075
3492.67051	0.06814	3492.6705	0.07026
3494.5991	0.06764	3494.5991	0.06975
3496.52768	0.06712	3496.5277	0.06923
3498.45627	0.06659	3498.4563	0.06871
3500.38486	0.06605	3500.3849	0.06817
3502.31344	0.0655	3502.3134	0.06762
3504.24203	0.06493	3504.242	0.06706
3506.17062	0.06435	3506.1706	0.06649
3508.0992	0.06375	3508.0992	0.0659
3510.02779	0.06314	3510.0278	0.0653
3511.95638	0.06251	3511.9564	0.06469
3513.88496	0.06187	3513.885	0.06407
3515.81355	0.06121	3515.8136	0.06343
3517.74214	0.06054	3517.7421	0.06278
3519.67072	0.05984	3519.6707	0.06211
3521.59931	0.05914	3521.5993	0.06143
3523.5279	0.05841	3523.5279	0.06073
3525.45648	0.05767	3525.4565	0.06002
3527.38507	0.05691	3527.3851	0.05929

3529.31366	0.05613	3529.3137	0.05855
3531.24224	0.05533	3531.2422	0.05779
3533.17083	0.05452	3533.1708	0.05702
3535.09942	0.05369	3535.0994	0.05623
3537.028	0.05285	3537.028	0.05542
3538.95659	0.05199	3538.9566	0.05461
3540.88518	0.05111	3540.8852	0.05377
3542.81376	0.05022	3542.8138	0.05293
3544.74235	0.04932	3544.7424	0.05206
3546.67094	0.0484	3546.6709	0.05119
3548.59952	0.04747	3548.5995	0.0503
3550.52811	0.04653	3550.5281	0.04941
3552.4567	0.04558	3552.4567	0.0485
3554.38528	0.04462	3554.3853	0.04758
3556.31387	0.04365	3556.3139	0.04665
3558.24246	0.04268	3558.2425	0.04571
3560.17104	0.04169	3560.171	0.04476
3562.09963	0.0407	3562.0996	0.04381
3564.02822	0.03971	3564.0282	0.04285
3565.9568	0.03872	3565.9568	0.04189
3567.88539	0.03772	3567.8854	0.04092
3569.81398	0.03672	3569.814	0.03995
3571.74256	0.03573	3571.7426	0.03897
3573.67115	0.03473	3573.6712	0.038
3575.59974	0.03374	3575.5997	0.03703
3577.52832	0.03275	3577.5283	0.03605
3579.45691	0.03177	3579.4569	0.03508
3581.3855	0.03079	3581.3855	0.03412
3583.31408	0.02982	3583.3141	0.03315
3585.24267	0.02886	3585.2427	0.0322
3587.17126	0.02791	3587.1713	0.03124
3589.09984	0.02697	3589.0998	0.0303
3591.02843	0.02605	3591.0284	0.02937
3592.95702	0.02513	3592.957	0.02844
3594.8856	0.02423	3594.8856	0.02752
3596.81419	0.02334	3596.8142	0.02662
3598.74278	0.02247	3598.7428	0.02572
1600.72696	0.02879	1600.727	0.02448
1602.65555	0.02941	1602.6556	0.02524
1604.58414	0.02997	1604.5841	0.02596
1606.51272	0.03047	1606.5127	0.02663
1608.44131	0.0309	1608.4413	0.02724
1610.3699	0.03125	1610.3699	0.02779
1612.29848	0.03153	1612.2985	0.02827
1614.22707	0.03174	1614.2271	0.02869
1616.15566	0.03188	1616.1557	0.02905
1618.08424	0.03195	1618.0842	0.02934
1620.01283	0.03195	1620.0128	0.02957
1621.94142	0.03188	1621.9414	0.02973
1623.87	0.03175	1623.87	0.02982
1625.79859	0.03155	1625.7986	0.02986
1627.72718	0.0313	1627.7272	0.02983
1629.65576	0.03099	1629.6558	0.02974
1631.58435	0.03063	1631.5844	0.02959
1633.51294	0.03022	1633.5129	0.02939
1635.44152	0.02976	1635.4415	0.02914

1637.37011	0.02927	1637.3701	0.02883
1639.2987	0.02874	1639.2987	0.02848
1641.22728	0.02818	1641.2273	0.02808
1643.15587	0.02759	1643.1559	0.02763
1645.08446	0.02697	1645.0845	0.02715
1647.01304	0.02633	1647.013	0.02662
1648.94163	0.02567	1648.9416	0.02606
1650.87022	0.02499	1650.8702	0.02546
1652.7988	0.0243	1652.7988	0.02483
1654.72739	0.02359	1654.7274	0.02417
1656.65598	0.02288	1656.656	0.02348
1658.58456	0.02215	1658.5846	0.02277
1660.51315	0.02142	1660.5132	0.02203
1662.44174	0.02068	1662.4417	0.02127
1664.37032	0.01995	1664.3703	0.02049
1666.29891	0.0192	1666.2989	0.0197
1668.2275	0.01846	1668.2275	0.0189
1670.15608	0.01772	1670.1561	0.01808
1672.08467	0.01698	1672.0847	0.01726
1674.01326	0.01625	1674.0133	0.01644
1675.94185	0.01552	1675.9419	0.01562
1677.87043	0.0148	1677.8704	0.0148
1679.79902	0.01408	1679.799	0.01398
1681.72761	0.01338	1681.7276	0.01318
1683.65619	0.01269	1683.6562	0.01238
1685.58478	0.012	1685.5848	0.0116
1687.51337	0.01134	1687.5134	0.01084
1689.44195	0.01068	1689.442	0.0101
1691.37054	0.01005	1691.3705	0.00938
1693.29913	0.00943	1693.2991	0.00869
1695.22771	0.00882	1695.2277	0.00802
1697.1563	0.00824	1697.1563	0.00738
1699.08489	0.00768	1699.0849	0.00678
1701.01347	0.00714	1701.0135	0.0062
1702.94206	0.00662	1702.9421	0.00565
1704.87065	0.00613	1704.8707	0.00513
1706.79923	0.00566	1706.7992	0.00465
1708.72782	0.00521	1708.7278	0.0042
1710.65641	0.00478	1710.6564	0.00378
1712.58499	0.00438	1712.585	0.00339
1714.51358	0.004	1714.5136	0.00303
1716.44217	0.00365	1716.4422	0.0027
1718.37075	0.00331	1718.3708	0.00239
1720.29934	0.003	1720.2993	0.00212
1722.22793	0.00271	1722.2279	0.00187
1724.15651	0.00245	1724.1565	0.00164
1726.0851	0.0022	1726.0851	0.00144
1728.01369	0.00197	1728.0137	0.00125
1729.94227	0.00176	1729.9423	0.00109
1731.87086	0.00157	1731.8709	0.00094383
1733.79945	0.0014	1733.7995	0.0008149
1735.72803	0.00124	1735.728	0.00070112
1737.65662	0.0011	1737.6566	0.0006011
1739.58521	0.00096689	1739.5852	0.00051354
1741.51379	0.00085024	1741.5138	0.00043718
1743.44238	0.00074556	1743.4424	0.00037086

1745.37097	0.00065194	1745.371	0.00031349
1747.29955	0.00056846	1747.2996	0.00026406
1749.22814	0.00049427	1749.2281	0.00022163
1751.15673	0.00042856	1751.1567	0.00018536
1753.08531	0.00037052	1753.0853	0.00015447
1755.0139	0.00031944	1755.0139	0.00012827
1756.94249	0.00027462	1756.9425	0.00010614
1758.87107	0.00023542	1758.8711	8.7511E-05
1760.79966	0.00020124	1760.7997	7.1895E-05
1762.72825	0.00017153	1762.7283	5.8856E-05
1764.65683	0.00014579	1764.6568	4.8009E-05
1766.58542	0.00012356	1766.5854	3.9021E-05
1768.51401	0.00010442	1768.514	3.1603E-05
1770.44259	8.7994E-05	1770.4426	2.5503E-05
1772.37118	7.3941E-05	1772.3712	2.0507E-05
1774.29977	6.1954E-05	1774.2998	1.6431E-05
1776.22835	5.1762E-05	1776.2284	1.3118E-05
1778.15694	4.3123E-05	1778.1569	1.0436E-05
1780.08553	3.5823E-05	1780.0855	8.2718E-06
1782.01411	2.9674E-05	1782.0141	6.5333E-06
1783.9427	0.00002451	1783.9427	5.1416E-06
1785.87129	2.0187E-05	1785.8713	4.032E-06
1787.79987	1.6578E-05	1787.7999	3.1504E-06
1789.72846	1.3576E-05	1789.7285	2.4528E-06
1791.65705	1.1086E-05	1791.6571	1.9029E-06
1793.58563	9.0262E-06	1793.5856	1.4709E-06
1795.51422	7.3283E-06	1795.5142	1.1329E-06
1797.44281	5.9327E-06	1797.4428	8.6949E-07
1799.37139	4.7891E-06	1799.3714	6.6492E-07

Fitting results of IR spectra of water-immersed coking coal with inhibitor

700-900

X Observed	Y Generated Coal	X Observed	Y Generated Coal+MgCl2	X Observed	Y Generated Coal+TEMPO
715.55356	7.6832E-05	715.55356	5.79196E-05	715.55356	7.68322E-05
717.43567	0.00013357	717.43567	9.57447E-05	717.43567	0.000114657
719.31777	0.00021868	719.31777	0.000161939	719.31777	0.000180851
721.31059	0.00034161	721.31059	0.000265957	721.31059	0.00028487
723.1927	0.00053073	723.1927	0.000426714	723.1927	0.000417258
725.18552	0.00079551	725.18552	0.000644208	725.18552	0.000606383
727.06762	0.00115	727.06762	0.000965721	727.06762	0.000852246
729.06044	0.00162	729.06044	0.00138	729.06044	0.00117
730.94255	0.0022	730.94255	0.00193	730.94255	0.00156
732.82466	0.00291	732.82466	0.00259	732.82466	0.00202
734.81747	0.00375	734.81747	0.00339	734.81747	0.00254
736.69958	0.00467	736.69958	0.00427	736.69958	0.00312
738.6924	0.00565	738.6924	0.0052	738.6924	0.00373
740.57451	0.00663	740.57451	0.00615	740.57451	0.00432
742.45661	0.00755	742.45661	0.00703	742.45661	0.00488
744.44943	0.00835	744.44943	0.00776	744.44943	0.00535
746.33154	0.00896	746.33154	0.00831	746.33154	0.00572
748.32436	0.00933	748.32436	0.00861	748.32436	0.00595
750.20646	0.00945	750.20646	0.00863	750.20646	0.00602
752.19928	0.00929	752.19928	0.00837	752.19928	0.00593
754.08139	0.00888	754.08139	0.00786	754.08139	0.00568
755.96349	0.00826	755.96349	0.00714	755.96349	0.00532
757.95631	0.00749	757.95631	0.00628	757.95631	0.00486
759.83842	0.00665	759.83842	0.00535	759.83842	0.00434
761.83124	0.00579	761.83124	0.00443	761.83124	0.00381
763.71335	0.00499	763.71335	0.00356	763.71335	0.00332
765.59545	0.00429	765.59545	0.0028	765.59545	0.00289
767.58827	0.00374	767.58827	0.00219	767.58827	0.00254
769.47038	0.00335	769.47038	0.00173	769.47038	0.0023
771.4632	0.00311	771.4632	0.00144	771.4632	0.00218
773.3453	0.00304	773.3453	0.00131	773.3453	0.00217
775.33812	0.00309	775.33812	0.00133	775.33812	0.00225
777.22023	0.00325	777.22023	0.00149	777.22023	0.00243
779.10233	0.00351	779.10233	0.00174	779.10233	0.00266
781.09515	0.00383	781.09515	0.00208	781.09515	0.00293
782.97726	0.00418	782.97726	0.00247	782.97726	0.00322
784.97008	0.00456	784.97008	0.00288	784.97008	0.00349
786.85218	0.00493	786.85218	0.00326	786.85218	0.00374
788.845	0.00529	788.845	0.00359	788.845	0.00394
790.72711	0.00562	790.72711	0.00387	790.72711	0.00411
792.60922	0.00591	792.60922	0.00408	792.60922	0.00424
794.60203	0.00616	794.60203	0.00422	794.60203	0.00432
796.48414	0.00636	796.48414	0.00431	796.48414	0.00438
798.47696	0.0065	798.47696	0.00436	798.47696	0.00443
800.35907	0.00657	800.35907	0.00437	800.35907	0.00446
802.24117	0.00659	802.24117	0.00434	802.24117	0.00447
804.23399	0.00653	804.23399	0.00428	804.23399	0.00446
806.1161	0.00639	806.1161	0.00417	806.1161	0.0044
808.10892	0.0062	808.10892	0.00399	808.10892	0.00429
809.99102	0.00593	809.99102	0.00375	809.99102	0.00411

811.98384	0.00561	811.98384	0.00342	811.98384	0.00387
813.86595	0.00524	813.86595	0.00305	813.86595	0.00356
815.74806	0.00484	815.74806	0.00262	815.74806	0.0032
817.74087	0.00443	817.74087	0.00219	817.74087	0.00281
819.62298	0.00401	819.62298	0.00177	819.62298	0.00242
821.6158	0.00361	821.6158	0.00139	821.6158	0.00206
823.49791	0.00324	823.49791	0.00107	823.49791	0.00175
825.38001	0.0029	825.38001	0.000823877	825.38001	0.0015
827.37283	0.0026	827.37283	0.000634752	827.37283	0.0013
829.25494	0.00235	829.25494	0.000502364	829.25494	0.00116
831.24776	0.00214	831.24776	0.000398345	831.24776	0.00109
833.12986	0.00198	833.12986	0.000313239	833.12986	0.00104
835.12268	0.00185	835.12268	0.000228132	835.12268	0.00102
837.00479	0.00177	837.00479	0.000171395	837.00479	0.00101
838.88689	0.00173	838.88689	0.000124113	838.88689	0.00101
840.87971	0.00174	840.87971	0.000114657	840.87971	0.00102
842.76182	0.00181	842.76182	0.00013357	842.76182	0.00104
844.75464	0.00193	844.75464	0.000199764	844.75464	0.00109
846.63674	0.00211	846.63674	0.000313239	846.63674	0.00116
848.62956	0.00237	848.62956	0.000492908	848.62956	0.00128
850.51167	0.00268	850.51167	0.000748227	850.51167	0.00145
852.39378	0.00307	852.39378	0.0011	852.39378	0.00167
854.38659	0.00351	854.38659	0.00154	854.38659	0.00195
856.2687	0.00399	856.2687	0.00209	856.2687	0.00227
858.26152	0.0045	858.26152	0.0027	858.26152	0.00263
860.14363	0.00501	860.14363	0.00336	860.14363	0.00299
862.02573	0.0055	862.02573	0.004	862.02573	0.00333
864.01855	0.00593	864.01855	0.00458	864.01855	0.00363
865.90066	0.00629	865.90066	0.00504	865.90066	0.00387
867.89348	0.00654	867.89348	0.00533	867.89348	0.00401
869.77558	0.0067	869.77558	0.00544	869.77558	0.00406
871.7684	0.00674	871.7684	0.00536	871.7684	0.004
873.65051	0.00669	873.65051	0.00512	873.65051	0.00386
875.53262	0.00654	875.53262	0.00477	875.53262	0.00363
877.52543	0.00631	877.52543	0.00435	877.52543	0.00336
879.40754	0.00602	879.40754	0.00393	879.40754	0.00307
881.40036	0.00571	881.40036	0.00356	881.40036	0.00278
883.28247	0.00539	883.28247	0.00325	883.28247	0.00253
885.27528	0.00508	885.27528	0.00302	885.27528	0.00232
887.15739	0.00478	887.15739	0.00286	887.15739	0.00216
889.0395	0.00449	889.0395	0.00274	889.0395	0.00205
891.03232	0.00424	891.03232	0.00266	891.03232	0.00199
892.91442	0.00399	892.91442	0.00256	892.91442	0.00193
894.90724	0.00376	894.90724	0.00245	894.90724	0.00187
896.78935	0.00353	896.78935	0.0023	896.78935	0.0018
898.67145	0.00329	898.67145	0.00211	898.67145	0.00168
1186.08082	0.01607	1186.08082	0.01373	1186.08082	0.01285
1188.00941	0.01584	1188.00941	0.01343	1188.00941	0.01267
1189.938	0.0156	1189.938	0.01311	1189.938	0.01248
1191.86658	0.01536	1191.86658	0.01279	1191.86658	0.01228
1193.79517	0.0151	1193.79517	0.01247	1193.79517	0.01208
1195.72376	0.01485	1195.72376	0.01214	1195.72376	0.01188
1197.65234	0.01459	1197.65234	0.01182	1197.65234	0.01168
1199.58093	0.01433	1199.58093	0.01149	1199.58093	0.01148
1201.50952	0.01408	1201.50952	0.01118	1201.50952	0.01128
1203.4381	0.01383	1203.4381	0.01087	1203.4381	0.01108

1205.36669	0.01359	1205.36669	0.01057	1205.36669	0.01089
1207.29528	0.01335	1207.29528	0.01028	1207.29528	0.0107
1209.22386	0.01313	1209.22386	0.01	1209.22386	0.01053
1211.15245	0.01292	1211.15245	0.00974	1211.15245	0.01036
1213.08104	0.01273	1213.08104	0.0095	1213.08104	0.0102
1215.00962	0.01254	1215.00962	0.00927	1215.00962	0.01005
1216.93821	0.01238	1216.93821	0.00905	1216.93821	0.0099
1218.8668	0.01222	1218.8668	0.00886	1218.8668	0.00977
1220.79538	0.01208	1220.79538	0.00868	1220.79538	0.00965
1222.72397	0.01195	1222.72397	0.00852	1222.72397	0.00953
1224.65256	0.01183	1224.65256	0.00837	1224.65256	0.00942
1226.58114	0.01173	1226.58114	0.00824	1226.58114	0.00932
1228.50973	0.01163	1228.50973	0.00812	1228.50973	0.00922
1230.43832	0.01154	1230.43832	0.00801	1230.43832	0.00913
1232.3669	0.01146	1232.3669	0.00791	1232.3669	0.00904
1234.29549	0.01138	1234.29549	0.00782	1234.29549	0.00895
1236.22408	0.0113	1236.22408	0.00773	1236.22408	0.00887
1238.15266	0.01122	1238.15266	0.00765	1238.15266	0.00878
1240.08125	0.01115	1240.08125	0.00756	1240.08125	0.00869
1242.00984	0.01107	1242.00984	0.00748	1242.00984	0.0086
1243.93842	0.01098	1243.93842	0.0074	1243.93842	0.0085
1245.86701	0.0109	1245.86701	0.00732	1245.86701	0.0084
1247.7956	0.0108	1247.7956	0.00724	1247.7956	0.0083
1249.72418	0.0107	1249.72418	0.00715	1249.72418	0.00819
1251.65277	0.0106	1251.65277	0.00706	1251.65277	0.00808
1253.58136	0.01049	1253.58136	0.00696	1253.58136	0.00796
1255.50994	0.01037	1255.50994	0.00686	1255.50994	0.00784
1257.43853	0.01024	1257.43853	0.00676	1257.43853	0.00771
1259.36712	0.01011	1259.36712	0.00665	1259.36712	0.00758
1261.2957	0.00998	1261.2957	0.00655	1261.2957	0.00745
1263.22429	0.00984	1263.22429	0.00643	1263.22429	0.00732
1265.15288	0.0097	1265.15288	0.00632	1265.15288	0.00719
1267.08146	0.00955	1267.08146	0.00621	1267.08146	0.00706
1269.01005	0.00941	1269.01005	0.0061	1269.01005	0.00693
1270.93864	0.00926	1270.93864	0.00599	1270.93864	0.0068
1272.86722	0.00912	1272.86722	0.00588	1272.86722	0.00668
1274.79581	0.00898	1274.79581	0.00578	1274.79581	0.00656
1276.7244	0.00884	1276.7244	0.00568	1276.7244	0.00644
1278.65298	0.0087	1278.65298	0.00558	1278.65298	0.00632
1280.58157	0.00857	1280.58157	0.00549	1280.58157	0.00621
1282.51016	0.00844	1282.51016	0.0054	1282.51016	0.00611
1284.43874	0.00832	1284.43874	0.00532	1284.43874	0.00601
1286.36733	0.00819	1286.36733	0.00524	1286.36733	0.00591
1288.29592	0.00807	1288.29592	0.00517	1288.29592	0.00581
1290.22451	0.00796	1290.22451	0.00509	1290.22451	0.00572
1292.15309	0.00784	1292.15309	0.00503	1292.15309	0.00563
1294.08168	0.00773	1294.08168	0.00496	1294.08168	0.00554
1296.01027	0.00761	1296.01027	0.00489	1296.01027	0.00545
1297.93885	0.0075	1297.93885	0.00483	1297.93885	0.00537
1299.86744	0.00738	1299.86744	0.00477	1299.86744	0.00528
1301.79603	0.00726	1301.79603	0.0047	1301.79603	0.00519
1303.72461	0.00714	1303.72461	0.00464	1303.72461	0.0051
1305.6532	0.00702	1305.6532	0.00457	1305.6532	0.005
1307.58179	0.00689	1307.58179	0.0045	1307.58179	0.00491
1309.51037	0.00676	1309.51037	0.00443	1309.51037	0.00481
1311.43896	0.00663	1311.43896	0.00436	1311.43896	0.00472

1313.36755	0.0065	1313.36755	0.00429	1313.36755	0.00462
1315.29613	0.00637	1315.29613	0.00422	1315.29613	0.00452
1317.22472	0.00624	1317.22472	0.00415	1317.22472	0.00443
1319.15331	0.00612	1319.15331	0.00409	1319.15331	0.00434
1321.08189	0.00599	1321.08189	0.00402	1321.08189	0.00425
1323.01048	0.00588	1323.01048	0.00396	1323.01048	0.00417
1324.93907	0.00577	1324.93907	0.00391	1324.93907	0.0041
1326.86765	0.00567	1326.86765	0.00386	1326.86765	0.00403
1328.79624	0.00558	1328.79624	0.00382	1328.79624	0.00398
1330.72483	0.00551	1330.72483	0.00378	1330.72483	0.00394
1332.65341	0.00545	1332.65341	0.00376	1332.65341	0.00391
1334.582	0.00542	1334.582	0.00375	1334.582	0.00389
1336.51059	0.0054	1336.51059	0.00376	1336.51059	0.00389
1338.43917	0.0054	1338.43917	0.00377	1338.43917	0.00391
1340.36776	0.00542	1340.36776	0.0038	1340.36776	0.00394
1342.29635	0.00546	1342.29635	0.00385	1342.29635	0.00399
1344.22493	0.00552	1344.22493	0.00391	1344.22493	0.00406
1346.15352	0.0056	1346.15352	0.00398	1346.15352	0.00414
1348.08211	0.00571	1348.08211	0.00406	1348.08211	0.00424
1350.01069	0.00583	1350.01069	0.00416	1350.01069	0.00436
1351.93928	0.00597	1351.93928	0.00427	1351.93928	0.00448
1353.86787	0.00613	1353.86787	0.00439	1353.86787	0.00462
1355.79645	0.00629	1355.79645	0.00452	1355.79645	0.00477
1357.72504	0.00647	1357.72504	0.00466	1357.72504	0.00492
1359.65363	0.00666	1359.65363	0.0048	1359.65363	0.00508
1361.58221	0.00685	1361.58221	0.00495	1361.58221	0.00524
1363.5108	0.00704	1363.5108	0.0051	1363.5108	0.00541
1365.43939	0.00723	1365.43939	0.00525	1365.43939	0.00557
1367.36797	0.00742	1367.36797	0.0054	1367.36797	0.00572
1369.29656	0.00761	1369.29656	0.00554	1369.29656	0.00587
1371.22515	0.00778	1371.22515	0.00569	1371.22515	0.00601
1373.15373	0.00794	1373.15373	0.00583	1373.15373	0.00614
1375.08232	0.00809	1375.08232	0.00596	1375.08232	0.00626
1377.01091	0.00823	1377.01091	0.00609	1377.01091	0.00636
1378.93949	0.00835	1378.93949	0.0062	1378.93949	0.00645
1380.86808	0.00845	1380.86808	0.00631	1380.86808	0.00653
1382.79667	0.00854	1382.79667	0.00641	1382.79667	0.00659
1384.72525	0.00861	1384.72525	0.00651	1384.72525	0.00663
1386.65384	0.00867	1386.65384	0.00659	1386.65384	0.00667
1388.58243	0.00871	1388.58243	0.00667	1388.58243	0.00669
1390.51101	0.00874	1390.51101	0.00674	1390.51101	0.0067
1392.4396	0.00876	1392.4396	0.0068	1392.4396	0.0067
1394.36819	0.00877	1394.36819	0.00685	1394.36819	0.00669
1396.29677	0.00877	1396.29677	0.00691	1396.29677	0.00667
1398.22536	0.00876	1398.22536	0.00695	1398.22536	0.00665
1400.15395	0.00875	1400.15395	0.007	1400.15395	0.00663
1402.08253	0.00874	1402.08253	0.00704	1402.08253	0.00661
1404.01112	0.00873	1404.01112	0.00708	1404.01112	0.00659
1405.93971	0.00872	1405.93971	0.00712	1405.93971	0.00657
1407.86829	0.00871	1407.86829	0.00716	1407.86829	0.00655
1409.79688	0.0087	1409.79688	0.0072	1409.79688	0.00654
1411.72547	0.0087	1411.72547	0.00724	1411.72547	0.00654
1413.65405	0.00871	1413.65405	0.00728	1413.65405	0.00654
1415.58264	0.00872	1415.58264	0.00732	1415.58264	0.00655
1417.51123	0.00873	1417.51123	0.00737	1417.51123	0.00657
1419.43981	0.00876	1419.43981	0.00741	1419.43981	0.00659

1421.3684	0.00878	1421.3684	0.00745	1421.3684	0.00662
1423.29699	0.00881	1423.29699	0.00748	1423.29699	0.00665
1425.22557	0.00884	1425.22557	0.00751	1425.22557	0.00669
1427.15416	0.00887	1427.15416	0.00754	1427.15416	0.00673
1429.08275	0.0089	1429.08275	0.00756	1429.08275	0.00676
1431.01133	0.00893	1431.01133	0.00758	1431.01133	0.0068
1432.93992	0.00896	1432.93992	0.00758	1432.93992	0.00683
1434.86851	0.00897	1434.86851	0.00757	1434.86851	0.00686
1436.79709	0.00899	1436.79709	0.00755	1436.79709	0.00689
1438.72568	0.00899	1438.72568	0.00752	1438.72568	0.0069
1440.65427	0.00898	1440.65427	0.00747	1440.65427	0.00691
1442.58285	0.00895	1442.58285	0.00741	1442.58285	0.0069
1444.51144	0.00892	1444.51144	0.00733	1444.51144	0.00688
1446.44003	0.00886	1446.44003	0.00724	1446.44003	0.00685
1448.36861	0.0088	1448.36861	0.00713	1448.36861	0.00681
1450.2972	0.00871	1450.2972	0.00701	1450.2972	0.00675
1452.22579	0.00861	1452.22579	0.00687	1452.22579	0.00668
1454.15437	0.0085	1454.15437	0.00671	1454.15437	0.00659
1456.08296	0.00836	1456.08296	0.00655	1456.08296	0.00649
1458.01155	0.00821	1458.01155	0.00637	1458.01155	0.00637
1459.94013	0.00805	1459.94013	0.00618	1459.94013	0.00625
1461.86872	0.00787	1461.86872	0.00598	1461.86872	0.00611
1463.79731	0.00768	1463.79731	0.00577	1463.79731	0.00597
1465.72589	0.00748	1465.72589	0.00556	1465.72589	0.00581
1467.65448	0.00726	1467.65448	0.00535	1467.65448	0.00565
1469.58307	0.00705	1469.58307	0.00513	1469.58307	0.00549
1471.51165	0.00682	1471.51165	0.00491	1471.51165	0.00532
1473.44024	0.00659	1473.44024	0.0047	1473.44024	0.00515
1475.36883	0.00637	1475.36883	0.00449	1475.36883	0.00498
1477.29741	0.00614	1477.29741	0.00429	1477.29741	0.00481
1479.226	0.00592	1479.226	0.00409	1479.226	0.00464
1481.15459	0.00571	1481.15459	0.0039	1481.15459	0.00448
1483.08318	0.00551	1483.08318	0.00372	1483.08318	0.00433
1485.01176	0.00532	1485.01176	0.00355	1485.01176	0.00418
1486.94035	0.00515	1486.94035	0.00339	1486.94035	0.00404
1488.86894	0.00499	1488.86894	0.00325	1488.86894	0.00391
1490.79752	0.00485	1490.79752	0.00312	1490.79752	0.00379
1492.72611	0.00473	1492.72611	0.003	1492.72611	0.00368
1494.6547	0.00463	1494.6547	0.00289	1494.6547	0.00358
1496.58328	0.00456	1496.58328	0.0028	1496.58328	0.00349
1498.51187	0.00451	1498.51187	0.00272	1498.51187	0.00341
1500.44046	0.00448	1500.44046	0.00265	1500.44046	0.00334
1502.36904	0.00448	1502.36904	0.0026	1502.36904	0.00329
1504.29763	0.0045	1504.29763	0.00256	1504.29763	0.00324
1506.22622	0.00454	1506.22622	0.00253	1506.22622	0.0032
1508.1548	0.00461	1508.1548	0.00252	1508.1548	0.00318
1510.08339	0.00469	1510.08339	0.00251	1510.08339	0.00316
1512.01198	0.00479	1512.01198	0.00252	1512.01198	0.00315
1513.94056	0.00491	1513.94056	0.00255	1513.94056	0.00315
1515.86915	0.00504	1515.86915	0.00258	1515.86915	0.00316
1517.79774	0.00519	1517.79774	0.00263	1517.79774	0.00317
1519.72632	0.00535	1519.72632	0.00269	1519.72632	0.0032
1521.65491	0.00553	1521.65491	0.00276	1521.65491	0.00323
1523.5835	0.00571	1523.5835	0.00285	1523.5835	0.00328
1525.51208	0.00591	1525.51208	0.00295	1525.51208	0.00333
1527.44067	0.00612	1527.44067	0.00307	1527.44067	0.00339

1529.36926	0.00634	1529.36926	0.00321	1529.36926	0.00347
1531.29784	0.00657	1531.29784	0.00337	1531.29784	0.00356
1533.22643	0.00682	1533.22643	0.00355	1533.22643	0.00367
1535.15502	0.00709	1535.15502	0.00375	1535.15502	0.00379
1537.0836	0.00738	1537.0836	0.00398	1537.0836	0.00393
1539.01219	0.00769	1539.01219	0.00424	1539.01219	0.0041
1540.94078	0.00802	1540.94078	0.00453	1540.94078	0.00429
1542.86936	0.00839	1542.86936	0.00485	1542.86936	0.0045
1544.79795	0.00878	1544.79795	0.0052	1544.79795	0.00475
1546.72654	0.00921	1546.72654	0.00559	1546.72654	0.00503
1548.65512	0.00967	1548.65512	0.00602	1548.65512	0.00534
1550.58371	0.01017	1550.58371	0.00649	1550.58371	0.00569
1552.5123	0.01071	1552.5123	0.007	1552.5123	0.00608
1554.44088	0.01129	1554.44088	0.00756	1554.44088	0.00651
1556.36947	0.01191	1556.36947	0.00816	1556.36947	0.00699
1558.29806	0.01257	1558.29806	0.00881	1558.29806	0.00752
1560.22664	0.01327	1560.22664	0.0095	1560.22664	0.0081
1562.15523	0.01401	1562.15523	0.01025	1562.15523	0.00872
1564.08382	0.01479	1564.08382	0.01104	1564.08382	0.0094
1566.0124	0.01561	1566.0124	0.01188	1566.0124	0.01012
1567.94099	0.01646	1567.94099	0.01277	1567.94099	0.0109
1569.86958	0.01733	1569.86958	0.0137	1569.86958	0.01172
1571.79816	0.01823	1571.79816	0.01467	1571.79816	0.01259
1573.72675	0.01916	1573.72675	0.01568	1573.72675	0.0135
1575.65534	0.0201	1575.65534	0.01673	1575.65534	0.01445
1577.58392	0.02105	1577.58392	0.01782	1577.58392	0.01544
1579.51251	0.02201	1579.51251	0.01893	1579.51251	0.01646
1581.4411	0.02297	1581.4411	0.02006	1581.4411	0.0175
1583.36968	0.02394	1583.36968	0.02122	1583.36968	0.01856
1585.29827	0.02489	1585.29827	0.02238	1585.29827	0.01964
1587.22686	0.02583	1587.22686	0.02356	1587.22686	0.02072
1589.15544	0.02676	1589.15544	0.02473	1589.15544	0.0218
1591.08403	0.02766	1591.08403	0.0259	1591.08403	0.02287
1593.01262	0.02854	1593.01262	0.02706	1593.01262	0.02392
1594.9412	0.02939	1594.9412	0.02819	1594.9412	0.02494
1596.86979	0.0302	1596.86979	0.02931	1596.86979	0.02593
1598.79838	0.03097	1598.79838	0.03039	1598.79838	0.02688
1600.72696	0.0317	1600.72696	0.03143	1600.72696	0.02778
1602.65555	0.03239	1602.65555	0.03242	1602.65555	0.02862
1604.58414	0.03302	1604.58414	0.03337	1604.58414	0.02939
1606.51272	0.0336	1606.51272	0.03426	1606.51272	0.0301
1608.44131	0.03413	1608.44131	0.03508	1608.44131	0.03073
1610.3699	0.03459	1610.3699	0.03584	1610.3699	0.03128
1612.29848	0.035	1612.29848	0.03654	1612.29848	0.03175
1614.22707	0.03535	1614.22707	0.03715	1614.22707	0.03213
1616.15566	0.03563	1616.15566	0.03769	1616.15566	0.03242
1618.08424	0.03585	1618.08424	0.03815	1618.08424	0.03263
1620.01283	0.03601	1620.01283	0.03853	1620.01283	0.03274
1621.94142	0.0361	1621.94142	0.03882	1621.94142	0.03277
1623.87	0.03613	1623.87	0.03903	1623.87	0.03271
1625.79859	0.0361	1625.79859	0.03916	1625.79859	0.03257
1627.72718	0.03599	1627.72718	0.0392	1627.72718	0.03235
1629.65576	0.03583	1629.65576	0.03916	1629.65576	0.03205
1631.58435	0.0356	1631.58435	0.03904	1631.58435	0.03168
1633.51294	0.03532	1633.51294	0.03883	1633.51294	0.03124
1635.44152	0.03497	1635.44152	0.03855	1635.44152	0.03075

1637.37011	0.03457	1637.37011	0.03819	1637.37011	0.03019
1639.2987	0.03411	1639.2987	0.03776	1639.2987	0.02959
1641.22728	0.03359	1641.22728	0.03725	1641.22728	0.02894
1643.15587	0.03303	1643.15587	0.03668	1643.15587	0.02826
1645.08446	0.03241	1645.08446	0.03605	1645.08446	0.02754
1647.01304	0.03175	1647.01304	0.03536	1647.01304	0.0268
1648.94163	0.03105	1648.94163	0.0346	1648.94163	0.02603
1650.87022	0.0303	1650.87022	0.0338	1650.87022	0.02525
1652.7988	0.02952	1652.7988	0.03295	1652.7988	0.02446
1654.72739	0.0287	1654.72739	0.03206	1654.72739	0.02365
1656.65598	0.02786	1656.65598	0.03112	1656.65598	0.02285
1658.58456	0.02698	1658.58456	0.03015	1658.58456	0.02204
1660.51315	0.02609	1660.51315	0.02915	1660.51315	0.02124
1662.44174	0.02517	1662.44174	0.02813	1662.44174	0.02044
1664.37032	0.02423	1664.37032	0.02708	1664.37032	0.01965
1666.29891	0.02328	1666.29891	0.02602	1666.29891	0.01887
1668.2275	0.02233	1668.2275	0.02494	1668.2275	0.01809
1670.15608	0.02136	1670.15608	0.02385	1670.15608	0.01734
1672.08467	0.0204	1672.08467	0.02276	1672.08467	0.01659
1674.01326	0.01944	1674.01326	0.02167	1674.01326	0.01586
1675.94185	0.01848	1675.94185	0.02059	1675.94185	0.01514
1677.87043	0.01753	1677.87043	0.01951	1677.87043	0.01445
1679.79902	0.01659	1679.79902	0.01845	1679.79902	0.01376
1681.72761	0.01566	1681.72761	0.0174	1681.72761	0.01309
1683.65619	0.01476	1683.65619	0.01637	1683.65619	0.01244
1685.58478	0.01387	1685.58478	0.01536	1685.58478	0.01181
1687.51337	0.013	1687.51337	0.01438	1687.51337	0.01119
1689.44195	0.01216	1689.44195	0.01343	1689.44195	0.0106
1691.37054	0.01135	1691.37054	0.01251	1691.37054	0.01001
1693.29913	0.01056	1693.29913	0.01162	1693.29913	0.00945
1695.22771	0.00981	1695.22771	0.01076	1695.22771	0.0089
1697.1563	0.00909	1697.1563	0.00994	1697.1563	0.00838
1699.08489	0.00839	1699.08489	0.00916	1699.08489	0.00787
1701.01347	0.00773	1701.01347	0.00842	1701.01347	0.00738
1702.94206	0.00711	1702.94206	0.00771	1702.94206	0.0069
1704.87065	0.00652	1704.87065	0.00705	1704.87065	0.00645
1706.79923	0.00596	1706.79923	0.00642	1706.79923	0.00601
1708.72782	0.00543	1708.72782	0.00584	1708.72782	0.0056
1710.65641	0.00494	1710.65641	0.00529	1710.65641	0.0052
1712.58499	0.00448	1712.58499	0.00478	1712.58499	0.00482
1714.51358	0.00405	1714.51358	0.0043	1714.51358	0.00446
1716.44217	0.00366	1716.44217	0.00386	1716.44217	0.00412
1718.37075	0.00329	1718.37075	0.00346	1718.37075	0.00379
1720.29934	0.00295	1720.29934	0.00309	1720.29934	0.00349
1722.22793	0.00264	1722.22793	0.00275	1722.22793	0.0032
1724.15651	0.00236	1724.15651	0.00244	1724.15651	0.00293
1726.0851	0.0021	1726.0851	0.00216	1726.0851	0.00267
1728.01369	0.00186	1728.01369	0.0019	1728.01369	0.00243
1729.94227	0.00164	1729.94227	0.00167	1729.94227	0.00221
1731.87086	0.00145	1731.87086	0.00147	1731.87086	0.00201
1733.79945	0.00128	1733.79945	0.00128	1733.79945	0.00182
1735.72803	0.00112	1735.72803	0.00112	1735.72803	0.00164
1737.65662	0.00097895	1737.65662	0.000969225	1737.65662	0.00148
1739.58521	0.00085373	1739.58521	0.000839069	1739.58521	0.00133
1741.51379	0.00074237	1741.51379	0.00072412	1741.51379	0.00119
1743.44238	0.00064367	1743.44238	0.000622957	1743.44238	0.00106

1745.37097	0.00055648	1745.37097	0.000534242	1745.37097	0.00094964
1747.29955	0.00047969	1747.29955	0.000456715	1747.29955	0.000845286
1749.22814	0.00041229	1749.22814	0.000389205	1749.22814	0.000750599
1751.15673	0.00035332	1751.15673	0.000330623	1751.15673	0.000664919
1753.08531	0.0003019	1753.08531	0.000279968	1753.08531	0.000587602
1755.0139	0.0002572	1755.0139	0.000236319	1755.0139	0.000518024
1756.94249	0.00021848	1756.94249	0.000198841	1756.94249	0.000455581
1758.87107	0.00018503	1758.87107	0.000166772	1758.87107	0.000399696
1760.79966	0.00015625	1760.79966	0.000139428	1760.79966	0.000349816
1762.72825	0.00013155	1762.72825	0.000116195	1762.72825	0.000305418
1764.65683	0.00011042	1764.65683	9.65227E-05	1764.65683	0.000266008
1766.58542	9.2415E-05	1766.58542	7.99239E-05	1766.58542	0.000231119
1768.51401	7.7114E-05	1768.51401	6.59671E-05	1768.51401	0.000200318
1770.44259	6.4155E-05	1770.44259	5.42724E-05	1770.44259	0.000173198
1772.37118	5.3213E-05	1772.37118	4.45073E-05	1772.37118	0.000149385
1774.29977	4.4006E-05	1774.29977	3.63817E-05	1774.29977	0.000128532
1776.22835	3.6283E-05	1776.22835	2.96437E-05	1776.22835	0.00011032
1778.15694	2.9826E-05	1778.15694	2.40757E-05	1778.15694	9.44576E-05
1780.08553	2.4444E-05	1780.08553	1.94904E-05	1780.08553	8.06783E-05
1782.01411	1.9973E-05	1782.01411	1.57274E-05	1782.01411	6.87408E-05
1783.9427	1.6271E-05	1783.9427	1.26499E-05	1783.9427	5.84265E-05
1785.87129	1.3215E-05	1785.87129	1.01417E-05	1785.87129	4.95385E-05
1787.79987	1.0701E-05	1787.79987	8.10455E-06	1787.79987	4.18999E-05
1789.72846	8.6391E-06	1789.72846	6.45561E-06	1789.72846	3.53524E-05
1791.65705	6.9533E-06	1791.65705	5.1255E-06	1791.65705	2.97552E-05
1793.58563	5.5796E-06	1793.58563	4.05627E-06	1793.58563	2.49829E-05
1795.51422	4.4638E-06	1795.51422	3.19968E-06	1795.51422	2.09247E-05
1797.44281	3.5603E-06	1797.44281	2.5158E-06	1797.44281	1.74828E-05
1799.37139	2.8311E-06	1799.37139	1.97167E-06	1799.37139	1.45714E-05

X Observed	Y Generated Coal+TPPI	X Observed	Y Generated Coal+PA
715.55356	4.8463E-05	715.55356	6.7376E-05
717.43567	7.6832E-05	717.43567	0.00011466
719.31777	0.00013357	719.31777	0.0001714
721.31059	0.00022813	721.31059	0.00024705
723.1927	0.00036052	723.1927	0.00036052
725.18552	0.0005591	725.18552	0.00051182
727.06762	0.00083333	727.06762	0.00070095
729.06044	0.00122	729.06044	0.00093735
730.94255	0.00171	730.94255	0.00121
732.82466	0.00234	732.82466	0.00153
734.81747	0.00308	734.81747	0.00189
736.69958	0.00394	736.69958	0.00227
738.6924	0.00489	738.6924	0.00265
740.57451	0.00586	740.57451	0.00302
742.45661	0.0068	742.45661	0.00334
744.44943	0.00764	744.44943	0.0036
746.33154	0.00832	746.33154	0.00379
748.32436	0.00877	748.32436	0.00388
750.20646	0.00896	750.20646	0.00386
752.19928	0.00885	752.19928	0.00375
754.08139	0.00848	754.08139	0.00354
755.96349	0.00789	755.96349	0.00325
757.95631	0.00712	757.95631	0.00292
759.83842	0.00626	759.83842	0.00255
761.83124	0.00538	761.83124	0.00219
763.71335	0.00455	763.71335	0.00184
765.59545	0.00382	765.59545	0.00153
767.58827	0.00324	767.58827	0.00128
769.47038	0.00284	769.47038	0.00111
771.4632	0.0026	771.4632	0.00101
773.3453	0.00253	773.3453	0.00101
775.33812	0.00257	775.33812	0.0011
777.22023	0.00272	777.22023	0.00128
779.10233	0.00294	779.10233	0.00152
781.09515	0.00318	781.09515	0.00182
782.97726	0.00341	782.97726	0.00214
784.97008	0.00361	784.97008	0.00245
786.85218	0.00376	786.85218	0.00272
788.845	0.00387	788.845	0.00295
790.72711	0.00393	790.72711	0.00311
792.60922	0.00394	792.60922	0.0032
794.60203	0.00394	794.60203	0.00323
796.48414	0.00394	796.48414	0.00322
798.47696	0.00394	798.47696	0.00317
800.35907	0.00393	800.35907	0.00311
802.24117	0.00392	802.24117	0.00305
804.23399	0.00387	804.23399	0.00296
806.1161	0.00376	806.1161	0.00286
808.10892	0.00359	808.10892	0.00272
809.99102	0.00333	809.99102	0.00254

811.98384	0.00301	811.98384	0.00232
813.86595	0.00264	813.86595	0.00206
815.74806	0.00223	815.74806	0.00178
817.74087	0.00184	817.74087	0.0015
819.62298	0.00146	819.62298	0.00123
821.6158	0.00114	821.6158	0.00099409
823.49791	0.00087116	823.49791	0.00079551
825.38001	0.00067258	825.38001	0.00063475
827.37283	0.00052128	827.37283	0.00051182
829.25494	0.0004078	829.25494	0.00043617
831.24776	0.00033215	831.24776	0.00037943
833.12986	0.00027541	833.12986	0.00034161
835.12268	0.00024705	835.12268	0.00031324
837.00479	0.00024705	837.00479	0.00030378
838.88689	0.00027541	838.88689	0.00029433
840.87971	0.00035106	840.87971	0.00029433
842.76182	0.00048345	842.76182	0.00030378
844.75464	0.00068203	844.75464	0.00033215
846.63674	0.00095627	846.63674	0.00037943
848.62956	0.00131	848.62956	0.00046454
850.51167	0.00175	850.51167	0.00058747
852.39378	0.00227	852.39378	0.00074823
854.38659	0.00287	854.38659	0.00096572
856.2687	0.00351	856.2687	0.00122
858.26152	0.00417	858.26152	0.0015
860.14363	0.0048	860.14363	0.00181
862.02573	0.00537	862.02573	0.00211
864.01855	0.00583	864.01855	0.00237
865.90066	0.00615	865.90066	0.0026
867.89348	0.00631	867.89348	0.00275
869.77558	0.00631	869.77558	0.00282
871.7684	0.00615	871.7684	0.00281
873.65051	0.00585	873.65051	0.00273
875.53262	0.00547	875.53262	0.00261
877.52543	0.00503	877.52543	0.00245
879.40754	0.00459	879.40754	0.00227
881.40036	0.00416	881.40036	0.00211
883.28247	0.00378	883.28247	0.00197
885.27528	0.00347	885.27528	0.00185
887.15739	0.00322	887.15739	0.00176
889.0395	0.00302	889.0395	0.00169
891.03232	0.00288	891.03232	0.00164
892.91442	0.00274	892.91442	0.00158
894.90724	0.00263	894.90724	0.00151
896.78935	0.00252	896.78935	0.00144
898.67145	0.00238	898.67145	0.00135
1186.08082	0.01474	1186.0808	0.01061
1188.00941	0.01451	1188.0094	0.0104
1189.938	0.01426	1189.938	0.01018
1191.86658	0.014	1191.8666	0.00996
1193.79517	0.01373	1193.7952	0.00973
1195.72376	0.01346	1195.7238	0.00949
1197.65234	0.01318	1197.6523	0.00925
1199.58093	0.0129	1199.5809	0.00901
1201.50952	0.01262	1201.5095	0.00878
1203.4381	0.01234	1203.4381	0.00854

1205.36669	0.01207	1205.3667	0.00832
1207.29528	0.0118	1207.2953	0.0081
1209.22386	0.01154	1209.2239	0.00789
1211.15245	0.01129	1211.1525	0.00769
1213.08104	0.01105	1213.081	0.0075
1215.00962	0.01082	1215.0096	0.00732
1216.93821	0.01061	1216.9382	0.00715
1218.8668	0.0104	1218.8668	0.007
1220.79538	0.01021	1220.7954	0.00686
1222.72397	0.01002	1222.724	0.00673
1224.65256	0.00985	1224.6526	0.00661
1226.58114	0.00969	1226.5811	0.0065
1228.50973	0.00954	1228.5097	0.0064
1230.43832	0.00939	1230.4383	0.00631
1232.3669	0.00925	1232.3669	0.00623
1234.29549	0.00911	1234.2955	0.00615
1236.22408	0.00898	1236.2241	0.00607
1238.15266	0.00884	1238.1527	0.006
1240.08125	0.00871	1240.0813	0.00593
1242.00984	0.00858	1242.0098	0.00586
1243.93842	0.00845	1243.9384	0.00579
1245.86701	0.00831	1245.867	0.00572
1247.7956	0.00817	1247.7956	0.00565
1249.72418	0.00803	1249.7242	0.00557
1251.65277	0.00788	1251.6528	0.00549
1253.58136	0.00773	1253.5814	0.00541
1255.50994	0.00757	1255.5099	0.00533
1257.43853	0.00742	1257.4385	0.00524
1259.36712	0.00726	1259.3671	0.00515
1261.2957	0.00709	1261.2957	0.00506
1263.22429	0.00693	1263.2243	0.00497
1265.15288	0.00677	1265.1529	0.00488
1267.08146	0.0066	1267.0815	0.00479
1269.01005	0.00644	1269.0101	0.0047
1270.93864	0.00629	1270.9386	0.00462
1272.86722	0.00613	1272.8672	0.00453
1274.79581	0.00598	1274.7958	0.00445
1276.7244	0.00584	1276.7244	0.00437
1278.65298	0.0057	1278.653	0.0043
1280.58157	0.00557	1280.5816	0.00423
1282.51016	0.00545	1282.5102	0.00417
1284.43874	0.00533	1284.4387	0.00411
1286.36733	0.00522	1286.3673	0.00405
1288.29592	0.00512	1288.2959	0.004
1290.22451	0.00502	1290.2245	0.00395
1292.15309	0.00493	1292.1531	0.0039
1294.08168	0.00484	1294.0817	0.00385
1296.01027	0.00476	1296.0103	0.0038
1297.93885	0.00469	1297.9389	0.00375
1299.86744	0.00461	1299.8674	0.0037
1301.79603	0.00455	1301.796	0.00365
1303.72461	0.00448	1303.7246	0.0036
1305.6532	0.00442	1305.6532	0.00354
1307.58179	0.00436	1307.5818	0.00348
1309.51037	0.0043	1309.5104	0.00342
1311.43896	0.00424	1311.439	0.00335

1313.36755	0.00419	1313.3676	0.00328
1315.29613	0.00414	1315.2961	0.00321
1317.22472	0.00409	1317.2247	0.00313
1319.15331	0.00404	1319.1533	0.00305
1321.08189	0.00401	1321.0819	0.00296
1323.01048	0.00397	1323.0105	0.00288
1324.93907	0.00394	1324.9391	0.00279
1326.86765	0.00392	1326.8677	0.00271
1328.79624	0.00391	1328.7962	0.00263
1330.72483	0.0039	1330.7248	0.00256
1332.65341	0.00391	1332.6534	0.0025
1334.582	0.00392	1334.582	0.00244
1336.51059	0.00394	1336.5106	0.0024
1338.43917	0.00398	1338.4392	0.00237
1340.36776	0.00403	1340.3678	0.00236
1342.29635	0.00409	1342.2964	0.00237
1344.22493	0.00416	1344.2249	0.0024
1346.15352	0.00424	1346.1535	0.00245
1348.08211	0.00433	1348.0821	0.00252
1350.01069	0.00443	1350.0107	0.00261
1351.93928	0.00453	1351.9393	0.00273
1353.86787	0.00465	1353.8679	0.00287
1355.79645	0.00477	1355.7965	0.00302
1357.72504	0.0049	1357.725	0.0032
1359.65363	0.00503	1359.6536	0.00339
1361.58221	0.00517	1361.5822	0.00359
1363.5108	0.0053	1363.5108	0.0038
1365.43939	0.00543	1365.4394	0.00401
1367.36797	0.00556	1367.368	0.00422
1369.29656	0.00569	1369.2966	0.00443
1371.22515	0.00581	1371.2252	0.00462
1373.15373	0.00593	1373.1537	0.0048
1375.08232	0.00604	1375.0823	0.00496
1377.01091	0.00614	1377.0109	0.0051
1378.93949	0.00624	1378.9395	0.00521
1380.86808	0.00633	1380.8681	0.0053
1382.79667	0.0064	1382.7967	0.00536
1384.72525	0.00647	1384.7253	0.00539
1386.65384	0.00653	1386.6538	0.0054
1388.58243	0.00659	1388.5824	0.00539
1390.51101	0.00664	1390.511	0.00535
1392.4396	0.00668	1392.4396	0.0053
1394.36819	0.00671	1394.3682	0.00524
1396.29677	0.00674	1396.2968	0.00516
1398.22536	0.00677	1398.2254	0.00509
1400.15395	0.00679	1400.154	0.00501
1402.08253	0.00682	1402.0825	0.00494
1404.01112	0.00684	1404.0111	0.00488
1405.93971	0.00686	1405.9397	0.00483
1407.86829	0.00688	1407.8683	0.0048
1409.79688	0.00691	1409.7969	0.00478
1411.72547	0.00693	1411.7255	0.00478
1413.65405	0.00696	1413.6541	0.0048
1415.58264	0.00699	1415.5826	0.00484
1417.51123	0.00702	1417.5112	0.00489
1419.43981	0.00705	1419.4398	0.00496

1421.3684	0.00709	1421.3684	0.00505
1423.29699	0.00712	1423.297	0.00514
1425.22557	0.00716	1425.2256	0.00524
1427.15416	0.00719	1427.1542	0.00534
1429.08275	0.00722	1429.0828	0.00545
1431.01133	0.00725	1431.0113	0.00555
1432.93992	0.00728	1432.9399	0.00564
1434.86851	0.00729	1434.8685	0.00573
1436.79709	0.00731	1436.7971	0.00581
1438.72568	0.00731	1438.7257	0.00587
1440.65427	0.00731	1440.6543	0.00592
1442.58285	0.0073	1442.5829	0.00595
1444.51144	0.00727	1444.5114	0.00596
1446.44003	0.00724	1446.44	0.00595
1448.36861	0.0072	1448.3686	0.00592
1450.2972	0.00714	1450.2972	0.00587
1452.22579	0.00707	1452.2258	0.00579
1454.15437	0.00699	1454.1544	0.0057
1456.08296	0.0069	1456.083	0.00559
1458.01155	0.0068	1458.0116	0.00546
1459.94013	0.00669	1459.9401	0.00532
1461.86872	0.00657	1461.8687	0.00516
1463.79731	0.00644	1463.7973	0.00499
1465.72589	0.0063	1465.7259	0.0048
1467.65448	0.00616	1467.6545	0.00462
1469.58307	0.00601	1469.5831	0.00442
1471.51165	0.00585	1471.5117	0.00422
1473.44024	0.00569	1473.4402	0.00402
1475.36883	0.00553	1475.3688	0.00383
1477.29741	0.00537	1477.2974	0.00364
1479.226	0.00521	1479.226	0.00345
1481.15459	0.00506	1481.1546	0.00328
1483.08318	0.0049	1483.0832	0.00312
1485.01176	0.00475	1485.0118	0.00297
1486.94035	0.00461	1486.9404	0.00283
1488.86894	0.00447	1488.8689	0.00271
1490.79752	0.00434	1490.7975	0.00261
1492.72611	0.00422	1492.7261	0.00253
1494.6547	0.0041	1494.6547	0.00247
1496.58328	0.004	1496.5833	0.00242
1498.51187	0.0039	1498.5119	0.0024
1500.44046	0.00382	1500.4405	0.00239
1502.36904	0.00374	1502.369	0.00241
1504.29763	0.00368	1504.2976	0.00243
1506.22622	0.00362	1506.2262	0.00248
1508.1548	0.00358	1508.1548	0.00253
1510.08339	0.00355	1510.0834	0.0026
1512.01198	0.00353	1512.012	0.00268
1513.94056	0.00352	1513.9406	0.00277
1515.86915	0.00353	1515.8692	0.00286
1517.79774	0.00355	1517.7977	0.00296
1519.72632	0.00358	1519.7263	0.00306
1521.65491	0.00363	1521.6549	0.00316
1523.5835	0.0037	1523.5835	0.00326
1525.51208	0.00378	1525.5121	0.00336
1527.44067	0.00389	1527.4407	0.00346

1529.36926	0.00401	1529.3693	0.00355
1531.29784	0.00415	1531.2978	0.00365
1533.22643	0.00432	1533.2264	0.00376
1535.15502	0.00452	1535.155	0.00386
1537.0836	0.00474	1537.0836	0.00397
1539.01219	0.00499	1539.0122	0.0041
1540.94078	0.00528	1540.9408	0.00423
1542.86936	0.00559	1542.8694	0.00438
1544.79795	0.00595	1544.798	0.00456
1546.72654	0.00634	1546.7265	0.00475
1548.65512	0.00677	1548.6551	0.00498
1550.58371	0.00724	1550.5837	0.00524
1552.5123	0.00776	1552.5123	0.00553
1554.44088	0.00832	1554.4409	0.00586
1556.36947	0.00892	1556.3695	0.00624
1558.29806	0.00956	1558.2981	0.00666
1560.22664	0.01025	1560.2266	0.00713
1562.15523	0.01098	1562.1552	0.00765
1564.08382	0.01176	1564.0838	0.00822
1566.0124	0.01257	1566.0124	0.00884
1567.94099	0.01342	1567.941	0.0095
1569.86958	0.0143	1569.8696	0.01022
1571.79816	0.01521	1571.7982	0.01098
1573.72675	0.01615	1573.7268	0.01178
1575.65534	0.01711	1575.6553	0.01261
1577.58392	0.01808	1577.5839	0.01349
1579.51251	0.01907	1579.5125	0.01439
1581.4411	0.02005	1581.4411	0.01531
1583.36968	0.02104	1583.3697	0.01626
1585.29827	0.02202	1585.2983	0.01721
1587.22686	0.02298	1587.2269	0.01817
1589.15544	0.02393	1589.1554	0.01913
1591.08403	0.02484	1591.084	0.02008
1593.01262	0.02572	1593.0126	0.02101
1594.9412	0.02656	1594.9412	0.02193
1596.86979	0.02736	1596.8698	0.02281
1598.79838	0.0281	1598.7984	0.02366
1600.72696	0.02879	1600.727	0.02448
1602.65555	0.02941	1602.6556	0.02524
1604.58414	0.02997	1604.5841	0.02596
1606.51272	0.03047	1606.5127	0.02663
1608.44131	0.0309	1608.4413	0.02724
1610.3699	0.03125	1610.3699	0.02779
1612.29848	0.03153	1612.2985	0.02827
1614.22707	0.03174	1614.2271	0.02869
1616.15566	0.03188	1616.1557	0.02905
1618.08424	0.03195	1618.0842	0.02934
1620.01283	0.03195	1620.0128	0.02957
1621.94142	0.03188	1621.9414	0.02973
1623.87	0.03175	1623.87	0.02982
1625.79859	0.03155	1625.7986	0.02986
1627.72718	0.0313	1627.7272	0.02983
1629.65576	0.03099	1629.6558	0.02974
1631.58435	0.03063	1631.5844	0.02959
1633.51294	0.03022	1633.5129	0.02939
1635.44152	0.02976	1635.4415	0.02914

1637.37011	0.02927	1637.3701	0.02883
1639.2987	0.02874	1639.2987	0.02848
1641.22728	0.02818	1641.2273	0.02808
1643.15587	0.02759	1643.1559	0.02763
1645.08446	0.02697	1645.0845	0.02715
1647.01304	0.02633	1647.013	0.02662
1648.94163	0.02567	1648.9416	0.02606
1650.87022	0.02499	1650.8702	0.02546
1652.7988	0.0243	1652.7988	0.02483
1654.72739	0.02359	1654.7274	0.02417
1656.65598	0.02288	1656.656	0.02348
1658.58456	0.02215	1658.5846	0.02277
1660.51315	0.02142	1660.5132	0.02203
1662.44174	0.02068	1662.4417	0.02127
1664.37032	0.01995	1664.3703	0.02049
1666.29891	0.0192	1666.2989	0.0197
1668.2275	0.01846	1668.2275	0.0189
1670.15608	0.01772	1670.1561	0.01808
1672.08467	0.01698	1672.0847	0.01726
1674.01326	0.01625	1674.0133	0.01644
1675.94185	0.01552	1675.9419	0.01562
1677.87043	0.0148	1677.8704	0.0148
1679.79902	0.01408	1679.799	0.01398
1681.72761	0.01338	1681.7276	0.01318
1683.65619	0.01269	1683.6562	0.01238
1685.58478	0.012	1685.5848	0.0116
1687.51337	0.01134	1687.5134	0.01084
1689.44195	0.01068	1689.442	0.0101
1691.37054	0.01005	1691.3705	0.00938
1693.29913	0.00943	1693.2991	0.00869
1695.22771	0.00882	1695.2277	0.00802
1697.1563	0.00824	1697.1563	0.00738
1699.08489	0.00768	1699.0849	0.00678
1701.01347	0.00714	1701.0135	0.0062
1702.94206	0.00662	1702.9421	0.00565
1704.87065	0.00613	1704.8707	0.00513
1706.79923	0.00566	1706.7992	0.00465
1708.72782	0.00521	1708.7278	0.0042
1710.65641	0.00478	1710.6564	0.00378
1712.58499	0.00438	1712.585	0.00339
1714.51358	0.004	1714.5136	0.00303
1716.44217	0.00365	1716.4422	0.0027
1718.37075	0.00331	1718.3708	0.00239
1720.29934	0.003	1720.2993	0.00212
1722.22793	0.00271	1722.2279	0.00187
1724.15651	0.00245	1724.1565	0.00164
1726.0851	0.0022	1726.0851	0.00144
1728.01369	0.00197	1728.0137	0.00125
1729.94227	0.00176	1729.9423	0.00109
1731.87086	0.00157	1731.8709	0.00094383
1733.79945	0.0014	1733.7995	0.0008149
1735.72803	0.00124	1735.728	0.00070112
1737.65662	0.0011	1737.6566	0.0006011
1739.58521	0.00096689	1739.5852	0.00051354
1741.51379	0.00085024	1741.5138	0.00043718
1743.44238	0.00074556	1743.4424	0.00037086

1745.37097	0.00065194	1745.371	0.00031349
1747.29955	0.00056846	1747.2996	0.00026406
1749.22814	0.00049427	1749.2281	0.00022163
1751.15673	0.00042856	1751.1567	0.00018536
1753.08531	0.00037052	1753.0853	0.00015447
1755.0139	0.00031944	1755.0139	0.00012827
1756.94249	0.00027462	1756.9425	0.00010614
1758.87107	0.00023542	1758.8711	8.7511E-05
1760.79966	0.00020124	1760.7997	7.1895E-05
1762.72825	0.00017153	1762.7283	5.8856E-05
1764.65683	0.00014579	1764.6568	4.8009E-05
1766.58542	0.00012356	1766.5854	3.9021E-05
1768.51401	0.00010442	1768.514	3.1603E-05
1770.44259	8.7994E-05	1770.4426	2.5503E-05
1772.37118	7.3941E-05	1772.3712	2.0507E-05
1774.29977	6.1954E-05	1774.2998	1.6431E-05
1776.22835	5.1762E-05	1776.2284	1.3118E-05
1778.15694	4.3123E-05	1778.1569	1.0436E-05
1780.08553	3.5823E-05	1780.0855	8.2718E-06
1782.01411	2.9674E-05	1782.0141	6.5333E-06
1783.9427	0.00002451	1783.9427	5.1416E-06
1785.87129	2.0187E-05	1785.8713	4.032E-06
1787.79987	1.6578E-05	1787.7999	3.1504E-06
1789.72846	1.3576E-05	1789.7285	2.4528E-06
1791.65705	1.1086E-05	1791.6571	1.9029E-06
1793.58563	9.0262E-06	1793.5856	1.4709E-06
1795.51422	7.3283E-06	1795.5142	1.1329E-06
1797.44281	5.9327E-06	1797.4428	8.6949E-07
1799.37139	4.7891E-06	1799.3714	6.6492E-07

Fitting results of IR spectra of water-immersed coking coal with inhibitor

**1000-1800**	X Observed	Y Generated Coal	X Observed	Y Generated Coal+MgCl2	X Observed	Y Generated Coal+TEMPO
	1000.9365	0.02555	1000.9365	0.02527	1000.9365	0.01354
	1002.86509	0.02693	1002.86509	0.02654	1002.86509	0.01471
	1004.79367	0.02829	1004.79367	0.02778	1004.79367	0.0159
	1006.72226	0.02959	1006.72226	0.02897	1006.72226	0.0171
	1008.65085	0.03085	1008.65085	0.0301	1008.65085	0.01831
	1010.57943	0.03203	1010.57943	0.03117	1010.57943	0.01951
	1012.50802	0.03314	1012.50802	0.03217	1012.50802	0.02068
	1014.43661	0.03416	1014.43661	0.03309	1014.43661	0.02183
	1016.36519	0.03508	1016.36519	0.03391	1016.36519	0.02292
	1018.29378	0.03589	1018.29378	0.03464	1018.29378	0.02396
	1020.22237	0.03658	1020.22237	0.03527	1020.22237	0.02493
	1022.15095	0.03716	1022.15095	0.03578	1022.15095	0.02582
	1024.07954	0.0376	1024.07954	0.03619	1024.07954	0.02662
	1026.00813	0.03792	1026.00813	0.03648	1026.00813	0.02732
	1027.93671	0.03812	1027.93671	0.03665	1027.93671	0.02791
	1029.8653	0.03818	1029.8653	0.03671	1029.8653	0.02838
	1031.79389	0.03811	1031.79389	0.03666	1031.79389	0.02874
	1033.72247	0.03793	1033.72247	0.0365	1033.72247	0.02898
	1035.65106	0.03762	1035.65106	0.03624	1035.65106	0.02911
	1037.57965	0.03721	1037.57965	0.03588	1037.57965	0.02911
	1039.50823	0.0367	1039.50823	0.03542	1039.50823	0.02901
	1041.43682	0.0361	1041.43682	0.03489	1041.43682	0.0288
	1043.36541	0.03542	1043.36541	0.03428	1043.36541	0.02849
	1045.29399	0.03467	1045.29399	0.0336	1045.29399	0.02809
	1047.22258	0.03388	1047.22258	0.03288	1047.22258	0.02762
	1049.15117	0.03304	1049.15117	0.03211	1049.15117	0.02708
	1051.07975	0.03217	1051.07975	0.03131	1051.07975	0.02649
	1053.00834	0.0313	1053.00834	0.03049	1053.00834	0.02586
	1054.93693	0.03042	1054.93693	0.02965	1054.93693	0.02521
	1056.86551	0.02956	1056.86551	0.02882	1056.86551	0.02455
	1058.7941	0.02873	1058.7941	0.02801	1058.7941	0.02388
	1060.72269	0.02793	1060.72269	0.02721	1060.72269	0.02323
	1062.65127	0.02718	1062.65127	0.02643	1062.65127	0.02261
	1064.57986	0.02648	1064.57986	0.0257	1064.57986	0.02202
	1066.50845	0.02584	1066.50845	0.025	1066.50845	0.02148
	1068.43703	0.02527	1068.43703	0.02435	1068.43703	0.02098
	1070.36562	0.02477	1070.36562	0.02375	1070.36562	0.02054
	1072.29421	0.02433	1072.29421	0.02321	1072.29421	0.02016
	1074.22279	0.02397	1074.22279	0.02272	1074.22279	0.01984
	1076.15138	0.02368	1076.15138	0.02228	1076.15138	0.01958
	1078.07997	0.02345	1078.07997	0.02189	1078.07997	0.01938
	1080.00855	0.02329	1080.00855	0.02156	1080.00855	0.01924
	1081.93714	0.02318	1081.93714	0.02127	1081.93714	0.01914
	1083.86573	0.02311	1083.86573	0.02103	1083.86573	0.01909
	1085.79431	0.02309	1085.79431	0.02083	1085.79431	0.01907
	1087.7229	0.0231	1087.7229	0.02066	1087.7229	0.01909
	1089.65149	0.02313	1089.65149	0.02052	1089.65149	0.01912
	1091.58007	0.02318	1091.58007	0.0204	1091.58007	0.01917
	1093.50866	0.02323	1093.50866	0.0203	1093.50866	0.01922
	1095.43725	0.02327	1095.43725	0.02021	1095.43725	0.01927

1097.36584	0.0233	1097.36584	0.02012	1097.36584	0.0193
1099.29442	0.02331	1099.29442	0.02003	1099.29442	0.01932
1101.22301	0.02329	1101.22301	0.01993	1101.22301	0.0193
1103.1516	0.02324	1103.1516	0.01982	1103.1516	0.01926
1105.08018	0.02314	1105.08018	0.01969	1105.08018	0.01918
1107.00877	0.023	1107.00877	0.01955	1107.00877	0.01905
1108.93736	0.02281	1108.93736	0.01937	1108.93736	0.01889
1110.86594	0.02257	1110.86594	0.01917	1110.86594	0.01867
1112.79453	0.02229	1112.79453	0.01894	1112.79453	0.01842
1114.72312	0.02196	1114.72312	0.01868	1114.72312	0.01812
1116.6517	0.02158	1116.6517	0.0184	1116.6517	0.01777
1118.58029	0.02116	1118.58029	0.01808	1118.58029	0.01739
1120.50888	0.0207	1120.50888	0.01774	1120.50888	0.01697
1122.43746	0.02021	1122.43746	0.01737	1122.43746	0.01653
1124.36605	0.01969	1124.36605	0.01698	1124.36605	0.01605
1126.29464	0.01915	1126.29464	0.01656	1126.29464	0.01556
1128.22322	0.0186	1128.22322	0.01613	1128.22322	0.01505
1130.15181	0.01803	1130.15181	0.01569	1130.15181	0.01453
1132.0804	0.01747	1132.0804	0.01523	1132.0804	0.014
1134.00898	0.01691	1134.00898	0.01477	1134.00898	0.01348
1135.93757	0.01636	1135.93757	0.01431	1135.93757	0.01297
1137.86616	0.01582	1137.86616	0.01384	1137.86616	0.01247
1139.79474	0.0153	1139.79474	0.01338	1139.79474	0.01198
1141.72333	0.01481	1141.72333	0.01293	1141.72333	0.01152
1143.65192	0.01434	1143.65192	0.01248	1143.65192	0.01108
1145.5805	0.0139	1145.5805	0.01205	1145.5805	0.01066
1147.50909	0.0135	1147.50909	0.01163	1147.50909	0.01027
1149.43768	0.01312	1149.43768	0.01122	1149.43768	0.00991
1151.36626	0.01278	1151.36626	0.01084	1151.36626	0.00958
1153.29485	0.01246	1153.29485	0.01047	1153.29485	0.00927
1155.22344	0.01218	1155.22344	0.01011	1155.22344	0.00899
1157.15202	0.01192	1157.15202	0.00978	1157.15202	0.00874
1159.08061	0.01169	1159.08061	0.00946	1159.08061	0.00851
1161.0092	0.01147	1161.0092	0.00916	1161.0092	0.00831
1162.93778	0.01128	1162.93778	0.00888	1162.93778	0.00812
1164.86637	0.0111	1164.86637	0.00861	1164.86637	0.00795
1166.79496	0.01093	1166.79496	0.00836	1166.79496	0.00779
1168.72354	0.01077	1168.72354	0.00811	1168.72354	0.00764
1170.65213	0.01062	1170.65213	0.00788	1170.65213	0.0075
1172.58072	0.01046	1172.58072	0.00765	1172.58072	0.00737
1174.5093	0.01031	1174.5093	0.00744	1174.5093	0.00724
1176.43789	0.01015	1176.43789	0.00723	1176.43789	0.00711
1178.36648	0.00998	1178.36648	0.00702	1178.36648	0.00697
1180.29506	0.00981	1180.29506	0.00681	1180.29506	0.00684
1182.22365	0.00963	1182.22365	0.00661	1182.22365	0.0067
1184.15224	0.00944	1184.15224	0.00641	1184.15224	0.00656
1186.08082	0.00925	1186.08082	0.00621	1186.08082	0.00641
1188.00941	0.00904	1188.00941	0.00602	1188.00941	0.00626
1189.938	0.00883	1189.938	0.00582	1189.938	0.0061
1191.86658	0.00862	1191.86658	0.00562	1191.86658	0.00594
1193.79517	0.0084	1193.79517	0.00542	1193.79517	0.00577
1195.72376	0.00818	1195.72376	0.00523	1195.72376	0.0056
1197.65234	0.00797	1197.65234	0.00504	1197.65234	0.00542
1199.58093	0.00776	1199.58093	0.00485	1199.58093	0.00525
1201.50952	0.00755	1201.50952	0.00466	1201.50952	0.00507
1203.4381	0.00735	1203.4381	0.00448	1203.4381	0.0049

1205.36669	0.00717	1205.36669	0.00431	1205.36669	0.00472
1207.29528	0.007	1207.29528	0.00414	1207.29528	0.00455
1209.22386	0.00684	1209.22386	0.00399	1209.22386	0.00439
1211.15245	0.0067	1211.15245	0.00384	1211.15245	0.00423
1213.08104	0.00658	1213.08104	0.00371	1213.08104	0.00407
1215.00962	0.00647	1215.00962	0.00359	1215.00962	0.00393
1216.93821	0.00639	1216.93821	0.00347	1216.93821	0.00379
1218.8668	0.00632	1218.8668	0.00338	1218.8668	0.00365
1220.79538	0.00627	1220.79538	0.00329	1220.79538	0.00353
1222.72397	0.00623	1222.72397	0.00321	1222.72397	0.00341
1224.65256	0.00621	1224.65256	0.00315	1224.65256	0.0033
1226.58114	0.00621	1226.58114	0.0031	1226.58114	0.0032
1228.50973	0.00621	1228.50973	0.00305	1228.50973	0.00311
1230.43832	0.00622	1230.43832	0.00302	1230.43832	0.00302
1232.3669	0.00624	1232.3669	0.00299	1232.3669	0.00294
1234.29549	0.00626	1234.29549	0.00296	1234.29549	0.00287
1236.22408	0.00627	1236.22408	0.00294	1236.22408	0.00279
1238.15266	0.00629	1238.15266	0.00292	1238.15266	0.00273
1240.08125	0.0063	1240.08125	0.00289	1240.08125	0.00267
1242.00984	0.0063	1242.00984	0.00287	1242.00984	0.00261
1243.93842	0.0063	1243.93842	0.00284	1243.93842	0.00255
1245.86701	0.00628	1245.86701	0.0028	1245.86701	0.0025
1247.7956	0.00625	1247.7956	0.00277	1247.7956	0.00245
1249.72418	0.00622	1249.72418	0.00272	1249.72418	0.0024
1251.65277	0.00617	1251.65277	0.00267	1251.65277	0.00235
1253.58136	0.00611	1253.58136	0.00262	1253.58136	0.0023
1255.50994	0.00605	1255.50994	0.00257	1255.50994	0.00226
1257.43853	0.00597	1257.43853	0.00251	1257.43853	0.00221
1259.36712	0.0059	1259.36712	0.00245	1259.36712	0.00217
1261.2957	0.00582	1261.2957	0.00239	1261.2957	0.00214
1263.22429	0.00574	1263.22429	0.00234	1263.22429	0.0021
1265.15288	0.00566	1265.15288	0.00229	1265.15288	0.00208
1267.08146	0.00559	1267.08146	0.00225	1267.08146	0.00205
1269.01005	0.00553	1269.01005	0.00222	1269.01005	0.00203
1270.93864	0.00548	1270.93864	0.0022	1270.93864	0.00202
1272.86722	0.00544	1272.86722	0.00219	1272.86722	0.00202
1274.79581	0.00542	1274.79581	0.0022	1274.79581	0.00203
1276.7244	0.00541	1276.7244	0.00222	1276.7244	0.00204
1278.65298	0.00542	1278.65298	0.00226	1278.65298	0.00207
1280.58157	0.00545	1280.58157	0.00232	1280.58157	0.00211
1282.51016	0.0055	1282.51016	0.00239	1282.51016	0.00216
1284.43874	0.00556	1284.43874	0.00247	1284.43874	0.00221
1286.36733	0.00564	1286.36733	0.00257	1286.36733	0.00229
1288.29592	0.00574	1288.29592	0.00268	1288.29592	0.00237
1290.22451	0.00585	1290.22451	0.0028	1290.22451	0.00246
1292.15309	0.00596	1292.15309	0.00293	1292.15309	0.00256
1294.08168	0.00609	1294.08168	0.00307	1294.08168	0.00267
1296.01027	0.00623	1296.01027	0.00321	1296.01027	0.00279
1297.93885	0.00636	1297.93885	0.00335	1297.93885	0.00291
1299.86744	0.0065	1299.86744	0.00349	1299.86744	0.00304
1301.79603	0.00663	1301.79603	0.00363	1301.79603	0.00317
1303.72461	0.00676	1303.72461	0.00376	1303.72461	0.0033
1305.6532	0.00688	1305.6532	0.00389	1305.6532	0.00342
1307.58179	0.007	1307.58179	0.00401	1307.58179	0.00355
1309.51037	0.0071	1309.51037	0.00412	1309.51037	0.00366
1311.43896	0.0072	1311.43896	0.00423	1311.43896	0.00377

1313.36755	0.00728	1313.36755	0.00432	1313.36755	0.00387
1315.29613	0.00735	1315.29613	0.00441	1315.29613	0.00396
1317.22472	0.00741	1317.22472	0.00448	1317.22472	0.00404
1319.15331	0.00746	1319.15331	0.00456	1319.15331	0.0041
1321.08189	0.00751	1321.08189	0.00463	1321.08189	0.00416
1323.01048	0.00755	1323.01048	0.00469	1323.01048	0.0042
1324.93907	0.00758	1324.93907	0.00476	1324.93907	0.00424
1326.86765	0.00761	1326.86765	0.00483	1326.86765	0.00426
1328.79624	0.00765	1328.79624	0.00491	1328.79624	0.00428
1330.72483	0.00769	1330.72483	0.005	1330.72483	0.0043
1332.65341	0.00774	1332.65341	0.0051	1332.65341	0.00431
1334.582	0.0078	1334.582	0.00522	1334.582	0.00433
1336.51059	0.00787	1336.51059	0.00536	1336.51059	0.00435
1338.43917	0.00795	1338.43917	0.00552	1338.43917	0.00438
1340.36776	0.00805	1340.36776	0.0057	1340.36776	0.00442
1342.29635	0.00817	1342.29635	0.00591	1342.29635	0.00448
1344.22493	0.00831	1344.22493	0.00614	1344.22493	0.00455
1346.15352	0.00846	1346.15352	0.0064	1346.15352	0.00464
1348.08211	0.00863	1348.08211	0.0067	1348.08211	0.00475
1350.01069	0.00883	1350.01069	0.00701	1350.01069	0.00489
1351.93928	0.00904	1351.93928	0.00736	1351.93928	0.00504
1353.86787	0.00926	1353.86787	0.00773	1353.86787	0.00522
1355.79645	0.0095	1355.79645	0.00812	1355.79645	0.00542
1357.72504	0.00975	1357.72504	0.00854	1357.72504	0.00564
1359.65363	0.01001	1359.65363	0.00897	1359.65363	0.00588
1361.58221	0.01028	1361.58221	0.00942	1361.58221	0.00613
1363.5108	0.01055	1363.5108	0.00988	1363.5108	0.00639
1365.43939	0.01082	1365.43939	0.01035	1365.43939	0.00665
1367.36797	0.01109	1367.36797	0.01083	1367.36797	0.00692
1369.29656	0.01136	1369.29656	0.0113	1369.29656	0.00719
1371.22515	0.01162	1371.22515	0.01177	1371.22515	0.00745
1373.15373	0.01187	1373.15373	0.01224	1373.15373	0.0077
1375.08232	0.01212	1375.08232	0.0127	1375.08232	0.00794
1377.01091	0.01235	1377.01091	0.01315	1377.01091	0.00816
1378.93949	0.01257	1378.93949	0.01358	1378.93949	0.00837
1380.86808	0.01279	1380.86808	0.01399	1380.86808	0.00855
1382.79667	0.01299	1382.79667	0.01439	1382.79667	0.00872
1384.72525	0.01318	1384.72525	0.01476	1384.72525	0.00886
1386.65384	0.01336	1386.65384	0.01511	1386.65384	0.00898
1388.58243	0.01354	1388.58243	0.01544	1388.58243	0.00907
1390.51101	0.01371	1390.51101	0.01574	1390.51101	0.00916
1392.4396	0.01387	1392.4396	0.01603	1392.4396	0.00922
1394.36819	0.01404	1394.36819	0.01629	1394.36819	0.00928
1396.29677	0.0142	1396.29677	0.01653	1396.29677	0.00932
1398.22536	0.01437	1398.22536	0.01674	1398.22536	0.00936
1400.15395	0.01455	1400.15395	0.01694	1400.15395	0.0094
1402.08253	0.01473	1402.08253	0.01712	1402.08253	0.00944
1404.01112	0.01491	1404.01112	0.01728	1404.01112	0.00949
1405.93971	0.01511	1405.93971	0.01743	1405.93971	0.00954
1407.86829	0.01532	1407.86829	0.01756	1407.86829	0.00961
1409.79688	0.01553	1409.79688	0.01767	1409.79688	0.00969
1411.72547	0.01576	1411.72547	0.01778	1411.72547	0.00978
1413.65405	0.01599	1413.65405	0.01787	1413.65405	0.00989
1415.58264	0.01623	1415.58264	0.01795	1415.58264	0.01001
1417.51123	0.01647	1417.51123	0.01801	1417.51123	0.01015
1419.43981	0.01671	1419.43981	0.01807	1419.43981	0.0103

1421.3684	0.01695	1421.3684	0.01811	1421.3684	0.01045
1423.29699	0.01718	1423.29699	0.01814	1423.29699	0.01061
1425.22557	0.0174	1425.22557	0.01815	1425.22557	0.01077
1427.15416	0.01761	1427.15416	0.01815	1427.15416	0.01093
1429.08275	0.01779	1429.08275	0.01813	1429.08275	0.01109
1431.01133	0.01795	1431.01133	0.01809	1431.01133	0.01123
1432.93992	0.01807	1432.93992	0.01803	1432.93992	0.01135
1434.86851	0.01816	1434.86851	0.01795	1434.86851	0.01145
1436.79709	0.01821	1436.79709	0.01785	1436.79709	0.01153
1438.72568	0.01822	1438.72568	0.01772	1438.72568	0.01158
1440.65427	0.01818	1440.65427	0.01756	1440.65427	0.0116
1442.58285	0.01809	1442.58285	0.01737	1442.58285	0.01158
1444.51144	0.01795	1444.51144	0.01716	1444.51144	0.01152
1446.44003	0.01776	1446.44003	0.01692	1446.44003	0.01142
1448.36861	0.01752	1448.36861	0.01664	1448.36861	0.01129
1450.2972	0.01722	1450.2972	0.01634	1450.2972	0.01111
1452.22579	0.01687	1452.22579	0.016	1452.22579	0.0109
1454.15437	0.01648	1454.15437	0.01564	1454.15437	0.01065
1456.08296	0.01603	1456.08296	0.01525	1456.08296	0.01036
1458.01155	0.01554	1458.01155	0.01483	1458.01155	0.01004
1459.94013	0.01502	1459.94013	0.01439	1459.94013	0.00969
1461.86872	0.01446	1461.86872	0.01393	1461.86872	0.00931
1463.79731	0.01386	1463.79731	0.01344	1463.79731	0.00891
1465.72589	0.01325	1465.72589	0.01294	1465.72589	0.00849
1467.65448	0.01261	1467.65448	0.01242	1467.65448	0.00805
1469.58307	0.01196	1469.58307	0.01189	1469.58307	0.00761
1471.51165	0.0113	1471.51165	0.01135	1471.51165	0.00715
1473.44024	0.01065	1473.44024	0.01081	1473.44024	0.0067
1475.36883	0.00999	1475.36883	0.01026	1475.36883	0.00625
1477.29741	0.00934	1477.29741	0.00971	1477.29741	0.0058
1479.226	0.00871	1479.226	0.00916	1479.226	0.00536
1481.15459	0.00809	1481.15459	0.00862	1481.15459	0.00493
1483.08318	0.0075	1483.08318	0.00808	1483.08318	0.00452
1485.01176	0.00693	1485.01176	0.00756	1485.01176	0.00412
1486.94035	0.0064	1486.94035	0.00705	1486.94035	0.00374
1488.86894	0.00589	1488.86894	0.00656	1488.86894	0.00338
1490.79752	0.00542	1490.79752	0.00609	1490.79752	0.00305
1492.72611	0.00499	1492.72611	0.00563	1492.72611	0.00273
1494.6547	0.0046	1494.6547	0.0052	1494.6547	0.00244
1496.58328	0.00425	1496.58328	0.00479	1496.58328	0.00216
1498.51187	0.00394	1498.51187	0.0044	1498.51187	0.00192
1500.44046	0.00367	1500.44046	0.00404	1500.44046	0.00169
1502.36904	0.00344	1502.36904	0.00371	1502.36904	0.00148
1504.29763	0.00326	1504.29763	0.00341	1504.29763	0.00129
1506.22622	0.00311	1506.22622	0.00314	1506.22622	0.00113
1508.1548	0.00301	1508.1548	0.00291	1508.1548	0.000981459
1510.08339	0.00294	1510.08339	0.0027	1510.08339	0.000852906
1512.01198	0.00292	1512.01198	0.00253	1512.01198	0.000742223
1513.94056	0.00293	1513.94056	0.00241	1513.94056	0.000649003
1515.86915	0.00298	1515.86915	0.00232	1515.86915	0.000573074
1517.79774	0.00307	1517.79774	0.00227	1517.79774	0.00051456
1519.72632	0.00319	1519.72632	0.00227	1519.72632	0.00047395
1521.65491	0.00334	1521.65491	0.00232	1521.65491	0.000452146
1523.5835	0.00353	1523.5835	0.00241	1523.5835	0.000450518
1525.51208	0.00375	1525.51208	0.00256	1525.51208	0.000470933
1527.44067	0.00401	1527.44067	0.00277	1527.44067	0.000515775

1529.36926	0.00429	1529.36926	0.00303	1529.36926	0.000587933
1531.29784	0.0046	1531.29784	0.00336	1531.29784	0.000690769
1533.22643	0.00494	1533.22643	0.00374	1533.22643	0.00082804
1535.15502	0.0053	1535.15502	0.00419	1535.15502	0.001
1537.0836	0.0057	1537.0836	0.0047	1537.0836	0.00122
1539.01219	0.00612	1539.01219	0.00527	1539.01219	0.00149
1540.94078	0.00656	1540.94078	0.00591	1540.94078	0.0018
1542.86936	0.00704	1542.86936	0.0066	1542.86936	0.00217
1544.79795	0.00754	1544.79795	0.00734	1544.79795	0.0026
1546.72654	0.00806	1546.72654	0.00814	1546.72654	0.00308
1548.65512	0.00862	1548.65512	0.00898	1548.65512	0.00362
1550.58371	0.0092	1550.58371	0.00985	1550.58371	0.00422
1552.5123	0.00982	1552.5123	0.01076	1552.5123	0.00488
1554.44088	0.01046	1554.44088	0.0117	1554.44088	0.00558
1556.36947	0.01114	1556.36947	0.01265	1556.36947	0.00632
1558.29806	0.01184	1558.29806	0.01362	1558.29806	0.0071
1560.22664	0.01259	1560.22664	0.01459	1560.22664	0.00791
1562.15523	0.01337	1562.15523	0.01557	1562.15523	0.00874
1564.08382	0.01418	1564.08382	0.01656	1564.08382	0.00958
1566.0124	0.01504	1566.0124	0.01755	1566.0124	0.01042
1567.94099	0.01593	1567.94099	0.01855	1567.94099	0.01126
1569.86958	0.01686	1569.86958	0.01956	1569.86958	0.01209
1571.79816	0.01782	1571.79816	0.0206	1571.79816	0.0129
1573.72675	0.01883	1573.72675	0.02167	1573.72675	0.01371
1575.65534	0.01986	1575.65534	0.0228	1575.65534	0.0145
1577.58392	0.02093	1577.58392	0.02399	1577.58392	0.01528
1579.51251	0.02202	1579.51251	0.02525	1579.51251	0.01606
1581.4411	0.02314	1581.4411	0.02662	1581.4411	0.01684
1583.36968	0.02427	1583.36968	0.02809	1583.36968	0.01764
1585.29827	0.02541	1585.29827	0.02969	1585.29827	0.01846
1587.22686	0.02656	1587.22686	0.03143	1587.22686	0.01931
1589.15544	0.02769	1589.15544	0.0333	1589.15544	0.02019
1591.08403	0.02881	1591.08403	0.03531	1591.08403	0.0211
1593.01262	0.0299	1593.01262	0.03745	1593.01262	0.02204
1594.9412	0.03096	1594.9412	0.03971	1594.9412	0.02301
1596.86979	0.03197	1596.86979	0.04208	1596.86979	0.02399
1598.79838	0.03291	1598.79838	0.04452	1598.79838	0.02496
1600.72696	0.03379	1600.72696	0.047	1600.72696	0.02592
1602.65555	0.03459	1602.65555	0.0495	1602.65555	0.02683
1604.58414	0.0353	1604.58414	0.05196	1604.58414	0.02769
1606.51272	0.0359	1606.51272	0.05435	1606.51272	0.02845
1608.44131	0.03641	1608.44131	0.05662	1608.44131	0.02912
1610.3699	0.0368	1610.3699	0.05874	1610.3699	0.02966
1612.29848	0.03707	1612.29848	0.06067	1612.29848	0.03006
1614.22707	0.03722	1614.22707	0.06238	1614.22707	0.03031
1616.15566	0.03725	1616.15566	0.06383	1616.15566	0.03042
1618.08424	0.03716	1618.08424	0.065	1618.08424	0.03036
1620.01283	0.03694	1620.01283	0.0659	1620.01283	0.03016
1621.94142	0.03661	1621.94142	0.0665	1621.94142	0.02982
1623.87	0.03617	1623.87	0.0668	1623.87	0.02936
1625.79859	0.03562	1625.79859	0.06683	1625.79859	0.02879
1627.72718	0.03497	1627.72718	0.06657	1627.72718	0.02814
1629.65576	0.03423	1629.65576	0.06605	1629.65576	0.02741
1631.58435	0.0334	1631.58435	0.06528	1631.58435	0.02664
1633.51294	0.03251	1633.51294	0.06428	1633.51294	0.02583
1635.44152	0.03155	1635.44152	0.06306	1635.44152	0.02501

1637.37011	0.03055	1637.37011	0.06163	1637.37011	0.02419
1639.2987	0.0295	1639.2987	0.06	1639.2987	0.02338
1641.22728	0.02842	1641.22728	0.05818	1641.22728	0.02257
1643.15587	0.02732	1643.15587	0.05618	1643.15587	0.02179
1645.08446	0.0262	1645.08446	0.05401	1645.08446	0.02102
1647.01304	0.02508	1647.01304	0.05167	1647.01304	0.02026
1648.94163	0.02396	1648.94163	0.04918	1648.94163	0.01951
1650.87022	0.02285	1650.87022	0.04654	1650.87022	0.01877
1652.7988	0.02176	1652.7988	0.04378	1652.7988	0.01802
1654.72739	0.02068	1654.72739	0.04092	1654.72739	0.01727
1656.65598	0.01963	1656.65598	0.03798	1656.65598	0.01651
1658.58456	0.0186	1658.58456	0.03499	1658.58456	0.01574
1660.51315	0.0176	1660.51315	0.03199	1660.51315	0.01495
1662.44174	0.01663	1662.44174	0.02901	1662.44174	0.01414
1664.37032	0.0157	1664.37032	0.02608	1664.37032	0.01332
1666.29891	0.01479	1666.29891	0.02324	1666.29891	0.01249
1668.2275	0.01392	1668.2275	0.02051	1668.2275	0.01165
1670.15608	0.01308	1670.15608	0.01794	1670.15608	0.01081
1672.08467	0.01227	1672.08467	0.01553	1672.08467	0.00997
1674.01326	0.0115	1674.01326	0.01332	1674.01326	0.00915
1675.94185	0.01075	1675.94185	0.0113	1675.94185	0.00834
1677.87043	0.01004	1677.87043	0.00949	1677.87043	0.00756
1679.79902	0.00936	1679.79902	0.00788	1679.79902	0.00681
1681.72761	0.0087	1681.72761	0.00648	1681.72761	0.00609
1683.65619	0.00808	1683.65619	0.00527	1683.65619	0.00541
1685.58478	0.00749	1685.58478	0.00424	1685.58478	0.00478
1687.51337	0.00692	1687.51337	0.00337	1687.51337	0.00419
1689.44195	0.00639	1689.44195	0.00265	1689.44195	0.00365
1691.37054	0.00588	1691.37054	0.00206	1691.37054	0.00316
1693.29913	0.00539	1693.29913	0.00158	1693.29913	0.00272
1695.22771	0.00494	1695.22771	0.0012	1695.22771	0.00232
1697.1563	0.00451	1697.1563	0.000904668	1697.1563	0.00197
1699.08489	0.00411	1699.08489	0.000672017	1699.08489	0.00166
1701.01347	0.00373	1701.01347	0.000493463	1701.01347	0.00138
1702.94206	0.00338	1702.94206	0.000358177	1702.94206	0.00115
1704.87065	0.00305	1704.87065	0.000256978	1704.87065	0.000948296
1706.79923	0.00274	1706.79923	0.000182237	1706.79923	0.000776679
1708.72782	0.00246	1708.72782	0.000127736	1708.72782	0.000631728
1710.65641	0.0022	1710.65641	8.84938E-05	1710.65641	0.000510279
1712.58499	0.00196	1712.58499	6.05944E-05	1712.58499	0.00040933
1714.51358	0.00174	1714.51358	4.10077E-05	1714.51358	0.000326082
1716.44217	0.00154	1716.44217	2.74288E-05	1716.44217	0.000257967
1718.37075	0.00136	1718.37075	1.81323E-05	1718.37075	0.000202669
1720.29934	0.0012	1720.29934	1.18468E-05	1720.29934	0.000158123
1722.22793	0.00105	1722.22793	7.64984E-06	1722.22793	0.000122515
1724.15651	0.00091958	1724.15651	4.88207E-06	1724.15651	9.42681E-05
1726.0851	0.00080098	1726.0851	3.07934E-06	1726.0851	7.20319E-05
1728.01369	0.00069529	1728.01369	1.91962E-06	1728.01369	5.46598E-05
1729.94227	0.00060146	1729.94227	1.18271E-06	1729.94227	4.11903E-05
1731.87086	0.0005185	1731.87086	7.20197E-07	1731.87086	3.08252E-05
1733.79945	0.00044543	1733.79945	4.3345E-07	1733.79945	2.29086E-05
1735.72803	0.00038133	1735.72803	2.57839E-07	1735.72803	1.69074E-05
1737.65662	0.00032531	1737.65662	1.51595E-07	1737.65662	1.23919E-05
1739.58521	0.00027655	1739.58521	8.80963E-08	1739.58521	9.01944E-06
1741.51379	0.00023427	1741.51379	5.06032E-08	1741.51379	6.51937E-06
1743.44238	0.00019776	1743.44238	2.87314E-08	1743.44238	4.67967E-06

1745.37097	0.00016635	1745.37097	1.61253E-08	1745.37097	3.33586E-06
1747.29955	0.00013943	1747.29955	8.94643E-09	1747.29955	2.36148E-06
1749.22814	0.00011646	1749.22814	4.90679E-09	1749.22814	1.66013E-06
1751.15673	9.6929E-05	1751.15673	2.66057E-09	1751.15673	0.000001159
1753.08531	8.0388E-05	1753.08531	1.42627E-09	1753.08531	8.03543E-07
1755.0139	6.6433E-05	1755.0139	7.55984E-10	1755.0139	5.53244E-07
1756.94249	5.4706E-05	1756.94249	3.96217E-10	1756.94249	3.78275E-07
1758.87107	0.00004489	1758.87107	2.05353E-10	1758.87107	2.56852E-07
1760.79966	3.6704E-05	1760.79966	1.05259E-10	1760.79966	1.73197E-07
1762.72825	2.9905E-05	1762.72825	5.33641E-11	1762.72825	1.15979E-07
1764.65683	2.4278E-05	1764.65683	2.67625E-11	1764.65683	7.71264E-08
1766.58542	1.9641E-05	1766.58542	1.32786E-11	1766.58542	5.09342E-08
1768.51401	1.5832E-05	1768.51401	6.51913E-12	1768.51401	3.3404E-08
1770.44259	1.2717E-05	1770.44259	3.1675E-12	1770.44259	2.17556E-08
1772.37118	1.0179E-05	1772.37118	1.52342E-12	1772.37118	1.40711E-08
1774.29977	8.1177E-06	1774.29977	7.25422E-13	1774.29977	9.03787E-09
1776.22835	6.4512E-06	1776.22835	3.42088E-13	1776.22835	5.76485E-09
1778.15694	5.1086E-06	1778.15694	1.59801E-13	1778.15694	3.65168E-09
1780.08553	4.031E-06	1780.08553	7.39677E-14	1780.08553	2.2971E-09
1782.01411	3.1694E-06	1782.01411	3.39367E-14	1782.01411	1.435E-09
1783.9427	2.4831E-06	1783.9427	1.54389E-14	1783.9427	8.90237E-10
1785.87129	1.9385E-06	1785.87129	6.96697E-15	1785.87129	5.48457E-10
1787.79987	1.508E-06	1787.79987	3.11982E-15	1787.79987	3.35554E-10
1789.72846	1.1689E-06	1789.72846	1.38693E-15	1789.72846	2.03876E-10
1791.65705	9.028E-07	1791.65705	6.12363E-16	1791.65705	1.23013E-10
1793.58563	6.9482E-07	1793.58563	2.68645E-16	1793.58563	7.37093E-11
1795.51422	5.3286E-07	1795.51422	1.17153E-16	1795.51422	4.38606E-11
1797.44281	4.0719E-07	1797.44281	5.08055E-17	1797.44281	2.59185E-11
1799.37139	3.1006E-07	1799.37139	2.19189E-17	1799.37139	1.521E-11

X Observed	Y Generated Coal+TPPI	X Observed	Y Generated Coal+PA
1000.9365	0.0214	1000.9365	0.01437
1002.86509	0.02246	1002.8651	0.01516
1004.79367	0.02349	1004.7937	0.01595
1006.72226	0.02449	1006.7223	0.01672
1008.65085	0.02545	1008.6509	0.01747
1010.57943	0.02635	1010.5794	0.01819
1012.50802	0.02721	1012.508	0.01888
1014.43661	0.02799	1014.4366	0.01954
1016.36519	0.02871	1016.3652	0.02016
1018.29378	0.02936	1018.2938	0.02073
1020.22237	0.02992	1020.2224	0.02125
1022.15095	0.0304	1022.151	0.02172
1024.07954	0.0308	1024.0795	0.02214
1026.00813	0.0311	1026.0081	0.0225
1027.93671	0.03132	1027.9367	0.02281
1029.8653	0.03146	1029.8653	0.02305
1031.79389	0.0315	1031.7939	0.02324
1033.72247	0.03146	1033.7225	0.02337
1035.65106	0.03135	1035.6511	0.02345
1037.57965	0.03115	1037.5797	0.02347
1039.50823	0.03089	1039.5082	0.02345
1041.43682	0.03056	1041.4368	0.02338
1043.36541	0.03018	1043.3654	0.02327
1045.29399	0.02975	1045.294	0.02312
1047.22258	0.02928	1047.2226	0.02294
1049.15117	0.02877	1049.1512	0.02274
1051.07975	0.02824	1051.0798	0.02252
1053.00834	0.0277	1053.0083	0.02229
1054.93693	0.02714	1054.9369	0.02204
1056.86551	0.02659	1056.8655	0.0218
1058.7941	0.02605	1058.7941	0.02156
1060.72269	0.02551	1060.7227	0.02132
1062.65127	0.025	1062.6513	0.0211
1064.57986	0.02452	1064.5799	0.02089
1066.50845	0.02406	1066.5085	0.0207
1068.43703	0.02364	1068.437	0.02053
1070.36562	0.02325	1070.3656	0.02039
1072.29421	0.0229	1072.2942	0.02027
1074.22279	0.02259	1074.2228	0.02017
1076.15138	0.02232	1076.1514	0.02009
1078.07997	0.02208	1078.08	0.02004
1080.00855	0.02188	1080.0086	0.02001
1081.93714	0.02171	1081.9371	0.01999
1083.86573	0.02157	1083.8657	0.01999
1085.79431	0.02145	1085.7943	0.02
1087.7229	0.02135	1087.7229	0.02001
1089.65149	0.02127	1089.6515	0.02004
1091.58007	0.02119	1091.5801	0.02006
1093.50866	0.02113	1093.5087	0.02007
1095.43725	0.02106	1095.4373	0.02008

1097.36584	0.02099	1097.3658	0.02007
1099.29442	0.02091	1099.2944	0.02005
1101.22301	0.02083	1101.223	0.02002
1103.1516	0.02072	1103.1516	0.01996
1105.08018	0.02061	1105.0802	0.01987
1107.00877	0.02047	1107.0088	0.01976
1108.93736	0.02031	1108.9374	0.01963
1110.86594	0.02013	1110.8659	0.01946
1112.79453	0.01993	1112.7945	0.01927
1114.72312	0.01971	1114.7231	0.01906
1116.6517	0.01947	1116.6517	0.01882
1118.58029	0.01921	1118.5803	0.01855
1120.50888	0.01893	1120.5089	0.01827
1122.43746	0.01865	1122.4375	0.01796
1124.36605	0.01835	1124.3661	0.01764
1126.29464	0.01805	1126.2946	0.01731
1128.22322	0.01774	1128.2232	0.01697
1130.15181	0.01744	1130.1518	0.01661
1132.0804	0.01714	1132.0804	0.01626
1134.00898	0.01684	1134.009	0.01591
1135.93757	0.01656	1135.9376	0.01556
1137.86616	0.01628	1137.8662	0.01521
1139.79474	0.01603	1139.7947	0.01487
1141.72333	0.01579	1141.7233	0.01454
1143.65192	0.01556	1143.6519	0.01422
1145.5805	0.01536	1145.5805	0.01392
1147.50909	0.01517	1147.5091	0.01363
1149.43768	0.015	1149.4377	0.01335
1151.36626	0.01485	1151.3663	0.01309
1153.29485	0.01472	1153.2949	0.01284
1155.22344	0.0146	1155.2234	0.0126
1157.15202	0.0145	1157.152	0.01237
1159.08061	0.01441	1159.0806	0.01216
1161.0092	0.01432	1161.0092	0.01195
1162.93778	0.01424	1162.9378	0.01175
1164.86637	0.01416	1164.8664	0.01156
1166.79496	0.01408	1166.795	0.01137
1168.72354	0.01399	1168.7235	0.01119
1170.65213	0.0139	1170.6521	0.011
1172.58072	0.01381	1172.5807	0.01081
1174.5093	0.0137	1174.5093	0.01062
1176.43789	0.01357	1176.4379	0.01043
1178.36648	0.01344	1178.3665	0.01023
1180.29506	0.01329	1180.2951	0.01003
1182.22365	0.01312	1182.2237	0.00982
1184.15224	0.01294	1184.1522	0.00961
1186.08082	0.01274	1186.0808	0.0094
1188.00941	0.01252	1188.0094	0.00918
1189.938	0.01229	1189.938	0.00895
1191.86658	0.01205	1191.8666	0.00873
1193.79517	0.0118	1193.7952	0.00851
1195.72376	0.01153	1195.7238	0.00828
1197.65234	0.01126	1197.6523	0.00806
1199.58093	0.01099	1199.5809	0.00784
1201.50952	0.01071	1201.5095	0.00763
1203.4381	0.01044	1203.4381	0.00743

1205.36669	0.01016	1205.3667	0.00724
1207.29528	0.00989	1207.2953	0.00705
1209.22386	0.00962	1209.2239	0.00688
1211.15245	0.00936	1211.1525	0.00672
1213.08104	0.00911	1213.081	0.00657
1215.00962	0.00887	1215.0096	0.00644
1216.93821	0.00865	1216.9382	0.00631
1218.8668	0.00843	1218.8668	0.00621
1220.79538	0.00823	1220.7954	0.00611
1222.72397	0.00804	1222.724	0.00602
1224.65256	0.00786	1224.6526	0.00595
1226.58114	0.00769	1226.5811	0.00588
1228.50973	0.00753	1228.5097	0.00582
1230.43832	0.00738	1230.4383	0.00577
1232.3669	0.00724	1232.3669	0.00572
1234.29549	0.00711	1234.2955	0.00568
1236.22408	0.00698	1236.2241	0.00563
1238.15266	0.00686	1238.1527	0.00559
1240.08125	0.00674	1240.0813	0.00554
1242.00984	0.00663	1242.0098	0.00549
1243.93842	0.00651	1243.9384	0.00544
1245.86701	0.0064	1245.867	0.00538
1247.7956	0.00628	1247.7956	0.00531
1249.72418	0.00617	1249.7242	0.00524
1251.65277	0.00605	1251.6528	0.00517
1253.58136	0.00593	1253.5814	0.00509
1255.50994	0.00582	1255.5099	0.005
1257.43853	0.0057	1257.4385	0.00491
1259.36712	0.00558	1259.3671	0.00483
1261.2957	0.00546	1261.2957	0.00474
1263.22429	0.00535	1263.2243	0.00465
1265.15288	0.00524	1265.1529	0.00456
1267.08146	0.00514	1267.0815	0.00448
1269.01005	0.00504	1269.0101	0.0044
1270.93864	0.00495	1270.9386	0.00433
1272.86722	0.00487	1272.8672	0.00428
1274.79581	0.0048	1274.7958	0.00423
1276.7244	0.00474	1276.7244	0.00419
1278.65298	0.00469	1278.653	0.00417
1280.58157	0.00466	1280.5816	0.00415
1282.51016	0.00464	1282.5102	0.00415
1284.43874	0.00463	1284.4387	0.00417
1286.36733	0.00463	1286.3673	0.00419
1288.29592	0.00465	1288.2959	0.00423
1290.22451	0.00468	1290.2245	0.00428
1292.15309	0.00472	1292.1531	0.00433
1294.08168	0.00477	1294.0817	0.00439
1296.01027	0.00483	1296.0103	0.00446
1297.93885	0.0049	1297.9389	0.00453
1299.86744	0.00497	1299.8674	0.00461
1301.79603	0.00505	1301.796	0.00468
1303.72461	0.00513	1303.7246	0.00476
1305.6532	0.00521	1305.6532	0.00483
1307.58179	0.00529	1307.5818	0.0049
1309.51037	0.00537	1309.5104	0.00497
1311.43896	0.00544	1311.439	0.00503

1313.36755	0.00552	1313.3676	0.00509
1315.29613	0.00559	1315.2961	0.00514
1317.22472	0.00565	1317.2247	0.00519
1319.15331	0.00572	1319.1533	0.00524
1321.08189	0.00578	1321.0819	0.00528
1323.01048	0.00584	1323.0105	0.00532
1324.93907	0.0059	1324.9391	0.00537
1326.86765	0.00596	1326.8677	0.00542
1328.79624	0.00603	1328.7962	0.00547
1330.72483	0.00609	1330.7248	0.00553
1332.65341	0.00617	1332.6534	0.0056
1334.582	0.00625	1334.582	0.00569
1336.51059	0.00635	1336.5106	0.00578
1338.43917	0.00645	1338.4392	0.00589
1340.36776	0.00656	1340.3678	0.00601
1342.29635	0.00669	1342.2964	0.00615
1344.22493	0.00684	1344.2249	0.00631
1346.15352	0.007	1346.1535	0.00648
1348.08211	0.00717	1348.0821	0.00667
1350.01069	0.00736	1350.0107	0.00688
1351.93928	0.00756	1351.9393	0.00711
1353.86787	0.00778	1353.8679	0.00735
1355.79645	0.00801	1355.7965	0.0076
1357.72504	0.00825	1357.725	0.00786
1359.65363	0.0085	1359.6536	0.00814
1361.58221	0.00876	1361.5822	0.00842
1363.5108	0.00903	1363.5108	0.00871
1365.43939	0.0093	1365.4394	0.009
1367.36797	0.00957	1367.368	0.00929
1369.29656	0.00985	1369.2966	0.00957
1371.22515	0.01012	1371.2252	0.00986
1373.15373	0.01039	1373.1537	0.01013
1375.08232	0.01066	1375.0823	0.0104
1377.01091	0.01092	1377.0109	0.01066
1378.93949	0.01118	1378.9395	0.01091
1380.86808	0.01142	1380.8681	0.01114
1382.79667	0.01166	1382.7967	0.01136
1384.72525	0.01189	1384.7253	0.01157
1386.65384	0.01211	1386.6538	0.01176
1388.58243	0.01232	1388.5824	0.01193
1390.51101	0.01252	1390.511	0.01209
1392.4396	0.01271	1392.4396	0.01223
1394.36819	0.01289	1394.3682	0.01236
1396.29677	0.01306	1396.2968	0.01248
1398.22536	0.01323	1398.2254	0.01258
1400.15395	0.01338	1400.154	0.01267
1402.08253	0.01353	1402.0825	0.01274
1404.01112	0.01367	1404.0111	0.01281
1405.93971	0.0138	1405.9397	0.01287
1407.86829	0.01393	1407.8683	0.01291
1409.79688	0.01405	1409.7969	0.01295
1411.72547	0.01417	1411.7255	0.01298
1413.65405	0.01428	1413.6541	0.013
1415.58264	0.01438	1415.5826	0.01302
1417.51123	0.01448	1417.5112	0.01303
1419.43981	0.01457	1419.4398	0.01303

1421.3684	0.01466	1421.3684	0.01303
1423.29699	0.01473	1423.297	0.01302
1425.22557	0.0148	1425.2256	0.013
1427.15416	0.01485	1427.1542	0.01298
1429.08275	0.0149	1429.0828	0.01294
1431.01133	0.01493	1431.0113	0.0129
1432.93992	0.01494	1432.9399	0.01284
1434.86851	0.01495	1434.8685	0.01277
1436.79709	0.01493	1436.7971	0.0127
1438.72568	0.0149	1438.7257	0.0126
1440.65427	0.01484	1440.6543	0.0125
1442.58285	0.01477	1442.5829	0.01237
1444.51144	0.01468	1444.5114	0.01224
1446.44003	0.01456	1446.44	0.01208
1448.36861	0.01442	1448.3686	0.01191
1450.2972	0.01425	1450.2972	0.01173
1452.22579	0.01407	1452.2258	0.01152
1454.15437	0.01386	1454.1544	0.0113
1456.08296	0.01362	1456.083	0.01106
1458.01155	0.01336	1458.0116	0.01081
1459.94013	0.01309	1459.9401	0.01054
1461.86872	0.01279	1461.8687	0.01026
1463.79731	0.01246	1463.7973	0.00996
1465.72589	0.01213	1465.7259	0.00966
1467.65448	0.01177	1467.6545	0.00934
1469.58307	0.0114	1469.5831	0.00901
1471.51165	0.01102	1471.5117	0.00868
1473.44024	0.01062	1473.4402	0.00834
1475.36883	0.01022	1475.3688	0.008
1477.29741	0.00981	1477.2974	0.00765
1479.226	0.0094	1479.226	0.00731
1481.15459	0.00898	1481.1546	0.00697
1483.08318	0.00857	1483.0832	0.00663
1485.01176	0.00816	1485.0118	0.00631
1486.94035	0.00775	1486.9404	0.00599
1488.86894	0.00736	1488.8689	0.00568
1490.79752	0.00697	1490.7975	0.00538
1492.72611	0.0066	1492.7261	0.0051
1494.6547	0.00624	1494.6547	0.00484
1496.58328	0.0059	1496.5833	0.00459
1498.51187	0.00558	1498.5119	0.00436
1500.44046	0.00529	1500.4405	0.00416
1502.36904	0.00501	1502.369	0.00397
1504.29763	0.00476	1504.2976	0.00382
1506.22622	0.00454	1506.2262	0.00368
1508.1548	0.00435	1508.1548	0.00357
1510.08339	0.00419	1510.0834	0.00349
1512.01198	0.00405	1512.012	0.00344
1513.94056	0.00395	1513.9406	0.00341
1515.86915	0.00388	1515.8692	0.00342
1517.79774	0.00385	1517.7977	0.00345
1519.72632	0.00385	1519.7263	0.00351
1521.65491	0.00388	1521.6549	0.0036
1523.5835	0.00395	1523.5835	0.00372
1525.51208	0.00406	1525.5121	0.00387
1527.44067	0.0042	1527.4407	0.00404

1529.36926	0.00437	1529.3693	0.00424
1531.29784	0.00458	1531.2978	0.00447
1533.22643	0.00483	1533.2264	0.00472
1535.15502	0.00511	1535.155	0.005
1537.0836	0.00542	1537.0836	0.0053
1539.01219	0.00577	1539.0122	0.00562
1540.94078	0.00614	1540.9408	0.00596
1542.86936	0.00655	1542.8694	0.00633
1544.79795	0.007	1544.798	0.0067
1546.72654	0.00747	1546.7265	0.0071
1548.65512	0.00797	1548.6551	0.00751
1550.58371	0.0085	1550.5837	0.00794
1552.5123	0.00906	1552.5123	0.00837
1554.44088	0.00965	1554.4409	0.00883
1556.36947	0.01027	1556.3695	0.00929
1558.29806	0.01092	1558.2981	0.00977
1560.22664	0.01159	1560.2266	0.01026
1562.15523	0.01229	1562.1552	0.01076
1564.08382	0.01302	1564.0838	0.01127
1566.0124	0.01377	1566.0124	0.0118
1567.94099	0.01454	1567.941	0.01234
1569.86958	0.01534	1569.8696	0.01289
1571.79816	0.01616	1571.7982	0.01346
1573.72675	0.017	1573.7268	0.01404
1575.65534	0.01785	1575.6553	0.01464
1577.58392	0.01872	1577.5839	0.01526
1579.51251	0.0196	1579.5125	0.01589
1581.4411	0.02048	1581.4411	0.01654
1583.36968	0.02136	1583.3697	0.01721
1585.29827	0.02223	1585.2983	0.01788
1587.22686	0.02309	1587.2269	0.01857
1589.15544	0.02394	1589.1554	0.01927
1591.08403	0.02475	1591.084	0.01997
1593.01262	0.02554	1593.0126	0.02067
1594.9412	0.02628	1594.9412	0.02137
1596.86979	0.02698	1596.8698	0.02206
1598.79838	0.02762	1598.7984	0.02274
1600.72696	0.02819	1600.727	0.0234
1602.65555	0.0287	1602.6556	0.02403
1604.58414	0.02913	1604.5841	0.02463
1606.51272	0.02949	1606.5127	0.02519
1608.44131	0.02976	1608.4413	0.0257
1610.3699	0.02994	1610.3699	0.02617
1612.29848	0.03003	1612.2985	0.02657
1614.22707	0.03003	1614.2271	0.02692
1616.15566	0.02994	1616.1557	0.0272
1618.08424	0.02977	1618.0842	0.02741
1620.01283	0.02951	1620.0128	0.02755
1621.94142	0.02916	1621.9414	0.02761
1623.87	0.02874	1623.87	0.0276
1625.79859	0.02825	1625.7986	0.02752
1627.72718	0.0277	1627.7272	0.02736
1629.65576	0.02708	1629.6558	0.02713
1631.58435	0.02642	1631.5844	0.02683
1633.51294	0.02571	1633.5129	0.02647
1635.44152	0.02496	1635.4415	0.02604

1637.37011	0.02418	1637.3701	0.02557
1639.2987	0.02339	1639.2987	0.02504
1641.22728	0.02257	1641.2273	0.02446
1643.15587	0.02175	1643.1559	0.02385
1645.08446	0.02092	1645.0845	0.0232
1647.01304	0.02009	1647.013	0.02252
1648.94163	0.01926	1648.9416	0.02181
1650.87022	0.01845	1650.8702	0.02109
1652.7988	0.01764	1652.7988	0.02036
1654.72739	0.01685	1654.7274	0.01961
1656.65598	0.01607	1656.656	0.01886
1658.58456	0.01531	1658.5846	0.01811
1660.51315	0.01456	1660.5132	0.01735
1662.44174	0.01384	1662.4417	0.0166
1664.37032	0.01313	1664.3703	0.01585
1666.29891	0.01245	1666.2989	0.01512
1668.2275	0.01178	1668.2275	0.01439
1670.15608	0.01113	1670.1561	0.01367
1672.08467	0.01049	1672.0847	0.01296
1674.01326	0.00988	1674.0133	0.01227
1675.94185	0.00929	1675.9419	0.01159
1677.87043	0.00871	1677.8704	0.01093
1679.79902	0.00816	1679.799	0.01028
1681.72761	0.00762	1681.7276	0.00965
1683.65619	0.0071	1683.6562	0.00904
1685.58478	0.0066	1685.5848	0.00845
1687.51337	0.00612	1687.5134	0.00787
1689.44195	0.00567	1689.442	0.00732
1691.37054	0.00523	1691.3705	0.00679
1693.29913	0.00481	1693.2991	0.00628
1695.22771	0.00441	1695.2277	0.00579
1697.1563	0.00404	1697.1563	0.00533
1699.08489	0.00368	1699.0849	0.00488
1701.01347	0.00335	1701.0135	0.00447
1702.94206	0.00304	1702.9421	0.00407
1704.87065	0.00274	1704.8707	0.0037
1706.79923	0.00247	1706.7992	0.00335
1708.72782	0.00222	1708.7278	0.00303
1710.65641	0.00198	1710.6564	0.00272
1712.58499	0.00177	1712.585	0.00244
1714.51358	0.00157	1714.5136	0.00218
1716.44217	0.00139	1716.4422	0.00194
1718.37075	0.00123	1718.3708	0.00173
1720.29934	0.00108	1720.2993	0.00153
1722.22793	0.00094875	1722.2279	0.00135
1724.15651	0.00082888	1724.1565	0.00118
1726.0851	0.00072163	1726.0851	0.00104
1728.01369	0.00062606	1728.0137	0.00090424
1729.94227	0.00054123	1729.9423	0.00078612
1731.87086	0.00046625	1731.8709	0.00068098
1733.79945	0.00040024	1733.7995	0.0005878
1735.72803	0.00034235	1735.728	0.00050554
1737.65662	0.0002918	1737.6566	0.00043322
1739.58521	0.00024783	1739.5852	0.0003699
1741.51379	0.00020974	1741.5138	0.0003147
1743.44238	0.00017687	1743.4424	0.00026676

1745.37097	0.00014862	1745.371	0.0002253
1747.29955	0.00012443	1747.2996	0.00018959
1749.22814	0.00010381	1749.2281	0.00015896
1751.15673	8.6298E-05	1751.1567	0.00013279
1753.08531	7.1483E-05	1753.0853	0.00011052
1755.0139	5.8999E-05	1755.0139	9.1655E-05
1756.94249	4.8522E-05	1756.9425	7.5729E-05
1758.87107	3.9762E-05	1758.8711	6.234E-05
1760.79966	3.2467E-05	1760.7997	5.1131E-05
1762.72825	2.6415E-05	1762.7283	4.1783E-05
1764.65683	2.1415E-05	1764.6568	3.4019E-05
1766.58542	1.7299E-05	1766.5854	2.7596E-05
1768.51401	1.3924E-05	1768.514	2.2304E-05
1770.44259	1.1167E-05	1770.4426	1.796E-05
1772.37118	8.9239E-06	1772.3712	1.4409E-05
1774.29977	7.1058E-06	1774.2998	1.1518E-05
1776.22835	5.6379E-06	1776.2284	9.1729E-06
1778.15694	4.4572E-06	1778.1569	7.2785E-06
1780.08553	3.5111E-06	1780.0855	5.7541E-06
1782.01411	2.7559E-06	1782.0141	4.5323E-06
1783.9427	2.1554E-06	1783.9427	3.5567E-06
1785.87129	1.6797E-06	1785.8713	2.7809E-06
1787.79987	1.3043E-06	1787.7999	2.1663E-06
1789.72846	1.0092E-06	1789.7285	1.6814E-06
1791.65705	7.7803E-07	1791.6571	1.3002E-06
1793.58563	5.9767E-07	1793.5856	1.0017E-06
1795.51422	4.5748E-07	1795.5142	7.6892E-07
1797.44281	3.4891E-07	1797.4428	5.8806E-07
1799.37139	2.6516E-07	1799.3714	4.4808E-07

Fitting results of IR spectra of water-immersed coking coal with inhibitor

**2800-3000**	X Observed	Y Generated Coal	X Observed	Y Generated Coal+MgCl2	X Observed	Y Generated Coal+TEMPO
	2800.3079	0.00085983	2800.3079	0.000459642	2800.3079	0.000440872
	2802.23649	0.00101	2802.23649	0.000563611	2802.23649	0.000527892
	2804.16507	0.00117	2804.16507	0.000681758	2804.16507	0.000624457
	2806.09366	0.00134	2806.09366	0.000813835	2806.09366	0.000729893
	2808.02225	0.00152	2808.02225	0.000959196	2808.02225	0.000843188
	2809.95083	0.00171	2809.95083	0.00112	2809.95083	0.000963065
	2811.87942	0.00191	2811.87942	0.00129	2811.87942	0.00109
	2813.80801	0.00211	2813.80801	0.00146	2813.80801	0.00122
	2815.73659	0.00232	2815.73659	0.00165	2815.73659	0.00135
	2817.66518	0.00254	2817.66518	0.00185	2817.66518	0.00148
	2819.59377	0.00276	2819.59377	0.00206	2819.59377	0.00162
	2821.52235	0.00299	2821.52235	0.00227	2821.52235	0.00176
	2823.45094	0.00324	2823.45094	0.0025	2823.45094	0.00191
	2825.37953	0.00351	2825.37953	0.00275	2825.37953	0.00208
	2827.30811	0.00382	2827.30811	0.00302	2827.30811	0.00226
	2829.2367	0.00416	2829.2367	0.00331	2829.2367	0.00247
	2831.16529	0.00455	2831.16529	0.00363	2831.16529	0.00272
	2833.09387	0.00499	2833.09387	0.00398	2833.09387	0.00301
	2835.02246	0.00549	2835.02246	0.00436	2835.02246	0.00335
	2836.95105	0.00605	2836.95105	0.00476	2836.95105	0.00373
	2838.87963	0.00666	2838.87963	0.00519	2838.87963	0.00415
	2840.80822	0.00731	2840.80822	0.00563	2840.80822	0.00461
	2842.73681	0.00798	2842.73681	0.00607	2842.73681	0.0051
	2844.66539	0.00864	2844.66539	0.0065	2844.66539	0.00558
	2846.59398	0.00926	2846.59398	0.00689	2846.59398	0.00605
	2848.52257	0.00982	2848.52257	0.00724	2848.52257	0.00646
	2850.45115	0.01029	2850.45115	0.00753	2850.45115	0.00682
	2852.37974	0.01064	2852.37974	0.00775	2852.37974	0.00708
	2854.30833	0.01085	2854.30833	0.00789	2854.30833	0.00725
	2856.23691	0.01092	2856.23691	0.00795	2856.23691	0.00732
	2858.1655	0.01086	2858.1655	0.00793	2858.1655	0.00728
	2860.09409	0.01067	2860.09409	0.00785	2860.09409	0.00716
	2862.02267	0.01039	2862.02267	0.0077	2862.02267	0.00696
	2863.95126	0.01004	2863.95126	0.00751	2863.95126	0.00672
	2865.87985	0.00965	2865.87985	0.0073	2865.87985	0.00646
	2867.80843	0.00927	2867.80843	0.00708	2867.80843	0.0062
	2869.73702	0.00891	2869.73702	0.00688	2869.73702	0.00596
	2871.66561	0.00862	2871.66561	0.0067	2871.66561	0.00576
	2873.59419	0.0084	2873.59419	0.00656	2873.59419	0.00562
	2875.52278	0.00827	2875.52278	0.00646	2875.52278	0.00554
	2877.45137	0.00823	2877.45137	0.00643	2877.45137	0.00551
	2879.37995	0.00827	2879.37995	0.00644	2879.37995	0.00555
	2881.30854	0.0084	2881.30854	0.00651	2881.30854	0.00564
	2883.23713	0.00858	2883.23713	0.00663	2883.23713	0.00577
	2885.16571	0.00882	2885.16571	0.00679	2885.16571	0.00593
	2887.0943	0.00909	2887.0943	0.00699	2887.0943	0.00611
	2889.02289	0.0094	2889.02289	0.00722	2889.02289	0.00631
	2890.95147	0.00972	2890.95147	0.00749	2890.95147	0.00653
	2892.88006	0.01007	2892.88006	0.00778	2892.88006	0.00675
	2894.80865	0.01044	2894.80865	0.0081	2894.80865	0.00698

2896.73724	0.01084	2896.73724	0.00844	2896.73724	0.00723
2898.66582	0.01128	2898.66582	0.00881	2898.66582	0.0075
2900.59441	0.01177	2900.59441	0.00921	2900.59441	0.0078
2902.523	0.01233	2902.523	0.00963	2902.523	0.00814
2904.45158	0.01295	2904.45158	0.01007	2904.45158	0.00853
2906.38017	0.01363	2906.38017	0.01052	2906.38017	0.00896
2908.30876	0.01436	2908.30876	0.01097	2908.30876	0.00942
2910.23734	0.01512	2910.23734	0.01141	2910.23734	0.00991
2912.16593	0.01587	2912.16593	0.01182	2912.16593	0.01041
2914.09452	0.01658	2914.09452	0.01217	2914.09452	0.01089
2916.0231	0.0172	2916.0231	0.01244	2916.0231	0.01133
2917.95169	0.01768	2917.95169	0.01262	2917.95169	0.01168
2919.88028	0.01798	2919.88028	0.01268	2919.88028	0.01193
2921.80886	0.01806	2921.80886	0.0126	2921.80886	0.01205
2923.73745	0.0179	2923.73745	0.0124	2923.73745	0.01201
2925.66604	0.0175	2925.66604	0.01206	2925.66604	0.01182
2927.59462	0.01687	2927.59462	0.01159	2927.59462	0.01147
2929.52321	0.01606	2929.52321	0.01103	2929.52321	0.01098
2931.4518	0.01511	2931.4518	0.0104	2931.4518	0.01038
2933.38038	0.01408	2933.38038	0.00973	2933.38038	0.00972
2935.30897	0.01305	2935.30897	0.00907	2935.30897	0.00902
2937.23756	0.0121	2937.23756	0.00847	2937.23756	0.00835
2939.16614	0.01127	2939.16614	0.00796	2939.16614	0.00774
2941.09473	0.01062	2941.09473	0.00756	2941.09473	0.00723
2943.02332	0.01016	2943.02332	0.00729	2943.02332	0.00685
2944.9519	0.00989	2944.9519	0.00714	2944.9519	0.00661
2946.88049	0.00977	2946.88049	0.00709	2946.88049	0.00649
2948.80908	0.00976	2948.80908	0.00709	2948.80908	0.00647
2950.73766	0.00977	2950.73766	0.00709	2950.73766	0.00651
2952.66625	0.00974	2952.66625	0.00704	2952.66625	0.00654
2954.59484	0.00961	2954.59484	0.0069	2954.59484	0.00652
2956.52342	0.00933	2956.52342	0.00662	2956.52342	0.0064
2958.45201	0.00886	2958.45201	0.0062	2958.45201	0.00615
2960.3806	0.00822	2960.3806	0.00565	2960.3806	0.00577
2962.30918	0.00742	2962.30918	0.005	2962.30918	0.00526
2964.23777	0.00652	2964.23777	0.00428	2964.23777	0.00465
2966.16636	0.00555	2966.16636	0.00354	2966.16636	0.00398
2968.09494	0.00459	2968.09494	0.00283	2968.09494	0.0033
2970.02353	0.00368	2970.02353	0.00219	2970.02353	0.00264
2971.95212	0.00286	2971.95212	0.00163	2971.95212	0.00204
2973.8807	0.00216	2973.8807	0.00117	2973.8807	0.00153
2975.80929	0.00157	2975.80929	0.000815018	2975.80929	0.0011
2977.73788	0.00111	2977.73788	0.000545644	2977.73788	0.000767576
2979.66646	0.00076135	2979.66646	0.000352306	2979.66646	0.000516009
2981.59505	0.00050472	2981.59505	0.000219366	2981.59505	0.000334784
2983.52364	0.00032402	2983.52364	0.000131718	2983.52364	0.000209609
2985.45222	0.00020143	2985.45222	7.62692E-05	2985.45222	0.00012664
2987.38081	0.00012125	2987.38081	4.25889E-05	2987.38081	7.38303E-05
2989.3094	7.0677E-05	2989.3094	2.29361E-05	2989.3094	4.15328E-05
2991.23798	0.00003989	2991.23798	1.19144E-05	2991.23798	2.25442E-05
2993.16657	2.18E-05	2993.16657	5.9707E-06	2993.16657	1.18077E-05
2995.09516	1.1535E-05	2995.09516	2.8873E-06	2995.09516	5.9675E-06
2997.02374	5.91E-06	2997.02374	1.3478E-06	2997.02374	2.91019E-06
2998.95233	2.9318E-06	2998.95233	6.07628E-07	2998.95233	1.36952E-06

X Observed	Y Generated Coal+TPPI	X Observed	Y Generated Coal+PA
2800.3079	0.00064696	2800.3079	0.00036196
2802.23649	0.00075806	2802.2365	0.00042812
2804.16507	0.00087919	2804.1651	0.00050083
2806.09366	0.00101	2806.0937	0.00057956
2808.02225	0.00115	2808.0223	0.00066347
2809.95083	0.0013	2809.9508	0.00075156
2811.87942	0.00145	2811.8794	0.00084268
2813.80801	0.00162	2813.808	0.00093565
2815.73659	0.00179	2815.7366	0.00103
2817.66518	0.00198	2817.6652	0.00112
2819.59377	0.00218	2819.5938	0.00122
2821.52235	0.0024	2821.5224	0.00131
2823.45094	0.00264	2823.4509	0.00141
2825.37953	0.0029	2825.3795	0.00152
2827.30811	0.00319	2827.3081	0.00164
2829.2367	0.00351	2829.2367	0.00177
2831.16529	0.00386	2831.1653	0.00193
2833.09387	0.00424	2833.0939	0.00212
2835.02246	0.00464	2835.0225	0.00234
2836.95105	0.00506	2836.9511	0.0026
2838.87963	0.00549	2838.8796	0.0029
2840.80822	0.0059	2840.8082	0.00322
2842.73681	0.0063	2842.7368	0.00356
2844.66539	0.00666	2844.6654	0.00389
2846.59398	0.00698	2846.594	0.00422
2848.52257	0.00723	2848.5226	0.0045
2850.45115	0.00742	2850.4512	0.00474
2852.37974	0.00754	2852.3797	0.00491
2854.30833	0.0076	2854.3083	0.00501
2856.23691	0.00759	2856.2369	0.00505
2858.1655	0.00753	2858.1655	0.00502
2860.09409	0.00743	2860.0941	0.00494
2862.02267	0.0073	2862.0227	0.00483
2863.95126	0.00716	2863.9513	0.0047
2865.87985	0.00701	2865.8799	0.00458
2867.80843	0.00687	2867.8084	0.00447
2869.73702	0.00675	2869.737	0.00437
2871.66561	0.00665	2871.6656	0.0043
2873.59419	0.00657	2873.5942	0.00425
2875.52278	0.00652	2875.5228	0.00421
2877.45137	0.0065	2877.4514	0.00419
2879.37995	0.00652	2879.38	0.00419
2881.30854	0.00657	2881.3085	0.00421
2883.23713	0.00666	2883.2371	0.00424
2885.16571	0.0068	2885.1657	0.00431
2887.0943	0.00697	2887.0943	0.0044
2889.02289	0.00719	2889.0229	0.00453
2890.95147	0.00745	2890.9515	0.00469
2892.88006	0.00776	2892.8801	0.00487
2894.80865	0.00809	2894.8087	0.00508

2896.73724	0.00846	2896.7372	0.00529
2898.66582	0.00885	2898.6658	0.0055
2900.59441	0.00926	2900.5944	0.00571
2902.523	0.00966	2902.523	0.00593
2904.45158	0.01007	2904.4516	0.00614
2906.38017	0.01046	2906.3802	0.00637
2908.30876	0.01082	2908.3088	0.00662
2910.23734	0.01114	2910.2373	0.00689
2912.16593	0.01141	2912.1659	0.00718
2914.09452	0.01163	2914.0945	0.00747
2916.0231	0.01177	2916.0231	0.00774
2917.95169	0.01183	2917.9517	0.00797
2919.88028	0.0118	2919.8803	0.00812
2921.80886	0.01169	2921.8089	0.00816
2923.73745	0.01149	2923.7375	0.00809
2925.66604	0.0112	2925.666	0.00788
2927.59462	0.01084	2927.5946	0.00756
2929.52321	0.01042	2929.5232	0.00714
2931.4518	0.00996	2931.4518	0.00667
2933.38038	0.00947	2933.3804	0.00619
2935.30897	0.00898	2935.309	0.00573
2937.23756	0.00851	2937.2376	0.00534
2939.16614	0.00807	2939.1661	0.00503
2941.09473	0.00768	2941.0947	0.00483
2943.02332	0.00733	2943.0233	0.00472
2944.9519	0.00704	2944.9519	0.00468
2946.88049	0.00679	2946.8805	0.00471
2948.80908	0.00657	2948.8091	0.00475
2950.73766	0.00635	2950.7377	0.00478
2952.66625	0.00613	2952.6663	0.00477
2954.59484	0.00588	2954.5948	0.00469
2956.52342	0.00559	2956.5234	0.00455
2958.45201	0.00525	2958.452	0.00432
2960.3806	0.00486	2960.3806	0.00401
2962.30918	0.00443	2962.3092	0.00365
2964.23777	0.00396	2964.2378	0.00324
2966.16636	0.00347	2966.1664	0.00282
2968.09494	0.00298	2968.0949	0.00239
2970.02353	0.0025	2970.0235	0.00198
2971.95212	0.00205	2971.9521	0.0016
2973.8807	0.00165	2973.8807	0.00126
2975.80929	0.00129	2975.8093	0.00096779
2977.73788	0.00098752	2977.7379	0.00072673
2979.66646	0.00073767	2979.6665	0.00053258
2981.59505	0.00053783	2981.5951	0.0003809
2983.52364	0.00038267	2983.5236	0.00026586
2985.45222	0.00026567	2985.4522	0.00018109
2987.38081	0.00017996	2987.3808	0.00012037
2989.3094	0.00011893	2989.3094	7.8087E-05
2991.23798	7.6673E-05	2991.238	4.9435E-05
2993.16657	4.8223E-05	2993.1666	3.0541E-05
2995.09516	2.9587E-05	2995.0952	1.8414E-05
2997.02374	1.7709E-05	2997.0237	1.0835E-05
2998.95233	1.034E-05	2998.9523	6.2213E-06

Fitting results of IR spectra of water-immersed coking coal with inhibitor

3000-3600	X Observed	Y Generated	X Observed	Y Generated	X Observed	Y Generated
		Raw coal + distilled water		Coal+MgCl2		Coal+TEMPO
	3000.8809	2.2284E-07	3000.8809	0.000211947	3000.8809	6.76328E-07
	3002.80949	2.7907E-07	3002.80949	0.000232803	3002.80949	8.26428E-07
	3004.73807	3.4855E-07	3004.73807	0.000255422	3004.73807	1.00744E-06
	3006.66666	4.3414E-07	3006.66666	0.00027992	3006.66666	1.22518E-06
	3008.59525	5.3928E-07	3008.59525	0.000306421	3008.59525	1.48643E-06
	3010.52383	6.6807E-07	3010.52383	0.000335051	3010.52383	1.79911E-06
	3012.45242	8.2538E-07	3012.45242	0.00036594	3012.45242	2.17238E-06
	3014.38101	1.017E-06	3014.38101	0.000399224	3014.38101	2.61687E-06
	3016.30959	1.2496E-06	3016.30959	0.000435042	3016.30959	3.14482E-06
	3018.23818	1.5314E-06	3018.23818	0.000473536	3018.23818	3.7703E-06
	3020.16677	1.8715E-06	3020.16677	0.000514852	3020.16677	4.50946E-06
	3022.09535	2.2811E-06	3022.09535	0.000559138	3022.09535	5.38073E-06
	3024.02394	2.7727E-06	3024.02394	0.000606545	3024.02394	6.4051E-06
	3025.95253	3.3612E-06	3025.95253	0.000657227	3025.95253	7.60642E-06
	3027.88111	4.0636E-06	3027.88111	0.000711336	3027.88111	9.01163E-06
	3029.8097	4.8995E-06	3029.8097	0.000769027	3029.8097	1.06511E-05
	3031.73829	5.8914E-06	3031.73829	0.000830455	3031.73829	1.25591E-05
	3033.66687	7.065E-06	3033.66687	0.000895774	3033.66687	1.47738E-05
	3035.59546	8.4496E-06	3035.59546	0.000965135	3035.59546	0.000017338
	3037.52405	1.0078E-05	3037.52405	0.00104	3037.52405	2.02991E-05
	3039.45263	1.1989E-05	3039.45263	0.00112	3039.45263	2.37097E-05
	3041.38122	1.4223E-05	3041.38122	0.0012	3041.38122	0.000027628
	3043.30981	1.6827E-05	3043.30981	0.00129	3043.30981	3.21179E-05
	3045.23839	1.9856E-05	3045.23839	0.00138	3045.23839	3.72495E-05
	3047.16698	2.3367E-05	3047.16698	0.00147	3047.16698	4.30992E-05
	3049.09557	2.7425E-05	3049.09557	0.00158	3049.09557	4.97503E-05
	3051.02415	3.2101E-05	3051.02415	0.00168	3051.02415	5.72928E-05
	3052.95274	3.7474E-05	3052.95274	0.00179	3052.95274	0.000065824
	3054.88133	4.363E-05	3054.88133	0.00191	3054.88133	7.54482E-05
	3056.80991	5.0662E-05	3056.80991	0.00203	3056.80991	8.62772E-05
	3058.7385	5.867E-05	3058.7385	0.00216	3058.7385	9.84298E-05
	3060.66709	6.7764E-05	3060.66709	0.0023	3060.66709	0.000112032
	3062.59567	7.8061E-05	3062.59567	0.00243	3062.59567	0.000127217
	3064.52426	8.9683E-05	3064.52426	0.00258	3064.52426	0.000144125
	3066.45285	0.00010276	3066.45285	0.00273	3066.45285	0.0001629
	3068.38143	0.00011744	3068.38143	0.00288	3068.38143	0.000183695
	3070.31002	0.00013386	3070.31002	0.00304	3070.31002	0.000206667
	3072.23861	0.00015218	3072.23861	0.00321	3072.23861	0.000231975
	3074.16719	0.00017256	3074.16719	0.00338	3074.16719	0.000259785
	3076.09578	0.00019515	3076.09578	0.00356	3076.09578	0.000290262
	3078.02437	0.00022012	3078.02437	0.00374	3078.02437	0.000323575
	3079.95295	0.00024766	3079.95295	0.00393	3079.95295	0.000359891
	3081.88154	0.00027791	3081.88154	0.00412	3081.88154	0.000399376
	3083.81013	0.00031107	3083.81013	0.00431	3083.81013	0.000442194
	3085.73871	0.00034729	3085.73871	0.00451	3085.73871	0.000488502
	3087.6673	0.00038674	3087.6673	0.00471	3087.6673	0.000538455
	3089.59589	0.00042959	3089.59589	0.00492	3089.59589	0.000592197
	3091.52447	0.00047599	3091.52447	0.00513	3091.52447	0.000649865
	3093.45306	0.00052608	3093.45306	0.00535	3093.45306	0.000711582
	3095.38165	0.00058001	3095.38165	0.00556	3095.38165	0.000777462

3097.31024	0.0006379	3097.31024	0.00578	3097.31024	0.000847603
3099.23882	0.00069985	3099.23882	0.00601	3099.23882	0.000922089
3101.16741	0.00076597	3101.16741	0.00623	3101.16741	0.001
3103.096	0.00083634	3103.096	0.00646	3103.096	0.00108
3105.02458	0.00091101	3105.02458	0.00668	3105.02458	0.00117
3106.95317	0.00099002	3106.95317	0.00691	3106.95317	0.00126
3108.88176	0.00107	3108.88176	0.00714	3108.88176	0.00136
3110.81034	0.00116	3110.81034	0.00737	3110.81034	0.00146
3112.73893	0.00125	3112.73893	0.0076	3112.73893	0.00157
3114.66752	0.00135	3114.66752	0.00784	3114.66752	0.00168
3116.5961	0.00145	3116.5961	0.00807	3116.5961	0.00179
3118.52469	0.00156	3118.52469	0.0083	3118.52469	0.00191
3120.45328	0.00166	3120.45328	0.00853	3120.45328	0.00203
3122.38186	0.00178	3122.38186	0.00877	3122.38186	0.00216
3124.31045	0.00189	3124.31045	0.009	3124.31045	0.00229
3126.23904	0.00202	3126.23904	0.00923	3126.23904	0.00242
3128.16762	0.00214	3128.16762	0.00947	3128.16762	0.00256
3130.09621	0.00227	3130.09621	0.0097	3130.09621	0.0027
3132.0248	0.0024	3132.0248	0.00994	3132.0248	0.00285
3133.95338	0.00253	3133.95338	0.01019	3133.95338	0.00299
3135.88197	0.00267	3135.88197	0.01043	3135.88197	0.00314
3137.81056	0.00281	3137.81056	0.01068	3137.81056	0.0033
3139.73914	0.00296	3139.73914	0.01094	3139.73914	0.00345
3141.66773	0.0031	3141.66773	0.0112	3141.66773	0.00361
3143.59632	0.00325	3143.59632	0.01147	3143.59632	0.00377
3145.5249	0.0034	3145.5249	0.01175	3145.5249	0.00393
3147.45349	0.00356	3147.45349	0.01204	3147.45349	0.0041
3149.38208	0.00372	3149.38208	0.01235	3149.38208	0.00426
3151.31066	0.00388	3151.31066	0.01267	3151.31066	0.00443
3153.23925	0.00404	3153.23925	0.013	3153.23925	0.0046
3155.16784	0.00421	3155.16784	0.01335	3155.16784	0.00478
3157.09642	0.00438	3157.09642	0.01372	3157.09642	0.00495
3159.02501	0.00455	3159.02501	0.01412	3159.02501	0.00513
3160.9536	0.00472	3160.9536	0.01453	3160.9536	0.00531
3162.88218	0.0049	3162.88218	0.01497	3162.88218	0.0055
3164.81077	0.00509	3164.81077	0.01544	3164.81077	0.00568
3166.73936	0.00527	3166.73936	0.01594	3166.73936	0.00587
3168.66794	0.00546	3168.66794	0.01646	3168.66794	0.00606
3170.59653	0.00566	3170.59653	0.01702	3170.59653	0.00626
3172.52512	0.00586	3172.52512	0.0176	3172.52512	0.00646
3174.4537	0.00606	3174.4537	0.01822	3174.4537	0.00666
3176.38229	0.00627	3176.38229	0.01887	3176.38229	0.00687
3178.31088	0.00649	3178.31088	0.01955	3178.31088	0.00708
3180.23946	0.0067	3180.23946	0.02027	3180.23946	0.00729
3182.16805	0.00693	3182.16805	0.02101	3182.16805	0.00751
3184.09664	0.00715	3184.09664	0.02178	3184.09664	0.00773
3186.02522	0.00739	3186.02522	0.02259	3186.02522	0.00795
3187.95381	0.00762	3187.95381	0.02341	3187.95381	0.00818
3189.8824	0.00787	3189.8824	0.02427	3189.8824	0.00841
3191.81098	0.00811	3191.81098	0.02514	3191.81098	0.00865
3193.73957	0.00836	3193.73957	0.02603	3193.73957	0.00889
3195.66816	0.00861	3195.66816	0.02694	3195.66816	0.00913
3197.59674	0.00887	3197.59674	0.02786	3197.59674	0.00937
3199.52533	0.00913	3199.52533	0.02878	3199.52533	0.00962
3201.45392	0.00939	3201.45392	0.02971	3201.45392	0.00987
3203.3825	0.00966	3203.3825	0.03063	3203.3825	0.01012

3205.31109	0.00993	3205.31109	0.03156	3205.31109	0.01038
3207.23968	0.01019	3207.23968	0.03247	3207.23968	0.01063
3209.16826	0.01046	3209.16826	0.03336	3209.16826	0.01089
3211.09685	0.01073	3211.09685	0.03424	3211.09685	0.01114
3213.02544	0.011	3213.02544	0.03509	3213.02544	0.0114
3214.95402	0.01127	3214.95402	0.03592	3214.95402	0.01166
3216.88261	0.01154	3216.88261	0.03672	3216.88261	0.01191
3218.8112	0.01181	3218.8112	0.03748	3218.8112	0.01217
3220.73978	0.01207	3220.73978	0.03821	3220.73978	0.01243
3222.66837	0.01234	3222.66837	0.0389	3222.66837	0.01268
3224.59696	0.0126	3224.59696	0.03956	3224.59696	0.01293
3226.52554	0.01286	3226.52554	0.04017	3226.52554	0.01318
3228.45413	0.01312	3228.45413	0.04075	3228.45413	0.01343
3230.38272	0.01338	3230.38272	0.04128	3230.38272	0.01368
3232.3113	0.01363	3232.3113	0.04177	3232.3113	0.01393
3234.23989	0.01389	3234.23989	0.04223	3234.23989	0.01417
3236.16848	0.01414	3236.16848	0.04266	3236.16848	0.01442
3238.09706	0.01439	3238.09706	0.04305	3238.09706	0.01466
3240.02565	0.01464	3240.02565	0.04341	3240.02565	0.0149
3241.95424	0.01489	3241.95424	0.04375	3241.95424	0.01514
3243.88282	0.01514	3243.88282	0.04407	3243.88282	0.01538
3245.81141	0.01539	3245.81141	0.04437	3245.81141	0.01562
3247.74	0.01564	3247.74	0.04466	3247.74	0.01586
3249.66858	0.0159	3249.66858	0.04494	3249.66858	0.0161
3251.59717	0.01615	3251.59717	0.04522	3251.59717	0.01634
3253.52576	0.01641	3253.52576	0.04551	3253.52576	0.01658
3255.45434	0.01667	3255.45434	0.0458	3255.45434	0.01682
3257.38293	0.01694	3257.38293	0.04611	3257.38293	0.01707
3259.31152	0.01721	3259.31152	0.04644	3259.31152	0.01732
3261.2401	0.01749	3261.2401	0.04679	3261.2401	0.01757
3263.16869	0.01777	3263.16869	0.04716	3263.16869	0.01783
3265.09728	0.01806	3265.09728	0.04757	3265.09728	0.01809
3267.02586	0.01836	3267.02586	0.04802	3267.02586	0.01835
3268.95445	0.01866	3268.95445	0.0485	3268.95445	0.01862
3270.88304	0.01897	3270.88304	0.04902	3270.88304	0.0189
3272.81162	0.01929	3272.81162	0.04959	3272.81162	0.01918
3274.74021	0.01962	3274.74021	0.0502	3274.74021	0.01947
3276.6688	0.01996	3276.6688	0.05086	3276.6688	0.01976
3278.59738	0.02031	3278.59738	0.05156	3278.59738	0.02006
3280.52597	0.02067	3280.52597	0.05231	3280.52597	0.02037
3282.45456	0.02104	3282.45456	0.05311	3282.45456	0.02069
3284.38314	0.02141	3284.38314	0.05395	3284.38314	0.02101
3286.31173	0.0218	3286.31173	0.05485	3286.31173	0.02135
3288.24032	0.0222	3288.24032	0.05579	3288.24032	0.02169
3290.16891	0.02261	3290.16891	0.05677	3290.16891	0.02204
3292.09749	0.02303	3292.09749	0.0578	3292.09749	0.0224
3294.02608	0.02346	3294.02608	0.05886	3294.02608	0.02277
3295.95467	0.0239	3295.95467	0.05997	3295.95467	0.02315
3297.88325	0.02435	3297.88325	0.06112	3297.88325	0.02354
3299.81184	0.02481	3299.81184	0.06231	3299.81184	0.02394
3301.74043	0.02529	3301.74043	0.06354	3301.74043	0.02436
3303.66901	0.02577	3303.66901	0.0648	3303.66901	0.02478
3305.5976	0.02627	3305.5976	0.06609	3305.5976	0.02522
3307.52619	0.02678	3307.52619	0.06742	3307.52619	0.02567
3309.45477	0.0273	3309.45477	0.06879	3309.45477	0.02613
3311.38336	0.02783	3311.38336	0.07018	3311.38336	0.02661

3313.31195	0.02838	3313.31195	0.07161	3313.31195	0.02711
3315.24053	0.02894	3315.24053	0.07306	3315.24053	0.02762
3317.16912	0.02951	3317.16912	0.07455	3317.16912	0.02814
3319.09771	0.03009	3319.09771	0.07608	3319.09771	0.02868
3321.02629	0.03069	3321.02629	0.07763	3321.02629	0.02924
3322.95488	0.0313	3322.95488	0.07922	3322.95488	0.02982
3324.88347	0.03193	3324.88347	0.08084	3324.88347	0.03041
3326.81205	0.03257	3326.81205	0.08249	3326.81205	0.03103
3328.74064	0.03323	3328.74064	0.08418	3328.74064	0.03166
3330.66923	0.0339	3330.66923	0.0859	3330.66923	0.03231
3332.59781	0.03459	3332.59781	0.08766	3332.59781	0.03298
3334.5264	0.03529	3334.5264	0.08945	3334.5264	0.03368
3336.45499	0.03601	3336.45499	0.09128	3336.45499	0.03439
3338.38357	0.03674	3338.38357	0.09315	3338.38357	0.03512
3340.31216	0.03749	3340.31216	0.09505	3340.31216	0.03588
3342.24075	0.03825	3342.24075	0.09699	3342.24075	0.03666
3344.16933	0.03903	3344.16933	0.09896	3344.16933	0.03745
3346.09792	0.03983	3346.09792	0.10097	3346.09792	0.03827
3348.02651	0.04064	3348.02651	0.10301	3348.02651	0.03911
3349.95509	0.04147	3349.95509	0.10508	3349.95509	0.03997
3351.88368	0.04231	3351.88368	0.10718	3351.88368	0.04084
3353.81227	0.04316	3353.81227	0.10931	3353.81227	0.04173
3355.74085	0.04402	3355.74085	0.11146	3355.74085	0.04264
3357.66944	0.0449	3357.66944	0.11363	3357.66944	0.04357
3359.59803	0.04579	3359.59803	0.11582	3359.59803	0.04451
3361.52661	0.04668	3361.52661	0.11803	3361.52661	0.04546
3363.4552	0.04759	3363.4552	0.12024	3363.4552	0.04643
3365.38379	0.0485	3365.38379	0.12245	3365.38379	0.0474
3367.31237	0.04942	3367.31237	0.12467	3367.31237	0.04838
3369.24096	0.05034	3369.24096	0.12688	3369.24096	0.04937
3371.16955	0.05127	3371.16955	0.12908	3371.16955	0.05036
3373.09813	0.05219	3373.09813	0.13125	3373.09813	0.05136
3375.02672	0.05312	3375.02672	0.13341	3375.02672	0.05235
3376.95531	0.05404	3376.95531	0.13553	3376.95531	0.05334
3378.88389	0.05495	3378.88389	0.13761	3378.88389	0.05433
3380.81248	0.05586	3380.81248	0.13965	3380.81248	0.05531
3382.74107	0.05676	3382.74107	0.14164	3382.74107	0.05628
3384.66965	0.05765	3384.66965	0.14357	3384.66965	0.05723
3386.59824	0.05853	3386.59824	0.14543	3386.59824	0.05818
3388.52683	0.05939	3388.52683	0.14722	3388.52683	0.0591
3390.45541	0.06024	3390.45541	0.14893	3390.45541	0.06
3392.384	0.06106	3392.384	0.15056	3392.384	0.06089
3394.31259	0.06187	3394.31259	0.1521	3394.31259	0.06175
3396.24117	0.06265	3396.24117	0.15354	3396.24117	0.06258
3398.16976	0.06341	3398.16976	0.15487	3398.16976	0.06338
3400.09835	0.06414	3400.09835	0.15611	3400.09835	0.06416
3402.02693	0.06484	3402.02693	0.15722	3402.02693	0.0649
3403.95552	0.06551	3403.95552	0.15823	3403.95552	0.0656
3405.88411	0.06615	3405.88411	0.15911	3405.88411	0.06627
3407.81269	0.06676	3407.81269	0.15987	3407.81269	0.06691
3409.74128	0.06734	3409.74128	0.16051	3409.74128	0.0675
3411.66987	0.06788	3411.66987	0.16102	3411.66987	0.06806
3413.59845	0.06838	3413.59845	0.16141	3413.59845	0.06857
3415.52704	0.06885	3415.52704	0.16166	3415.52704	0.06904
3417.45563	0.06928	3417.45563	0.16179	3417.45563	0.06947
3419.38421	0.06967	3419.38421	0.16178	3419.38421	0.06986

3421.3128	0.07003	3421.3128	0.16165	3421.3128	0.0702
3423.24139	0.07034	3423.24139	0.1614	3423.24139	0.0705
3425.16997	0.07062	3425.16997	0.16102	3425.16997	0.07076
3427.09856	0.07086	3427.09856	0.16052	3427.09856	0.07098
3429.02715	0.07106	3429.02715	0.1599	3429.02715	0.07115
3430.95573	0.07122	3430.95573	0.15917	3430.95573	0.07129
3432.88432	0.07135	3432.88432	0.15833	3432.88432	0.07138
3434.81291	0.07144	3434.81291	0.15738	3434.81291	0.07143
3436.74149	0.07149	3436.74149	0.15633	3436.74149	0.07144
3438.67008	0.07151	3438.67008	0.15519	3438.67008	0.07142
3440.59867	0.07149	3440.59867	0.15396	3440.59867	0.07136
3442.52725	0.07144	3442.52725	0.15264	3442.52725	0.07127
3444.45584	0.07135	3444.45584	0.15125	3444.45584	0.07114
3446.38443	0.07124	3446.38443	0.14978	3446.38443	0.07098
3448.31301	0.07109	3448.31301	0.14825	3448.31301	0.07079
3450.2416	0.07092	3450.2416	0.14666	3450.2416	0.07057
3452.17019	0.07072	3452.17019	0.14502	3452.17019	0.07033
3454.09877	0.07049	3454.09877	0.14333	3454.09877	0.07006
3456.02736	0.07023	3456.02736	0.14159	3456.02736	0.06977
3457.95595	0.06996	3457.95595	0.13982	3457.95595	0.06945
3459.88453	0.06966	3459.88453	0.13803	3459.88453	0.06912
3461.81312	0.06934	3461.81312	0.13621	3461.81312	0.06877
3463.74171	0.069	3463.74171	0.13437	3463.74171	0.0684
3465.67029	0.06864	3465.67029	0.13252	3465.67029	0.06801
3467.59888	0.06826	3467.59888	0.13066	3467.59888	0.06761
3469.52747	0.06787	3469.52747	0.12879	3469.52747	0.0672
3471.45605	0.06746	3471.45605	0.12693	3471.45605	0.06677
3473.38464	0.06704	3473.38464	0.12508	3473.38464	0.06634
3475.31323	0.06661	3475.31323	0.12323	3475.31323	0.0659
3477.24181	0.06616	3477.24181	0.12139	3477.24181	0.06544
3479.1704	0.0657	3479.1704	0.11957	3479.1704	0.06499
3481.09899	0.06524	3481.09899	0.11776	3481.09899	0.06452
3483.02758	0.06476	3483.02758	0.11598	3483.02758	0.06405
3484.95616	0.06427	3484.95616	0.11421	3484.95616	0.06357
3486.88475	0.06377	3486.88475	0.11247	3486.88475	0.06309
3488.81334	0.06327	3488.81334	0.11075	3488.81334	0.0626
3490.74192	0.06276	3490.74192	0.10905	3490.74192	0.06211
3492.67051	0.06224	3492.67051	0.10738	3492.67051	0.06161
3494.5991	0.06171	3494.5991	0.10574	3494.5991	0.06111
3496.52768	0.06117	3496.52768	0.10412	3496.52768	0.0606
3498.45627	0.06063	3498.45627	0.10252	3498.45627	0.06009
3500.38486	0.06008	3500.38486	0.10095	3500.38486	0.05958
3502.31344	0.05953	3502.31344	0.0994	3502.31344	0.05905
3504.24203	0.05896	3504.24203	0.09787	3504.24203	0.05853
3506.17062	0.05839	3506.17062	0.09636	3506.17062	0.058
3508.0992	0.05781	3508.0992	0.09487	3508.0992	0.05746
3510.02779	0.05722	3510.02779	0.09341	3510.02779	0.05691
3511.95638	0.05663	3511.95638	0.09195	3511.95638	0.05636
3513.88496	0.05602	3513.88496	0.09051	3513.88496	0.0558
3515.81355	0.05541	3515.81355	0.08909	3515.81355	0.05523
3517.74214	0.05479	3517.74214	0.08768	3517.74214	0.05465
3519.67072	0.05416	3519.67072	0.08627	3519.67072	0.05407
3521.59931	0.05352	3521.59931	0.08488	3521.59931	0.05347
3523.5279	0.05287	3523.5279	0.08349	3523.5279	0.05286
3525.45648	0.05221	3525.45648	0.08211	3525.45648	0.05224
3527.38507	0.05155	3527.38507	0.08073	3527.38507	0.05162

3529.31366	0.05087	3529.31366	0.07935	3529.31366	0.05098
3531.24224	0.05018	3531.24224	0.07798	3531.24224	0.05032
3533.17083	0.04948	3533.17083	0.07661	3533.17083	0.04966
3535.09942	0.04877	3535.09942	0.07523	3535.09942	0.04899
3537.028	0.04806	3537.028	0.07385	3537.028	0.0483
3538.95659	0.04733	3538.95659	0.07247	3538.95659	0.0476
3540.88518	0.04659	3540.88518	0.07109	3540.88518	0.04689
3542.81376	0.04584	3542.81376	0.0697	3542.81376	0.04616
3544.74235	0.04508	3544.74235	0.06831	3544.74235	0.04543
3546.67094	0.04431	3546.67094	0.06692	3546.67094	0.04468
3548.59952	0.04354	3548.59952	0.06552	3548.59952	0.04392
3550.52811	0.04275	3550.52811	0.06412	3550.52811	0.04315
3552.4567	0.04196	3552.4567	0.06271	3552.4567	0.04237
3554.38528	0.04116	3554.38528	0.0613	3554.38528	0.04158
3556.31387	0.04035	3556.31387	0.05988	3556.31387	0.04078
3558.24246	0.03953	3558.24246	0.05847	3558.24246	0.03997
3560.17104	0.03871	3560.17104	0.05705	3560.17104	0.03916
3562.09963	0.03788	3562.09963	0.05563	3562.09963	0.03833
3564.02822	0.03705	3564.02822	0.05421	3564.02822	0.0375
3565.9568	0.03622	3565.9568	0.05279	3565.9568	0.03667
3567.88539	0.03538	3567.88539	0.05138	3567.88539	0.03582
3569.81398	0.03454	3569.81398	0.04996	3569.81398	0.03498
3571.74256	0.03369	3571.74256	0.04856	3571.74256	0.03413
3573.67115	0.03285	3573.67115	0.04715	3573.67115	0.03327
3575.59974	0.032	3575.59974	0.04576	3575.59974	0.03242
3577.52832	0.03116	3577.52832	0.04437	3577.52832	0.03157
3579.45691	0.03032	3579.45691	0.04299	3579.45691	0.03071
3581.3855	0.02948	3581.3855	0.04163	3581.3855	0.02986
3583.31408	0.02864	3583.31408	0.04027	3583.31408	0.02901
3585.24267	0.02781	3585.24267	0.03893	3585.24267	0.02816
3587.17126	0.02698	3587.17126	0.0376	3587.17126	0.02732
3589.09984	0.02616	3589.09984	0.03629	3589.09984	0.02649
3591.02843	0.02535	3591.02843	0.035	3591.02843	0.02566
3592.95702	0.02454	3592.95702	0.03373	3592.95702	0.02483
3594.8856	0.02374	3594.8856	0.03247	3594.8856	0.02402
3596.81419	0.02296	3596.81419	0.03124	3596.81419	0.02321
3598.74278	0.02218	3598.74278	0.03003	3598.74278	0.02242

X Observed	Y Generated	X Observed	Y Generated
	Coal+TPPI		Coal+PA
3000.8809	2.9052E-05	3000.8809	8.7528E-05
3002.80949	3.2594E-05	3002.8095	9.6193E-05
3004.73807	3.6518E-05	3004.7381	0.0001056
3006.66666	4.0856E-05	3006.6667	0.00011579
3008.59525	4.5645E-05	3008.5953	0.00012682
3010.52383	5.0924E-05	3010.5238	0.00013874
3012.45242	5.6734E-05	3012.4524	0.00015161
3014.38101	6.3117E-05	3014.381	0.00016549
3016.30959	7.0121E-05	3016.3096	0.00018044
3018.23818	7.7792E-05	3018.2382	0.00019651
3020.16677	8.6181E-05	3020.1668	0.00021377
3022.09535	9.534E-05	3022.0954	0.00023229
3024.02394	0.00010533	3024.0239	0.00025212
3025.95253	0.00011619	3025.9525	0.00027333
3027.88111	0.000128	3027.8811	0.00029599
3029.8097	0.00014081	3029.8097	0.00032017
3031.73829	0.00015469	3031.7383	0.00034594
3033.66687	0.00016969	3033.6669	0.00037335
3035.59546	0.00018589	3035.5955	0.00040248
3037.52405	0.00020335	3037.5241	0.00043339
3039.45263	0.00022214	3039.4526	0.00046614
3041.38122	0.00024233	3041.3812	0.00050081
3043.30981	0.00026398	3043.3098	0.00053744
3045.23839	0.00028716	3045.2384	0.0005761
3047.16698	0.00031194	3047.167	0.00061684
3049.09557	0.00033838	3049.0956	0.00065972
3051.02415	0.00036656	3051.0242	0.00070478
3052.95274	0.00039653	3052.9527	0.00075208
3054.88133	0.00042835	3054.8813	0.00080164
3056.80991	0.00046208	3056.8099	0.0008535
3058.7385	0.00049778	3058.7385	0.0009077
3060.66709	0.0005355	3060.6671	0.00096425
3062.59567	0.00057527	3062.5957	0.00102
3064.52426	0.00061715	3064.5243	0.00108
3066.45285	0.00066117	3066.4529	0.00115
3068.38143	0.00070736	3068.3814	0.00121
3070.31002	0.00075575	3070.31	0.00128
3072.23861	0.00080634	3072.2386	0.00135
3074.16719	0.00085916	3074.1672	0.00143
3076.09578	0.00091421	3076.0958	0.0015
3078.02437	0.00097149	3078.0244	0.00158
3079.95295	0.00103	3079.953	0.00166
3081.88154	0.00109	3081.8815	0.00174
3083.81013	0.00116	3083.8101	0.00183
3085.73871	0.00122	3085.7387	0.00191
3087.6673	0.00129	3087.6673	0.002
3089.59589	0.00136	3089.5959	0.00209
3091.52447	0.00143	3091.5245	0.00218
3093.45306	0.00151	3093.4531	0.00228
3095.38165	0.00158	3095.3817	0.00237

3097.31024	0.00166	3097.3102	0.00247
3099.23882	0.00174	3099.2388	0.00256
3101.16741	0.00182	3101.1674	0.00266
3103.096	0.00191	3103.096	0.00276
3105.02458	0.00199	3105.0246	0.00287
3106.95317	0.00208	3106.9532	0.00297
3108.88176	0.00217	3108.8818	0.00307
3110.81034	0.00225	3110.8103	0.00318
3112.73893	0.00235	3112.7389	0.00328
3114.66752	0.00244	3114.6675	0.00339
3116.5961	0.00253	3116.5961	0.00349
3118.52469	0.00263	3118.5247	0.0036
3120.45328	0.00272	3120.4533	0.00371
3122.38186	0.00282	3122.3819	0.00382
3124.31045	0.00292	3124.3105	0.00393
3126.23904	0.00303	3126.239	0.00404
3128.16762	0.00313	3128.1676	0.00415
3130.09621	0.00324	3130.0962	0.00427
3132.0248	0.00334	3132.0248	0.00438
3133.95338	0.00345	3133.9534	0.0045
3135.88197	0.00357	3135.882	0.00462
3137.81056	0.00368	3137.8106	0.00474
3139.73914	0.0038	3139.7391	0.00486
3141.66773	0.00393	3141.6677	0.00499
3143.59632	0.00405	3143.5963	0.00512
3145.5249	0.00418	3145.5249	0.00525
3147.45349	0.00432	3147.4535	0.00538
3149.38208	0.00446	3149.3821	0.00552
3151.31066	0.0046	3151.3107	0.00567
3153.23925	0.00475	3153.2393	0.00581
3155.16784	0.0049	3155.1678	0.00597
3157.09642	0.00507	3157.0964	0.00613
3159.02501	0.00523	3159.025	0.00629
3160.9536	0.0054	3160.9536	0.00646
3162.88218	0.00558	3162.8822	0.00664
3164.81077	0.00577	3164.8108	0.00682
3166.73936	0.00596	3166.7394	0.00701
3168.66794	0.00616	3168.6679	0.00721
3170.59653	0.00637	3170.5965	0.00741
3172.52512	0.00658	3172.5251	0.00762
3174.4537	0.0068	3174.4537	0.00784
3176.38229	0.00702	3176.3823	0.00806
3178.31088	0.00725	3178.3109	0.00829
3180.23946	0.00749	3180.2395	0.00852
3182.16805	0.00773	3182.1681	0.00876
3184.09664	0.00798	3184.0966	0.00901
3186.02522	0.00823	3186.0252	0.00926
3187.95381	0.00848	3187.9538	0.00951
3189.8824	0.00874	3189.8824	0.00977
3191.81098	0.009	3191.811	0.01003
3193.73957	0.00926	3193.7396	0.0103
3195.66816	0.00953	3195.6682	0.01056
3197.59674	0.00979	3197.5967	0.01083
3199.52533	0.01005	3199.5253	0.01109
3201.45392	0.01031	3201.4539	0.01136
3203.3825	0.01057	3203.3825	0.01162

3205.31109	0.01083	3205.3111	0.01188
3207.23968	0.01108	3207.2397	0.01214
3209.16826	0.01133	3209.1683	0.0124
3211.09685	0.01157	3211.0969	0.01264
3213.02544	0.01181	3213.0254	0.01289
3214.95402	0.01204	3214.954	0.01313
3216.88261	0.01227	3216.8826	0.01336
3218.8112	0.01249	3218.8112	0.01359
3220.73978	0.01271	3220.7398	0.01381
3222.66837	0.01292	3222.6684	0.01403
3224.59696	0.01312	3224.597	0.01424
3226.52554	0.01332	3226.5255	0.01444
3228.45413	0.01352	3228.4541	0.01464
3230.38272	0.01371	3230.3827	0.01483
3232.3113	0.0139	3232.3113	0.01502
3234.23989	0.01408	3234.2399	0.01521
3236.16848	0.01426	3236.1685	0.01539
3238.09706	0.01444	3238.0971	0.01557
3240.02565	0.01462	3240.0257	0.01575
3241.95424	0.01479	3241.9542	0.01593
3243.88282	0.01497	3243.8828	0.0161
3245.81141	0.01516	3245.8114	0.01628
3247.74	0.01534	3247.74	0.01646
3249.66858	0.01553	3249.6686	0.01665
3251.59717	0.01572	3251.5972	0.01684
3253.52576	0.01592	3253.5258	0.01703
3255.45434	0.01612	3255.4543	0.01723
3257.38293	0.01634	3257.3829	0.01744
3259.31152	0.01656	3259.3115	0.01766
3261.2401	0.01679	3261.2401	0.01788
3263.16869	0.01702	3263.1687	0.01811
3265.09728	0.01727	3265.0973	0.01835
3267.02586	0.01753	3267.0259	0.0186
3268.95445	0.01779	3268.9545	0.01886
3270.88304	0.01807	3270.883	0.01913
3272.81162	0.01836	3272.8116	0.01941
3274.74021	0.01865	3274.7402	0.0197
3276.6688	0.01896	3276.6688	0.02
3278.59738	0.01928	3278.5974	0.02031
3280.52597	0.0196	3280.526	0.02063
3282.45456	0.01993	3282.4546	0.02095
3284.38314	0.02028	3284.3831	0.02129
3286.31173	0.02062	3286.3117	0.02163
3288.24032	0.02098	3288.2403	0.02198
3290.16891	0.02134	3290.1689	0.02234
3292.09749	0.02171	3292.0975	0.0227
3294.02608	0.02209	3294.0261	0.02308
3295.95467	0.02247	3295.9547	0.02345
3297.88325	0.02286	3297.8833	0.02383
3299.81184	0.02325	3299.8118	0.02422
3301.74043	0.02364	3301.7404	0.02462
3303.66901	0.02404	3303.669	0.02501
3305.5976	0.02444	3305.5976	0.02542
3307.52619	0.02485	3307.5262	0.02582
3309.45477	0.02526	3309.4548	0.02624
3311.38336	0.02568	3311.3834	0.02665

3313.31195	0.0261	3313.312	0.02708
3315.24053	0.02653	3315.2405	0.02751
3317.16912	0.02696	3317.1691	0.02794
3319.09771	0.02739	3319.0977	0.02839
3321.02629	0.02784	3321.0263	0.02884
3322.95488	0.02829	3322.9549	0.0293
3324.88347	0.02875	3324.8835	0.02977
3326.81205	0.02922	3326.8121	0.03025
3328.74064	0.02969	3328.7406	0.03074
3330.66923	0.03018	3330.6692	0.03124
3332.59781	0.03068	3332.5978	0.03176
3334.5264	0.03119	3334.5264	0.03229
3336.45499	0.03172	3336.455	0.03283
3338.38357	0.03225	3338.3836	0.03339
3340.31216	0.03281	3340.3122	0.03396
3342.24075	0.03337	3342.2408	0.03455
3344.16933	0.03395	3344.1693	0.03516
3346.09792	0.03455	3346.0979	0.03578
3348.02651	0.03516	3348.0265	0.03642
3349.95509	0.03578	3349.9551	0.03708
3351.88368	0.03643	3351.8837	0.03775
3353.81227	0.03708	3353.8123	0.03845
3355.74085	0.03776	3355.7409	0.03916
3357.66944	0.03844	3357.6694	0.03988
3359.59803	0.03914	3359.598	0.04062
3361.52661	0.03986	3361.5266	0.04138
3363.4552	0.04058	3363.4552	0.04215
3365.38379	0.04132	3365.3838	0.04293
3367.31237	0.04206	3367.3124	0.04373
3369.24096	0.04281	3369.241	0.04453
3371.16955	0.04357	3371.1696	0.04534
3373.09813	0.04433	3373.0981	0.04616
3375.02672	0.04509	3375.0267	0.04698
3376.95531	0.04586	3376.9553	0.04781
3378.88389	0.04662	3378.8839	0.04863
3380.81248	0.04738	3380.8125	0.04946
3382.74107	0.04813	3382.7411	0.05027
3384.66965	0.04887	3384.6697	0.05109
3386.59824	0.0496	3386.5982	0.05189
3388.52683	0.05032	3388.5268	0.05268
3390.45541	0.05102	3390.4554	0.05346
3392.384	0.05171	3392.384	0.05423
3394.31259	0.05237	3394.3126	0.05497
3396.24117	0.05302	3396.2412	0.0557
3398.16976	0.05363	3398.1698	0.0564
3400.09835	0.05423	3400.0984	0.05708
3402.02693	0.05479	3402.0269	0.05773
3403.95552	0.05533	3403.9555	0.05836
3405.88411	0.05583	3405.8841	0.05895
3407.81269	0.05631	3407.8127	0.05952
3409.74128	0.05675	3409.7413	0.06005
3411.66987	0.05715	3411.6699	0.06055
3413.59845	0.05752	3413.5985	0.06101
3415.52704	0.05786	3415.527	0.06144
3417.45563	0.05816	3417.4556	0.06183
3419.38421	0.05842	3419.3842	0.06218

3421.3128	0.05864	3421.3128	0.0625
3423.24139	0.05883	3423.2414	0.06278
3425.16997	0.05899	3425.17	0.06302
3427.09856	0.05911	3427.0986	0.06322
3429.02715	0.05919	3429.0272	0.06339
3430.95573	0.05924	3430.9557	0.06353
3432.88432	0.05926	3432.8843	0.06362
3434.81291	0.05925	3434.8129	0.06369
3436.74149	0.0592	3436.7415	0.06372
3438.67008	0.05913	3438.6701	0.06371
3440.59867	0.05903	3440.5987	0.06368
3442.52725	0.05891	3442.5273	0.06361
3444.45584	0.05876	3444.4558	0.06352
3446.38443	0.05859	3446.3844	0.0634
3448.31301	0.05839	3448.313	0.06326
3450.2416	0.05818	3450.2416	0.06309
3452.17019	0.05795	3452.1702	0.06289
3454.09877	0.0577	3454.0988	0.06268
3456.02736	0.05744	3456.0274	0.06245
3457.95595	0.05717	3457.956	0.06219
3459.88453	0.05689	3459.8845	0.06193
3461.81312	0.05659	3461.8131	0.06164
3463.74171	0.05629	3463.7417	0.06135
3465.67029	0.05598	3465.6703	0.06104
3467.59888	0.05566	3467.5989	0.06072
3469.52747	0.05534	3469.5275	0.06038
3471.45605	0.05501	3471.4561	0.06004
3473.38464	0.05468	3473.3846	0.0597
3475.31323	0.05435	3475.3132	0.05934
3477.24181	0.05402	3477.2418	0.05898
3479.1704	0.05368	3479.1704	0.05861
3481.09899	0.05334	3481.099	0.05824
3483.02758	0.053	3483.0276	0.05786
3484.95616	0.05266	3484.9562	0.05748
3486.88475	0.05232	3486.8848	0.05709
3488.81334	0.05197	3488.8133	0.0567
3490.74192	0.05162	3490.7419	0.05631
3492.67051	0.05127	3492.6705	0.05591
3494.5991	0.05092	3494.5991	0.0555
3496.52768	0.05057	3496.5277	0.0551
3498.45627	0.0502	3498.4563	0.05469
3500.38486	0.04984	3500.3849	0.05427
3502.31344	0.04947	3502.3134	0.05385
3504.24203	0.04909	3504.242	0.05342
3506.17062	0.04871	3506.1706	0.05298
3508.0992	0.04831	3508.0992	0.05254
3510.02779	0.04791	3510.0278	0.05209
3511.95638	0.0475	3511.9564	0.05163
3513.88496	0.04708	3513.885	0.05117
3515.81355	0.04665	3515.8136	0.05069
3517.74214	0.04621	3517.7421	0.05021
3519.67072	0.04575	3519.6707	0.04971
3521.59931	0.04528	3521.5993	0.04921
3523.5279	0.0448	3523.5279	0.04869
3525.45648	0.0443	3525.4565	0.04816
3527.38507	0.04379	3527.3851	0.04762

3529.31366	0.04326	3529.3137	0.04707
3531.24224	0.04272	3531.2422	0.0465
3533.17083	0.04217	3533.1708	0.04592
3535.09942	0.04159	3535.0994	0.04533
3537.028	0.04101	3537.028	0.04472
3538.95659	0.0404	3538.9566	0.04411
3540.88518	0.03978	3540.8852	0.04347
3542.81376	0.03915	3542.8138	0.04283
3544.74235	0.0385	3544.7424	0.04217
3546.67094	0.03784	3546.6709	0.0415
3548.59952	0.03716	3548.5995	0.04082
3550.52811	0.03647	3550.5281	0.04012
3552.4567	0.03577	3552.4567	0.03942
3554.38528	0.03506	3554.3853	0.0387
3556.31387	0.03434	3556.3139	0.03797
3558.24246	0.0336	3558.2425	0.03724
3560.17104	0.03286	3560.171	0.03649
3562.09963	0.0321	3562.0996	0.03574
3564.02822	0.03134	3564.0282	0.03497
3565.9568	0.03058	3565.9568	0.0342
3567.88539	0.02981	3567.8854	0.03343
3569.81398	0.02903	3569.814	0.03265
3571.74256	0.02825	3571.7426	0.03186
3573.67115	0.02747	3573.6712	0.03107
3575.59974	0.02669	3575.5997	0.03028
3577.52832	0.02591	3577.5283	0.02949
3579.45691	0.02513	3579.4569	0.0287
3581.3855	0.02436	3581.3855	0.02791
3583.31408	0.02358	3583.3141	0.02712
3585.24267	0.02282	3585.2427	0.02633
3587.17126	0.02205	3587.1713	0.02555
3589.09984	0.0213	3589.0998	0.02477
3591.02843	0.02055	3591.0284	0.024
3592.95702	0.01981	3592.957	0.02323
3594.8856	0.01908	3594.8856	0.02247
3596.81419	0.01836	3596.8142	0.02172
3598.74278	0.01766	3598.7428	0.02097
